# PTR-ToF-MS VOC Profiling of Raw and Cooked Gilthead Sea Bream Fillet (*Sparus aurata*): Effect of Rearing System, Season, and Geographical Origin

**DOI:** 10.3390/molecules30020402

**Published:** 2025-01-18

**Authors:** Iuliia Khomenko, Valentina Ting, Fabio Brambilla, Mirco Perbellini, Luca Cappellin, Franco Biasioli

**Affiliations:** 1 Research and Innovation Centre, Fondazione Edmund Mach, 38098 San Michele all’Adige, TN, Italy; franco.biasioli@fmach.it; 2 Independent Researcher, 38098 San Michele all’Adige, TN, Italy; valentinating.888@gmail.com; 3 VRM S.r.l. Naturalleva, 37044 Cologna Veneta, VR, Italy; fabio_brambilla@naturalleva.it (F.B.); mirco_perbellini@naturalleva.it (M.P.); 4 Department of Chemical Sciences, University of Padua, 35131 Padua, PD, Italy; luca.cappellin@unipd.it

**Keywords:** volatile organic compounds, fish, proton transfer reaction–mass spectrometry, automation

## Abstract

This study explores the impact of geographical origin, harvest time, and cooking on the volatile organic compound (VOC) profiles of wild and reared seabream from the Adriatic and Tyrrhenian Seas. A Proton Transfer Reaction–Time of Flight–Mass Spectrometry (PTR-ToF-MS) allowed for VOC profiling with high sensitivity and high throughput. A total of 227 mass peaks were identified. Principal component analysis (PCA) showed a clear separation between cooked and raw samples, with cooking causing a significant increase in 64% of VOCs, especially hydrogen sulphide, methanethiol, and butanal. A two-way ANOVA revealed significant effects of origin, time, and their interaction on VOC concentration, with 102 mass peaks varying significantly based on all three factors. Seasonal effects were also notable, particularly in reared fish from the Adriatic Sea, where compounds like monoterpenes and aromatics were higher during non-breeding months, likely due to environmental factors unique to that area. Differences between wild and reared fish were influenced by lipid content and seasonal changes, impacting the VOC profile of seabream. These findings provide valuable insights into how cooking, geographical origin, and seasonality interact to define the flavour profile of seabream, with potential applications in improving quality control and product differentiation in seafood production.

## 1. Introduction

In recent decades, an increase in the consumption of aquatic foods has become apparent. With an average annual increase of 3% globally (from 1961 to 2019), the consumption rate increase is almost double that of the annual global population growth (1.6%) for the same timeline. This increase in consumption can be attributed to greater supply, higher household incomes, technological advances, health claims, and a shift in consumer preference [1]. Gilthead sea bream (*Sparus aurata*) is one of the main marine fish species reared around the Mediterranean coast. Worldwide, it is the fourth highest in production species, amounting to 282.1 thousand tons per year as of 2020, only behind Atlantic salmon, milkfish and mullet [2]. Therefore, gilthead sea bream has emerged as a crucial species over the past decade and has been the subject of extensive study based on aquaculture practices [3] and quality improvement.

Flavour and aroma are among the most important factors in determining the freshness and quality of fish. The mild, delicate flavours and aromas present in very fresh fish are distinguished by their green, mild, and herbal qualities, which are easily identifiable and commonly associated with fresh seafood [4]. These characteristics arise from a complex mixture of VOCs like 6-, 8-, and 9-carbon aldehydes, ketones, and alcohols, which are produced from long-chain polyunsaturated fatty acids via specific lipoxygenase activity and oxidation [4]. However, other factors, including microbial metabolism, environmental conditions, enzymatic reactions, and protein degradation, also contribute to the production of VOCs in fish flesh [5]. These types of reactions produce different classes of VOCs, such as carbonyls, aldehydes, ketones, alcohols, aromatics, alkanes, terpenes, esters, and pyrazines, which are also linked to freshness and quality traits [6]. While these compounds play a role in defining the unique flavour of fish, a significant number of them can be responsible for a transition into the undesirable ‘fishy odour’, which is dependent on their concentration and odour threshold values [7].

Many studies have explored the VOCs present in sea bream, with a focus on those of microbiological and bacterial origin. These compounds play a pivotal role in the degradation of fish, causing off-flavours/off-odours and finally the organoleptic rejection of the product [8]. However, in recent years, according to the goals of sustainable aquaculture development, research has shifted to the study of VOCs that differentiate the wild and reared sea breams and, more generally, to the effect of production and technological parameters on fish quality. These studies can provide insight into the origin of a more desirable aroma of wild sea bream according to consumer preferences [7]. In general, the meat of reared fish tends to be softer in texture, with a milder, less robust flavour compared to wild sea bream, which could be attributed to higher fat content and lower swimming intensity [9]. Additionally, the colour and aroma of reared meat are somewhat determined not just by physical activity but also certain organisms and algae within the growing environment that affect the fish [10].

Several studies have noted a more intense aromatic profile with higher amounts of VOCs, both in the cooked and raw wild fish of different species compared to the reared fish, showing a distinct difference between the two [7,11,12,13]. Additionally, the geographical origin of the fish is another factor that influences its aromatic profile: different concentrations of VOCs are found in both wild and reared fish according to the geographical area. However, sometimes no differences are observed, as in the study of reared sea bass from two different geographical origins [14]. 

Numerous studies have explored the VOC profile of cooked and/or raw fish using gas chromatography (GC) involving extensive sample preparation and lengthy analysis times [6,7,9]. Aiming to improve throughput, this study uses the Proton Transfer Reaction–Time of Flight–Mass Spectrometry (PTR-ToF-MS), equipped with a multipurpose sampler to measure VOCs in real time down to low concentrations in a rapid (1 min per sample) and non-invasive way. This method has been successfully used on meat products [15] and represents the initial exploration in gilthead seabream, where the VOC profile of the same sample is analyzed in its raw state and then reanalysed after cooking in the same sequence of analysis. This allows for a more robust understanding of how the cooking process alters the VOC profile. Other factors explored include a comparison between different geographical origins, harvest times in different seasons, and farming methods (wild or reared).

## 2. Results and Discussion

### 2.1. Differences Between Raw and Cooked Sea Bream

**Figure 1:**
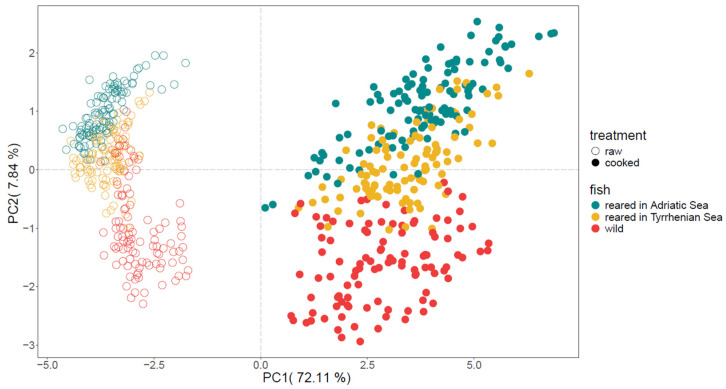
Principal component analysis (PCA) on the measured VOC concentration for raw and cooked wild sea bream that were reared in either the Adriatic, Tyrrhenian, or Levant Sea.

A total of 227 mass peaks were identified that significantly differentiated between the raw and cooked sea bream samples. The plots of all mass peaks are listed in Supplementary Figure S1. After cooking, 64% of the total mass peaks increased significantly in concentration. Raw fresh fish is known to have a very mild aroma, which intensifies after thermal degradation, in which the aroma profile is highly dependent on the fish species and cooking procedure [9,16]. The score plot in Figure 1 and loading plot (Supplementary Figure S2) illustrate this distinct separation between the cooked and raw fish samples from both wild and reared fish (originating from the Adriatic and Tyrrhenian Seas) using principal component analysis (PCA). The cooked fish samples are positioned on the positive side of PC1, while the raw fish samples are on the negative side of PC1, with an explained variance of 79.95% through PC1 and PC2.

The major influence of cooking was observed in mass peaks such as *m*/*z* 34.995 (H_2_SH^+^—t.i. hydrogen sulphide), *m*/*z* 49.011 (CH_4_SH^+^—t.i. methanethiol), *m*/*z* 83.086 (C_6_H_11_^+^—t.i. hexenol), *m*/*z* 46.038 (C^13^CH_4_OH^+^—t.i. isotope of acetaldehyde), *m*/*z* 59.049 (C_3_H_6_OH^+^—t.i. acetone), and *m*/*z* 33.034 (CH_4_OH^+^—t.i. methanol). This increase in VOC concentration arises from several reactions that take place during the cooking process, including lipid hydrolysis, lipid oxidation, and the degradation of nitrogen-containing compounds, such as proteins and amino acids via Maillard-type or similar reactions [16,17,18].

**Figure 2:**
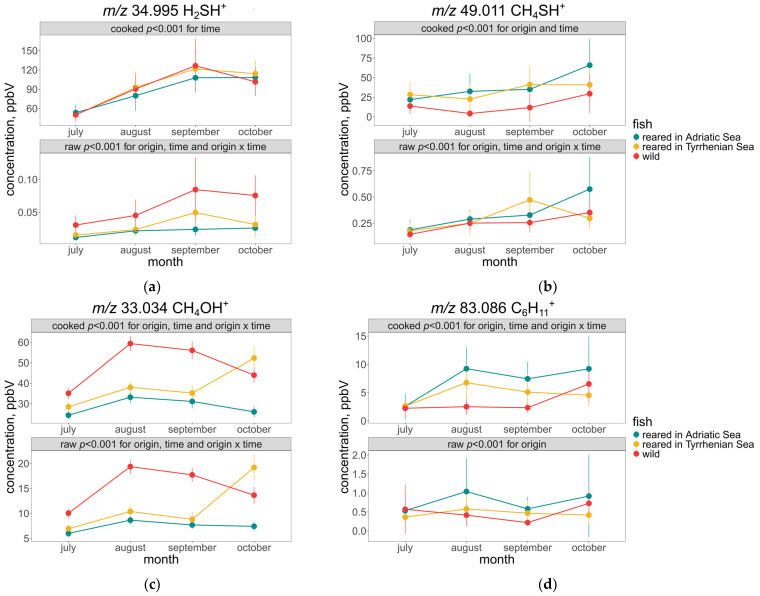
Differences in t.i. (**a**) hydrogen sulphide (*m*/*z* 34.995) (mean ± SD), (**b**) methanethiol (*m*/*z* 49.011), (**c**) methanol, and (**d**) hexenol (*m*/*z* 83.086) between cooked and raw fish samples and the level of significance according to a two-way ANOVA of geographical origin and time of harvest.

Figure 2a illustrates the differences in t.i. hydrogen sulphide concentration before and after cooking, which is in agreement with previous studies of the formation of this compound during amino acid and protein degradation for other fish species, like salmon [17] and tuna [18,19]. Moreover, despite the low concentration in the raw samples, the two-way ANOVA showed significant differences (*p* < 0.001) in time, origin, and their interaction between fish samples of different origin. In the cooked samples, the differences in origin were less evident, but the same increasing trend in time for the first three months, with the slight decrease in October, was observed, as well as in raw samples. Other compounds that showed similar behaviour include t.i. methanethiol (Figure 2b) and methanol (Figure 2c). Interestingly, although similar in trends, the scale is vastly different between raw and cooked fish, indicating a large increase in these compounds due to the cooking process.

In fish, methanethiol, along with hydrogen sulphide, is formed from proteins and lipids through thermal degradation and enzymatic reactions. This compound contributes to the aroma and flavour profile of cooked fish with a distinct seafood odour. In this study, the release of methanethiol and hydrogen sulphide occurred, with both sulphur-containing compounds produced during the cooking process of proteins through the breakdown of methionine and cysteine, respectively. Like methanethiol, hydrogen sulphide also contributes to the odour of cooked fish. It is released from the sulphur-containing amino acids, such as cysteine and methionine, during thermal processing [16]. The presence of methanol is usually found in pectin-rich products such as vegetables. It has a relatively low odour threshold and does not contribute to the aroma of cooked food [20]. However, studies have detected its presence as a byproduct of thermal degradation with significant relevance to meat flavour [15,21], which also increased in this study after cooking.

Among the higher alcohols tentatively identified in this study, hexenol (Figure 2d) showed the biggest increase in concentration after cooking. Its formation could be explained by a lipoxygenase-initiated peroxidation of the n-3 and n-6 polyunsaturated fatty acid [6].

### 2.2. Differences Between Wild and Reared Sea Bream

Numerous studies have shown differences between wild and reared fish. Grigorakis et al. [9] reported wild sea bream to contain a higher number of taste-contributing compounds compared to the reared fish after cooking. Focusing on Figure 1, a clear separation is observed, with the wild sea bream on the negative side of PC2 compared to the reared sea bream on the positive side of PC2. Amongst the compounds that drive this separation, the t.i. butanal (*m*/*z* 73.066) was generally higher in concentration for the majority of the harvest months compared to the reared sea bream (Figure 2b). Other mass peaks with higher concentrations in wild fish samples include *m*/*z* 65.022 (C_2_H_6_^34^SH^+^—t.i. isotope of dimethyl sulphide) and *m*/*z* 91.074 (C_4_H_10_OH_2_H^+^—t.i. butanediol). On the contrary, some mass peaks, such as *m*/*z* 143.144 (C_9_H_18_OH^+^—t.i. hexenyl acetate), *m*/*z* 87.0811 (C_5_H_10_OH^+^—t.i. methyl butanal), and *m*/*z* 97.0654 (C_6_H_8_OH^+^—t.i. hexadienal), showed an opposite trend where the concentration of reared fish, regardless of origin and time, were significantly higher. The seasonal fluctuations observed here could be attributed to changes in muscle and fat content. An increase in fat deposits in late summer and early autumn was found to be associated with feeding intensity. In the case of wild fish, these changes may also relate to gonadal maturation and spawning activities. In terms of aroma and volatile compounds, these seasonal changes likely influence the overall sensory profile of the fish. For instance, higher fat content during late summer and early autumn may result in a richer and more pronounced aroma and flavour, while lower fat content after winter may yield a lighter and fresher aroma profile. Additionally, the differences observed between wild and reared gilthead sea bream, such as variations in lipid content and fatty acid profiles, can further impact the aroma composition and overall flavour characteristics of the fish. These differences in aroma and volatile compounds contribute to the distinct sensory experiences associated with wild and reared sea bream [22,23].

Lipid oxidation aldehydes and alcohols were observed in low concentrations, indicative of the freshness of both fish types. Butanal appears in a higher concentration in the raw fish and increases after cooking, compared to benzyl alcohol. In a study conducted by Vidal et al. [24], butanal was only found in the wild sea bass, and it was noted that butanal may originate from microbial processes acting on aliphatic or aromatic amino acids, which are found in higher levels in wild fish samples, aligning with our study. These microorganisms are likely responsible for generating these compounds, which are less common in farmed fish compared to their wild counterparts.

### 2.3. Differences Between Geographical Origin and Harvest Month

**Figure 3:**
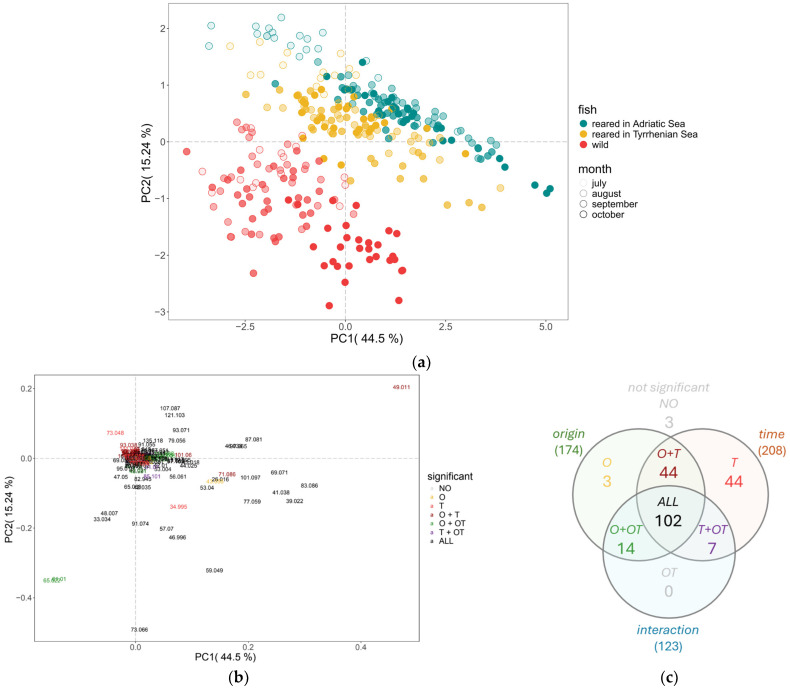
The score plot (**a**) and loading plot (**b**) of the principal component analysis (PCA) on the measured VOC concentration for cooked wild sea bream from the Levant Sea, and cooked sea bream reared in either the Adriatic or Tyrrhenian Sea. The different colours in the score plot (**a**) show the geographical origin reported in the legend, and colour shades indicate the months in which the fish were harvested. The colours of the loading plot (**b**) correspond to the classification according to the two-way ANOVA results presented, as well as those in the Venn diagram (**c**).

Geographical differences and harvest month effects for the three fish types were compared. A clear difference between wild and reared sea bream at different states (raw and cooked) was observed in Figure 1; therefore, it would be interesting to zoom into the differences in VOC concentration of the cooked sea bream samples. Figure 3a is a PCA on the VOC concentrations of the cooked wild and reared sea bream samples with an explained variance of 59.74% from PC1 and PC2. In this study, the VOC profile of cooked wild sea bream is shown to be significantly different compared to the cooked reared sea bream; however, sea bream reared in either the Adriatic or Tyrrhenian Sea were more similar. To investigate if there were specific VOCs that could be used to differentiate the fish samples from different geographical origins, specific compounds were selected. A two-way ANOVA was performed to understand the effects of time and geographical origin, as well as possible interactions between these factors. Significant differences (*p* < 0.001) were observed in 174 mass peaks for origin, 208 mass peaks for time, and 123 mass peaks for the interaction between origin and time. Three mass peaks were not significantly different for any factor (Figure 3b).

In Figure 3c, a Venn diagram summarizes the results obtained from the two-way ANOVA performed based on two independent variables: the geographical origins of the seabream (O) and the time of harvest by month (T), along with their interaction (OT). The interactions between O and T represent a combined effect between how geographical origin and time of harvest may affect changes in VOC concentration. The results highlight how geographical origin and time of harvest each independently affect certain VOCs in cooked fish, while other VOCs are influenced by both factors (interaction effect). As shown in the Venn diagram (Figure 3c), 3 mass peaks differed only by origin (O), 44 mass peaks only by time (T), 44 mass peaks by both origin and time (O + T), 14 mass peaks by origin and its interaction with time (O + OT), 7 mass peaks by time and its interaction with origin (T + OT), and 102 mass peaks by origin, time, and their interaction (ALL).

Overall, these findings underscore that both geographical origin and harvest timing significantly influence the VOC profile of seabream, providing insight into how these factors can affect the sensory qualities of the fish. A full list of compounds and their significant levels can be found in Supplementary Table S1.

**Figure 4:**
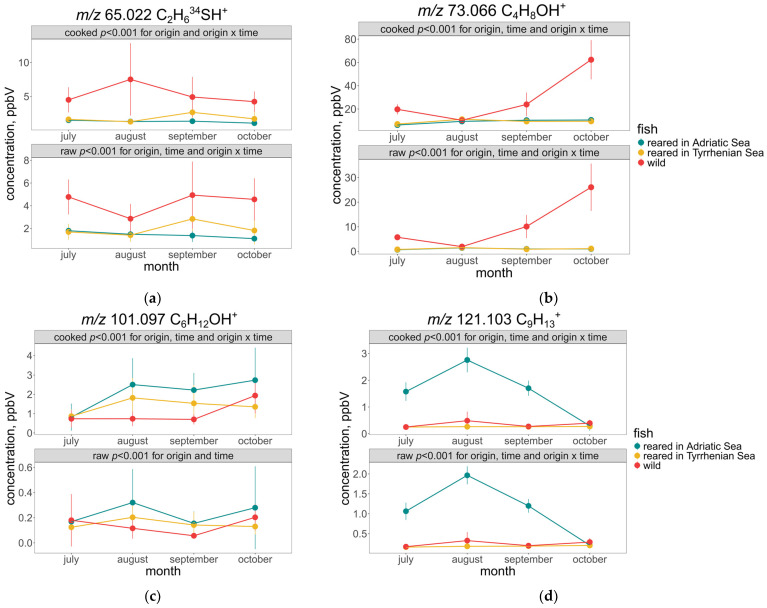
Selected mass peaks (mean ± SD), which distinguish different fish geographical origins, are plotted for three fish types under two conditions (cooked and raw). The two selected mass peaks are tentatively identified as (**a**) an isotope of dimethyl sulphide (*m*/*z* 65.022); (**b**) 2-Methyl propanal and butanal (*m*/*z* 73.066); (**c**) hexanal (*m*/*z* 101.097); and (**d**) 1,2,4-Trimethylbenzene, 1,3,5-Trimethylbenzene, 1-Ethyl-2-methylbenzene, and Propylbenzene (*m*/*z* 121.103).

Diving deeper into differences in seabream based on geographical origin, it was observed that fish reared in the Adriatic Sea displayed significantly distinct patterns for several classes of VOCs, like sulphur-containing compounds, aldehydes, aromatic compounds, and monoterpenes. In contrast to two sulphur-containing compounds described before (Figure 2a,c), t.i. dimethyl sulphide (Figure 4c), with its saltwater fish odour [12], was already present in raw samples and was constantly higher in wild samples. Its formation in fish flesh might have various origins, like food storage, the oxidation of methanethiol, bacteria contamination [13], and dietary history [12]. Since all samples were treated in the same manner, it is possible to assume that the fish diet played a major role in its formation.

Various aldehydes tentatively identified in this study can have different origins. Figure 4b shows that 2-Methyl propanal and butanal were higher in wild fish with the constant increase during autumn months. Vidal et al. found a higher concentration of butanal in wild sea bass samples compared to the reared ones and explained its presence as a result of microbial activity on aliphatic or aromatic amino acids [24]. Previous studies showed the differences in fatty acid profiles of wild and reared sea bream [9], which can explain the differences in some VOCs like t.i. hexanal (Figure 4c). This aldehyde is usually formed during the oxidation of n-6 fatty acids and usually found in different fresh saltwater fish samples [25]. In this study, the hexanal level was low in the raw fish and increased with cooking, and was higher in reared fish samples, especially those from the Adriatic Sea. Grigorakis et al. found higher levels of hexanal in wild sea bream [9]. On the contrary, Vidal et al. reported higher levels in reared sea bass, explaining that the levels of the molar percentage of linoleic groups in reared samples is much higher than in wild ones [24].

For the aromatic compounds such as *m*/*z* 121.103 (C_9_H_13_^+^—t.i. 1,2,4-Trimethylbenzene, 1,3,5-Trimethylbenzene, 1-Ethyl-2-methylbenzene# and Propylbenzene) (Figure 4b), and aromatic monoterpenoids such as *m*/*z* 133.102 (C_10_H_13_^+^) and 135.118 (C_10_H_15_^+^) and 147.118 (C_11_H_15_^+^), the highest concentrations of these compounds were found in the summertime during the non-breeding season (August–September) [26], and the VOCs gradually declined to reach their lowest concentration in October, as the seasons changed to autumn.

The low VOC concentrations detected signify the sensitivity of the PTR-ToF-MS; nonetheless, these differences significantly differentiate reared fish from the Adriatic Sea from fish of other geographical origins. Aromatic compounds are usually associated with the metabolism of carotenoids or the thermal degradation of sugars and amino acids [9]. Moreover, the effect of cooking on the compounds mentioned above was minimal (~1.5–2 fold) in comparison to Section 3.1. The differences shown in fish reared in the Adriatic Sea could be due to environmental factors that are specific to that area. Excess aromatic compounds such as 1,2,4-Trimethylbenzene, 1,3,5-Trimethylbenzene, 1-Ethyl-2-methylbenzene, and Propylbenzene may also indicate elevated levels of pollutants due to the enclosed nature of the Adriatic Sea, its shallow average depth (relative to the Tyrrhenian and Levant Seas [27]), and its proximity to a greater number of pollution hotspots [28].

With regard to harvest time, wild seabream and seabream reared in the Adriatic Sea shift from negative to positive PC1 as harvest months progress from July to October. This could be indicative of what was described in earlier sections, showing a rise in certain compounds during that month.

## 3. Materials and Methods

### 3.1. Fish Samples

Details about the gilthead sea bream (*Sparus aurata*) samples are provided in Table 1. All fish samples were slaughtered by the ice-killing method. After slaughtering, fish were kept on ice for 1 day (24 h) during transportation to the laboratory and until sample preparation. The room temperature of the laboratory was 10 °C.

### 3.2. Sample Preparation

In this study, a total of 108 sea bream were analyzed, with ten fish selected from each category (reared in Adriatic Sea, reared in Tyrrhenian Sea, and wild) and harvest date. An analysis was performed on both the raw and cooked dorsal fillet of the sea bream. Triplicate analysis was performed on each fish, with 3 g of dorsal fillet inserted into 22 mL vials at 10 °C and then stored at −80 °C prior to analysis. A total of 324 raw fish vials were prepared. Before sample measurement, the vials of raw fish were thawed at 4 °C, then incubated at 25 °C for 25 min and measured using the PTR-ToF-MS for 1 min to assess the VOC content of the raw fish. Cooking was then simulated by incubating the vial for 25 min at 70 °C, the temperature recommended for the thermal treatment of meat according to European Union regulations [30]. The vial was then cooled for 25 min at 25 °C, followed by a 1 min measurement, similarly to the raw sample.

As previously described, the 324 raw fish samples were measured, followed by incubation to obtain measurements for cooked fish samples, resulting in a total of 648 samples measured (324 raw and 324 cooked fish samples). The sample order was randomized to decrease the occurrence of memory effects. This also included a 5 min measurement of zero air after each sample. The generation of zero air was achieved via a catalytic VOC scrubber (GCU unit, Ionicon Analytik GmbH, Innsbruck, Austria).

### 3.3. Volatile Organic Compound Analysis by PTR-ToF-MS

The dynamic headspace of the raw and cooked fish samples were measured through a direct injection into the PTR-ToF-MS 8000 (Ionicon Analytik, GmbH, Innsbruck, Austria) coupled with a modified GC auto-sampler (MPS Multipurpose Sampler, Gerstel, Mülheim an der Ruhr, Germany) to automate and standardize measurements. Here, the autosampler was equipped with thermostatic trays used to control the temperature of the vials during measurement and a Purge Tool (Gerstel, Mülheim an der Ruhr, Germany) for the dynamic headspace measurements.

Vials were sampled using a Purge Tool by flushing zero air (40 sccm for 60 s) into the vial through a heated (40 °C) inlet needle. The headspace was delivered through an outflow needle (40 °C) into the PTR-ToF-MS inlet. Teflon fittings were used as connectors between the needle and the PTR-ToF-MS inlet. 

The PTR-ToF-MS conditions were configured in accordance with Farneti et al. [31] using the standard operating conditions of drift voltage 628 V, drift pressure 2.8 mbar, drift tube temperature 110 °C, and E/N 130 Td (Td: Townsend; 1 Td = 10^−17^ V cm^2^) in the primary ion mode (H_3_O^+^) throughout the experiment. With a sampling time of 0.1 ns per channel, each acquisition amounted to 350,000 channels, resulting in a mass spectrum range of *m*/*z* 21–400. Each sample was analyzed for 1 min.

### 3.4. Data Extraction and Statistical Analysis

The example of PTR-ToF-MS spectra are shown in Supplementary Figure S3. PTR-ToF-MS spectra were processed according to the methodology reported by Cappellin et al. [32], with slight modifications. As the first step of data processing, signal distortions related to the detector dead time were accounted for using a correction approach based on the Poisson statistics, according to Cappellin et al. [33]. Because the external calibration provided by the acquisition software did not achieve a sufficient mass accuracy, internal mass calibration was carried out, achieving a mass accuracy of greater than 0.001 Th. Subsequent data-processing steps, including noise reduction, baseline removal, and peak intensity extraction, were performed using modified Gaussians to fit spectral peaks by in-house software [32]. Headspace VOC concentrations, expressed as ppbv (parts per billion by volume), were estimated from the integrated signal over 20 s of spectra acquisition using the formula described by Lindinger et al. [34], considering hydronium (H_3_O^+^) as the primary ion and a constant reaction rate coefficient of 2 × 10^−9^ cm^3^/s in the calculations. This approach introduces a systematic error of up to 30% that can be accounted for if the actual rate coefficient is known [32].

From the total dataset of blank samples and raw and cooked fish samples, 383 mass peaks were extracted. A one-way analysis of variance (ANOVA) was applied to select mass peaks whose concentrations were statistically higher (*p* < 0.001) in fish samples rather than in blank ones. Mass peaks belonging to isotopologues and water clusters were excluded from the dataset. After this procedure, the dataset was reduced to 227 mass peaks, which were used for the further data analysis. The mass peak list is provided in Supplementary Table S1.

A two-way ANOVA was used to investigate the differences in tentatively identified VOC profiles of the fish samples based on their origin and time effects for raw and cooked fish. Principal component analysis (PCA) was applied on data that was log transformed and mean centred. All analyses were performed in R 4.2.3 using libraries such as ggplot2 (ver. 3.5.0), agricolae (ver. 1.3–5), mixOmics (ver. 6.22.0), and VennDiagram (ver. 1.7.3).

## 4. Conclusions

The findings in this paper reveal that both geographical origin and harvest time play critical roles in shaping the VOC profile of seabream, with distinct differences observed between wild and reared fish. Cooking significantly amplified the concentrations of certain VOCs, such as methanethiol and hydrogen sulphide, which impart characteristic seafood aromas and flavour. The influence of geographical origin was most evident in reared fish, particularly those reared in the Adriatic Sea, where monoterpenes and aromatic compounds reached peak concentrations during non-breeding seasons. This pattern may reflect the enclosed nature and shallow depth of the Adriatic Sea, as well as potential exposure to regional pollutants. Seasonal fluctuations, linked to the fat and muscle content in fish, further impact VOCs, underscoring the dynamic sensory qualities across different harvest months. The results in this paper emphasize the importance of origin and seasonality in defining the VOC profile of seabream, supporting potential applications in food production and quality control. Additionally, the marked VOC differences between wild and reared seabream provide a framework for better aligning product characteristics with consumer preferences, which can potentially be used to improve quality control and product differentiation amongst other seafood products.

## Figures and Tables

**Table 1:** Sea bream samples used in the study categorized by their geographical origin and date of harvest.

Sample Name	Reared in Adriatic Sea	Reared in Tyrrhenian Sea	Wild
Harvest date	19 July 2018	Non-breeding season	
	9 August 2018	Non-breeding season	
	13 September 2018	Non-breeding season	
	18 October 2018	Early breeding season (gonad maturation in the beginning of October) [29]	
Geographical origin	Adriatic Sea	Italian Tyrrhenian Sea	Levant Sea
Rearing system	Extruded fish feed fed on schedule		Natural food from the wild
Habitat	Open sea floating cage		Wild at sea
Number of samples	6 different fish fillets for first harvest date and 10 for other dates		
Replicates	3 replicates		

## Data Availability

The raw data supporting the conclusions of this article will be made available by the authors on request.

## Supplementary Materials

The following supporting information can be downloaded at: https://www.mdpi.com/article/10.3390/molecules30020402/s1, Figure S1: 227 mass peaks (mean ± SD) plotted for three fish types under two conditions (cooked and raw); Figure S2: A loading plot of principal component analysis (PCA) on the measured VOC concentration for raw and cooked wild sea bream that were reared in either the Adriatic, Tyrrhenian, or Levant Sea.; Figure S3: The average mass spectra of a fish sample of different origin both raw and cooked sampled in September. Table S1: VOC associated with fish samples and significantly different from blank samples.
