# Supplementary material for: PTR-ToF-MS VOC Profiling of Raw and Cooked Gilthead Sea Bream Fillet (Sparus aurata): Effect of Rearing System, Season, and Geographical Origin
Source: Molecules. 2025 Jan 18;30(2):402. doi: 10.3390/molecules30020402 (PMC11767258; doi:10.3390/molecules30020402)
Supplement: Supplementary file 1 [file molecules-30-00402-s001.zip › Figure_S1.pdf]

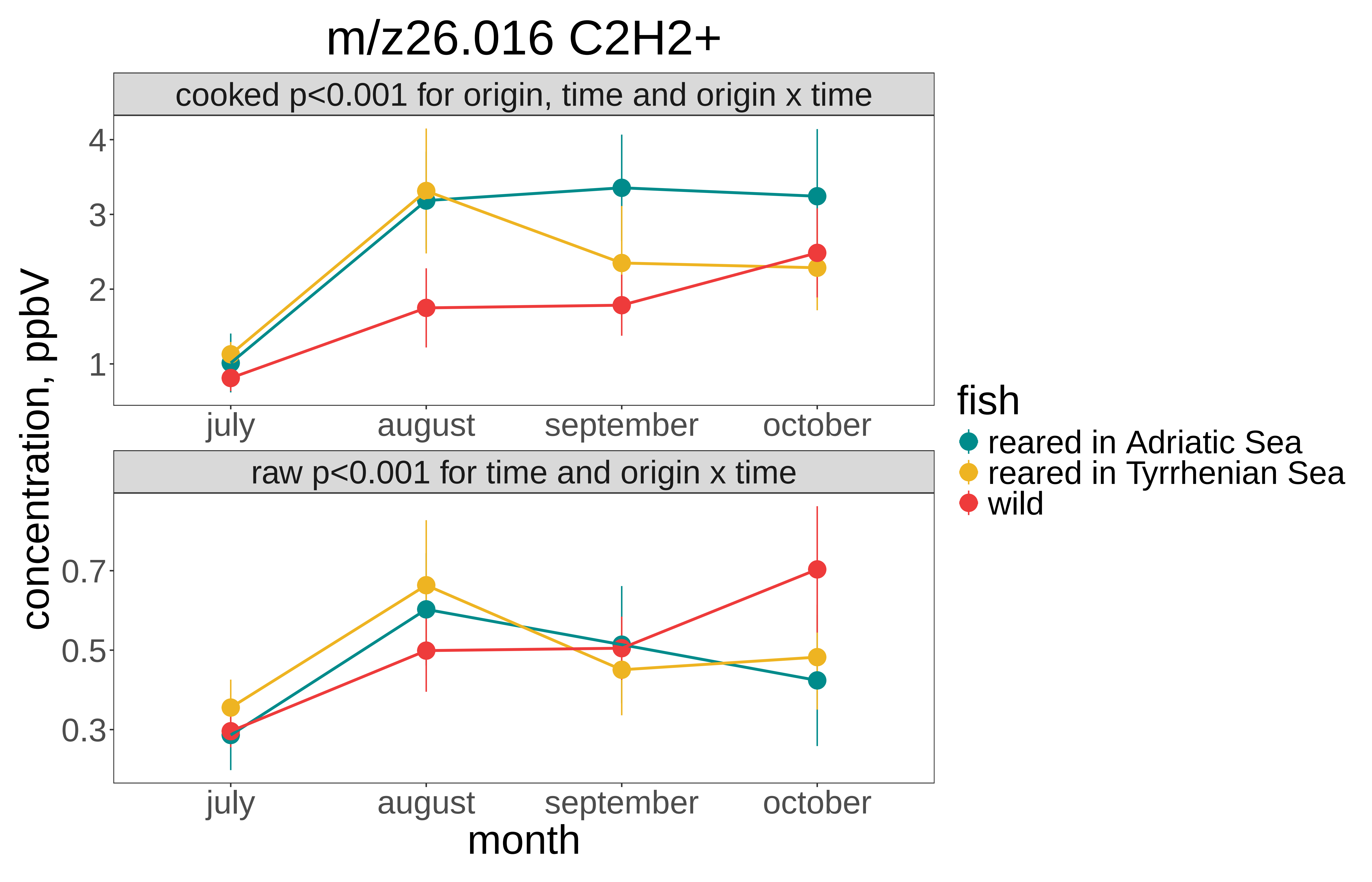

m/z26.016 C2H2+
cooked p<0.001 for origin, time and origin x time
raw p<0.001 for time and origin x time
concentration, ppbV
4
3
2
1
0.7
0.5
0.3
july
august
september
october
month
fish
reared in Adriatic Sea
reared in Tyrrhenian Sea
wild

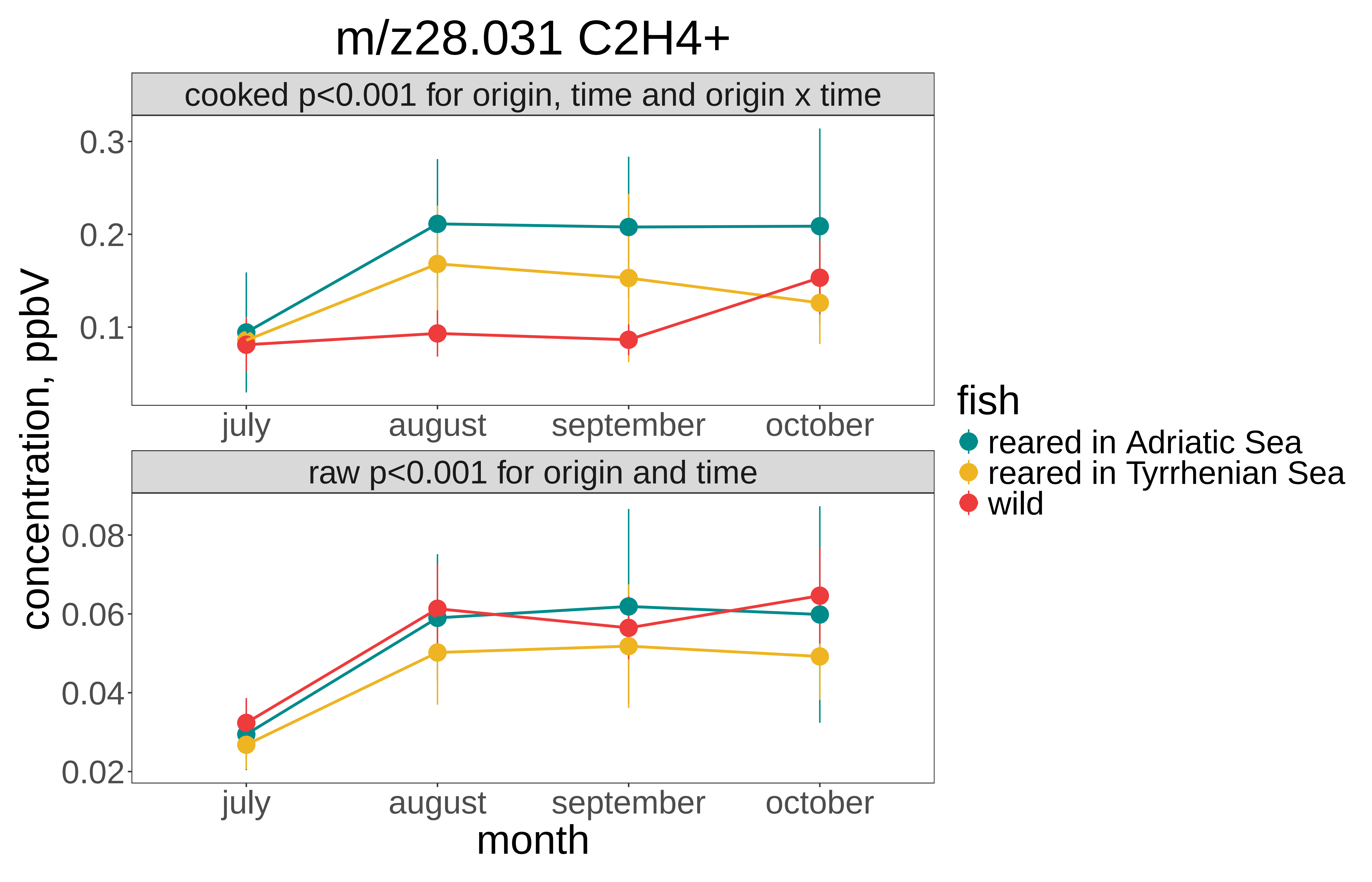

m/z28.031 C2H4+
cooked p<0.001 for origin, time and origin x time
0.3
0.2
0.1
july
august
september
october
raw p<0.001 for origin and time
0.08
0.06
0.04
0.02
july
august
september
october
concentration, ppbV
month
fish
reared in Adriatic Sea
reared in Tyrrhenian Sea
wild

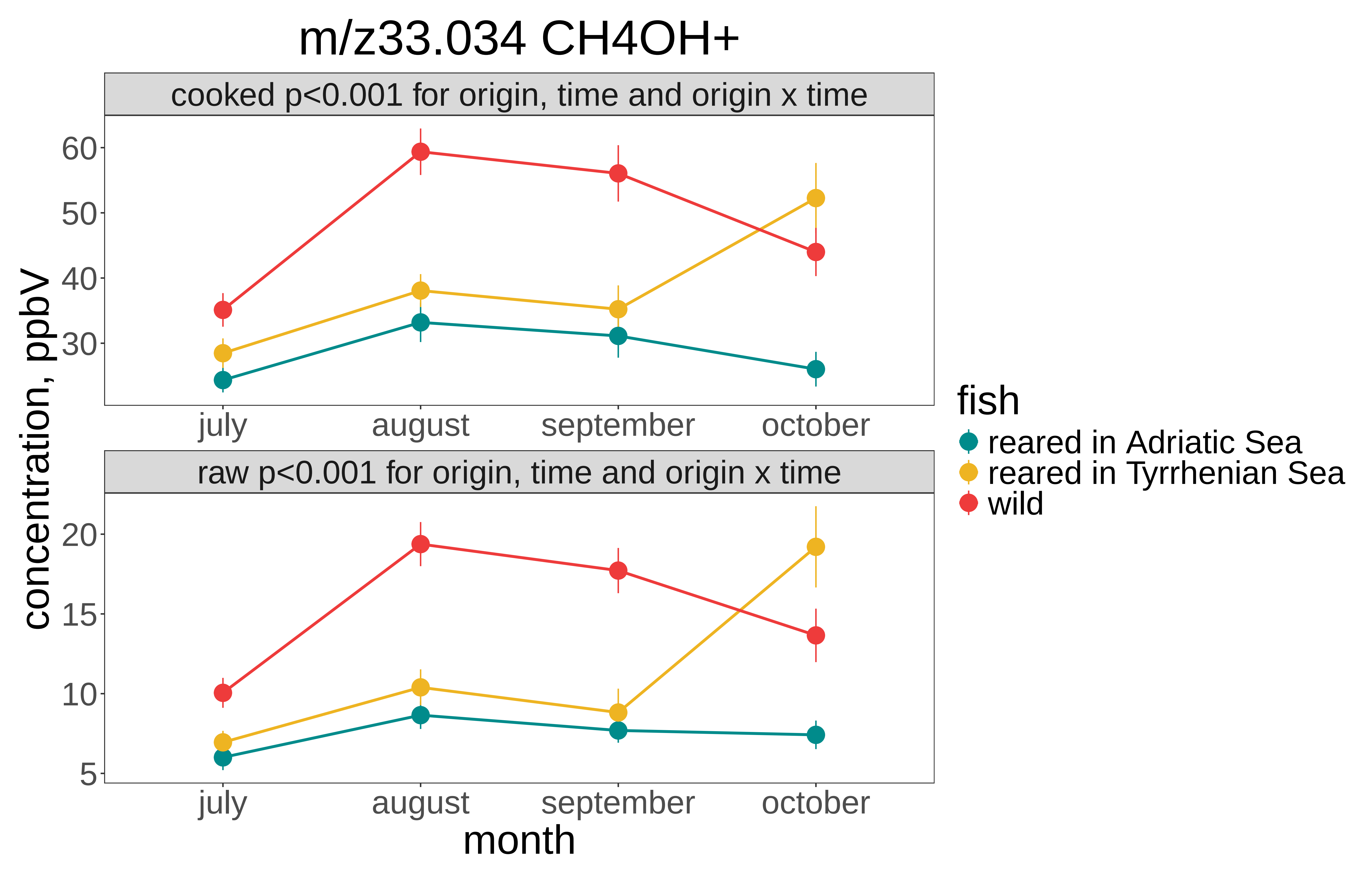

m/z33.034 CH4OH+
cooked p<0.001 for origin, time and origin x time
raw p<0.001 for origin, time and origin x time
concentration, ppbV
60
50
40
30
20
15
10
5
july
august
september
october
month
fish
reared in Adriatic Sea
reared in Tyrrhenian Sea
wild

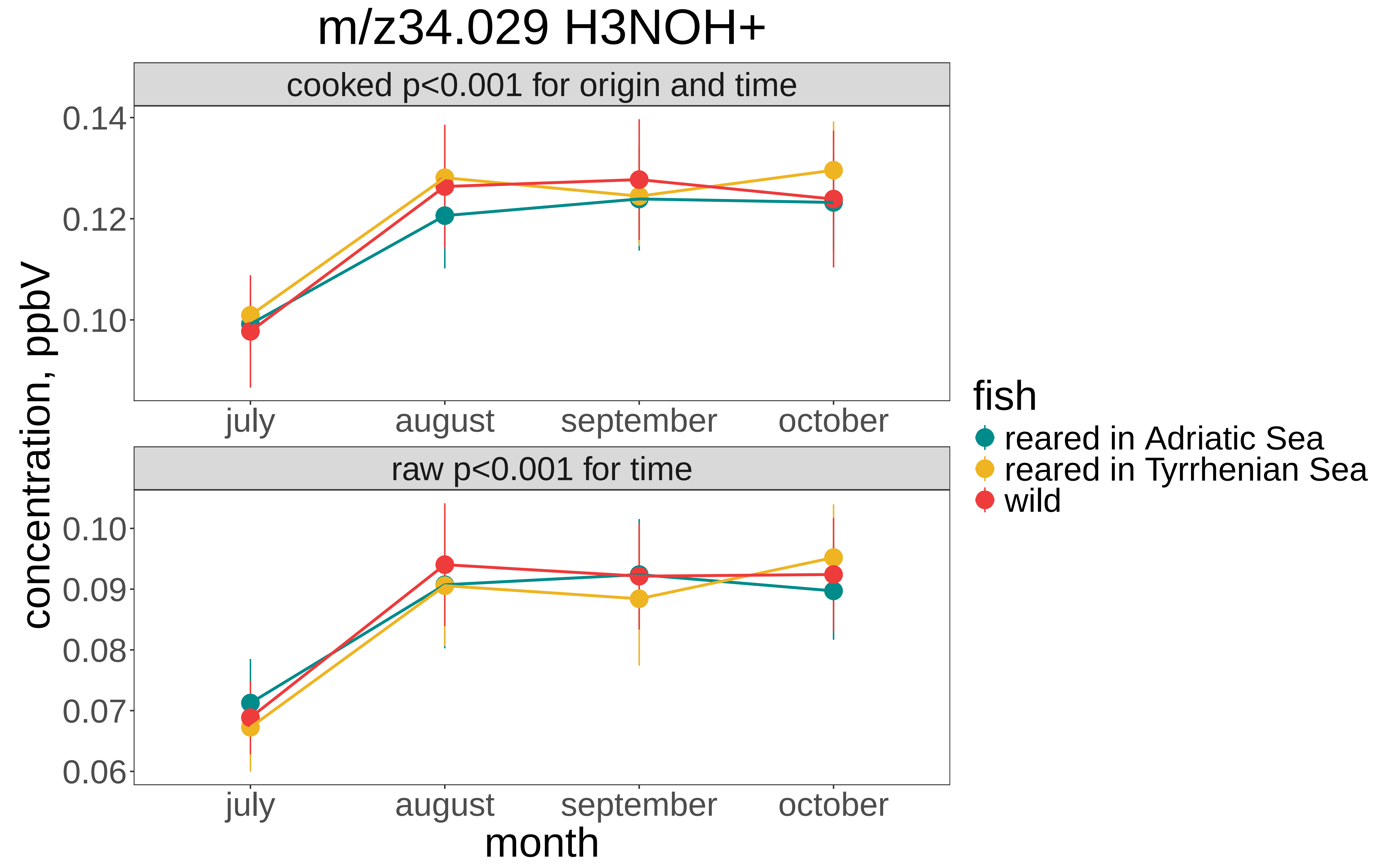

m/z34.029 H3NOH+
cooked p<0.001 for origin and time
raw p<0.001 for time
concentration, ppbV
0.14
0.12
0.10
0.10
0.09
0.08
0.07
0.06
july
august
september
october
month
fish
reared in Adriatic Sea
reared in Tyrrhenian Sea
wild

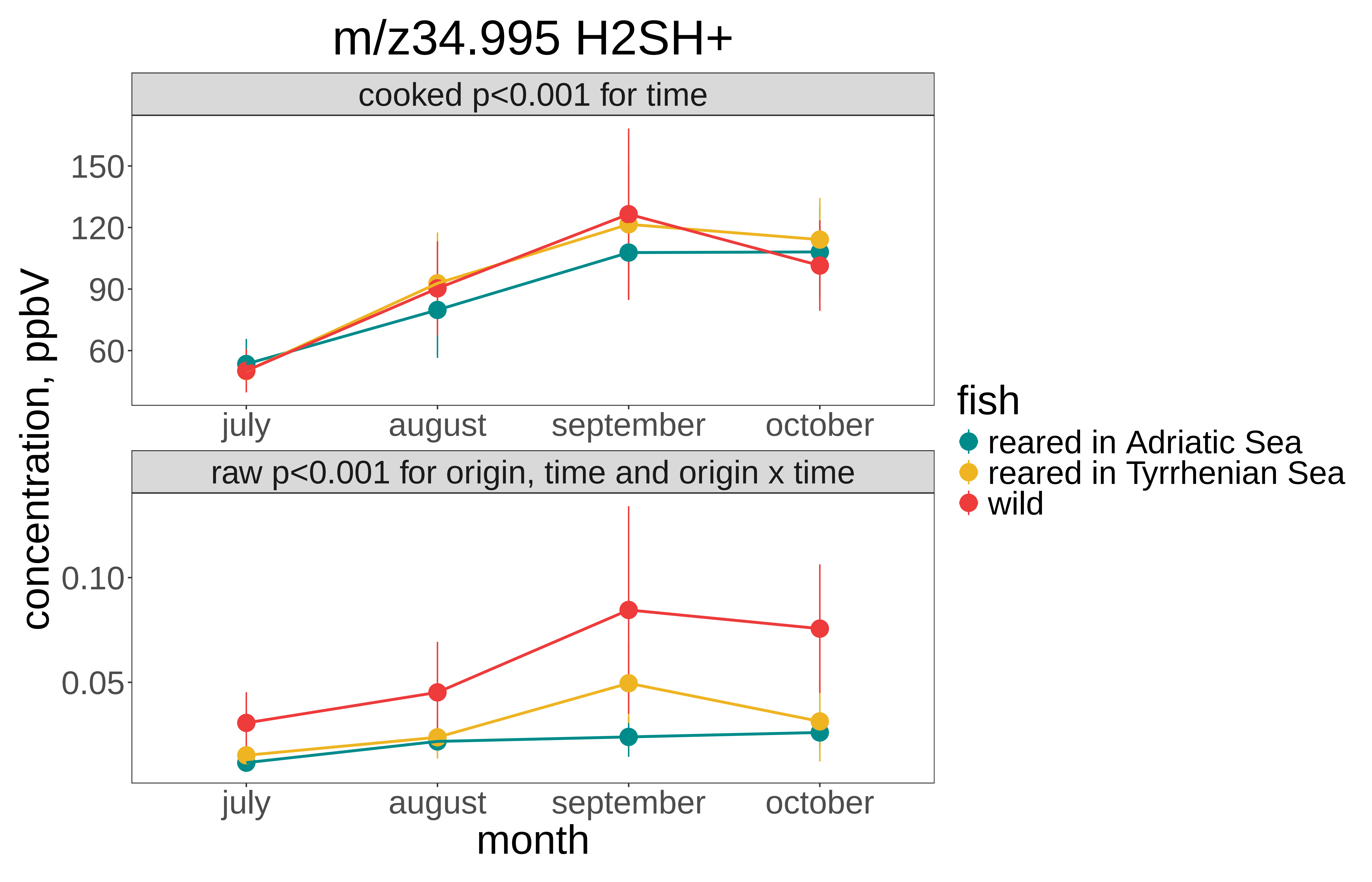

m/z34.995 H2SH+
cooked p<0.001 for time
150
120
90
60
july
august
september
october
raw p<0.001 for origin, time and origin x time
0.10
0.05
july
august
september
october
month
concentration, ppbV
fish
reared in Adriatic Sea
reared in Tyrrhenian Sea
wild

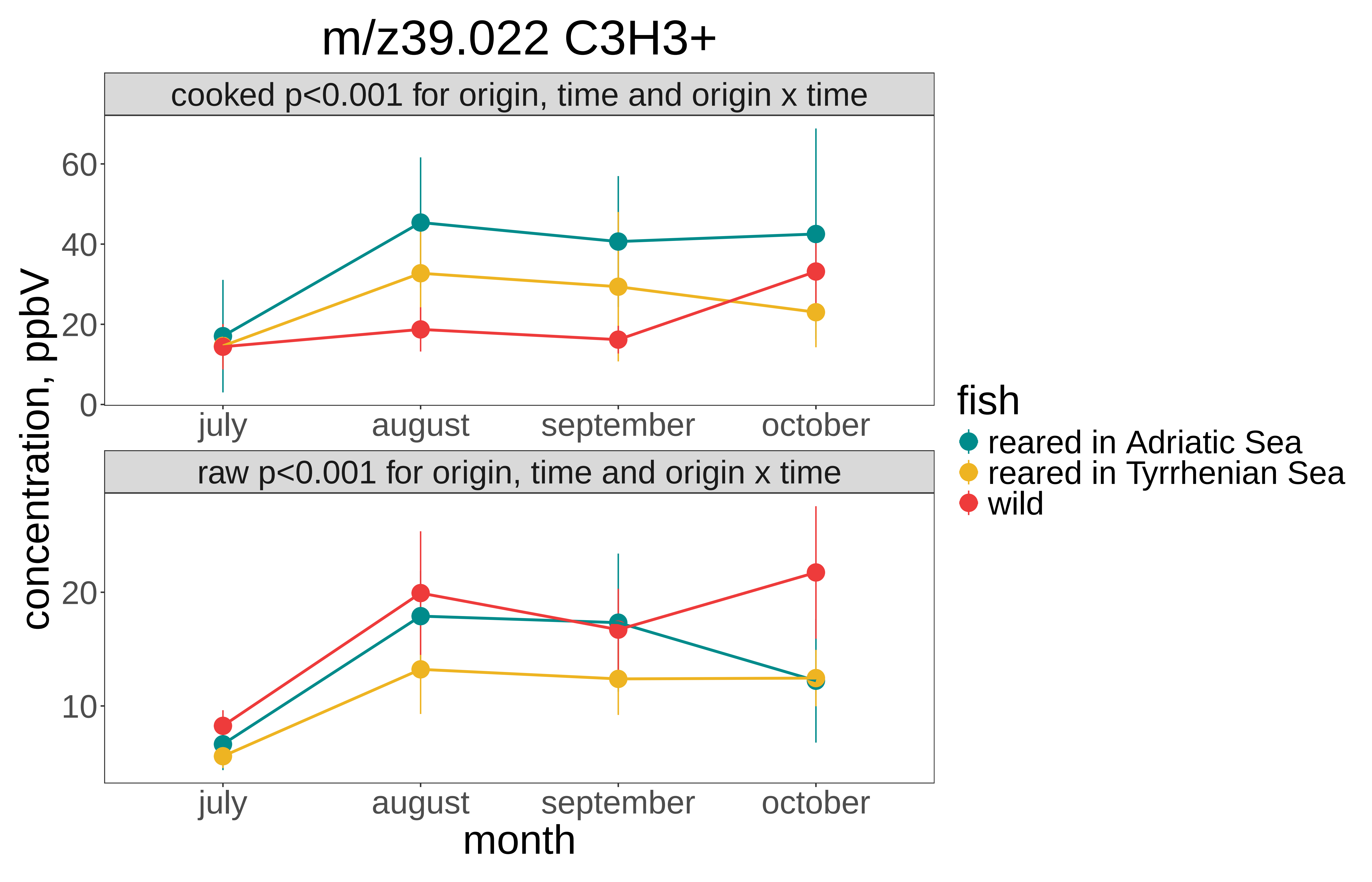
m/z39.022 C3H3+
cooked p<0.001 for origin, time and origin x time
raw p<0.001 for origin, time and origin x time
concentration, ppbV
0
20
40
60
10
20
july
august
september
october
month
fish
reared in Adriatic Sea
reared in Tyrrhenian Sea
wild

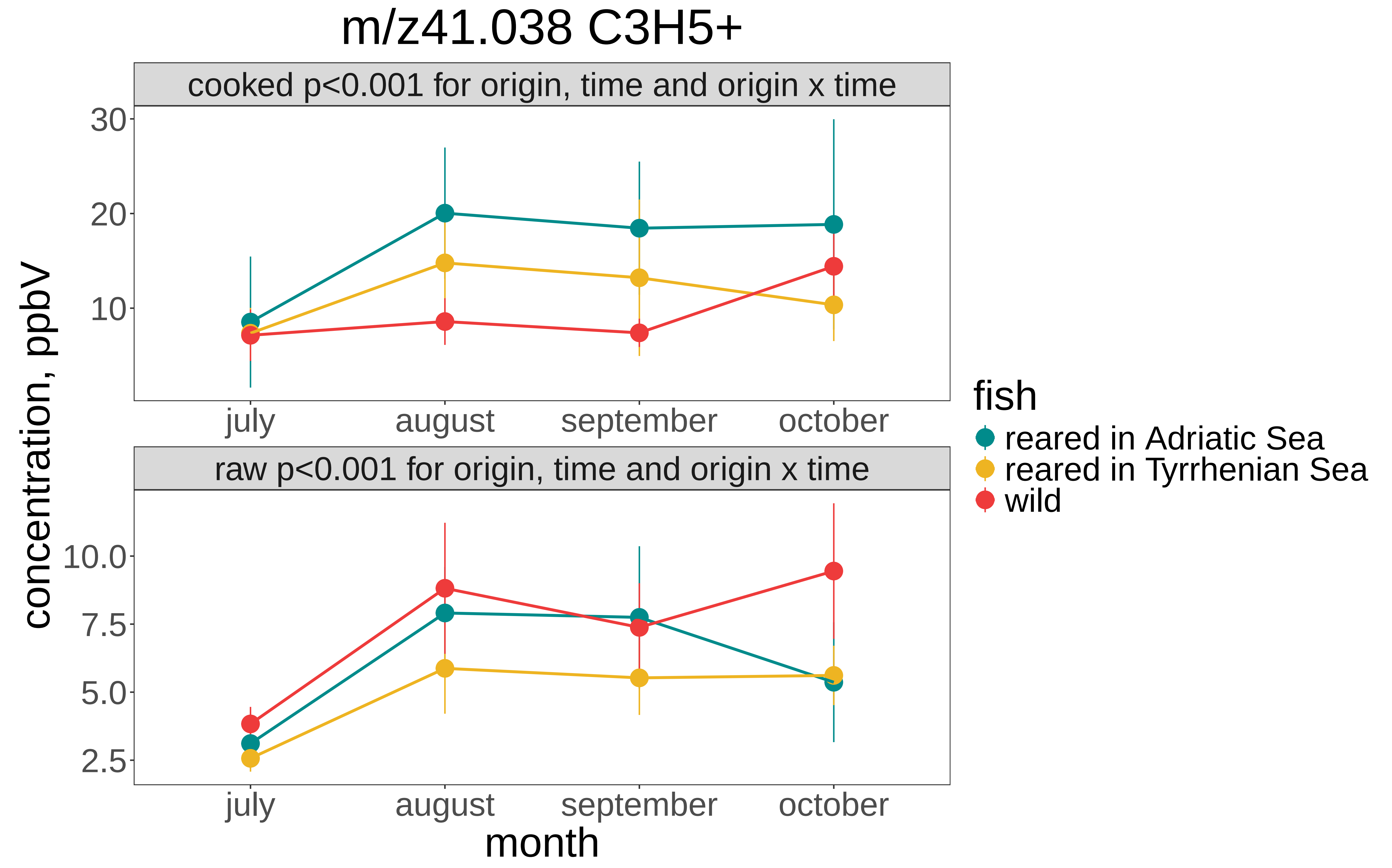

m/z41.038 C3H5+
cooked p<0.001 for origin, time and origin x time
raw p<0.001 for origin, time and origin x time
concentration, ppbV
30
20
10
10.0
7.5
5.0
2.5
july
august
september
october
month
fish
reared in Adriatic Sea
reared in Tyrrhenian Sea
wild

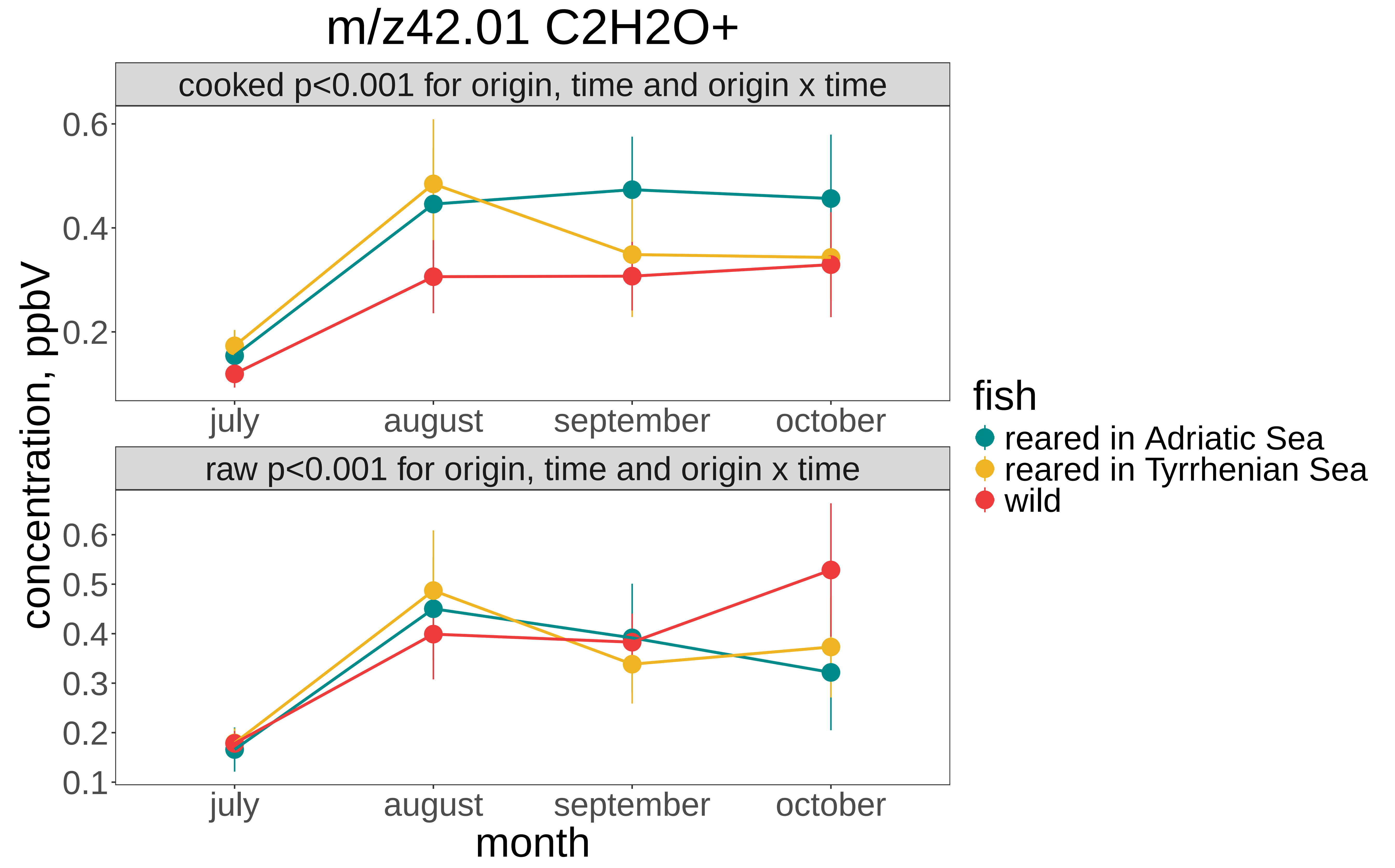

m/z42.01 C2H2O+
cooked p<0.001 for origin, time and origin x time
raw p<0.001 for origin, time and origin x time
concentration, ppbV
0.6
0.4
0.2
0.6
0.5
0.4
0.3
0.2
0.1
july
august
september
october
month
fish
reared in Adriatic Sea
reared in Tyrrhenian Sea
wild

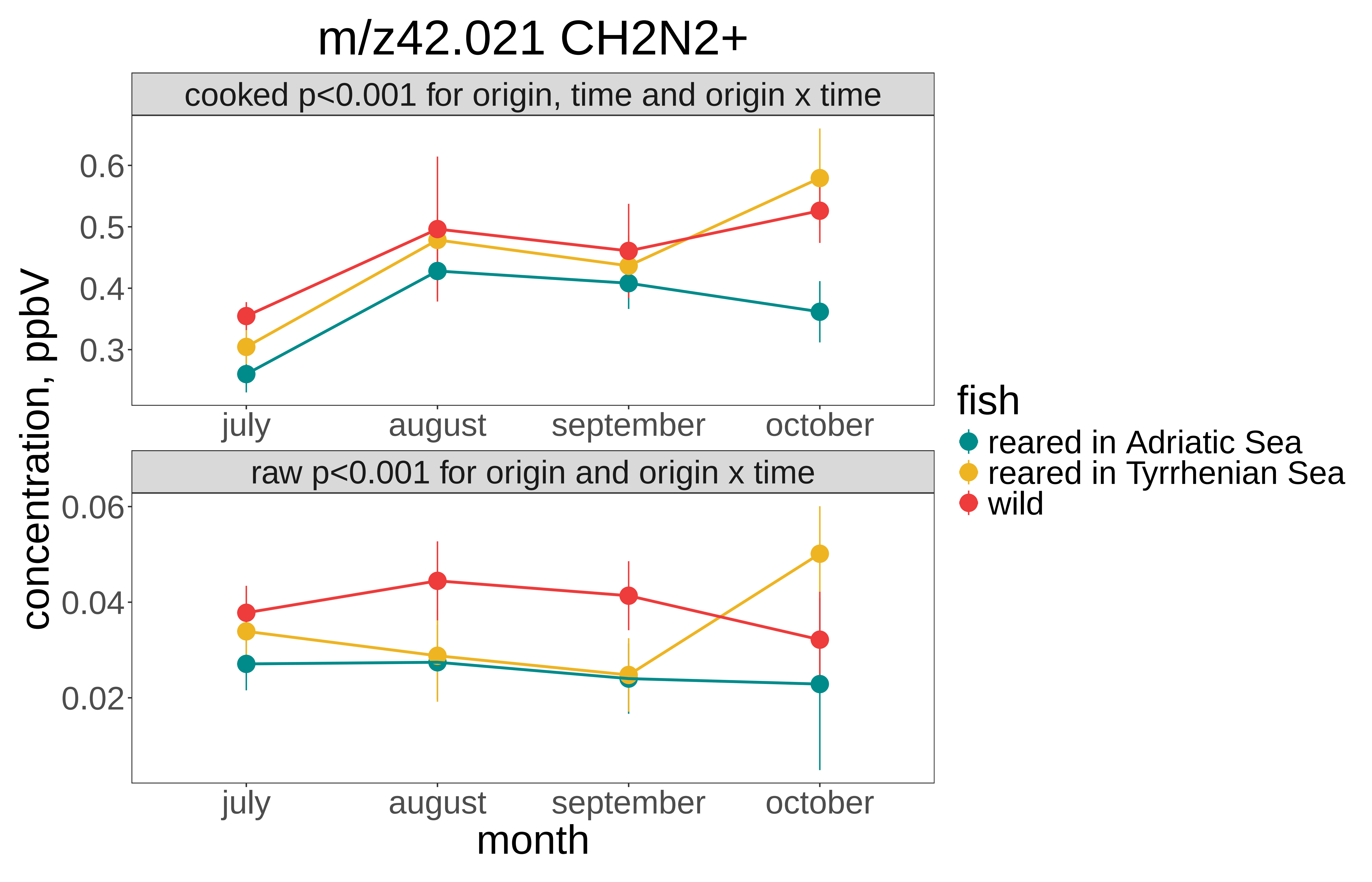

m/z42.021 CH2N2+
cooked p<0.001 for origin, time and origin x time
raw p<0.001 for origin and origin x time
concentration, ppbV
0.6
0.5
0.4
0.3
0.06
0.04
0.02
july
august
september
october
month
fish
reared in Adriatic Sea
reared in Tyrrhenian Sea
wild

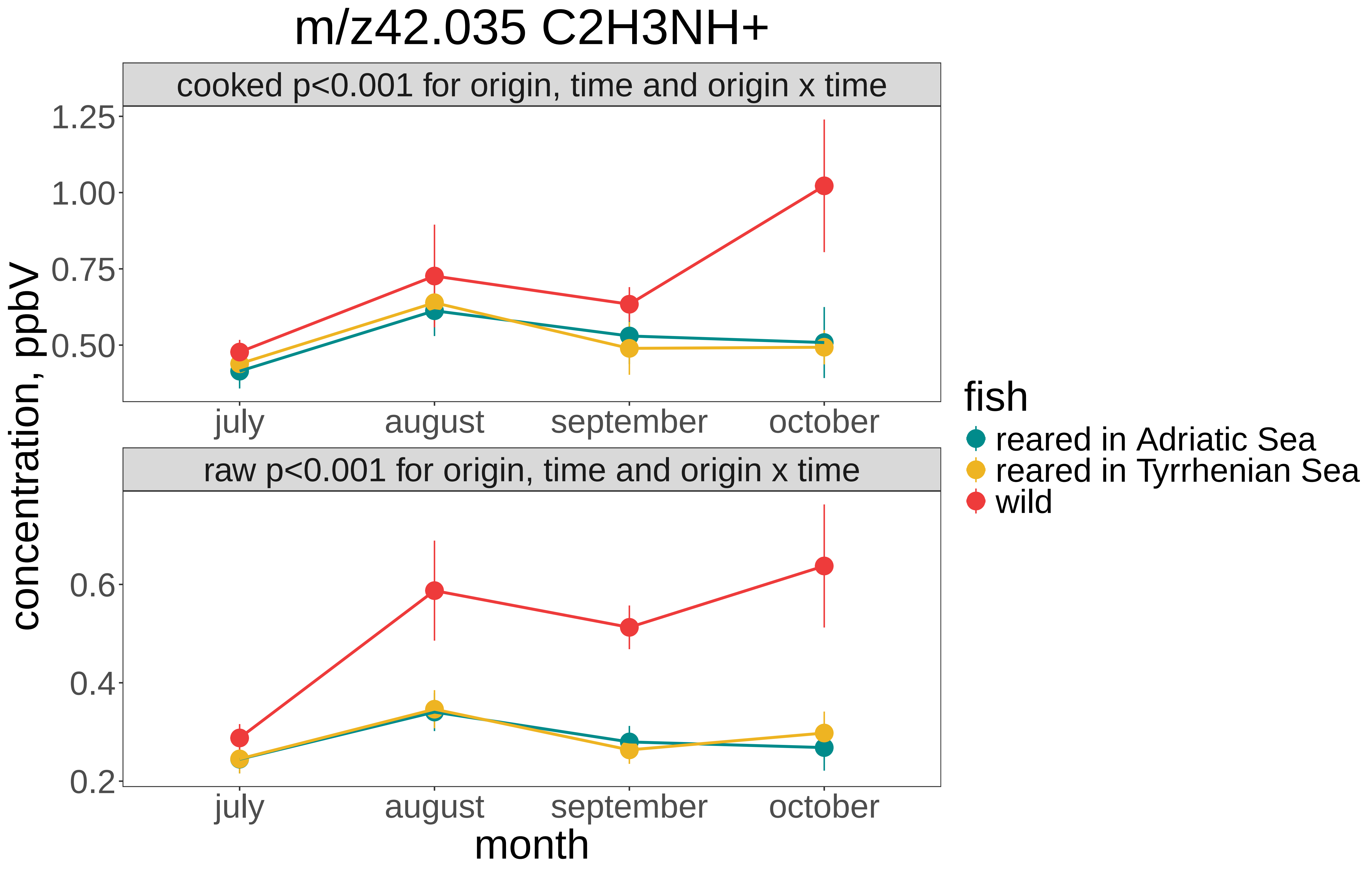

m/z42.035 C2H3NH+
cooked p<0.001 for origin, time and origin x time
raw p<0.001 for origin, time and origin x time
concentration, ppbV
1.25
1.00
0.75
0.50
0.6
0.4
0.2
july
august
september
october
month
fish
reared in Adriatic Sea
reared in Tyrrhenian Sea
wild

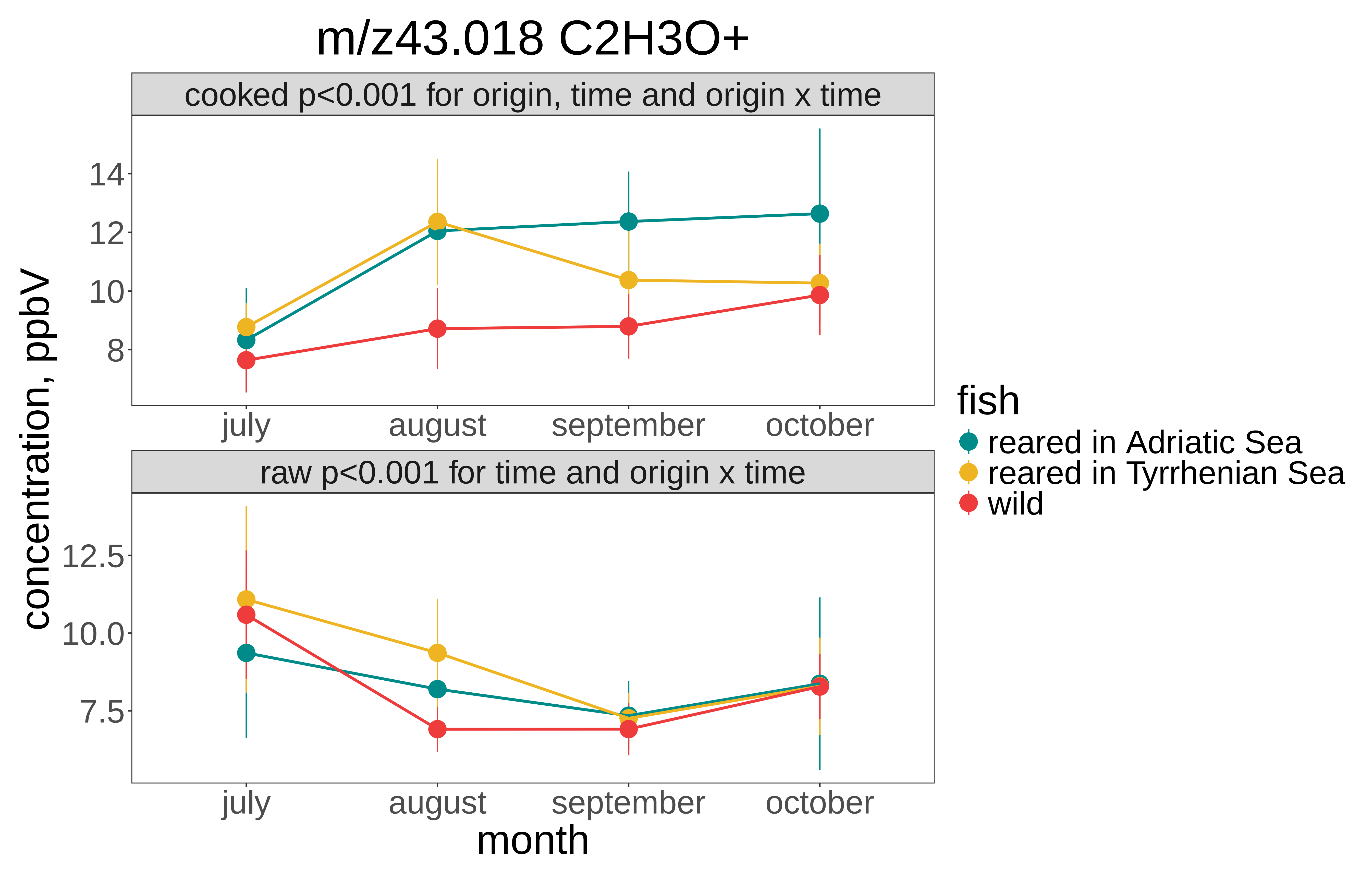

m/z43.018 C2H3O+
cooked p<0.001 for origin, time and origin x time
raw p<0.001 for time and origin x time
concentration, ppbV
month
july
august
september
october
14
12
10
8
12.5
10.0
7.5
fish
reared in Adriatic Sea
reared in Tyrrhenian Sea
wild

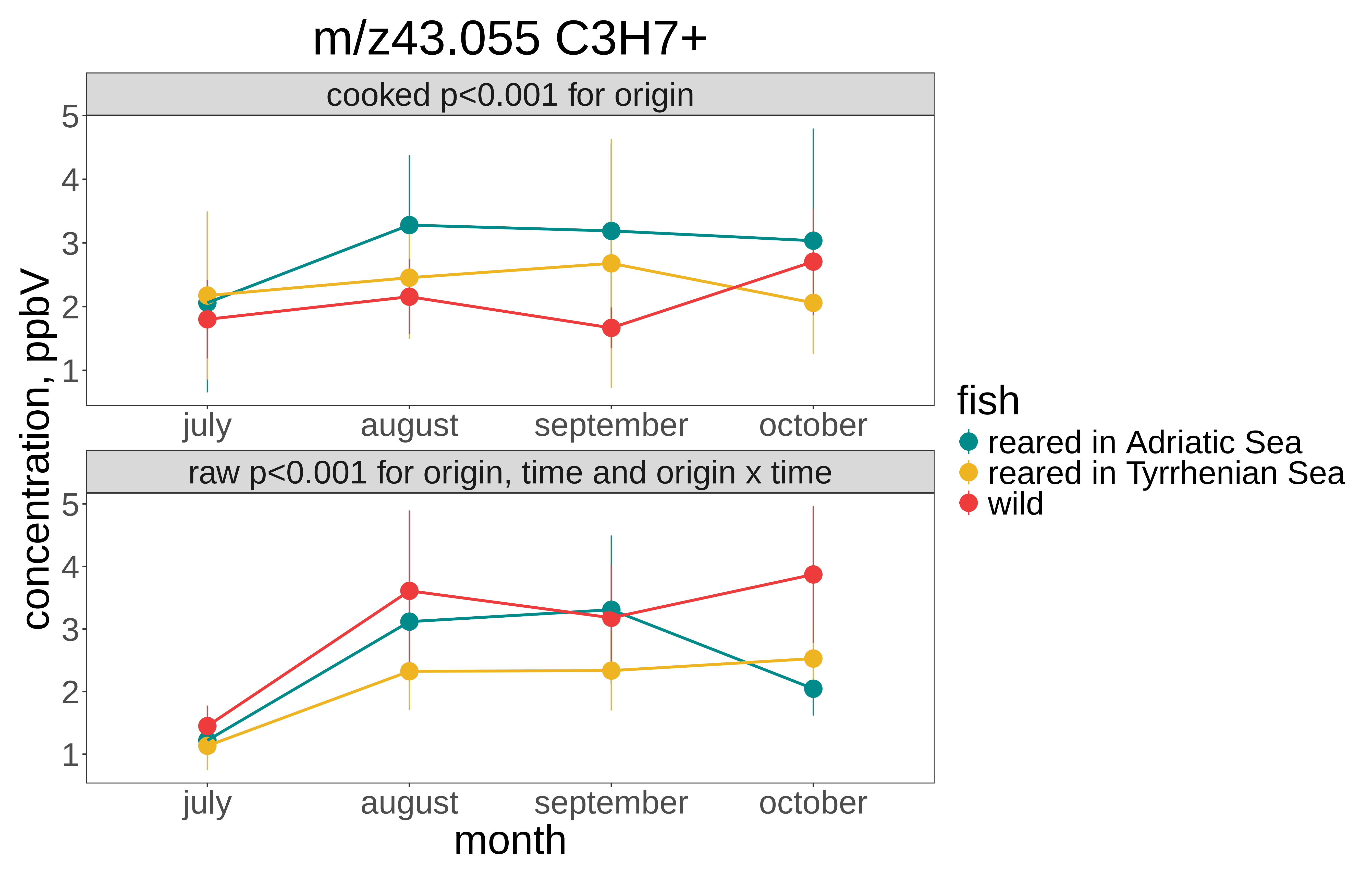

m/z43.055 C3H7+
cooked p<0.001 for origin
raw p<0.001 for origin, time and origin x time
concentration, ppbV
month
july
august
september
october
1
2
3
4
5
fish
reared in Adriatic Sea
reared in Tyrrhenian Sea
wild

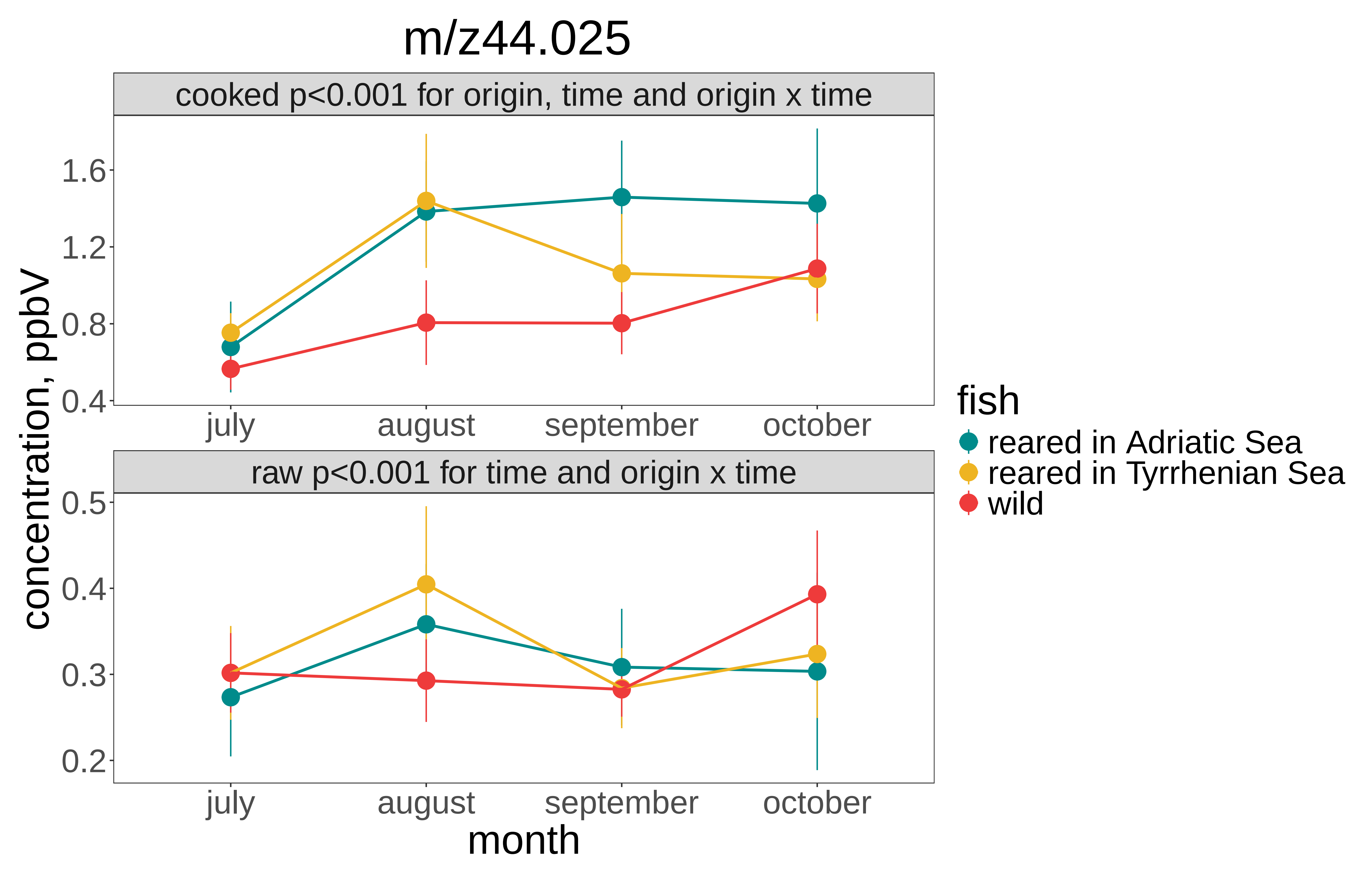

m/z44.025
cooked p<0.001 for origin, time and origin x time
raw p<0.001 for time and origin x time
concentration, ppbV
1.6
1.2
0.8
0.4
0.5
0.4
0.3
0.2
july
august
september
october
month
fish
reared in Adriatic Sea
reared in Tyrrhenian Sea
wild

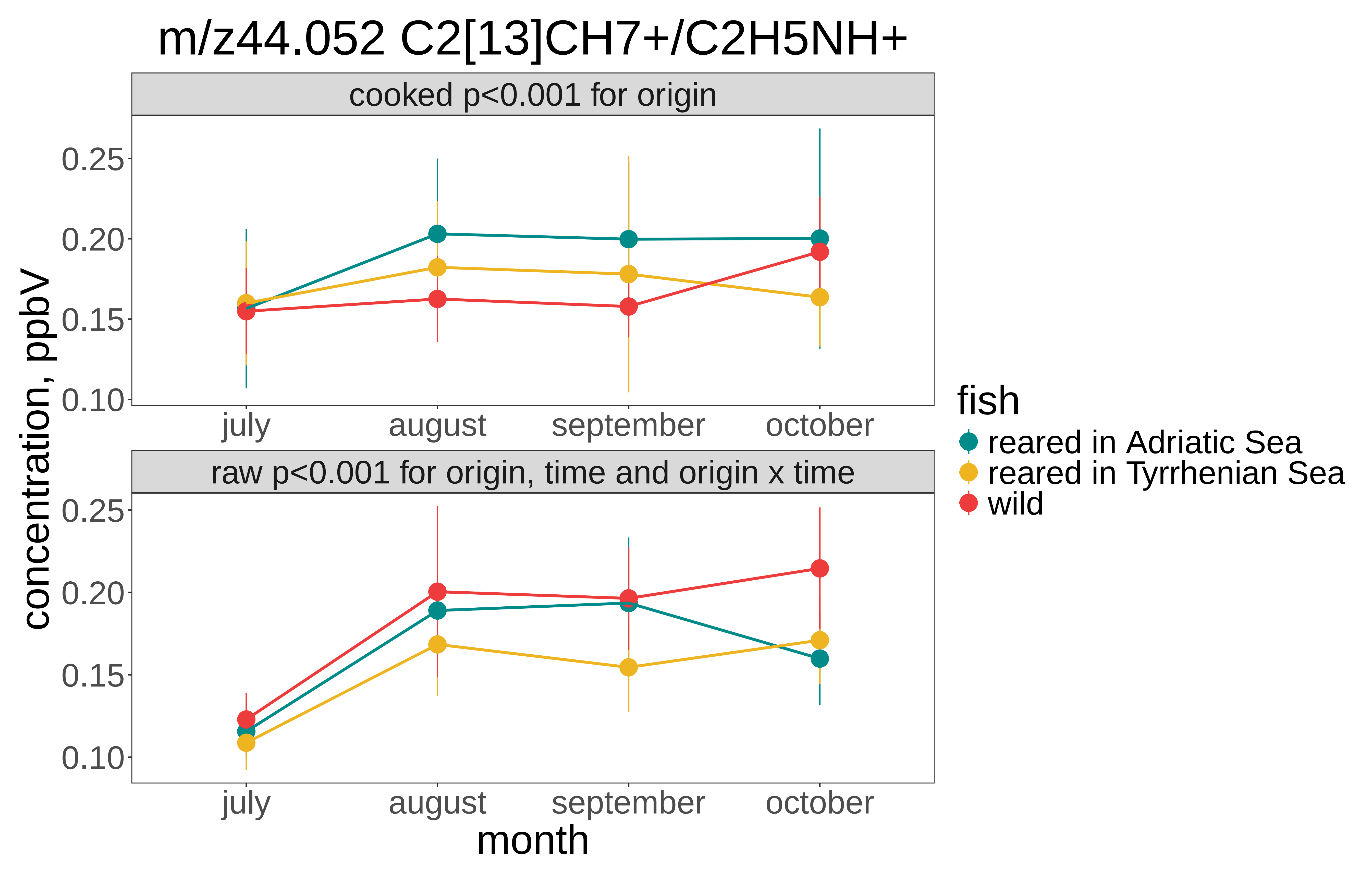
m/z44.052 C2[13]CH7+/C2H5NH+
cooked p<0.001 for origin
raw p<0.001 for origin, time and origin x time
concentration, ppbV
0.10
0.15
0.20
0.25
july
august
september
october
month
fish
reared in Adriatic Sea
reared in Tyrrhenian Sea
wild

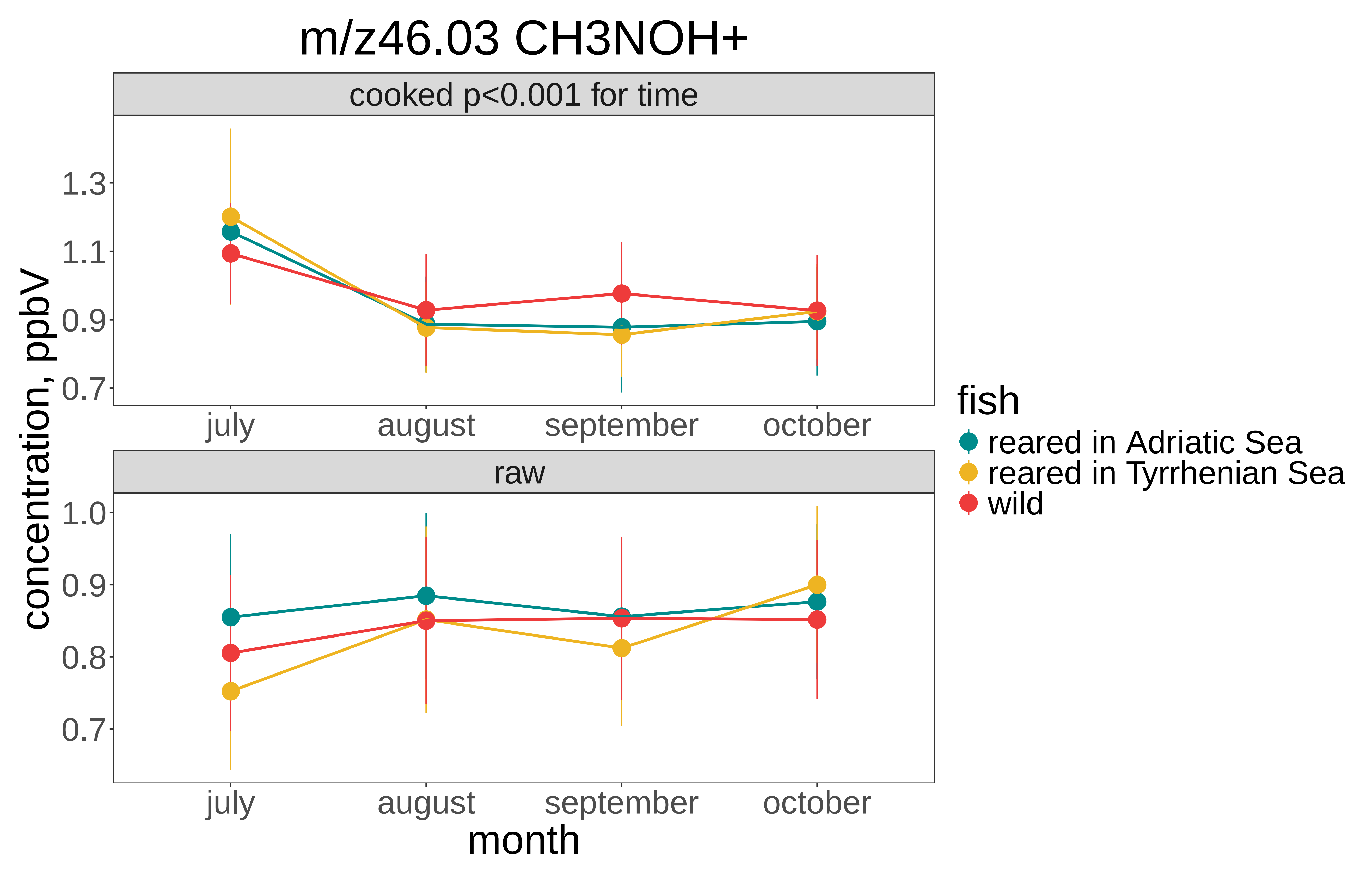
m/z46.03 CH3NOH+
cooked p<0.001 for time
raw
concentration, ppbV
1.3
1.1
0.9
0.7
1.0
0.9
0.8
0.7
july
august
september
october
month
fish
reared in Adriatic Sea
reared in Tyrrhenian Sea
wild

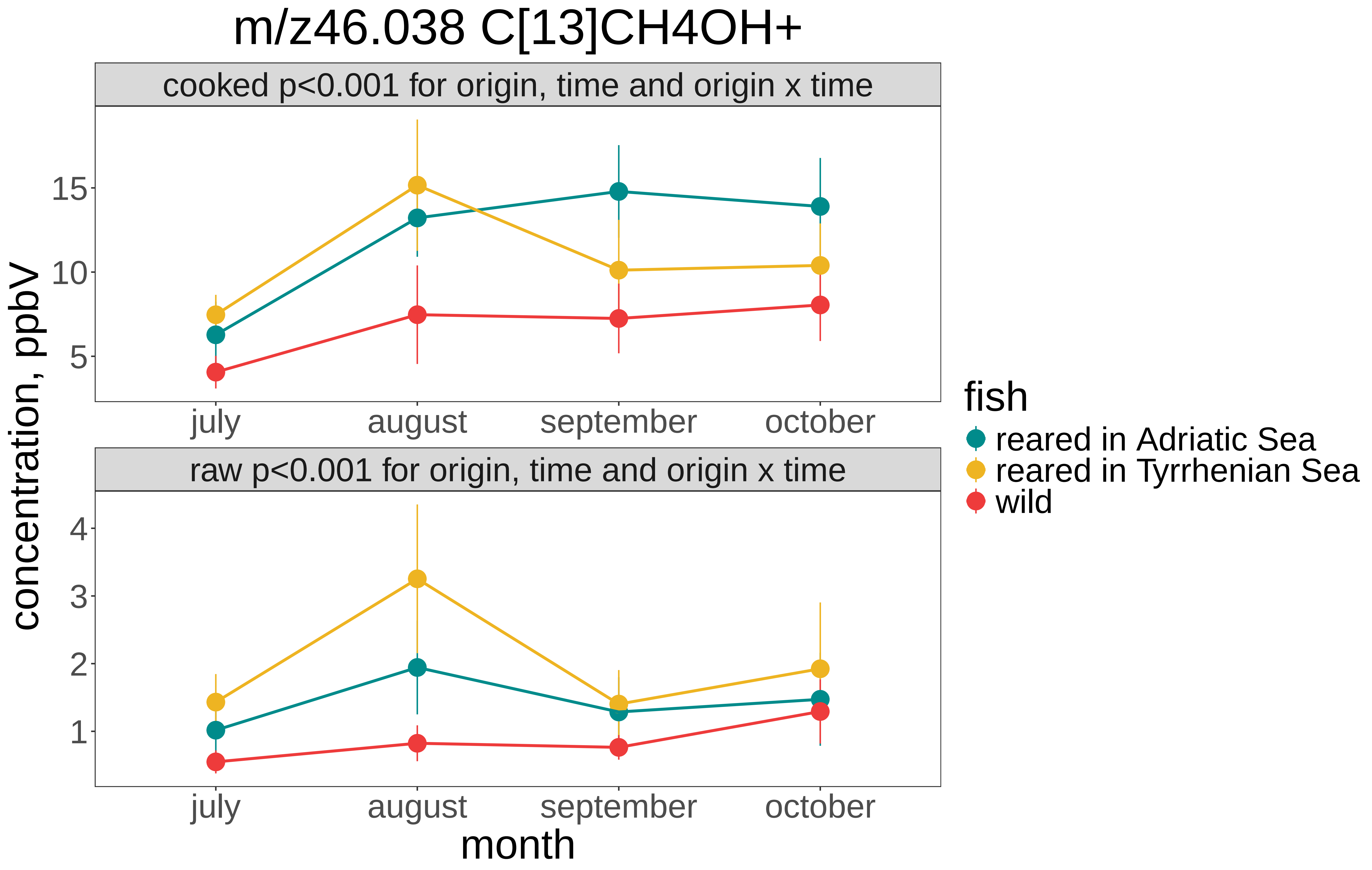
m/z46.038 C[13]CH4OH+
cooked p<0.001 for origin, time and origin x time
raw p<0.001 for origin, time and origin x time
concentration, ppbV
15
10
5
4
3
2
1
july
august
september
october
month
fish
reared in Adriatic Sea
reared in Tyrrhenian Sea
wild

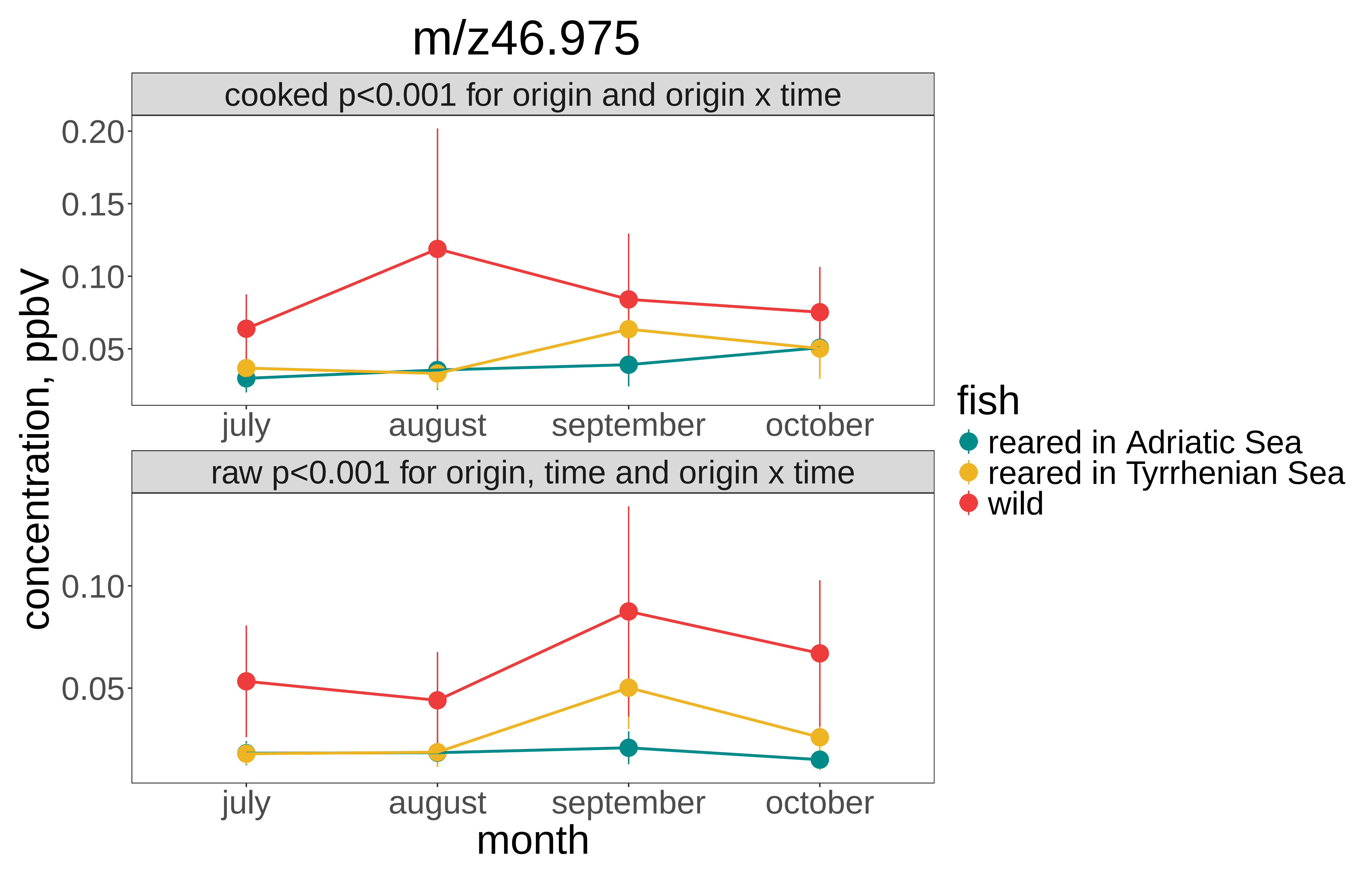

m/z46.975
cooked p<0.001 for origin and origin x time
raw p<0.001 for origin, time and origin x time
concentration, ppbV
0.20
0.15
0.10
0.05
july
august
september
october
month
fish
reared in Adriatic Sea
reared in Tyrrhenian Sea
wild

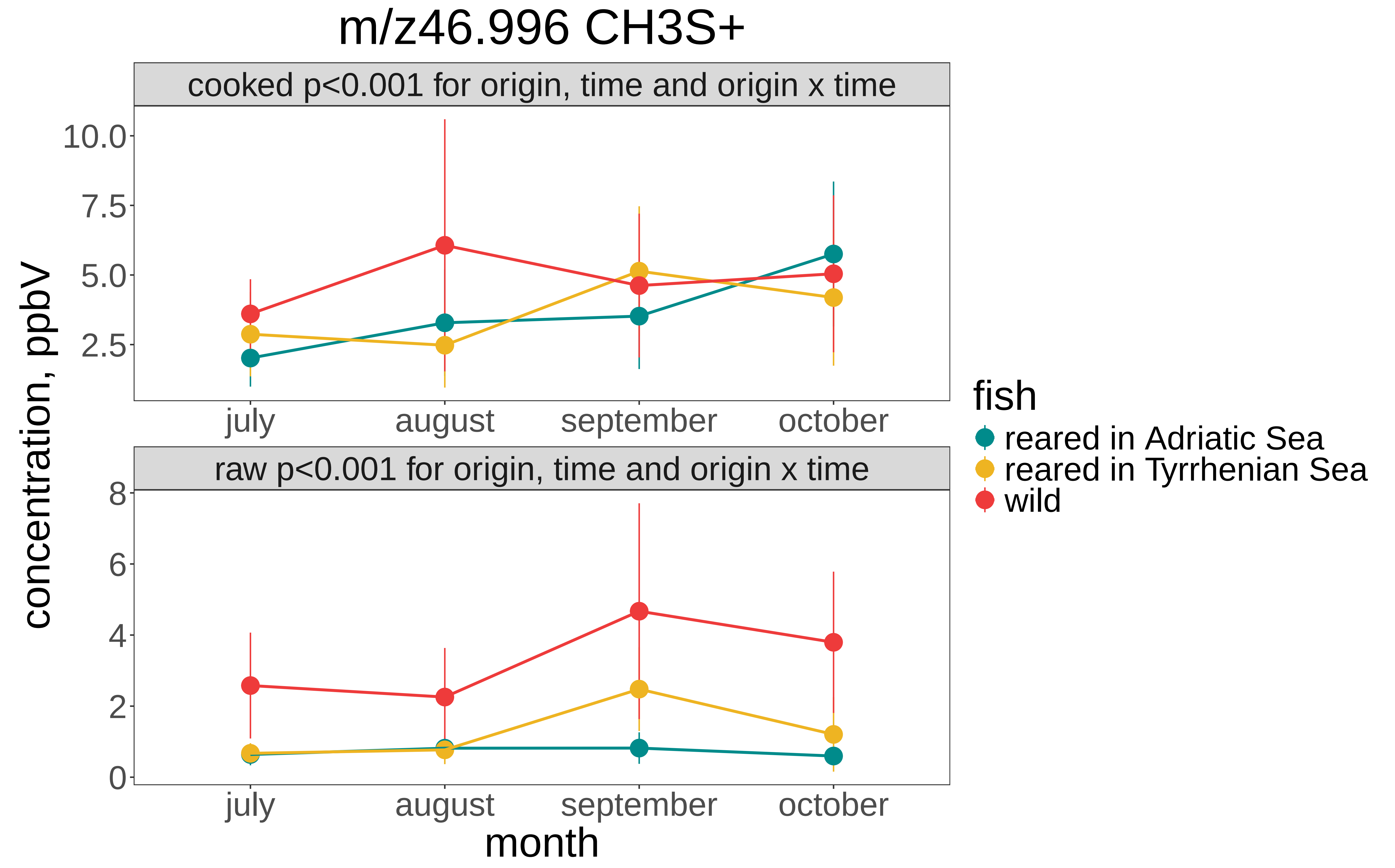

m/z46.996 CH3S+
cooked p<0.001 for origin, time and origin x time
raw p<0.001 for origin, time and origin x time
10.0
7.5
5.0
2.5
8
6
4
2
0
concentration, ppbV
july
august
september
october
month
fish
reared in Adriatic Sea
reared in Tyrrhenian Sea
wild

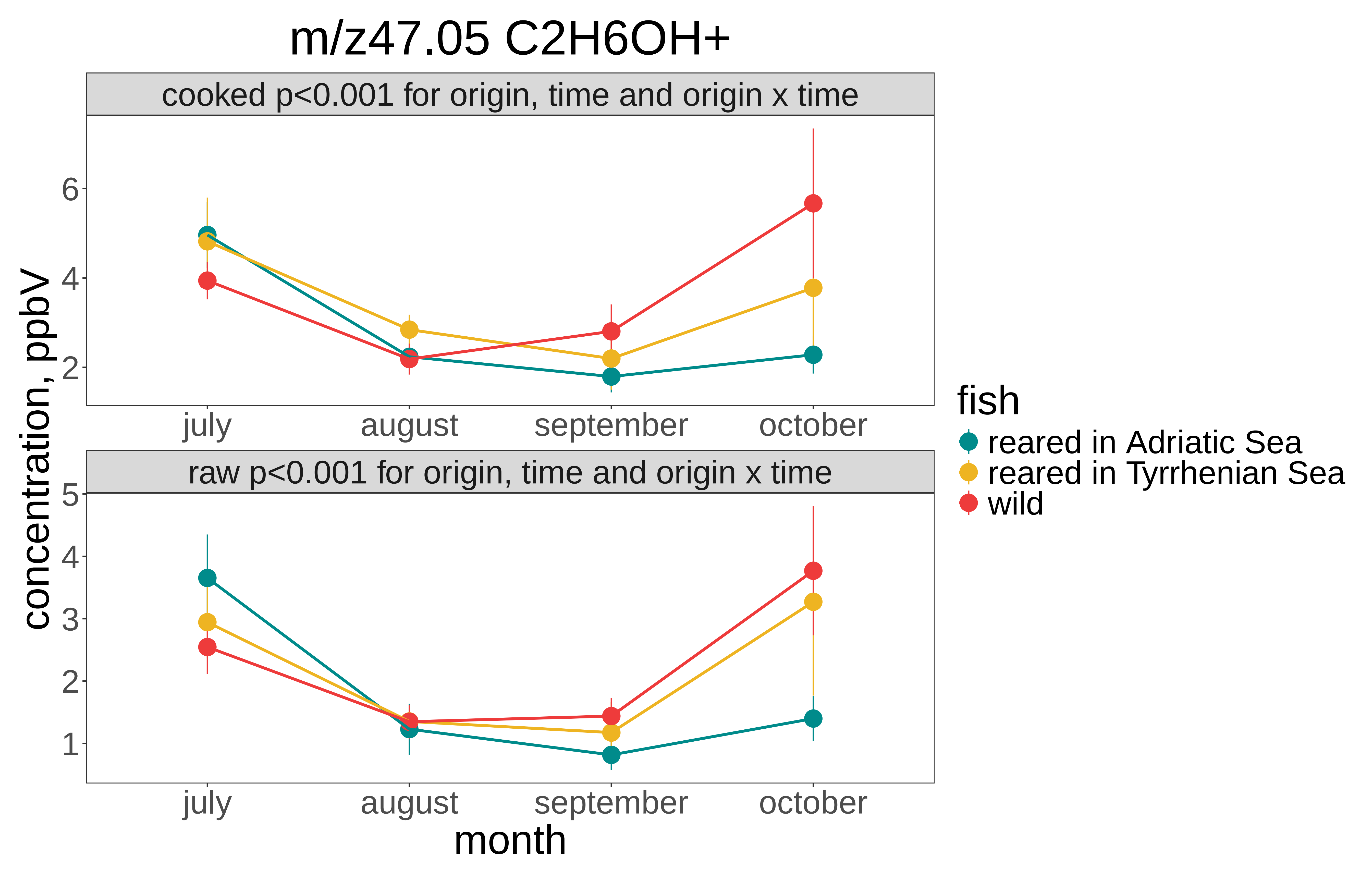
m/z47.05 C2H6OH+
cooked p<0.001 for origin, time and origin x time
raw p<0.001 for origin, time and origin x time
concentration, ppbV
6
4
2
5
4
3
2
1
july
august
september
october
month
fish
reared in Adriatic Sea
reared in Tyrrhenian Sea
wild

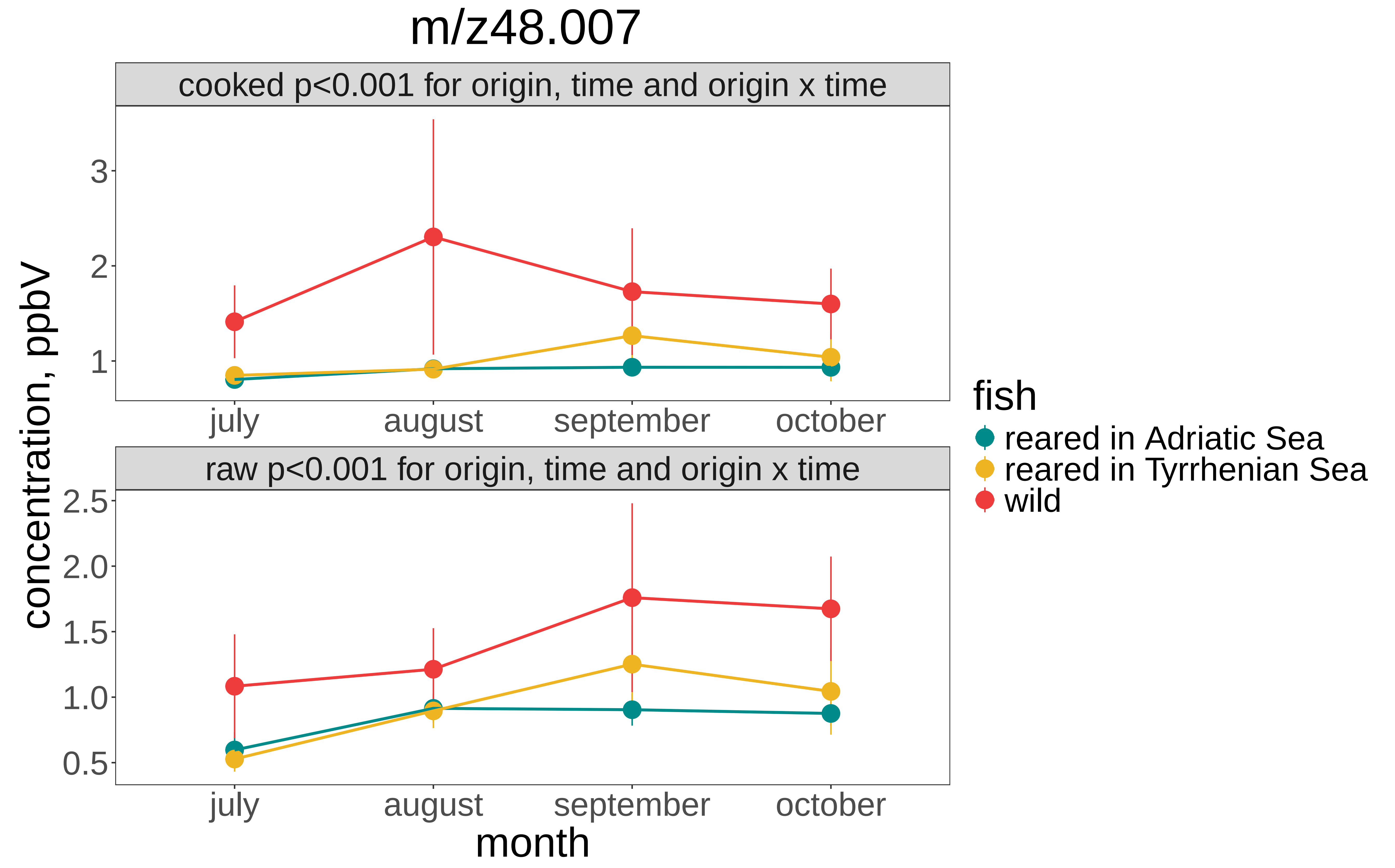

m/z48.007
cooked p<0.001 for origin, time and origin x time
raw p<0.001 for origin, time and origin x time
concentration, ppbV
3
2
1
2.5
2.0
1.5
1.0
0.5
july
august
september
october
month
fish
reared in Adriatic Sea
reared in Tyrrhenian Sea
wild

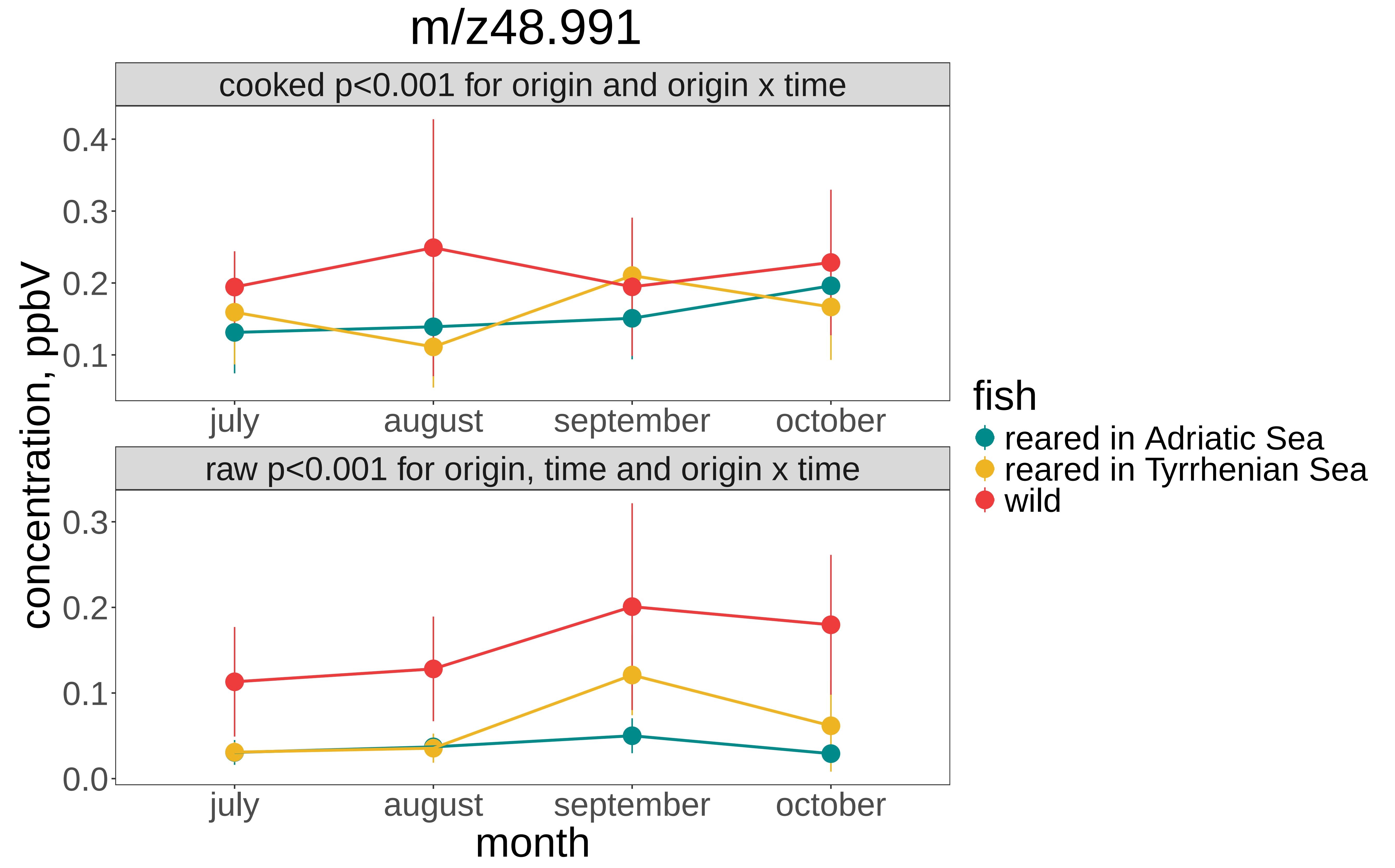

m/z48.991
cooked p<0.001 for origin and origin x time
raw p<0.001 for origin, time and origin x time
concentration, ppbV
0.4
0.3
0.2
0.1
0.0
july
august
september
october
month
fish
reared in Adriatic Sea
reared in Tyrrhenian Sea
wild

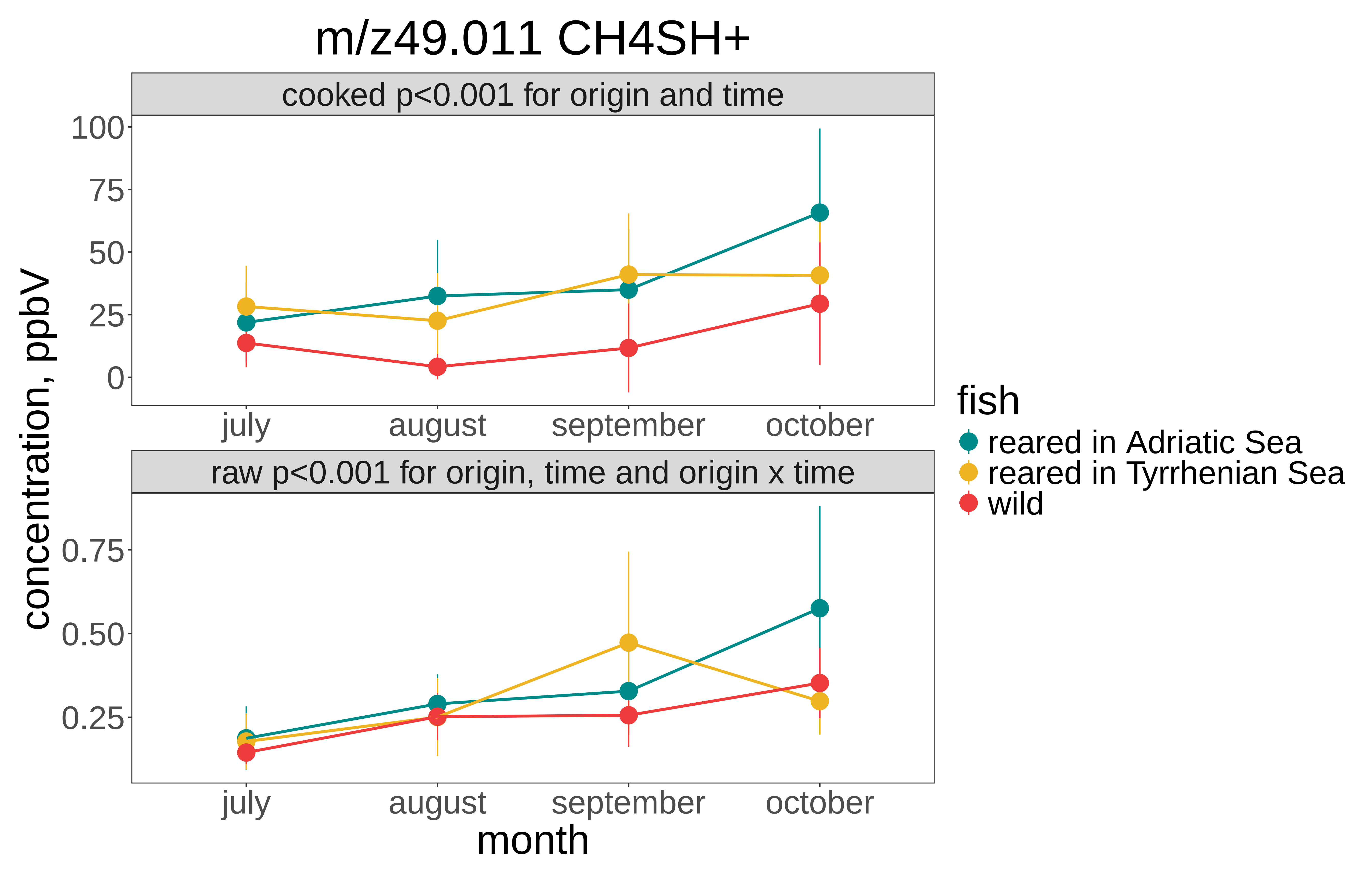
m/z49.011 CH4SH+
cooked p<0.001 for origin and time
raw p<0.001 for origin, time and origin x time
concentration, ppbV
100
75
50
25
0
0.75
0.50
0.25
july
august
september
october
month
fish
reared in Adriatic Sea
reared in Tyrrhenian Sea
wild

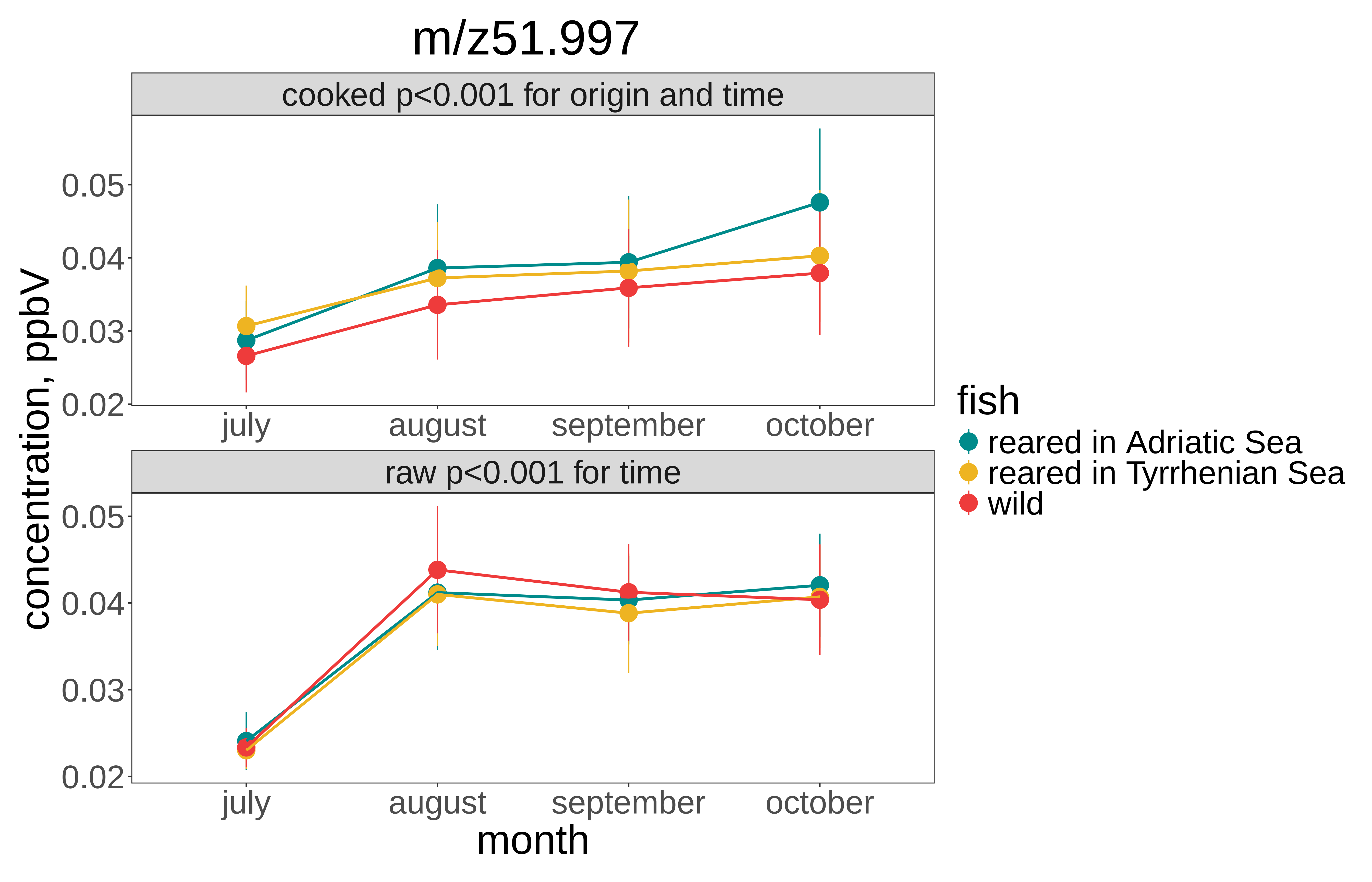

m/z51.997
cooked p<0.001 for origin and time
raw p<0.001 for time
0.05
0.04
0.03
0.02
july
august
september
october
concentration, ppbV
month
fish
reared in Adriatic Sea
reared in Tyrrhenian Sea
wild

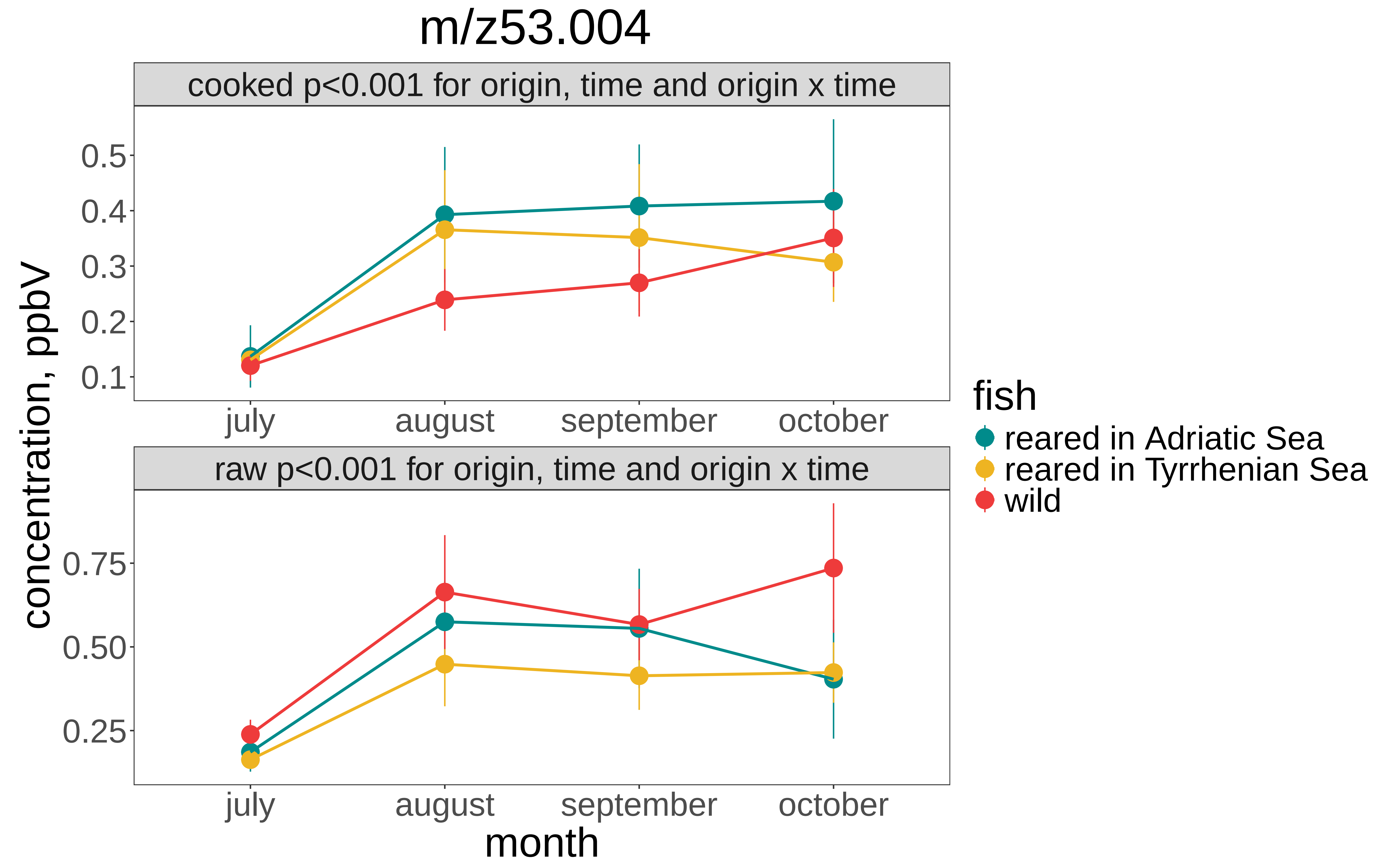
m/z53.004
cooked p<0.001 for origin, time and origin x time
raw p<0.001 for origin, time and origin x time
concentration, ppbV
0.5
0.4
0.3
0.2
0.1
0.75
0.50
0.25
july
august
september
october
month
fish
reared in Adriatic Sea
reared in Tyrrhenian Sea
wild

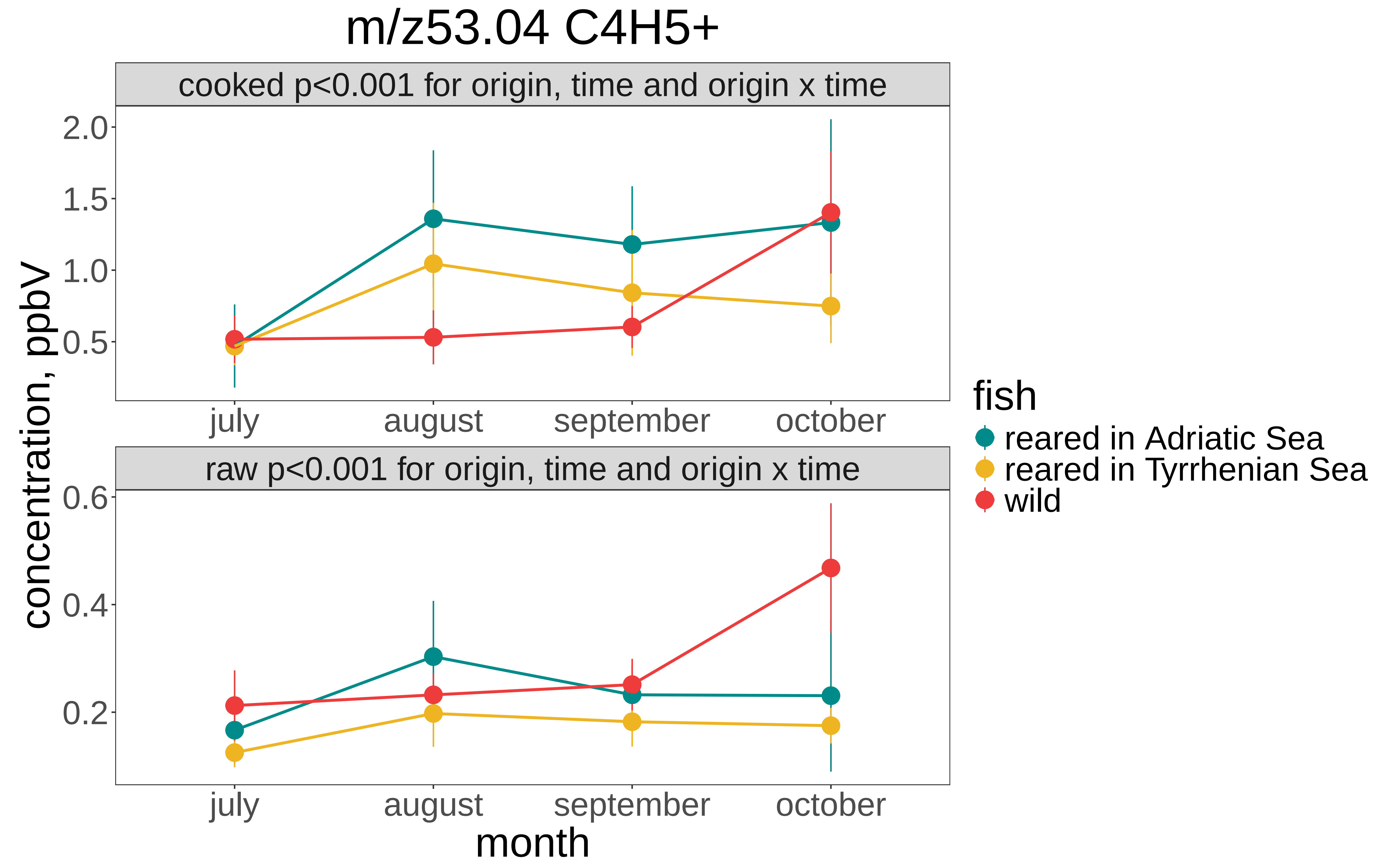

m/z53.04 C4H5+
cooked p<0.001 for origin, time and origin x time
raw p<0.001 for origin, time and origin x time
concentration, ppbV
2.0
1.5
1.0
0.5
0.6
0.4
0.2
july
august
september
october
month
fish
reared in Adriatic Sea
reared in Tyrrhenian Sea
wild

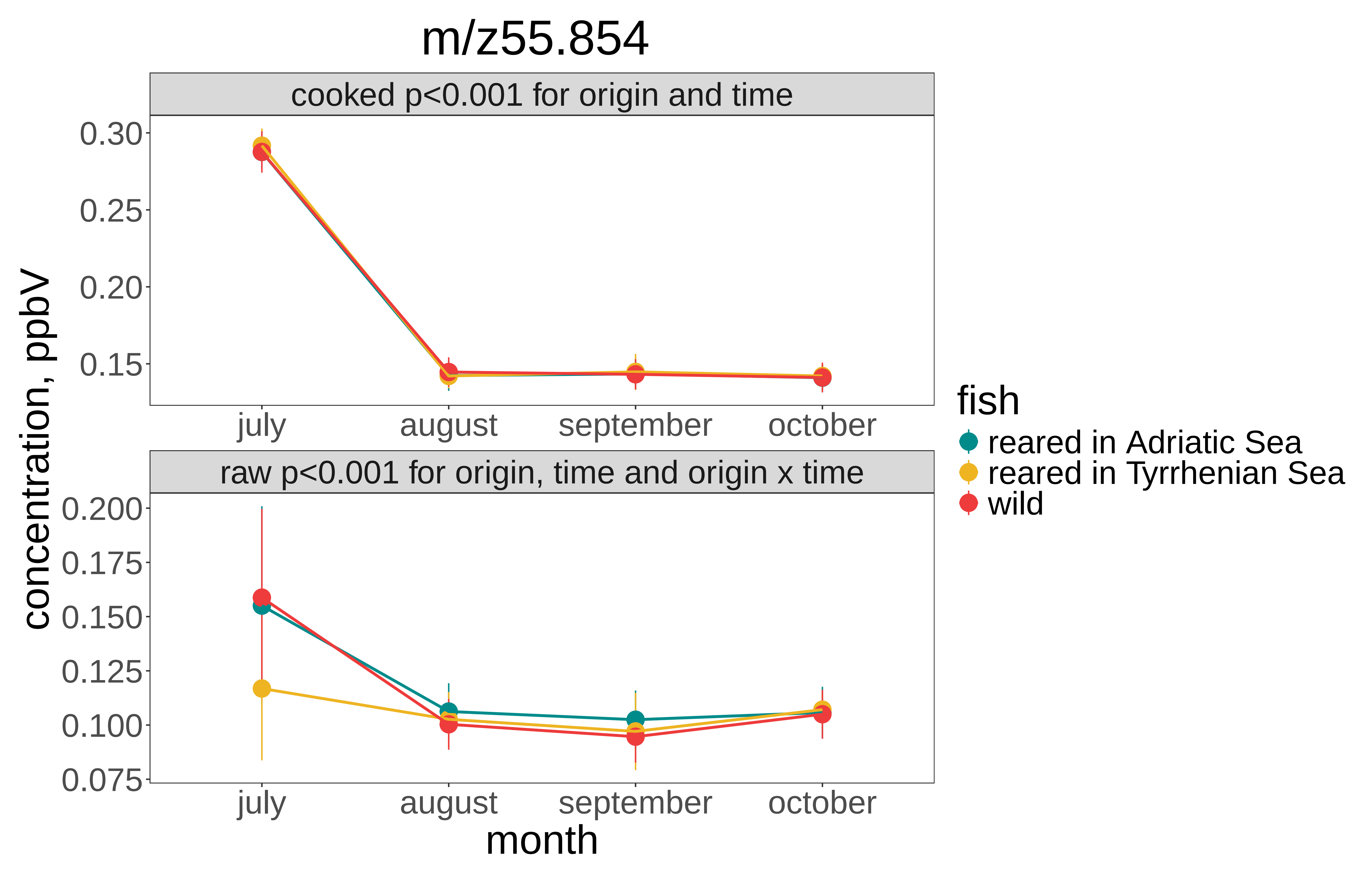
m/z55.854
cooked p<0.001 for origin and time
0.30
0.25
0.20
0.15
july
august
september
october
raw p<0.001 for origin, time and origin x time
0.200
0.175
0.150
0.125
0.100
0.075
july
august
september
october
concentration, ppbV
month
fish
reared in Adriatic Sea
reared in Tyrrhenian Sea
wild

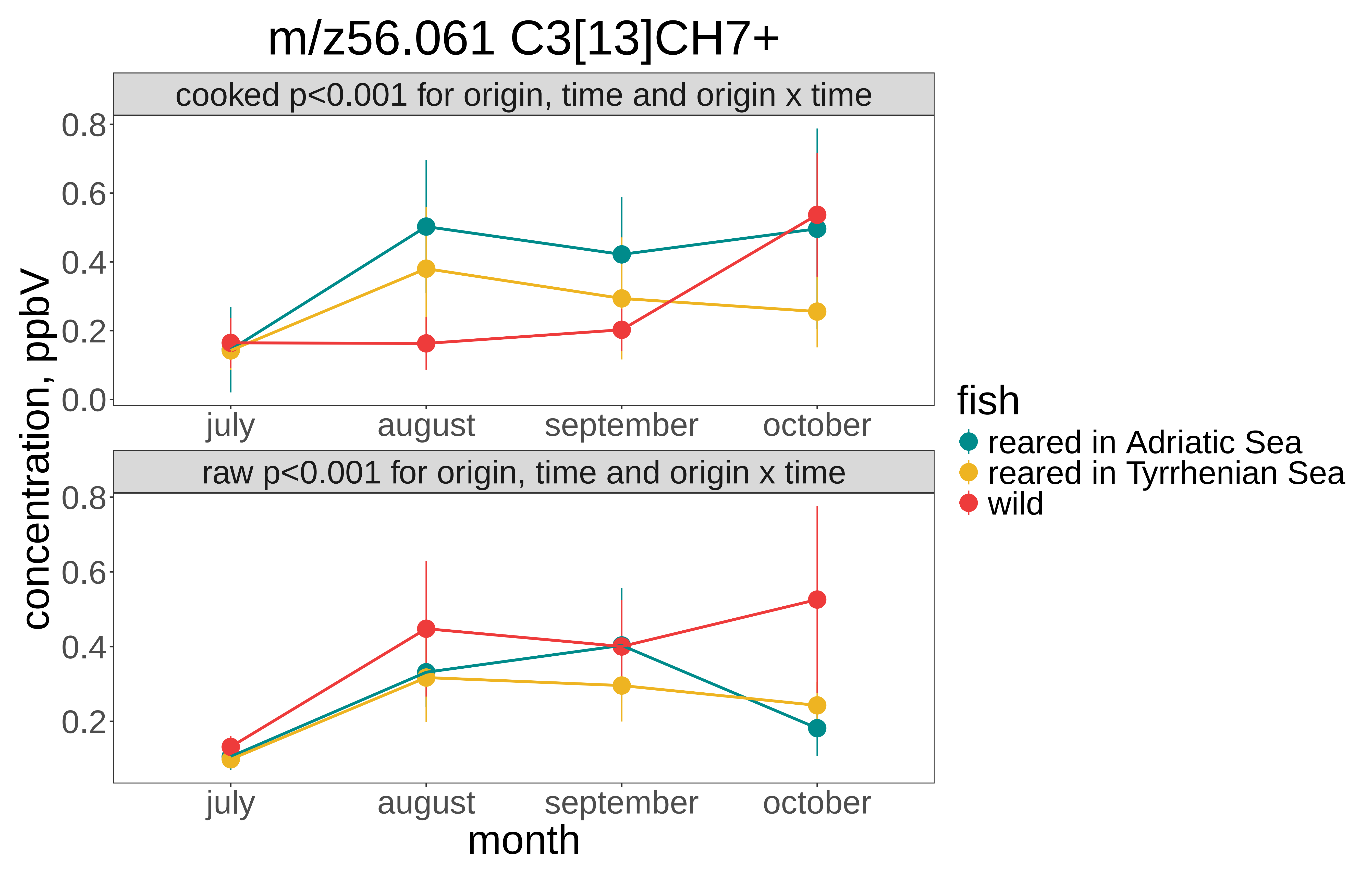
m/z56.061 C3[13]CH7+
cooked p<0.001 for origin, time and origin x time
raw p<0.001 for origin, time and origin x time
concentration, ppbV
0.0
0.2
0.4
0.6
0.8
july
august
september
october
month
fish
reared in Adriatic Sea
reared in Tyrrhenian Sea
wild

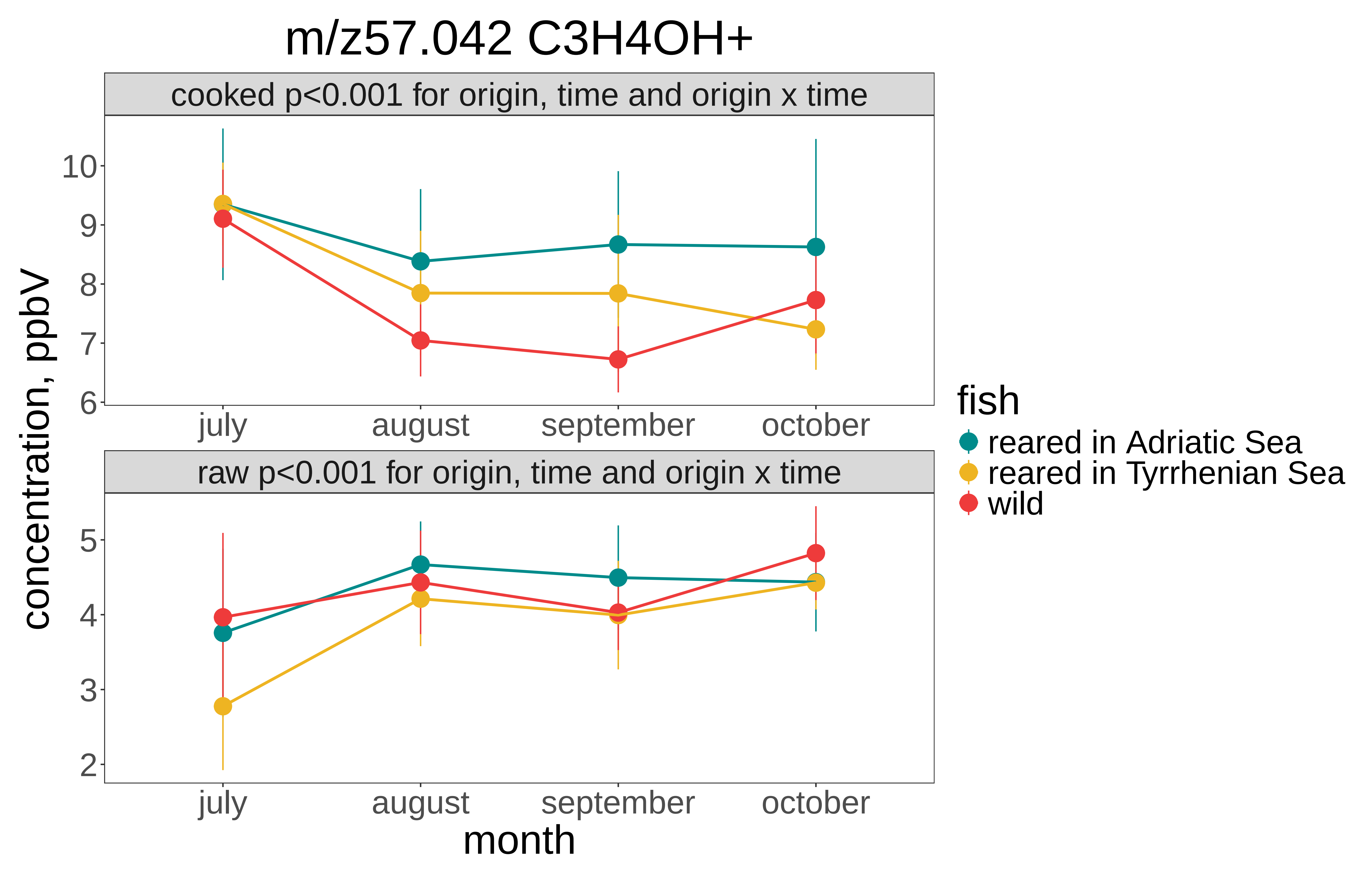
m/z57.042 C3H4OH+
cooked p<0.001 for origin, time and origin x time
raw p<0.001 for origin, time and origin x time
concentration, ppbV
10
9
8
7
6
5
4
3
2
july
august
september
october
month
fish
reared in Adriatic Sea
reared in Tyrrhenian Sea
wild

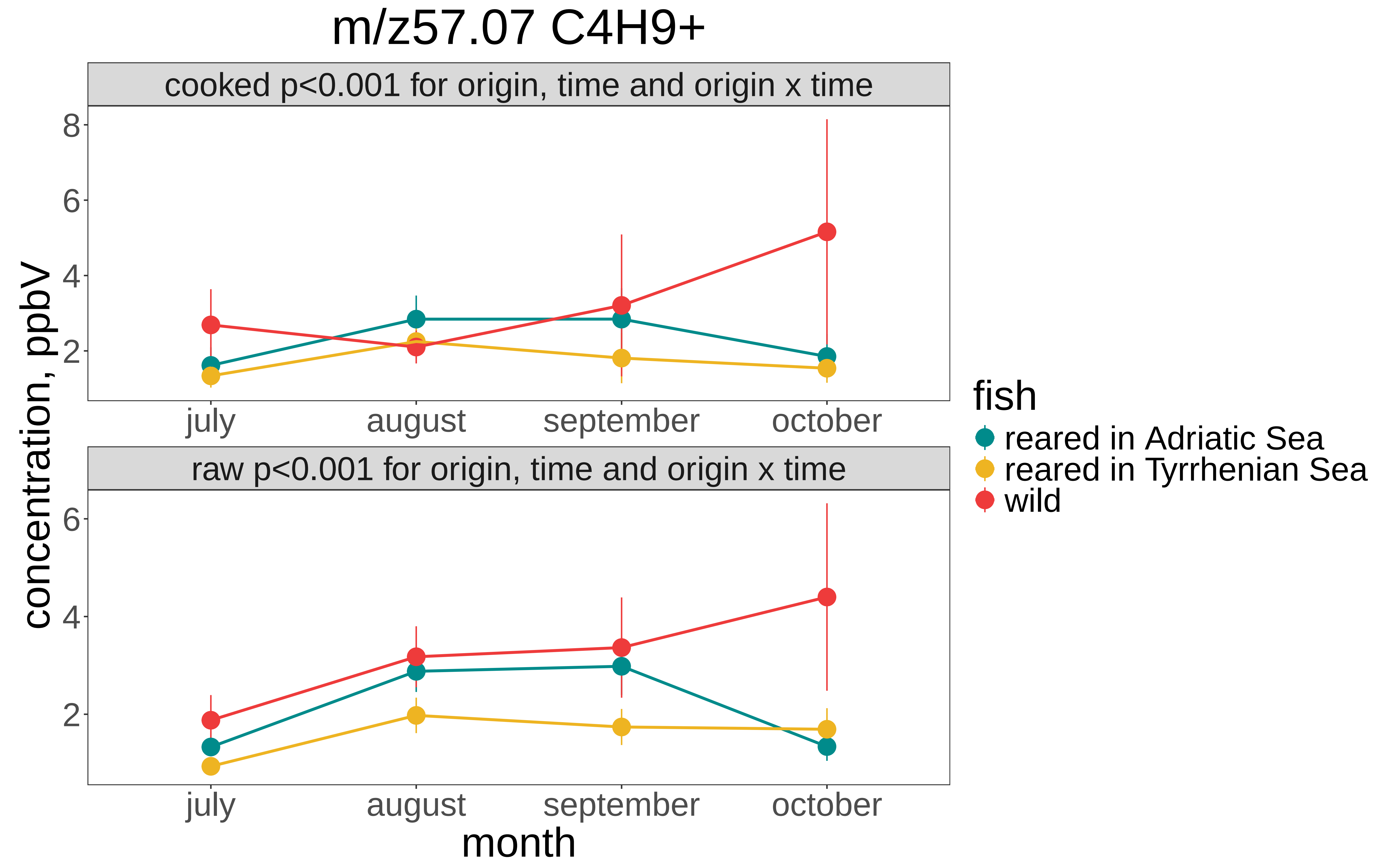
m/z57.07 C4H9+
cooked p<0.001 for origin, time and origin x time
raw p<0.001 for origin, time and origin x time
concentration, ppbV
month
july
august
september
october
fish
reared in Adriatic Sea
reared in Tyrrhenian Sea
wild

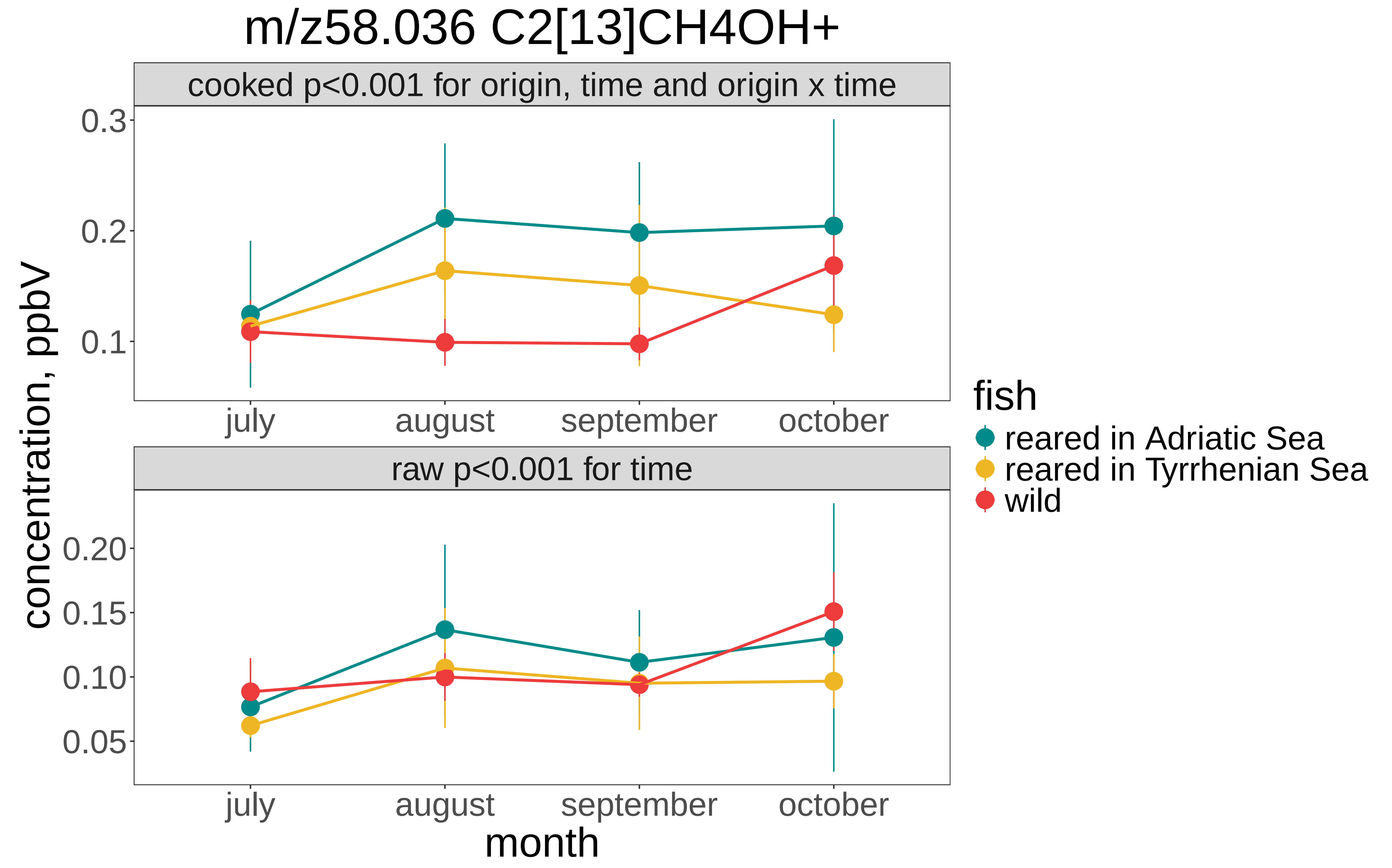

m/z58.036 C2[13]CH4OH+
cooked p<0.001 for origin, time and origin x time
raw p<0.001 for time
concentration, ppbV
0.3
0.2
0.1
0.20
0.15
0.10
0.05
july
august
september
october
month
fish
reared in Adriatic Sea
reared in Tyrrhenian Sea
wild

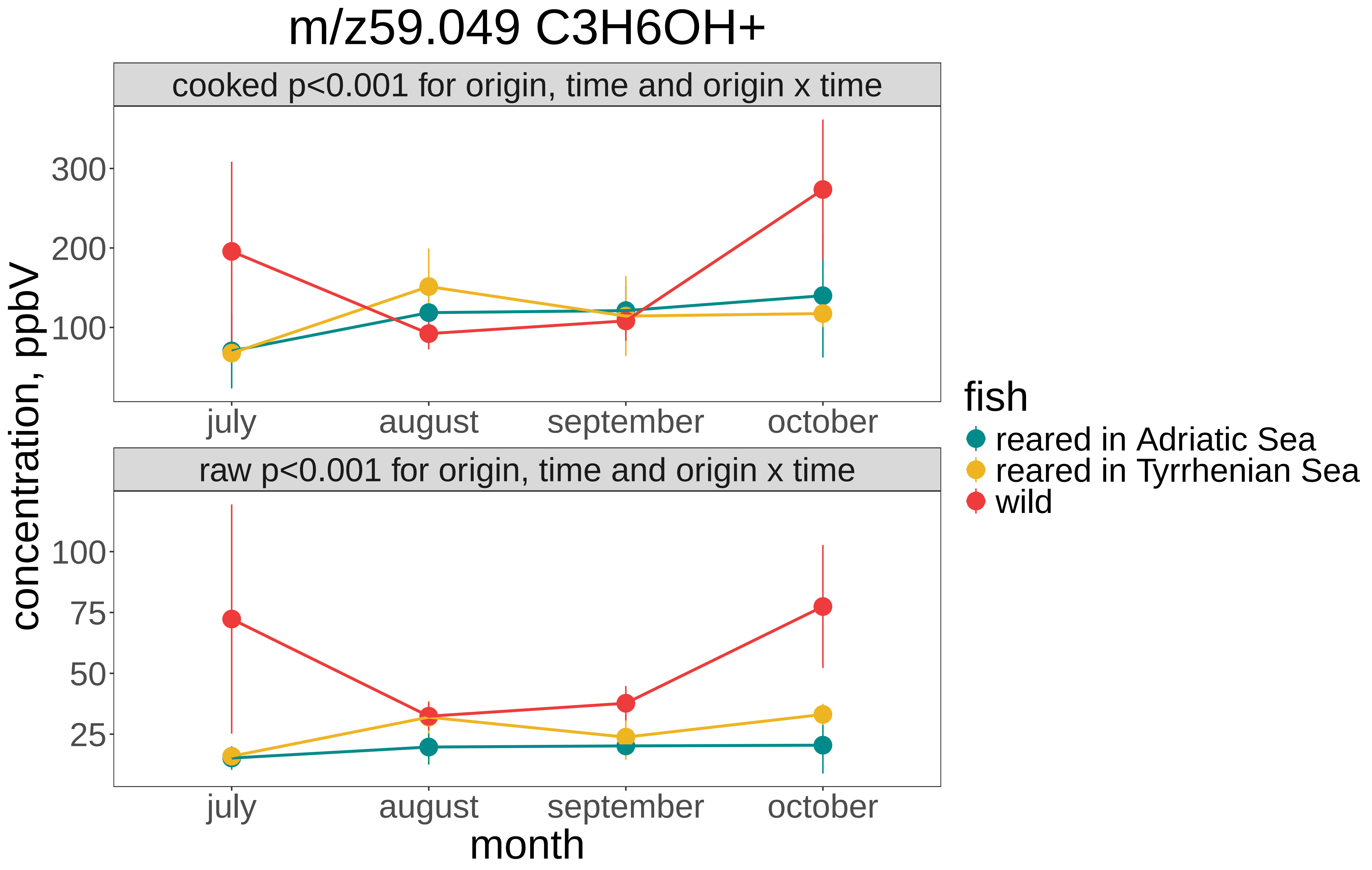
m/z59.049 C3H6OH+
cooked p<0.001 for origin, time and origin x time
raw p<0.001 for origin, time and origin x time
concentration, ppbV
300
200
100
100
75
50
25
july
august
september
october
month
fish
reared in Adriatic Sea
reared in Tyrrhenian Sea
wild

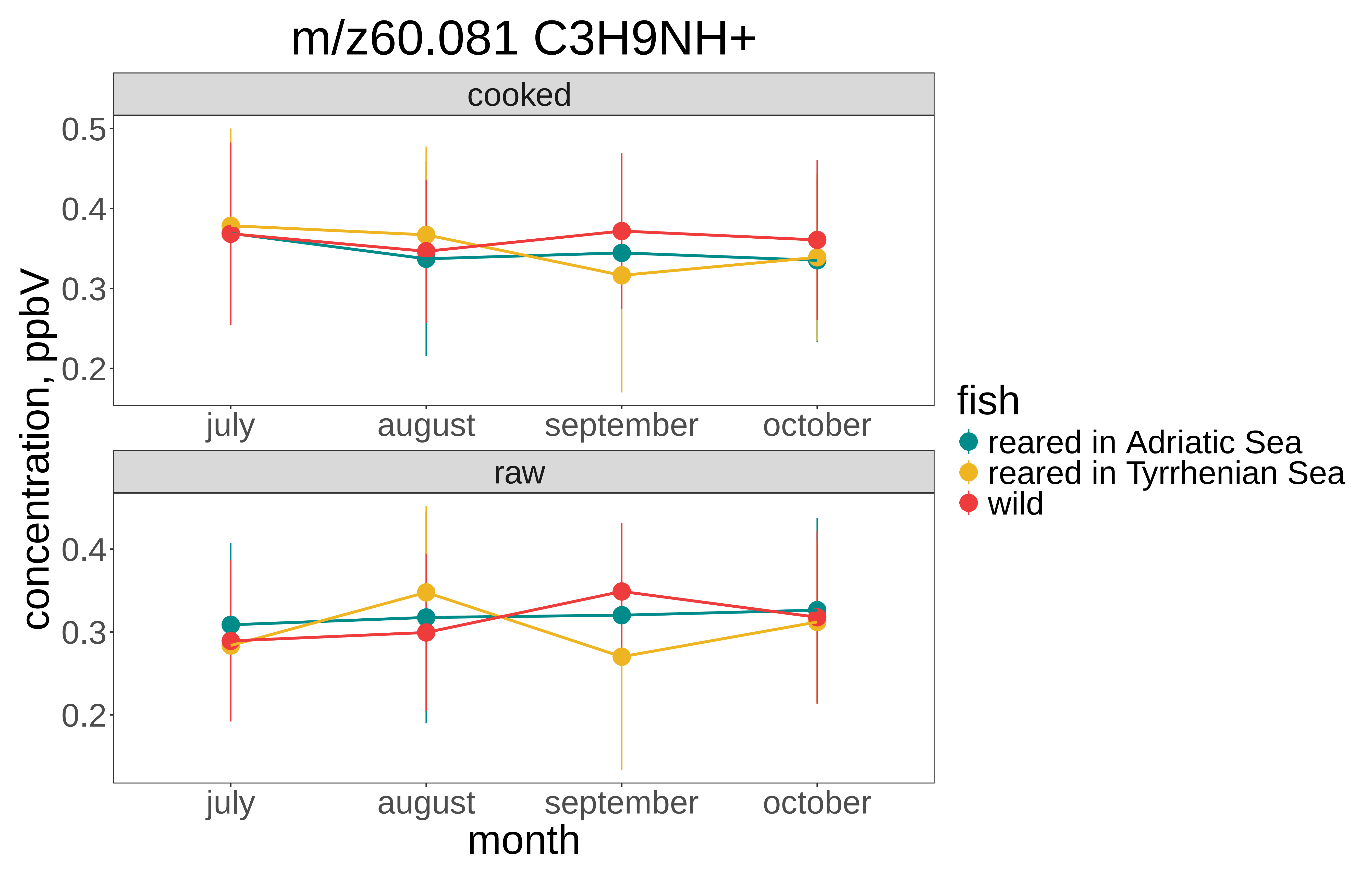

m/z60.081 C3H9NH+
cooked
raw
0.5
0.4
0.3
0.2
july
august
september
october
concentration, ppbV
month
fish
reared in Adriatic Sea
reared in Tyrrhenian Sea
wild

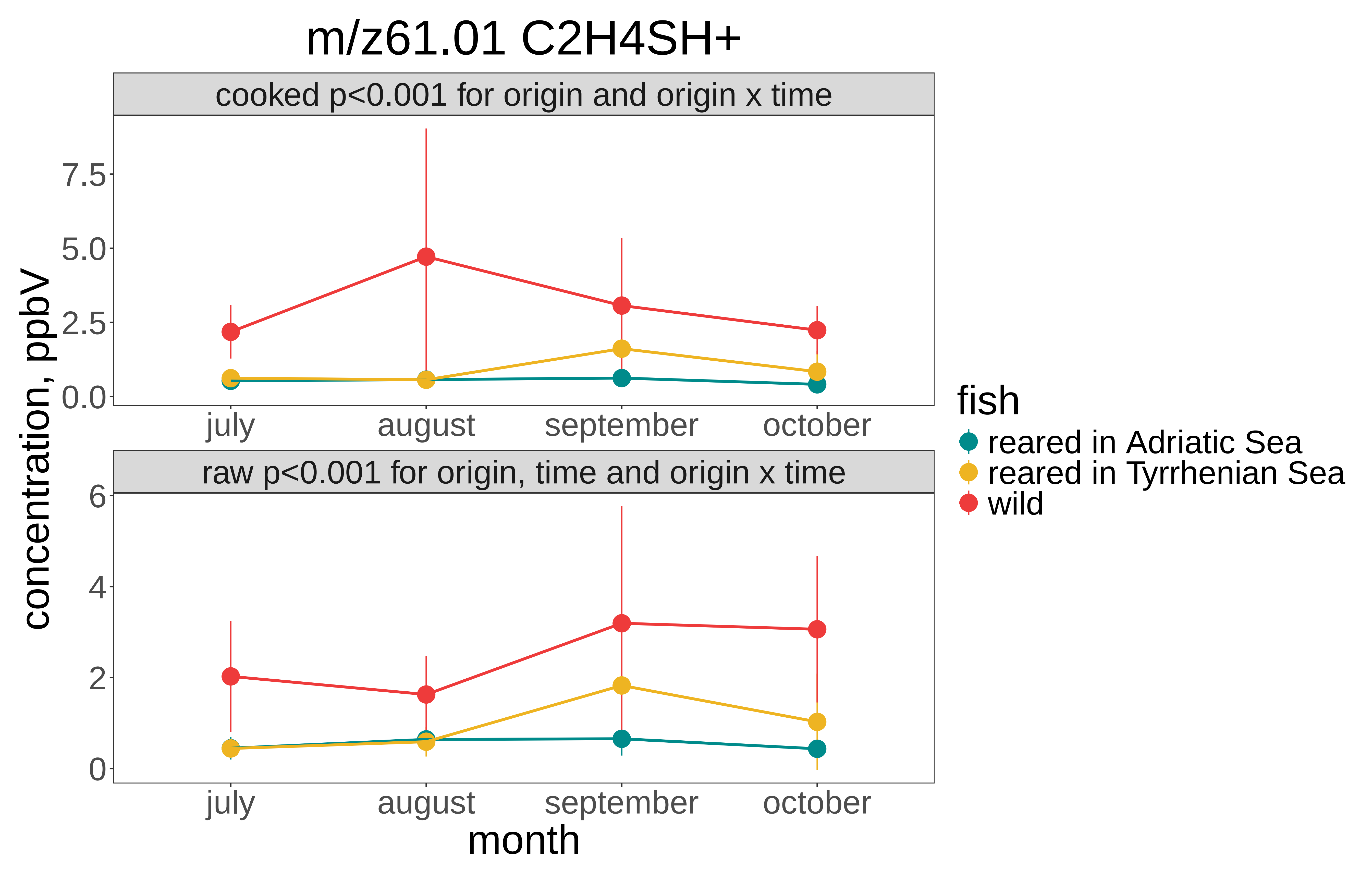
m/z61.01 C2H4SH+
cooked p<0.001 for origin and origin x time
raw p<0.001 for origin, time and origin x time
concentration, ppbV
7.5
5.0
2.5
0.0
6
4
2
0
july
august
september
october
month
fish
reared in Adriatic Sea
reared in Tyrrhenian Sea
wild

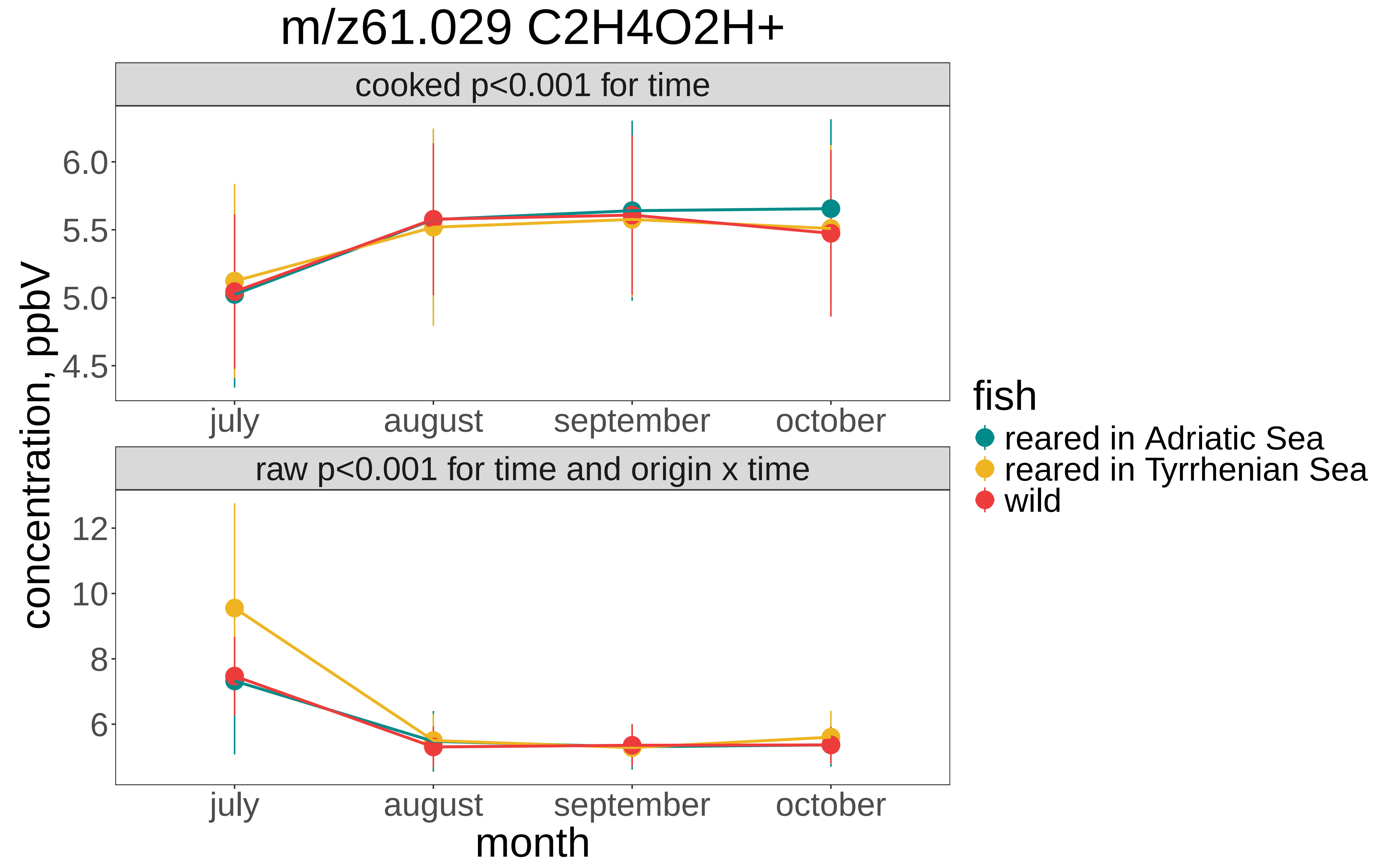
m/z61.029 C2H4O2H+
cooked p<0.001 for time
raw p<0.001 for time and origin x time
concentration, ppbV
6.0
5.5
5.0
4.5
12
10
8
6
july
august
september
october
month
fish
reared in Adriatic Sea
reared in Tyrrhenian Sea
wild

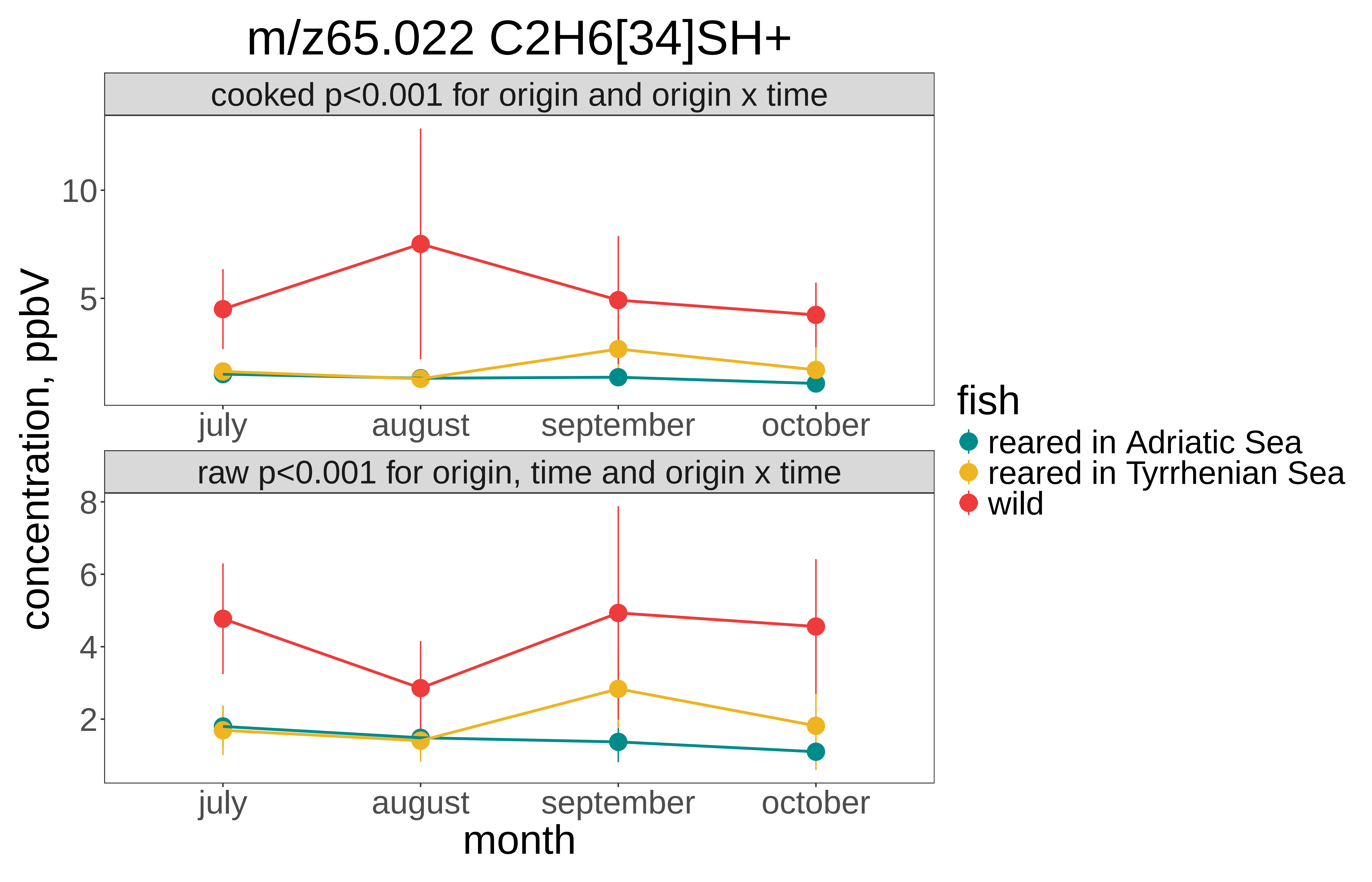

m/z65.022 C2H6[34]SH+
cooked p<0.001 for origin and origin x time
raw p<0.001 for origin, time and origin x time
concentration, ppbV
10
5
8
6
4
2
july
august
september
october
month
fish
reared in Adriatic Sea
reared in Tyrrhenian Sea
wild

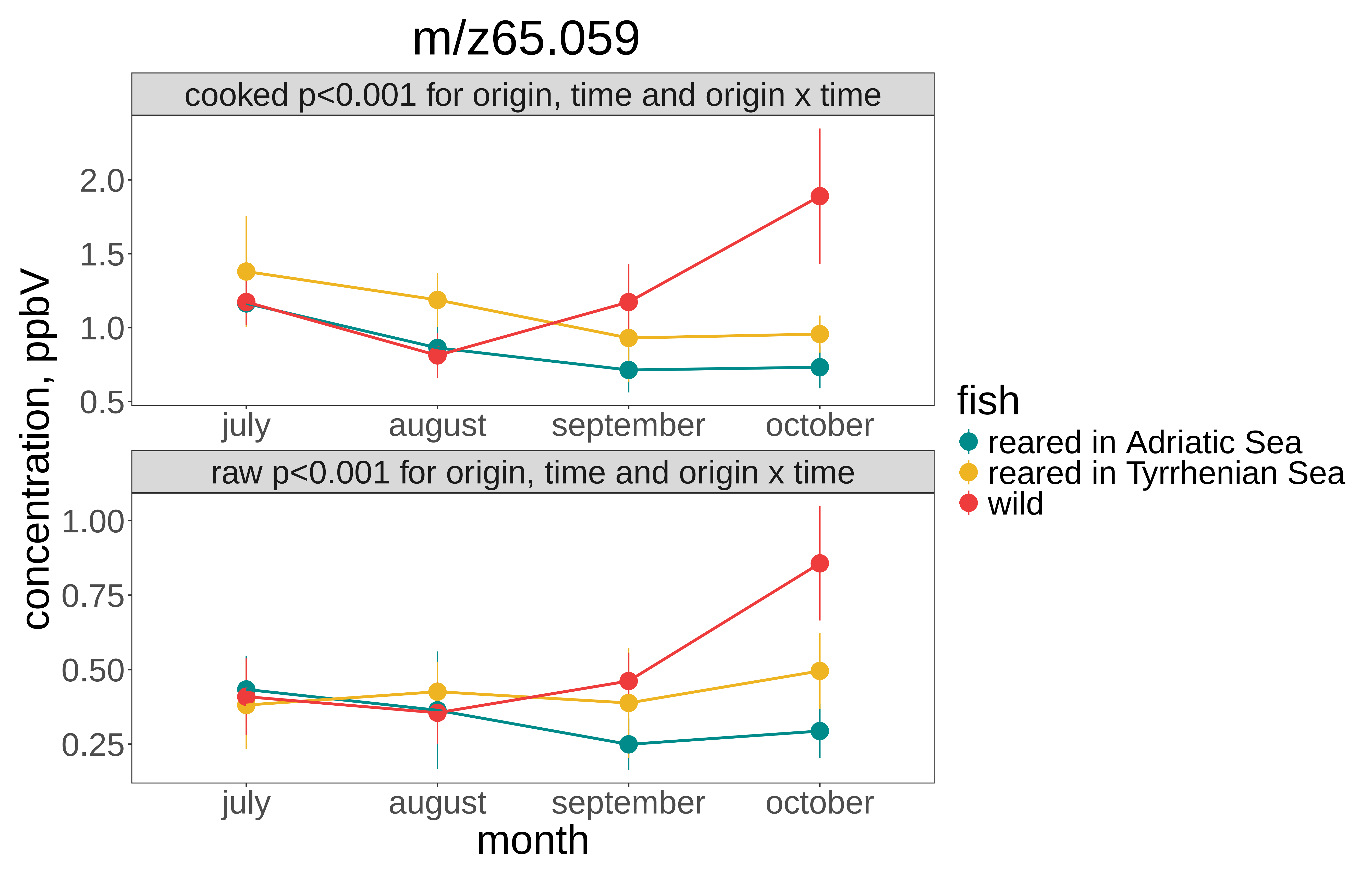
m/z65.059
cooked p<0.001 for origin, time and origin x time
raw p<0.001 for origin, time and origin x time
concentration, ppbV
2.0
1.5
1.0
0.5
1.00
0.75
0.50
0.25
july
august
september
october
month
fish
reared in Adriatic Sea
reared in Tyrrhenian Sea
wild

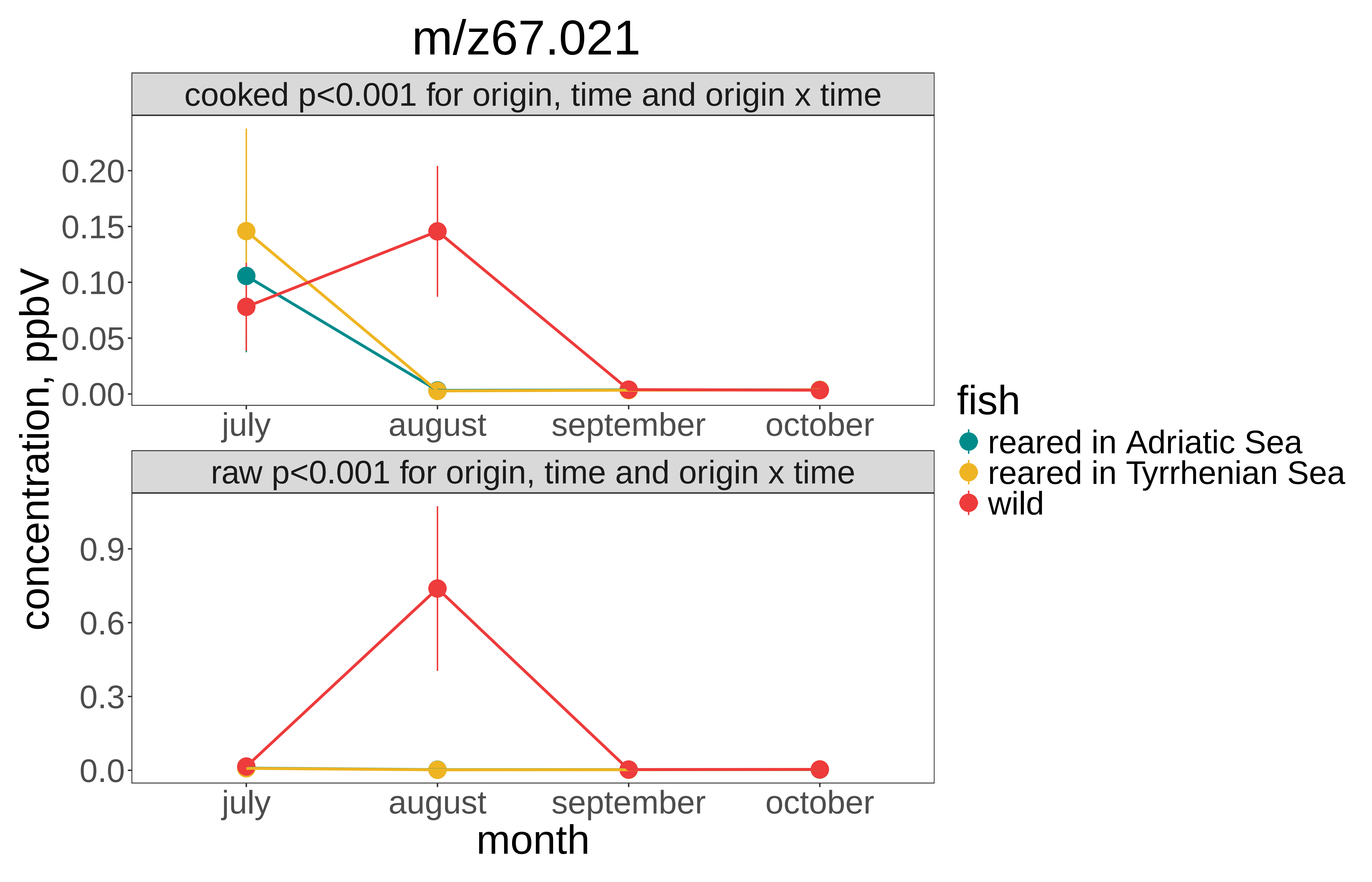
m/z67.021
cooked p<0.001 for origin, time and origin x time
raw p<0.001 for origin, time and origin x time
concentration, ppbV
0.20
0.15
0.10
0.05
0.00
0.9
0.6
0.3
0.0
july
august
september
october
month
fish
reared in Adriatic Sea
reared in Tyrrhenian Sea
wild

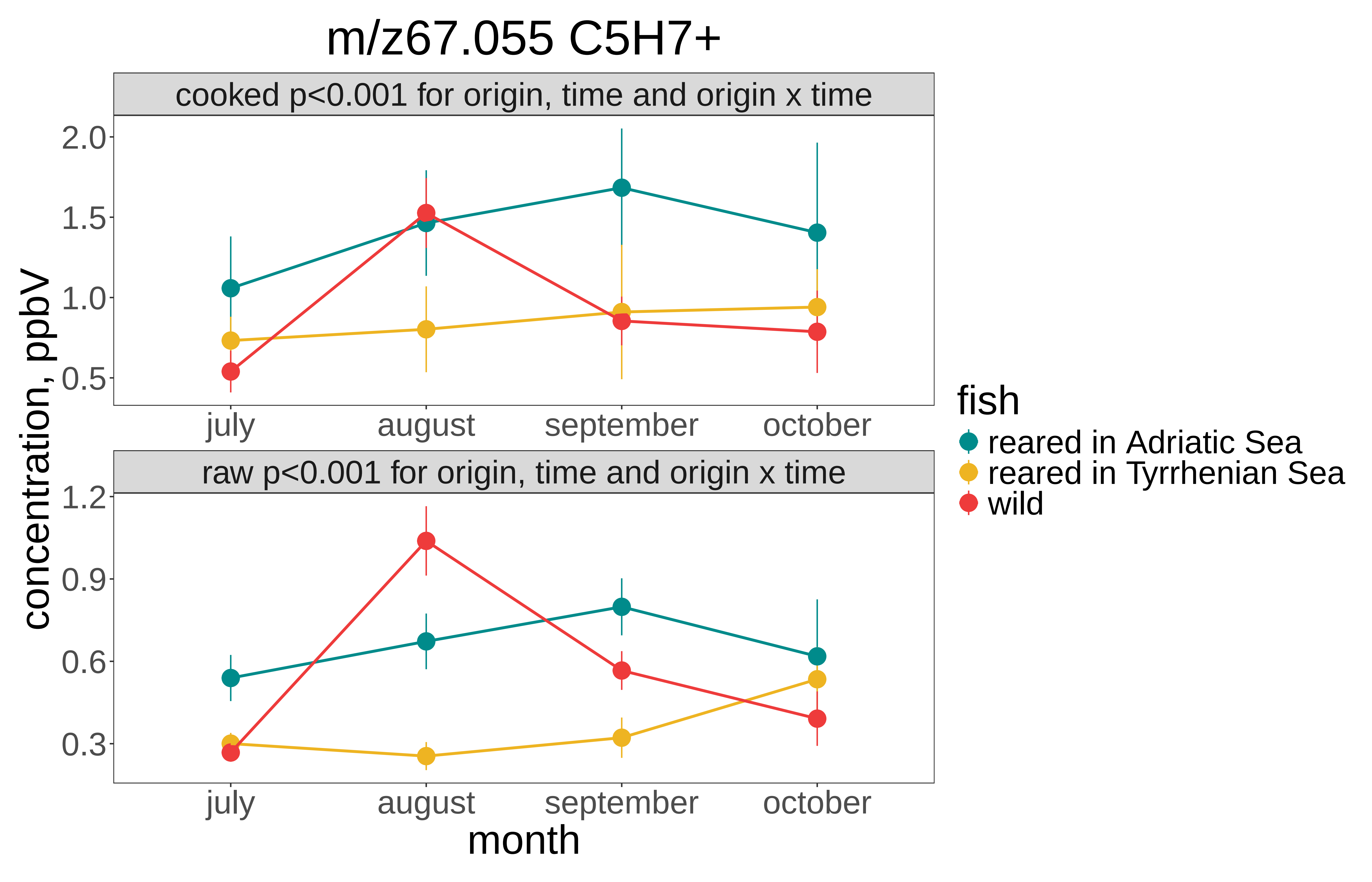

m/z67.055 C5H7+
cooked p<0.001 for origin, time and origin x time
raw p<0.001 for origin, time and origin x time
concentration, ppbV
2.0
1.5
1.0
0.5
1.2
0.9
0.6
0.3
july
august
september
october
month
fish
reared in Adriatic Sea
reared in Tyrrhenian Sea
wild

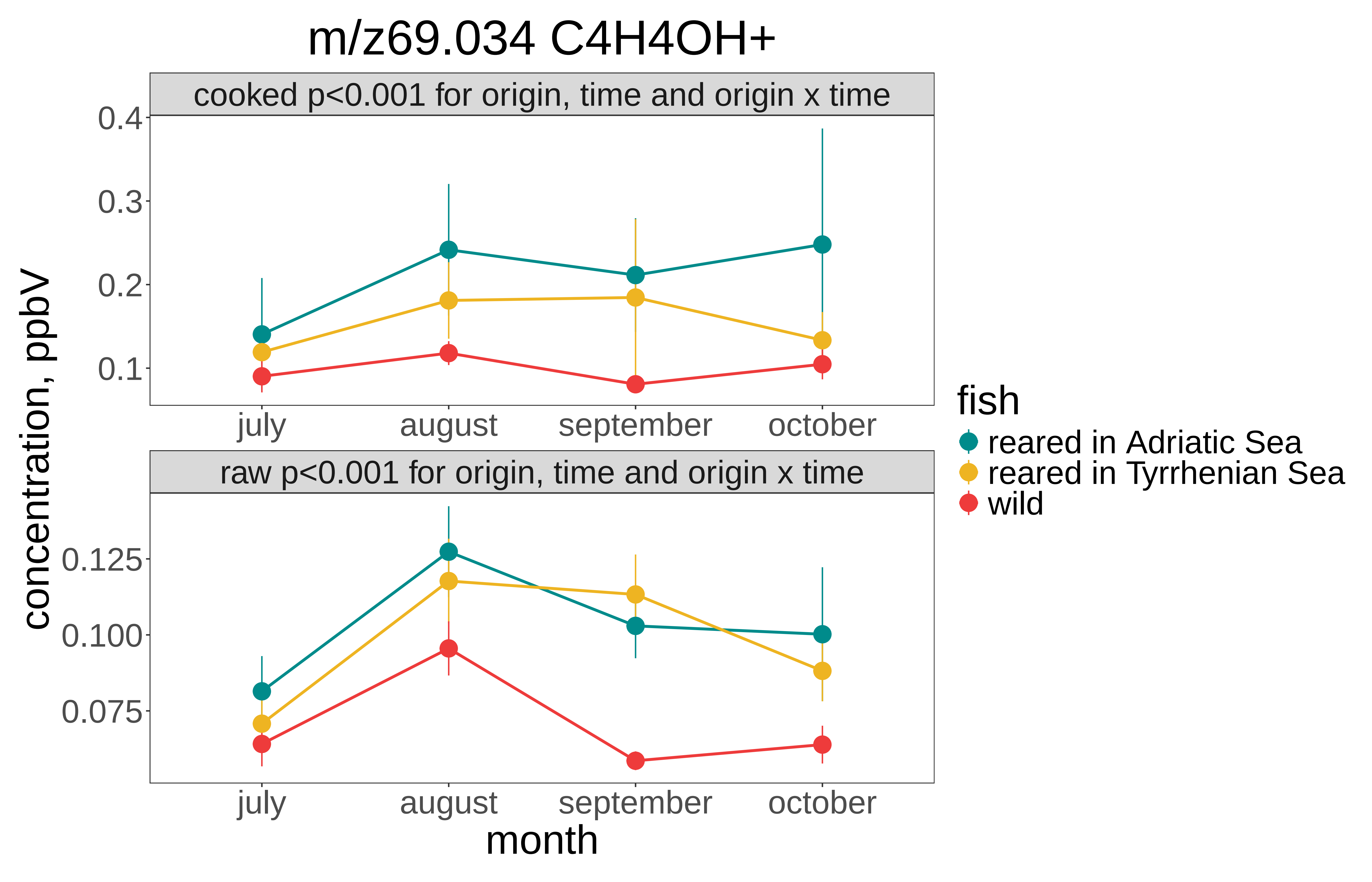
m/z69.034 C4H4OH+
cooked p<0.001 for origin, time and origin x time
raw p<0.001 for origin, time and origin x time
concentration, ppbV
0.4
0.3
0.2
0.1
0.125
0.100
0.075
july
august
september
october
month
fish
reared in Adriatic Sea
reared in Tyrrhenian Sea
wild

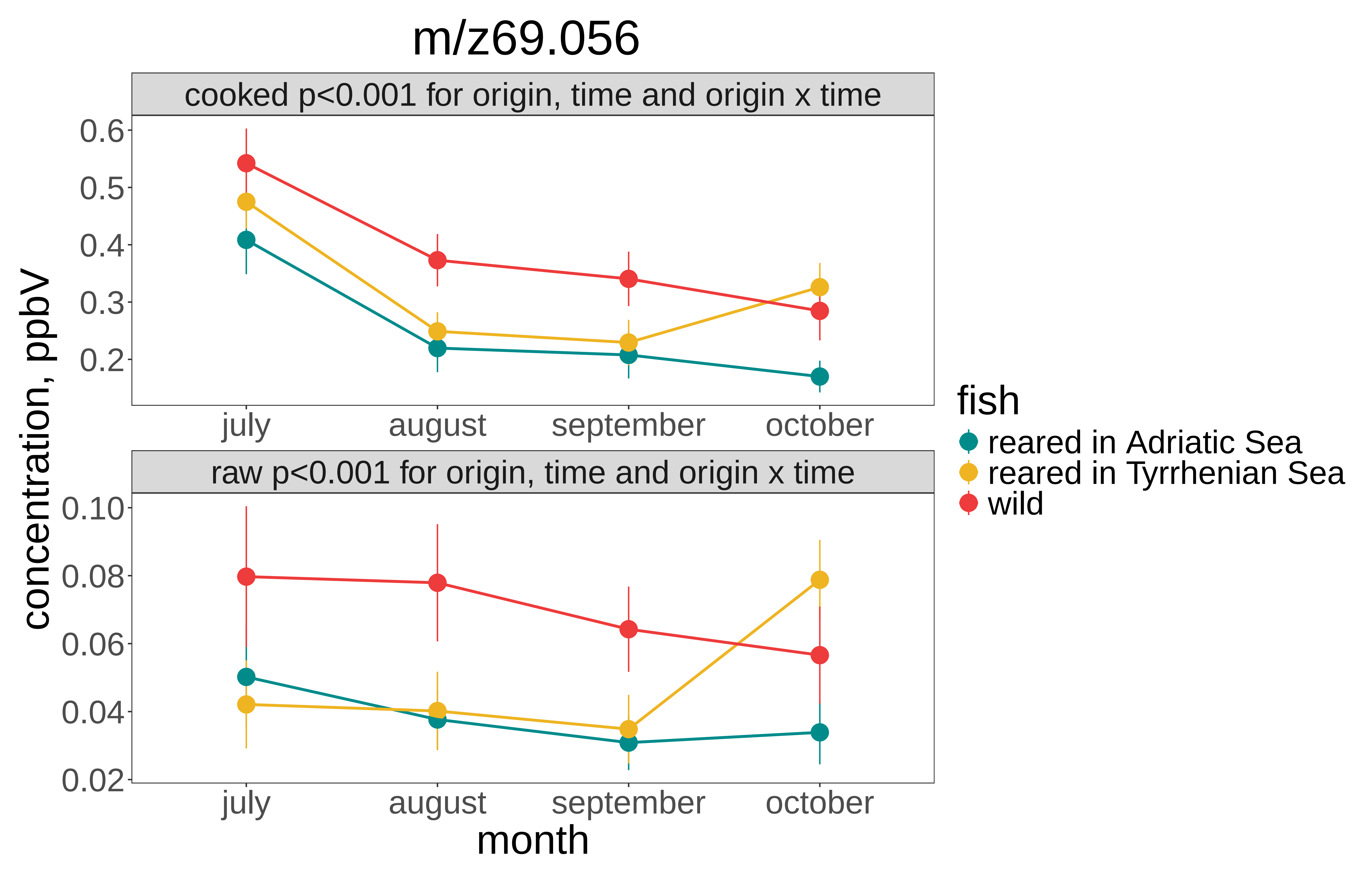
m/z69.056
cooked p<0.001 for origin, time and origin x time
raw p<0.001 for origin, time and origin x time
concentration, ppbV
0.6
0.5
0.4
0.3
0.2
0.10
0.08
0.06
0.04
0.02
july
august
september
october
month
fish
reared in Adriatic Sea
reared in Tyrrhenian Sea
wild

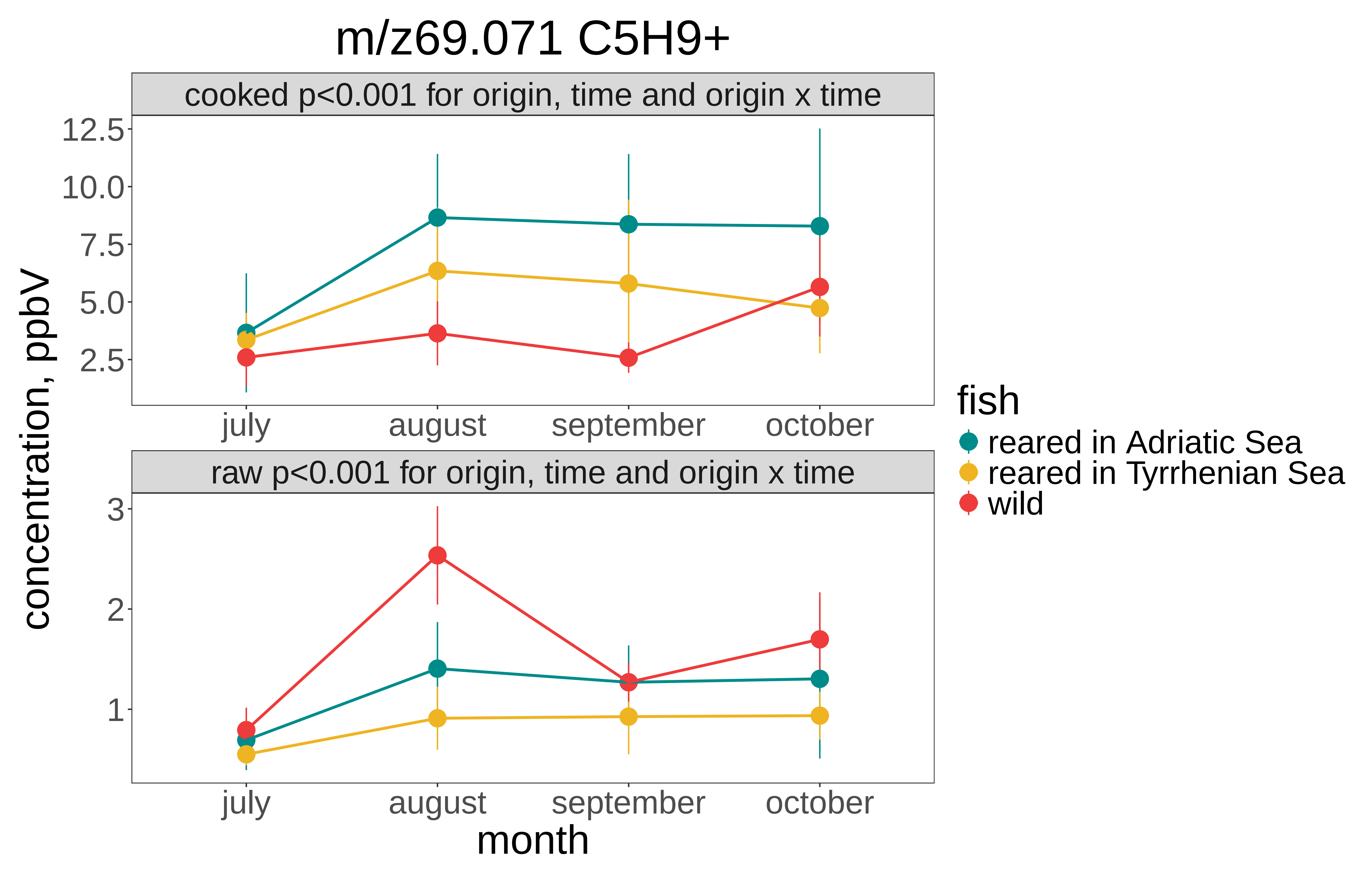
m/z69.071 C5H9+
cooked p<0.001 for origin, time and origin x time
raw p<0.001 for origin, time and origin x time
concentration, ppbV
12.5
10.0
7.5
5.0
2.5
3
2
1
july
august
september
october
month
fish
reared in Adriatic Sea
reared in Tyrrhenian Sea
wild

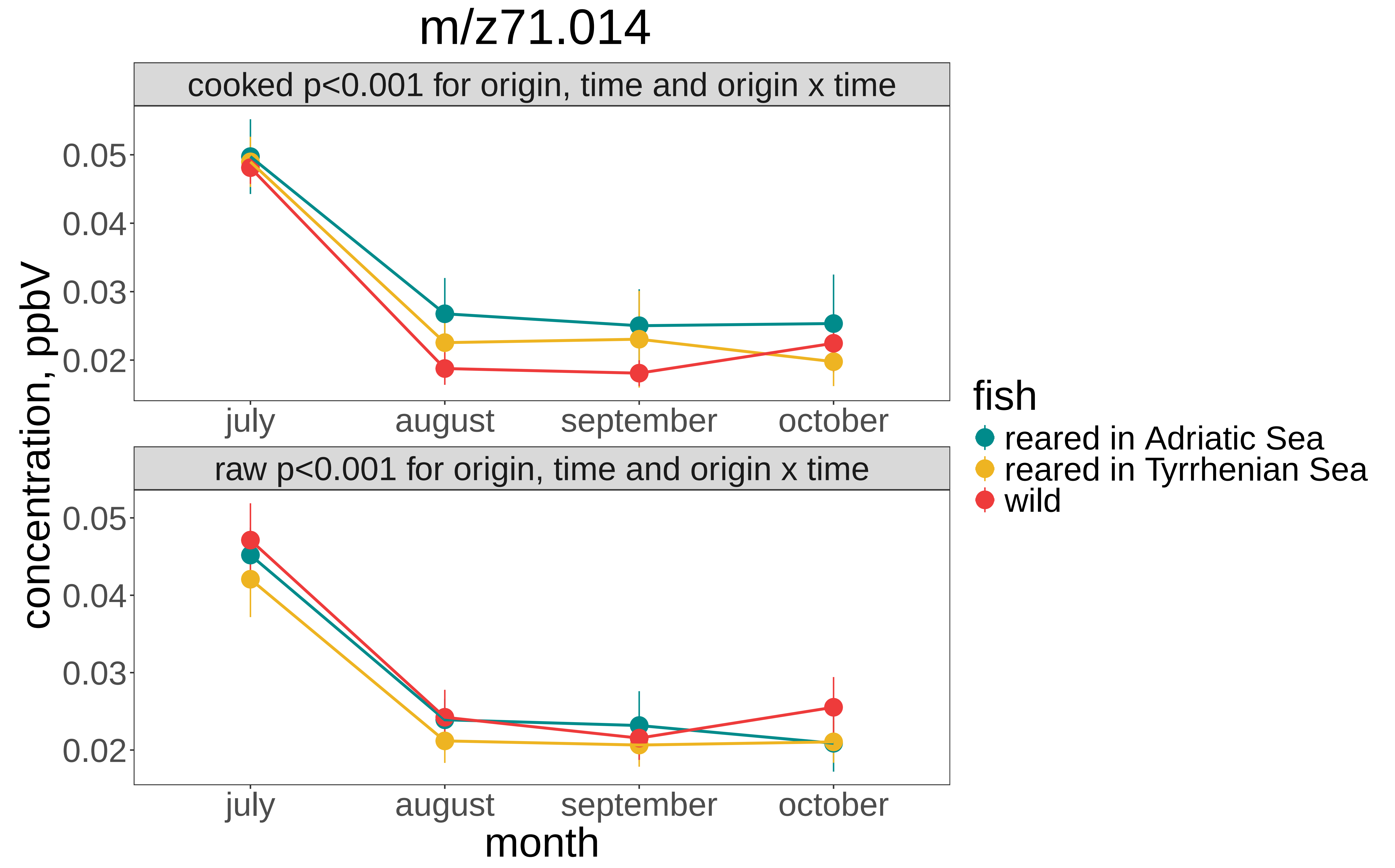
m/z71.014
cooked p<0.001 for origin, time and origin x time
raw p<0.001 for origin, time and origin x time
concentration, ppbV
0.05
0.04
0.03
0.02
july
august
september
october
month
fish
reared in Adriatic Sea
reared in Tyrrhenian Sea
wild

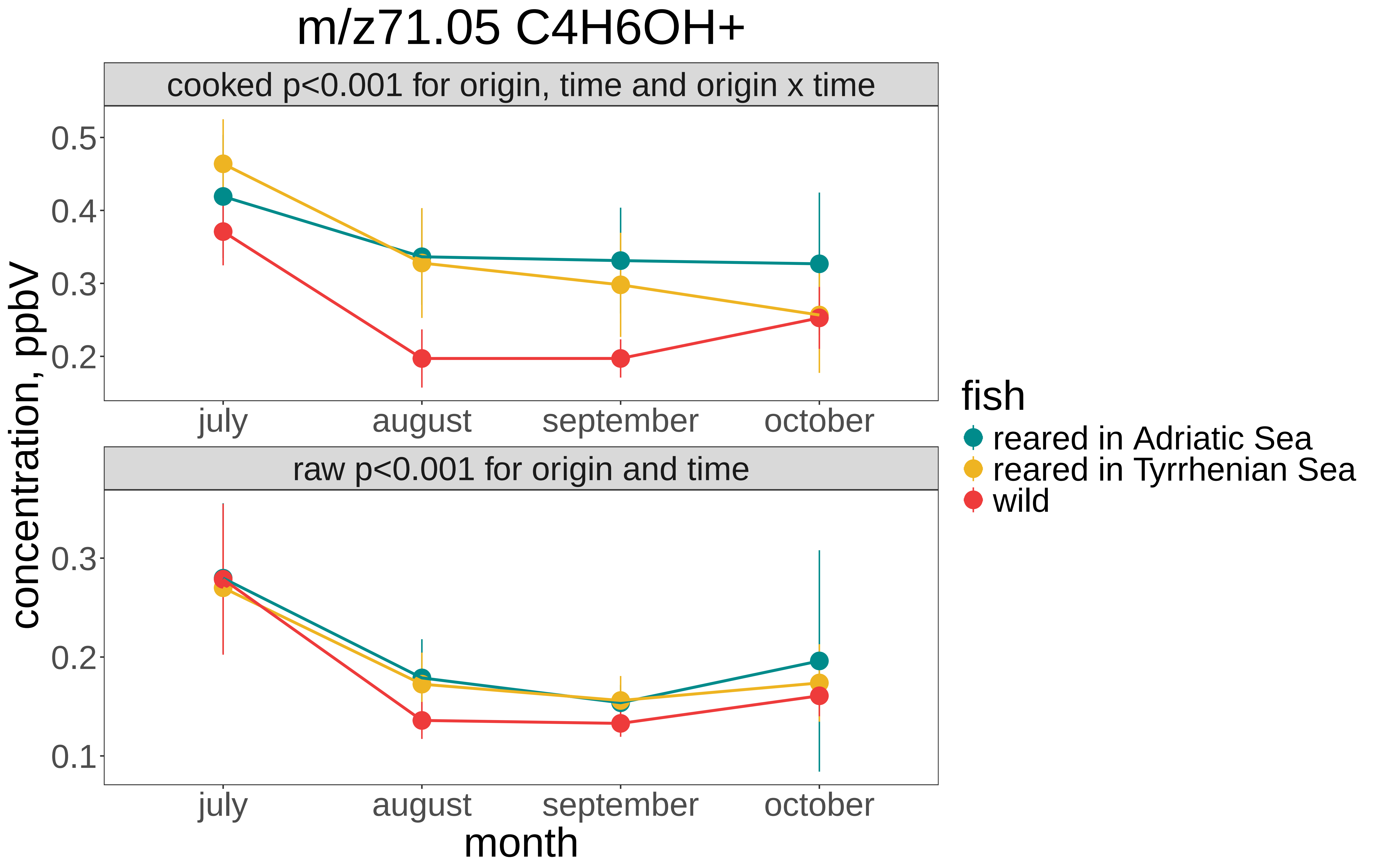
m/z71.05 C4H6OH+
cooked p<0.001 for origin, time and origin x time
raw p<0.001 for origin and time
concentration, ppbV
0.5
0.4
0.3
0.2
0.1
july
august
september
october
month
fish
reared in Adriatic Sea
reared in Tyrrhenian Sea
wild

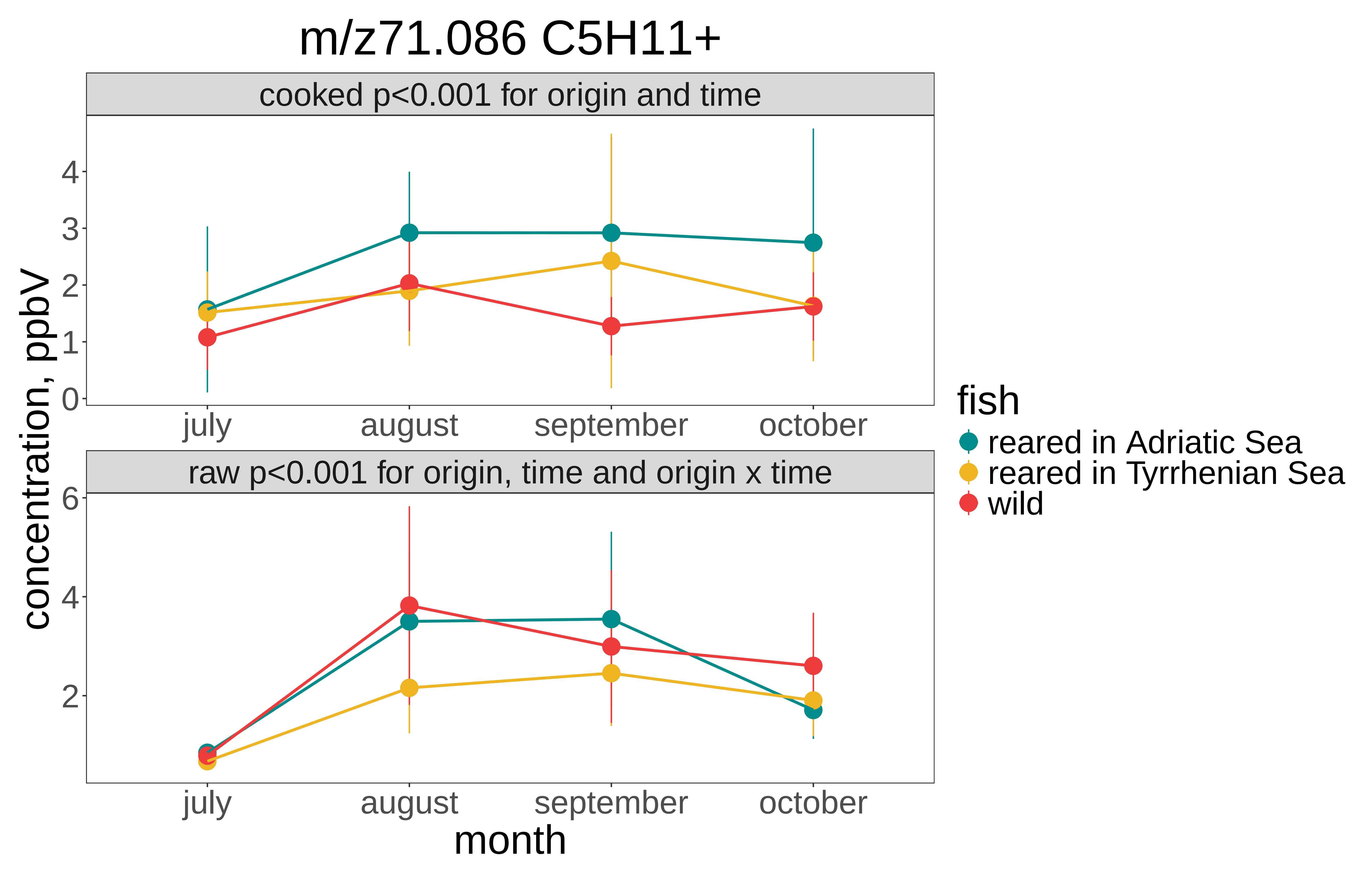
m/z71.086 C5H11+
cooked p<0.001 for origin and time
raw p<0.001 for origin, time and origin x time
concentration, ppbV
0
1
2
3
4
6
july
august
september
october
month
fish
reared in Adriatic Sea
reared in Tyrrhenian Sea
wild

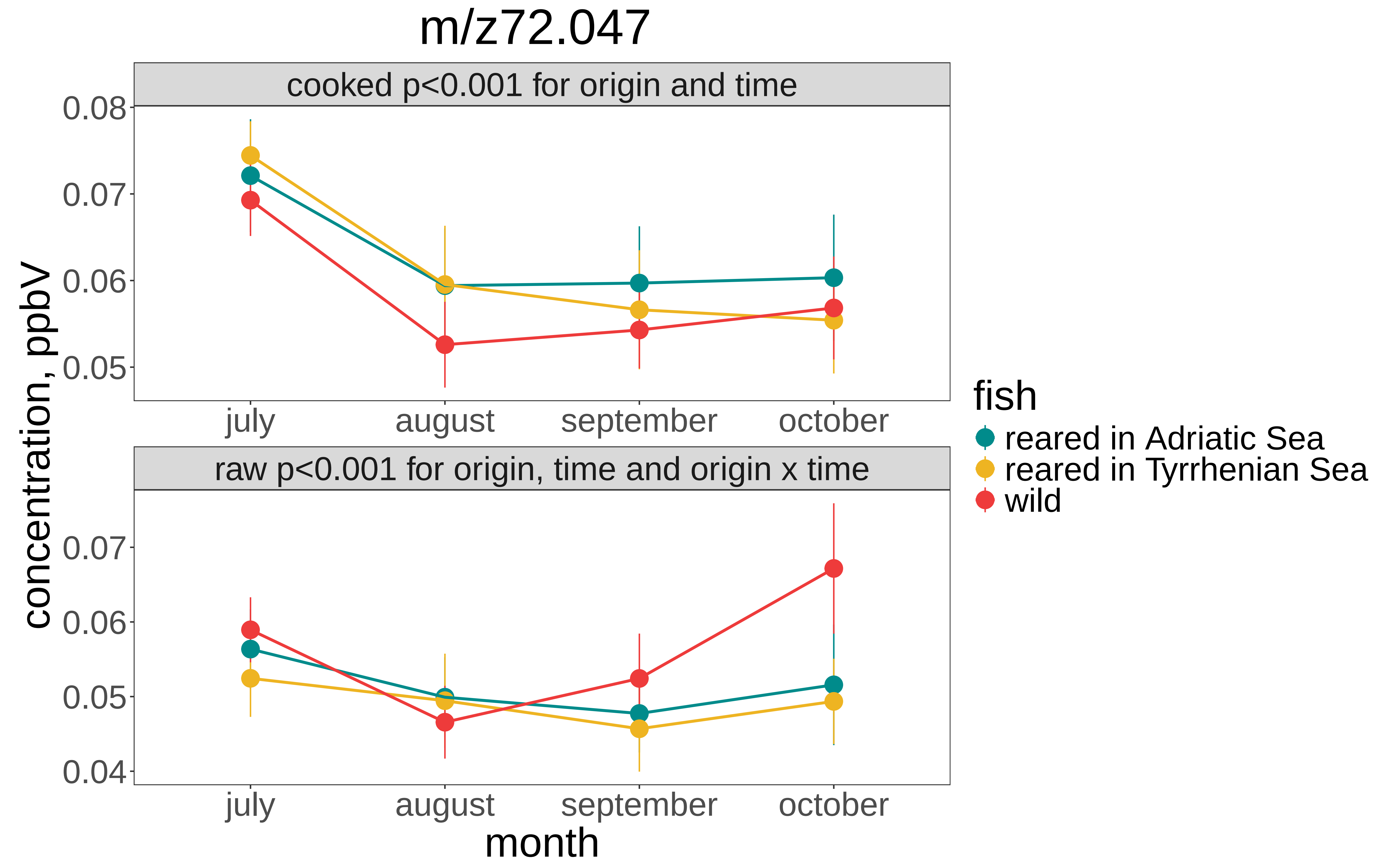
m/z72.047
cooked p<0.001 for origin and time
raw p<0.001 for origin, time and origin x time
concentration, ppbV
0.08
0.07
0.06
0.05
0.04
july
august
september
october
month
fish
reared in Adriatic Sea
reared in Tyrrhenian Sea
wild

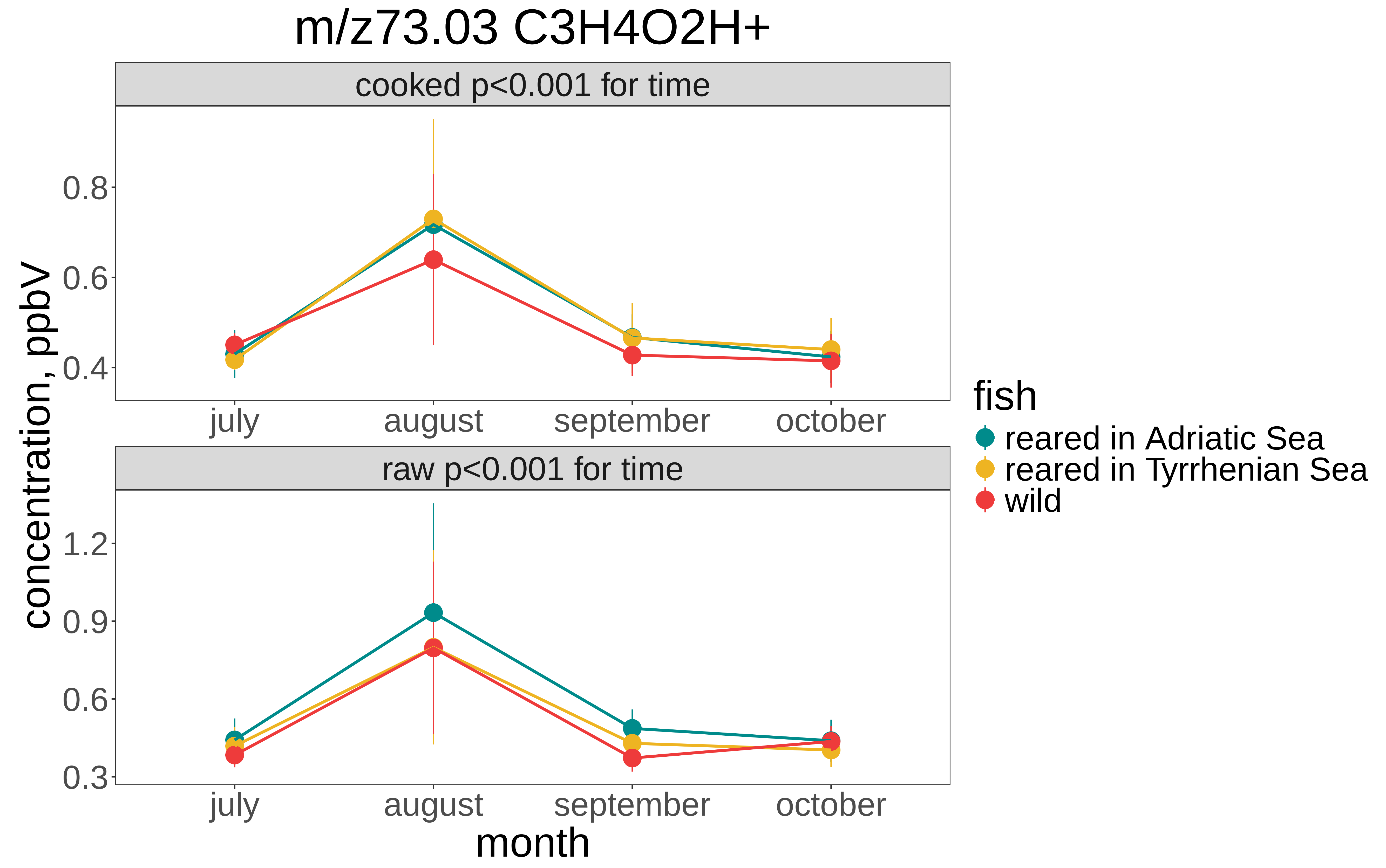
m/z73.03 C3H4O2H+
cooked p<0.001 for time
raw p<0.001 for time
concentration, ppbV
0.8
0.6
0.4
1.2
0.9
0.6
0.3
july
august
september
october
month
fish
reared in Adriatic Sea
reared in Tyrrhenian Sea
wild

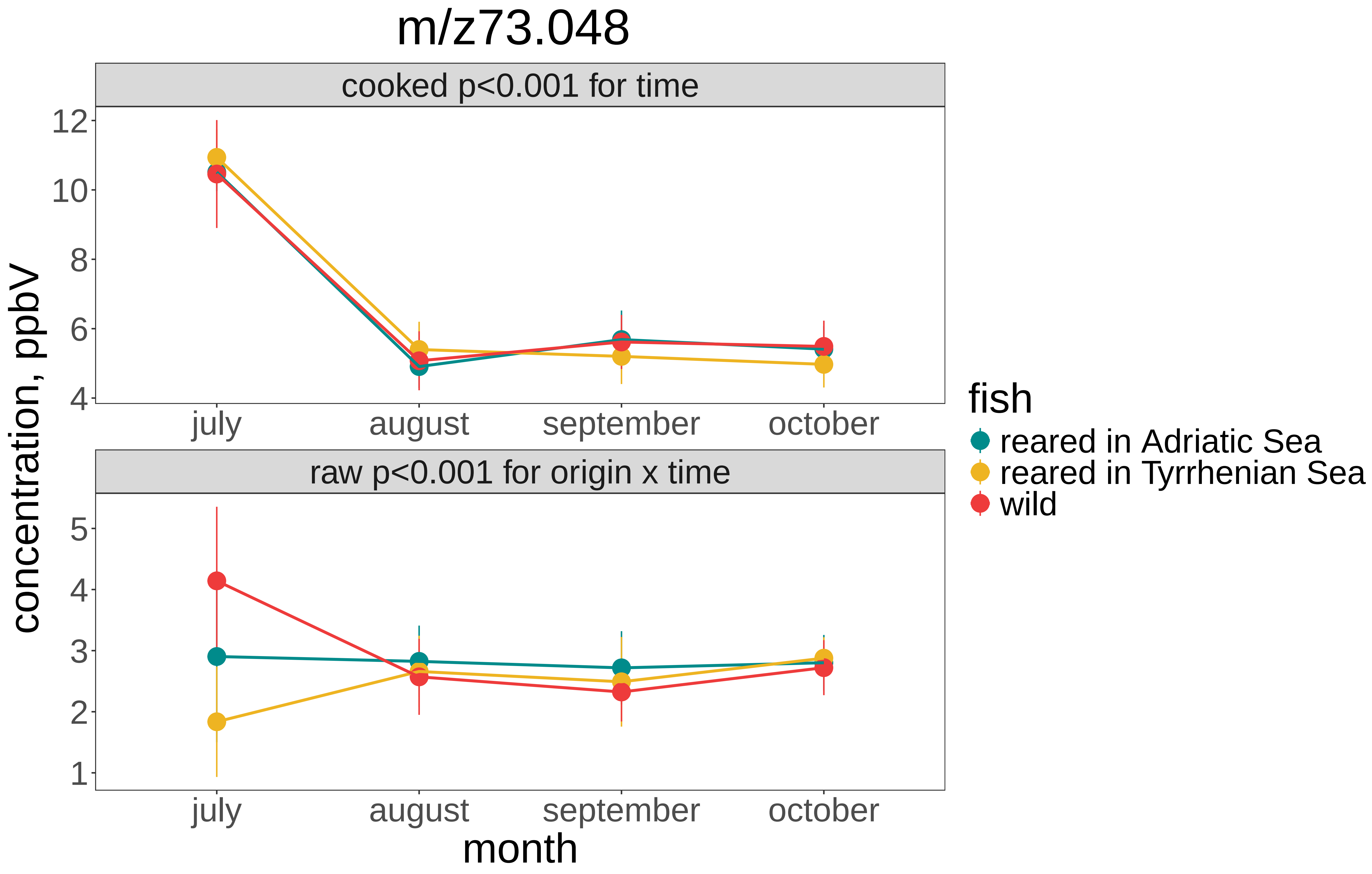
m/z73.048
cooked p<0.001 for time
raw p<0.001 for origin x time
concentration, ppbV
month
july
august
september
october
12
10
8
6
4
5
3
2
1
fish
reared in Adriatic Sea
reared in Tyrrhenian Sea
wild

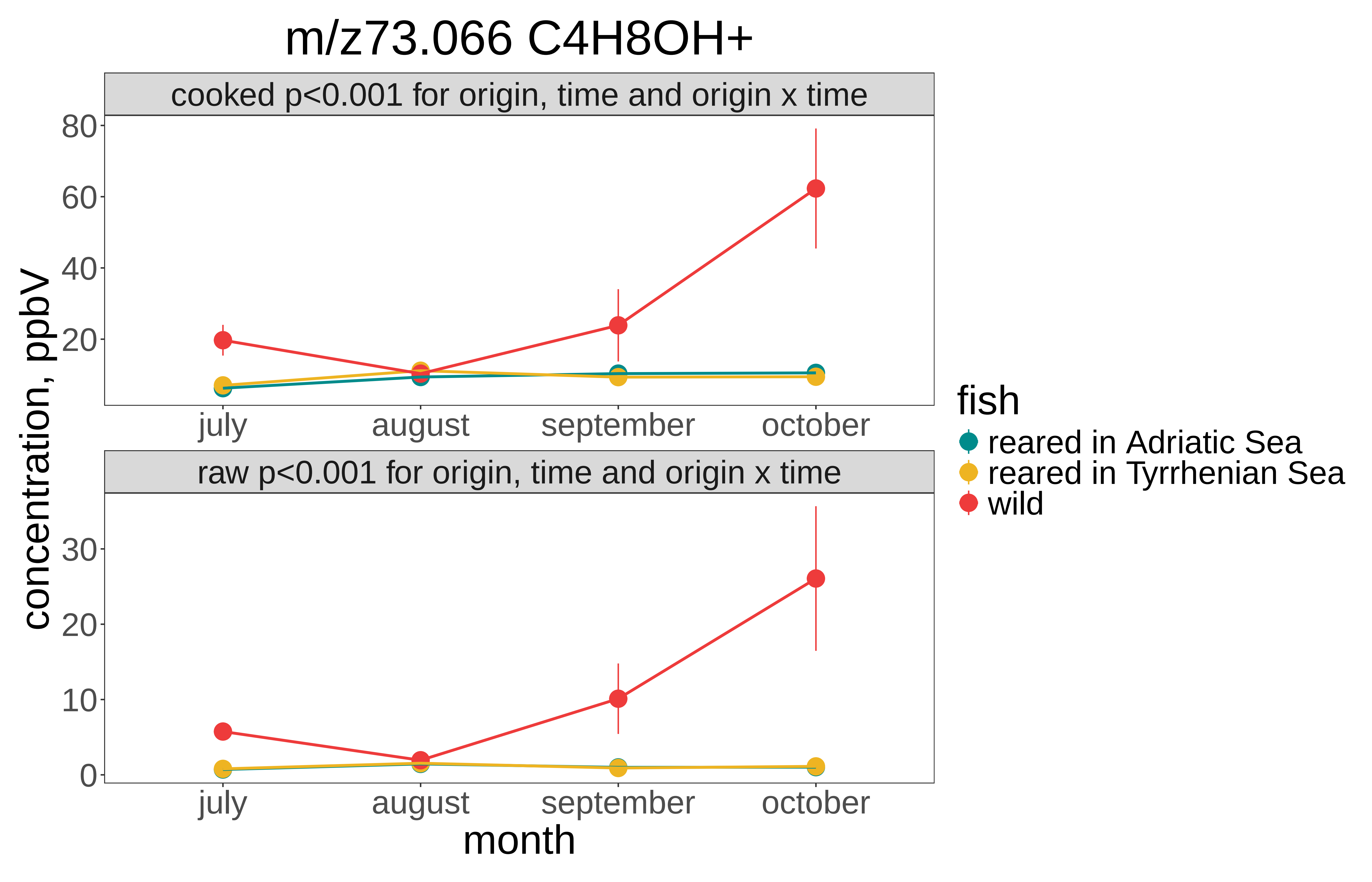
m/z73.066 C4H8OH+
cooked p<0.001 for origin, time and origin x time
raw p<0.001 for origin, time and origin x time
concentration, ppbV
80
60
40
20
30
20
10
0
july
august
september
october
month
fish
reared in Adriatic Sea
reared in Tyrrhenian Sea
wild

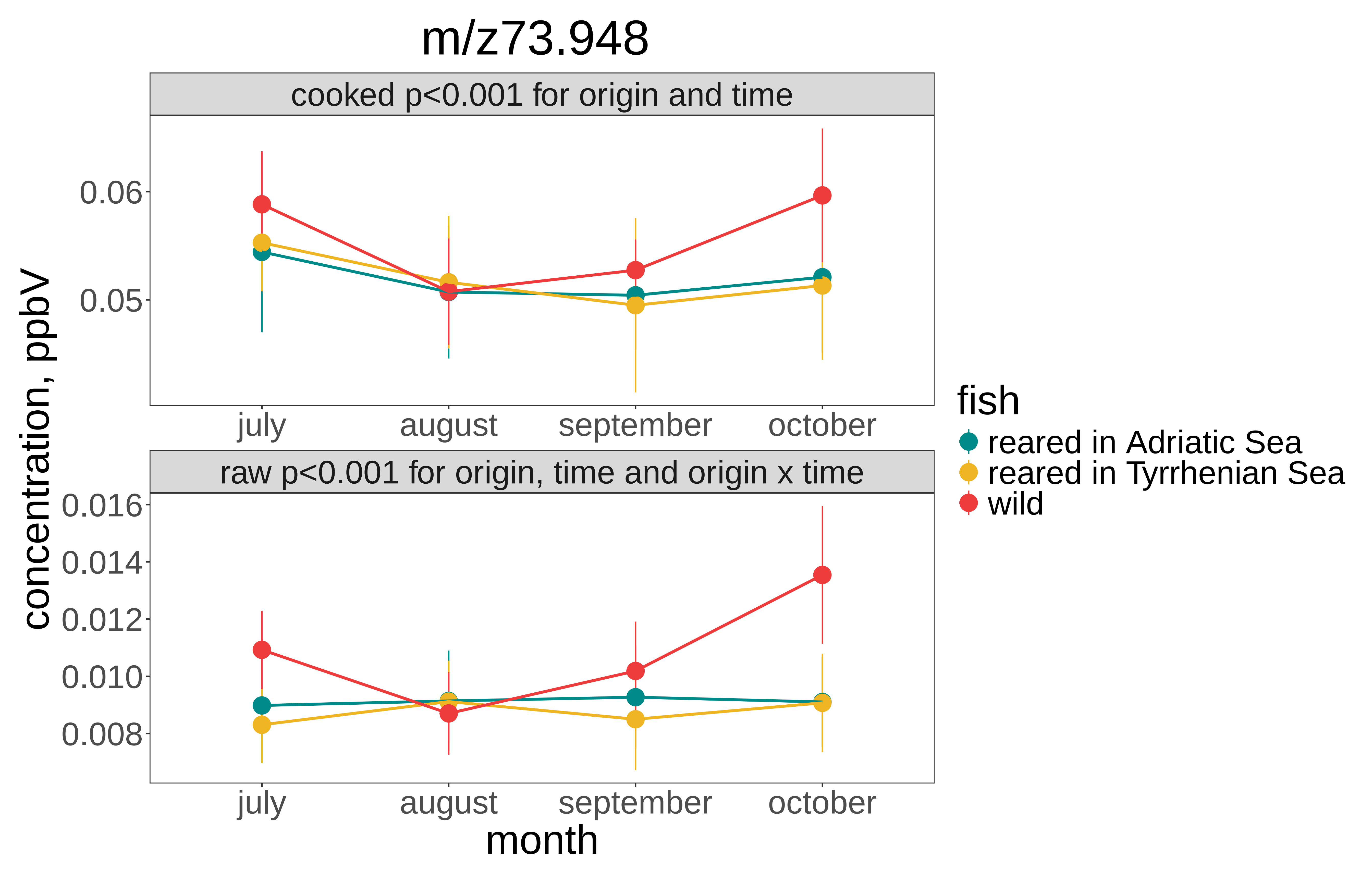
m/z73.948
cooked p<0.001 for origin and time
raw p<0.001 for origin, time and origin x time
concentration, ppbV
0.06
0.05
0.016
0.014
0.012
0.010
0.008
july
august
september
october
month
fish
reared in Adriatic Sea
reared in Tyrrhenian Sea
wild

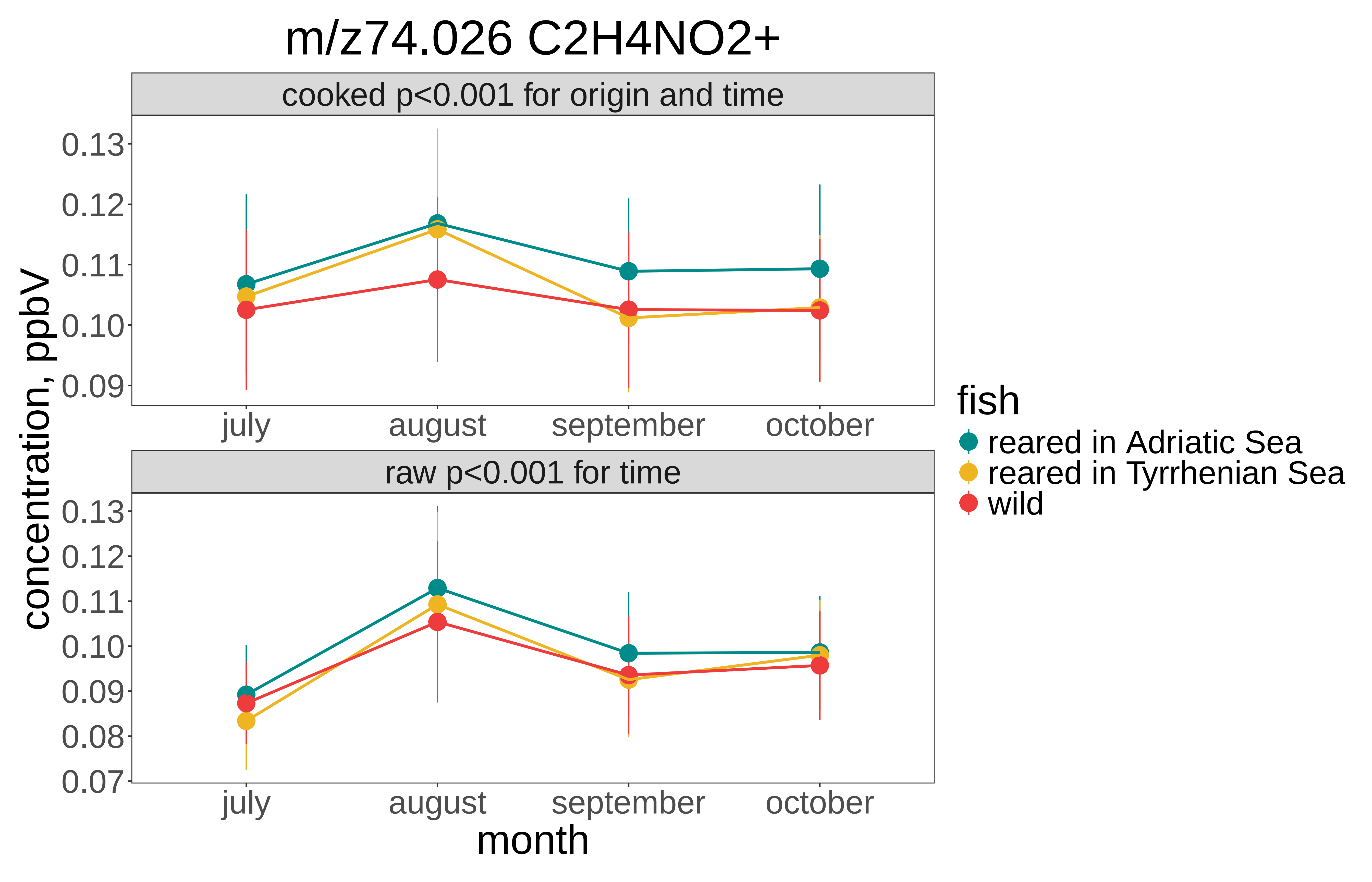
m/z74.026 C2H4NO2+
cooked p<0.001 for origin and time
raw p<0.001 for time
concentration, ppbV
0.13
0.12
0.11
0.10
0.09
0.08
0.07
july
august
september
october
month
fish
reared in Adriatic Sea
reared in Tyrrhenian Sea
wild

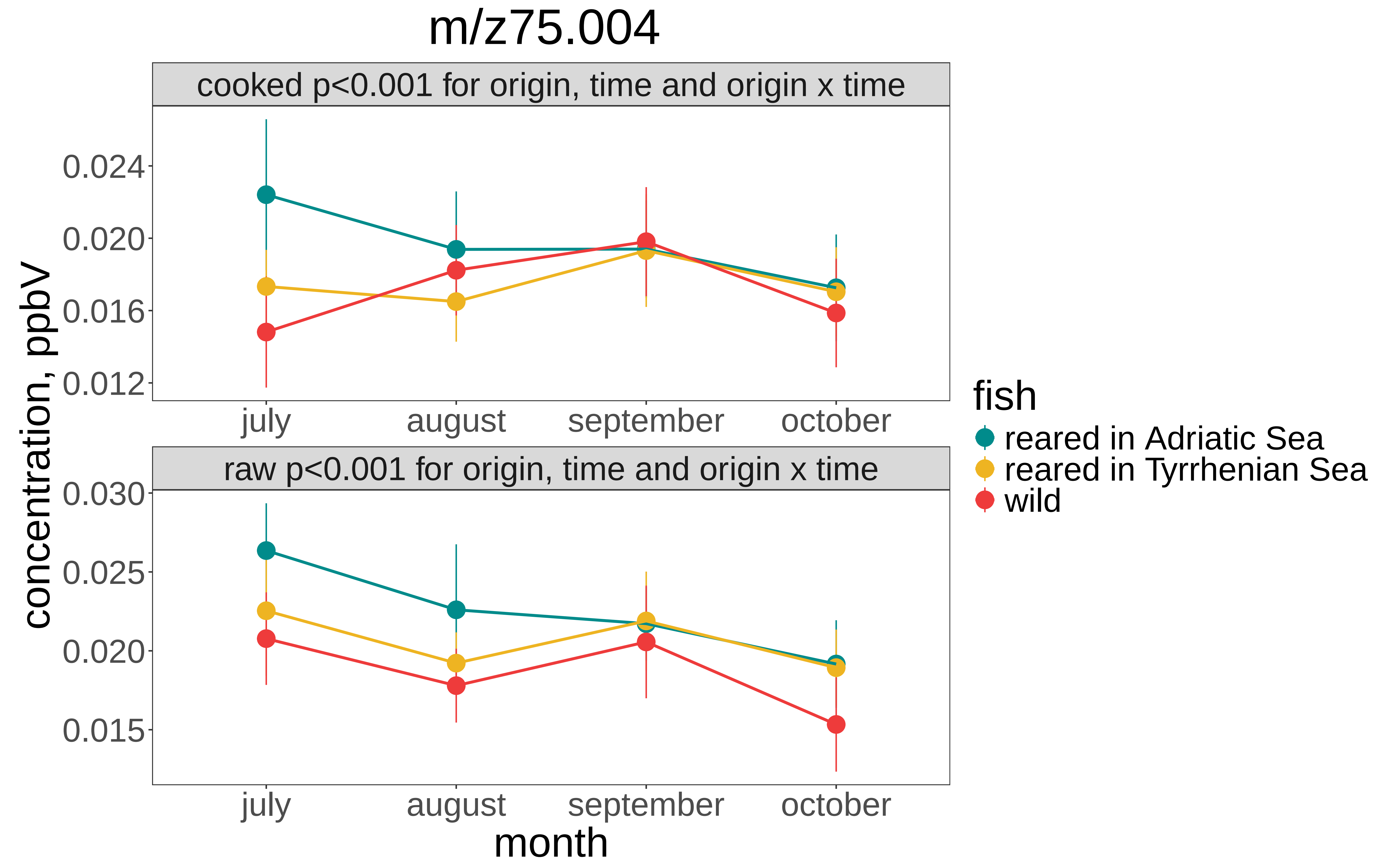

m/z75.004
cooked p<0.001 for origin, time and origin x time
raw p<0.001 for origin, time and origin x time
concentration, ppbV
0.024
0.020
0.016
0.012
0.030
0.025
0.020
0.015
july
august
september
october
month
fish
reared in Adriatic Sea
reared in Tyrrhenian Sea
wild

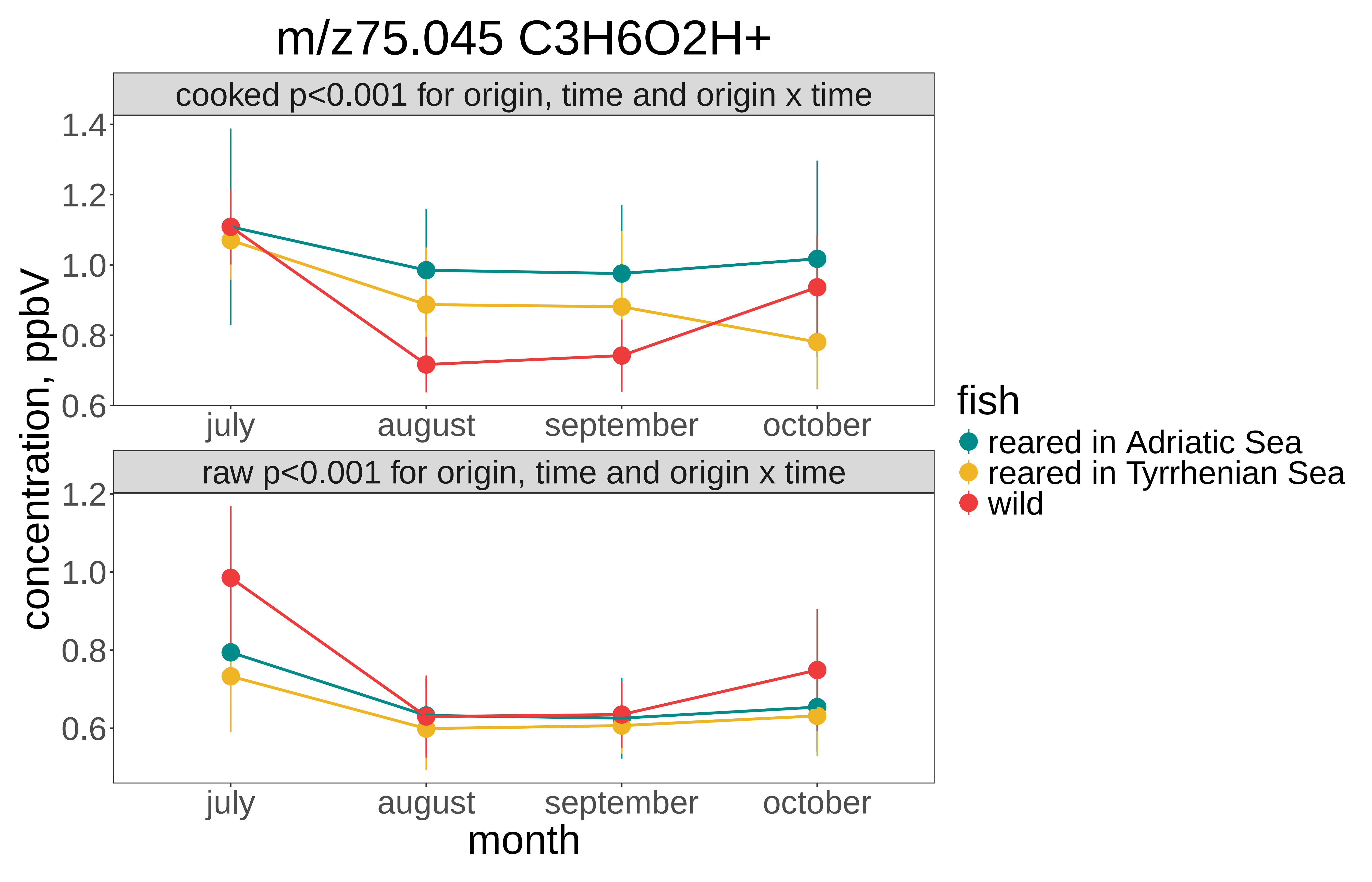
m/z75.045 C3H6O2H+
cooked p<0.001 for origin, time and origin x time
raw p<0.001 for origin, time and origin x time
concentration, ppbV
1.4
1.2
1.0
0.8
0.6
july
august
september
october
month
fish
reared in Adriatic Sea
reared in Tyrrhenian Sea
wild

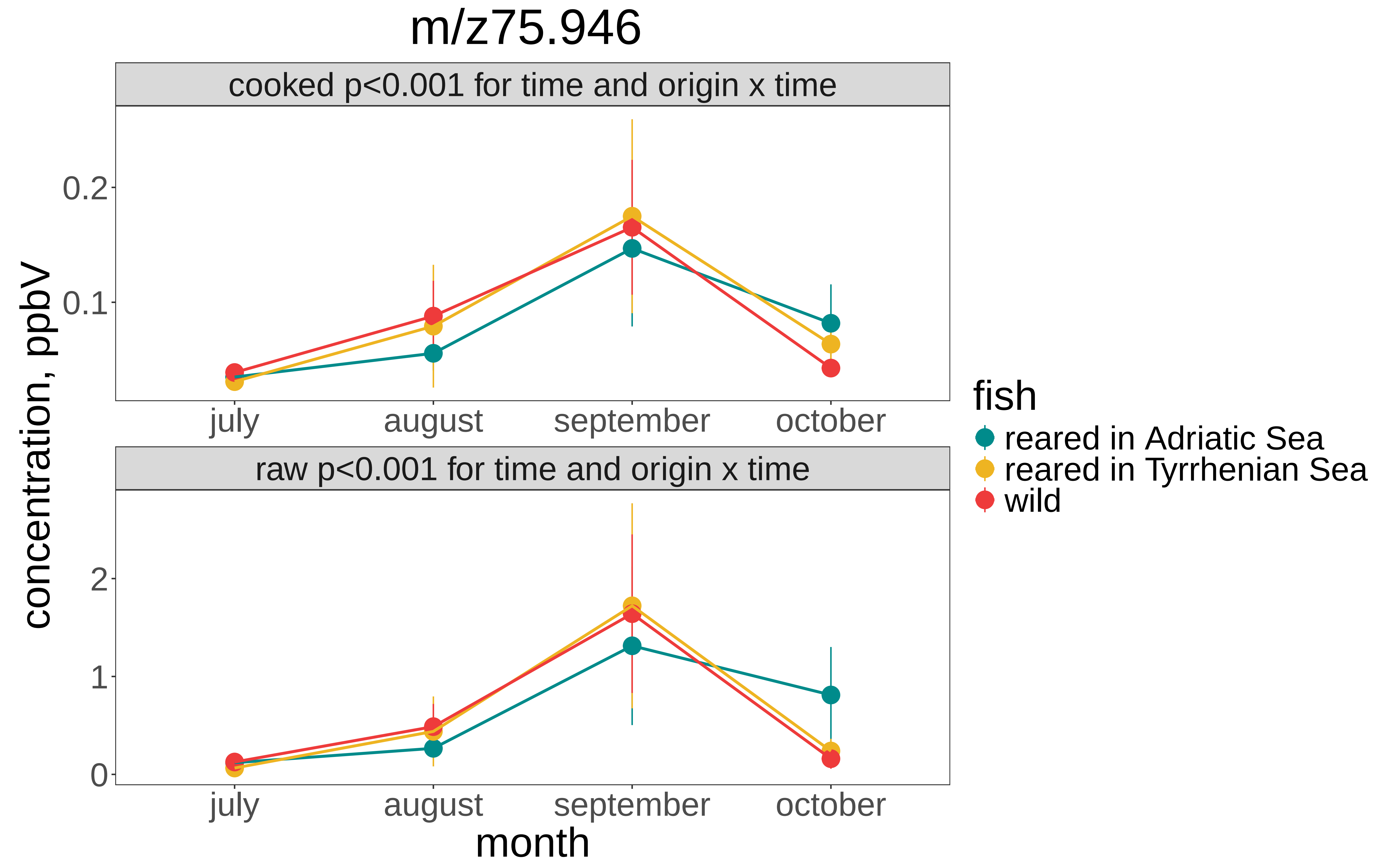
m/z75.946
cooked p<0.001 for time and origin x time
raw p<0.001 for time and origin x time
concentration, ppbV
0.2
0.1
2
1
0
july
august
september
october
month
fish
reared in Adriatic Sea
reared in Tyrrhenian Sea
wild

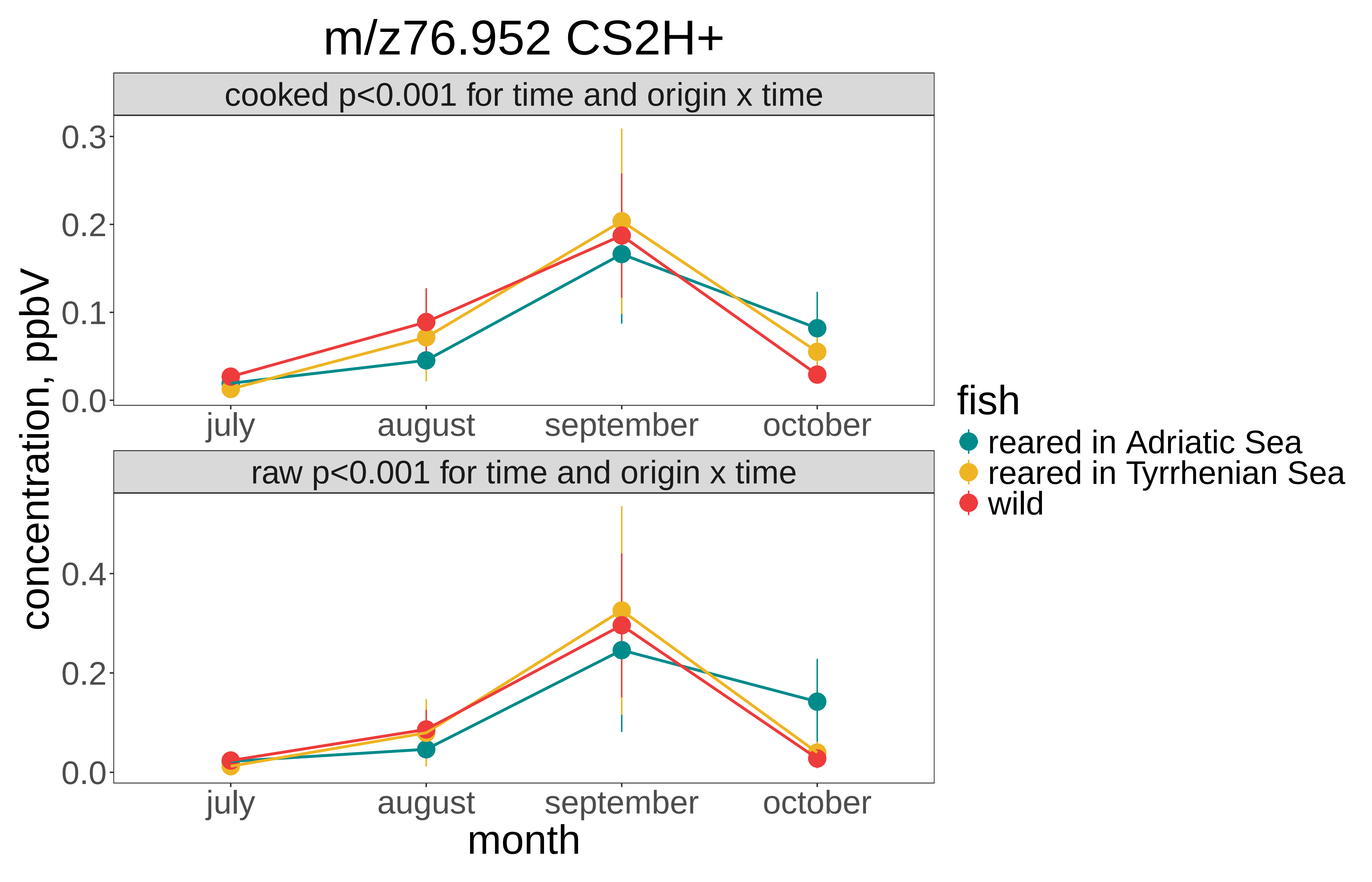

m/z76.952 CS2H+
cooked p<0.001 for time and origin x time
raw p<0.001 for time and origin x time
concentration, ppbV
0.0
0.1
0.2
0.3
0.4
july
august
september
october
month
fish
reared in Adriatic Sea
reared in Tyrrhenian Sea
wild

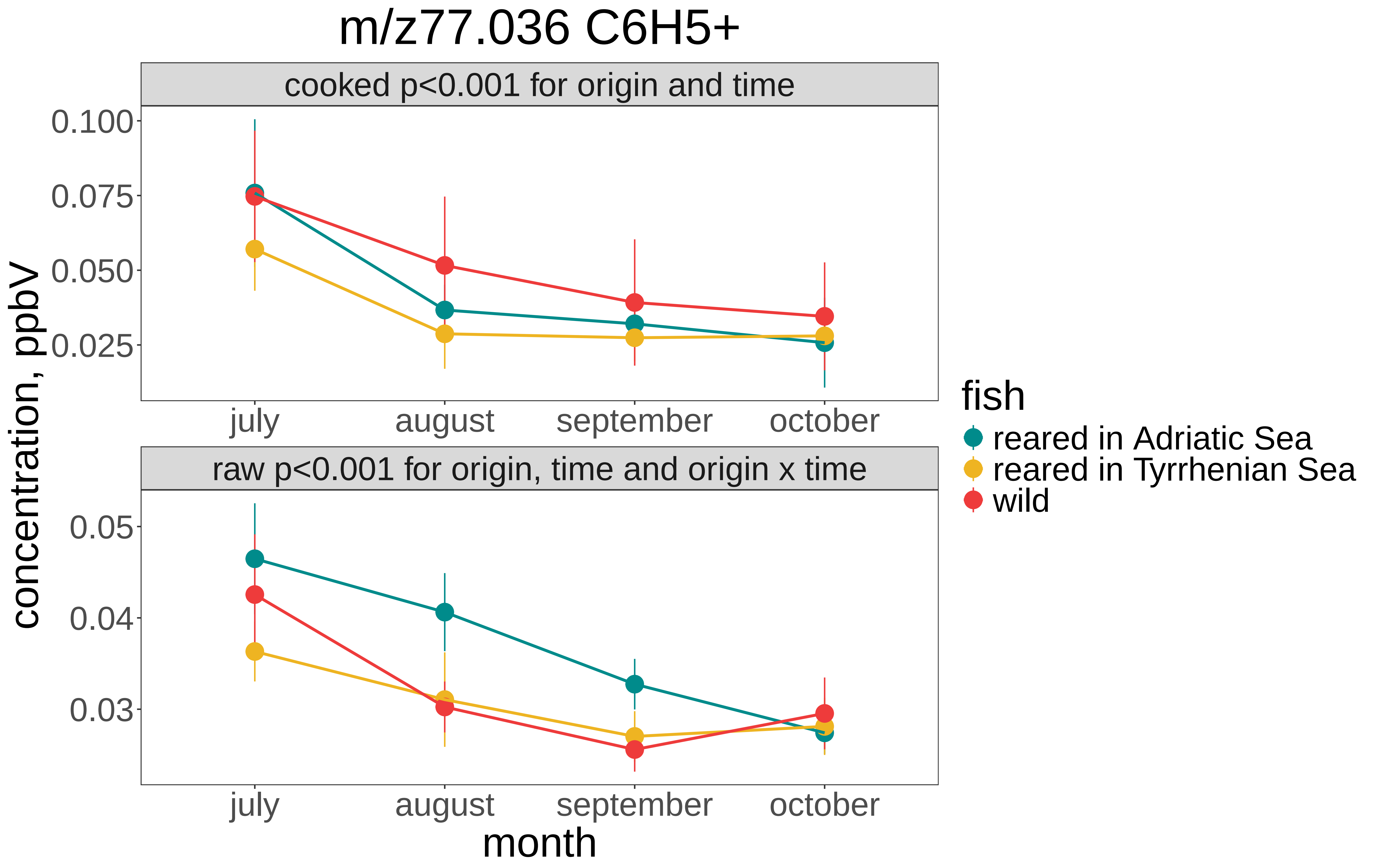
m/z77.036 C6H5+
cooked p<0.001 for origin and time
raw p<0.001 for origin, time and origin x time
concentration, ppbV
0.100
0.075
0.050
0.025
0.05
0.04
0.03
july
august
september
october
month
fish
reared in Adriatic Sea
reared in Tyrrhenian Sea
wild

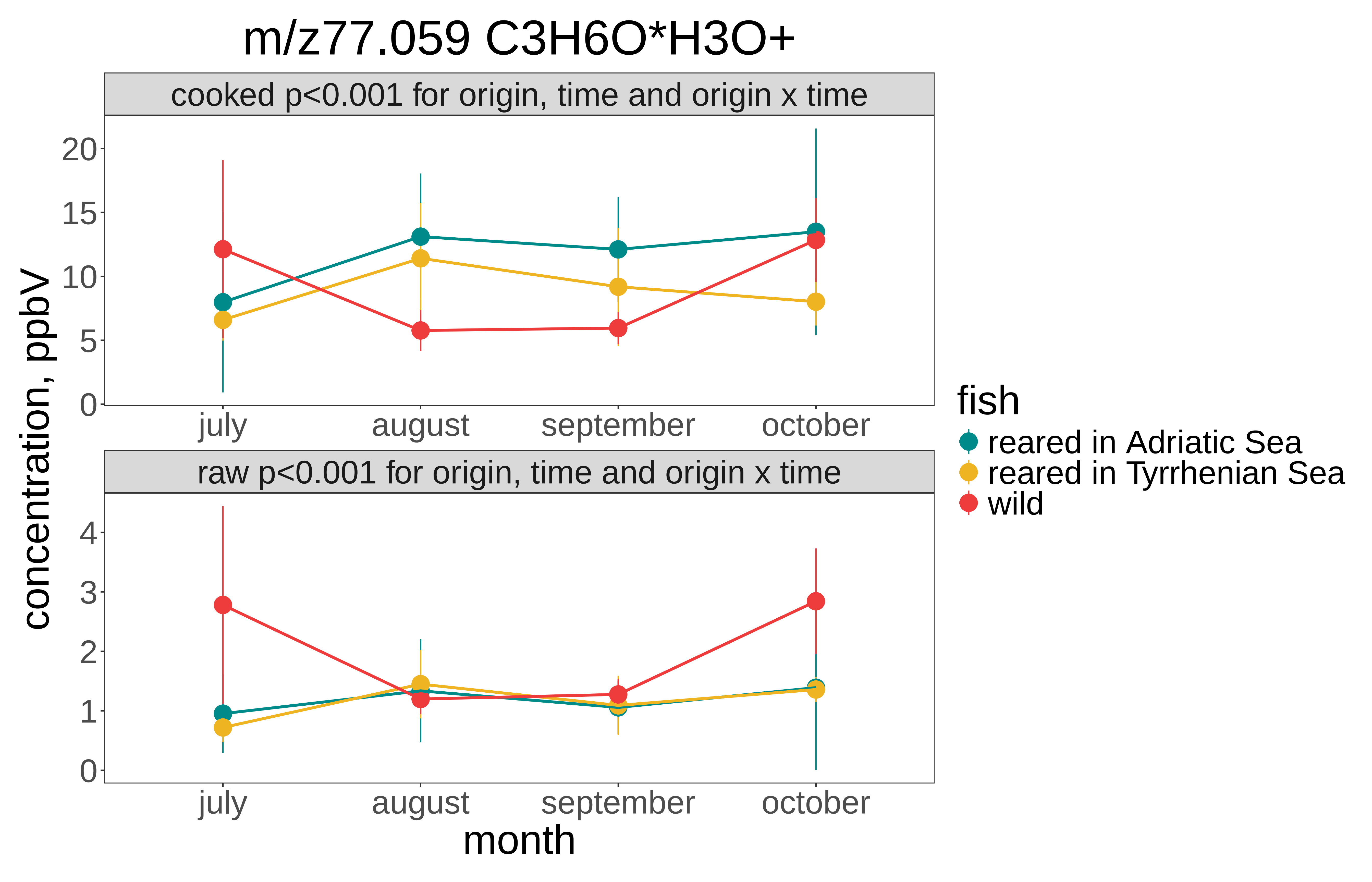
m/z77.059 C3H6O*H3O+
cooked p<0.001 for origin, time and origin x time
raw p<0.001 for origin, time and origin x time
concentration, ppbV
month
july
august
september
october
20
15
10
5
0
4
3
2
1
fish
reared in Adriatic Sea
reared in Tyrrhenian Sea
wild

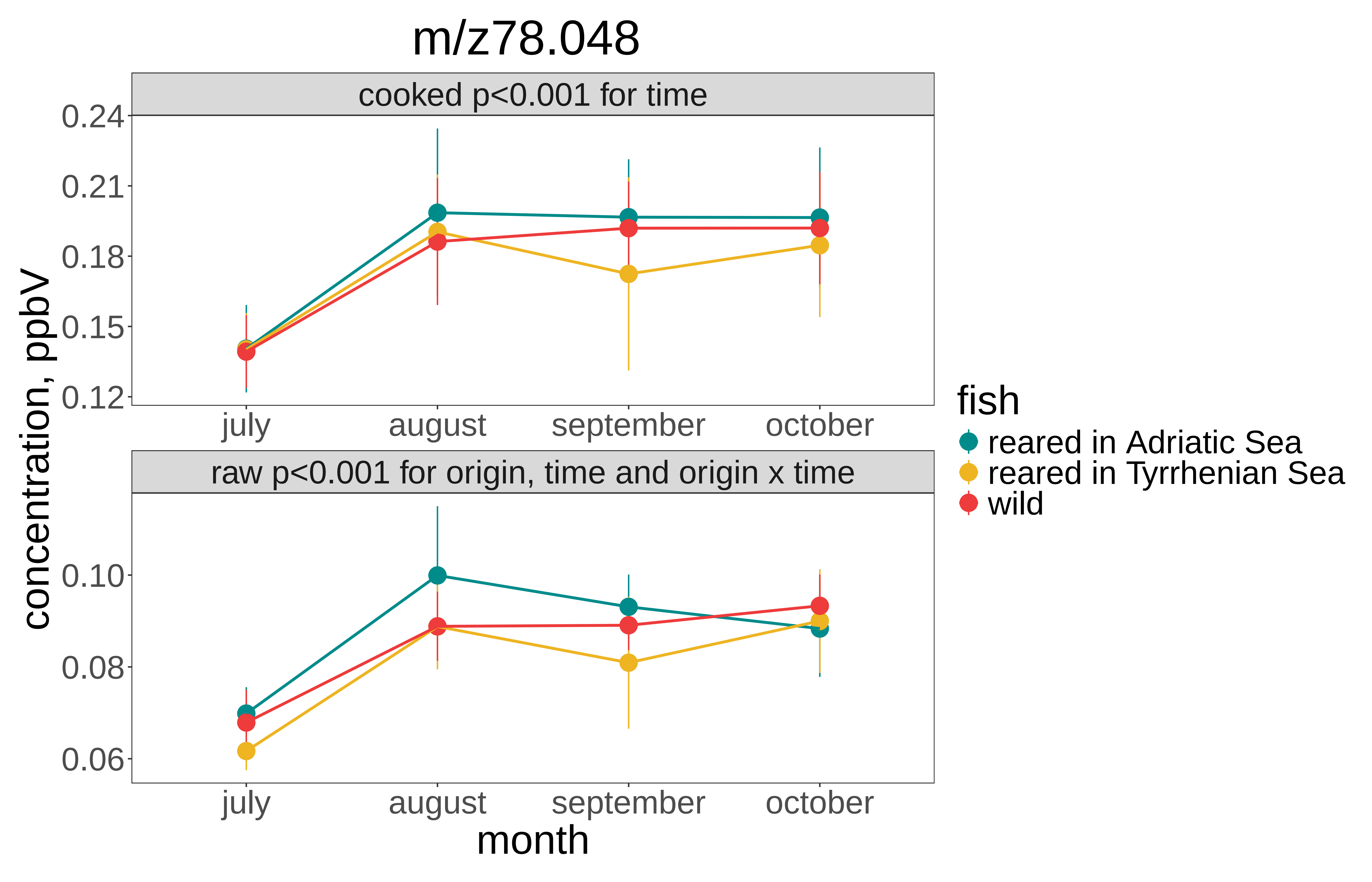
m/z78.048
cooked p<0.001 for time
raw p<0.001 for origin, time and origin x time
concentration, ppbV
0.24
0.21
0.18
0.15
0.12
0.10
0.08
0.06
july
august
september
october
month
fish
reared in Adriatic Sea
reared in Tyrrhenian Sea
wild

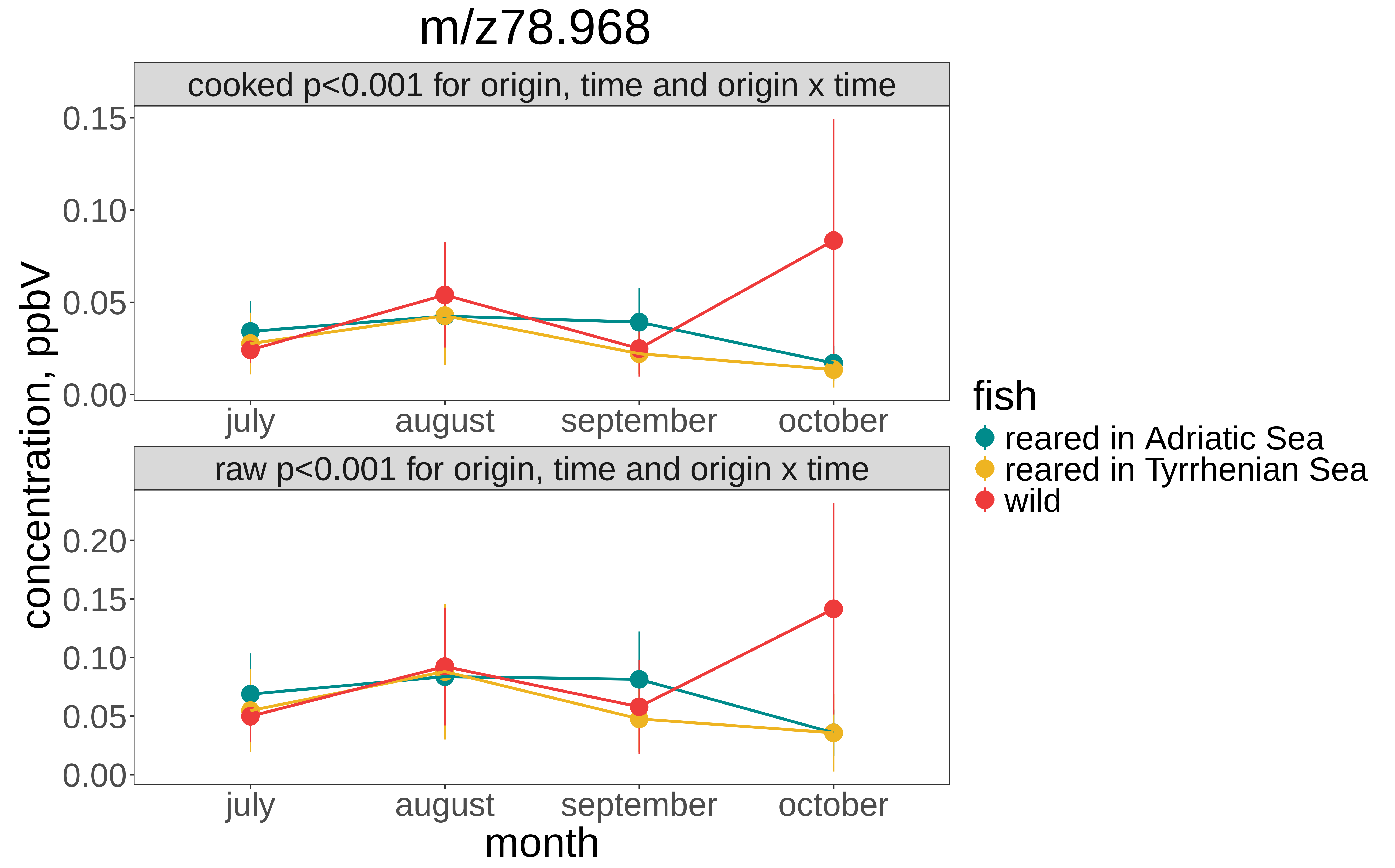
m/z78.968
cooked p<0.001 for origin, time and origin x time
raw p<0.001 for origin, time and origin x time
concentration, ppbV
0.15
0.10
0.05
0.00
0.20
july
august
september
october
month
fish
reared in Adriatic Sea
reared in Tyrrhenian Sea
wild

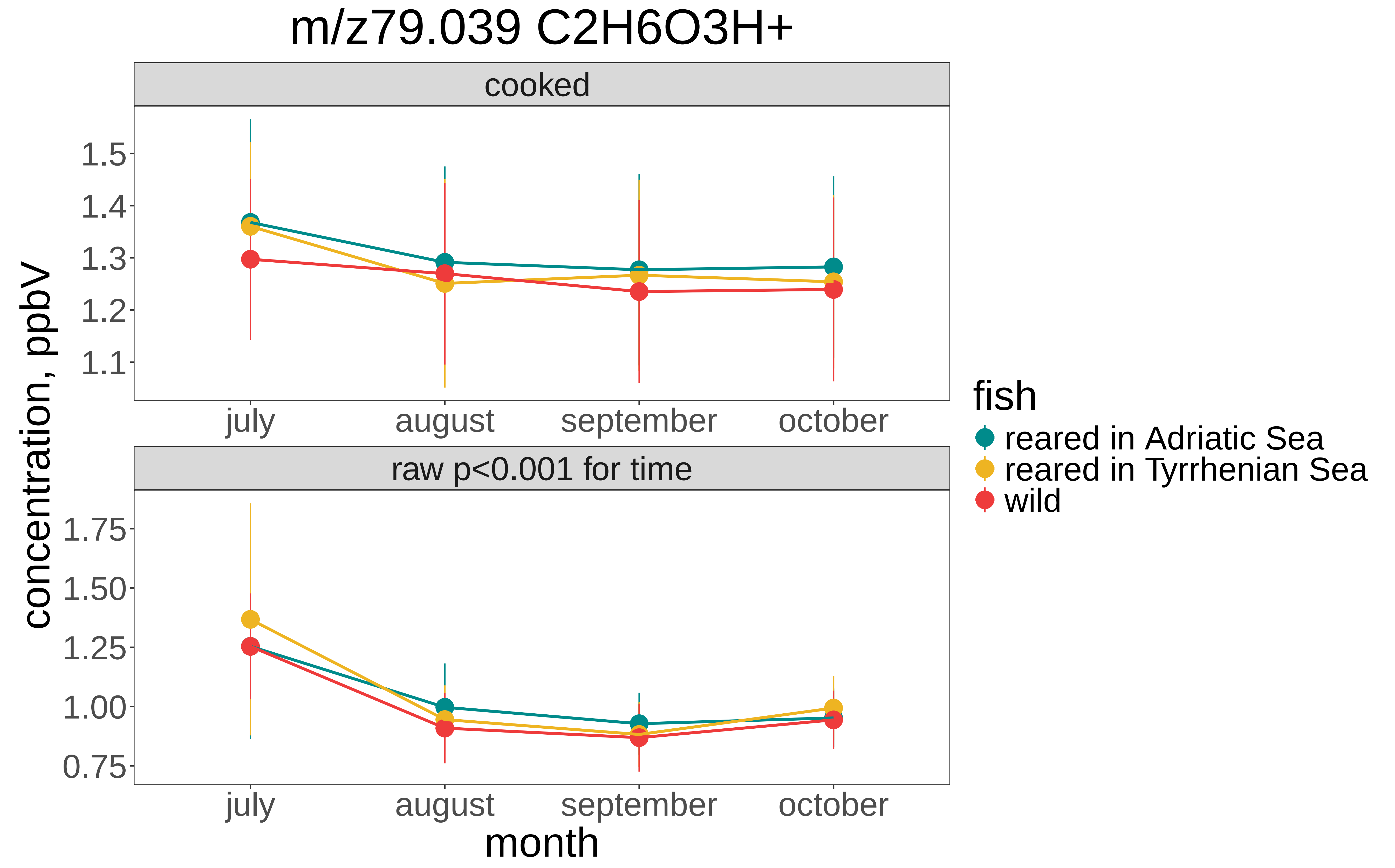

m/z79.039 C2H6O3H+
cooked
raw p<0.001 for time
concentration, ppbV
month
july
august
september
october
fish
reared in Adriatic Sea
reared in Tyrrhenian Sea
wild

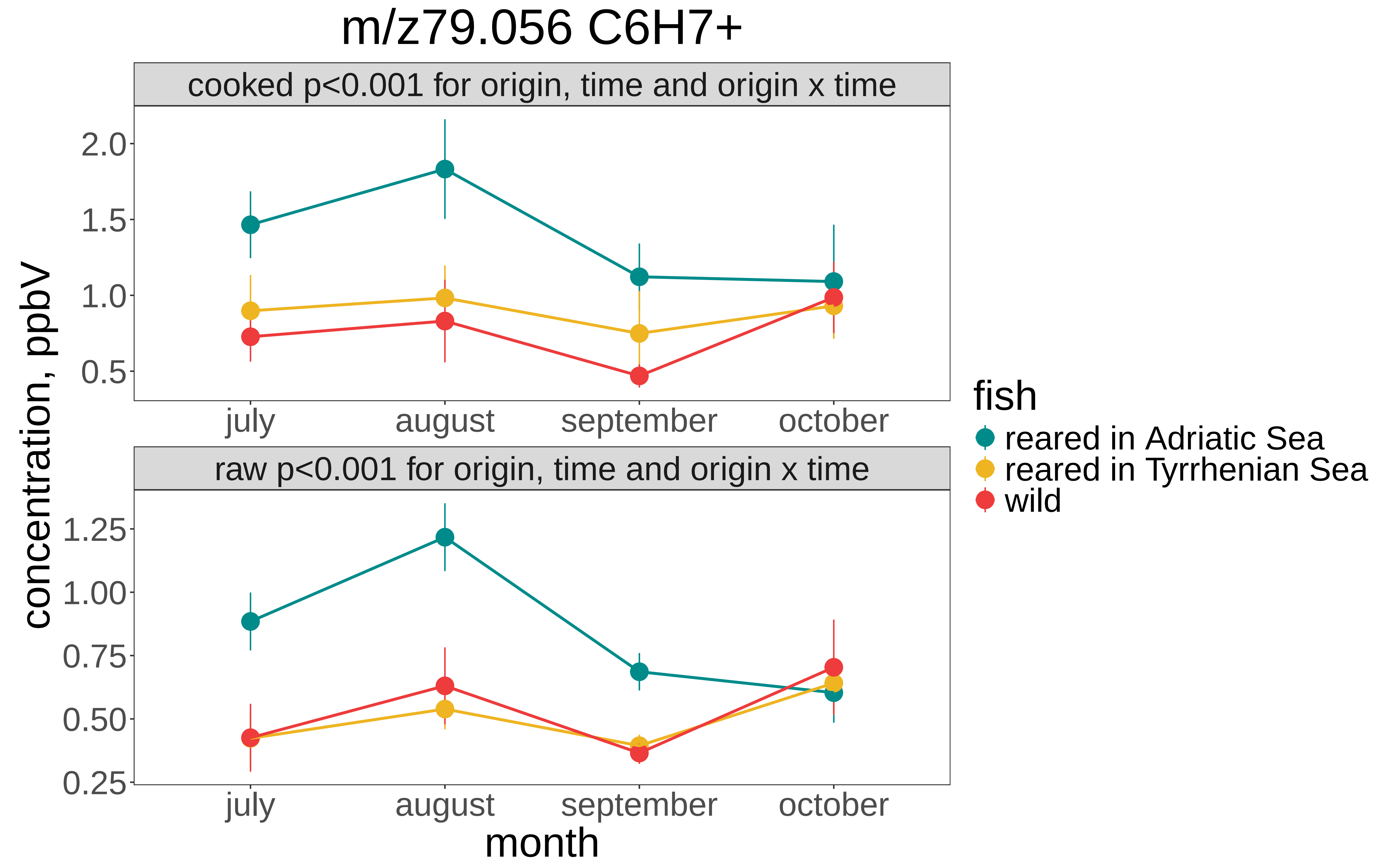

m/z79.056 C6H7+
cooked p<0.001 for origin, time and origin x time
raw p<0.001 for origin, time and origin x time
concentration, ppbV
2.0
1.5
1.0
0.5
1.25
1.00
0.75
0.50
0.25
july
august
september
october
month
fish
reared in Adriatic Sea
reared in Tyrrhenian Sea
wild

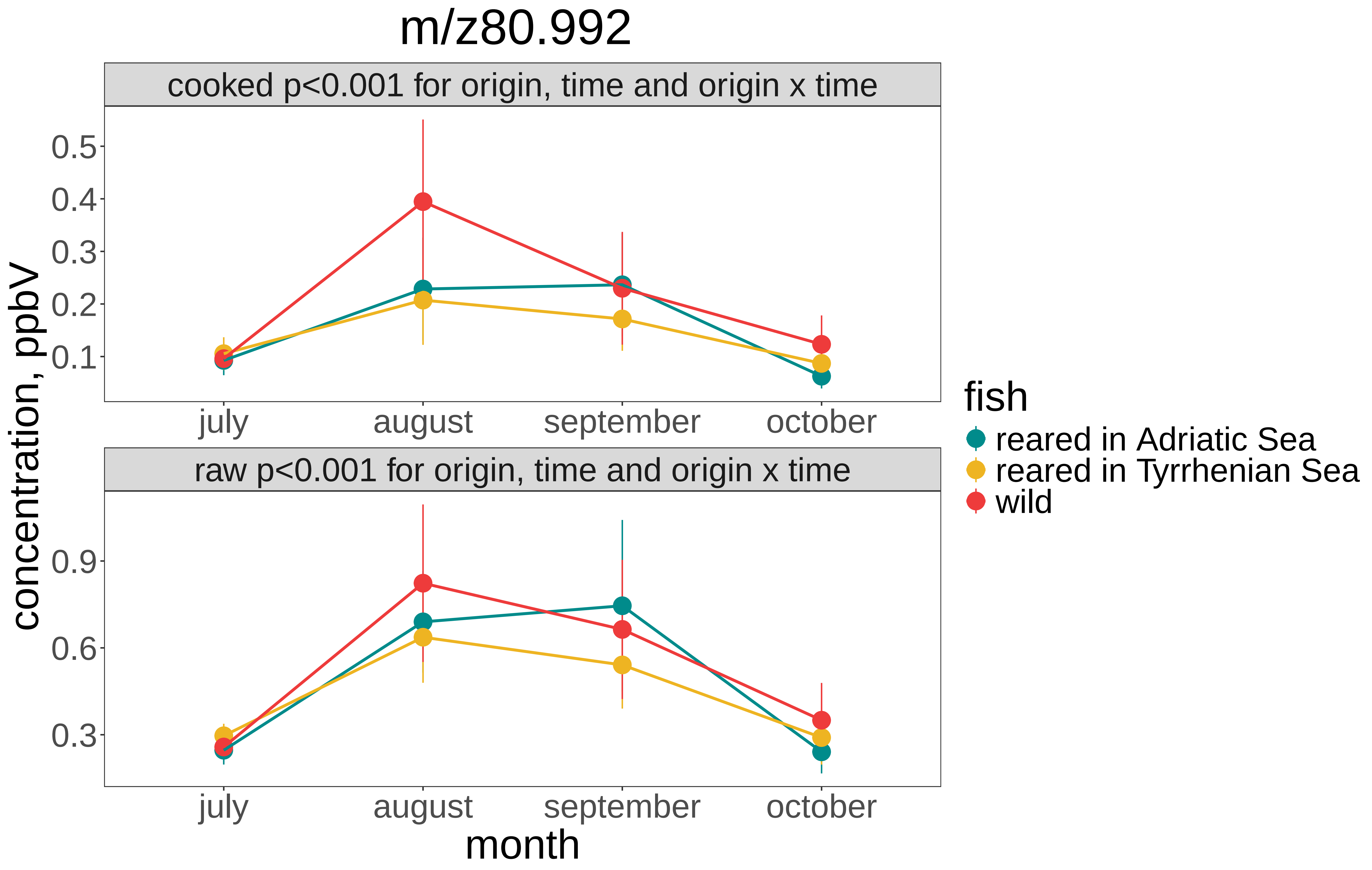
m/z80.992
cooked p<0.001 for origin, time and origin x time
raw p<0.001 for origin, time and origin x time
concentration, ppbV
0.5
0.4
0.3
0.2
0.1
0.9
0.6
0.3
july
august
september
october
month
fish
reared in Adriatic Sea
reared in Tyrrhenian Sea
wild

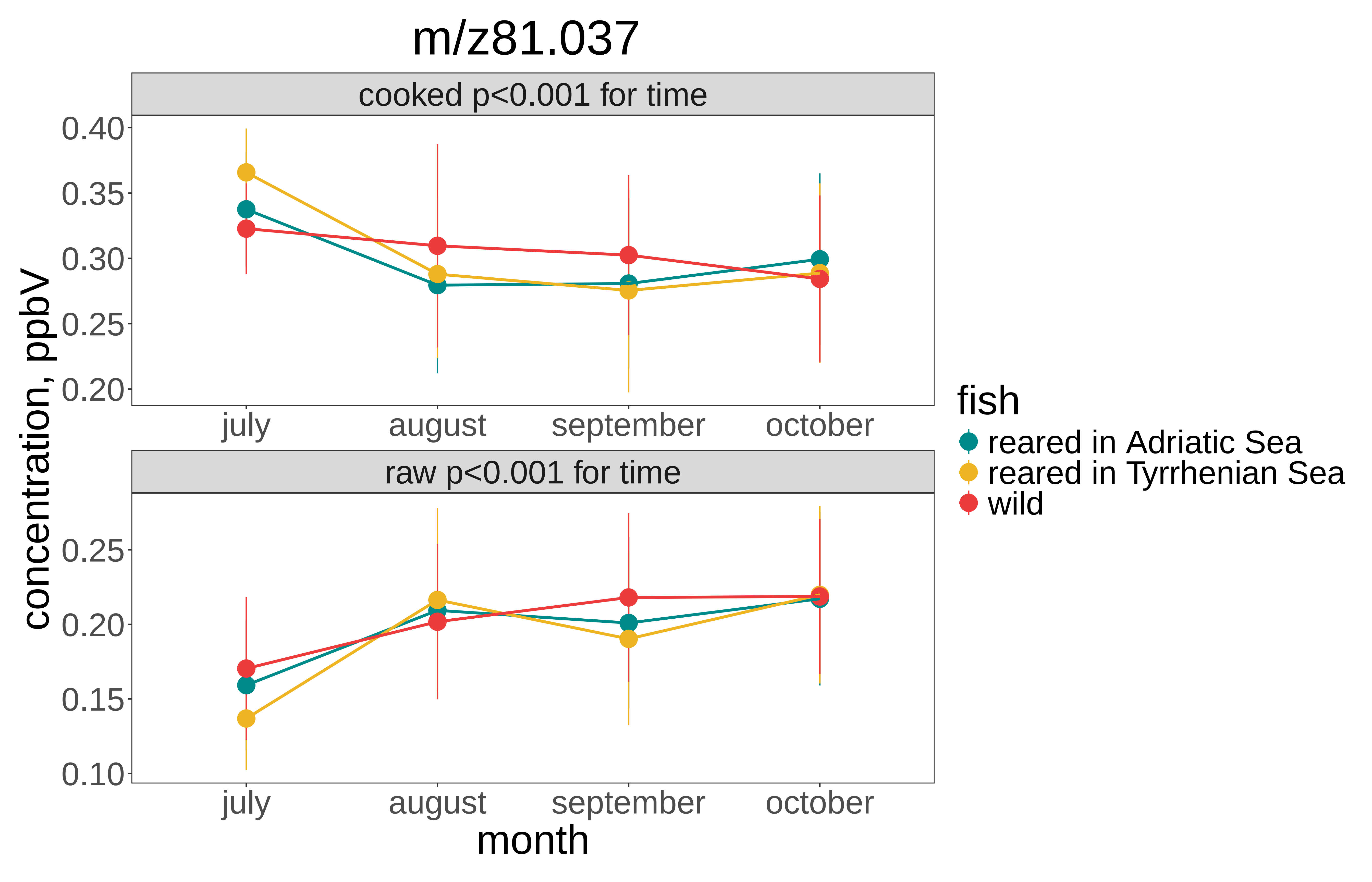

m/z81.037
cooked p<0.001 for time
raw p<0.001 for time
concentration, ppbV
0.40
0.35
0.30
0.25
0.20
0.15
0.10
july
august
september
october
month
fish
reared in Adriatic Sea
reared in Tyrrhenian Sea
wild

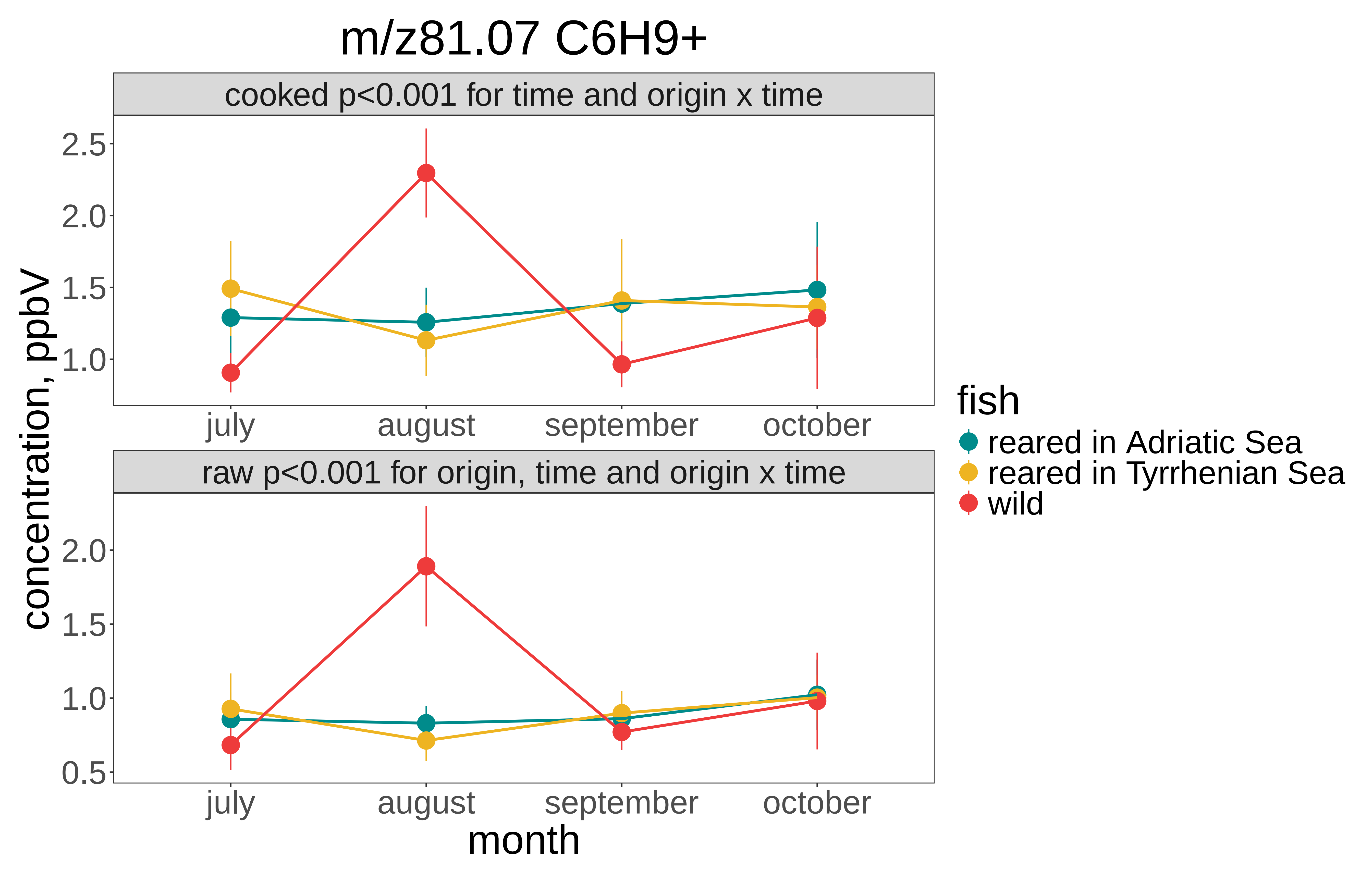
m/z81.07 C6H9+
cooked p<0.001 for time and origin x time
raw p<0.001 for origin, time and origin x time
concentration, ppbV
2.5
2.0
1.5
1.0
0.5
july
august
september
october
month
fish
reared in Adriatic Sea
reared in Tyrrhenian Sea
wild

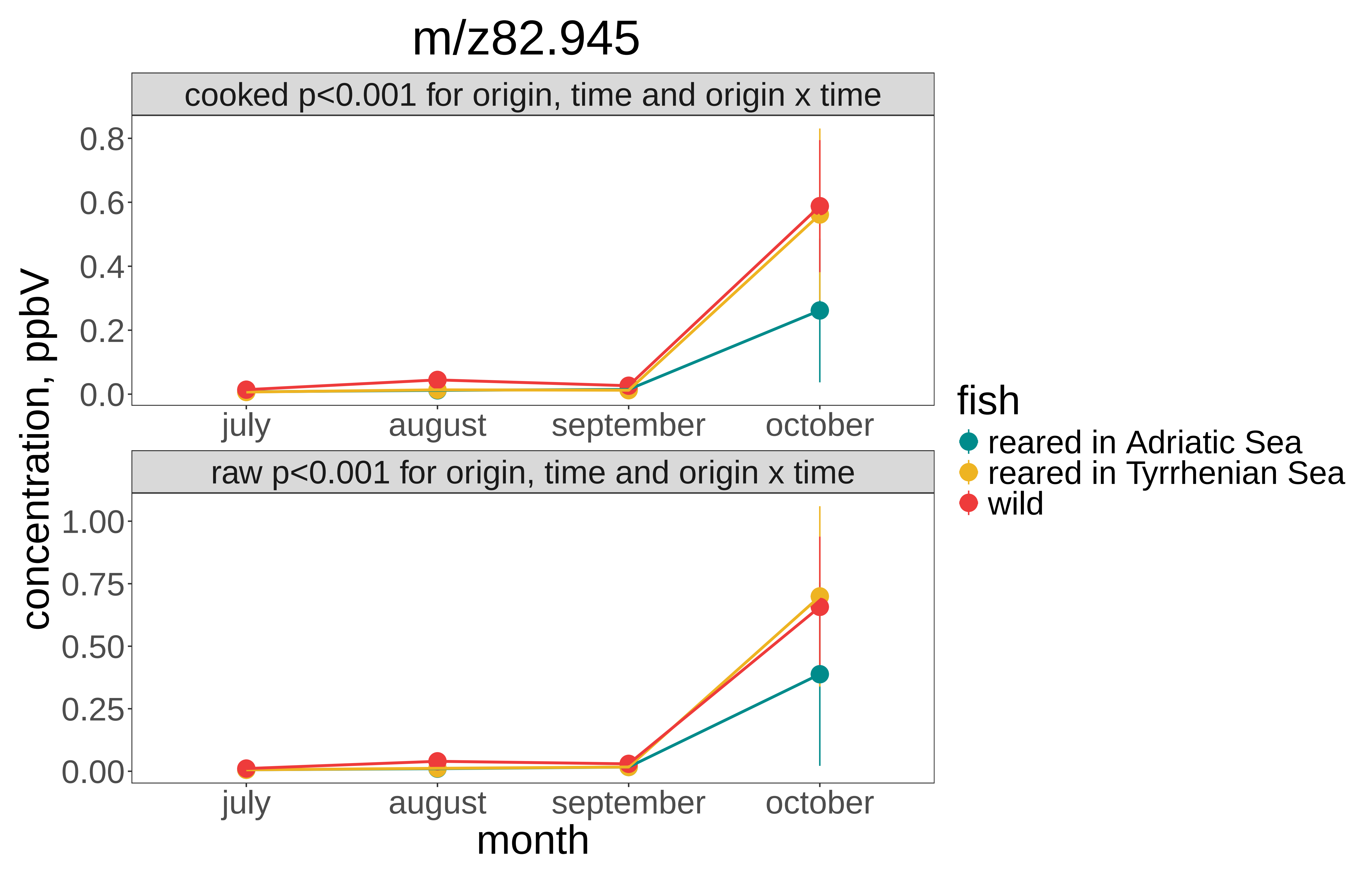

m/z82.945
cooked p<0.001 for origin, time and origin x time
raw p<0.001 for origin, time and origin x time
concentration, ppbV
0.8
0.6
0.4
0.2
0.0
1.00
0.75
0.50
0.25
0.00
july
august
september
october
month
fish
reared in Adriatic Sea
reared in Tyrrhenian Sea
wild

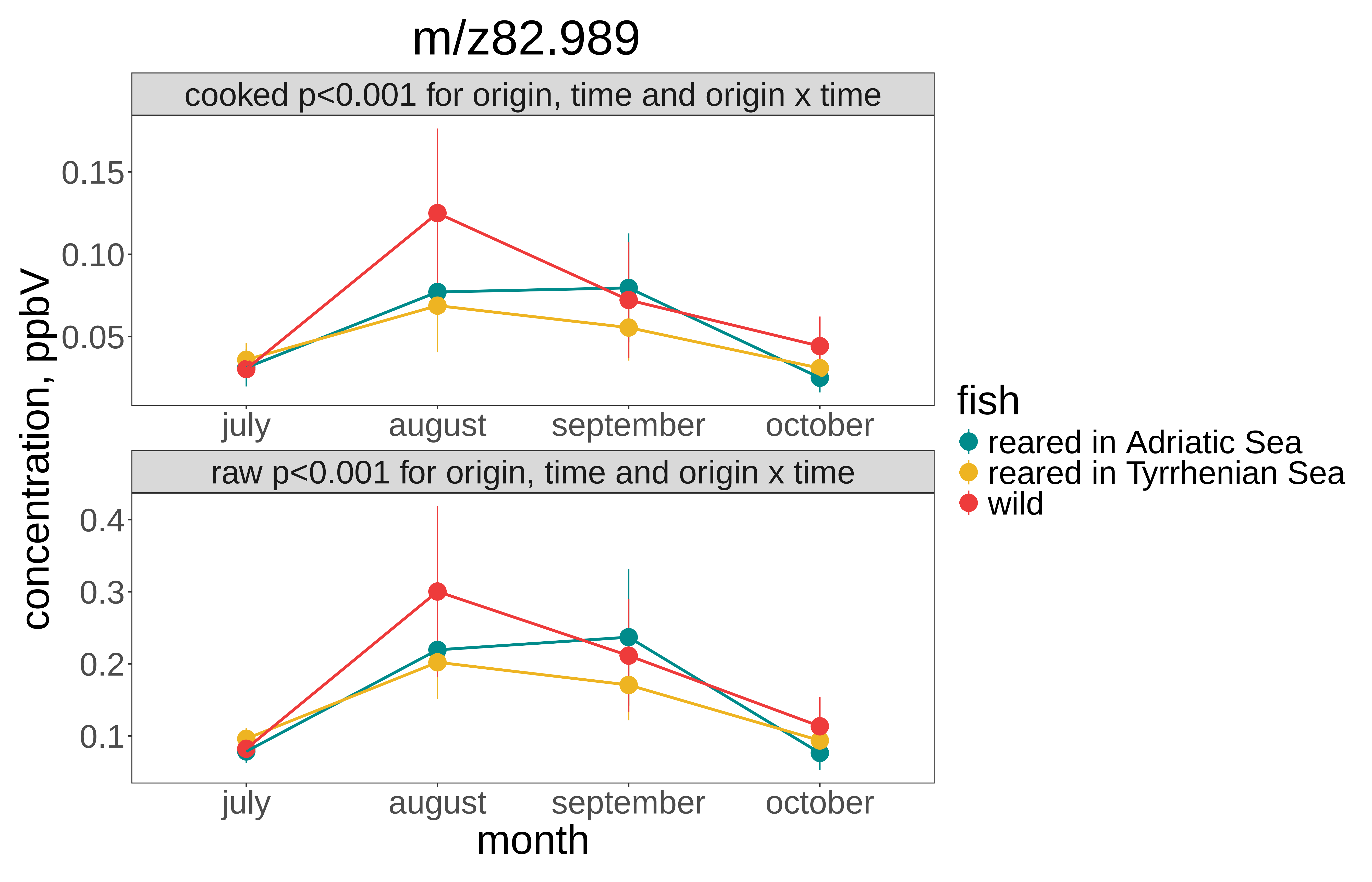
m/z82.989
cooked p<0.001 for origin, time and origin x time
raw p<0.001 for origin, time and origin x time
0.15
0.10
0.05
0.4
0.3
0.2
0.1
concentration, ppbV
july
august
september
october
month
fish
reared in Adriatic Sea
reared in Tyrrhenian Sea
wild

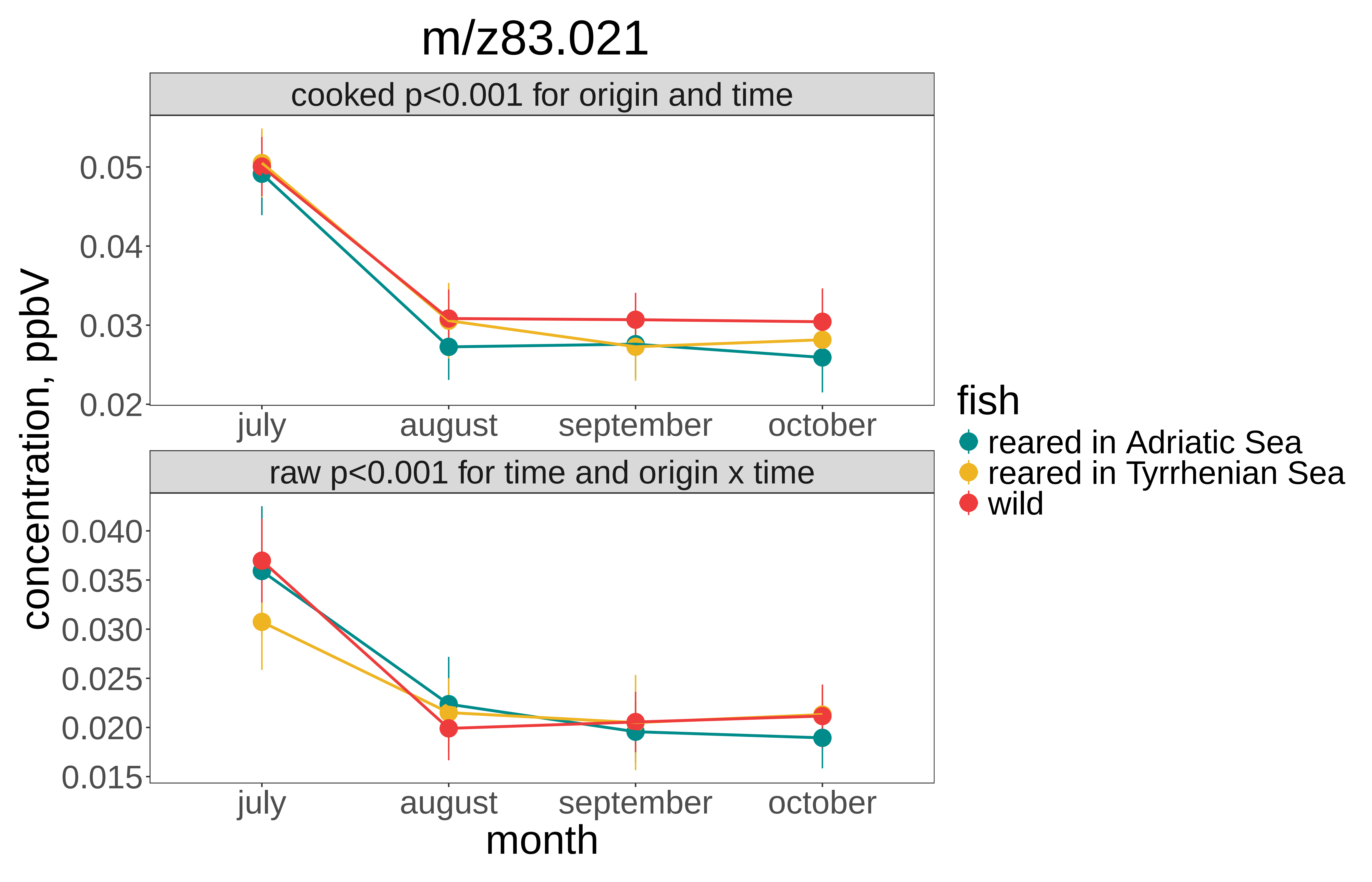

m/z83.021
cooked p<0.001 for origin and time
raw p<0.001 for time and origin x time
concentration, ppbV
0.05
0.04
0.03
0.02
0.040
0.035
0.030
0.025
0.020
0.015
july
august
september
october
month
fish
reared in Adriatic Sea
reared in Tyrrhenian Sea
wild

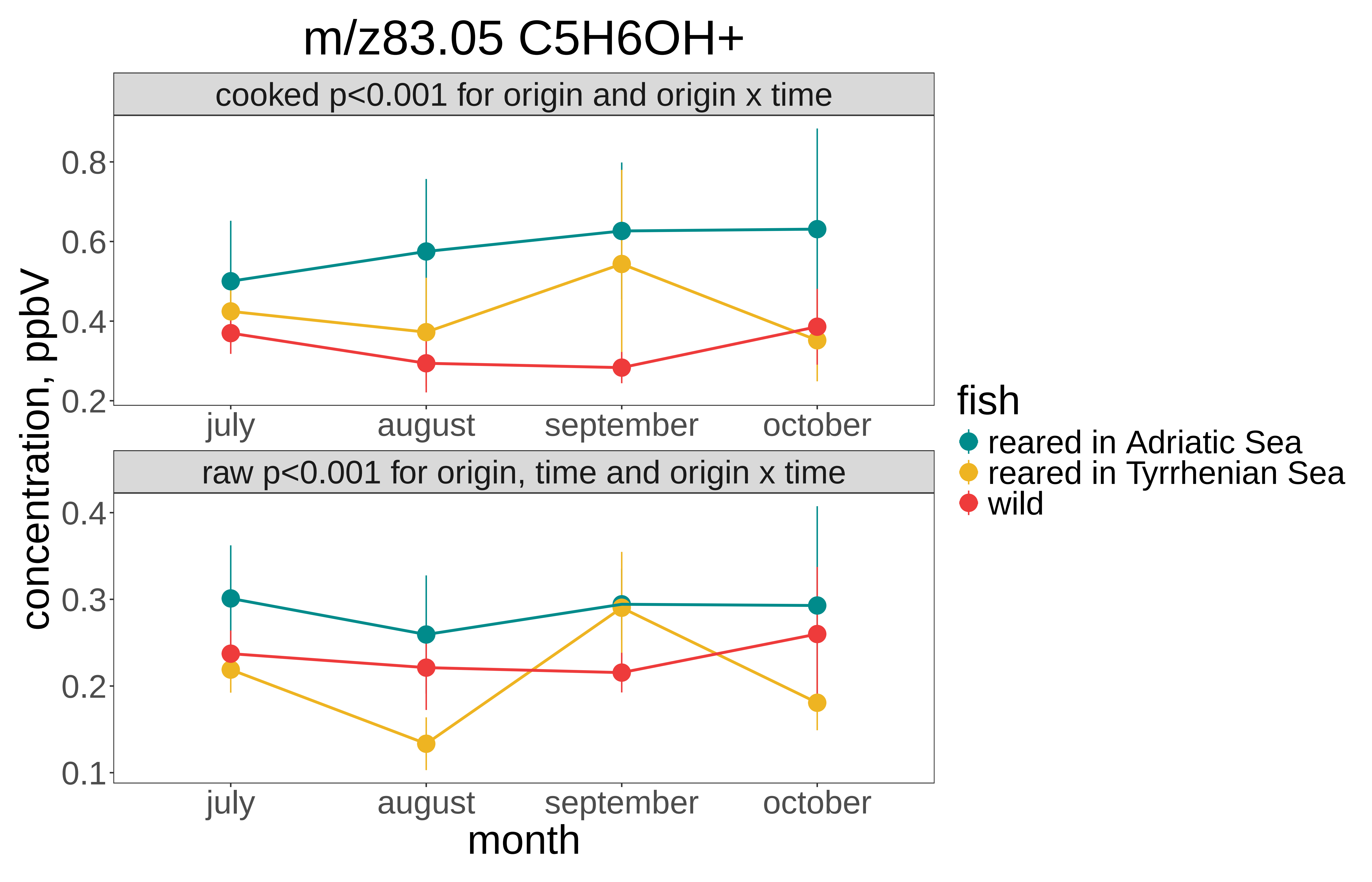
m/z83.05 C5H6OH+
cooked p<0.001 for origin and origin x time
raw p<0.001 for origin, time and origin x time
concentration, ppbV
0.8
0.6
0.4
0.2
0.4
0.3
0.2
0.1
july
august
september
october
month
fish
reared in Adriatic Sea
reared in Tyrrhenian Sea
wild

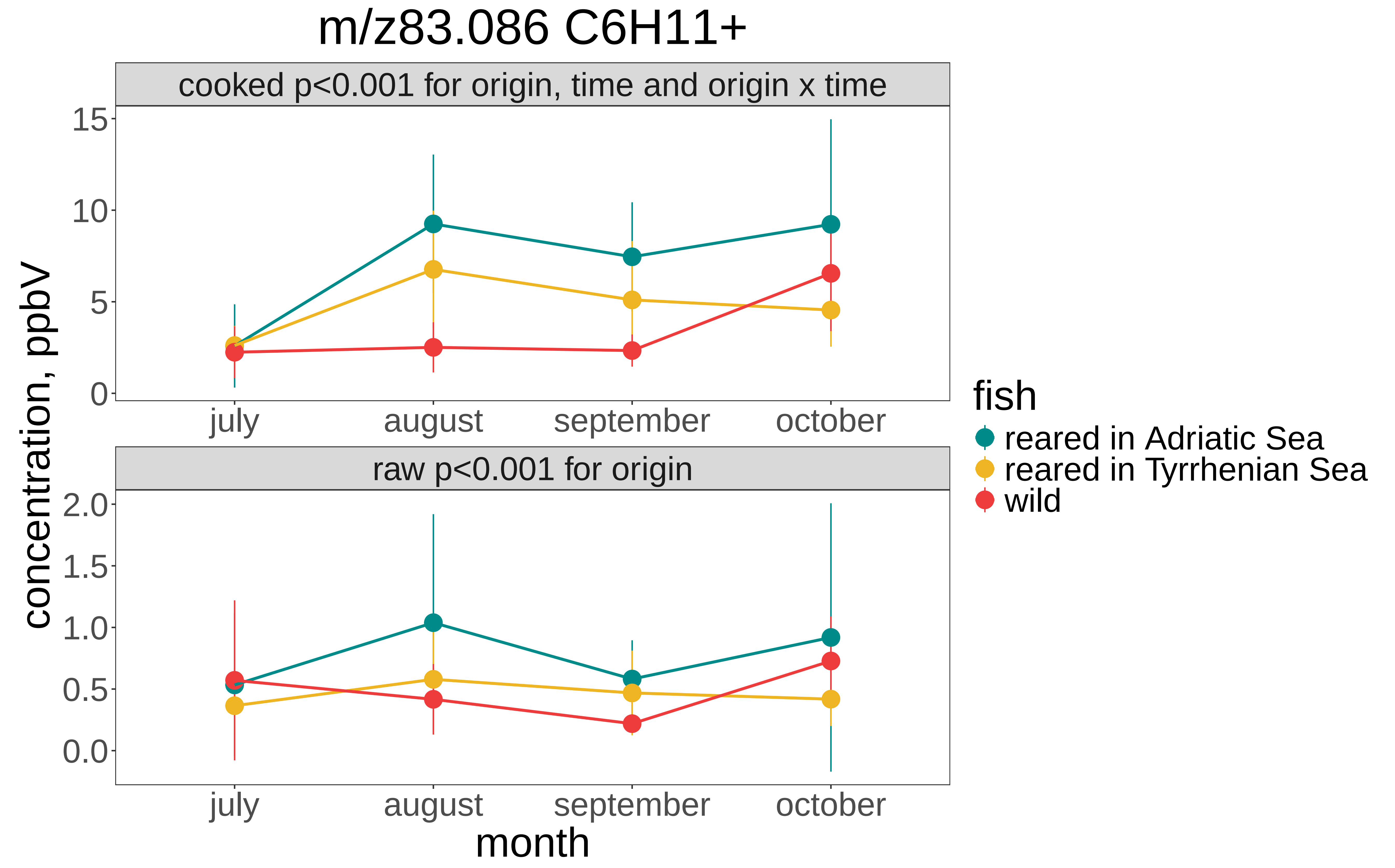

m/z83.086 C6H11+
cooked p<0.001 for origin, time and origin x time
raw p<0.001 for origin
concentration, ppbV
15
10
5
0
2.0
1.5
1.0
0.5
0.0
july
august
september
october
month
fish
reared in Adriatic Sea
reared in Tyrrhenian Sea
wild

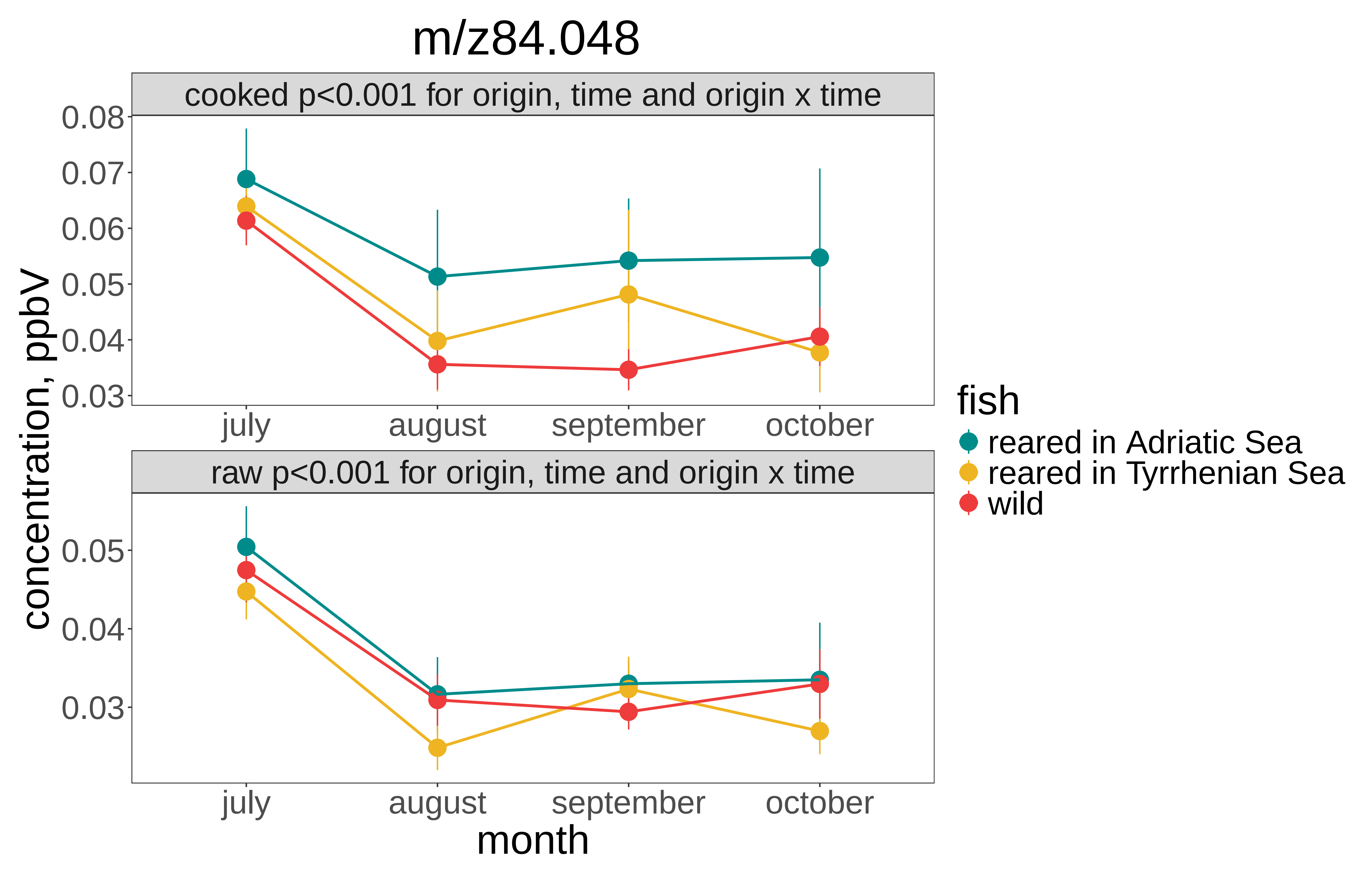
m/z84.048
cooked p<0.001 for origin, time and origin x time
0.08
0.07
0.06
0.05
0.04
0.03
july
august
september
october
raw p<0.001 for origin, time and origin x time
0.05
0.04
0.03
july
august
september
october
concentration, ppbV
month
fish
reared in Adriatic Sea
reared in Tyrrhenian Sea
wild

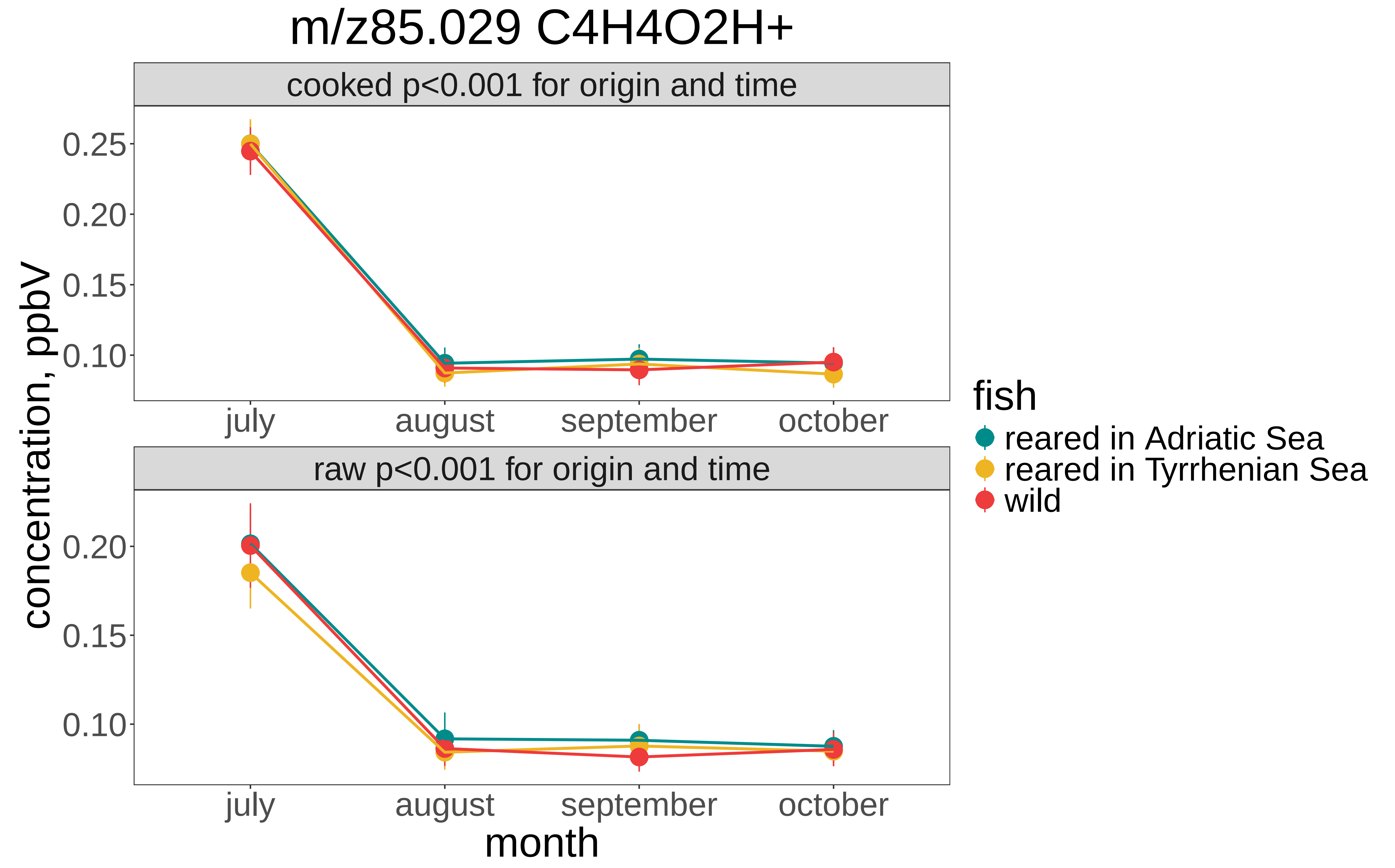
m/z85.029 C4H4O2H+
cooked p<0.001 for origin and time
raw p<0.001 for origin and time
0.25
0.20
0.15
0.10
july
august
september
october
month
concentration, ppbV
fish
reared in Adriatic Sea
reared in Tyrrhenian Sea
wild

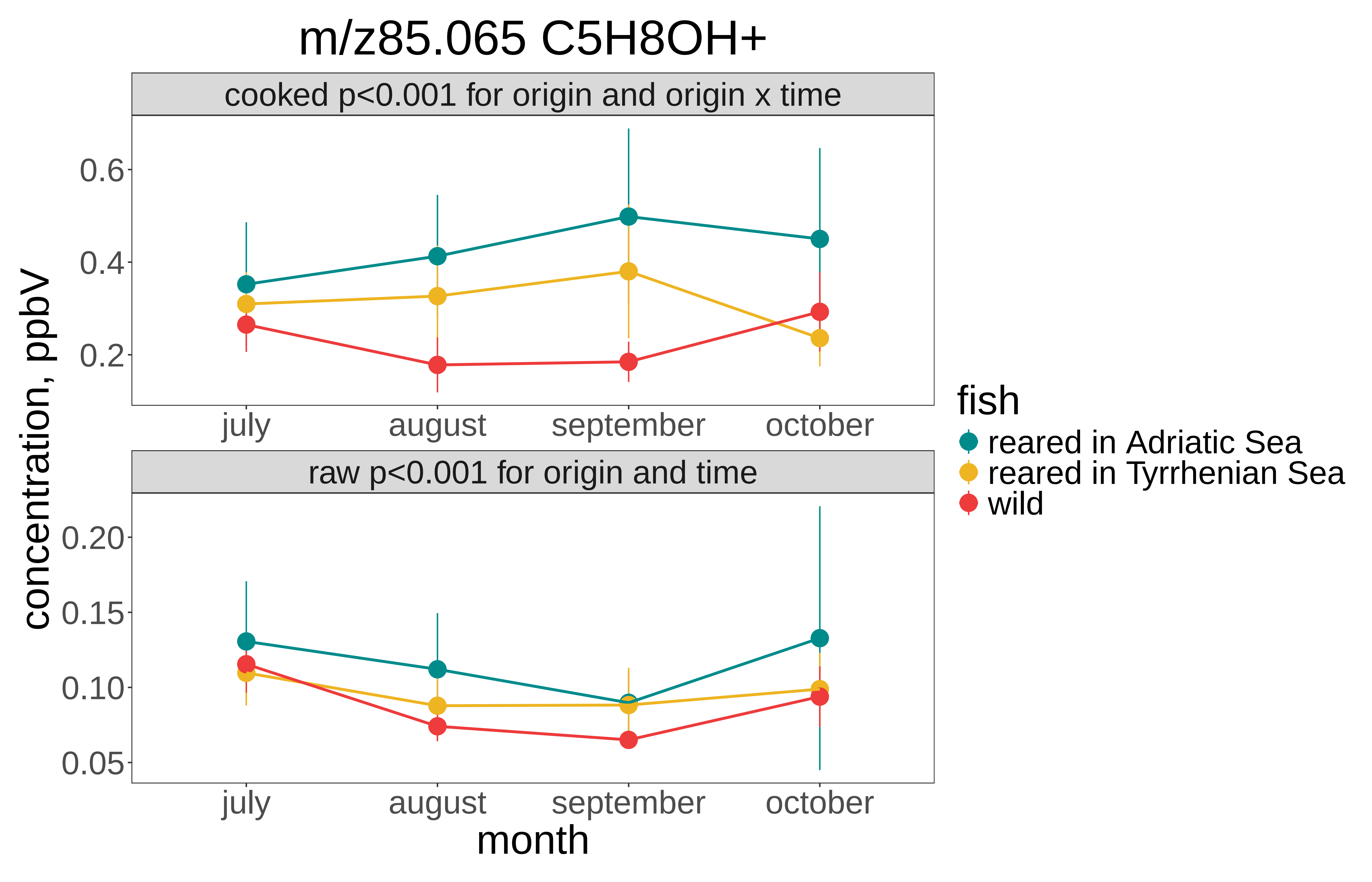
m/z85.065 C5H8OH+
cooked p<0.001 for origin and origin x time
raw p<0.001 for origin and time
0.6
0.4
0.2
0.20
0.15
0.10
0.05
july
august
september
october
concentration, ppbV
month
fish
reared in Adriatic Sea
reared in Tyrrhenian Sea
wild

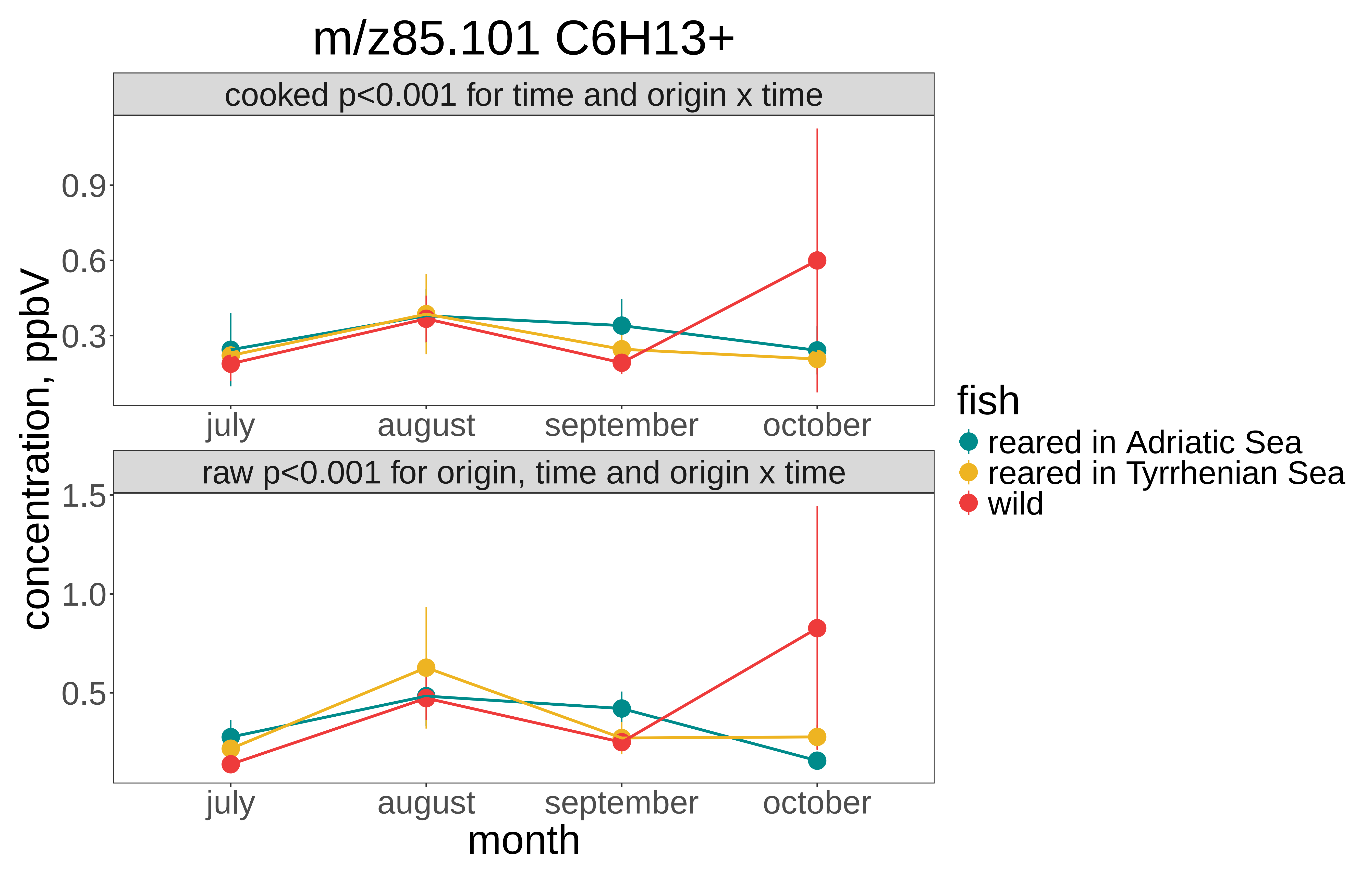
m/z85.101 C6H13+
cooked p<0.001 for time and origin x time
raw p<0.001 for origin, time and origin x time
concentration, ppbV
0.9
0.6
0.3
1.5
1.0
0.5
july
august
september
october
month
fish
reared in Adriatic Sea
reared in Tyrrhenian Sea
wild

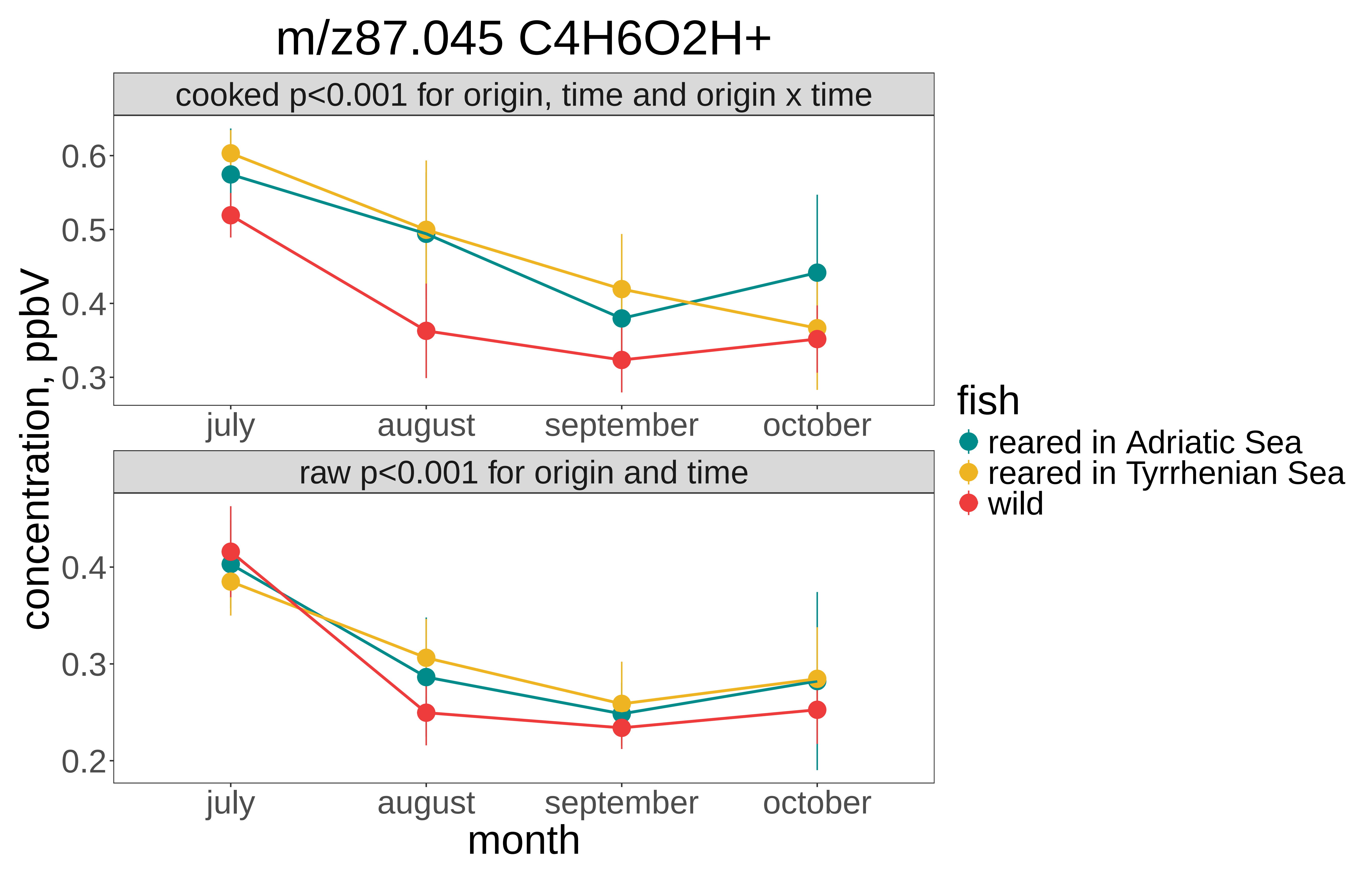
m/z87.045 C4H6O2H+
cooked p<0.001 for origin, time and origin x time
raw p<0.001 for origin and time
concentration, ppbV
0.6
0.5
0.4
0.3
0.4
0.3
0.2
july
august
september
october
month
fish
reared in Adriatic Sea
reared in Tyrrhenian Sea
wild

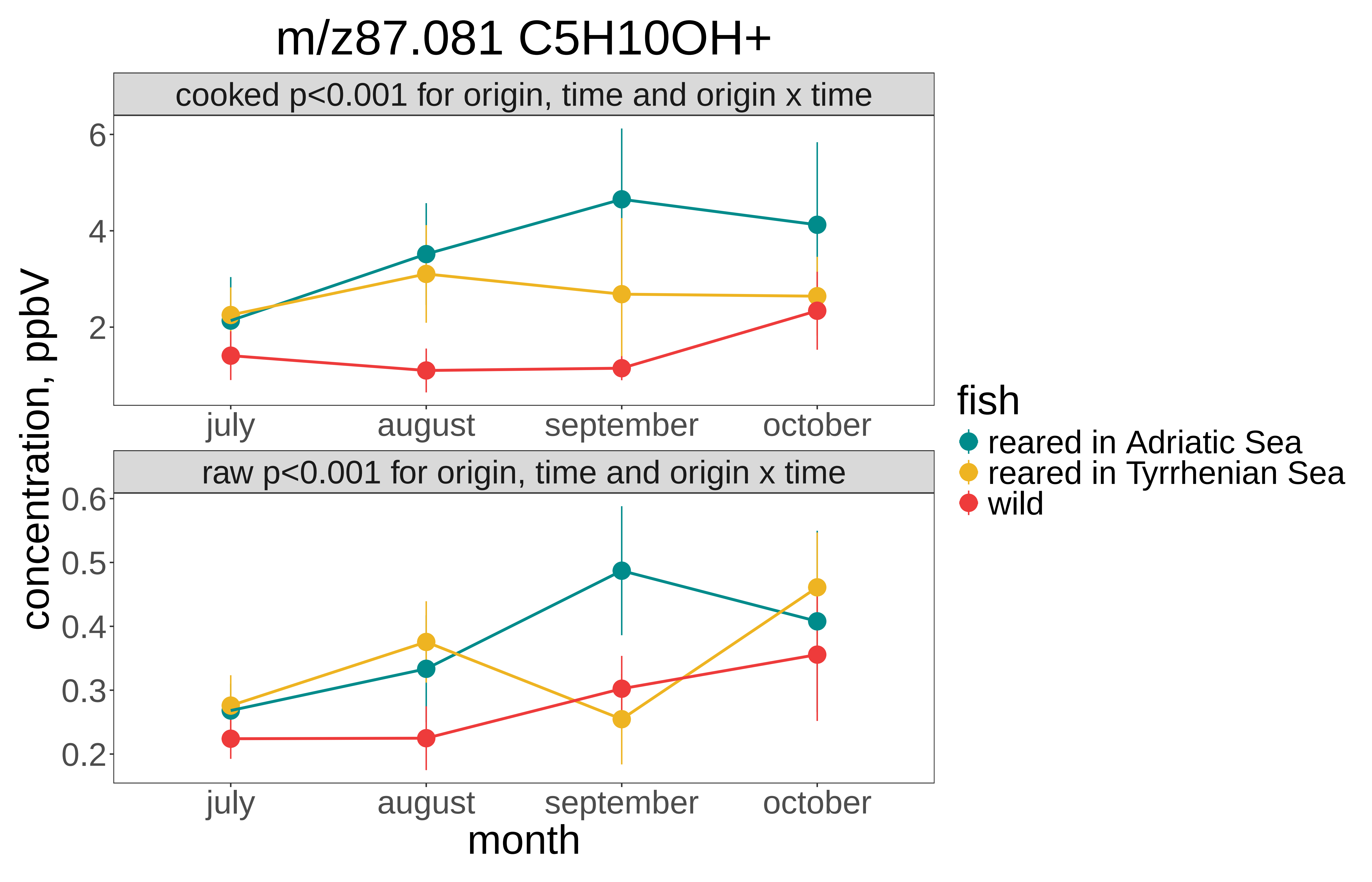
m/z87.081 C5H10OH+
cooked p<0.001 for origin, time and origin x time
raw p<0.001 for origin, time and origin x time
concentration, ppbV
month
july
august
september
october
6
4
2
0.6
0.5
0.4
0.3
0.2
fish
reared in Adriatic Sea
reared in Tyrrhenian Sea
wild

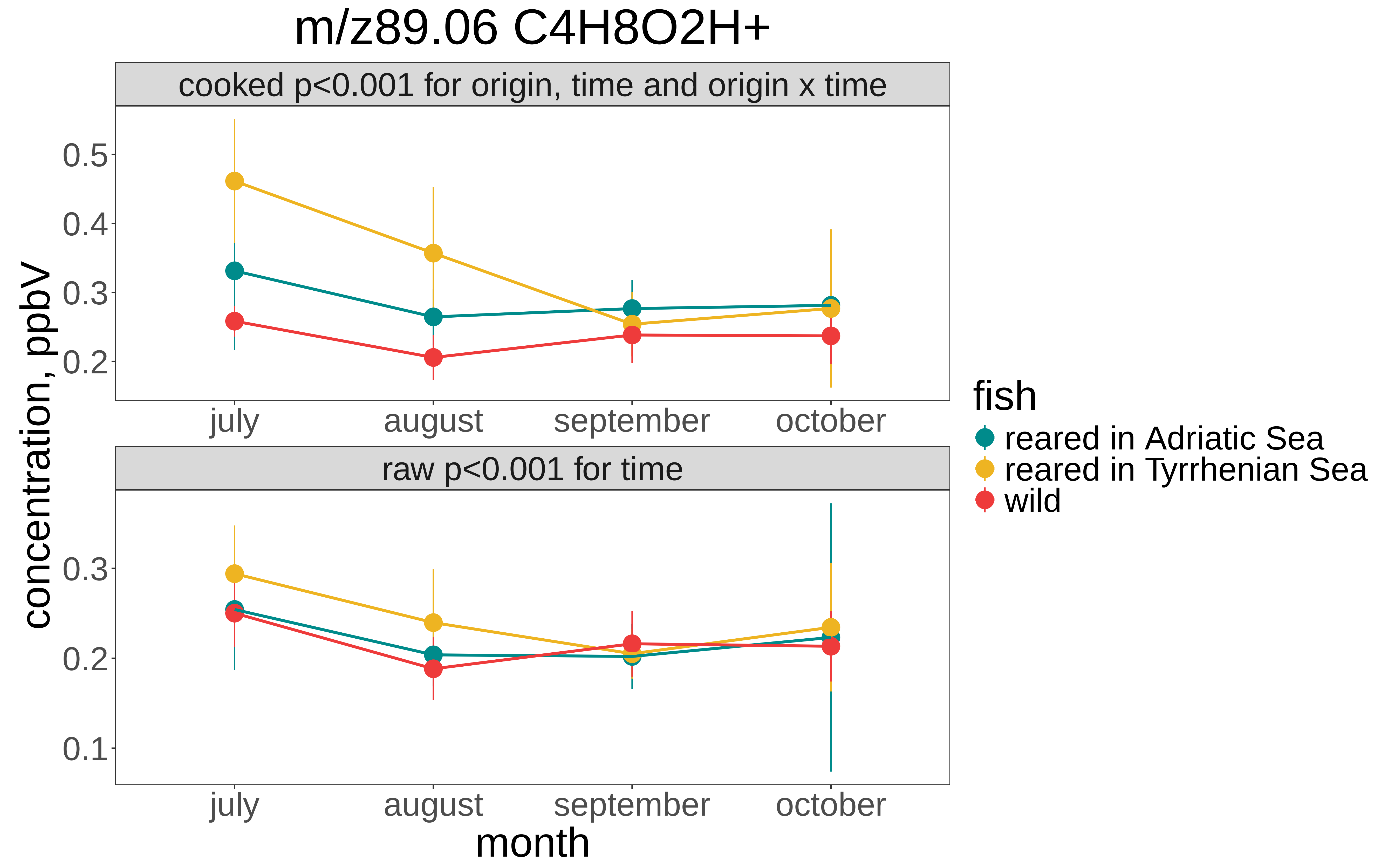
m/z89.06 C4H8O2H+
cooked p<0.001 for origin, time and origin x time
raw p<0.001 for time
concentration, ppbV
0.5
0.4
0.3
0.2
0.1
july
august
september
october
month
fish
reared in Adriatic Sea
reared in Tyrrhenian Sea
wild

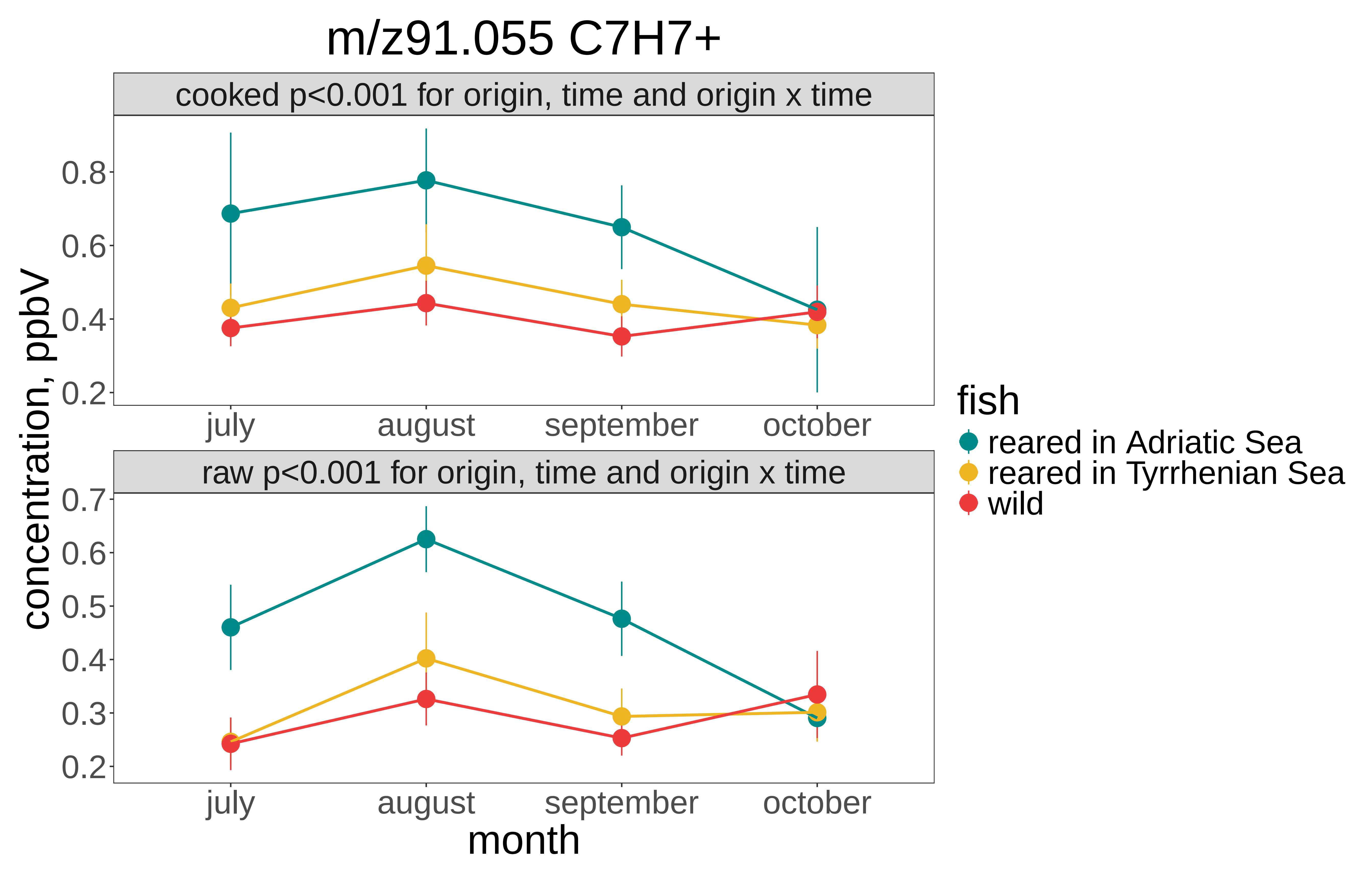
m/z91.055 C7H7+
cooked p<0.001 for origin, time and origin x time
raw p<0.001 for origin, time and origin x time
concentration, ppbV
0.8
0.6
0.4
0.2
0.7
0.6
0.5
0.4
0.3
0.2
july
august
september
october
month
fish
reared in Adriatic Sea
reared in Tyrrhenian Sea
wild

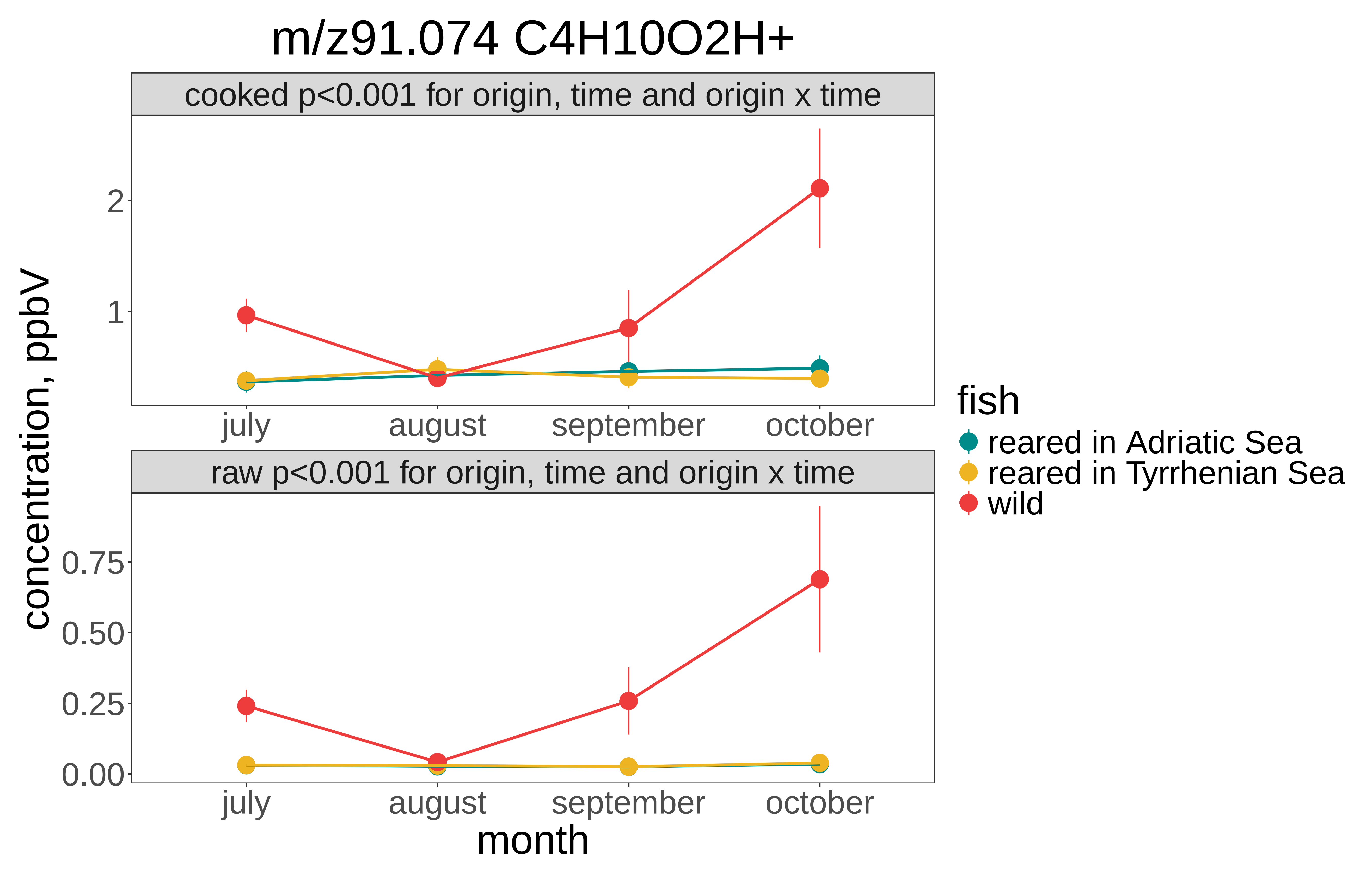
m/z91.074 C4H10O2H+
cooked p<0.001 for origin, time and origin x time
raw p<0.001 for origin, time and origin x time
concentration, ppbV
2
1
0.75
0.50
0.25
0.00
july
august
september
october
month
fish
reared in Adriatic Sea
reared in Tyrrhenian Sea
wild

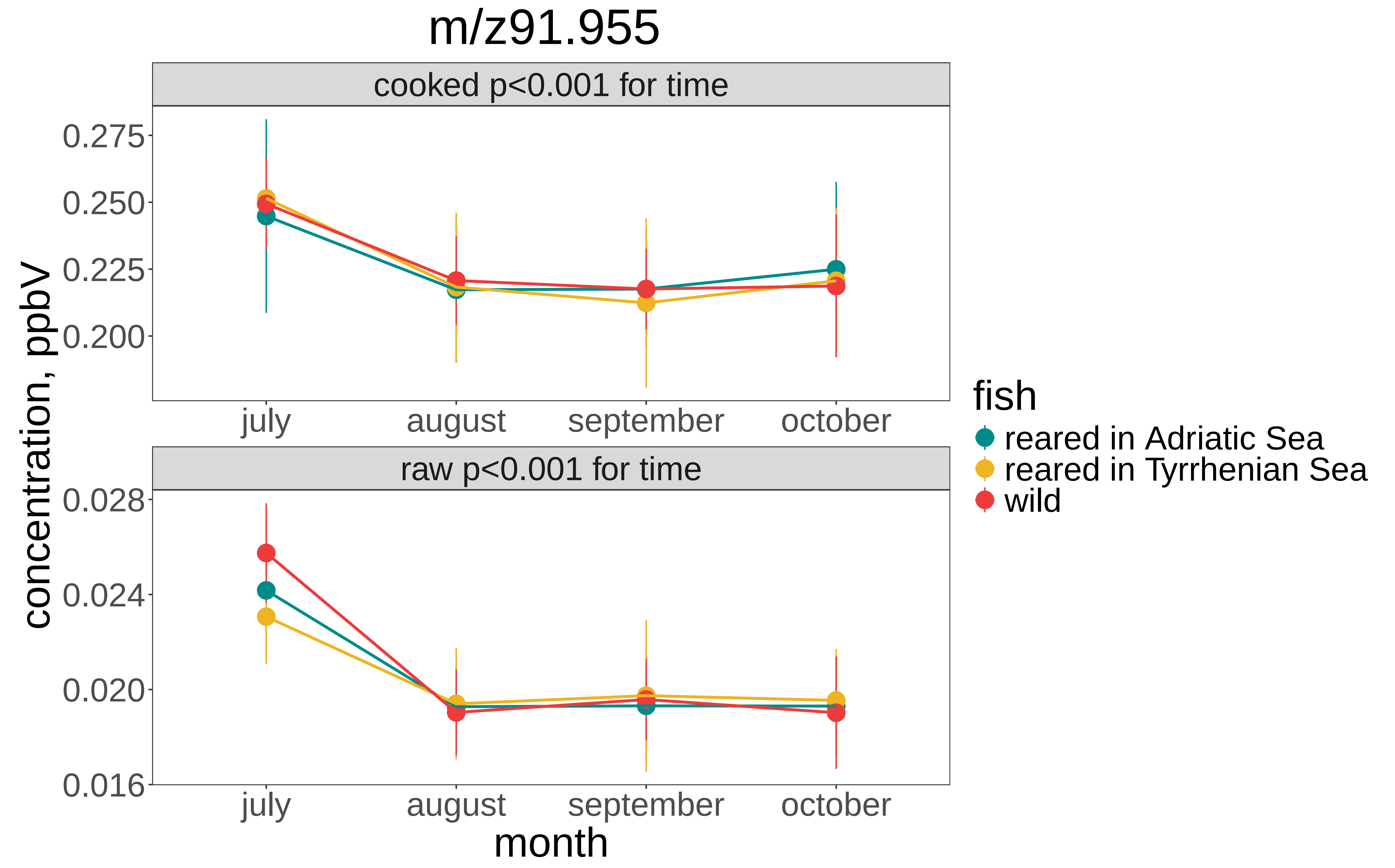

m/z91.955
cooked p<0.001 for time
0.275
0.250
0.225
0.200
july
august
september
october
raw p<0.001 for time
0.028
0.024
0.020
0.016
july
august
september
october
concentration, ppbV
month
fish
reared in Adriatic Sea
reared in Tyrrhenian Sea
wild

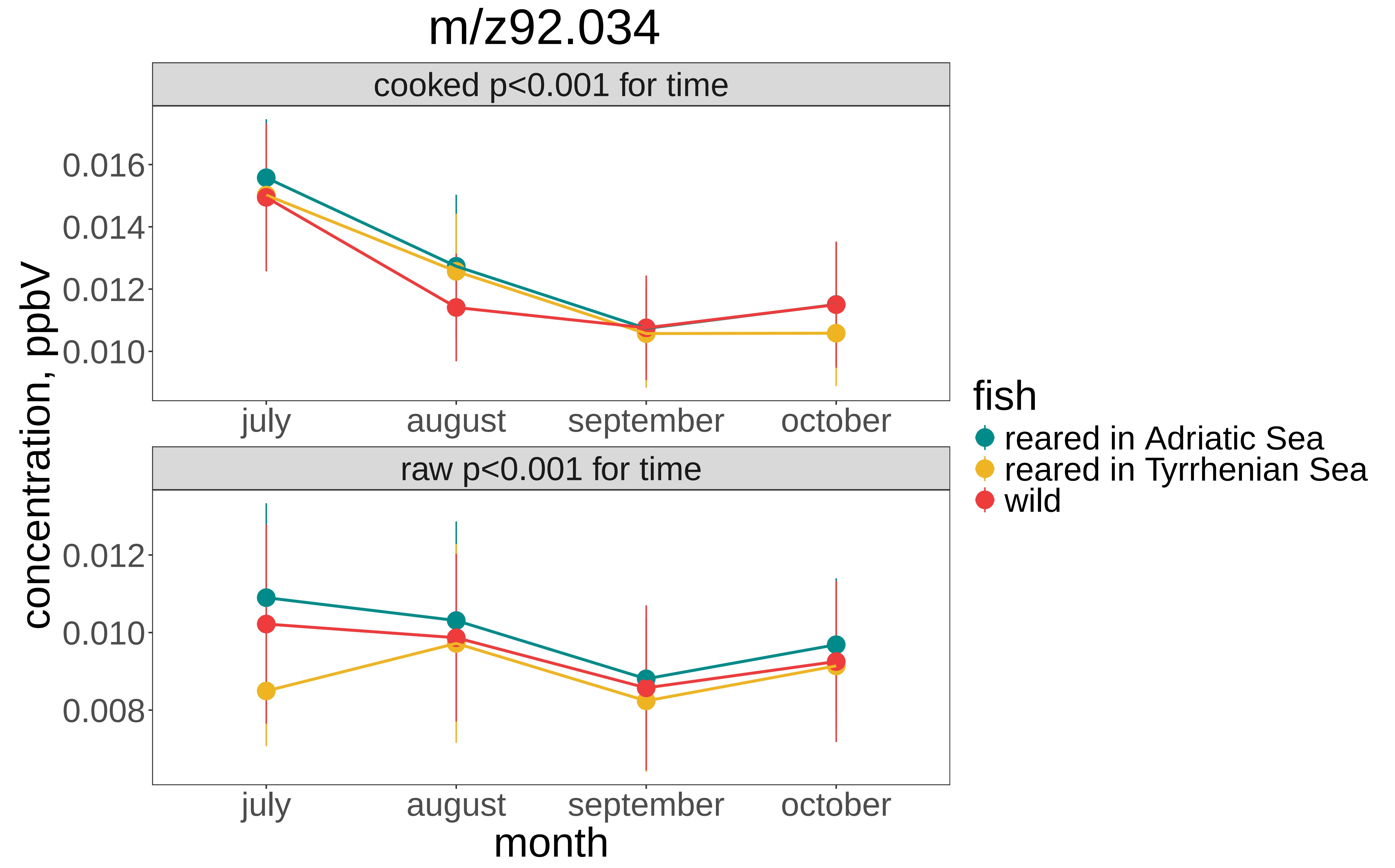
m/z92.034
cooked p<0.001 for time
raw p<0.001 for time
concentration, ppbV
0.016
0.014
0.012
0.010
0.008
july
august
september
october
month
fish
reared in Adriatic Sea
reared in Tyrrhenian Sea
wild

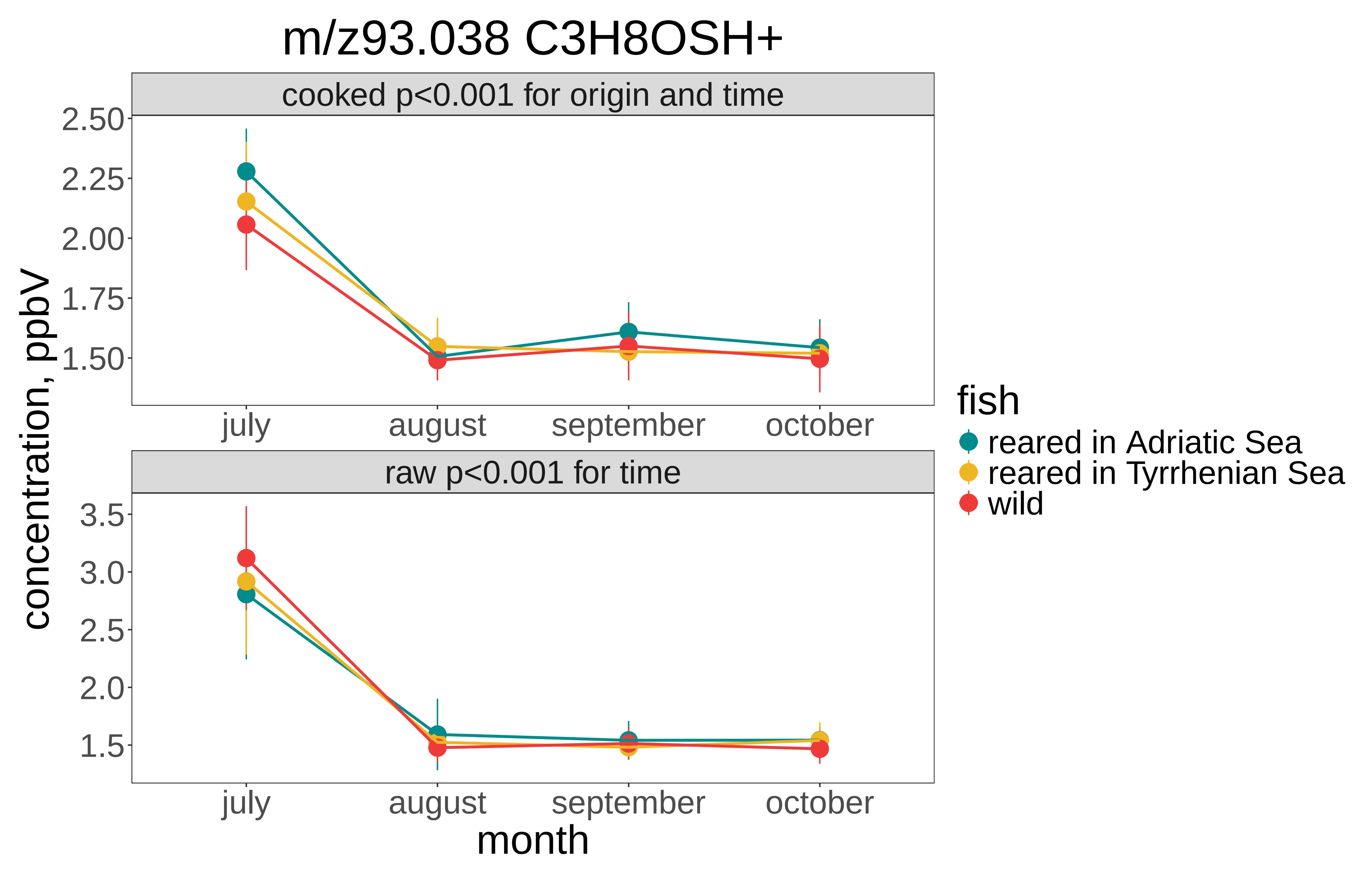
m/z93.038 C3H8OSH+
cooked p<0.001 for origin and time
raw p<0.001 for time
concentration, ppbV
2.50
2.25
2.00
1.75
1.50
3.5
3.0
2.5
2.0
1.5
july
august
september
october
month
fish
reared in Adriatic Sea
reared in Tyrrhenian Sea
wild

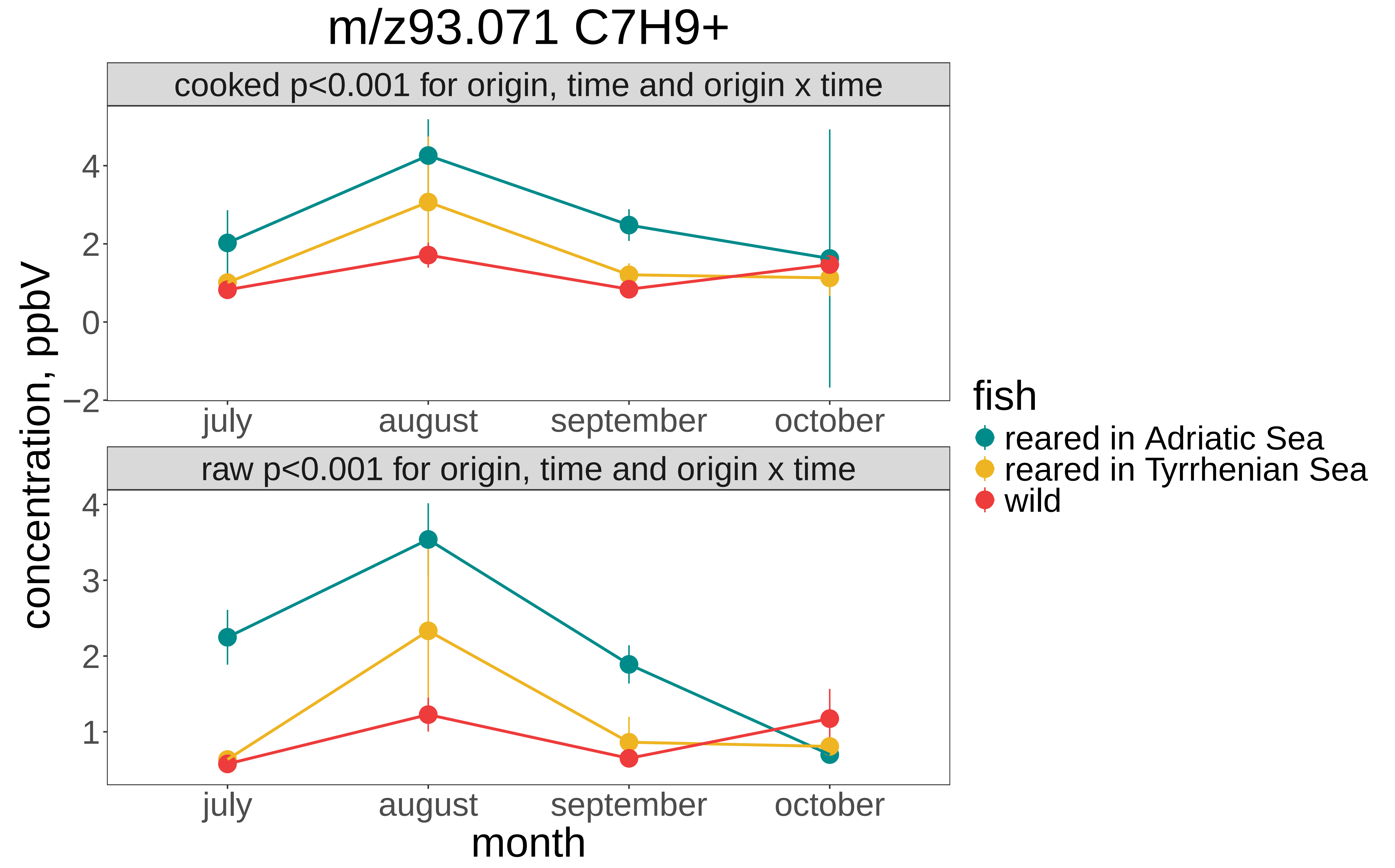

m/z93.071 C7H9+
cooked p<0.001 for origin, time and origin x time
raw p<0.001 for origin, time and origin x time
concentration, ppbV
4
2
0
−2
4
3
2
1
july
august
september
october
month
fish
reared in Adriatic Sea
reared in Tyrrhenian Sea
wild

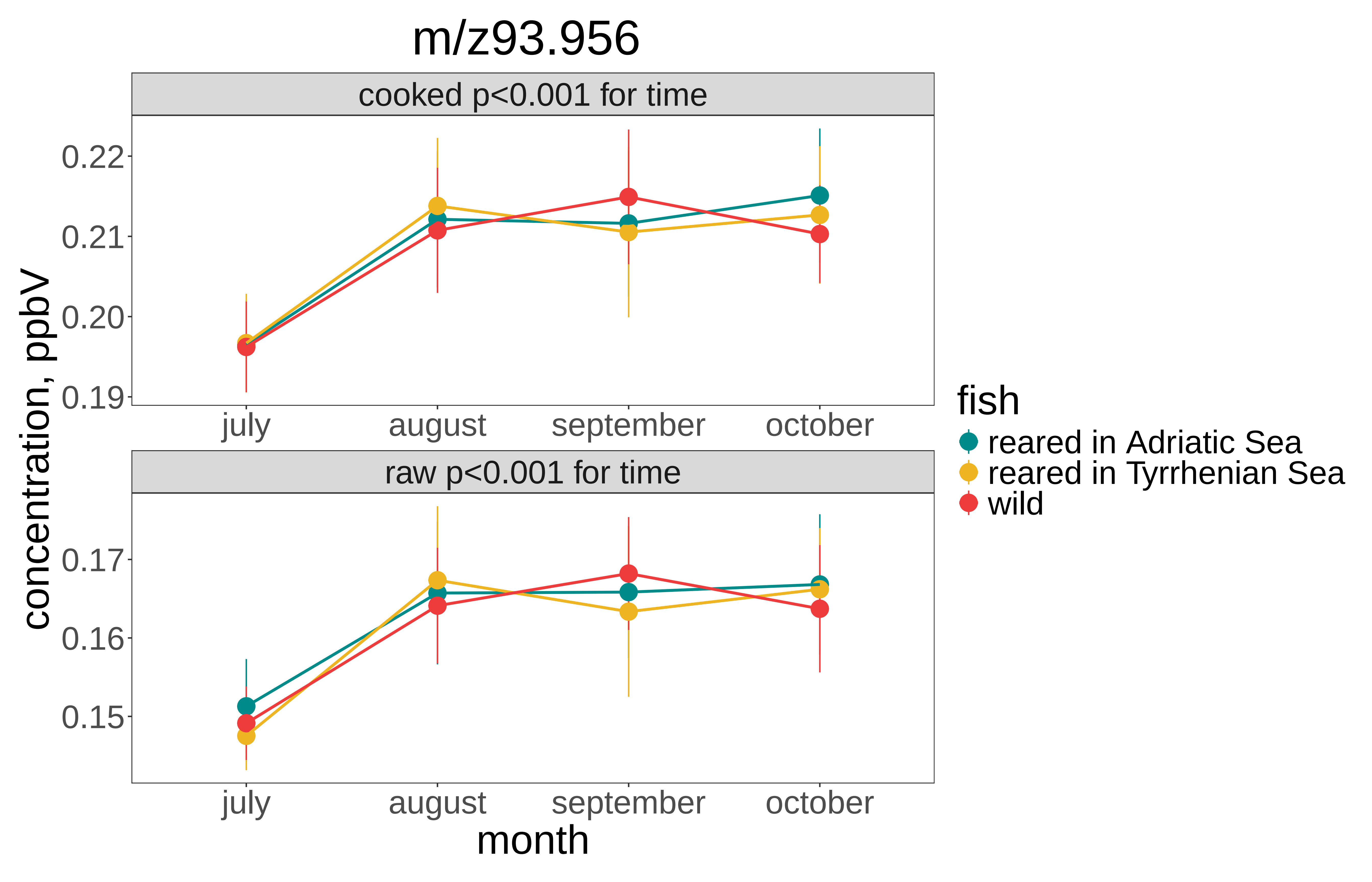
m/z93.956
cooked p<0.001 for time
raw p<0.001 for time
concentration, ppbV
0.22
0.21
0.20
0.19
0.17
0.16
0.15
july
august
september
october
month
fish
reared in Adriatic Sea
reared in Tyrrhenian Sea
wild

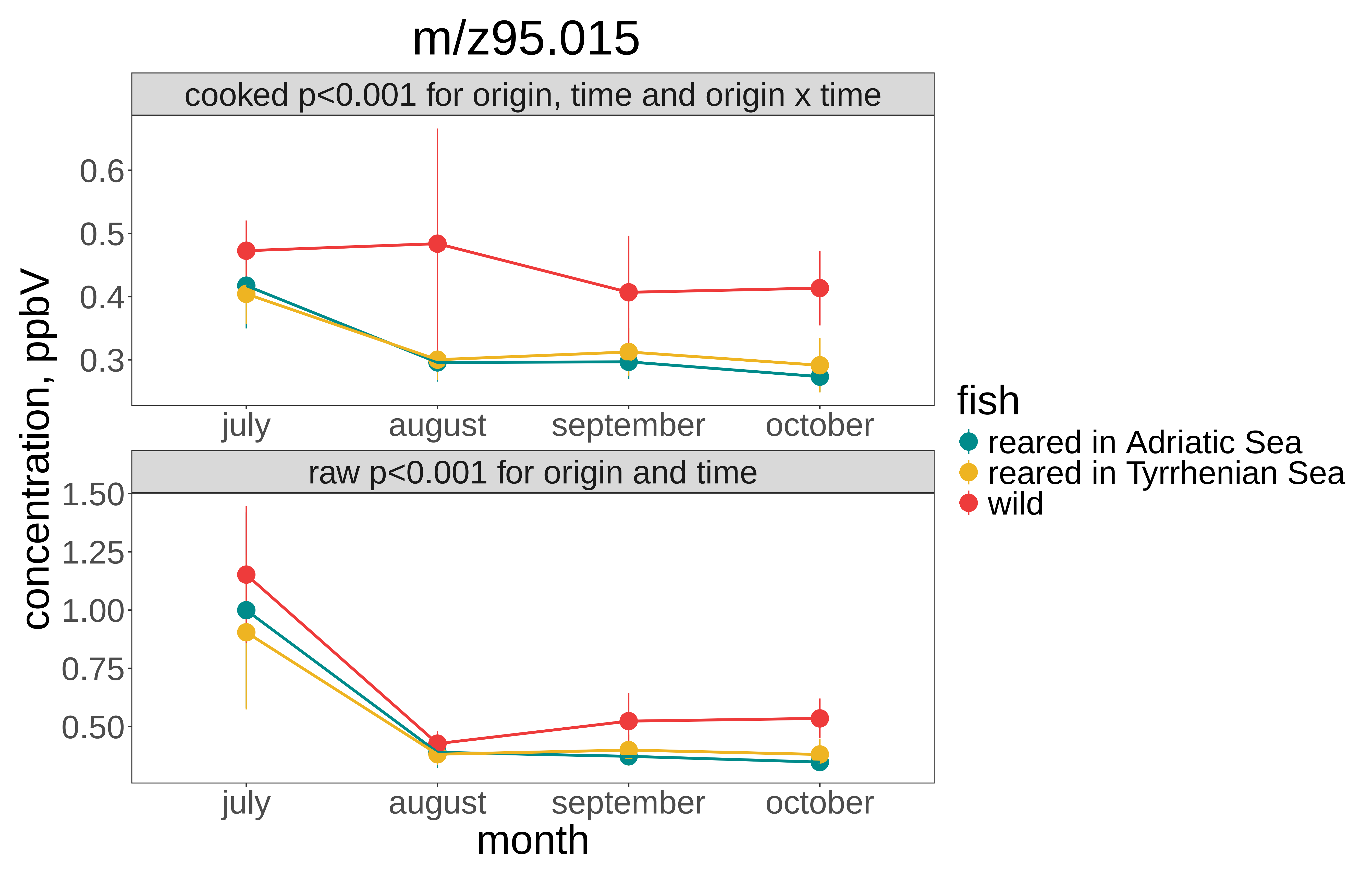

m/z95.015
cooked p<0.001 for origin, time and origin x time
raw p<0.001 for origin and time
concentration, ppbV
0.6
0.5
0.4
0.3
1.50
1.25
1.00
0.75
0.50
july
august
september
october
month
fish
reared in Adriatic Sea
reared in Tyrrhenian Sea
wild

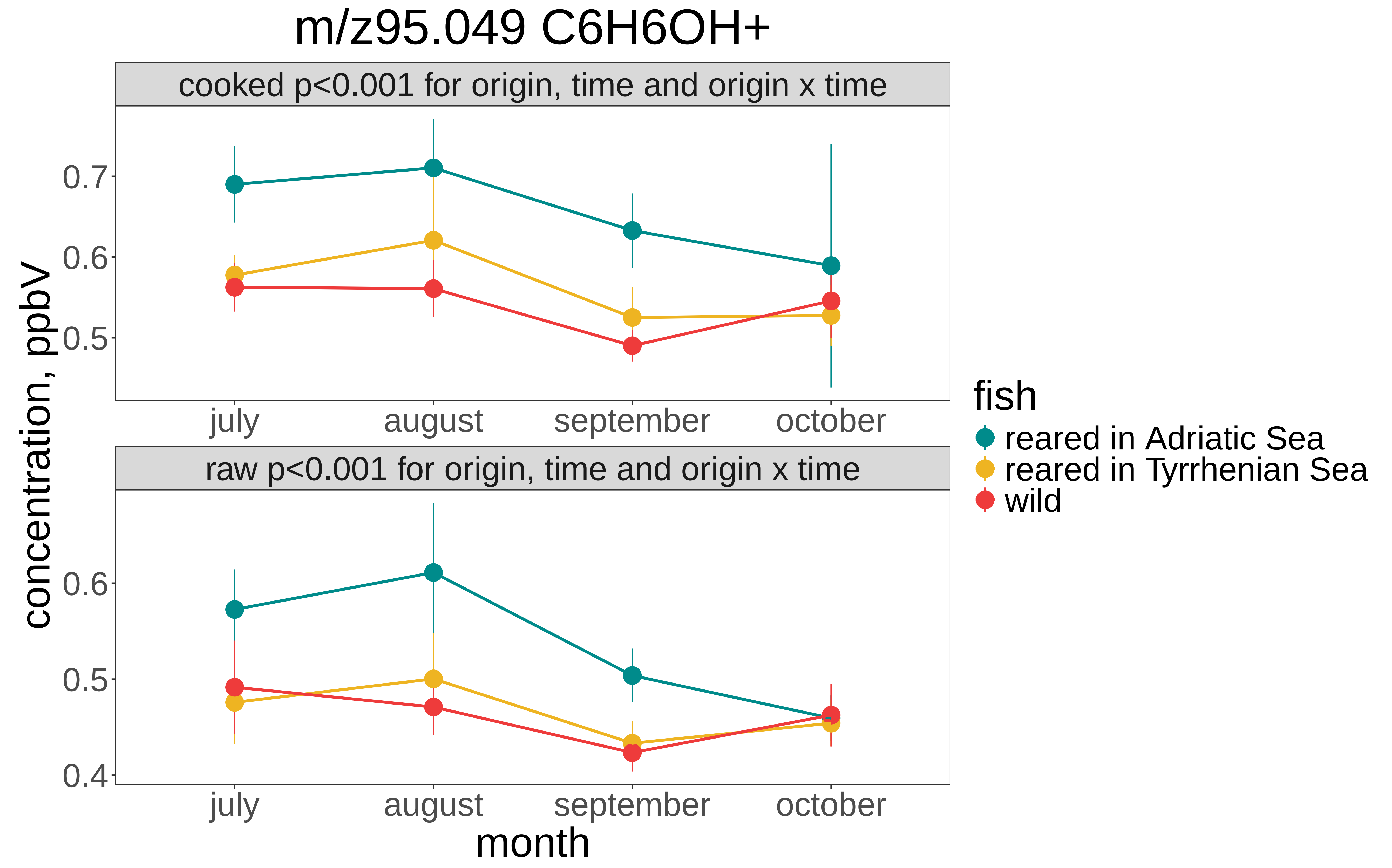
m/z95.049 C6H6OH+
cooked p<0.001 for origin, time and origin x time
raw p<0.001 for origin, time and origin x time
concentration, ppbV
0.7
0.6
0.5
0.4
july
august
september
october
month
fish
reared in Adriatic Sea
reared in Tyrrhenian Sea
wild

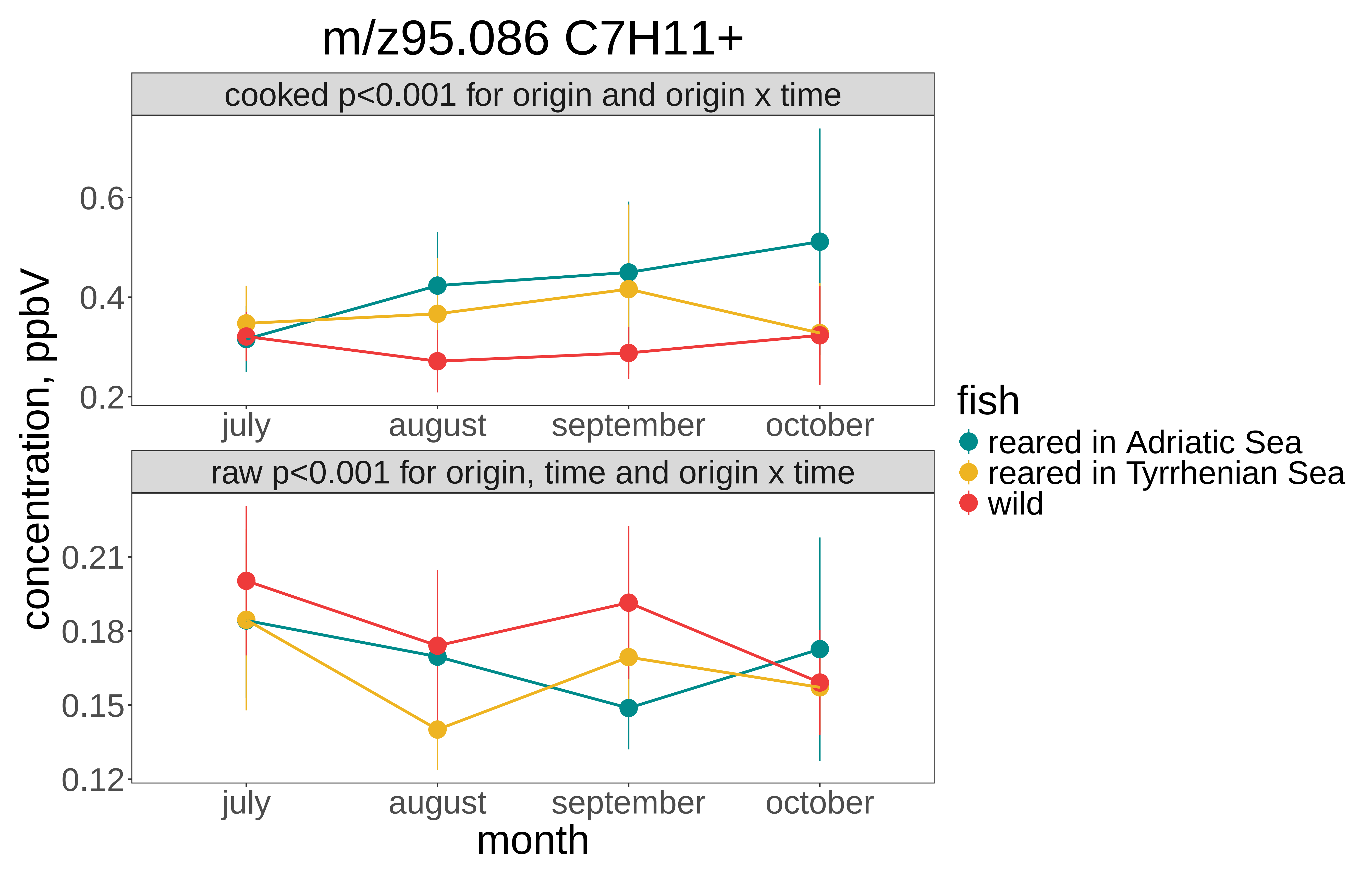
m/z95.086 C7H11+
cooked p<0.001 for origin and origin x time
raw p<0.001 for origin, time and origin x time
concentration, ppbV
0.6
0.4
0.2
0.21
0.18
0.15
0.12
july
august
september
october
month
fish
reared in Adriatic Sea
reared in Tyrrhenian Sea
wild

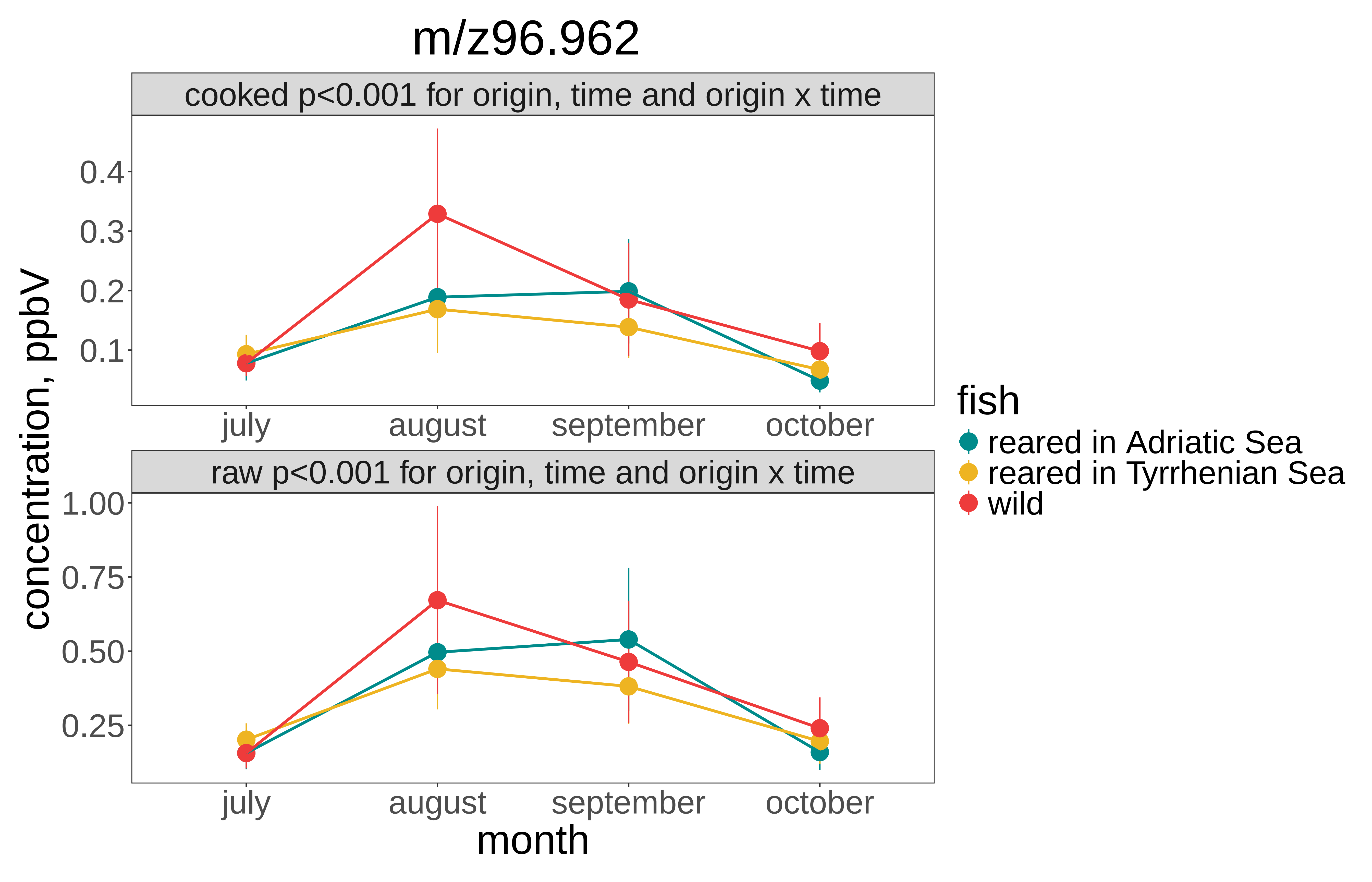

m/z96.962
cooked p<0.001 for origin, time and origin x time
raw p<0.001 for origin, time and origin x time
concentration, ppbV
0.1
0.2
0.3
0.4
0.25
0.50
0.75
1.00
july
august
september
october
month
fish
reared in Adriatic Sea
reared in Tyrrhenian Sea
wild

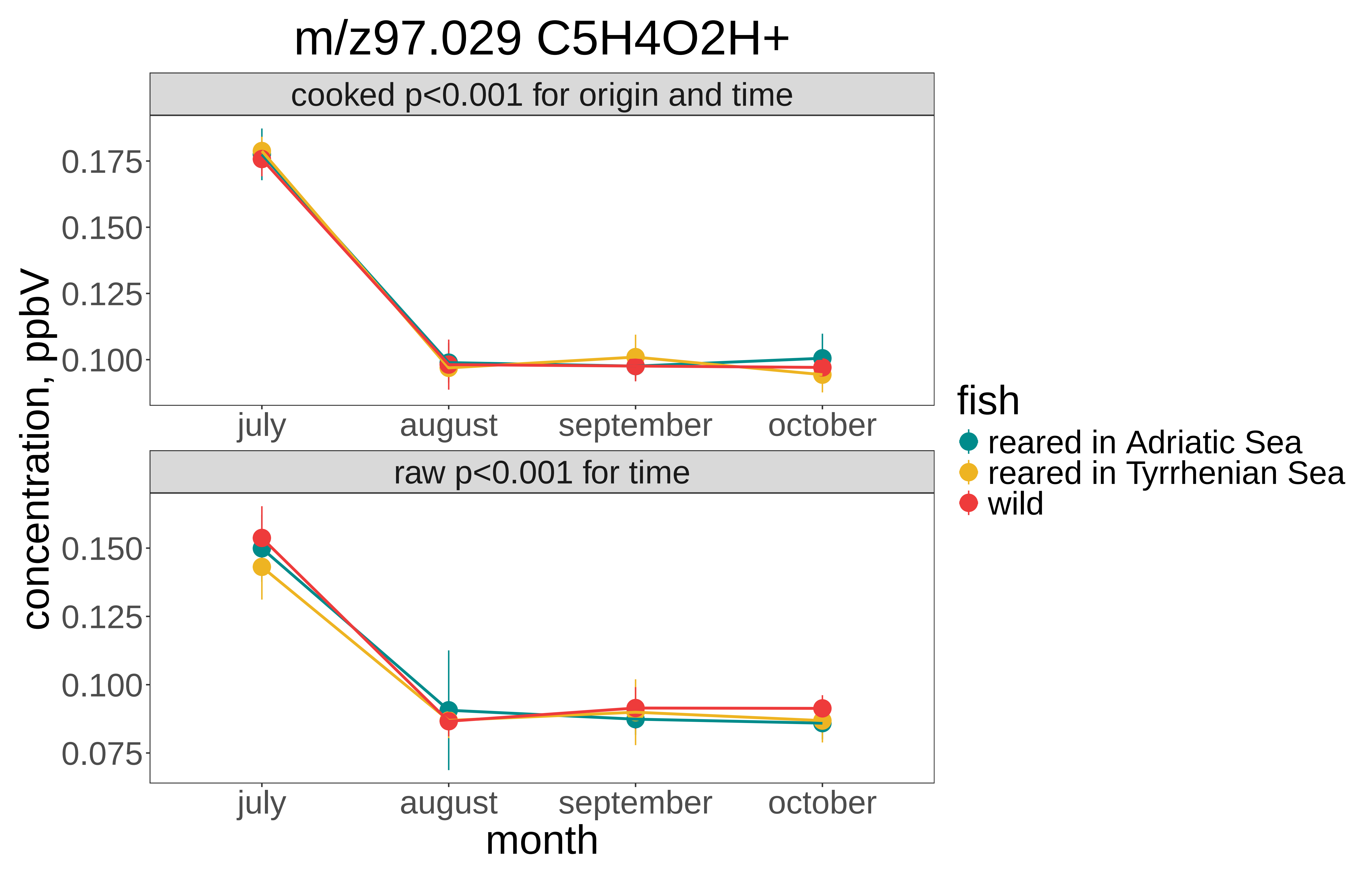
m/z97.029 C5H4O2H+
cooked p<0.001 for origin and time
raw p<0.001 for time
concentration, ppbV
0.175
0.150
0.125
0.100
0.075
july
august
september
october
month
fish
reared in Adriatic Sea
reared in Tyrrhenian Sea
wild

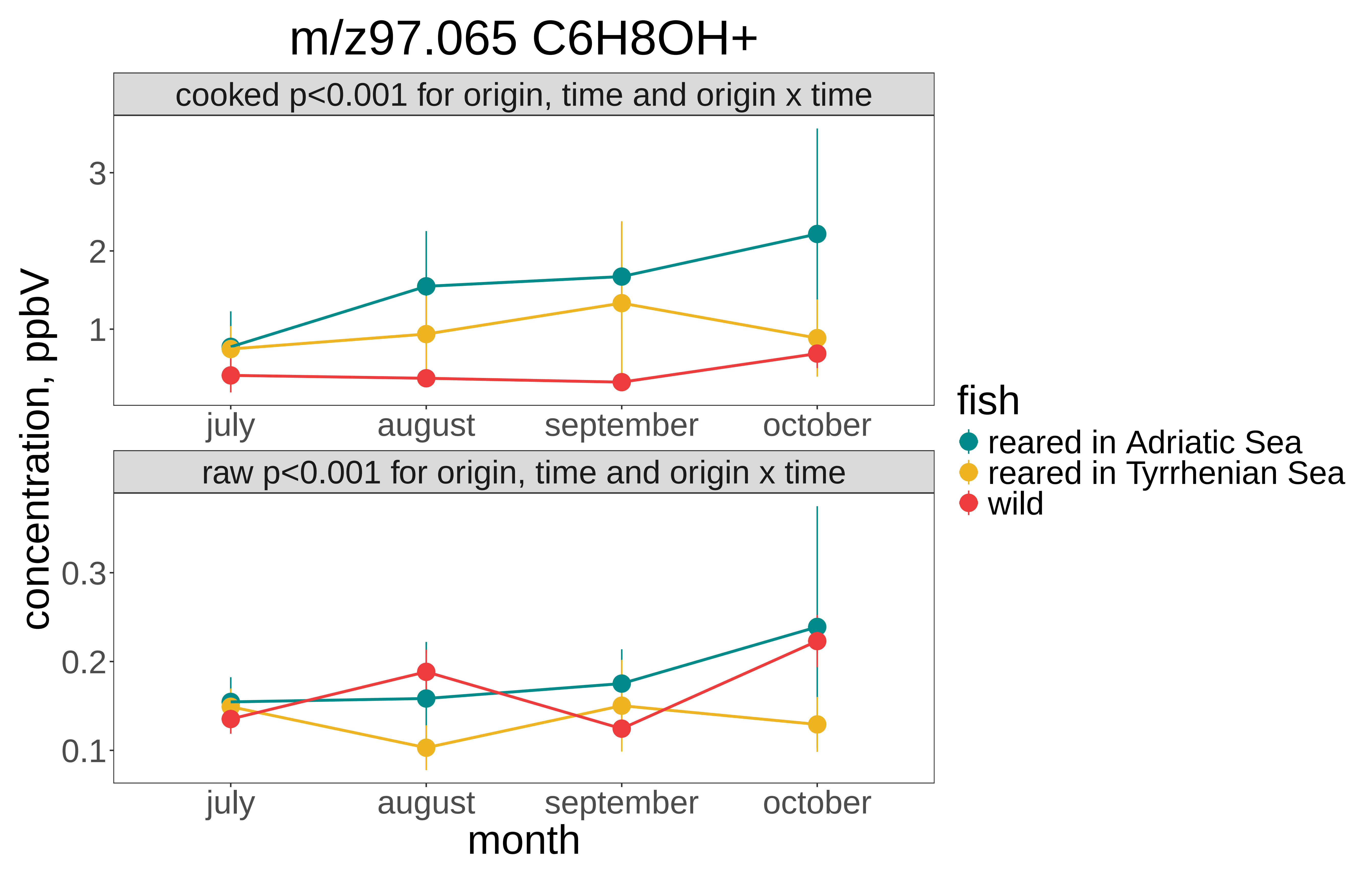
m/z97.065 C6H8OH+
cooked p<0.001 for origin, time and origin x time
raw p<0.001 for origin, time and origin x time
concentration, ppbV
3
2
1
0.3
0.2
0.1
july
august
september
october
month
fish
reared in Adriatic Sea
reared in Tyrrhenian Sea
wild

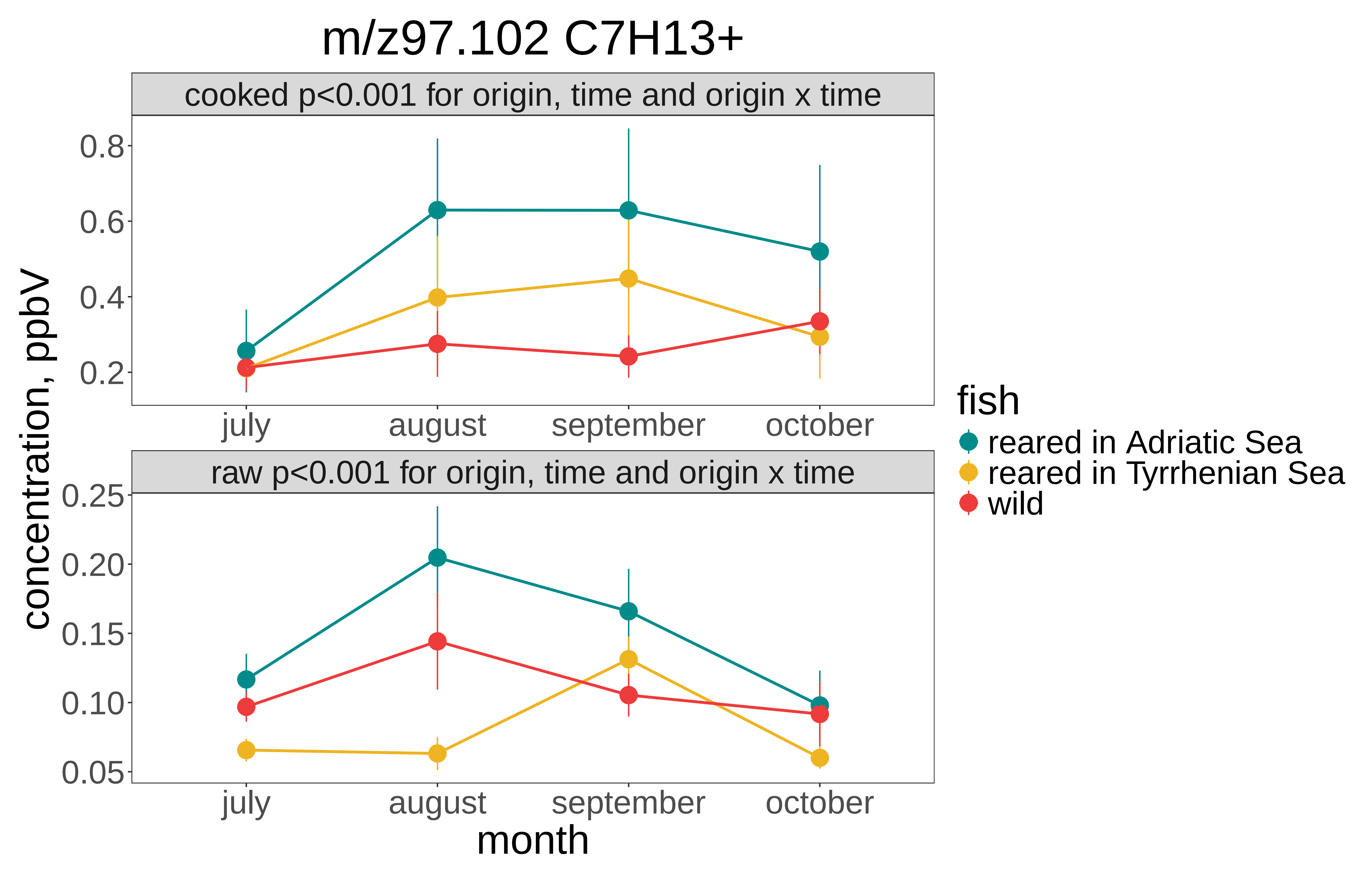
m/z97.102 C7H13+
cooked p<0.001 for origin, time and origin x time
raw p<0.001 for origin, time and origin x time
concentration, ppbV
0.8
0.6
0.4
0.2
0.25
0.20
0.15
0.10
0.05
july
august
september
october
month
fish
reared in Adriatic Sea
reared in Tyrrhenian Sea
wild

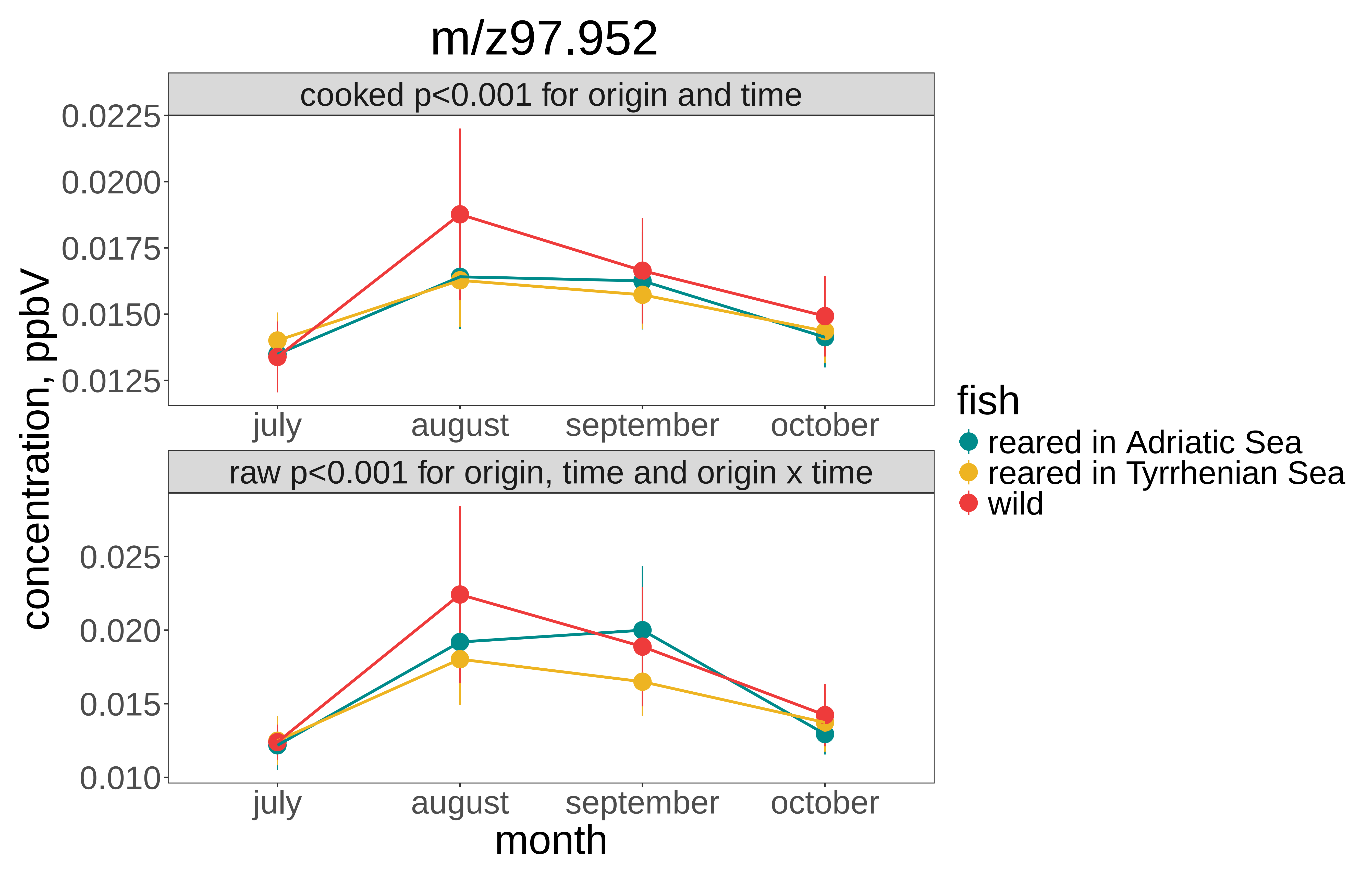
m/z97.952
cooked p<0.001 for origin and time
raw p<0.001 for origin, time and origin x time
0.0225
0.0200
0.0175
0.0150
0.0125
0.025
0.020
0.015
0.010
concentration, ppbV
july
august
september
october
month
fish
reared in Adriatic Sea
reared in Tyrrhenian Sea
wild

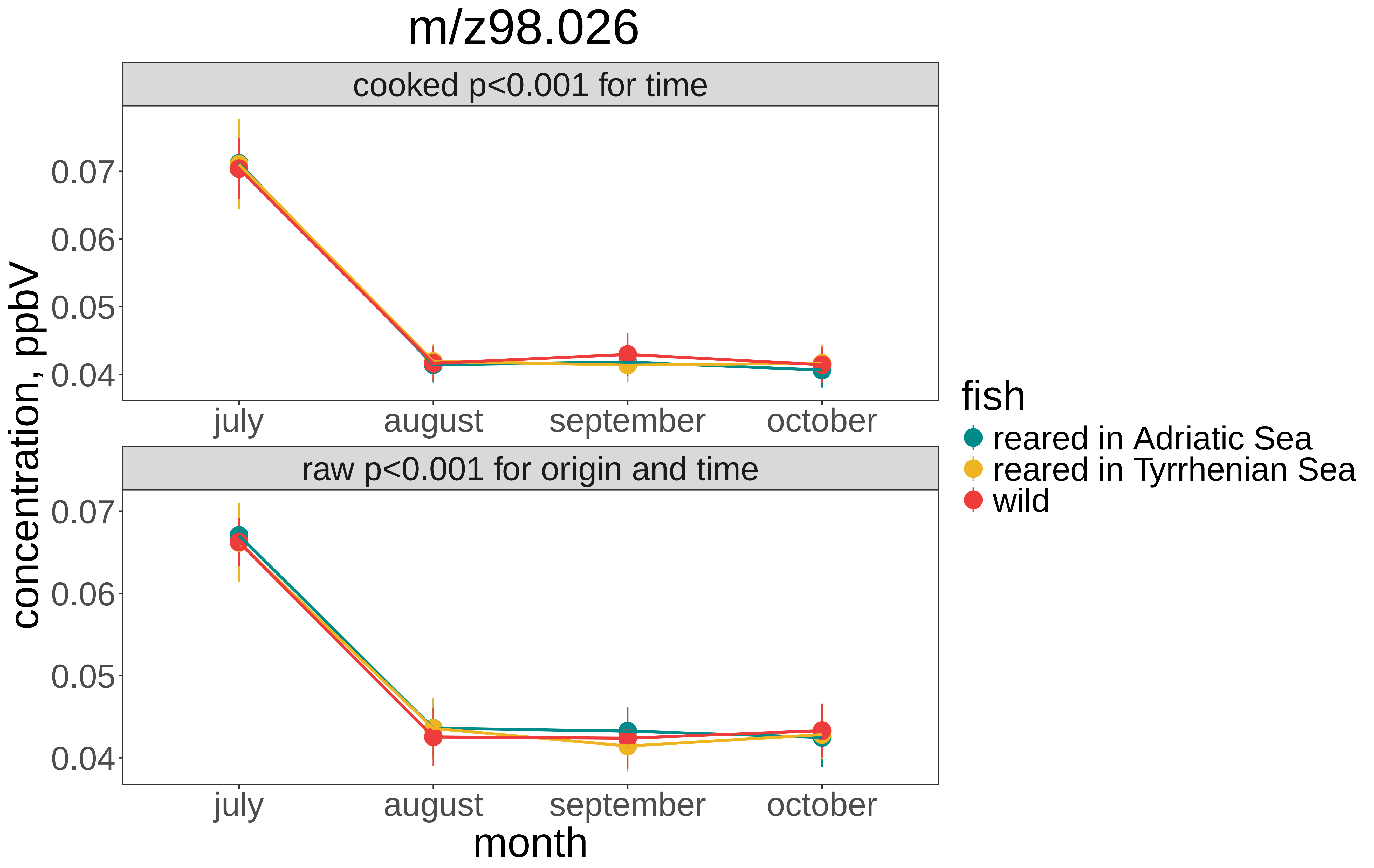

m/z98.026
cooked p<0.001 for time
raw p<0.001 for origin and time
concentration, ppbV
0.07
0.06
0.05
0.04
july
august
september
october
month
fish
reared in Adriatic Sea
reared in Tyrrhenian Sea
wild

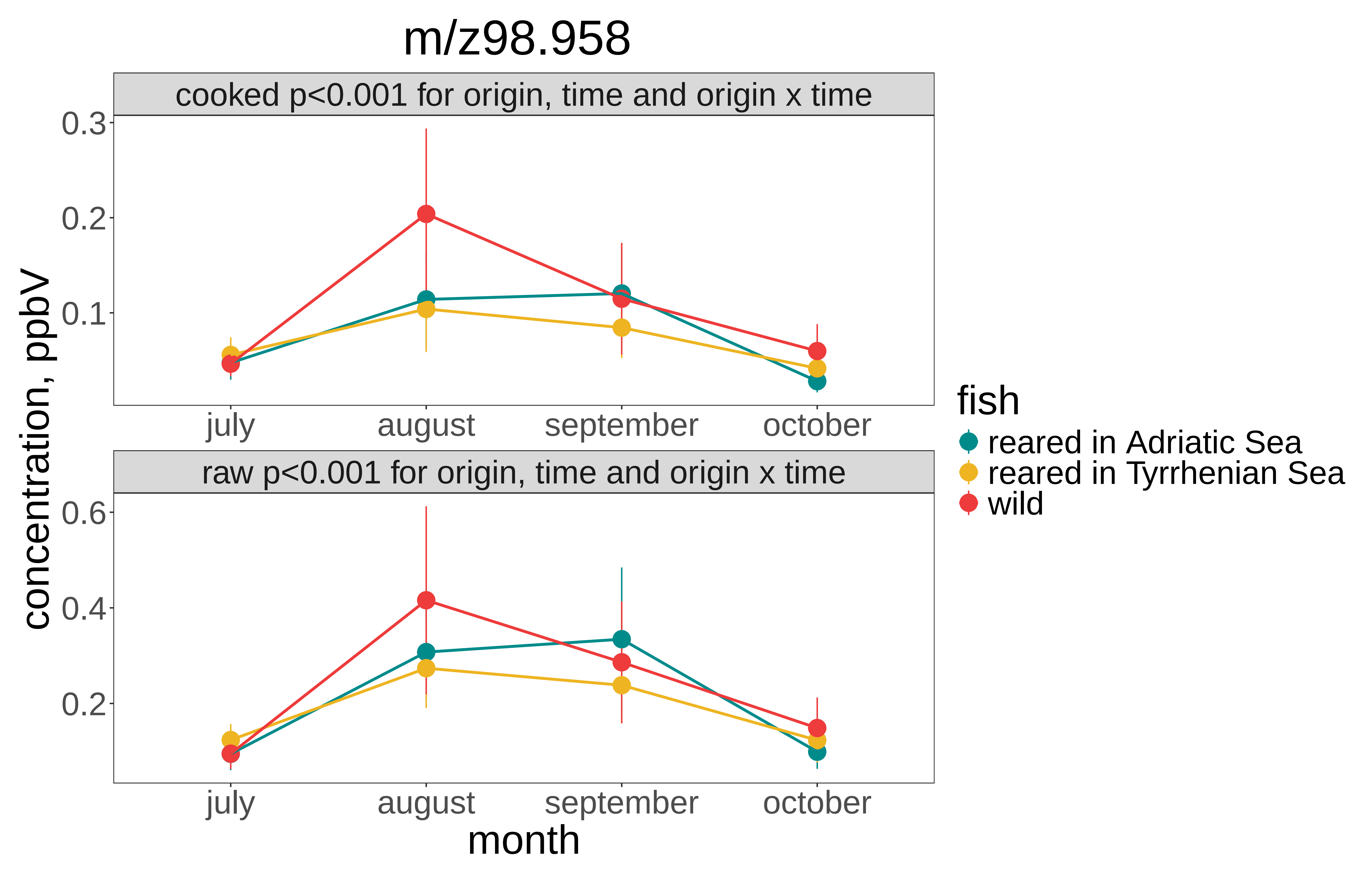

m/z98.958
cooked p<0.001 for origin, time and origin x time
raw p<0.001 for origin, time and origin x time
0.3
0.2
0.1
0.6
0.4
0.2
concentration, ppbV
july
august
september
october
month
fish
reared in Adriatic Sea
reared in Tyrrhenian Sea
wild

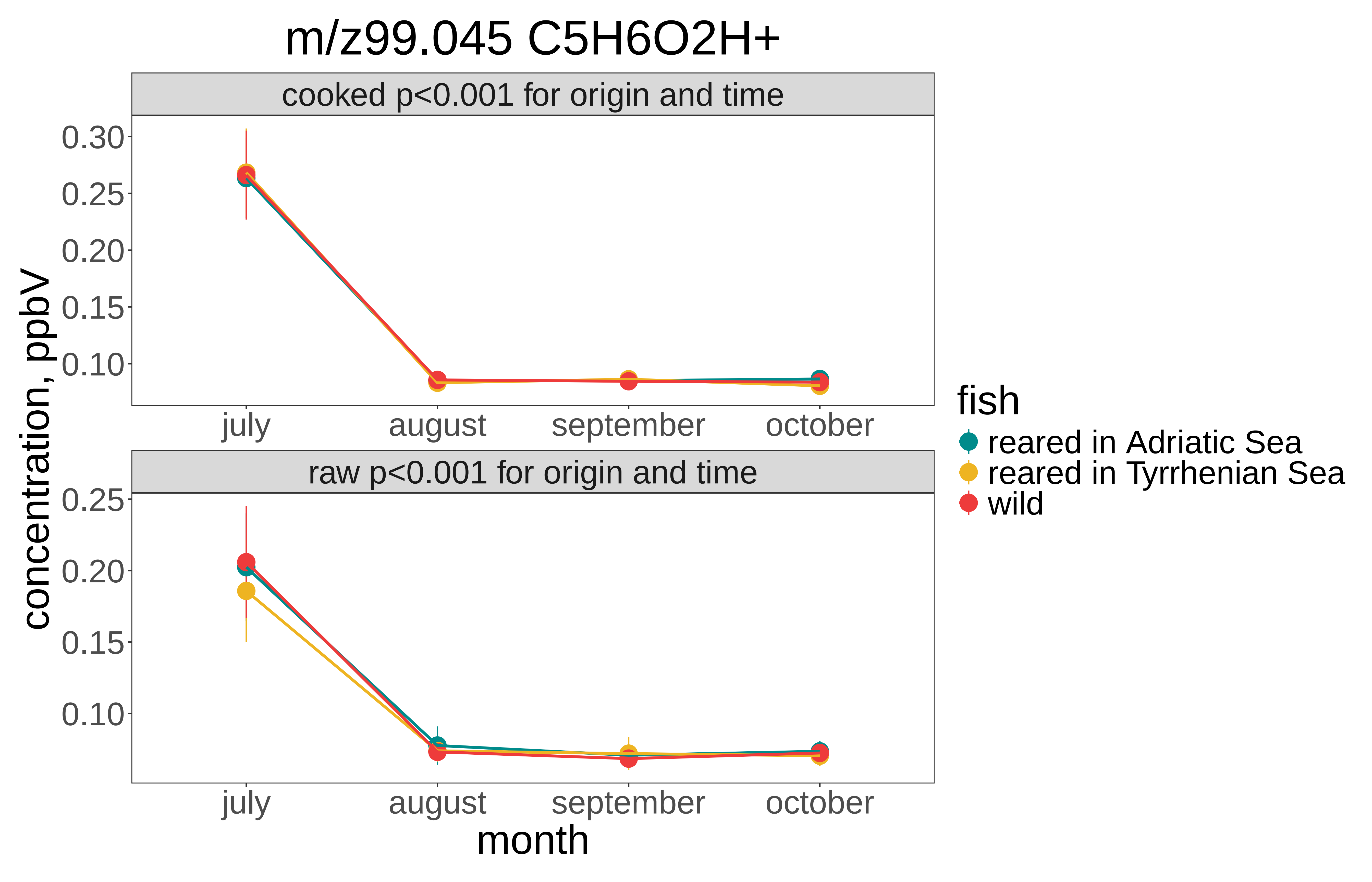

m/z99.045 C5H6O2H+
cooked p<0.001 for origin and time
raw p<0.001 for origin and time
concentration, ppbV
0.30
0.25
0.20
0.15
0.10
july
august
september
october
month
fish
reared in Adriatic Sea
reared in Tyrrhenian Sea
wild

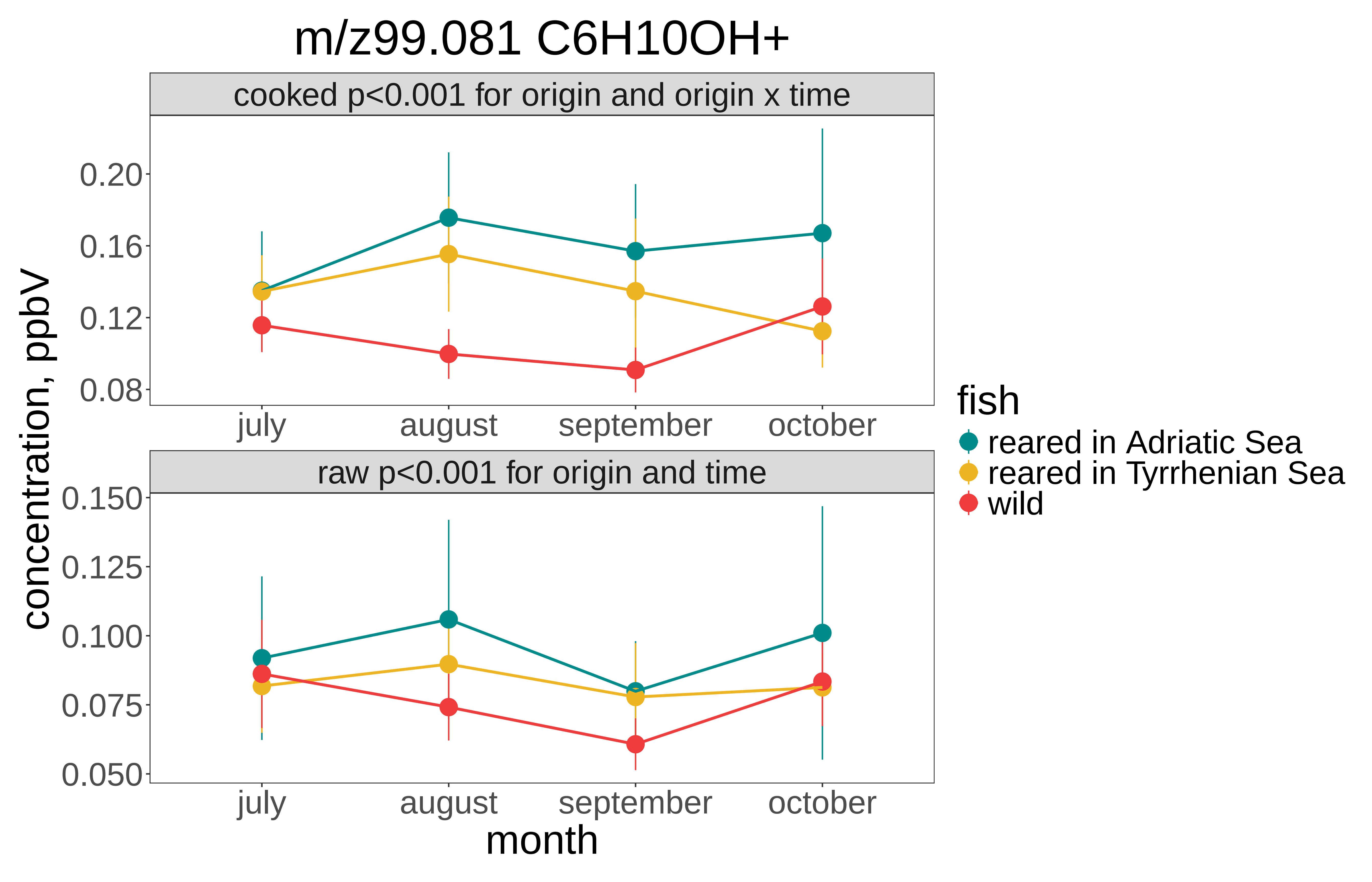

m/z99.081 C6H10OH+
cooked p<0.001 for origin and origin x time
raw p<0.001 for origin and time
0.20
0.16
0.12
0.08
0.150
0.125
0.100
0.075
0.050
july
august
september
october
concentration, ppbV
month
fish
reared in Adriatic Sea
reared in Tyrrhenian Sea
wild

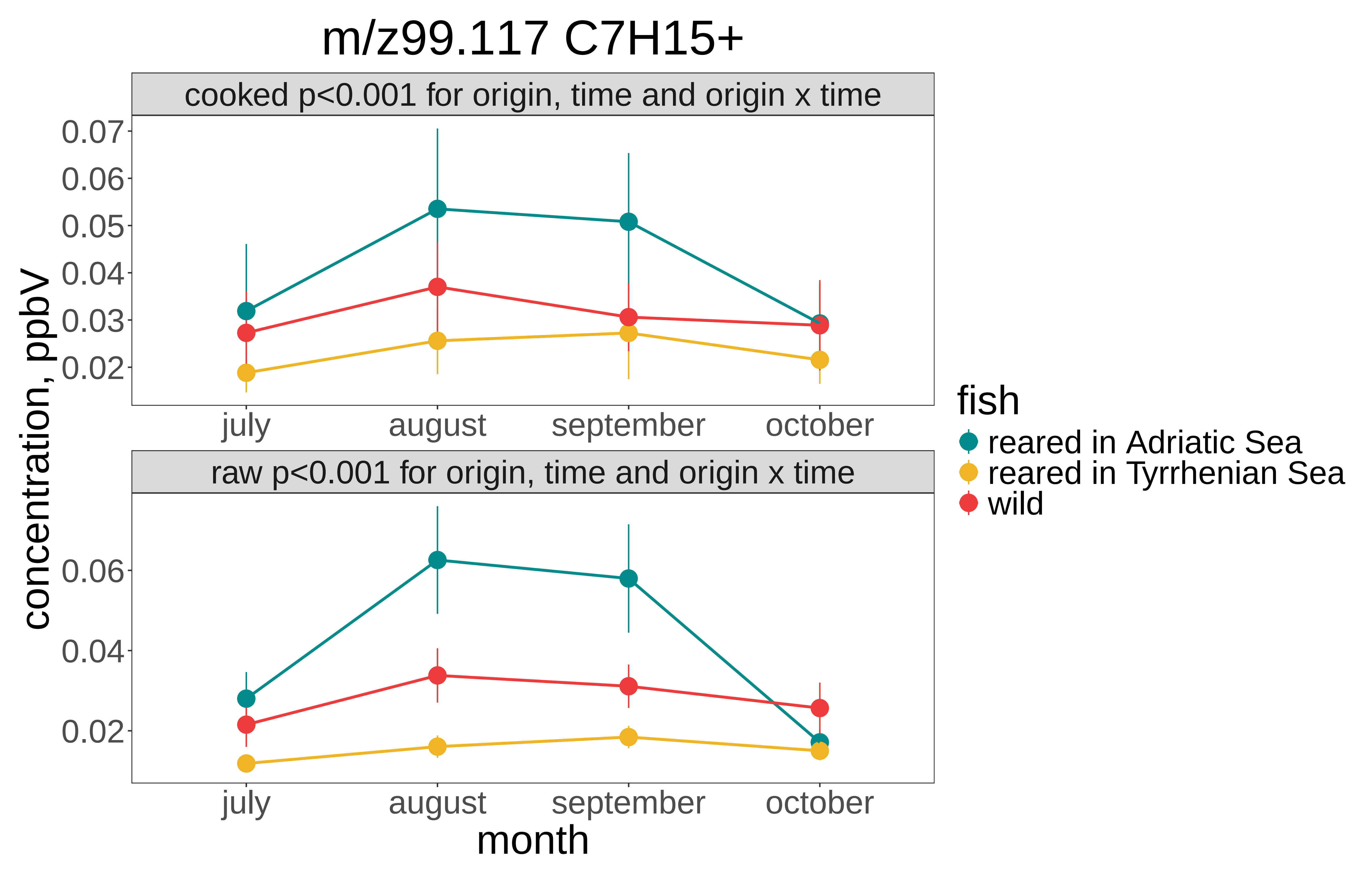

m/z99.117 C7H15+
cooked p<0.001 for origin, time and origin x time
raw p<0.001 for origin, time and origin x time
concentration, ppbV
0.07
0.06
0.05
0.04
0.03
0.02
0.06
0.04
0.02
july
august
september
october
july
august
september
october
month
fish
reared in Adriatic Sea
reared in Tyrrhenian Sea
wild

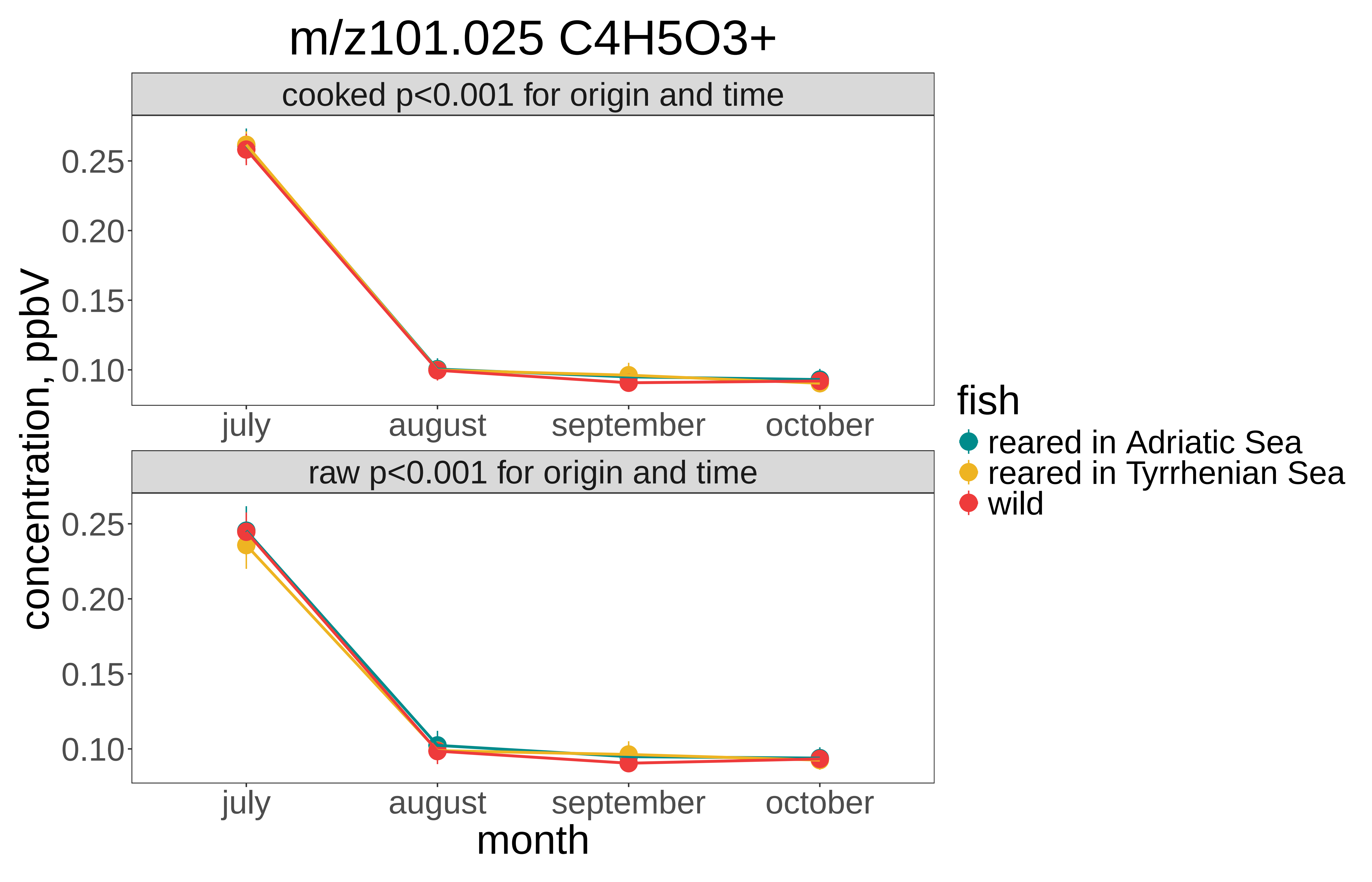

m/z101.025 C4H5O3+
cooked p<0.001 for origin and time
raw p<0.001 for origin and time
concentration, ppbV
0.25
0.20
0.15
0.10
july
august
september
october
month
fish
reared in Adriatic Sea
reared in Tyrrhenian Sea
wild

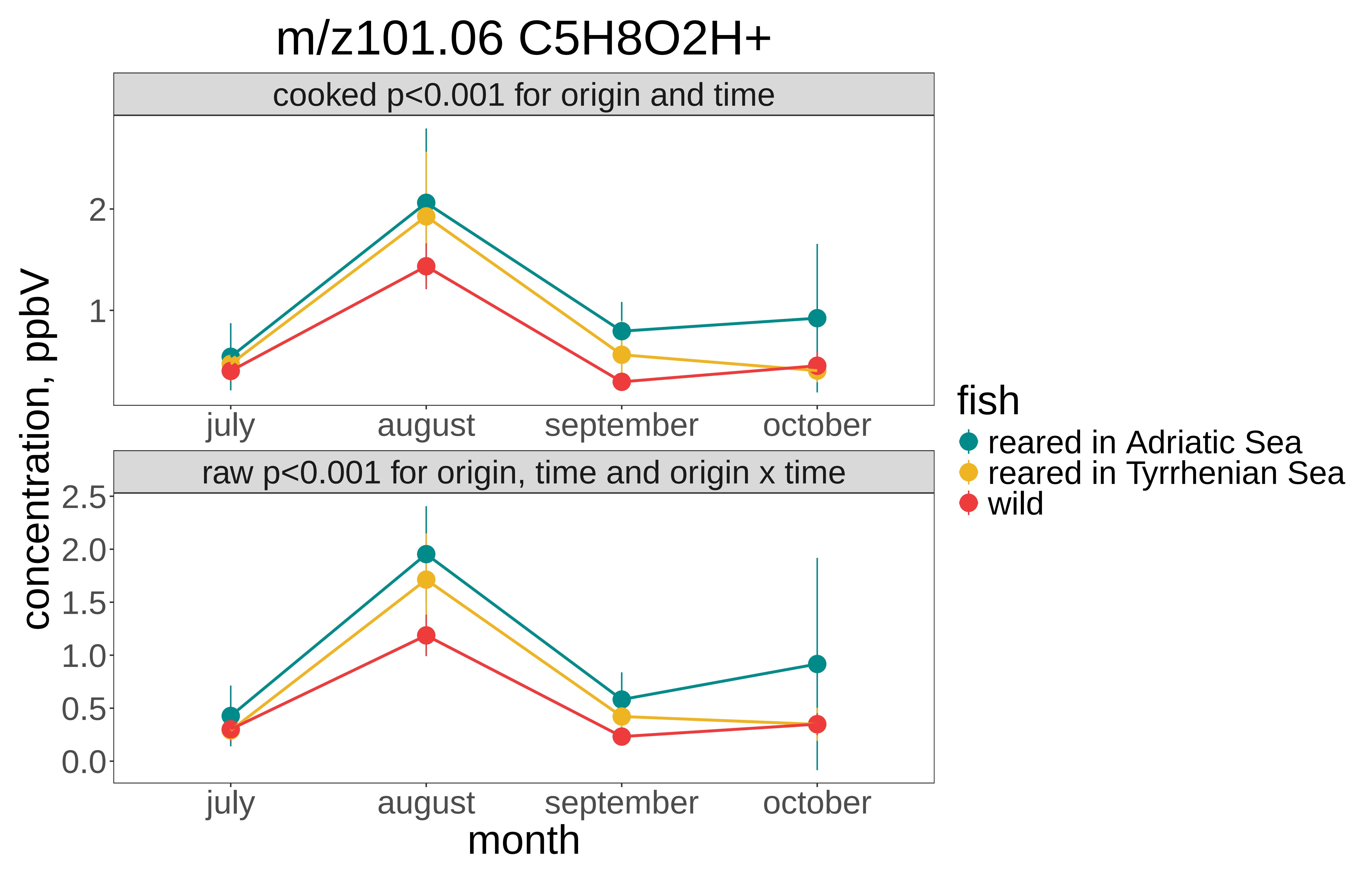
m/z101.06 C5H8O2H+
cooked p<0.001 for origin and time
raw p<0.001 for origin, time and origin x time
concentration, ppbV
2
1
2.5
2.0
1.5
1.0
0.5
0.0
july
august
september
october
month
fish
reared in Adriatic Sea
reared in Tyrrhenian Sea
wild

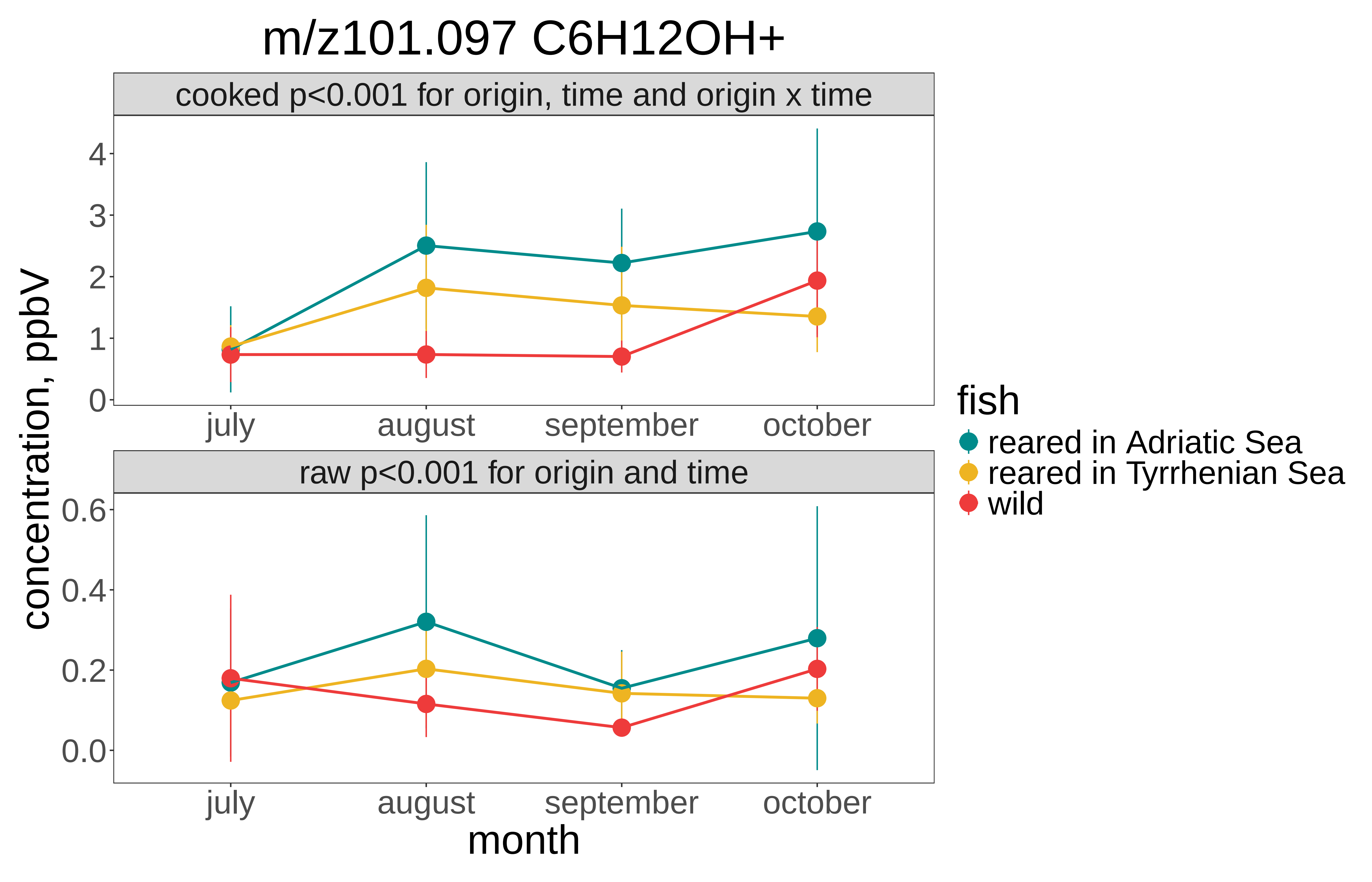

m/z101.097 C6H12OH+
cooked p<0.001 for origin, time and origin x time
raw p<0.001 for origin and time
concentration, ppbV
0
1
2
3
4
0.0
0.2
0.4
0.6
july
august
september
october
month
fish
reared in Adriatic Sea
reared in Tyrrhenian Sea
wild

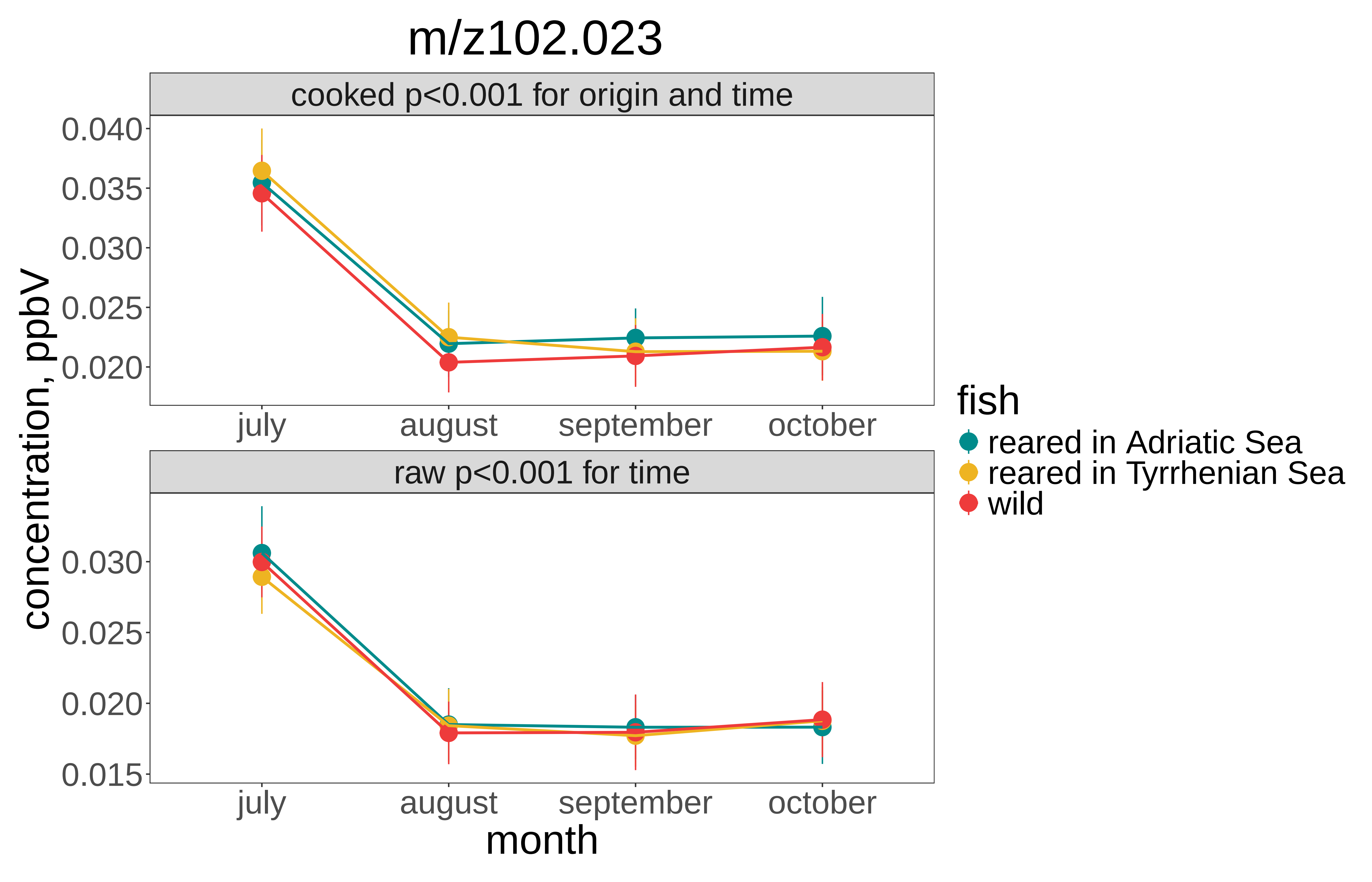

m/z102.023
cooked p<0.001 for origin and time
raw p<0.001 for time
0.040
0.035
0.030
0.025
0.020
0.015
july
august
september
october
concentration, ppbV
month
fish
reared in Adriatic Sea
reared in Tyrrhenian Sea
wild

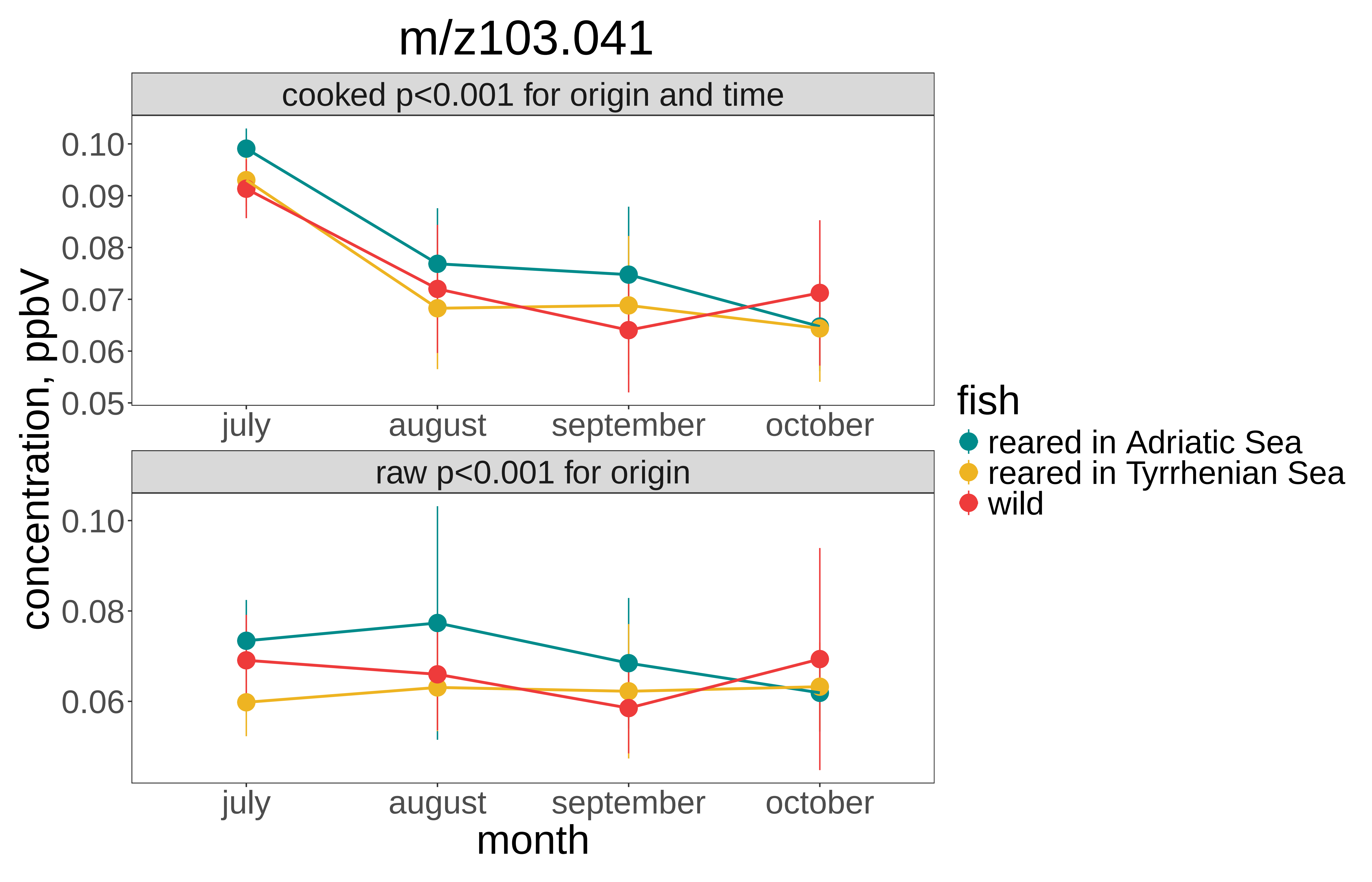

m/z103.041
cooked p<0.001 for origin and time
raw p<0.001 for origin
0.10
0.09
0.08
0.07
0.06
0.05
0.10
0.08
0.06
concentration, ppbV
july
august
september
october
july
august
september
october
month
fish
reared in Adriatic Sea
reared in Tyrrhenian Sea
wild

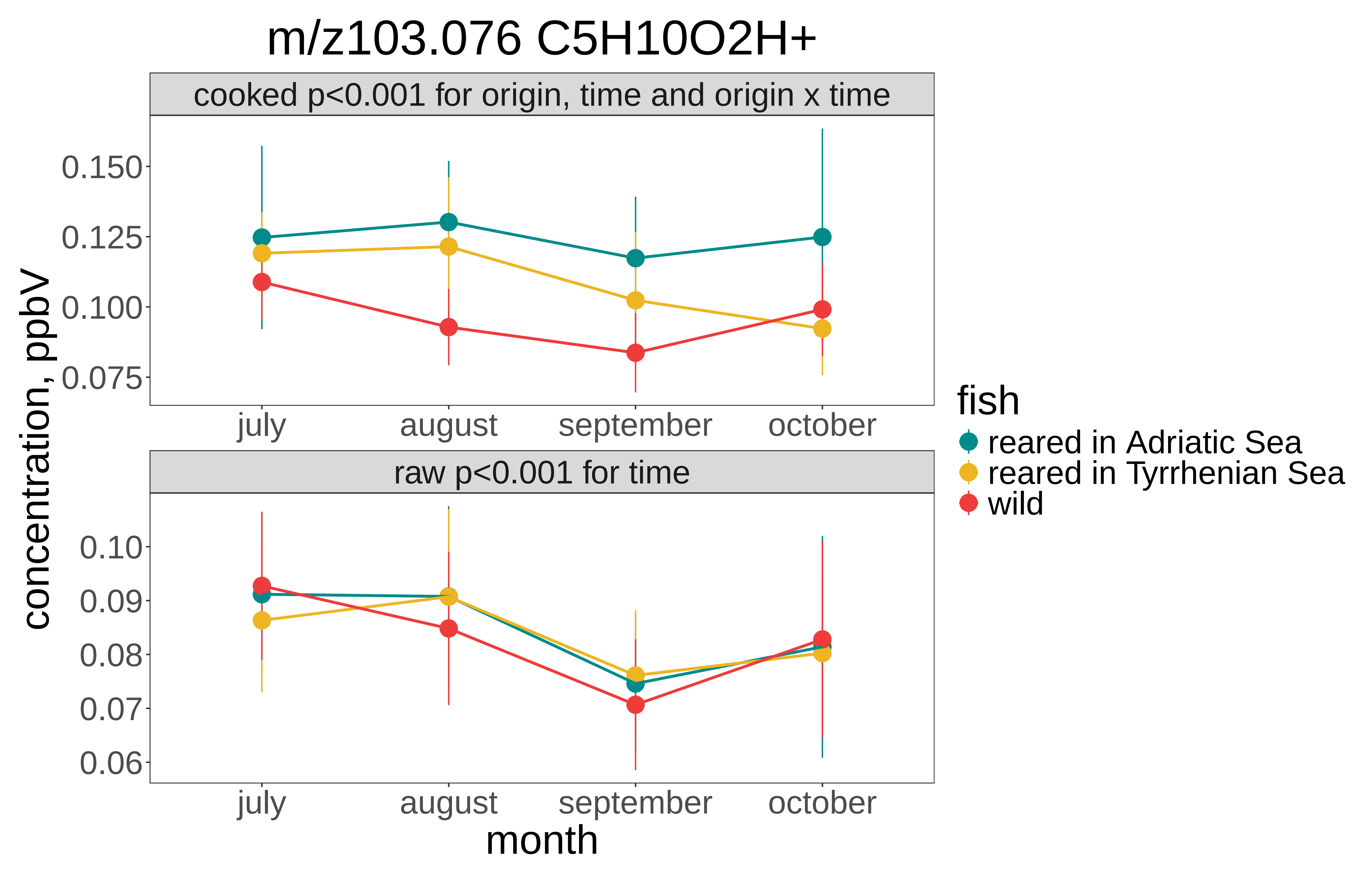
m/z103.076 C5H10O2H+
cooked p<0.001 for origin, time and origin x time
raw p<0.001 for time
concentration, ppbV
0.150
0.125
0.100
0.075
0.10
0.09
0.08
0.07
0.06
july
august
september
october
month
fish
reared in Adriatic Sea
reared in Tyrrhenian Sea
wild

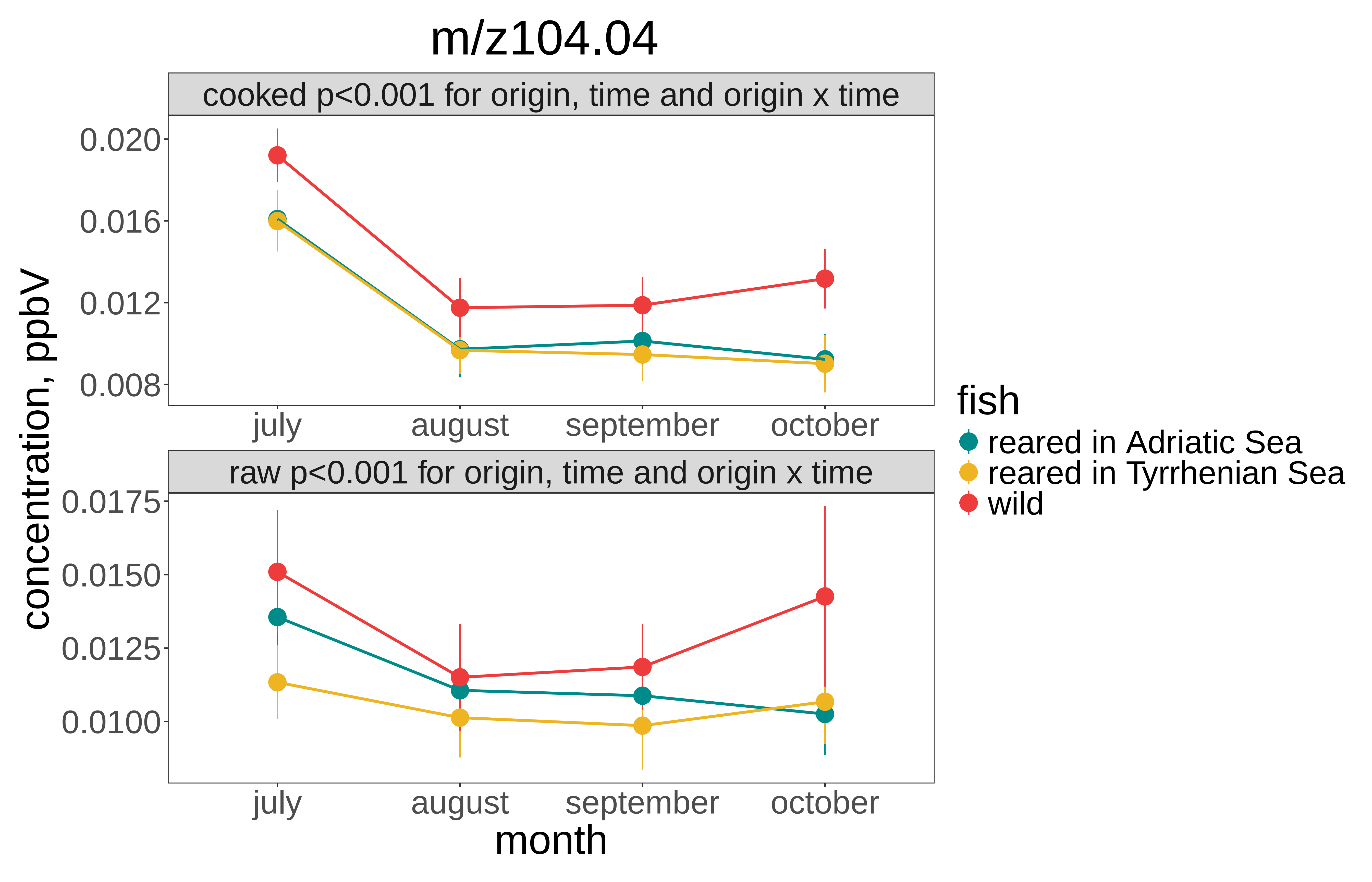

m/z104.04
cooked p<0.001 for origin, time and origin x time
0.020
0.016
0.012
0.008
july
august
september
october
raw p<0.001 for origin, time and origin x time
0.0175
0.0150
0.0125
0.0100
july
august
september
october
concentration, ppbV
month
fish
reared in Adriatic Sea
reared in Tyrrhenian Sea
wild

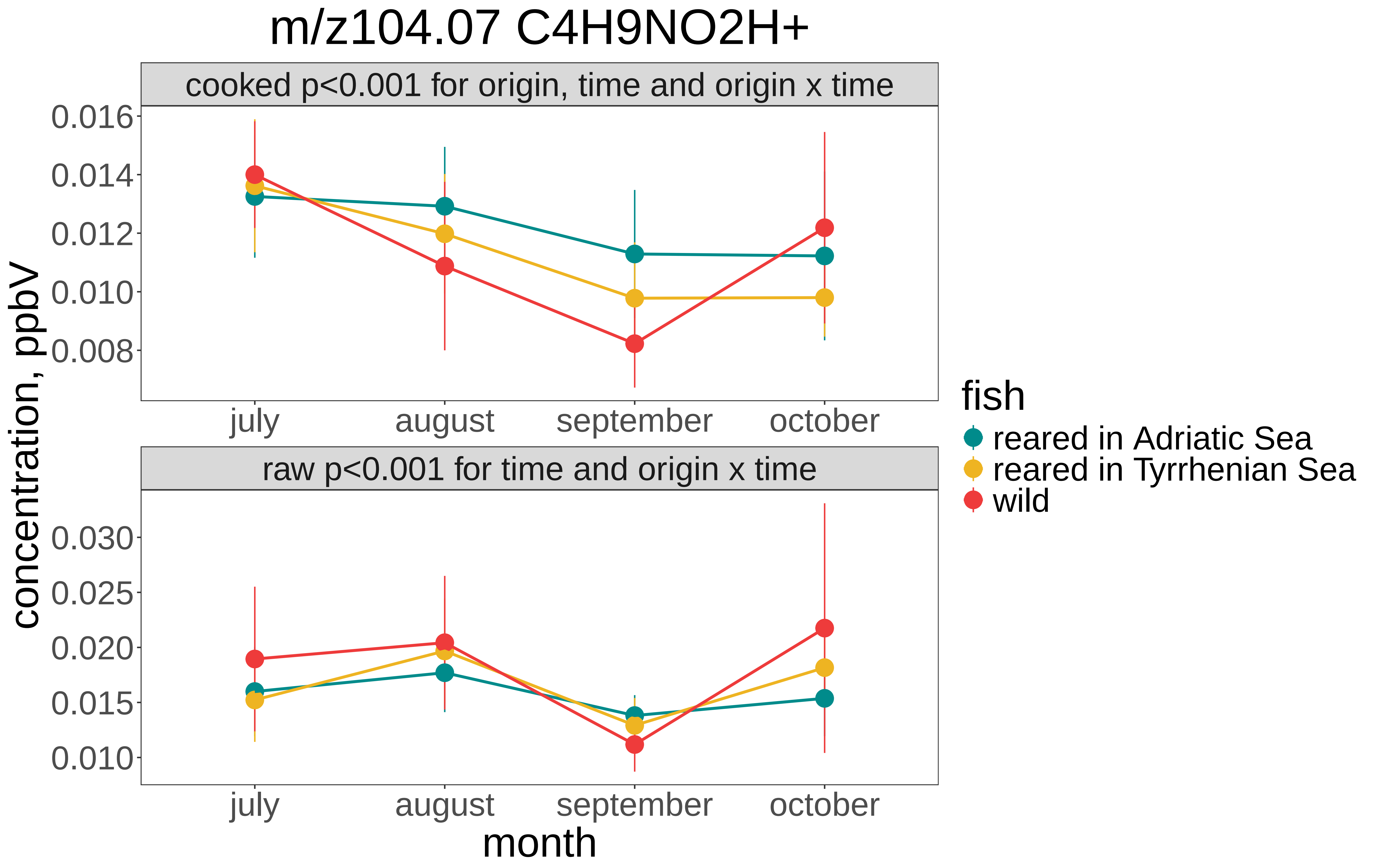
m/z104.07 C4H9NO2H+
cooked p<0.001 for origin, time and origin x time
raw p<0.001 for time and origin x time
concentration, ppbV
0.016
0.014
0.012
0.010
0.008
0.030
0.025
0.020
0.015
0.010
july
august
september
october
month
fish
reared in Adriatic Sea
reared in Tyrrhenian Sea
wild

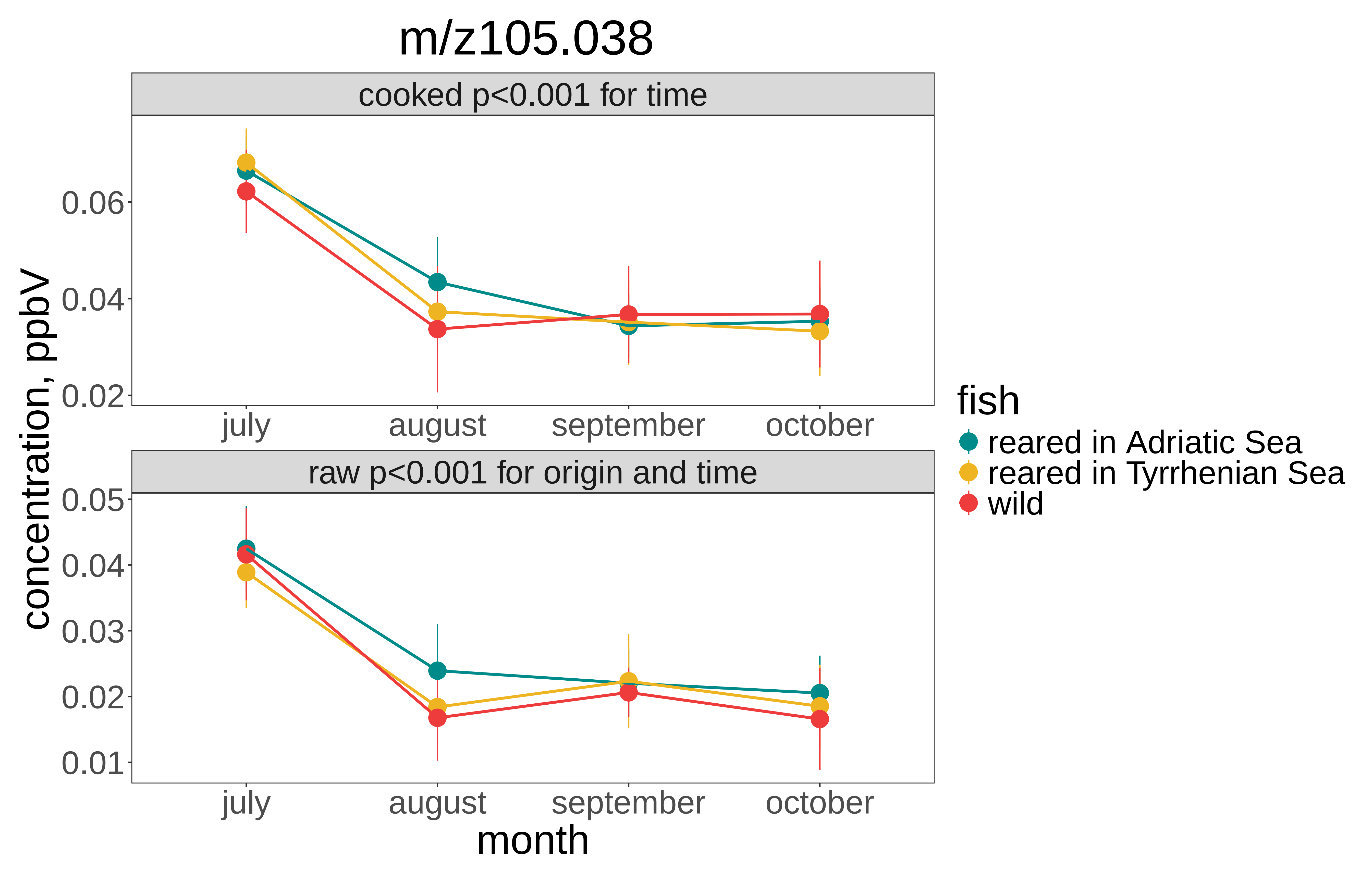
m/z105.038
cooked p<0.001 for time
raw p<0.001 for origin and time
concentration, ppbV
0.06
0.04
0.02
0.05
0.04
0.03
0.02
0.01
july
august
september
october
month
fish
reared in Adriatic Sea
reared in Tyrrhenian Sea
wild

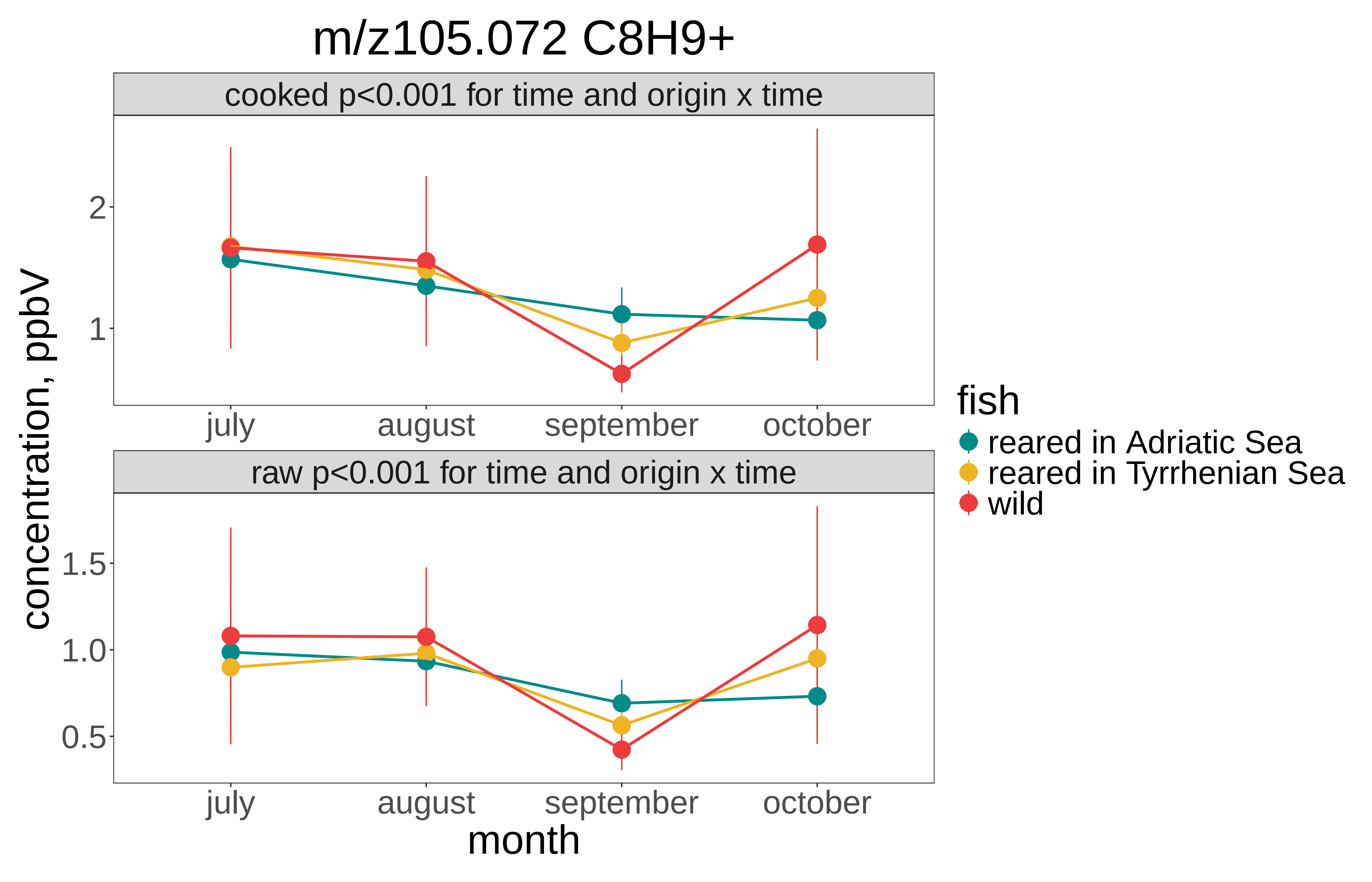
m/z105.072 C8H9+
cooked p<0.001 for time and origin x time
raw p<0.001 for time and origin x time
concentration, ppbV
2
1
1.5
1.0
0.5
july
august
september
october
month
fish
reared in Adriatic Sea
reared in Tyrrhenian Sea
wild

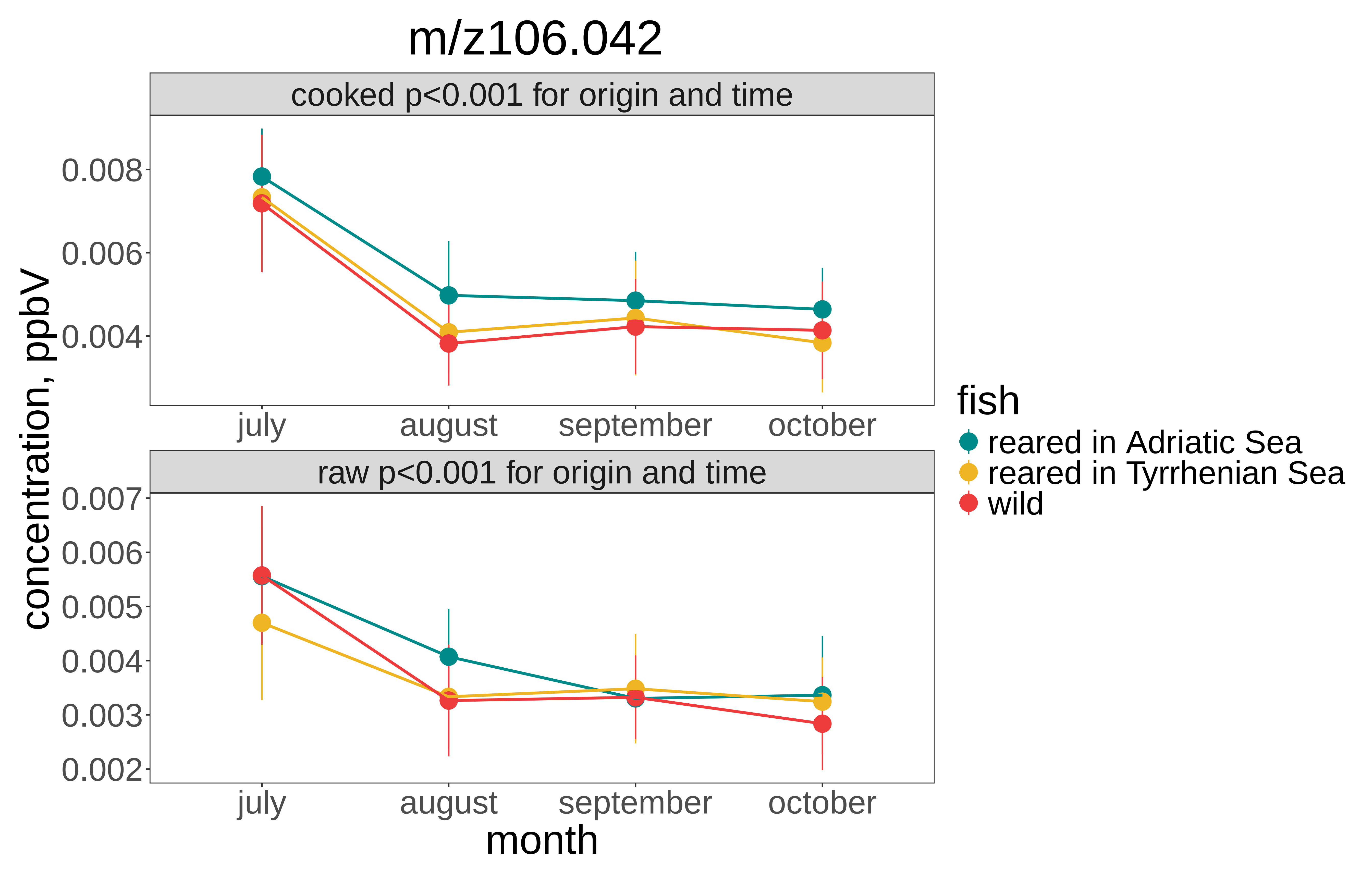
m/z106.042
cooked p<0.001 for origin and time
raw p<0.001 for origin and time
concentration, ppbV
0.008
0.006
0.004
0.007
0.006
0.005
0.004
0.003
0.002
july
august
september
october
month
fish
reared in Adriatic Sea
reared in Tyrrhenian Sea
wild

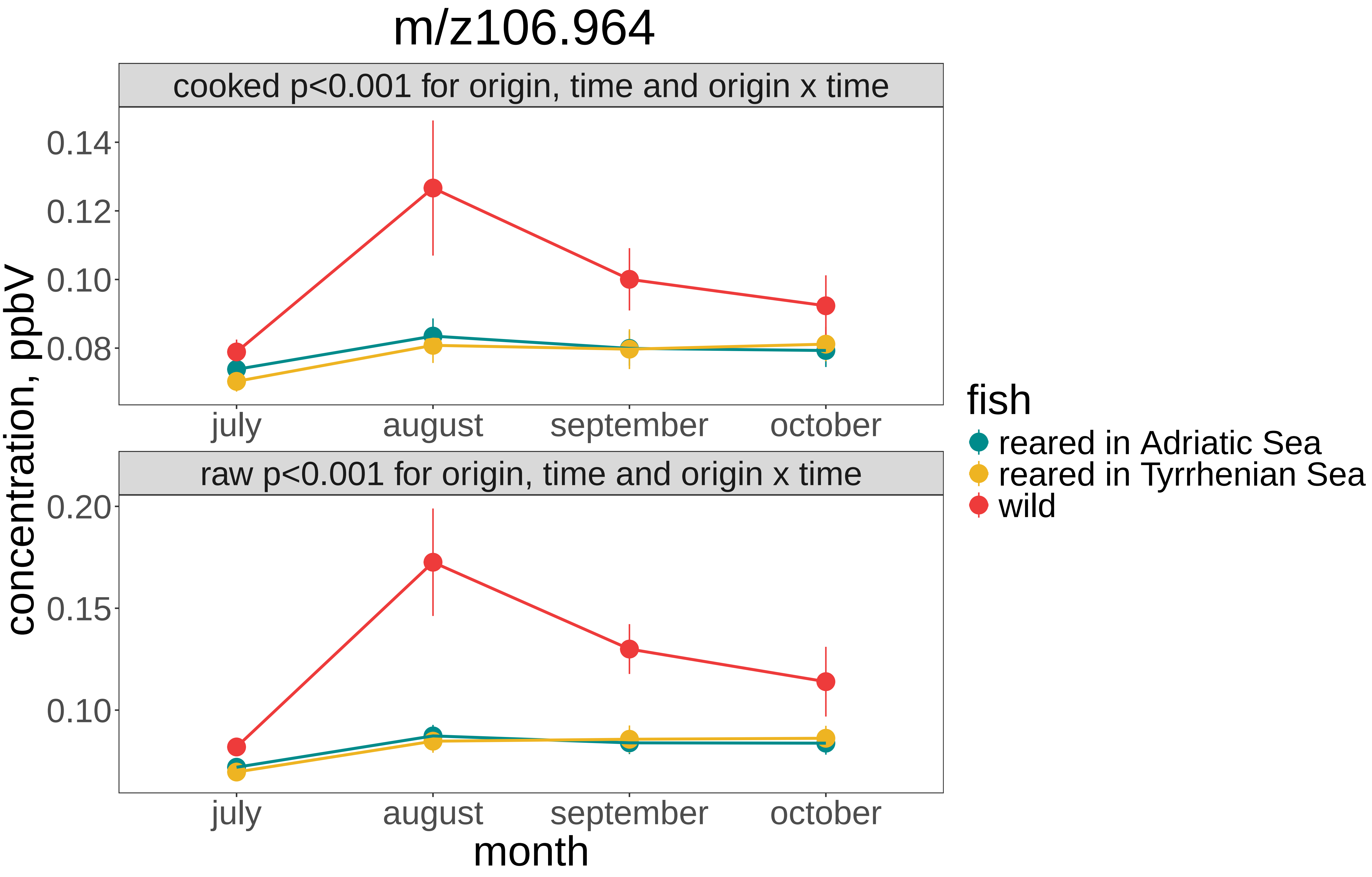
m/z106.964
cooked p<0.001 for origin, time and origin x time
raw p<0.001 for origin, time and origin x time
0.14
0.12
0.10
0.08
0.20
0.15
0.10
july
august
september
october
concentration, ppbV
month
fish
reared in Adriatic Sea
reared in Tyrrhenian Sea
wild

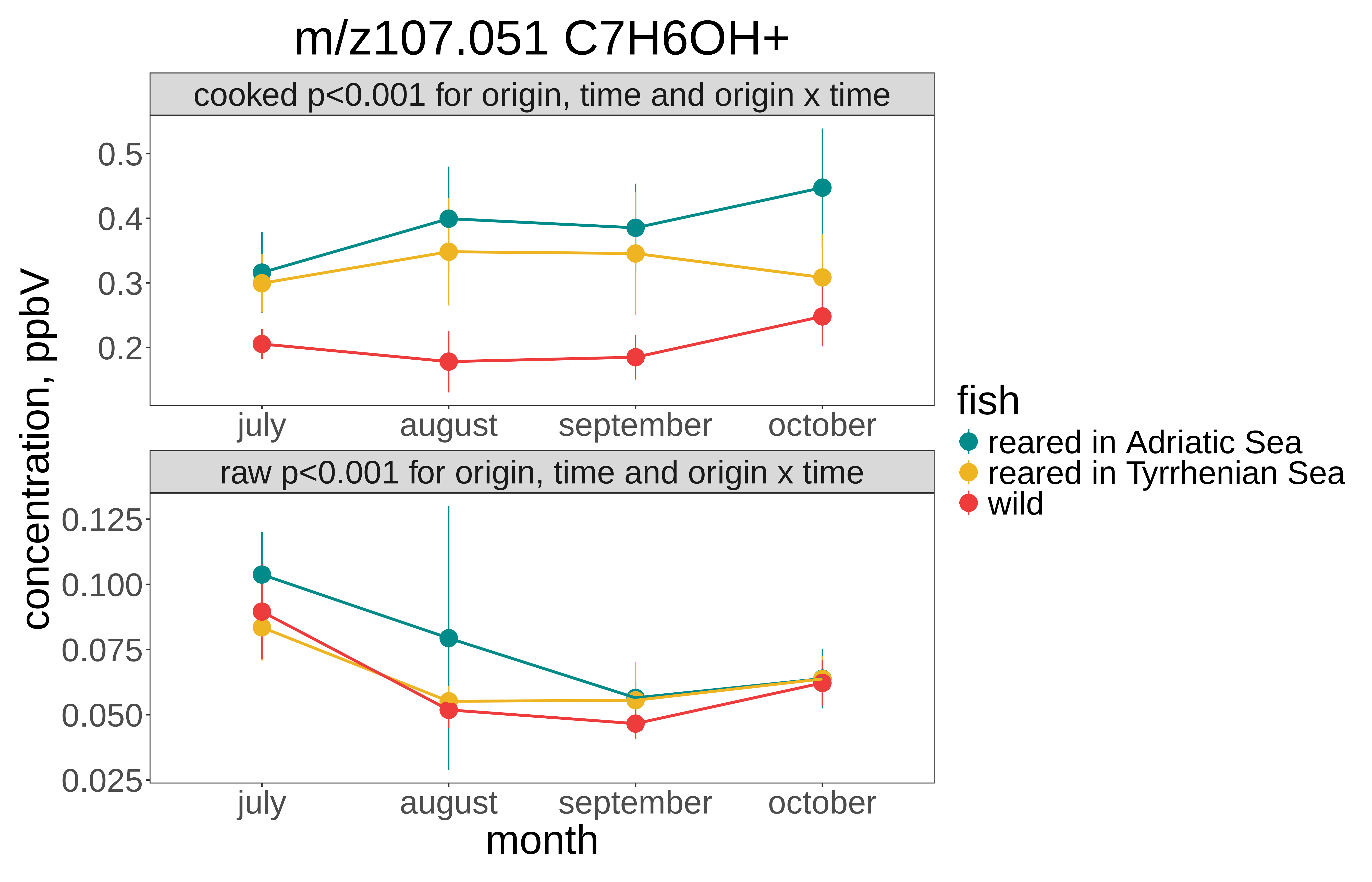

m/z107.051 C7H6OH+
cooked p<0.001 for origin, time and origin x time
raw p<0.001 for origin, time and origin x time
concentration, ppbV
0.5
0.4
0.3
0.2
0.125
0.100
0.075
0.050
0.025
july
august
september
october
month
fish
reared in Adriatic Sea
reared in Tyrrhenian Sea
wild

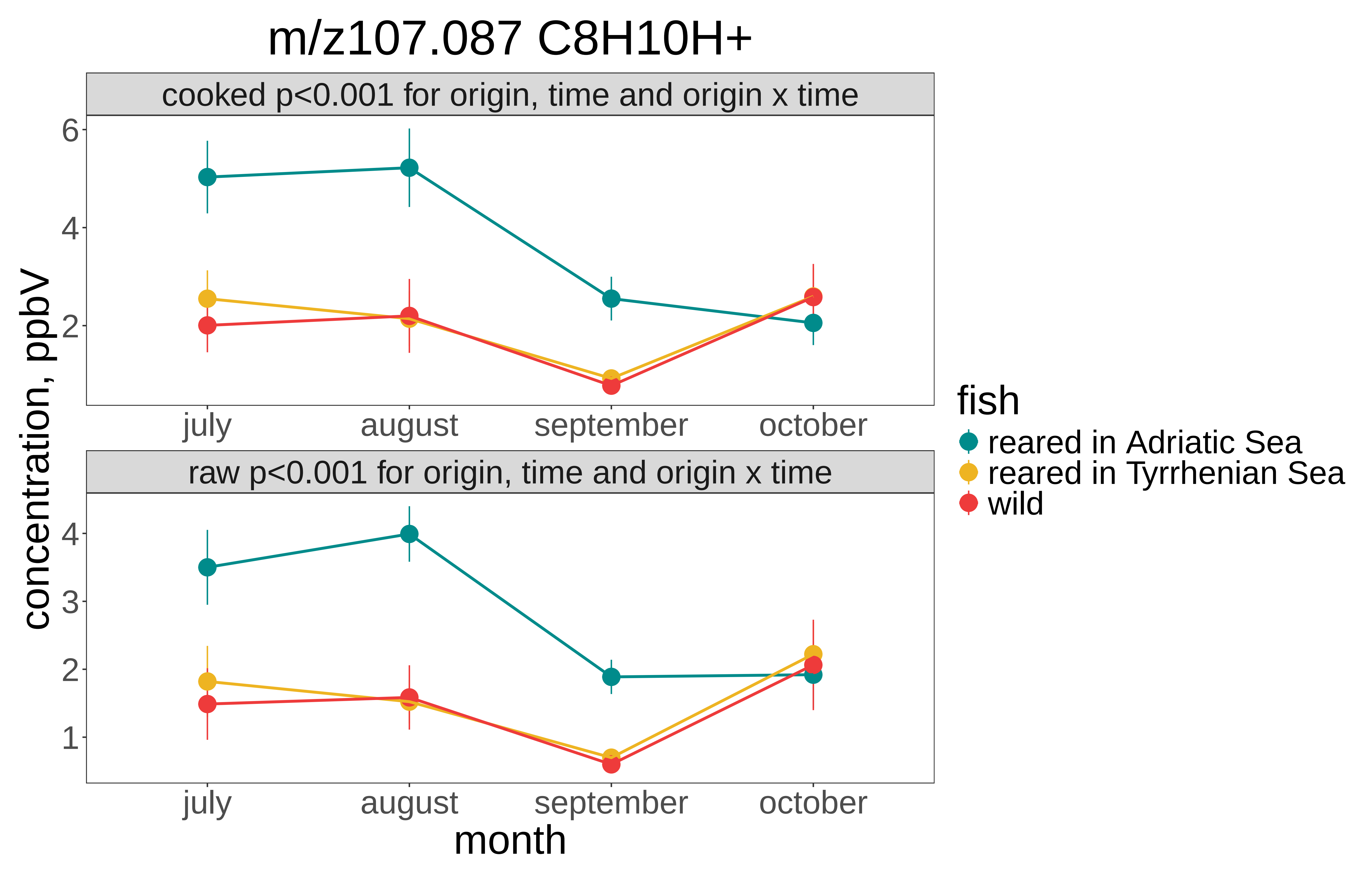
m/z107.087 C8H10H+
cooked p<0.001 for origin, time and origin x time
raw p<0.001 for origin, time and origin x time
concentration, ppbV
6
4
2
4
3
2
1
july
august
september
october
july
august
september
october
month
fish
reared in Adriatic Sea
reared in Tyrrhenian Sea
wild

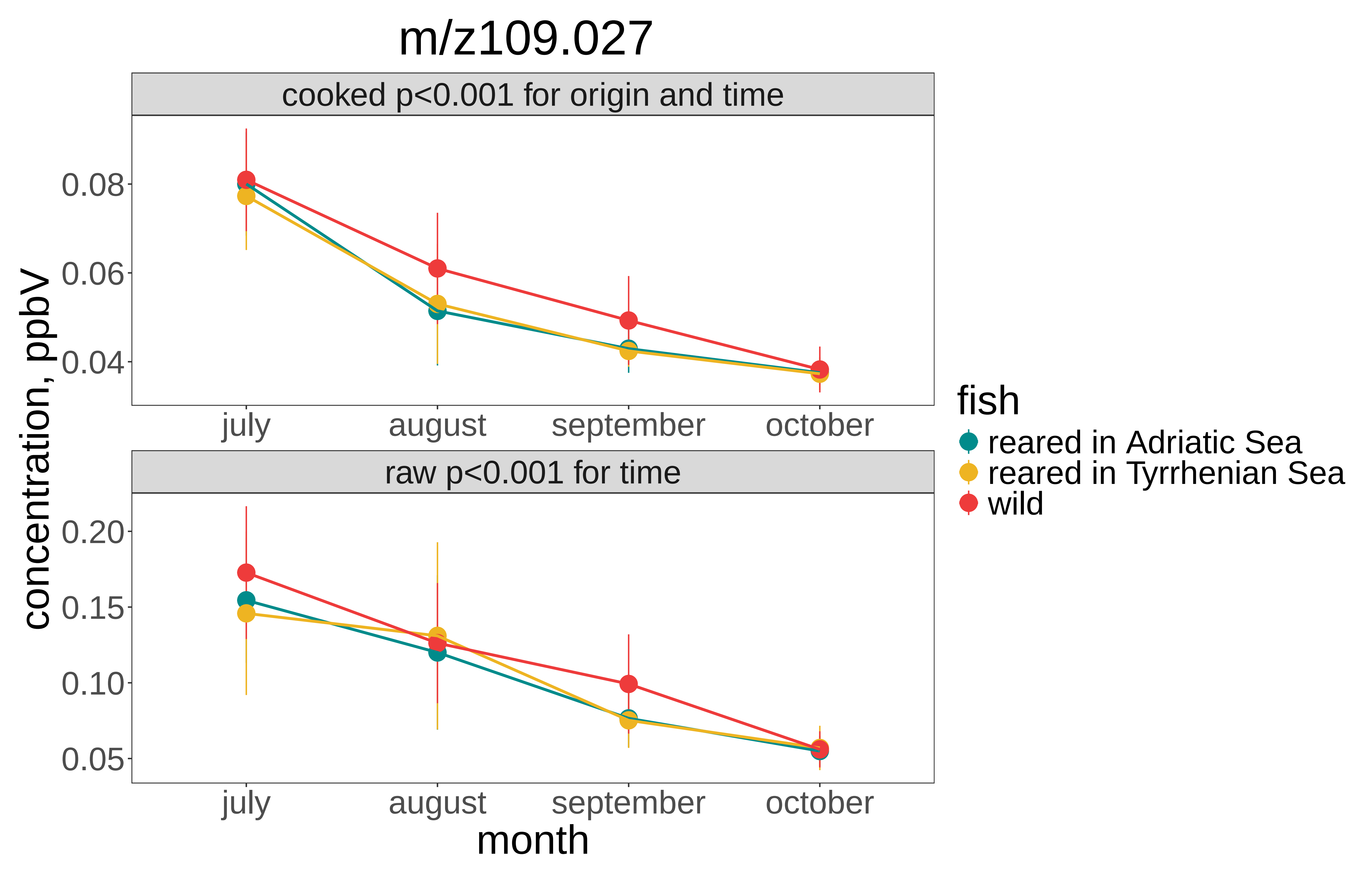
m/z109.027
cooked p<0.001 for origin and time
raw p<0.001 for time
concentration, ppbV
0.08
0.06
0.04
0.20
0.15
0.10
0.05
july
august
september
october
month
fish
reared in Adriatic Sea
reared in Tyrrhenian Sea
wild

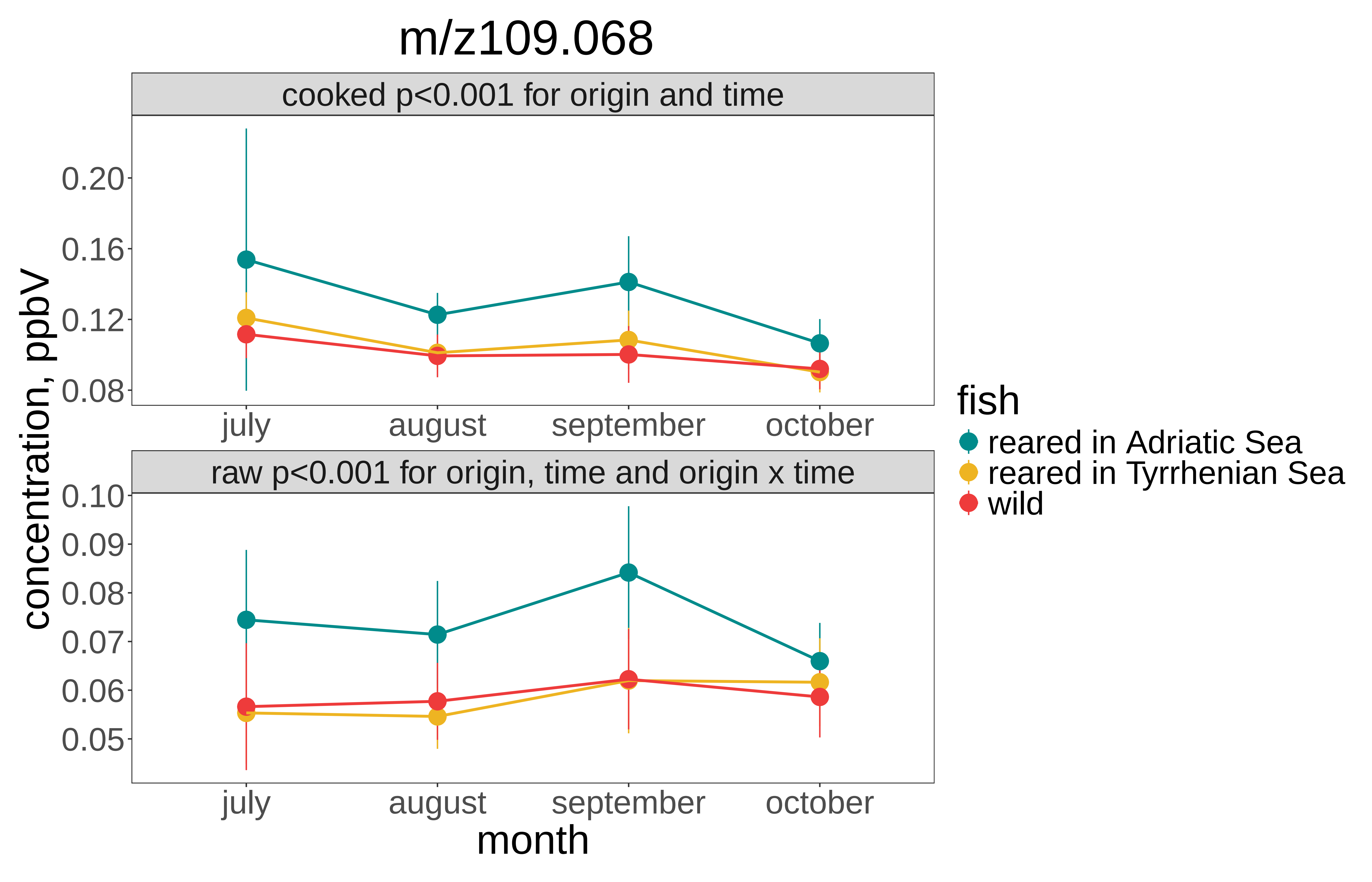
m/z109.068
cooked p<0.001 for origin and time
raw p<0.001 for origin, time and origin x time
concentration, ppbV
0.20
0.16
0.12
0.08
0.10
0.09
0.08
0.07
0.06
0.05
july
august
september
october
month
fish
reared in Adriatic Sea
reared in Tyrrhenian Sea
wild

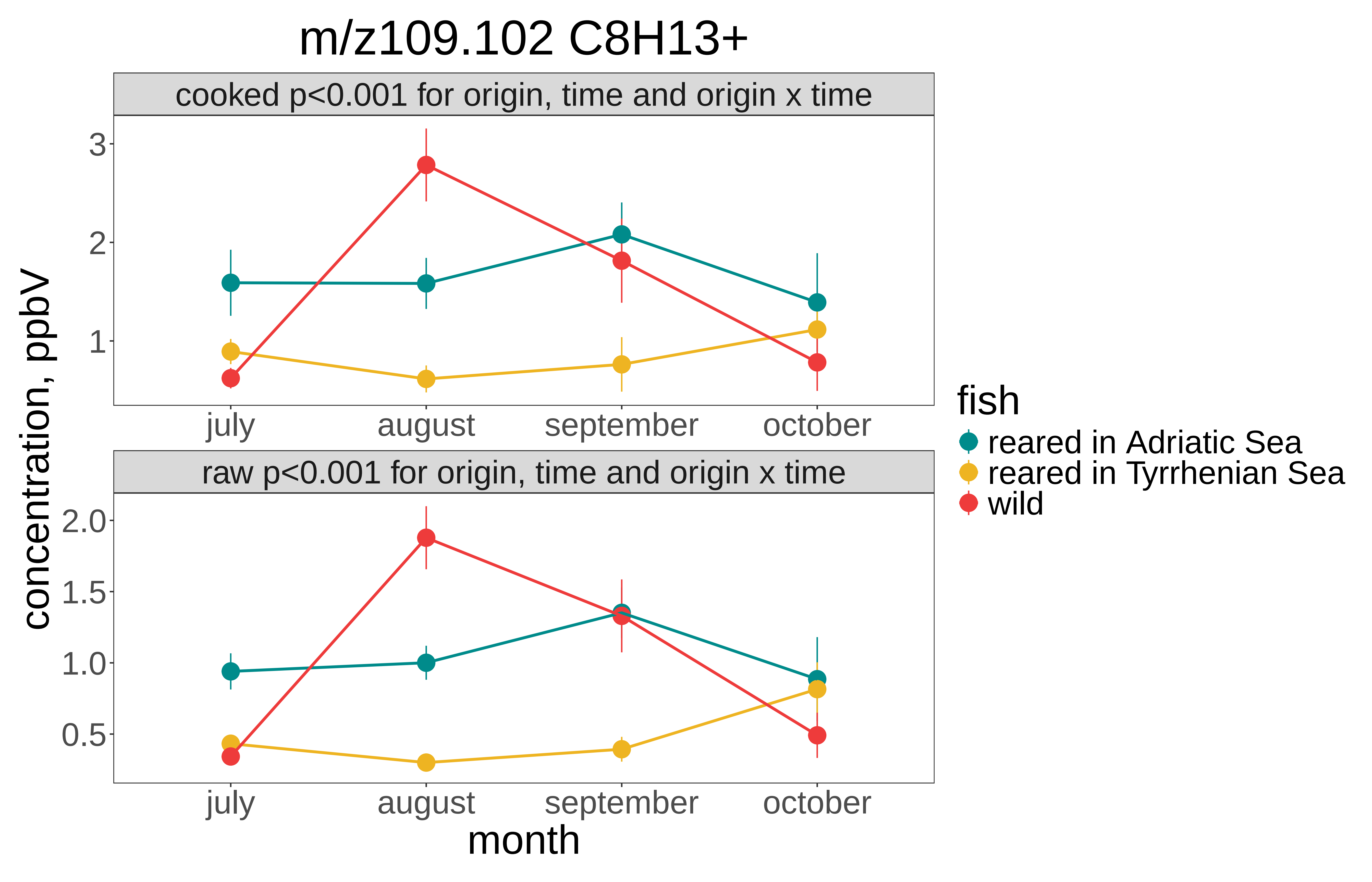
m/z109.102 C8H13+
cooked p<0.001 for origin, time and origin x time
raw p<0.001 for origin, time and origin x time
concentration, ppbV
3
2
1
2.0
1.5
1.0
0.5
july
august
september
october
month
fish
reared in Adriatic Sea
reared in Tyrrhenian Sea
wild

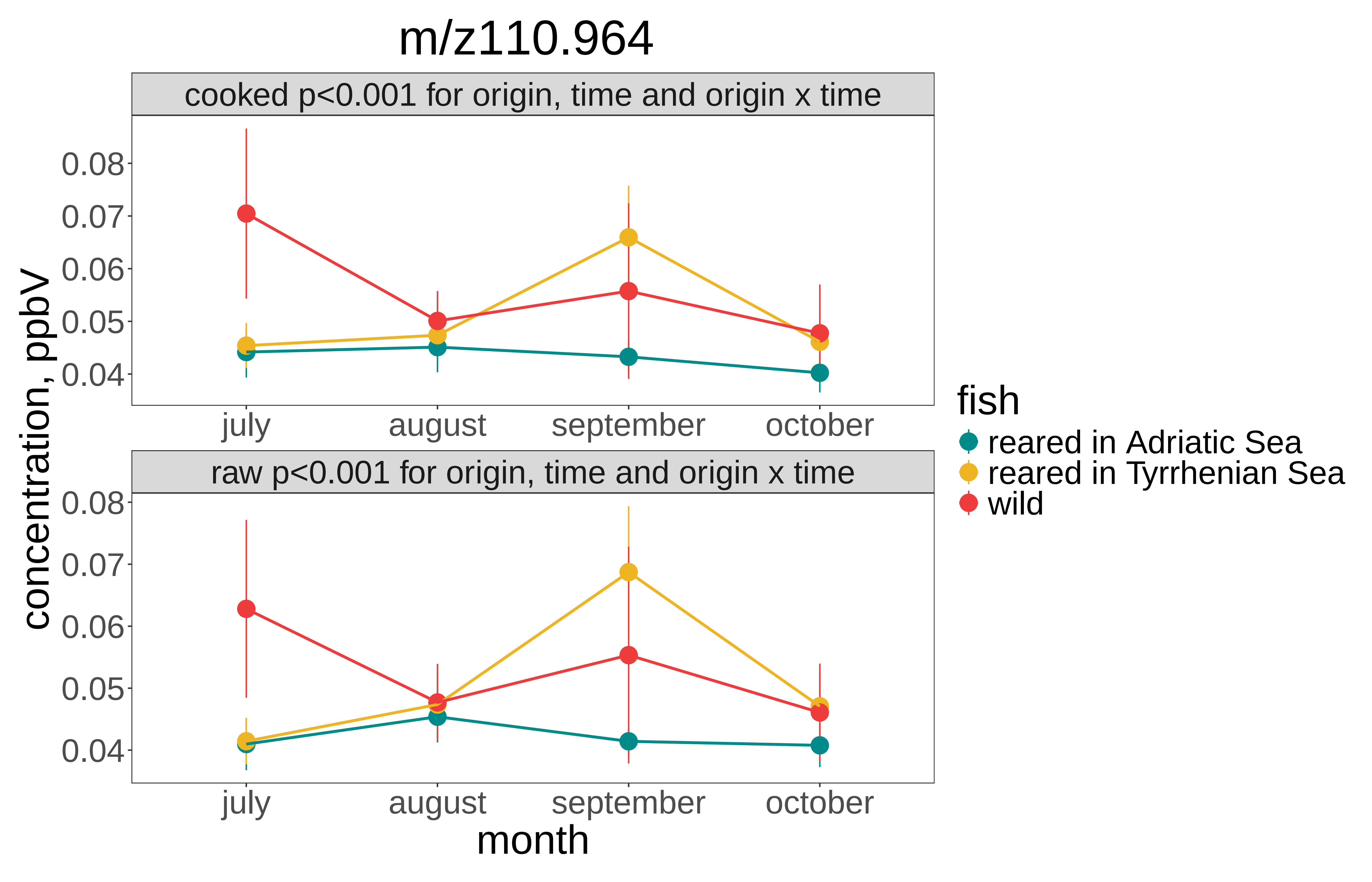

m/z110.964
cooked p<0.001 for origin, time and origin x time
raw p<0.001 for origin, time and origin x time
0.08
0.07
0.06
0.05
0.04
july
august
september
october
concentration, ppbV
month
fish
reared in Adriatic Sea
reared in Tyrrhenian Sea
wild

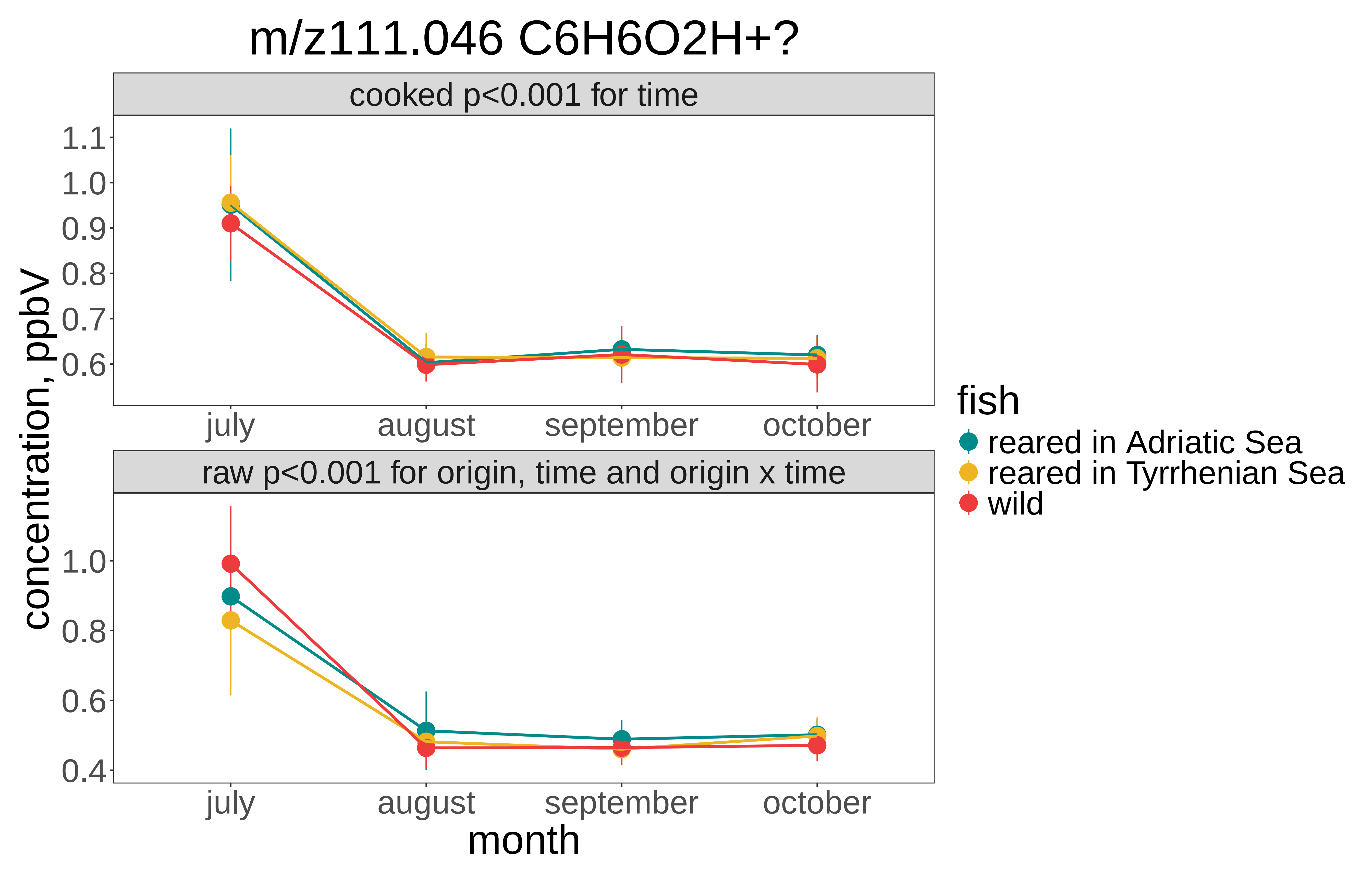

m/z111.046 C6H6O2H+?
cooked p<0.001 for time
raw p<0.001 for origin, time and origin x time
concentration, ppbV
1.1
1.0
0.9
0.8
0.7
0.6
0.4
july
august
september
october
month
fish
reared in Adriatic Sea
reared in Tyrrhenian Sea
wild

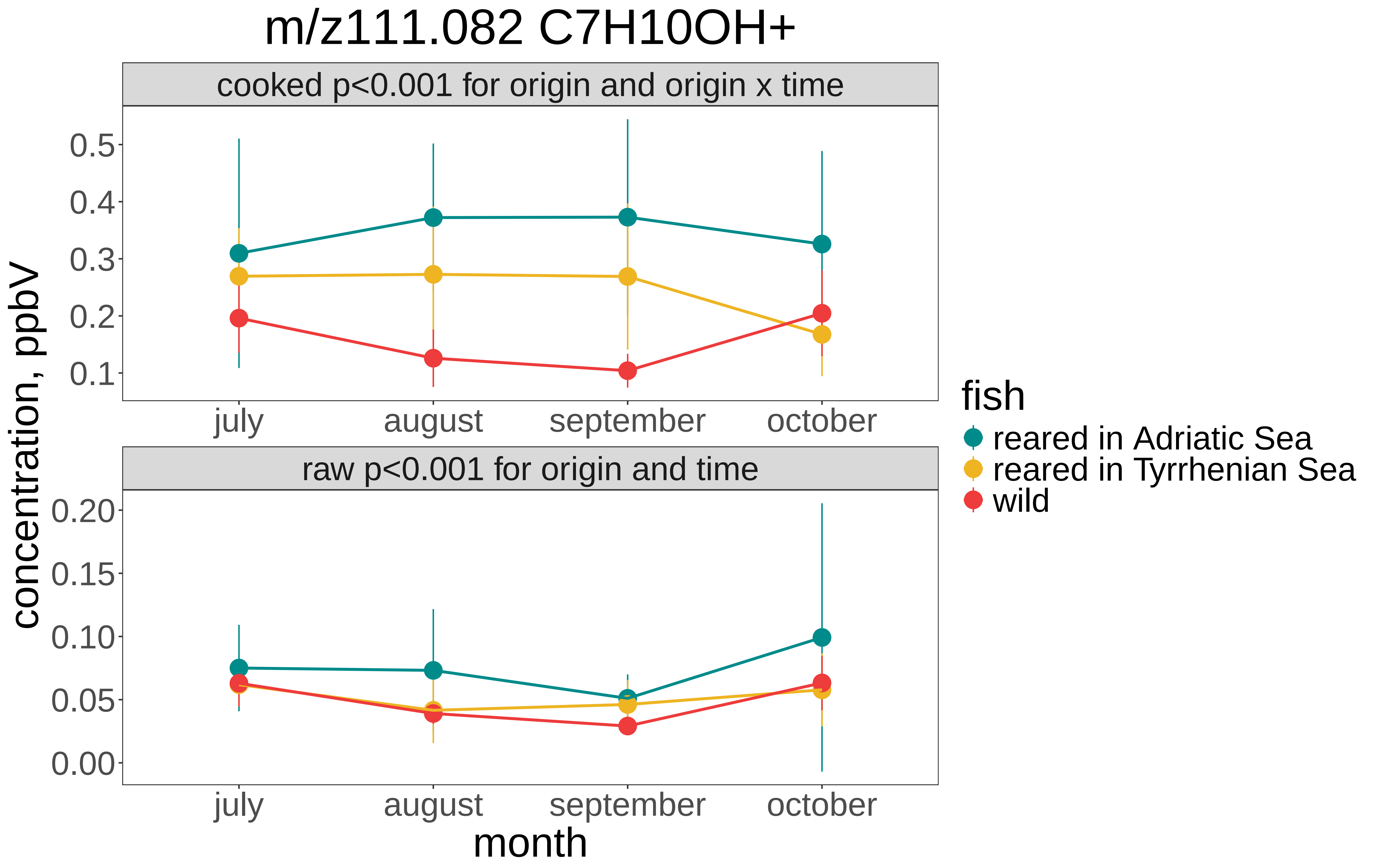
m/z111.082 C7H10OH+
cooked p<0.001 for origin and origin x time
raw p<0.001 for origin and time
concentration, ppbV
0.5
0.4
0.3
0.2
0.1
0.20
0.15
0.10
0.05
0.00
july
august
september
october
month
fish
reared in Adriatic Sea
reared in Tyrrhenian Sea
wild

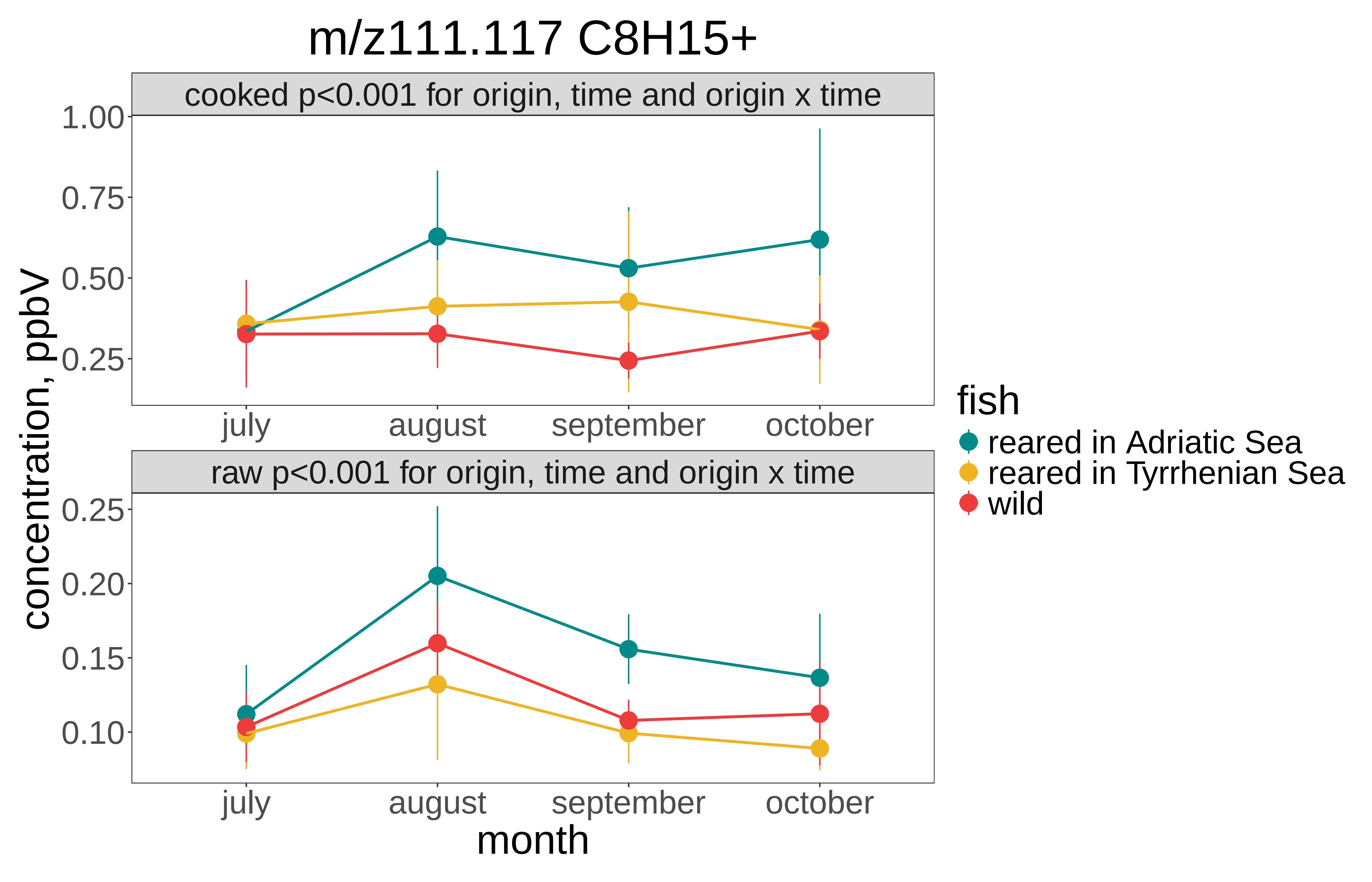

m/z111.117 C8H15+
cooked p<0.001 for origin, time and origin x time
raw p<0.001 for origin, time and origin x time
concentration, ppbV
1.00
0.75
0.50
0.25
0.20
0.15
0.10
july
august
september
october
month
fish
reared in Adriatic Sea
reared in Tyrrhenian Sea
wild

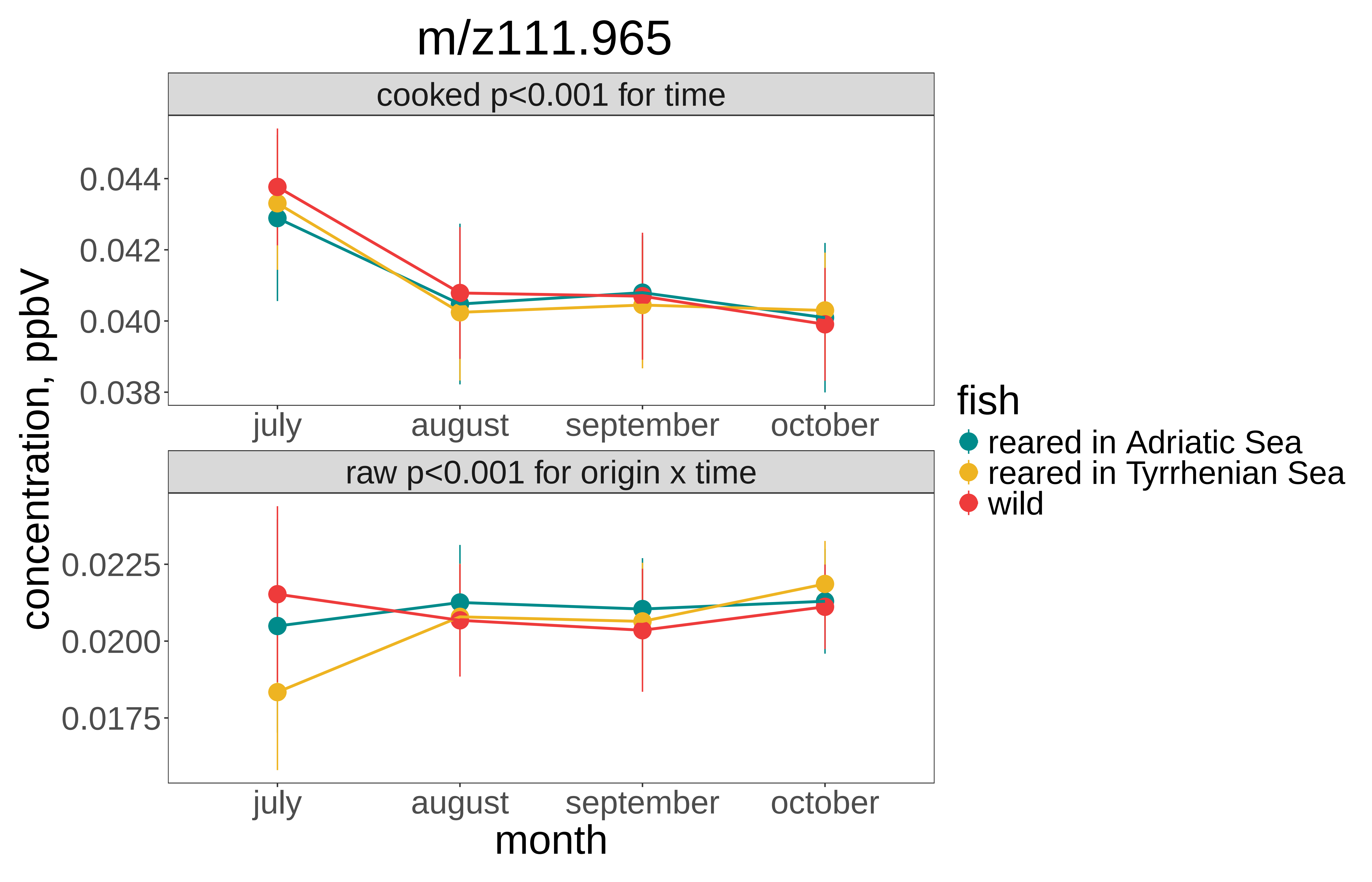

m/z111.965
cooked p<0.001 for time
raw p<0.001 for origin x time
0.044
0.042
0.040
0.038
0.0225
0.0200
0.0175
july
august
september
october
concentration, ppbV
month
fish
reared in Adriatic Sea
reared in Tyrrhenian Sea
wild

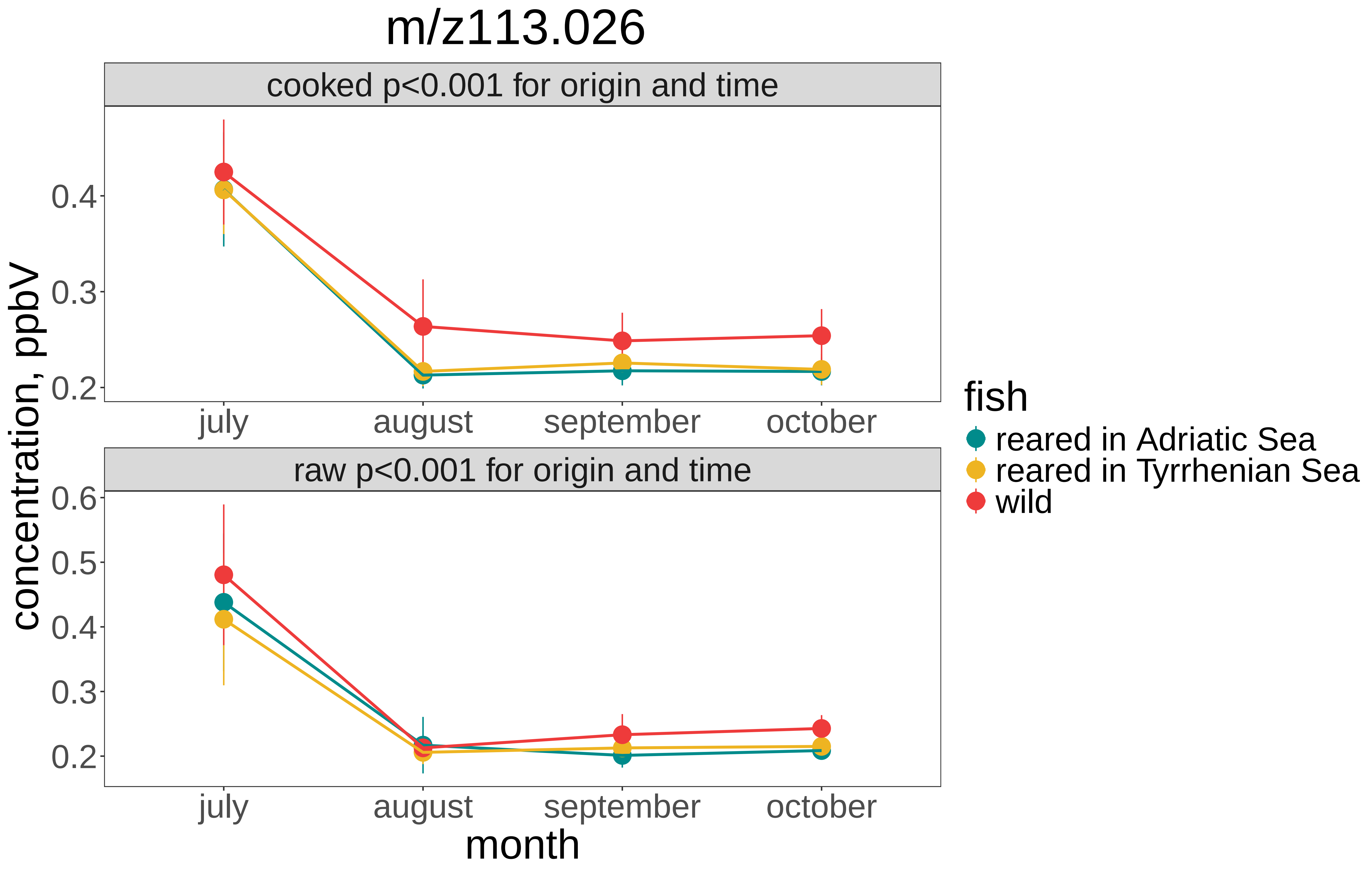

m/z113.026
cooked p<0.001 for origin and time
raw p<0.001 for origin and time
concentration, ppbV
0.4
0.3
0.2
0.6
0.5
0.4
0.3
0.2
july
august
september
october
july
august
september
october
month
fish
reared in Adriatic Sea
reared in Tyrrhenian Sea
wild

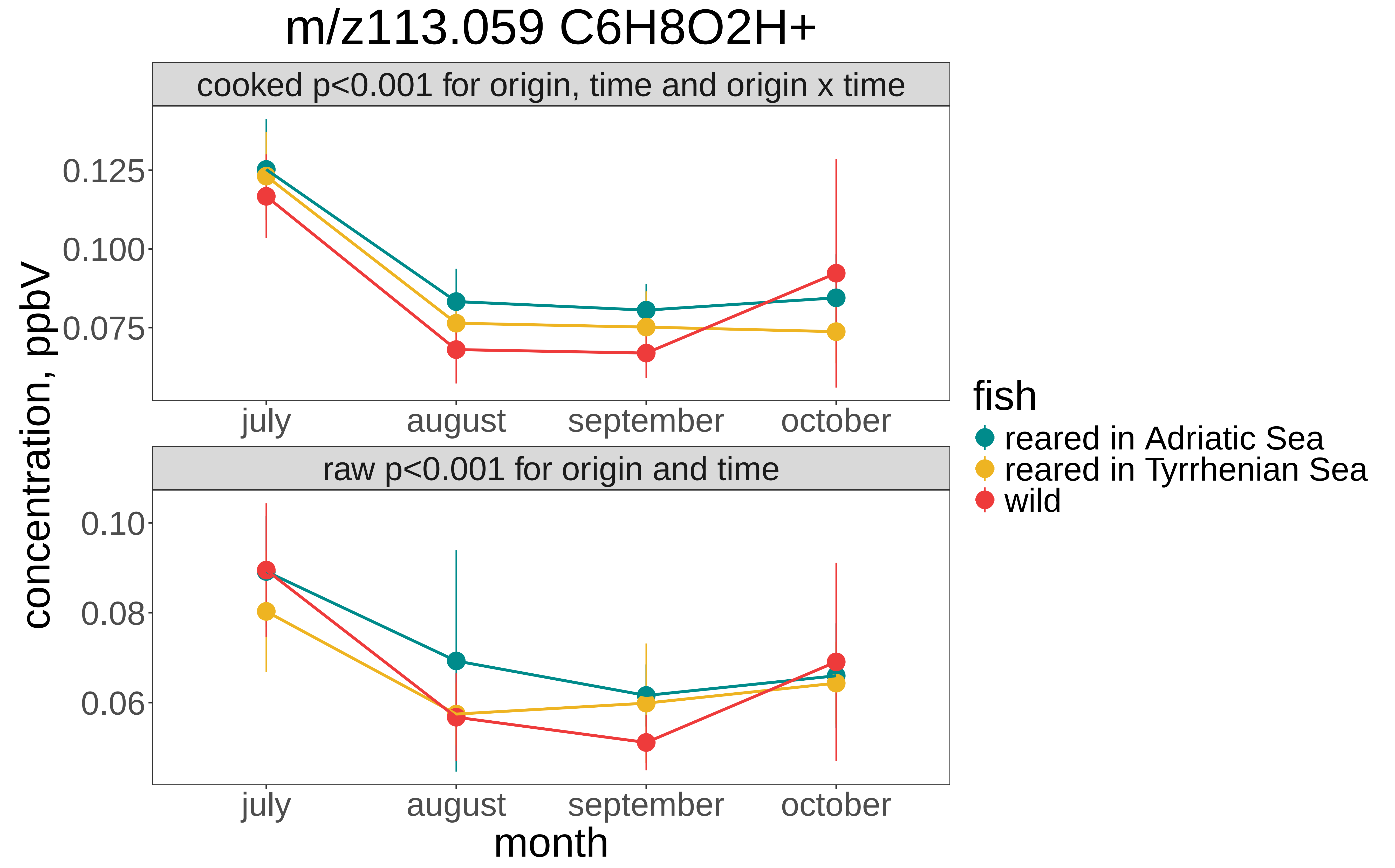
m/z113.059 C6H8O2H+
cooked p<0.001 for origin, time and origin x time
raw p<0.001 for origin and time
concentration, ppbV
0.125
0.100
0.075
0.10
0.08
0.06
july
august
september
october
month
fish
reared in Adriatic Sea
reared in Tyrrhenian Sea
wild

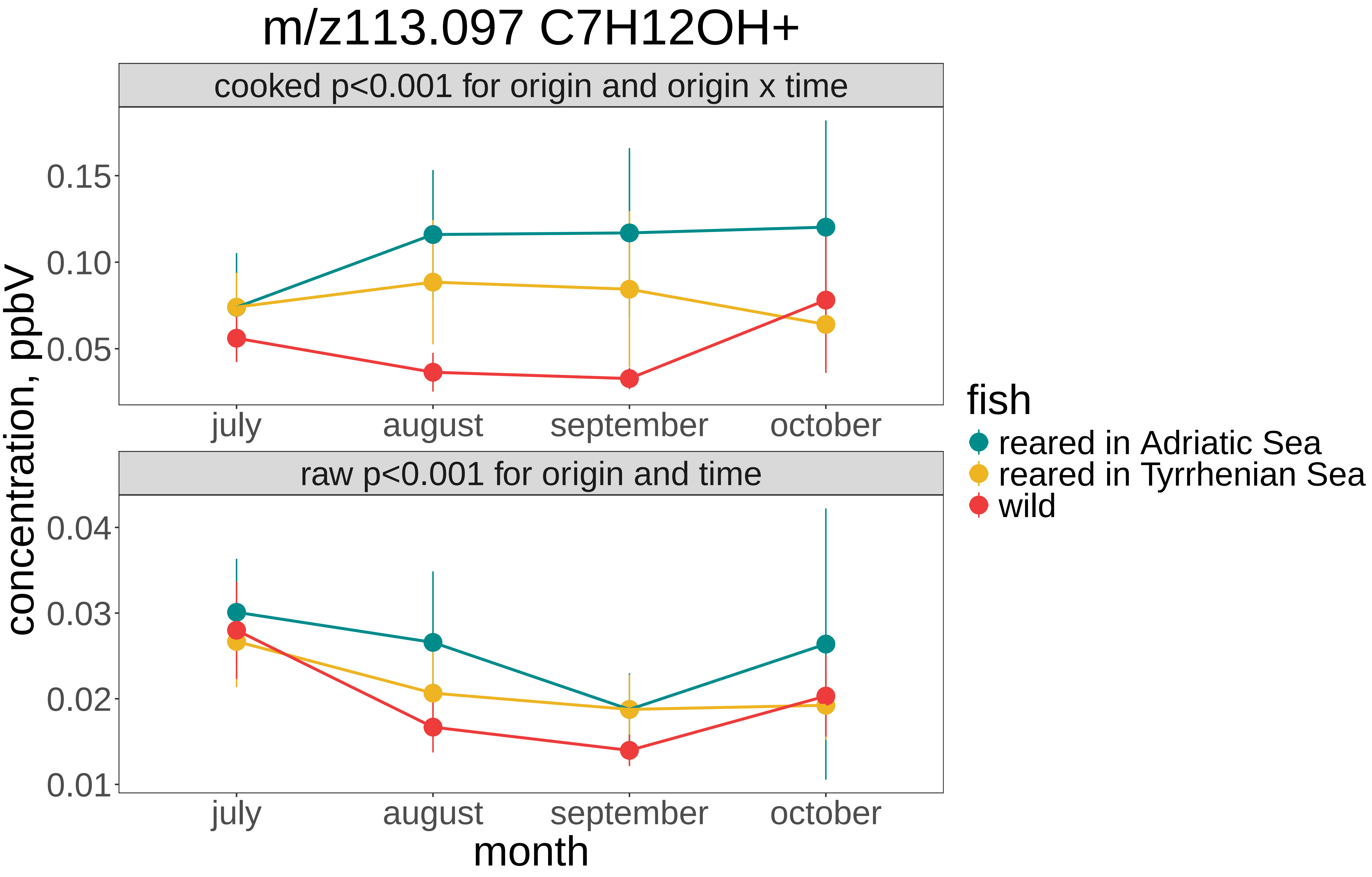

m/z113.097 C7H12OH+
cooked p<0.001 for origin and origin x time
raw p<0.001 for origin and time
concentration, ppbV
0.15
0.10
0.05
0.04
0.03
0.02
0.01
july
august
september
october
month
fish
reared in Adriatic Sea
reared in Tyrrhenian Sea
wild

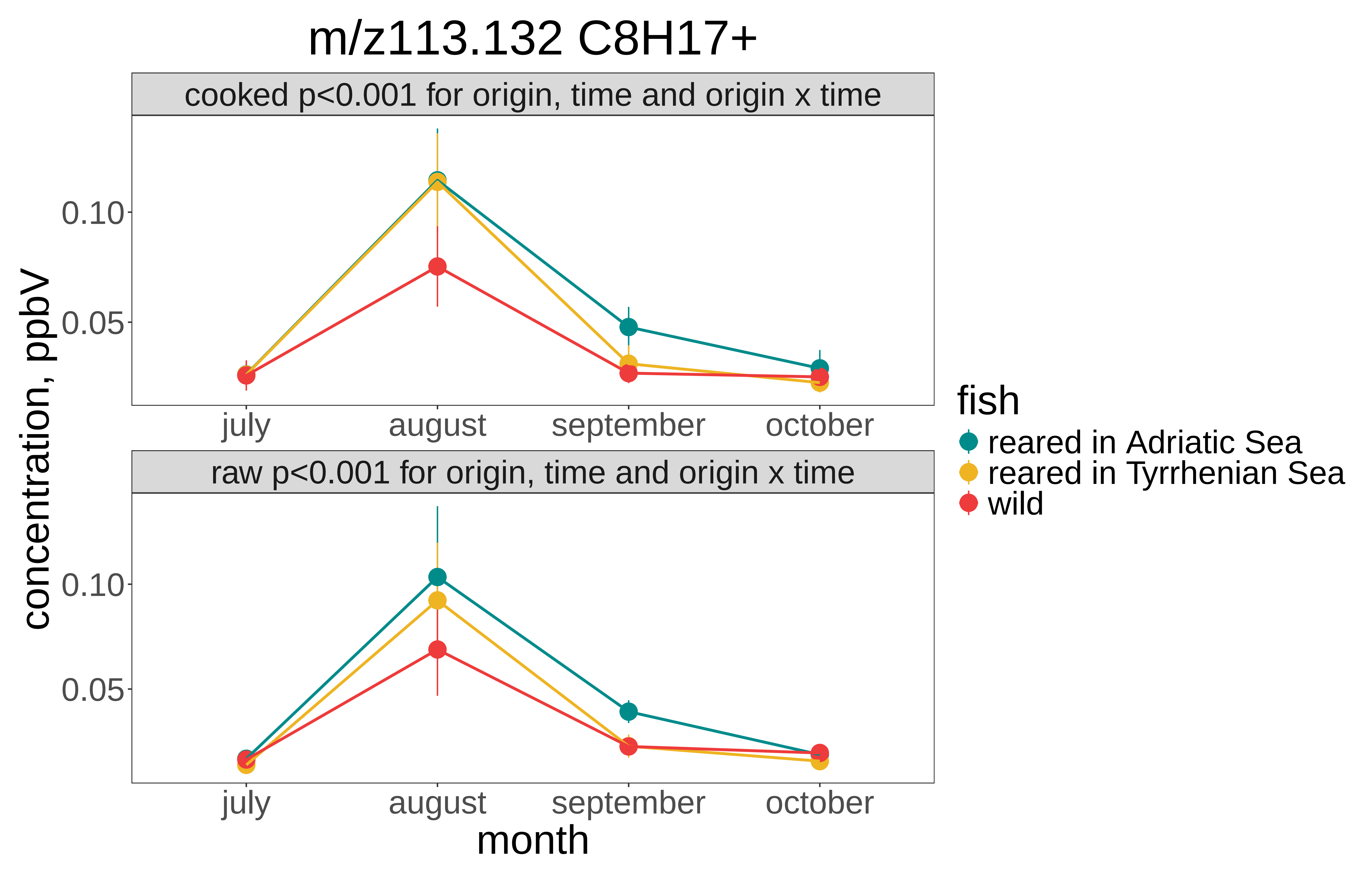
m/z113.132 C8H17+
cooked p<0.001 for origin, time and origin x time
raw p<0.001 for origin, time and origin x time
concentration, ppbV
0.10
0.05
0.10
0.05
july
august
september
october
july
august
september
october
month
fish
reared in Adriatic Sea
reared in Tyrrhenian Sea
wild

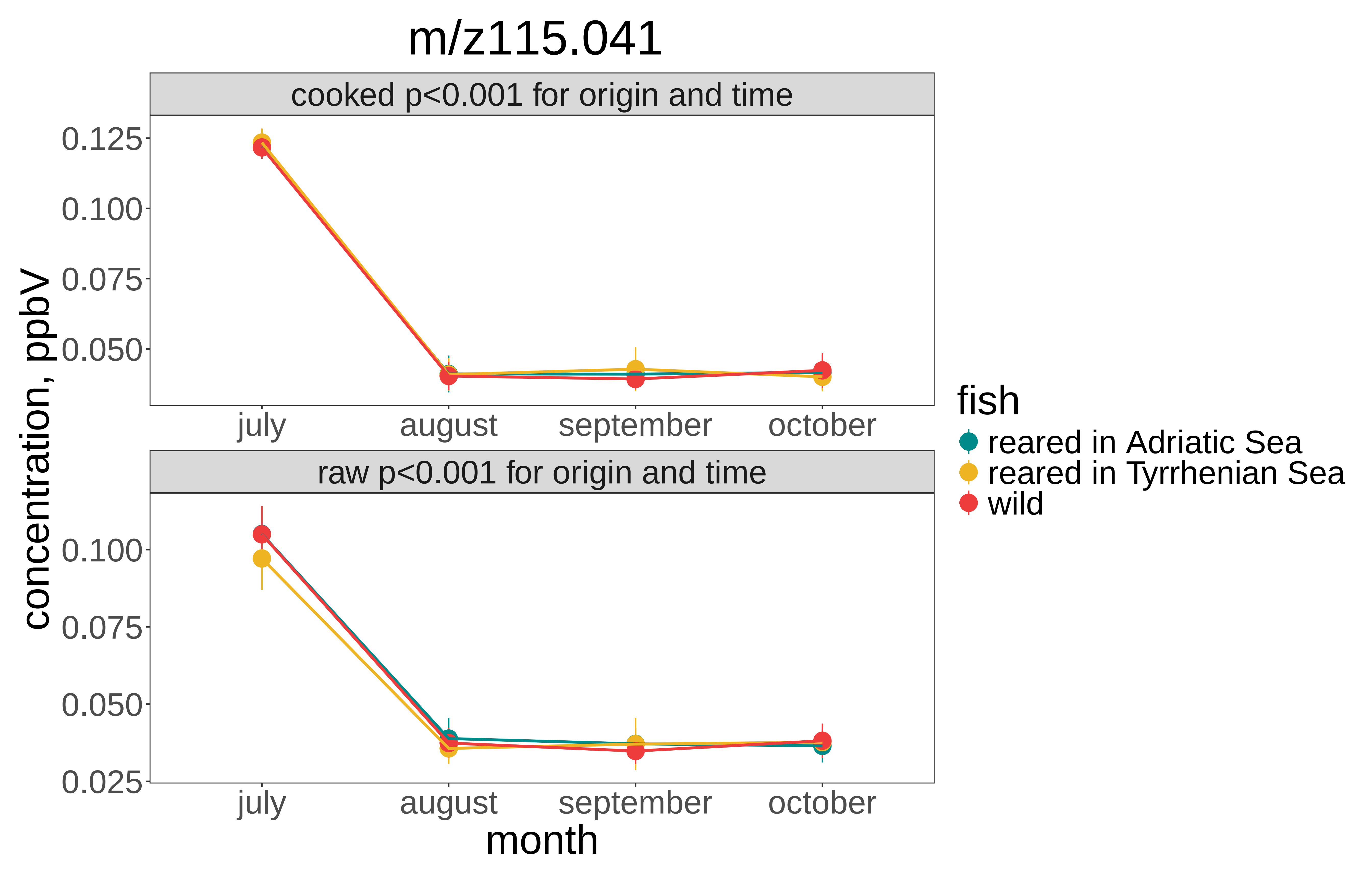
m/z115.041
cooked p<0.001 for origin and time
raw p<0.001 for origin and time
concentration, ppbV
0.125
0.100
0.075
0.050
0.025
july
august
september
october
month
fish
reared in Adriatic Sea
reared in Tyrrhenian Sea
wild

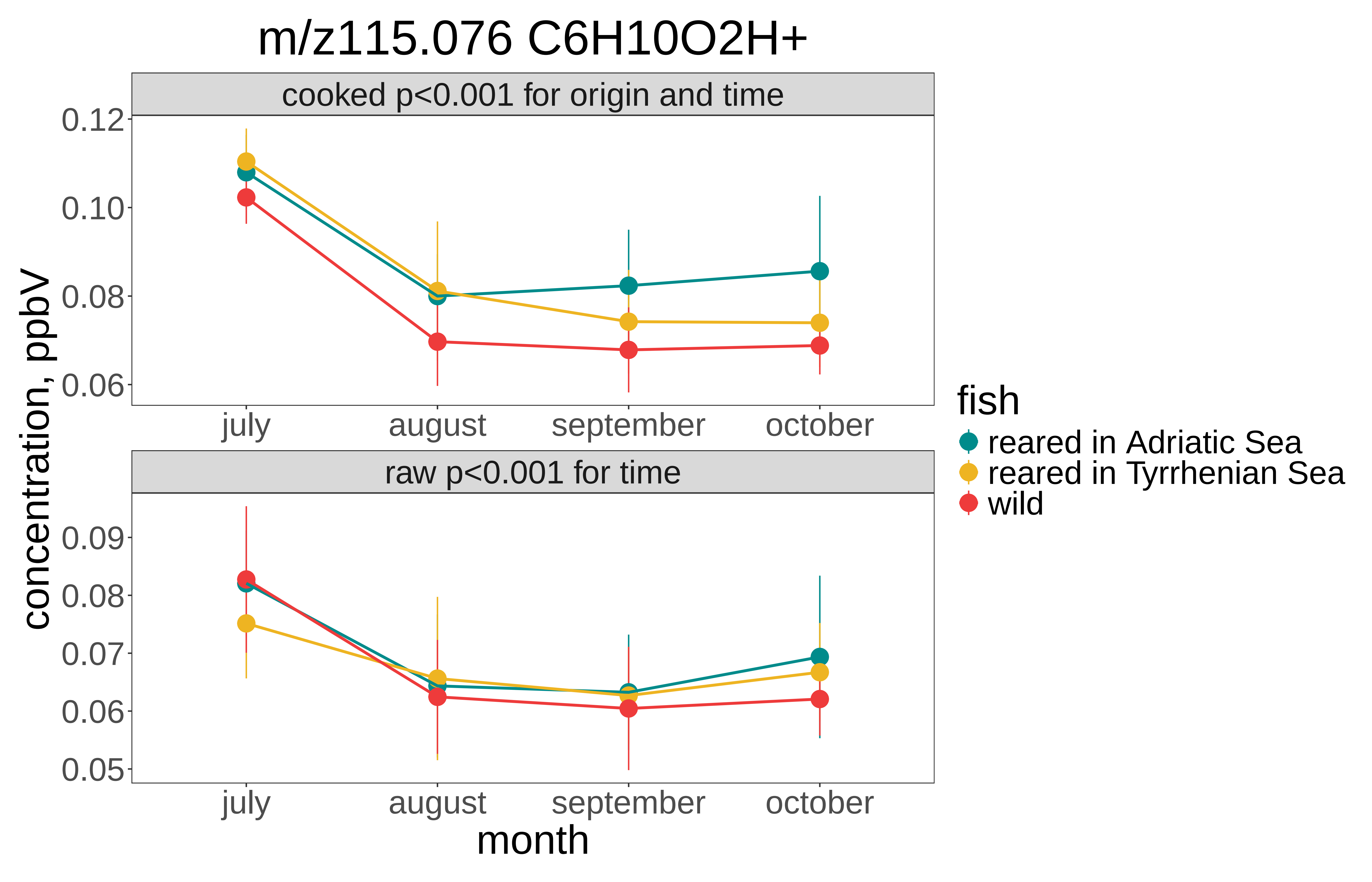
m/z115.076 C6H10O2H+
cooked p<0.001 for origin and time
raw p<0.001 for time
concentration, ppbV
0.12
0.10
0.08
0.06
0.09
0.08
0.07
0.06
0.05
july
august
september
october
month
fish
reared in Adriatic Sea
reared in Tyrrhenian Sea
wild

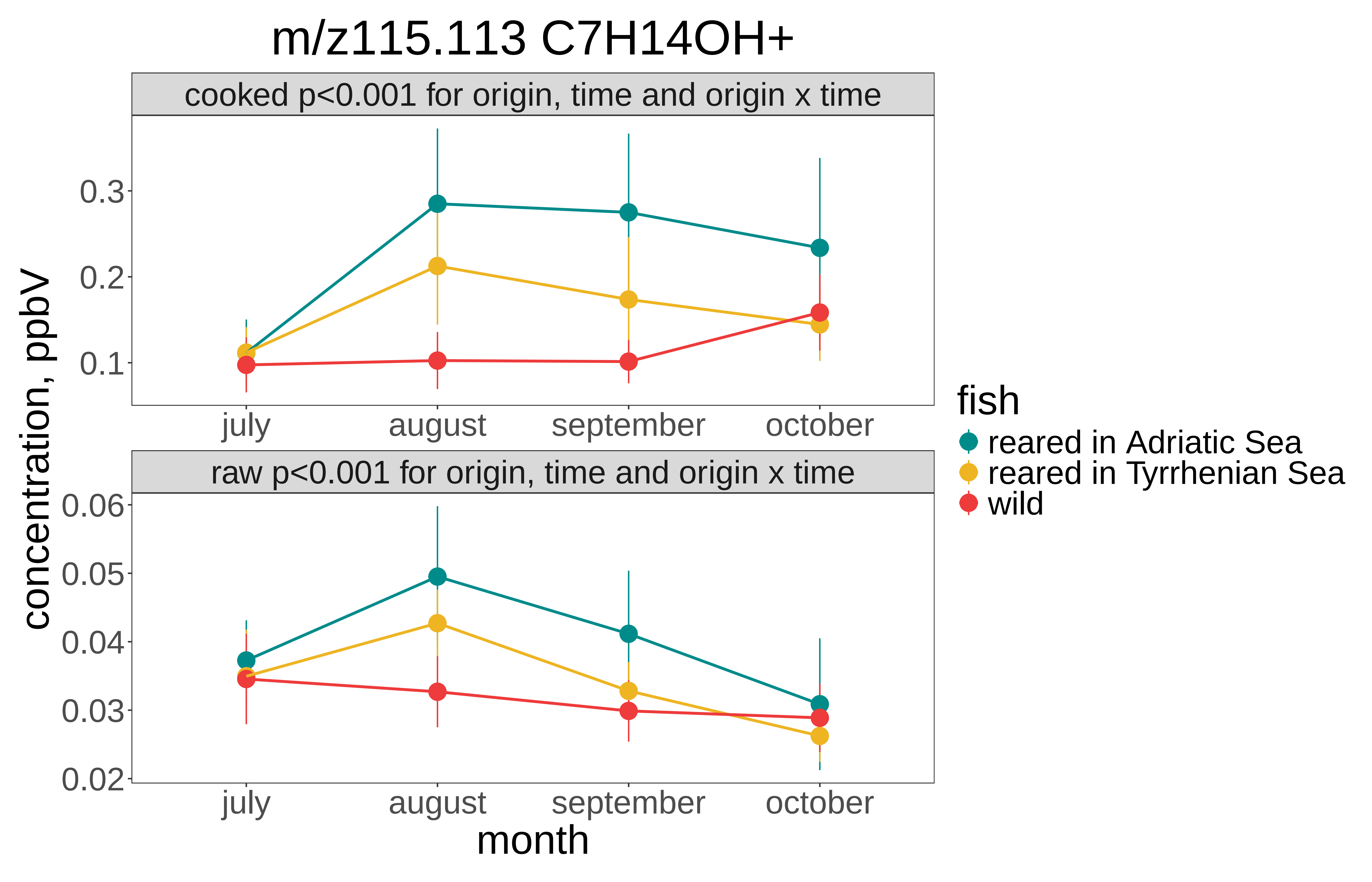

m/z115.113 C7H14OH+
cooked p<0.001 for origin, time and origin x time
raw p<0.001 for origin, time and origin x time
concentration, ppbV
0.3
0.2
0.1
0.06
0.05
0.04
0.03
0.02
july
august
september
october
month
fish
reared in Adriatic Sea
reared in Tyrrhenian Sea
wild

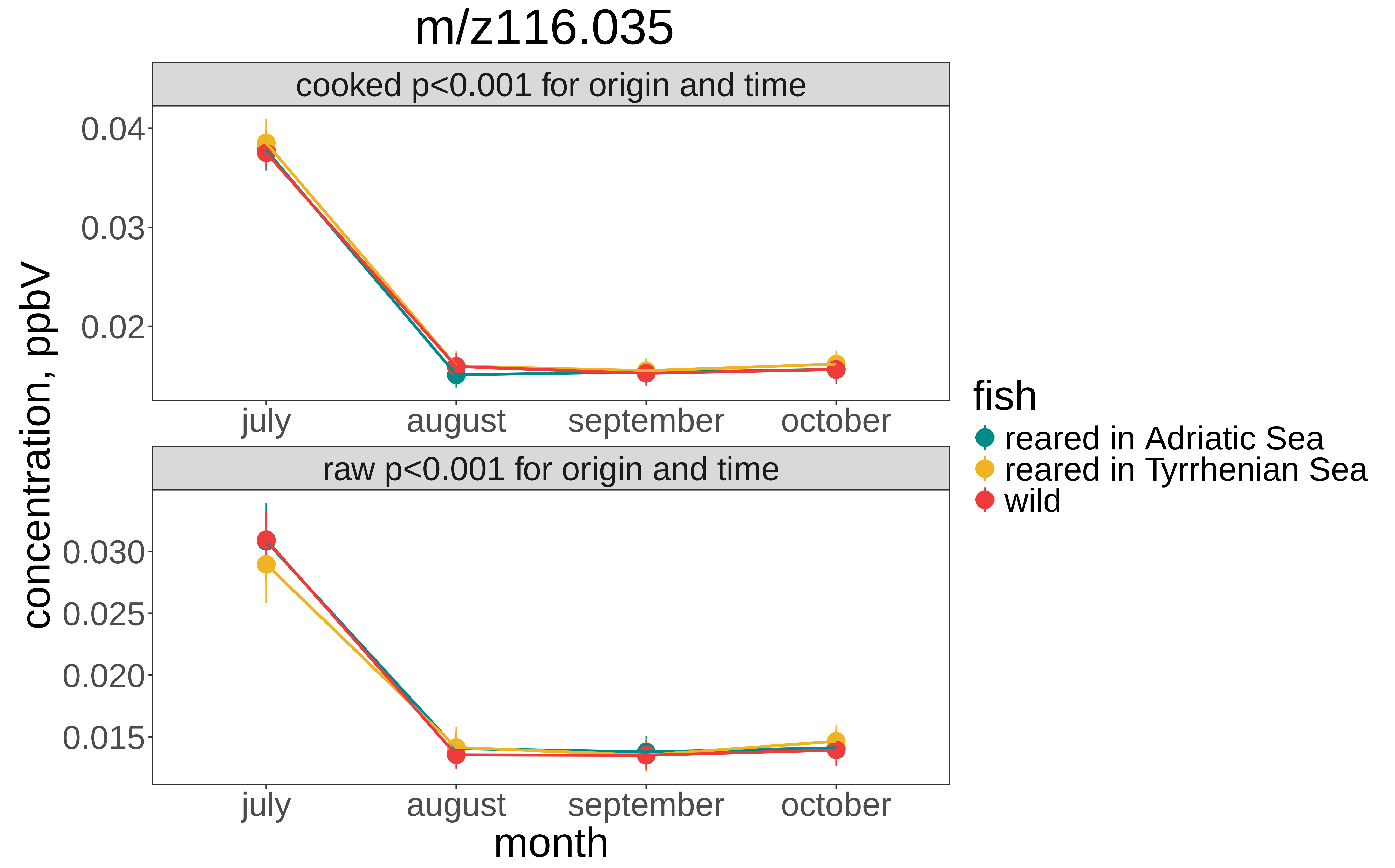

m/z116.035
cooked p<0.001 for origin and time
raw p<0.001 for origin and time
concentration, ppbV
0.04
0.03
0.02
0.030
0.025
0.020
0.015
july
august
september
october
month
fish
reared in Adriatic Sea
reared in Tyrrhenian Sea
wild

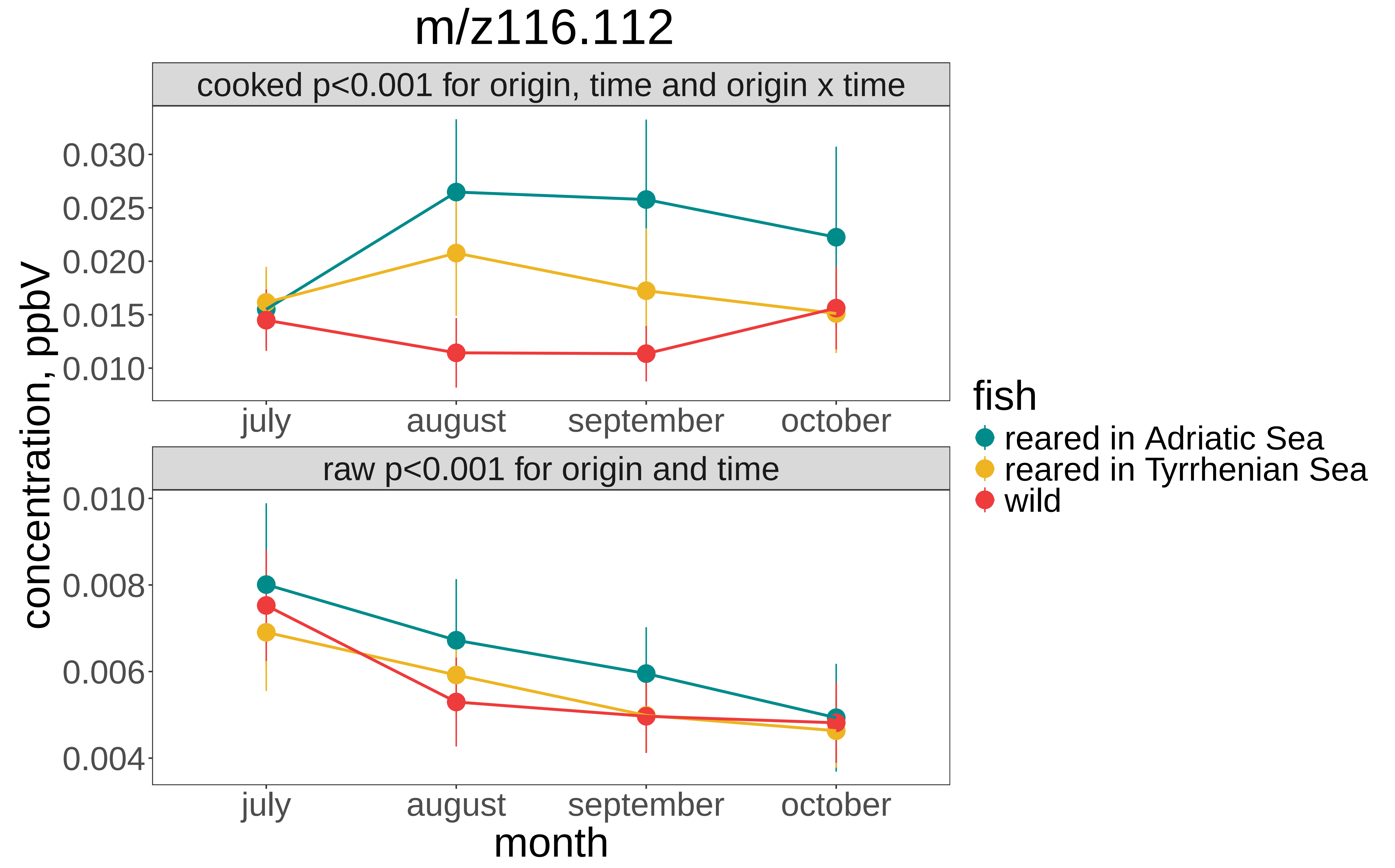
m/z116.112
cooked p<0.001 for origin, time and origin x time
raw p<0.001 for origin and time
concentration, ppbV
0.030
0.025
0.020
0.015
0.010
0.010
0.008
0.006
0.004
july
august
september
october
month
fish
reared in Adriatic Sea
reared in Tyrrhenian Sea
wild

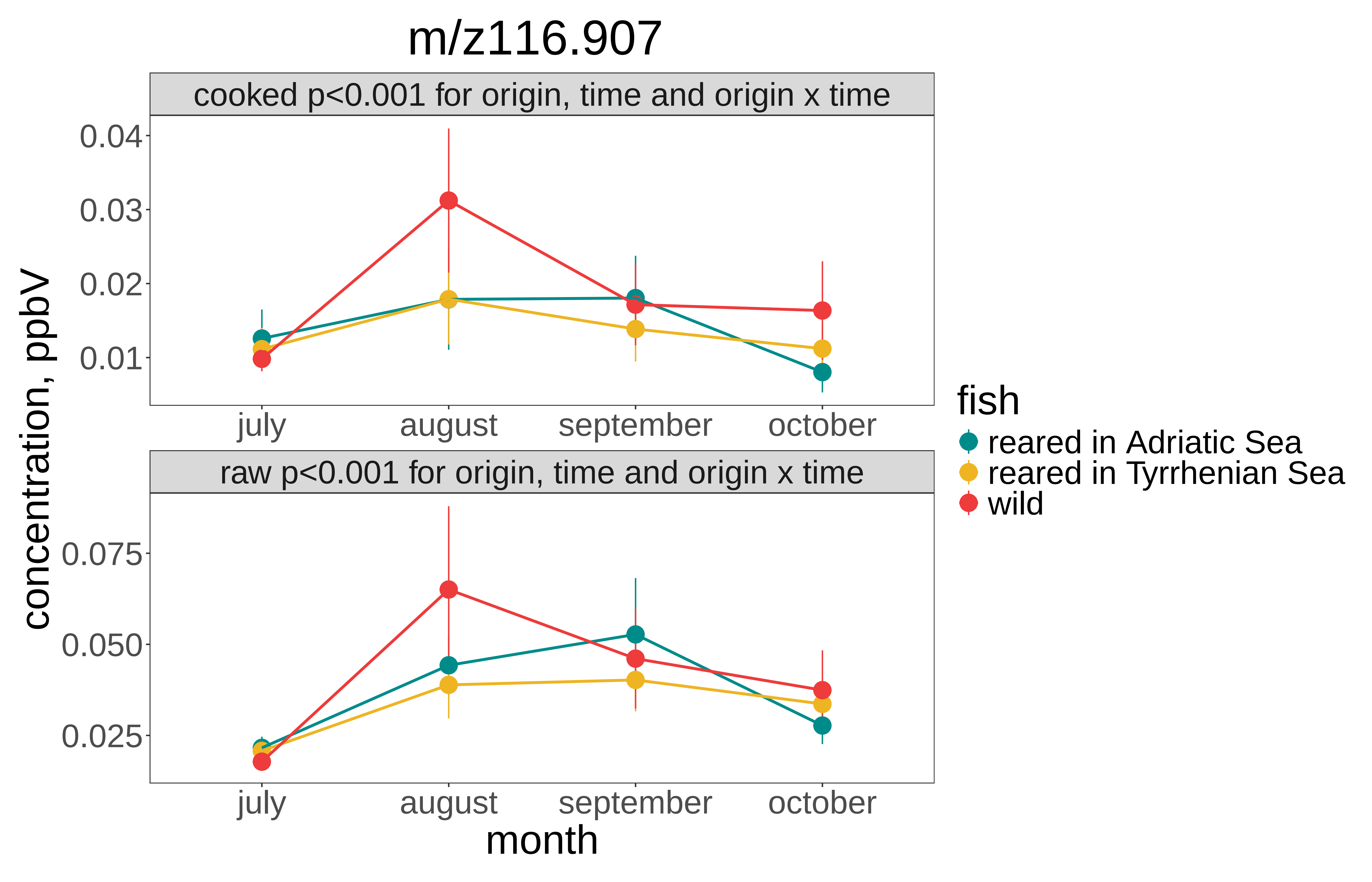
m/z116.907
cooked p<0.001 for origin, time and origin x time
raw p<0.001 for origin, time and origin x time
concentration, ppbV
0.04
0.03
0.02
0.01
0.075
0.050
0.025
july
august
september
october
month
fish
reared in Adriatic Sea
reared in Tyrrhenian Sea
wild

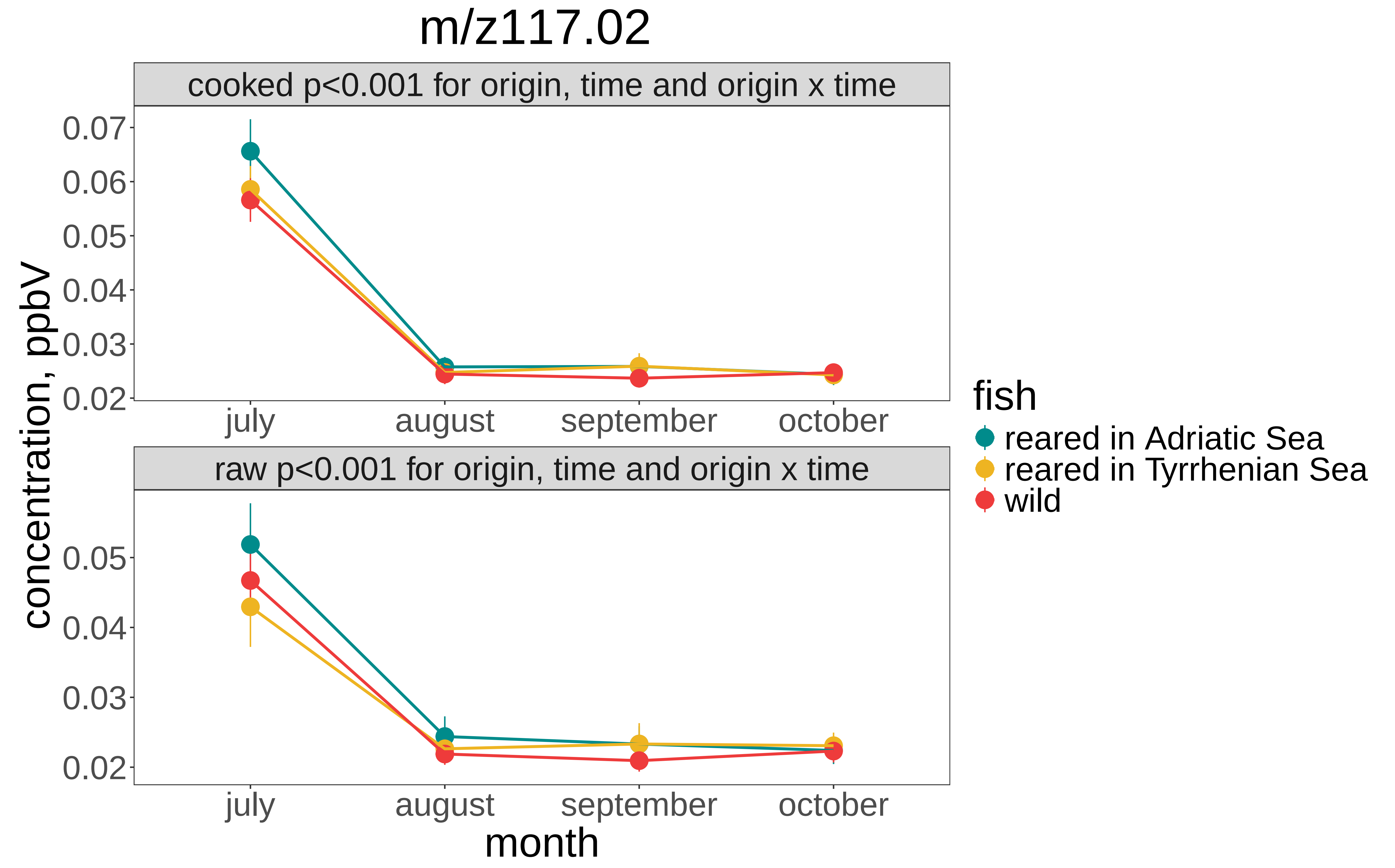
m/z117.02
cooked p<0.001 for origin, time and origin x time
raw p<0.001 for origin, time and origin x time
concentration, ppbV
0.07
0.06
0.05
0.04
0.03
0.02
july
august
september
october
month
fish
reared in Adriatic Sea
reared in Tyrrhenian Sea
wild

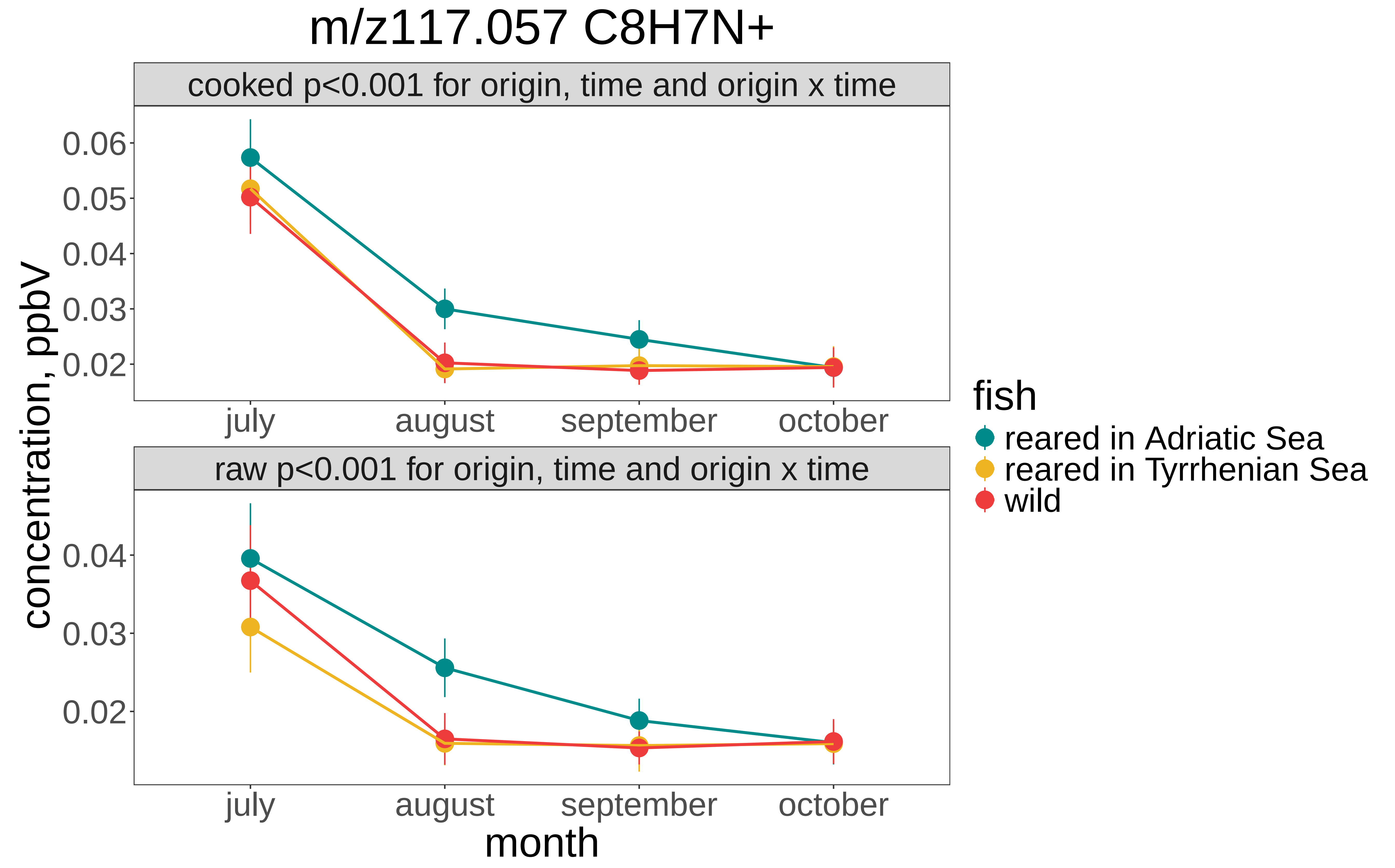
m/z117.057 C8H7N+
cooked p<0.001 for origin, time and origin x time
raw p<0.001 for origin, time and origin x time
concentration, ppbV
0.06
0.05
0.04
0.03
0.02
0.04
0.03
0.02
july
august
september
october
july
august
september
october
month
fish
reared in Adriatic Sea
reared in Tyrrhenian Sea
wild

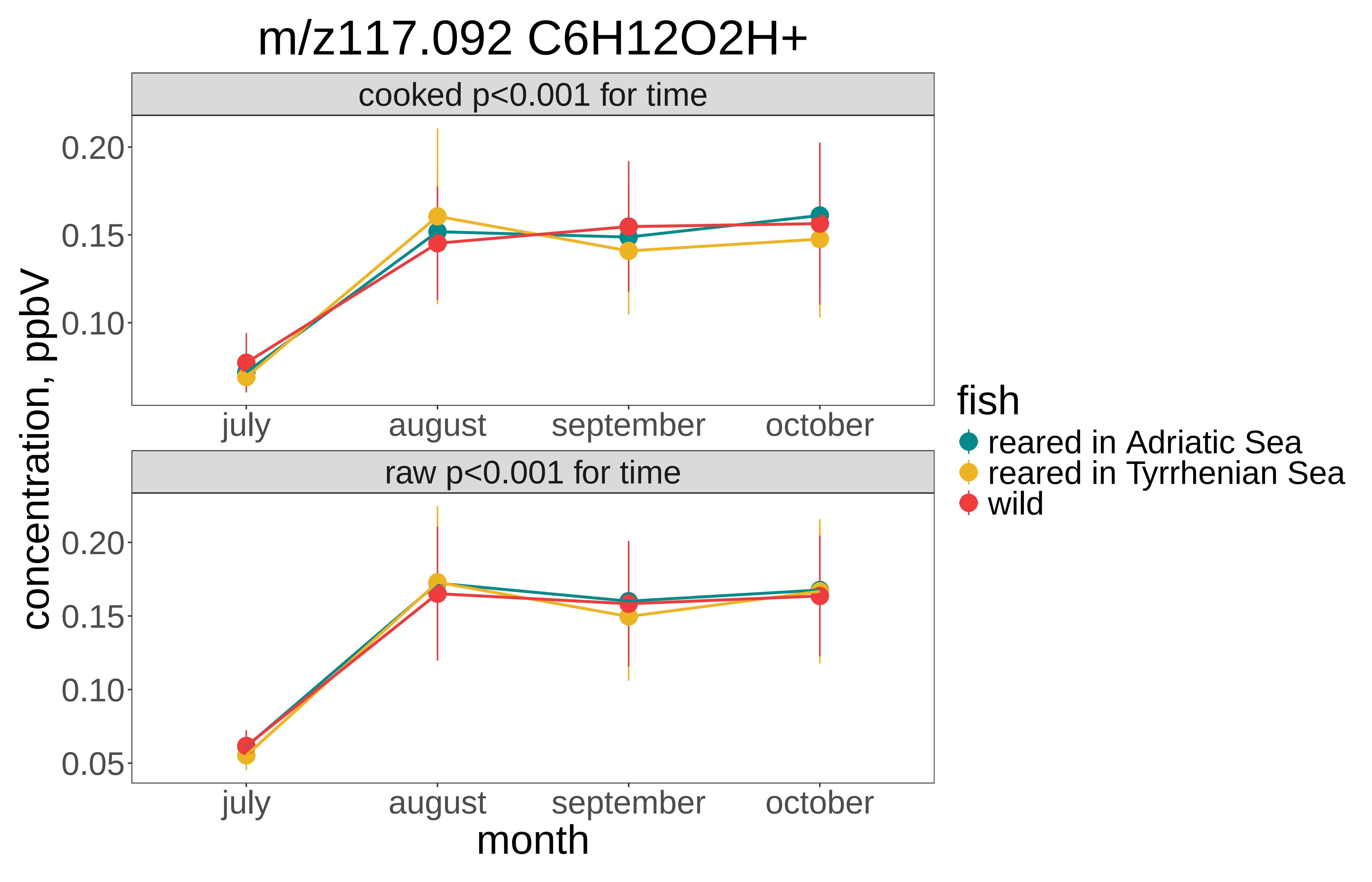

m/z117.092 C6H12O2H+
cooked p<0.001 for time
raw p<0.001 for time
0.20
0.15
0.10
0.05
july
august
september
october
concentration, ppbV
month
fish
reared in Adriatic Sea
reared in Tyrrhenian Sea
wild

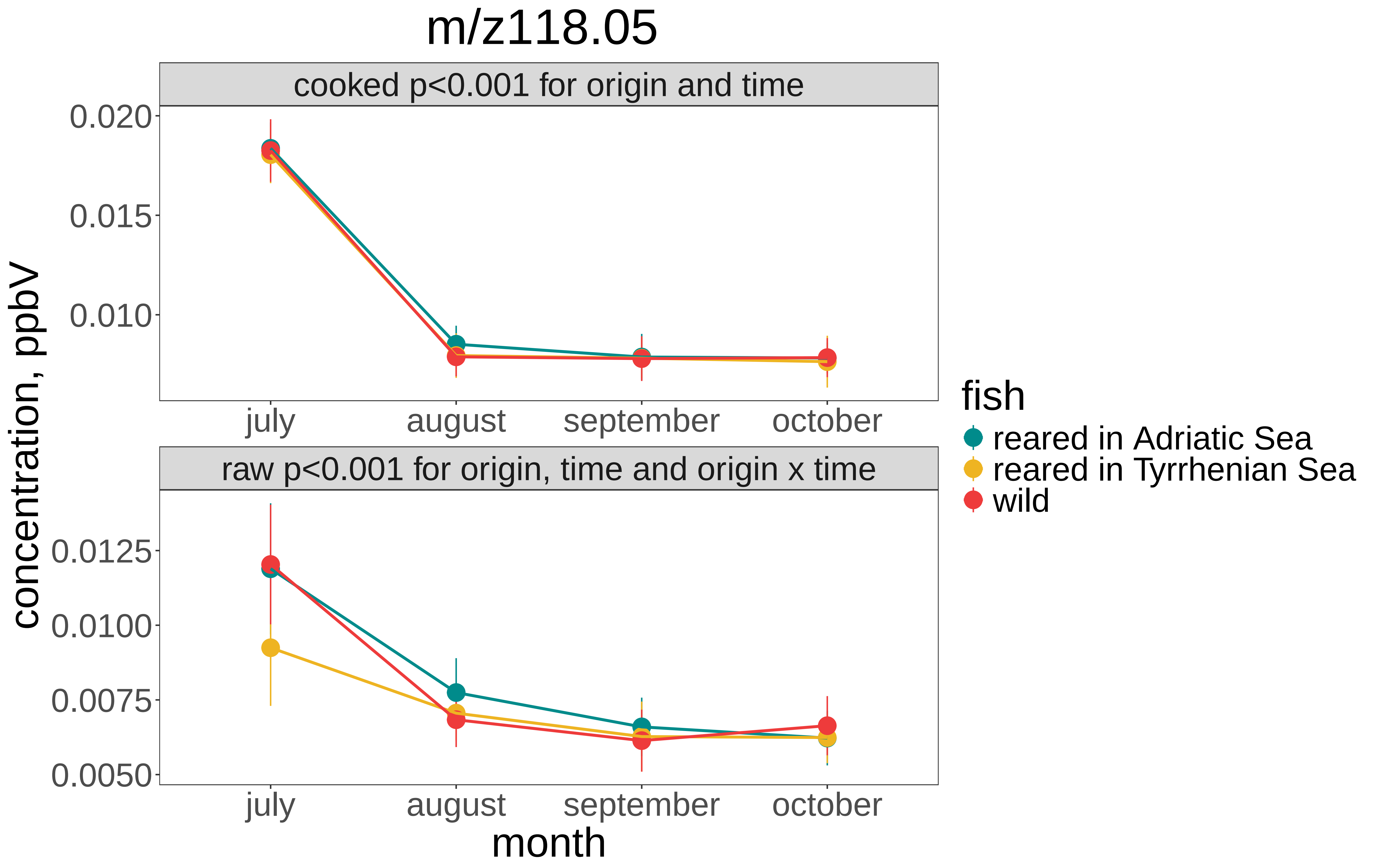

m/z118.05
cooked p<0.001 for origin and time
0.020
0.015
0.010
july
august
september
october
raw p<0.001 for origin, time and origin x time
0.0125
0.0100
0.0075
0.0050
july
august
september
october
month
concentration, ppbV
fish
reared in Adriatic Sea
reared in Tyrrhenian Sea
wild

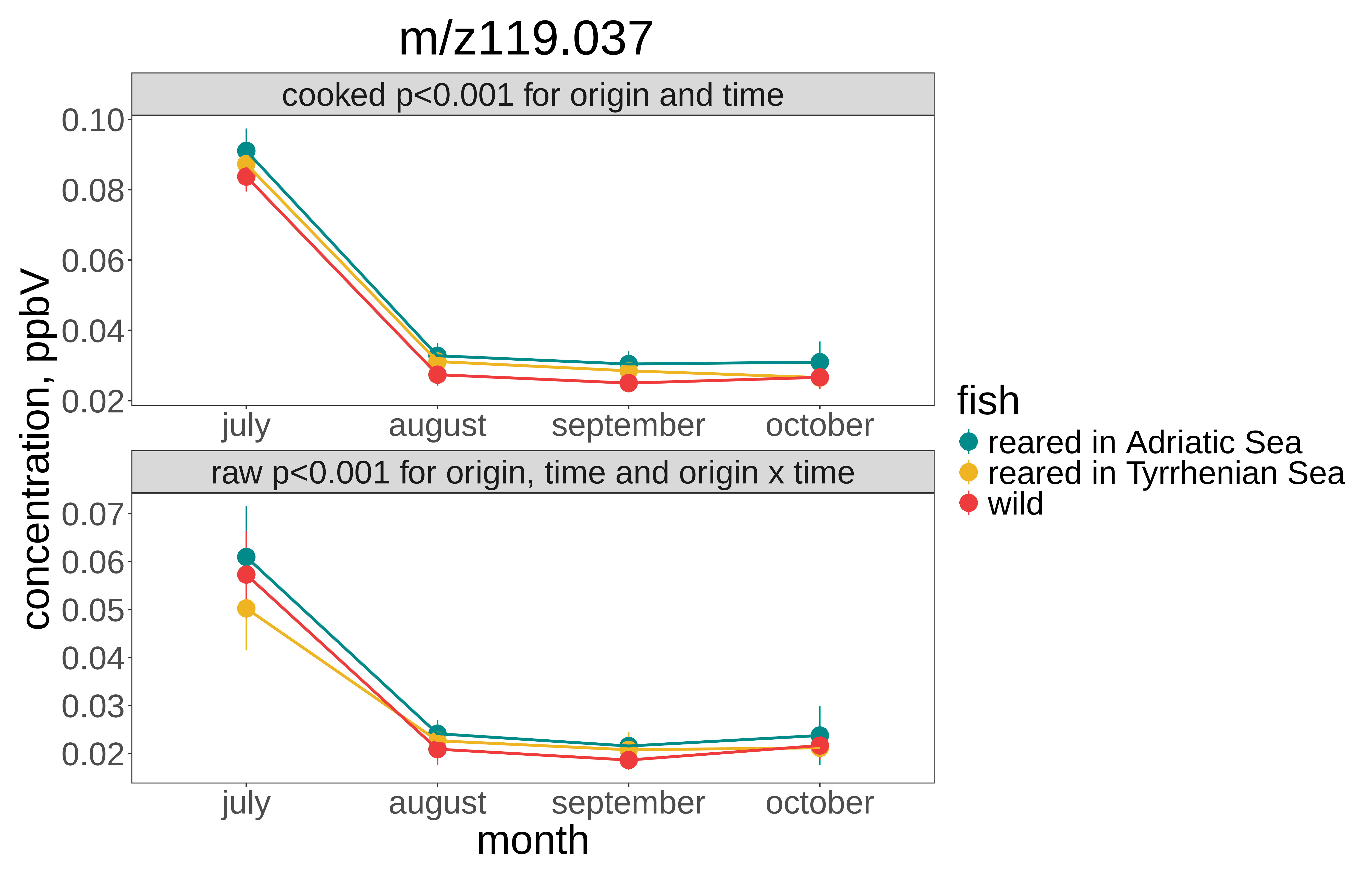

m/z119.037
cooked p<0.001 for origin and time
raw p<0.001 for origin, time and origin x time
concentration, ppbV
0.10
0.08
0.06
0.04
0.02
0.07
0.06
0.05
0.04
0.03
0.02
july
august
september
october
month
fish
reared in Adriatic Sea
reared in Tyrrhenian Sea
wild

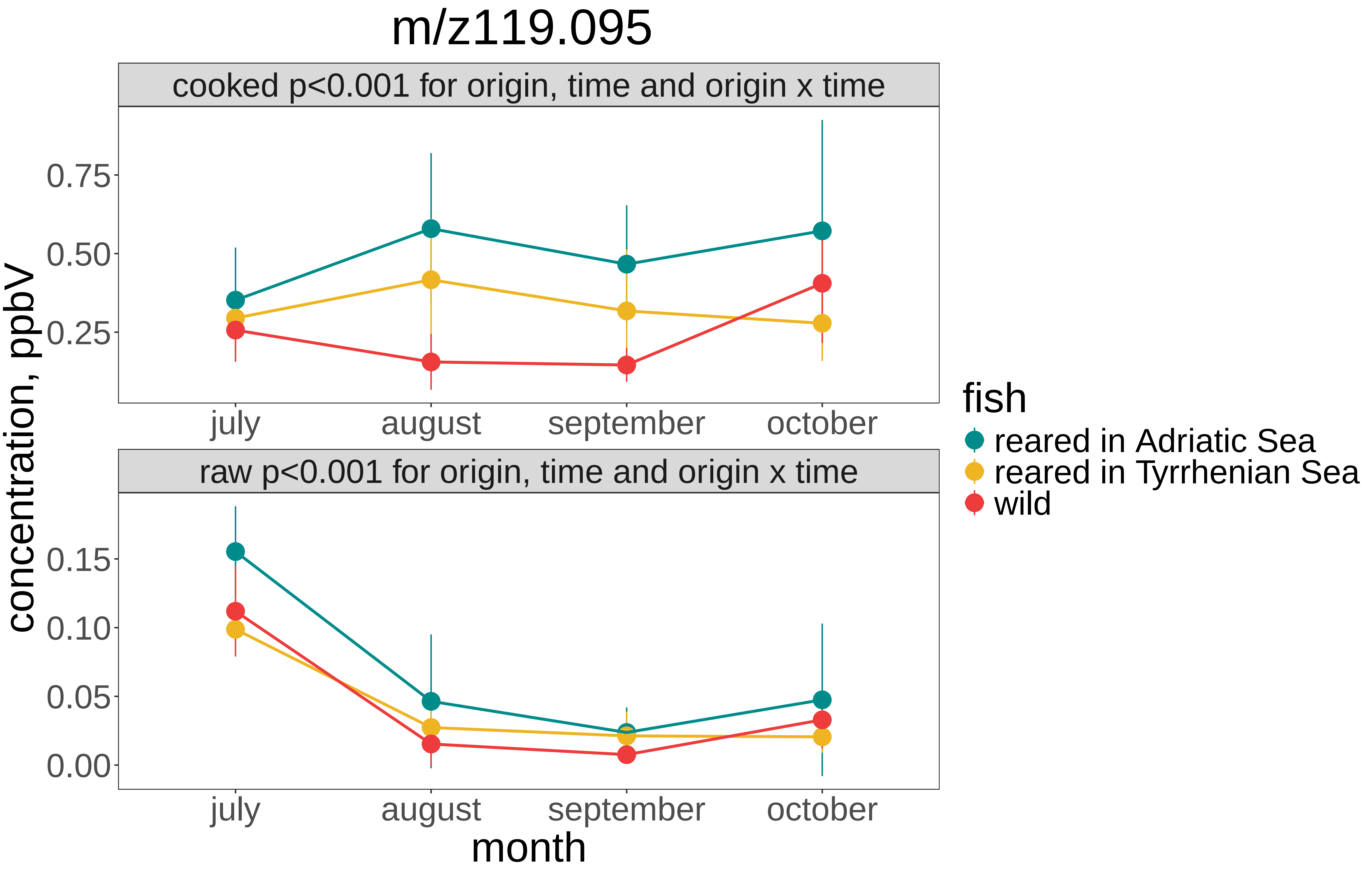

m/z119.095
cooked p<0.001 for origin, time and origin x time
raw p<0.001 for origin, time and origin x time
concentration, ppbV
0.75
0.50
0.25
0.15
0.10
0.05
0.00
july
august
september
october
month
fish
reared in Adriatic Sea
reared in Tyrrhenian Sea
wild

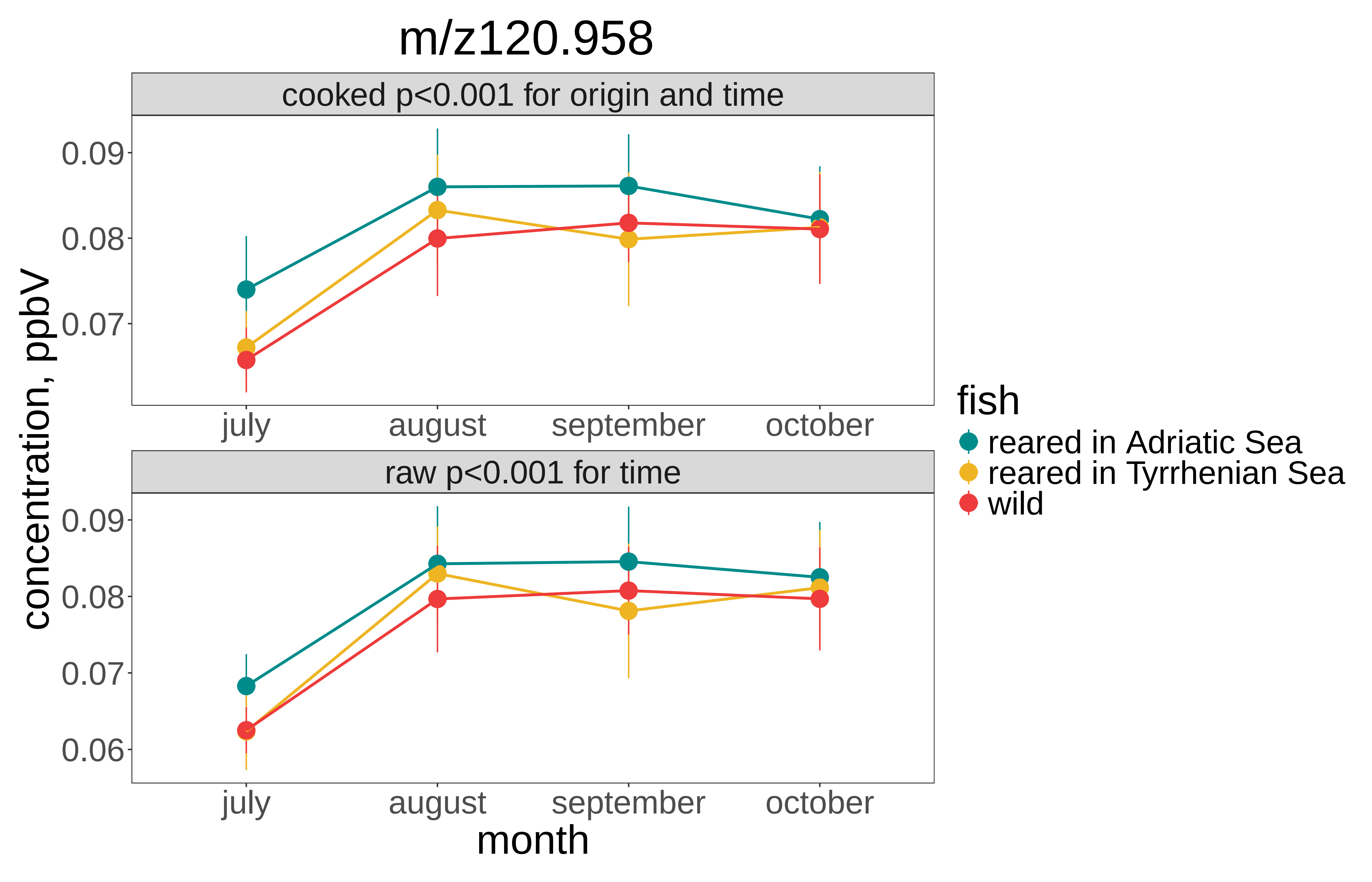
m/z120.958
cooked p<0.001 for origin and time
raw p<0.001 for time
0.09
0.08
0.07
0.06
july
august
september
october
concentration, ppbV
month
fish
reared in Adriatic Sea
reared in Tyrrhenian Sea
wild

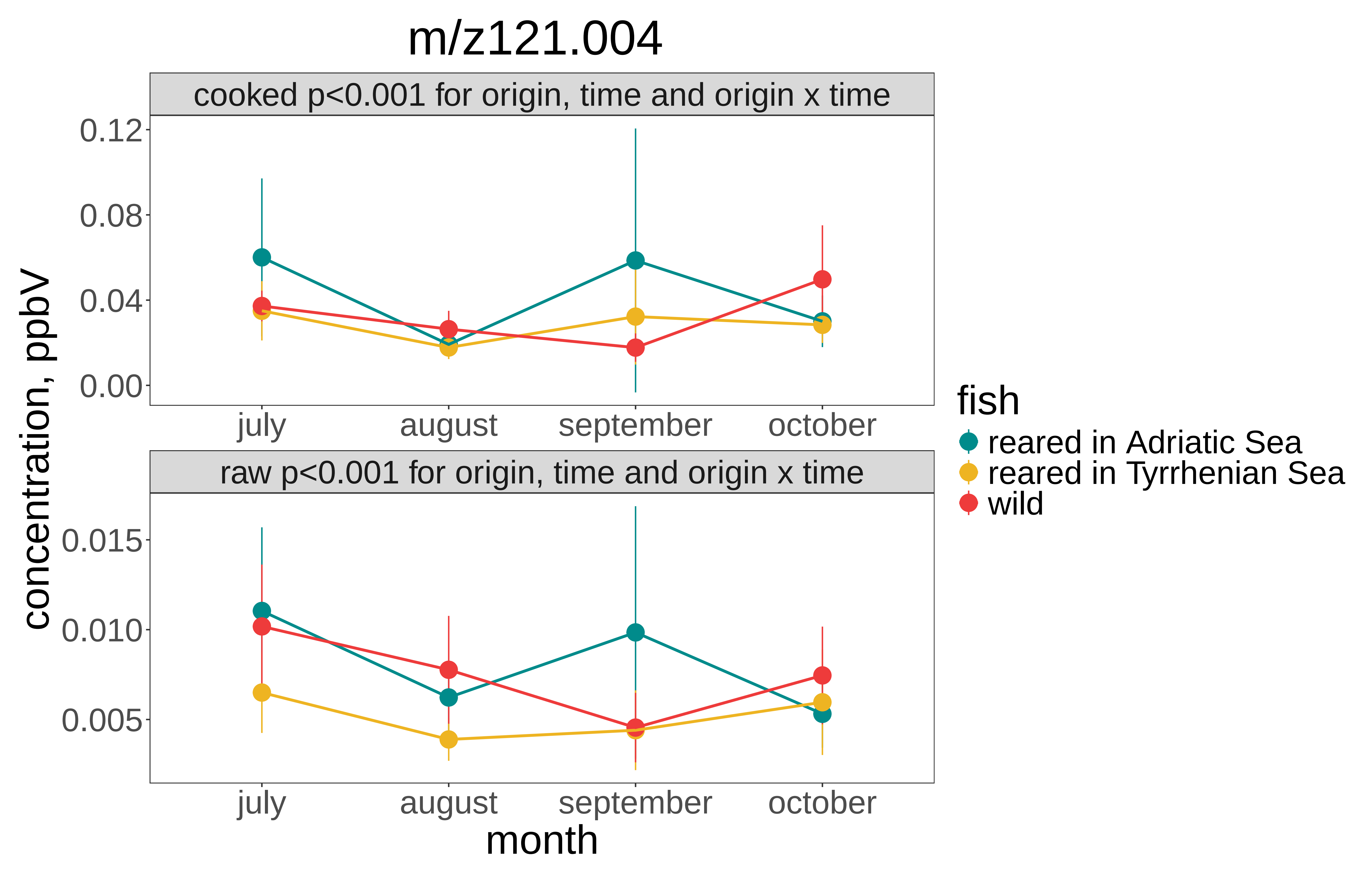
m/z121.004
cooked p<0.001 for origin, time and origin x time
raw p<0.001 for origin, time and origin x time
concentration, ppbV
0.12
0.08
0.04
0.00
0.015
0.010
0.005
july
august
september
october
month
fish
reared in Adriatic Sea
reared in Tyrrhenian Sea
wild

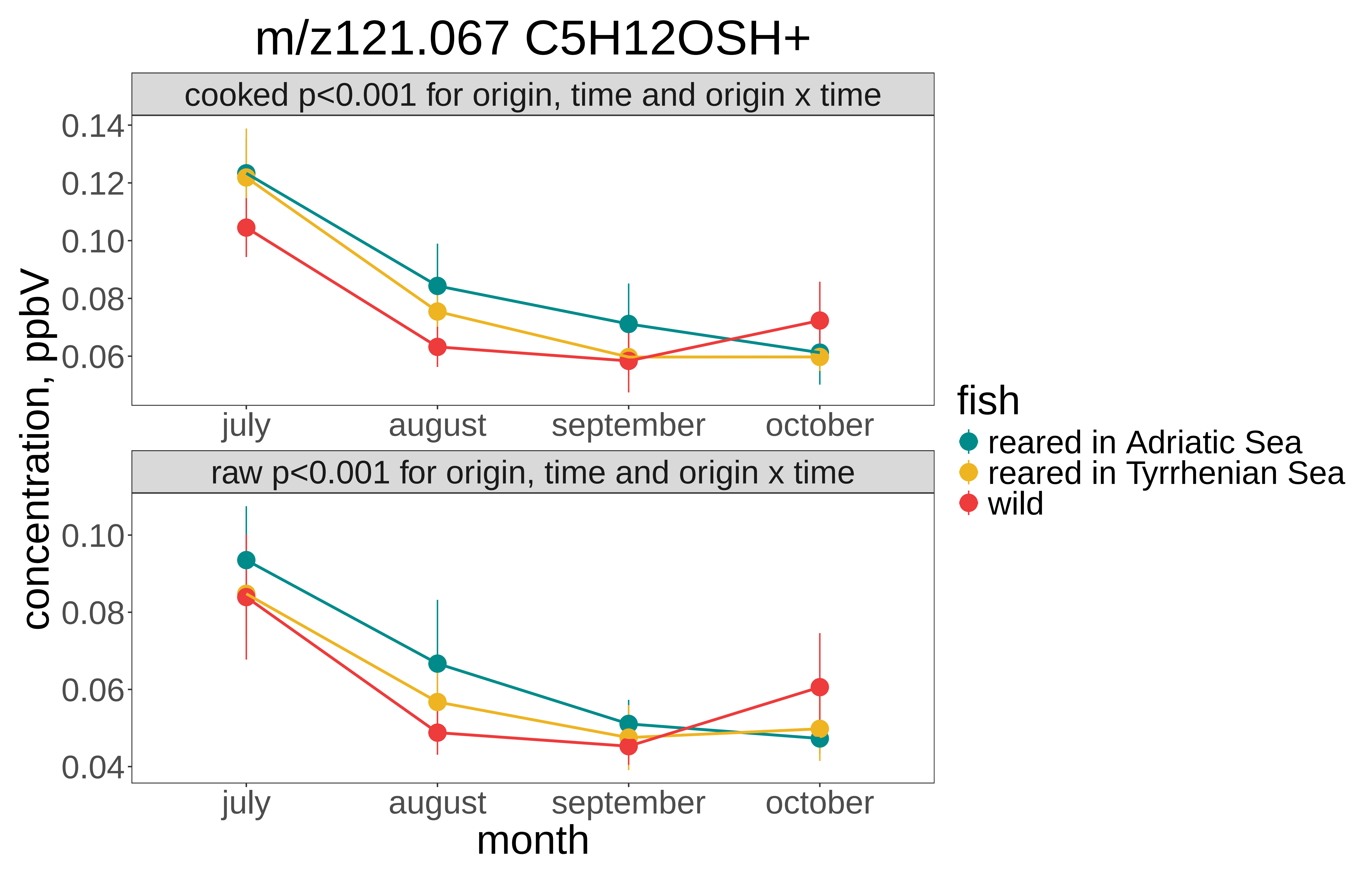
m/z121.067 C5H12OSH+
cooked p<0.001 for origin, time and origin x time
raw p<0.001 for origin, time and origin x time
concentration, ppbV
0.14
0.12
0.10
0.08
0.06
0.04
july
august
september
october
month
fish
reared in Adriatic Sea
reared in Tyrrhenian Sea
wild

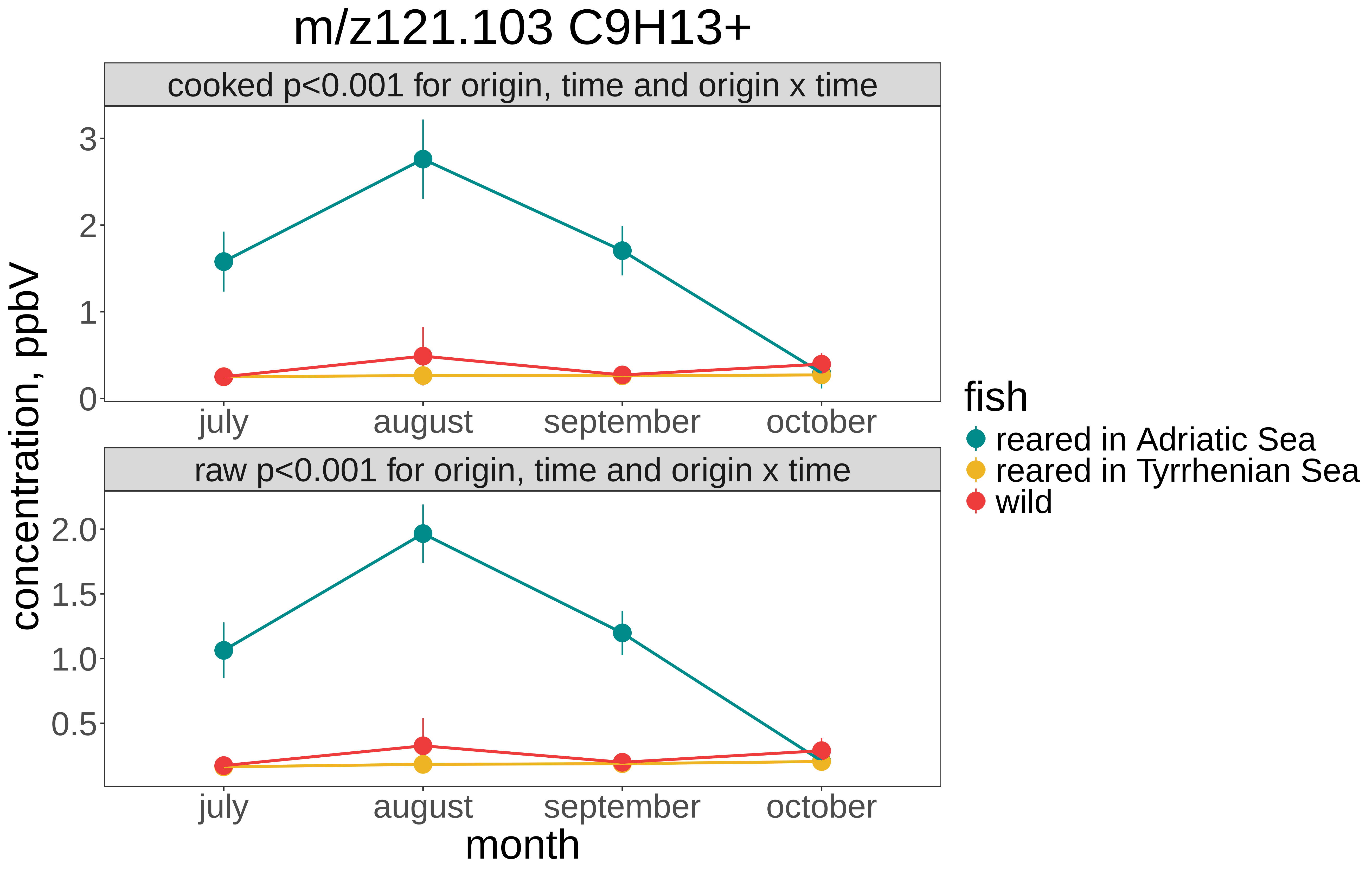
m/z121.103 C9H13+
cooked p<0.001 for origin, time and origin x time
raw p<0.001 for origin, time and origin x time
concentration, ppbV
0
1
2
3
0.5
1.0
1.5
2.0
july
august
september
october
month
fish
reared in Adriatic Sea
reared in Tyrrhenian Sea
wild

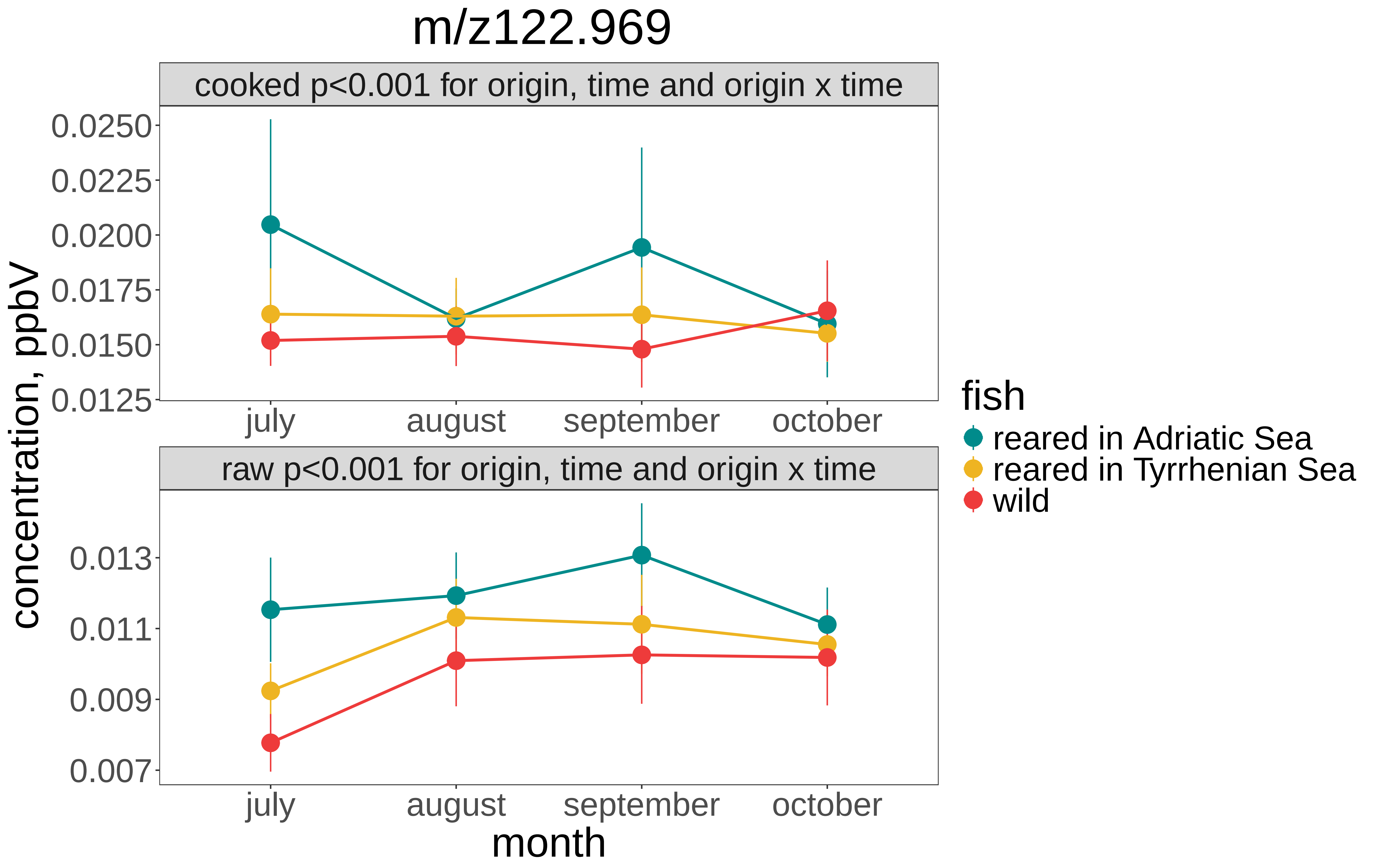

m/z122.969
cooked p<0.001 for origin, time and origin x time
raw p<0.001 for origin, time and origin x time
0.0250
0.0225
0.0200
0.0175
0.0150
0.0125
0.013
0.011
0.009
0.007
july
august
september
october
month
concentration, ppbV
fish
reared in Adriatic Sea
reared in Tyrrhenian Sea
wild

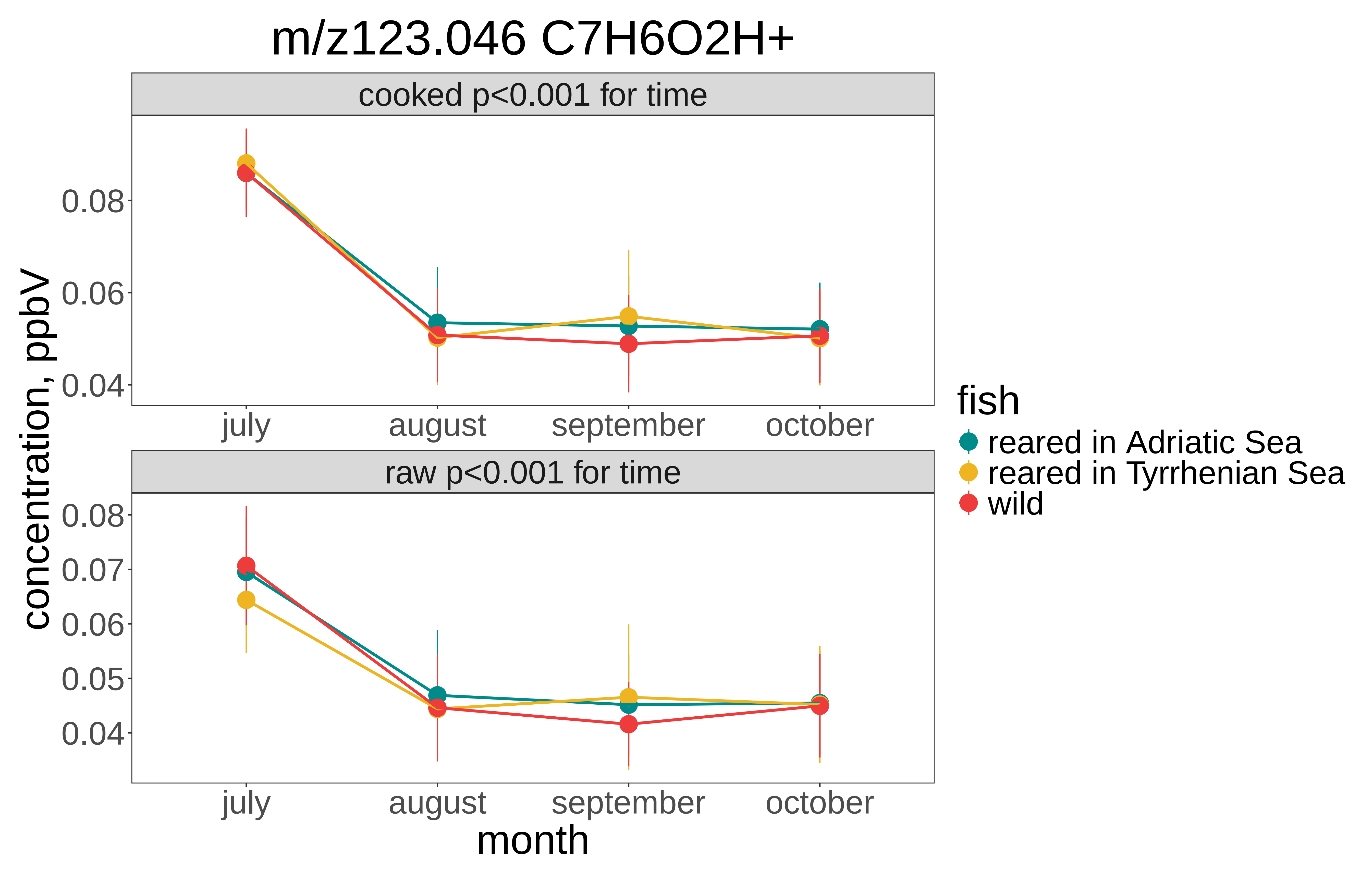
m/z123.046 C7H6O2H+
cooked p<0.001 for time
raw p<0.001 for time
0.08
0.06
0.04
0.08
0.07
0.06
0.05
0.04
concentration, ppbV
july
august
september
october
month
fish
reared in Adriatic Sea
reared in Tyrrhenian Sea
wild

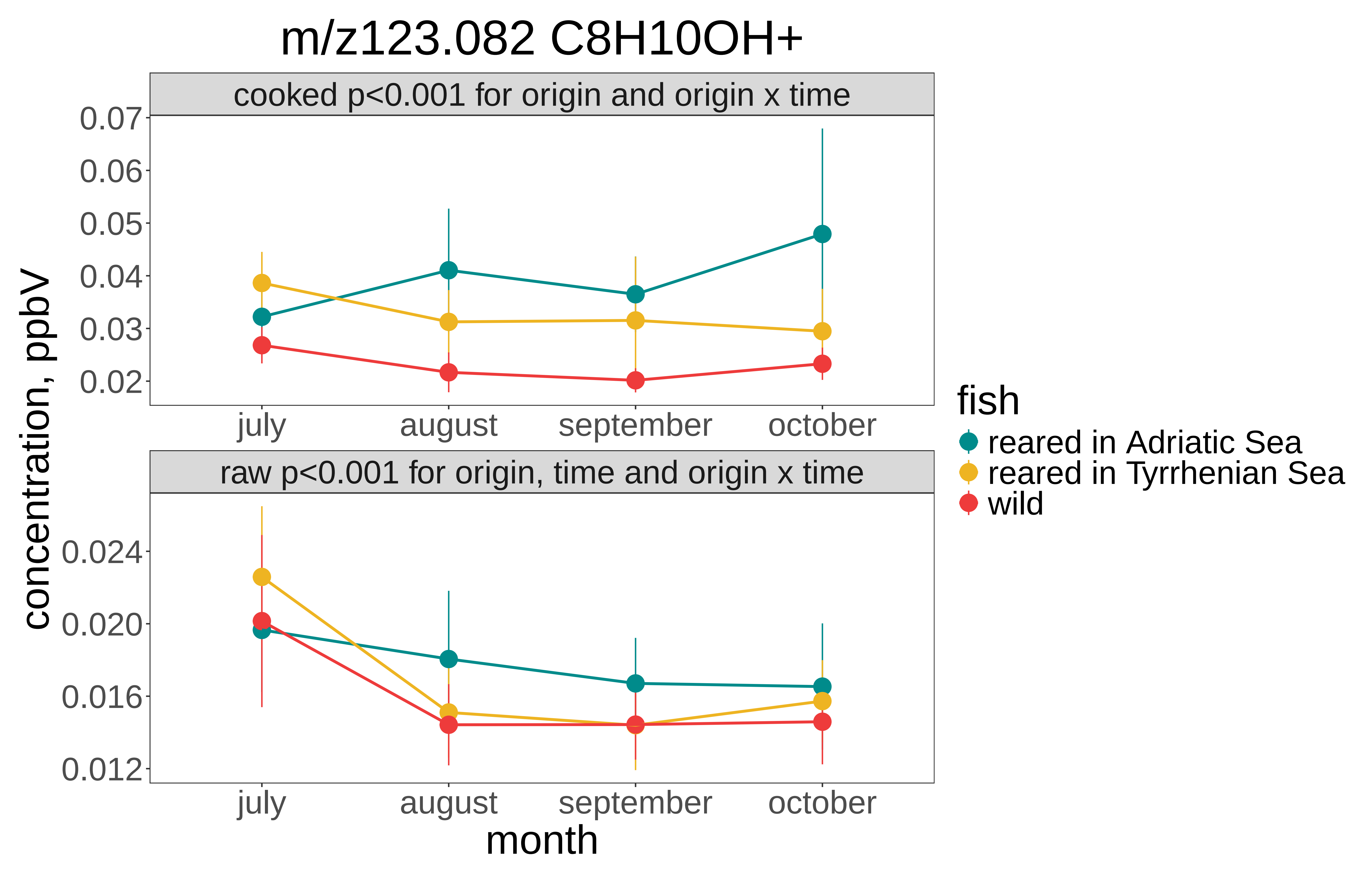

m/z123.082 C8H10OH+
cooked p<0.001 for origin and origin x time
raw p<0.001 for origin, time and origin x time
concentration, ppbV
0.07
0.06
0.05
0.04
0.03
0.02
0.024
0.020
0.016
0.012
july
august
september
october
month
fish
reared in Adriatic Sea
reared in Tyrrhenian Sea
wild

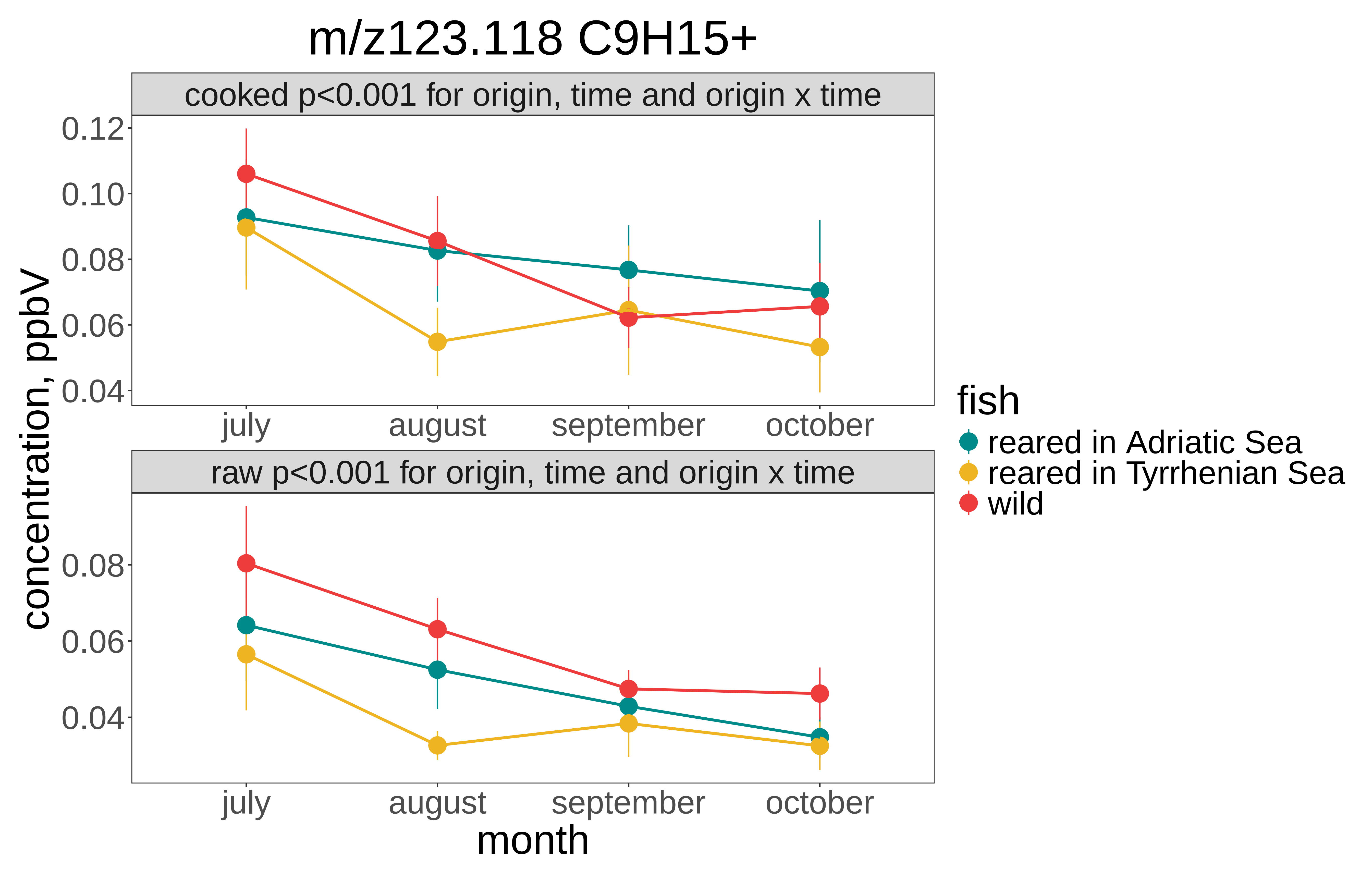

m/z123.118 C9H15+
cooked p<0.001 for origin, time and origin x time
raw p<0.001 for origin, time and origin x time
0.12
0.10
0.08
0.06
0.04
concentration, ppbV
july
august
september
october
month
fish
reared in Adriatic Sea
reared in Tyrrhenian Sea
wild

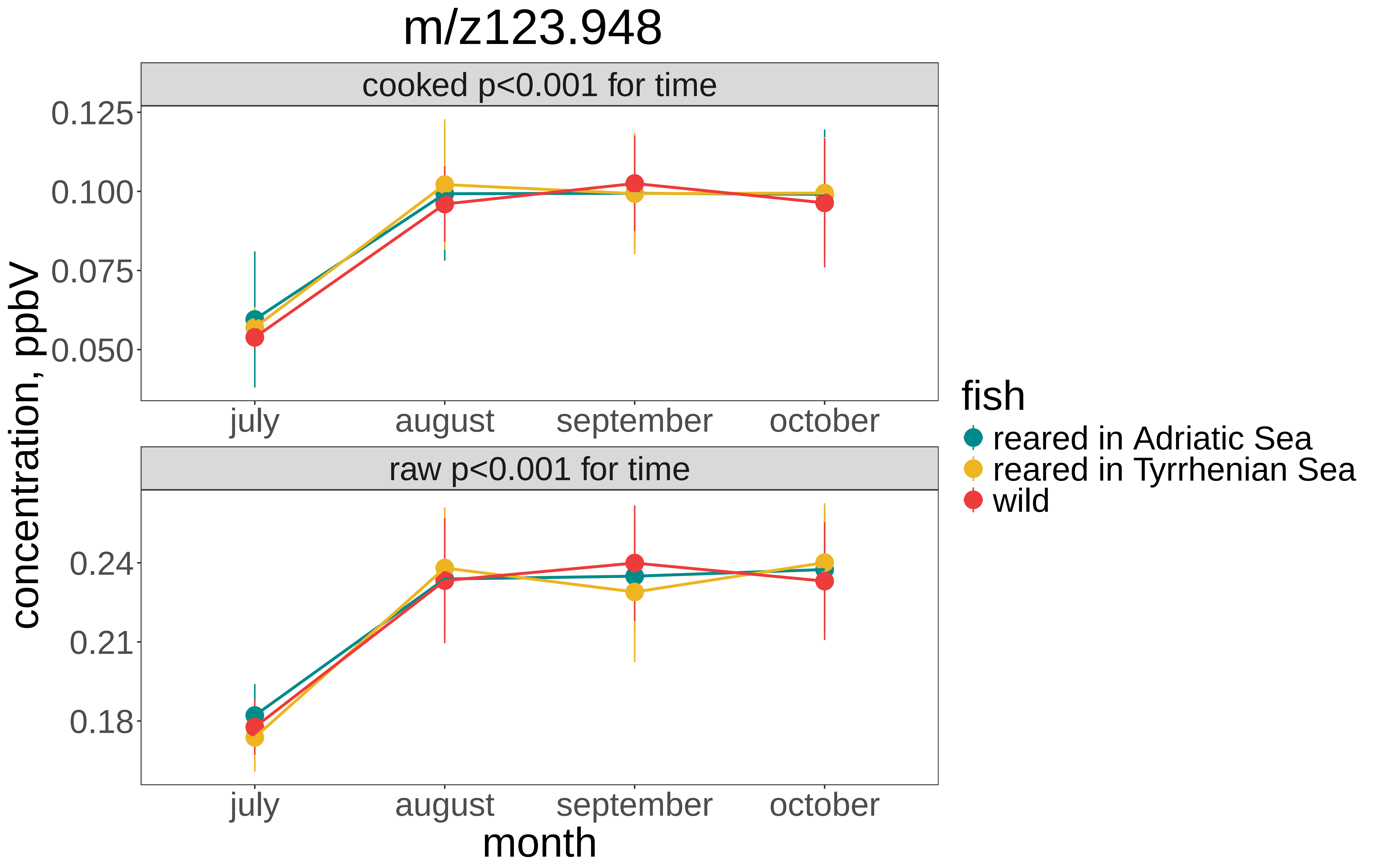

m/z123.948
cooked p<0.001 for time
raw p<0.001 for time
0.125
0.100
0.075
0.050
0.24
0.21
0.18
concentration, ppbV
july
august
september
october
month
fish
reared in Adriatic Sea
reared in Tyrrhenian Sea
wild

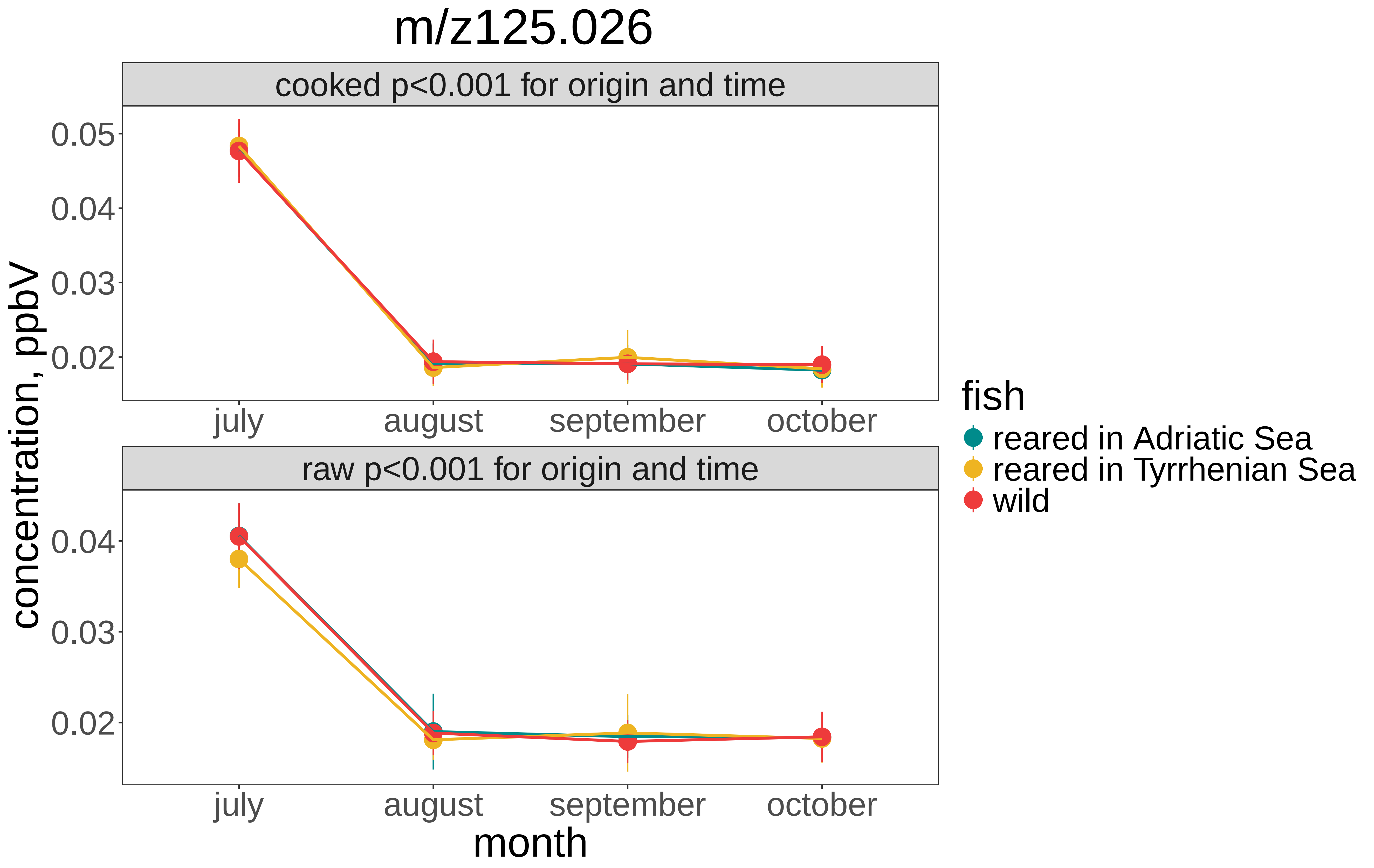

m/z125.026
cooked p<0.001 for origin and time
0.05
0.04
0.03
0.02
july
august
september
october
raw p<0.001 for origin and time
0.04
0.03
0.02
july
august
september
october
concentration, ppbV
month
fish
reared in Adriatic Sea
reared in Tyrrhenian Sea
wild

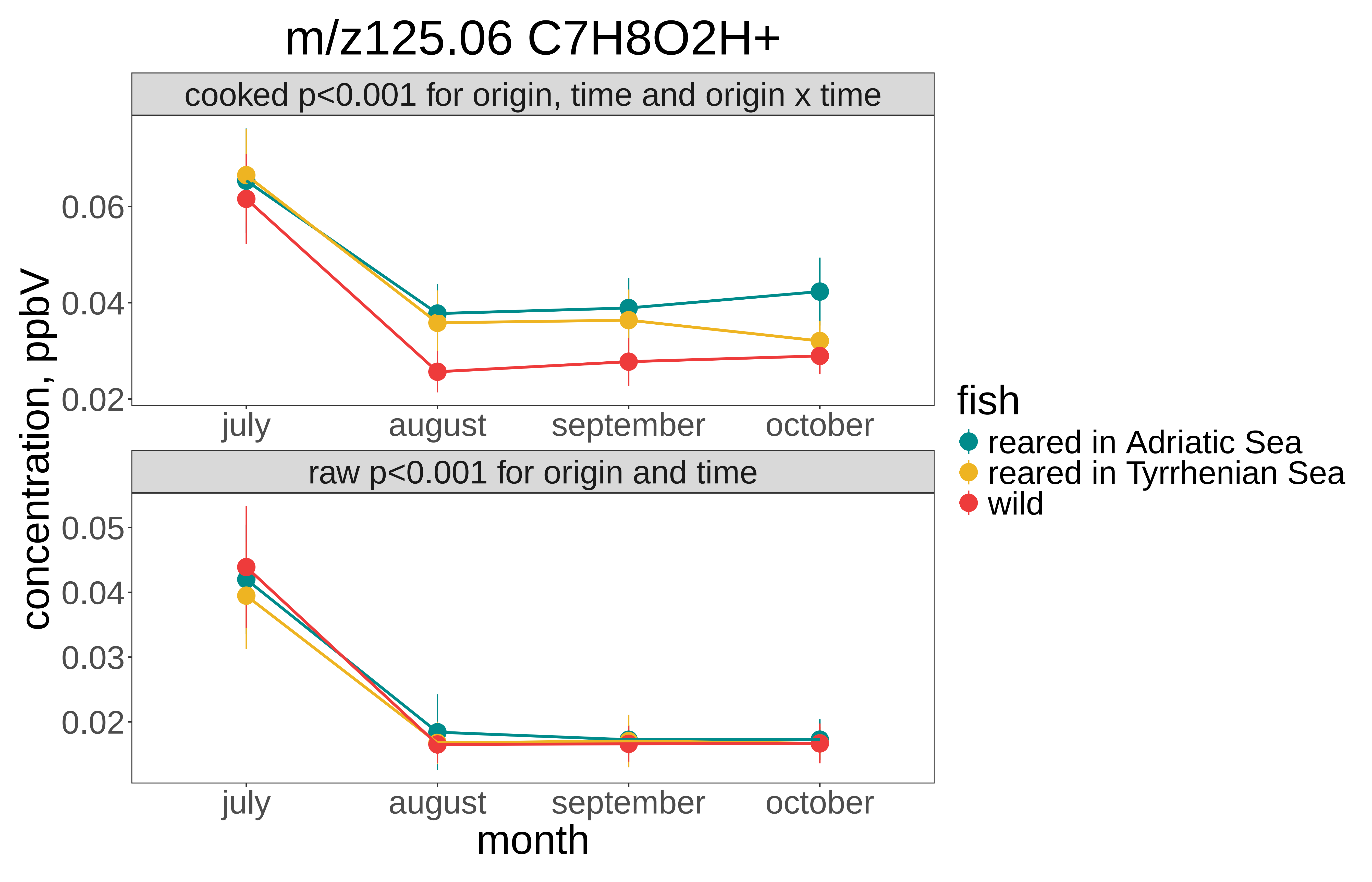
m/z125.06 C7H8O2H+
cooked p<0.001 for origin, time and origin x time
raw p<0.001 for origin and time
concentration, ppbV
0.06
0.04
0.02
0.05
0.04
0.03
0.02
july
august
september
october
month
fish
reared in Adriatic Sea
reared in Tyrrhenian Sea
wild

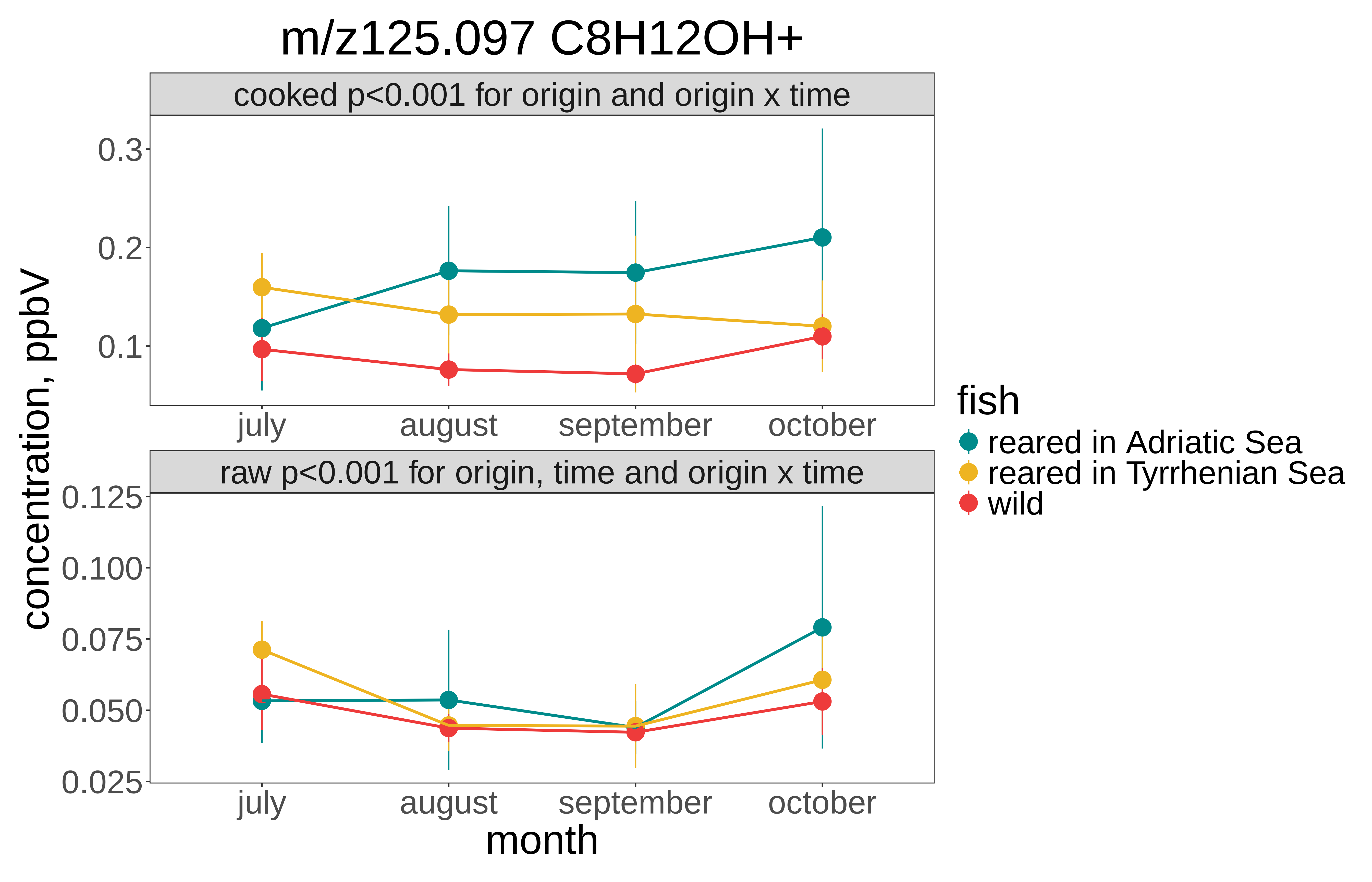
m/z125.097 C8H12OH+
cooked p<0.001 for origin and origin x time
raw p<0.001 for origin, time and origin x time
concentration, ppbV
0.3
0.2
0.1
0.125
0.100
0.075
0.050
0.025
july
august
september
october
month
fish
reared in Adriatic Sea
reared in Tyrrhenian Sea
wild

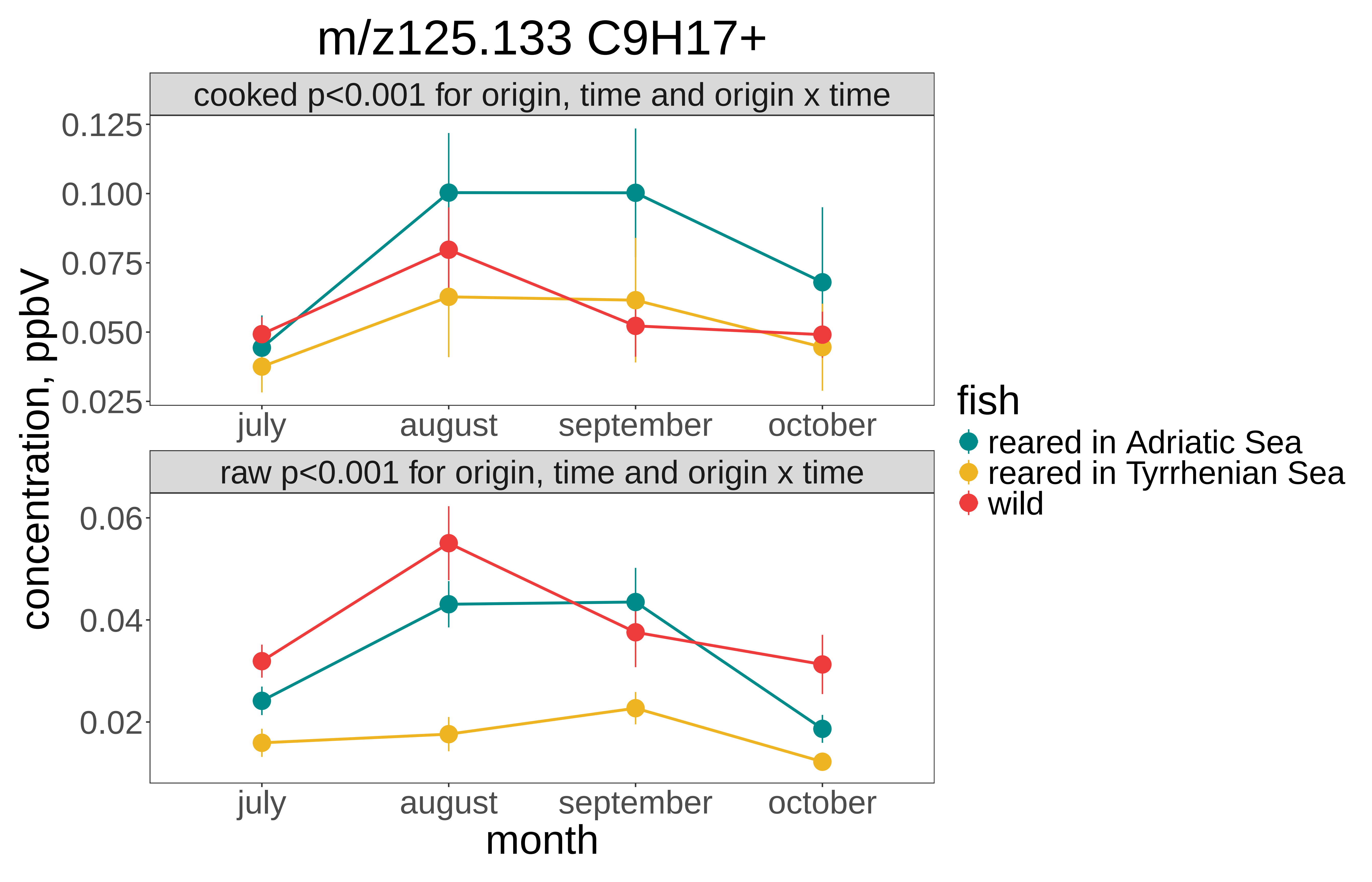

m/z125.133 C9H17+
cooked p<0.001 for origin, time and origin x time
raw p<0.001 for origin, time and origin x time
concentration, ppbV
0.125
0.100
0.075
0.050
0.025
0.06
0.04
0.02
july
august
september
october
month
fish
reared in Adriatic Sea
reared in Tyrrhenian Sea
wild

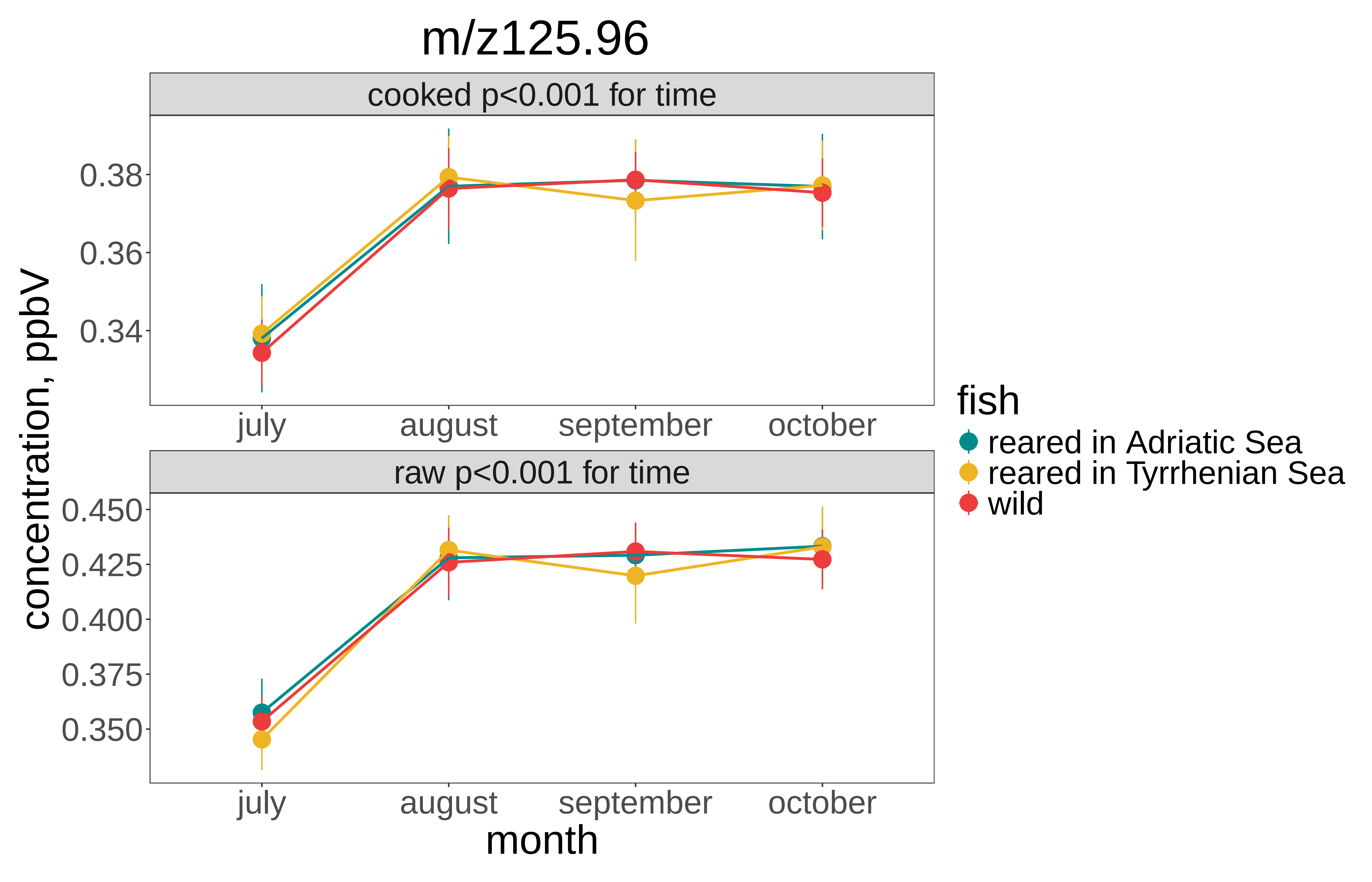

m/z125.96
cooked p<0.001 for time
raw p<0.001 for time
concentration, ppbV
0.38
0.36
0.34
0.450
0.425
0.400
0.375
0.350
july
august
september
october
month
fish
reared in Adriatic Sea
reared in Tyrrhenian Sea
wild

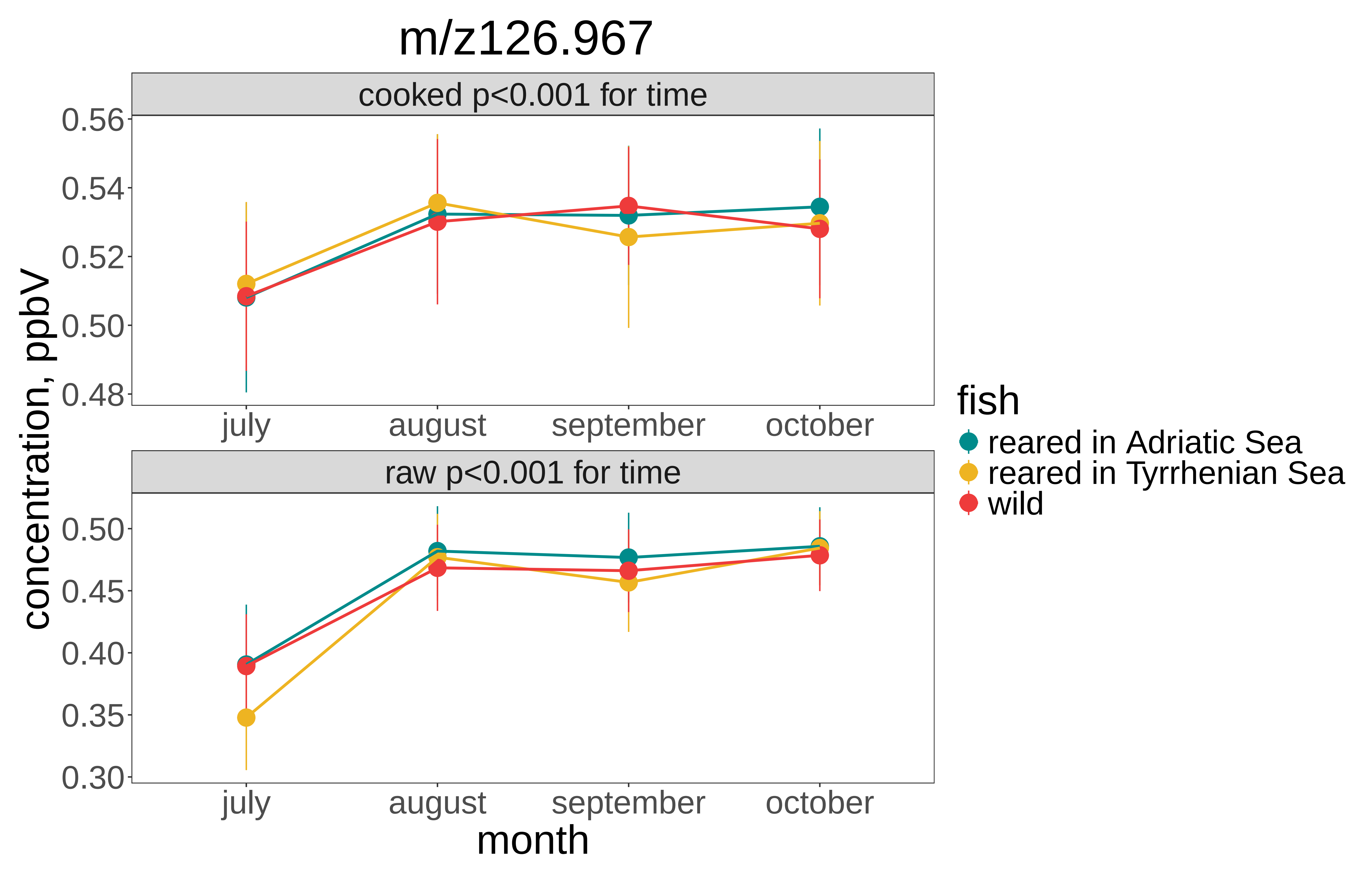

m/z126.967
cooked p<0.001 for time
raw p<0.001 for time
concentration, ppbV
0.56
0.54
0.52
0.50
0.48
0.50
0.45
0.40
0.35
0.30
july
august
september
october
month
fish
reared in Adriatic Sea
reared in Tyrrhenian Sea
wild

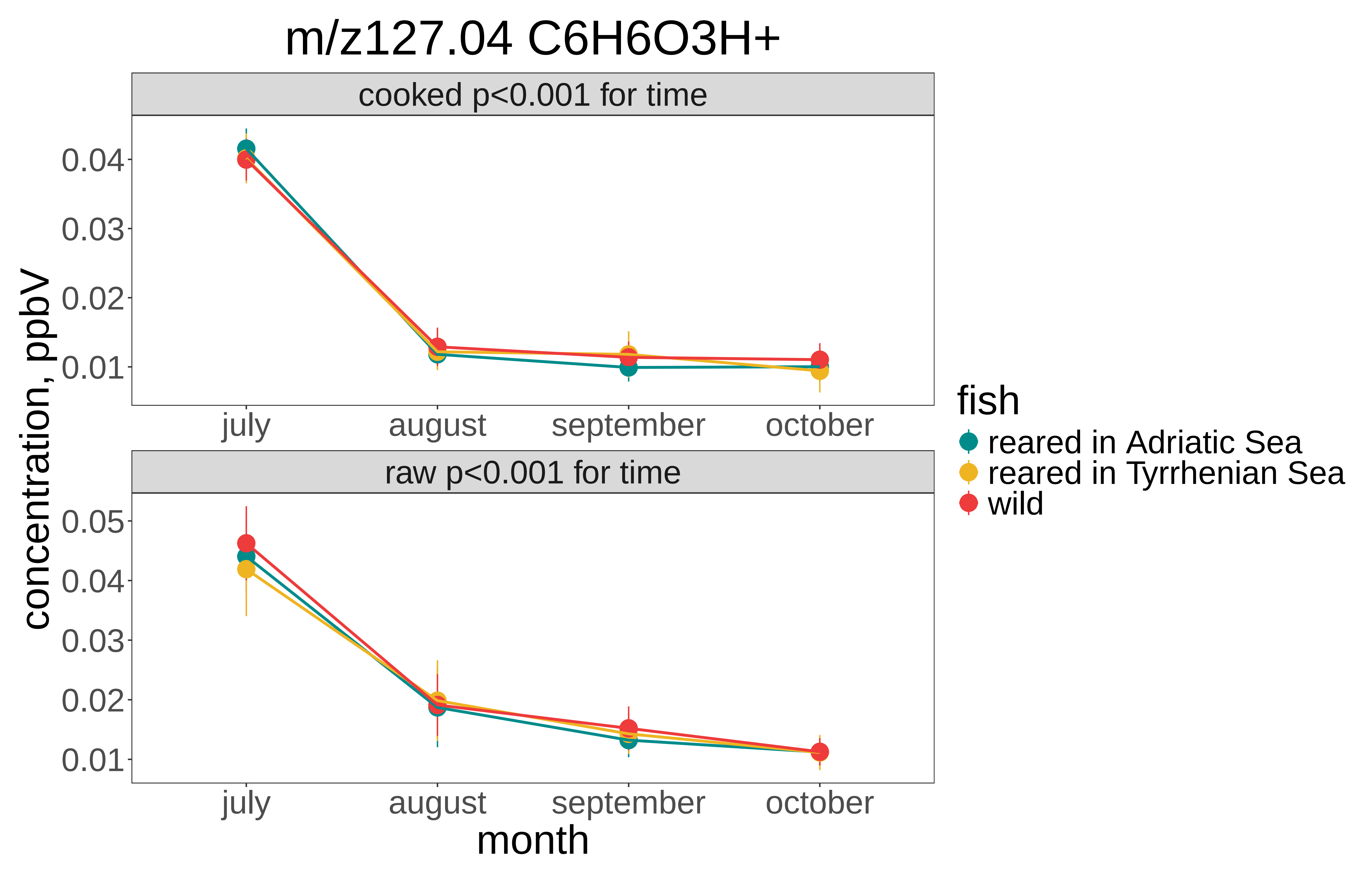
m/z127.04 C6H6O3H+
cooked p<0.001 for time
raw p<0.001 for time
concentration, ppbV
0.01
0.02
0.03
0.04
0.05
july
august
september
october
month
fish
reared in Adriatic Sea
reared in Tyrrhenian Sea
wild

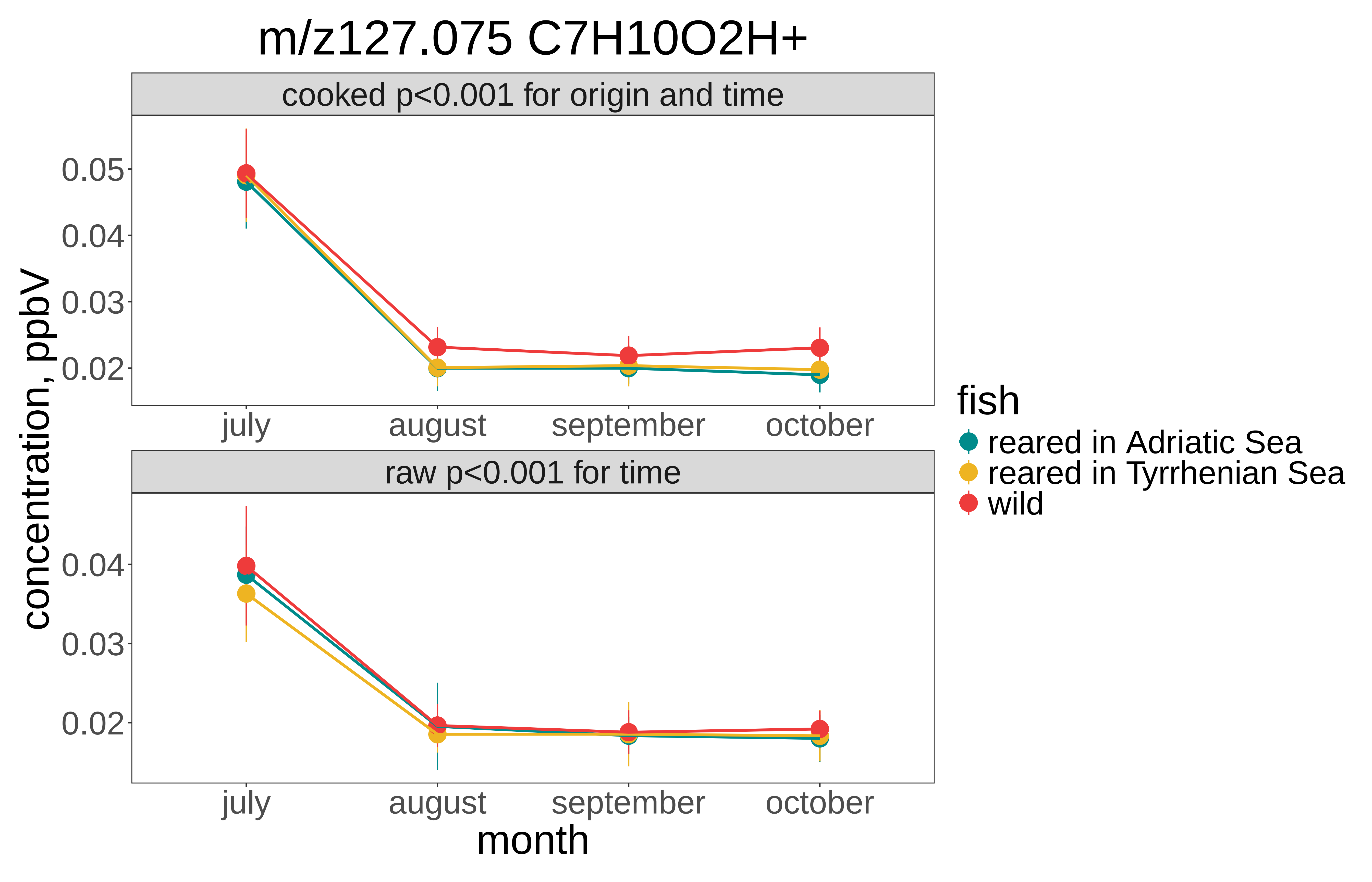
m/z127.075 C7H10O2H+
cooked p<0.001 for origin and time
raw p<0.001 for time
concentration, ppbV
0.05
0.04
0.03
0.02
0.04
0.03
0.02
july
august
september
october
july
august
september
october
month
fish
reared in Adriatic Sea
reared in Tyrrhenian Sea
wild

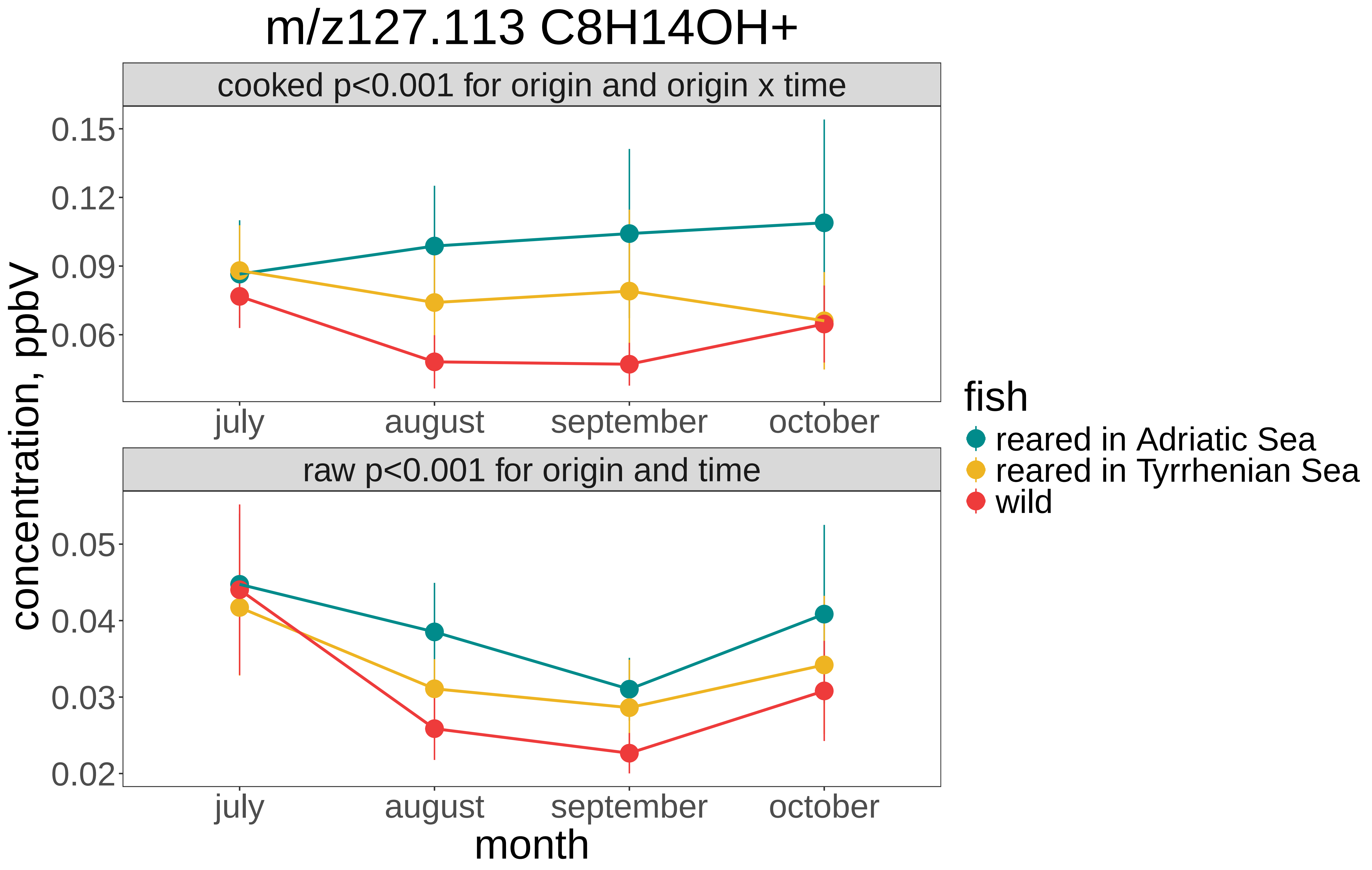
m/z127.113 C8H14OH+
cooked p<0.001 for origin and origin x time
raw p<0.001 for origin and time
concentration, ppbV
0.15
0.12
0.09
0.06
0.05
0.04
0.03
0.02
july
august
september
october
month
fish
reared in Adriatic Sea
reared in Tyrrhenian Sea
wild

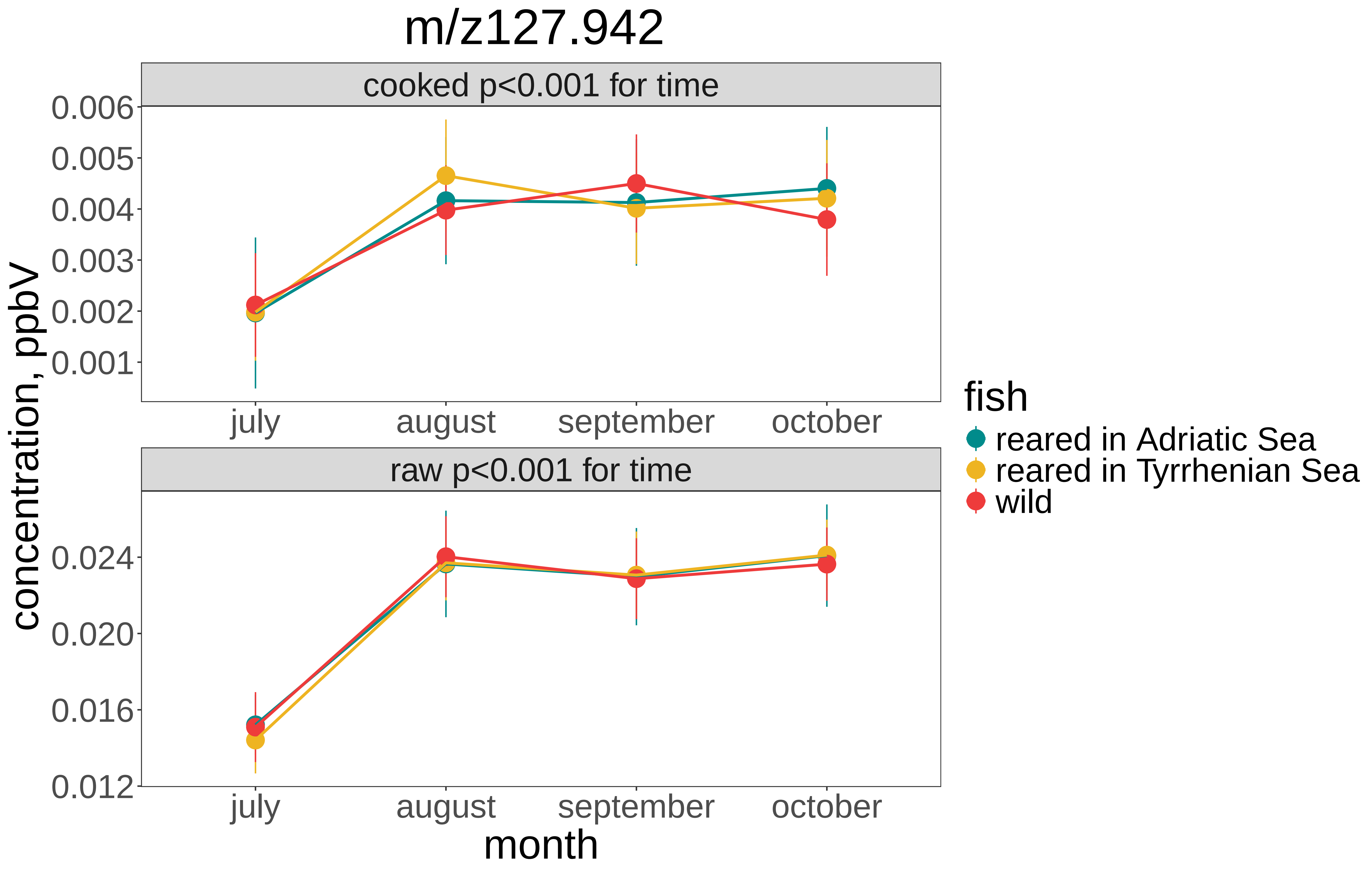
m/z127.942
cooked p<0.001 for time
raw p<0.001 for time
0.006
0.005
0.004
0.003
0.002
0.001
0.024
0.020
0.016
0.012
concentration, ppbV
july
august
september
october
month
fish
reared in Adriatic Sea
reared in Tyrrhenian Sea
wild

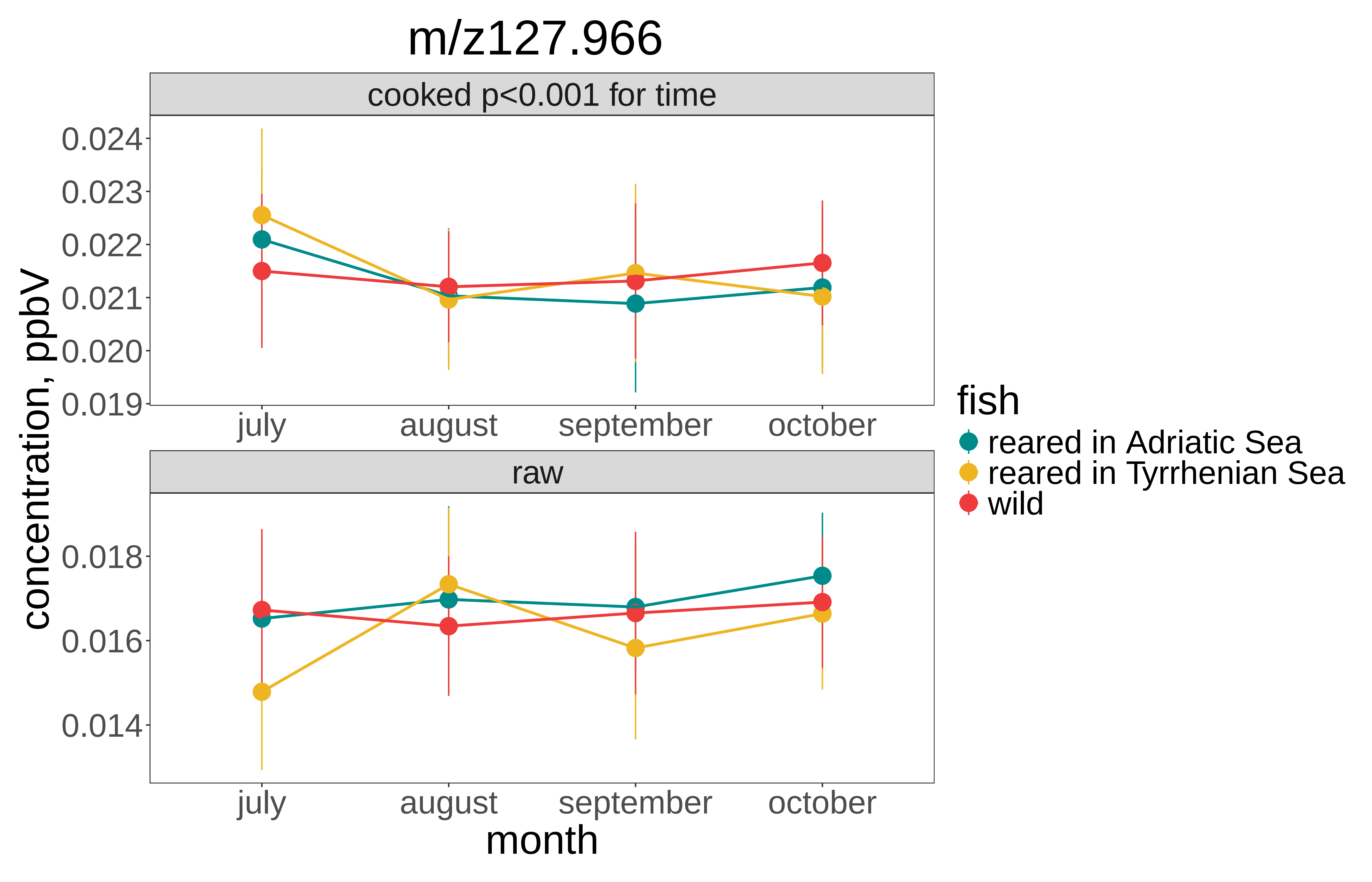
m/z127.966
cooked p<0.001 for time
raw
concentration, ppbV
0.024
0.023
0.022
0.021
0.020
0.019
0.018
0.016
0.014
july
august
september
october
month
fish
reared in Adriatic Sea
reared in Tyrrhenian Sea
wild

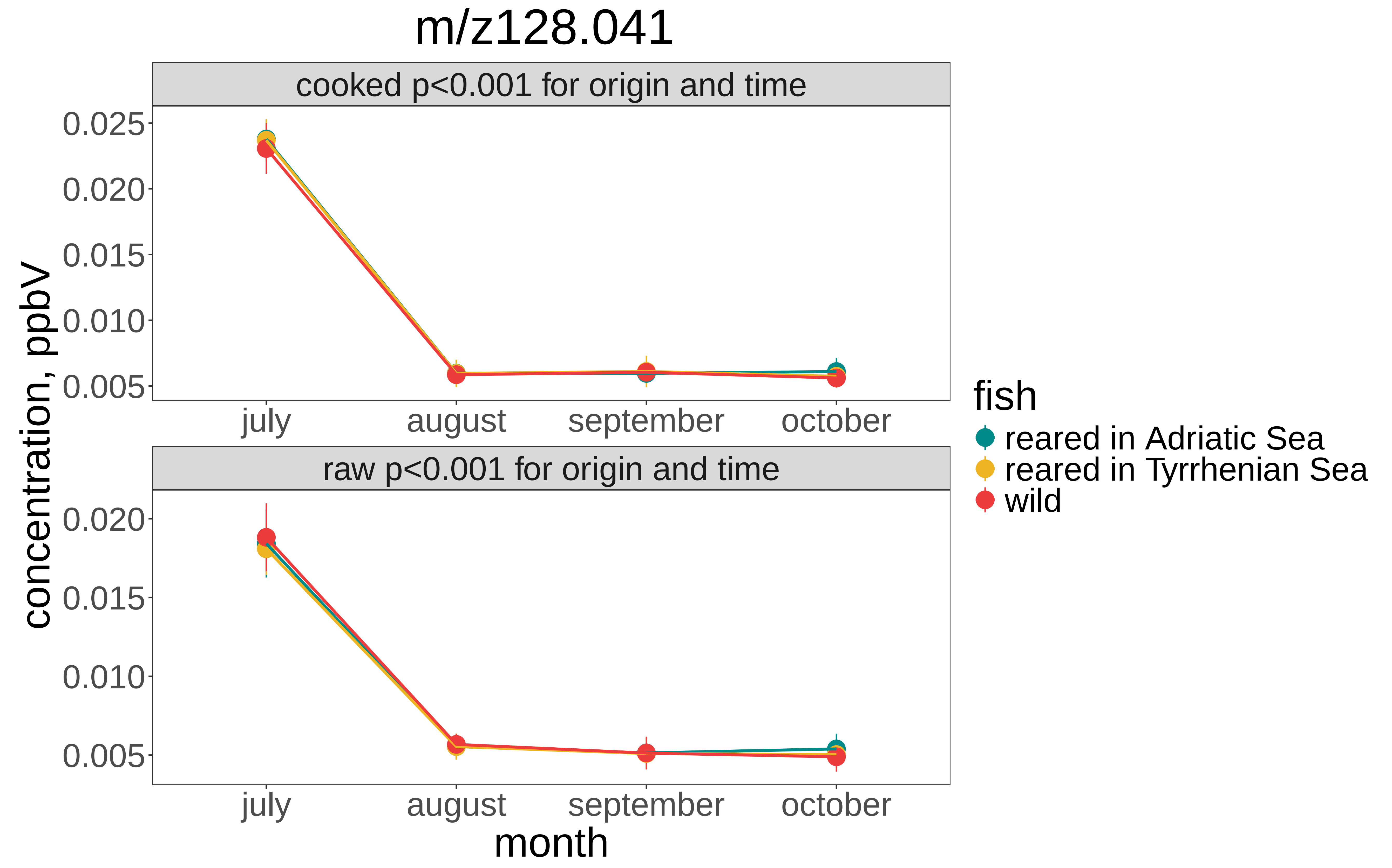
m/z128.041
cooked p<0.001 for origin and time
raw p<0.001 for origin and time
0.025
0.020
0.015
0.010
0.005
july
august
september
october
concentration, ppbV
month
fish
reared in Adriatic Sea
reared in Tyrrhenian Sea
wild

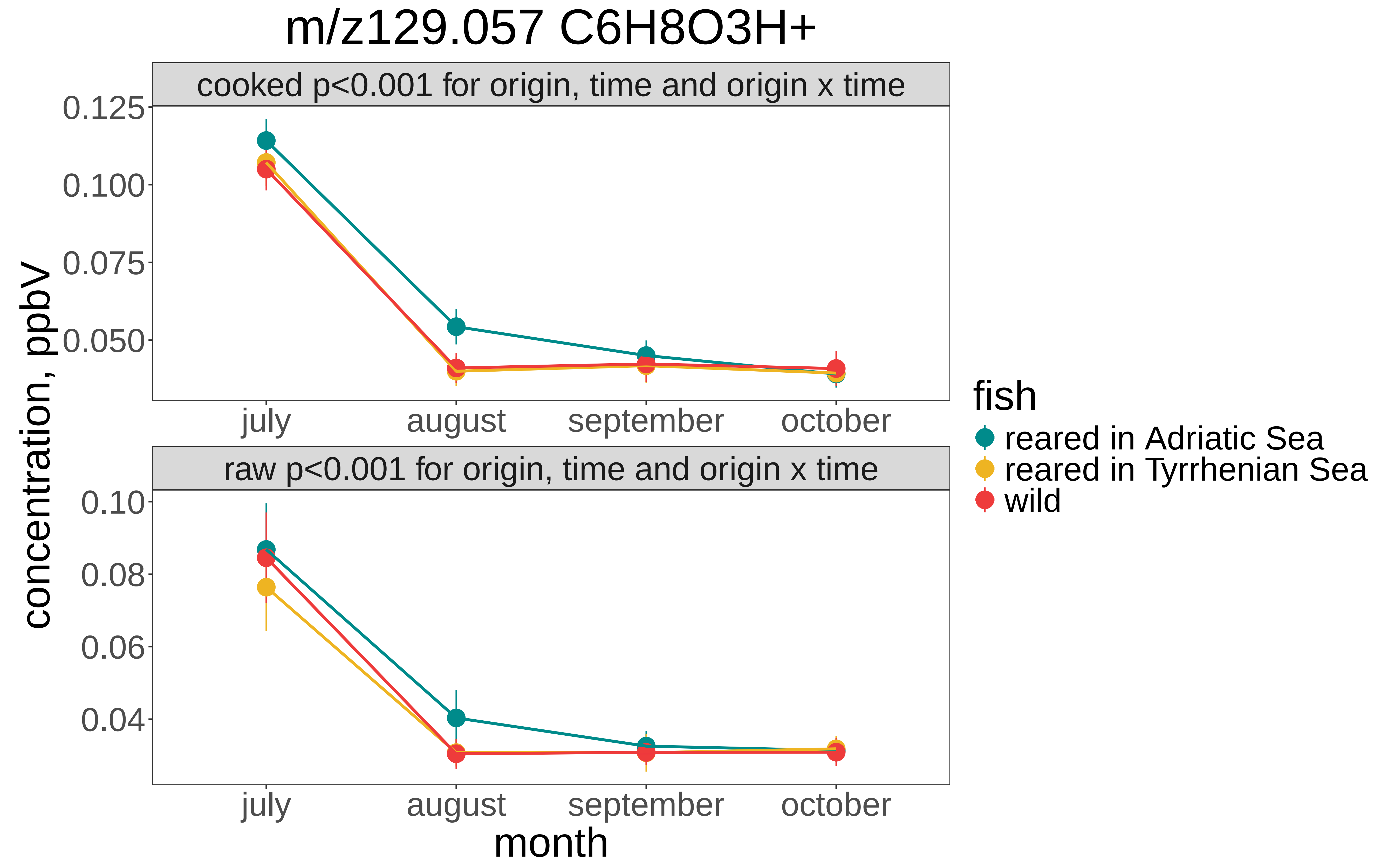

m/z129.057 C6H8O3H+
cooked p<0.001 for origin, time and origin x time
raw p<0.001 for origin, time and origin x time
concentration, ppbV
0.125
0.100
0.075
0.050
0.10
0.08
0.06
0.04
july
august
september
october
month
fish
reared in Adriatic Sea
reared in Tyrrhenian Sea
wild

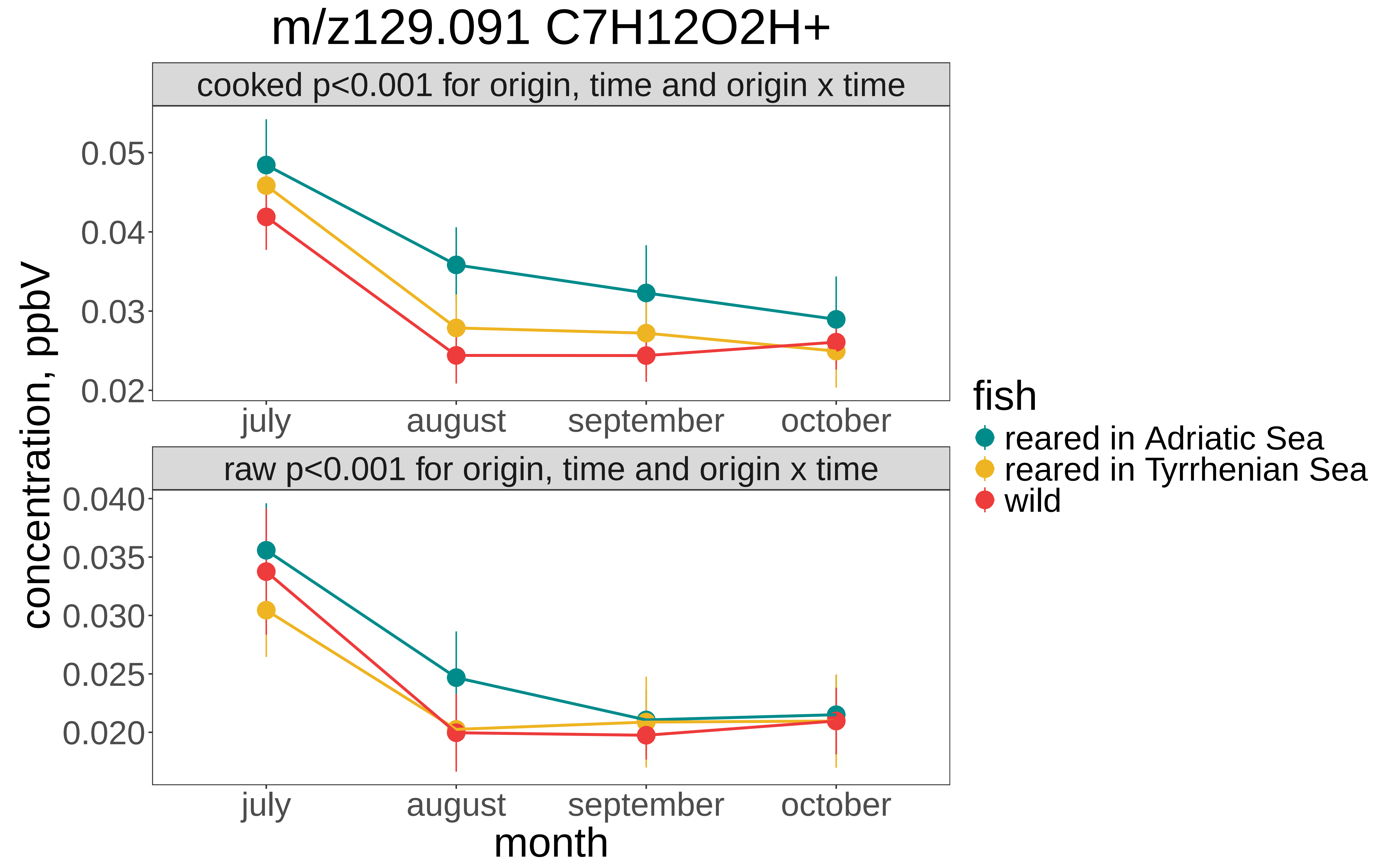

m/z129.091 C7H12O2H+
cooked p<0.001 for origin, time and origin x time
raw p<0.001 for origin, time and origin x time
concentration, ppbV
0.05
0.04
0.03
0.02
0.040
0.035
0.030
0.025
0.020
july
august
september
october
month
fish
reared in Adriatic Sea
reared in Tyrrhenian Sea
wild

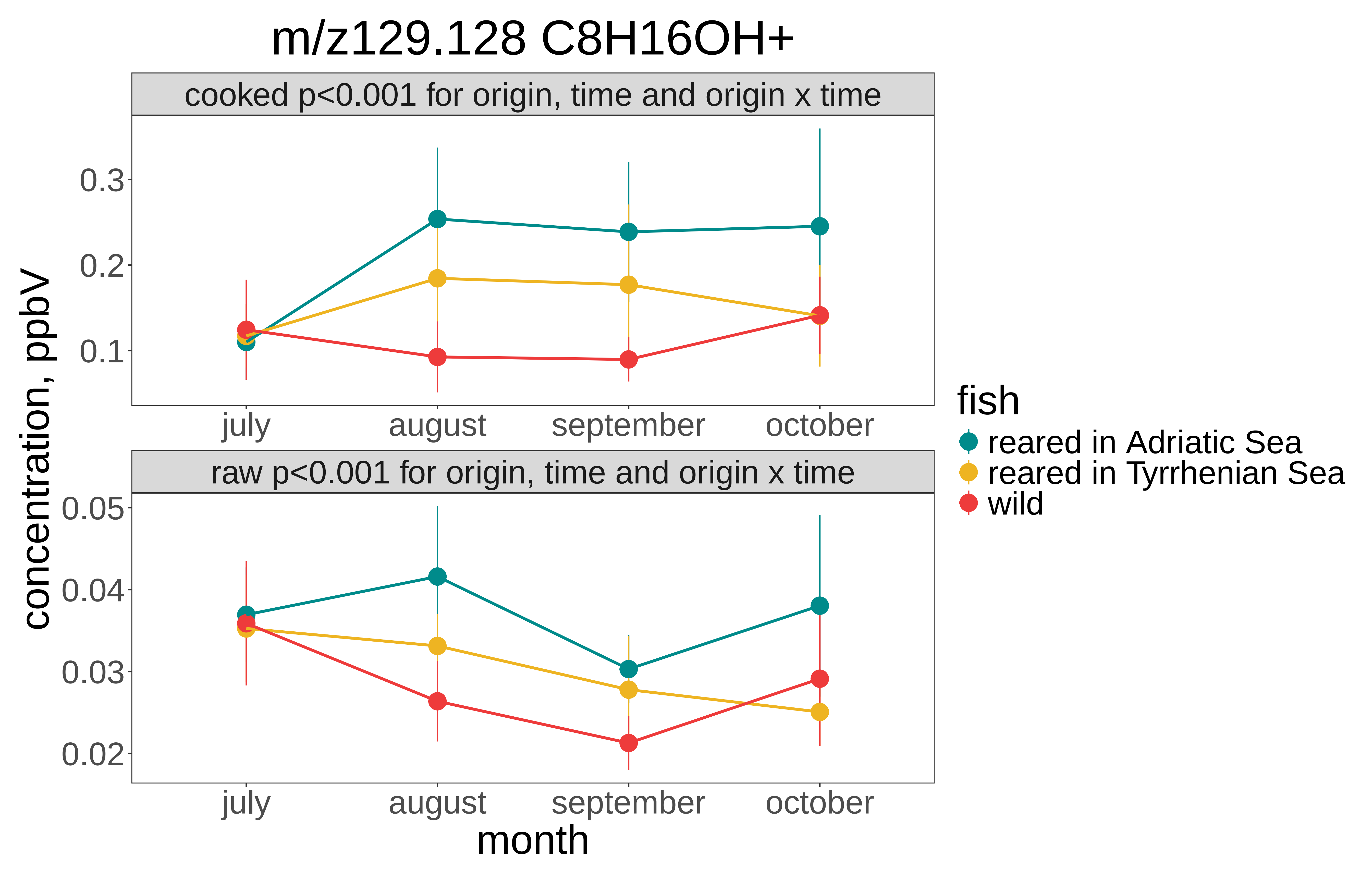
m/z129.128 C8H16OH+
cooked p<0.001 for origin, time and origin x time
raw p<0.001 for origin, time and origin x time
concentration, ppbV
0.3
0.2
0.1
0.05
0.04
0.03
0.02
july
august
september
october
month
fish
reared in Adriatic Sea
reared in Tyrrhenian Sea
wild

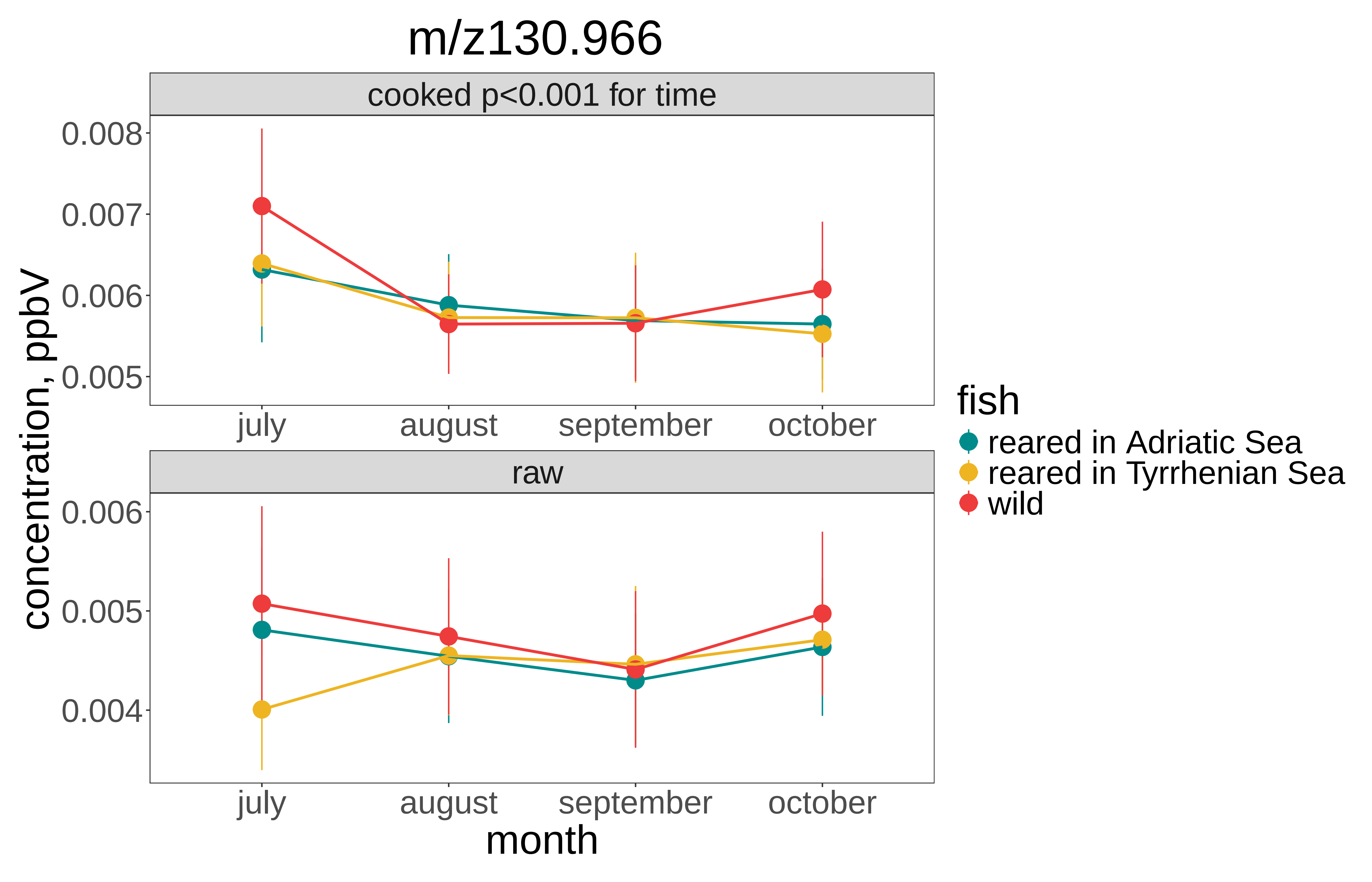
m/z130.966
cooked p<0.001 for time
raw
0.008
0.007
0.006
0.005
0.004
july
august
september
october
concentration, ppbV
month
fish
reared in Adriatic Sea
reared in Tyrrhenian Sea
wild

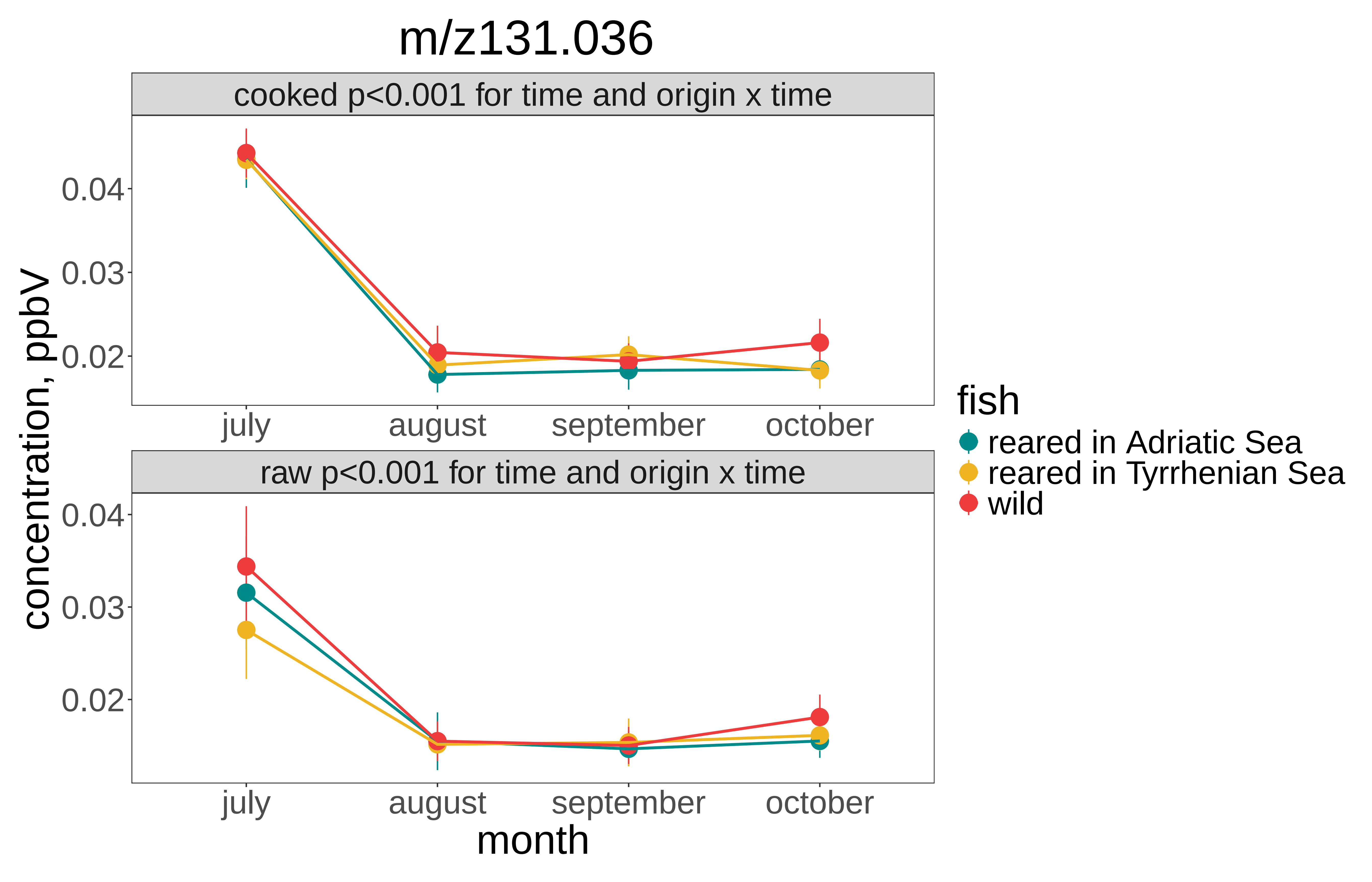
m/z131.036
cooked p<0.001 for time and origin x time
raw p<0.001 for time and origin x time
0.04
0.03
0.02
concentration, ppbV
july
august
september
october
month
fish
reared in Adriatic Sea
reared in Tyrrhenian Sea
wild

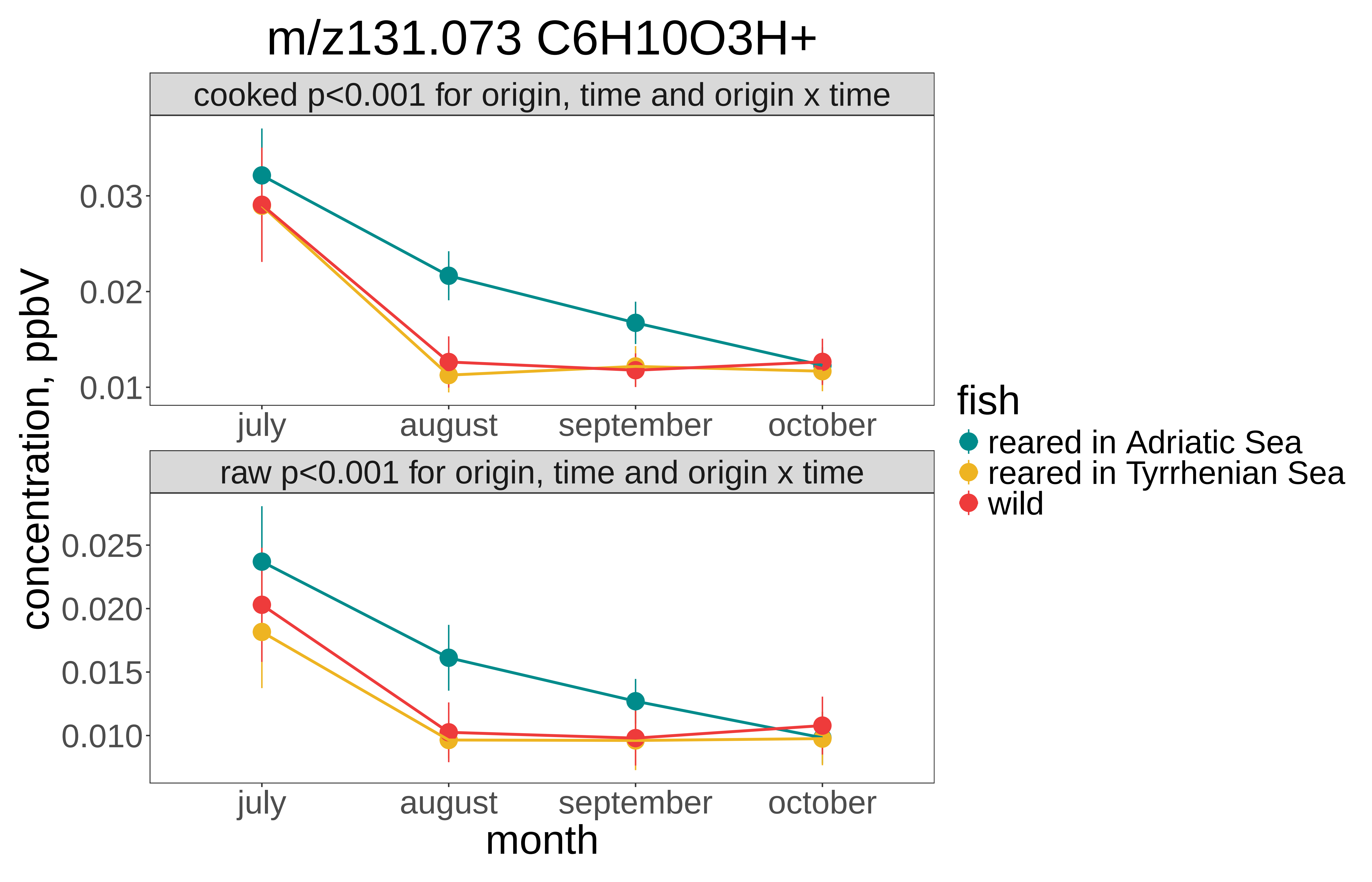
m/z131.073 C6H10O3H+
cooked p<0.001 for origin, time and origin x time
raw p<0.001 for origin, time and origin x time
concentration, ppbV
0.03
0.02
0.01
0.025
0.020
0.015
0.010
july
august
september
october
month
fish
reared in Adriatic Sea
reared in Tyrrhenian Sea
wild

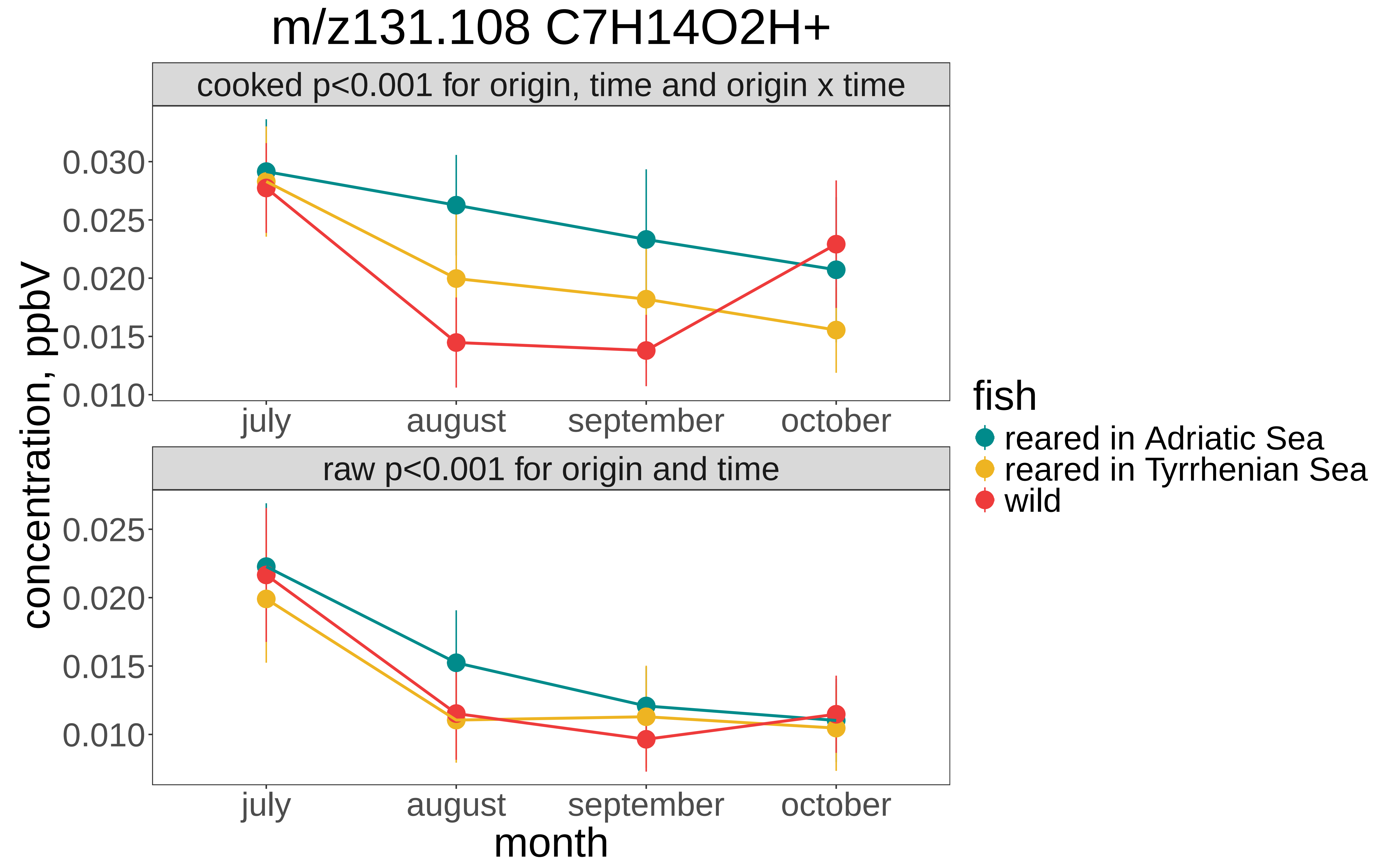

m/z131.108 C7H14O2H+
cooked p<0.001 for origin, time and origin x time
raw p<0.001 for origin and time
0.030
0.025
0.020
0.015
0.010
july
august
september
october
concentration, ppbV
month
fish
reared in Adriatic Sea
reared in Tyrrhenian Sea
wild

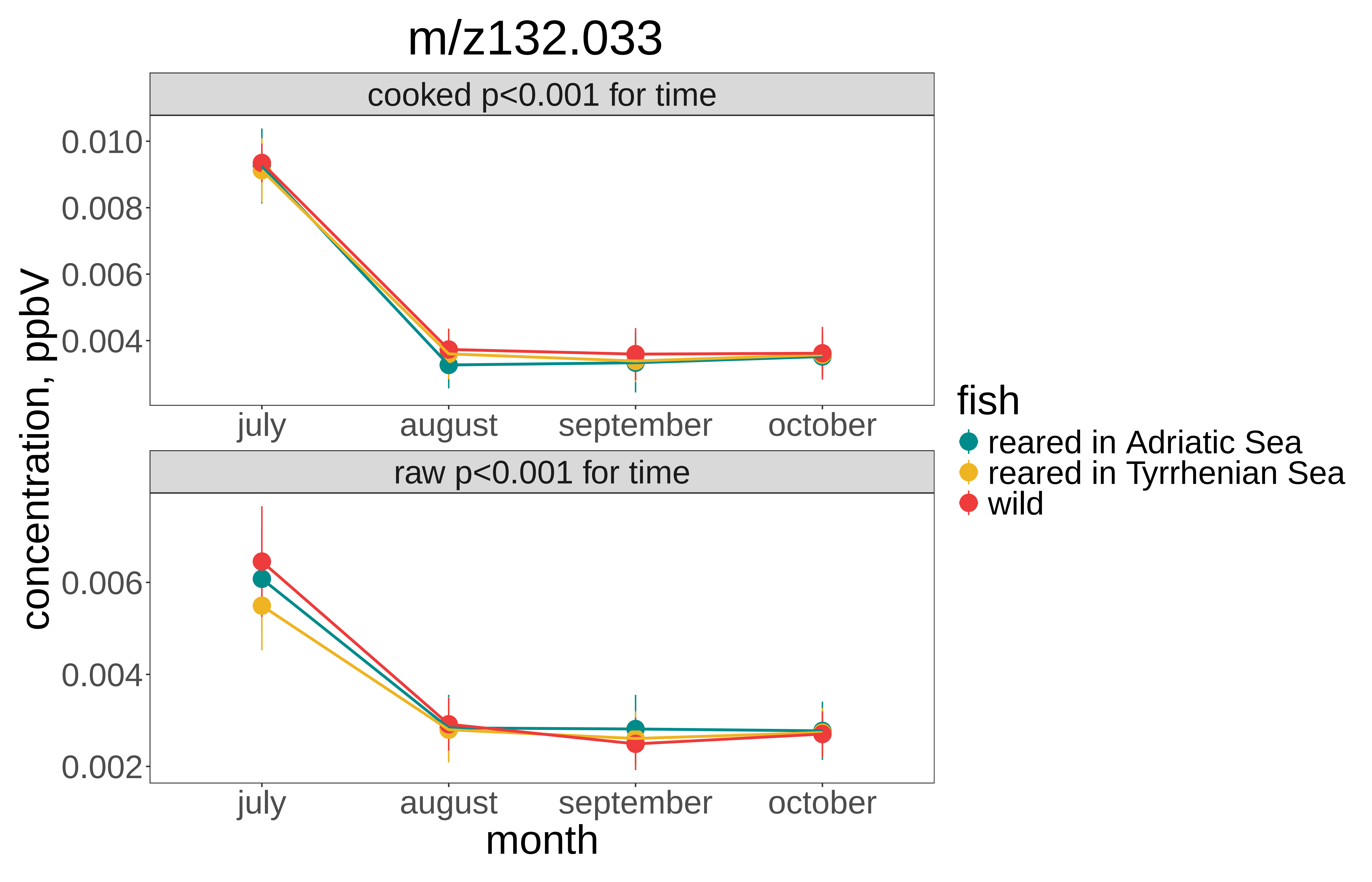

m/z132.033
cooked p<0.001 for time
raw p<0.001 for time
0.010
0.008
0.006
0.004
0.002
concentration, ppbV
july
august
september
october
month
fish
reared in Adriatic Sea
reared in Tyrrhenian Sea
wild

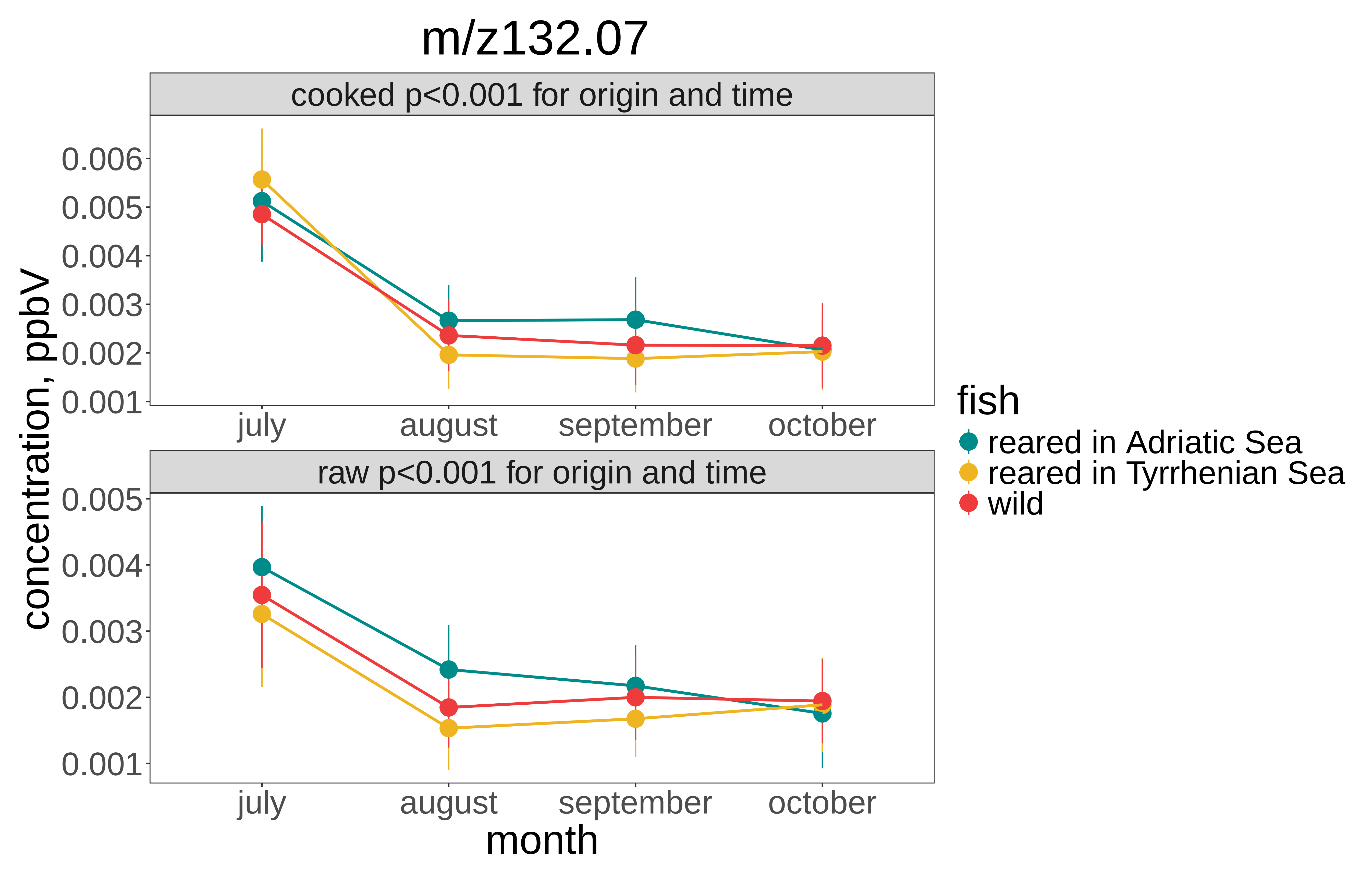
m/z132.07
cooked p<0.001 for origin and time
raw p<0.001 for origin and time
concentration, ppbV
0.006
0.005
0.004
0.003
0.002
0.001
july
august
september
october
month
fish
reared in Adriatic Sea
reared in Tyrrhenian Sea
wild

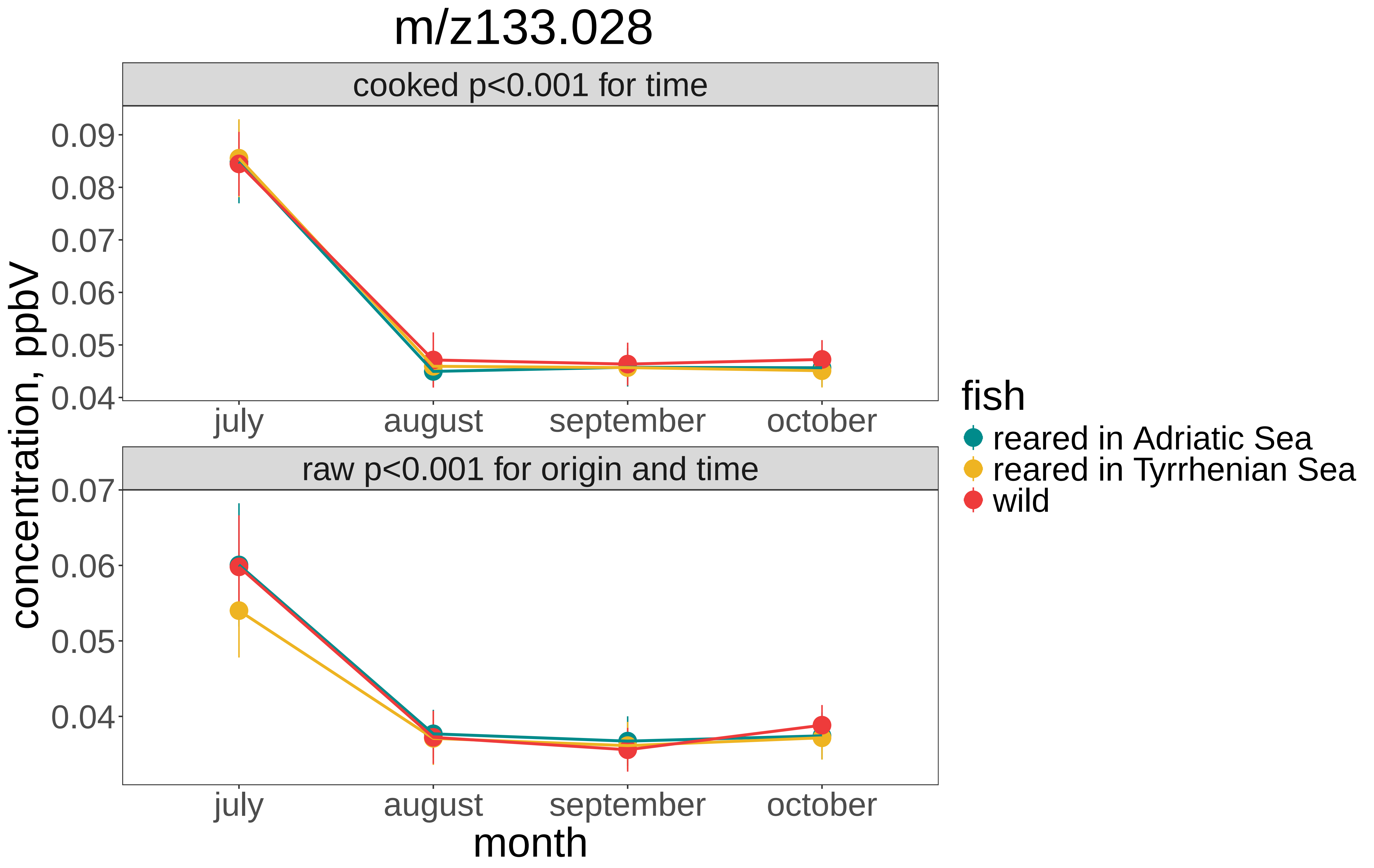
m/z133.028
cooked p<0.001 for time
raw p<0.001 for origin and time
concentration, ppbV
0.09
0.08
0.07
0.06
0.05
0.04
july
august
september
october
month
fish
reared in Adriatic Sea
reared in Tyrrhenian Sea
wild

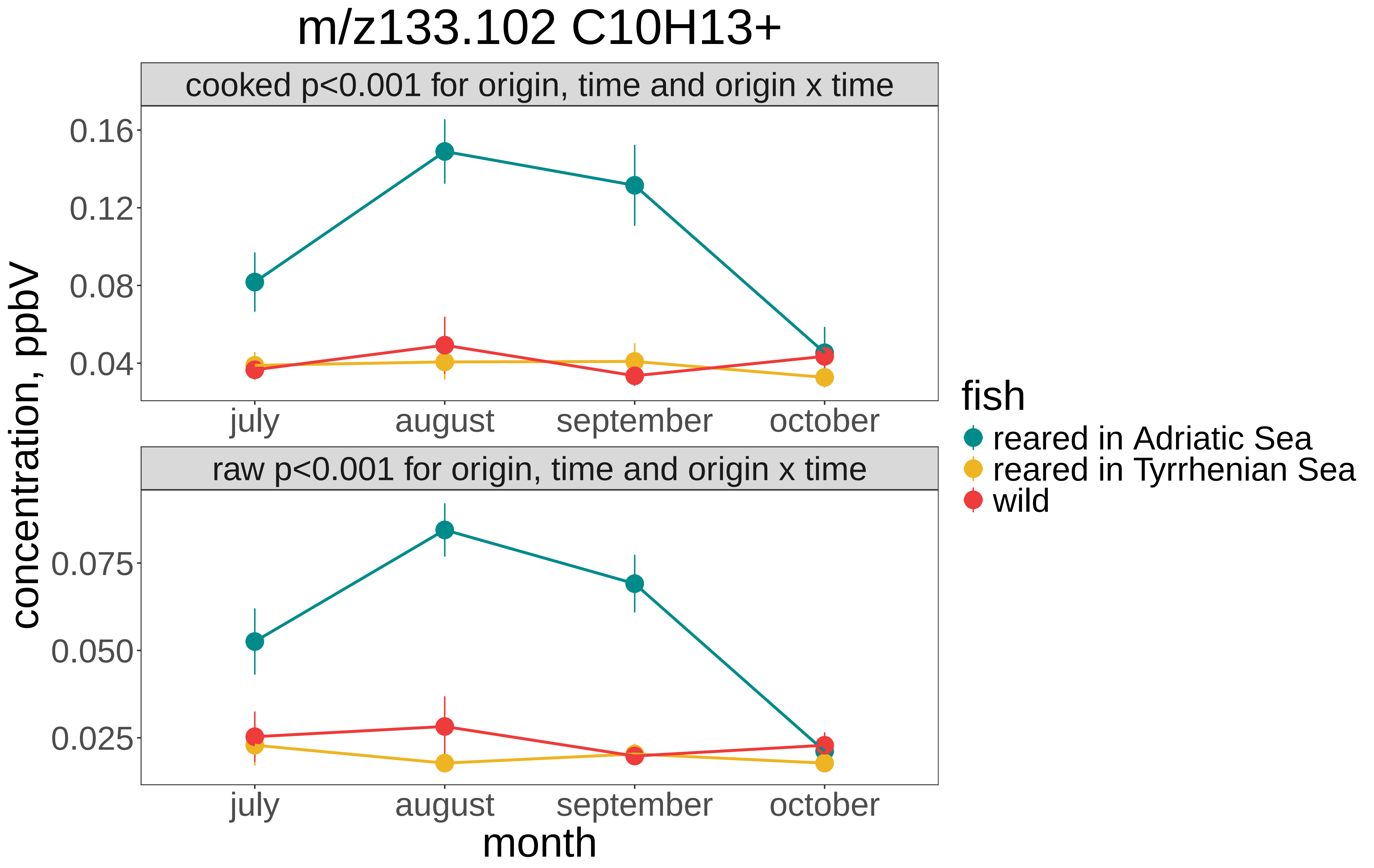
m/z133.102 C10H13+
cooked p<0.001 for origin, time and origin x time
raw p<0.001 for origin, time and origin x time
concentration, ppbV
0.16
0.12
0.08
0.04
0.075
0.050
0.025
july
august
september
october
month
fish
reared in Adriatic Sea
reared in Tyrrhenian Sea
wild

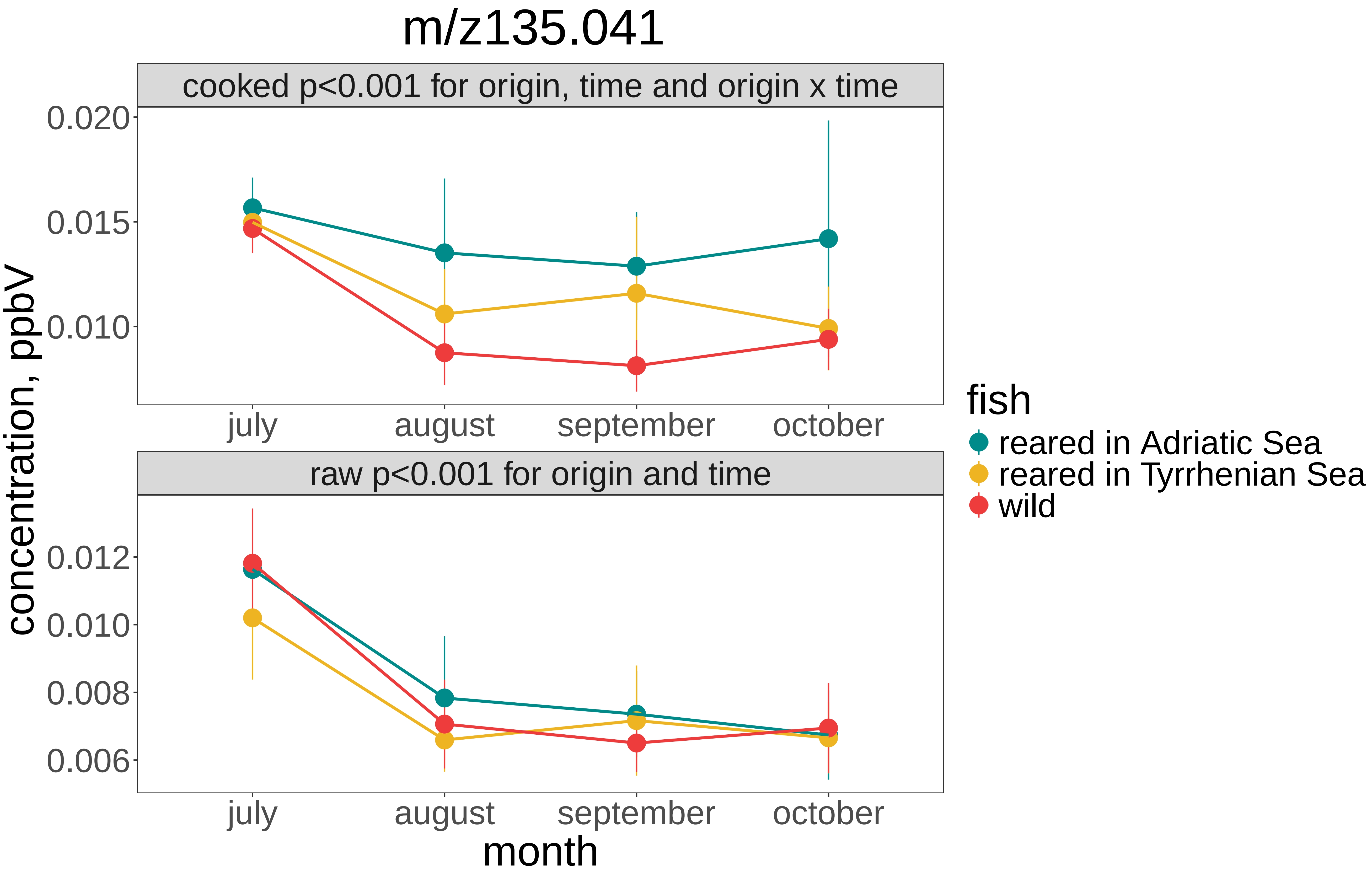

m/z135.041
cooked p<0.001 for origin, time and origin x time
raw p<0.001 for origin and time
concentration, ppbV
0.020
0.015
0.010
0.012
0.010
0.008
0.006
july
august
september
october
month
fish
reared in Adriatic Sea
reared in Tyrrhenian Sea
wild

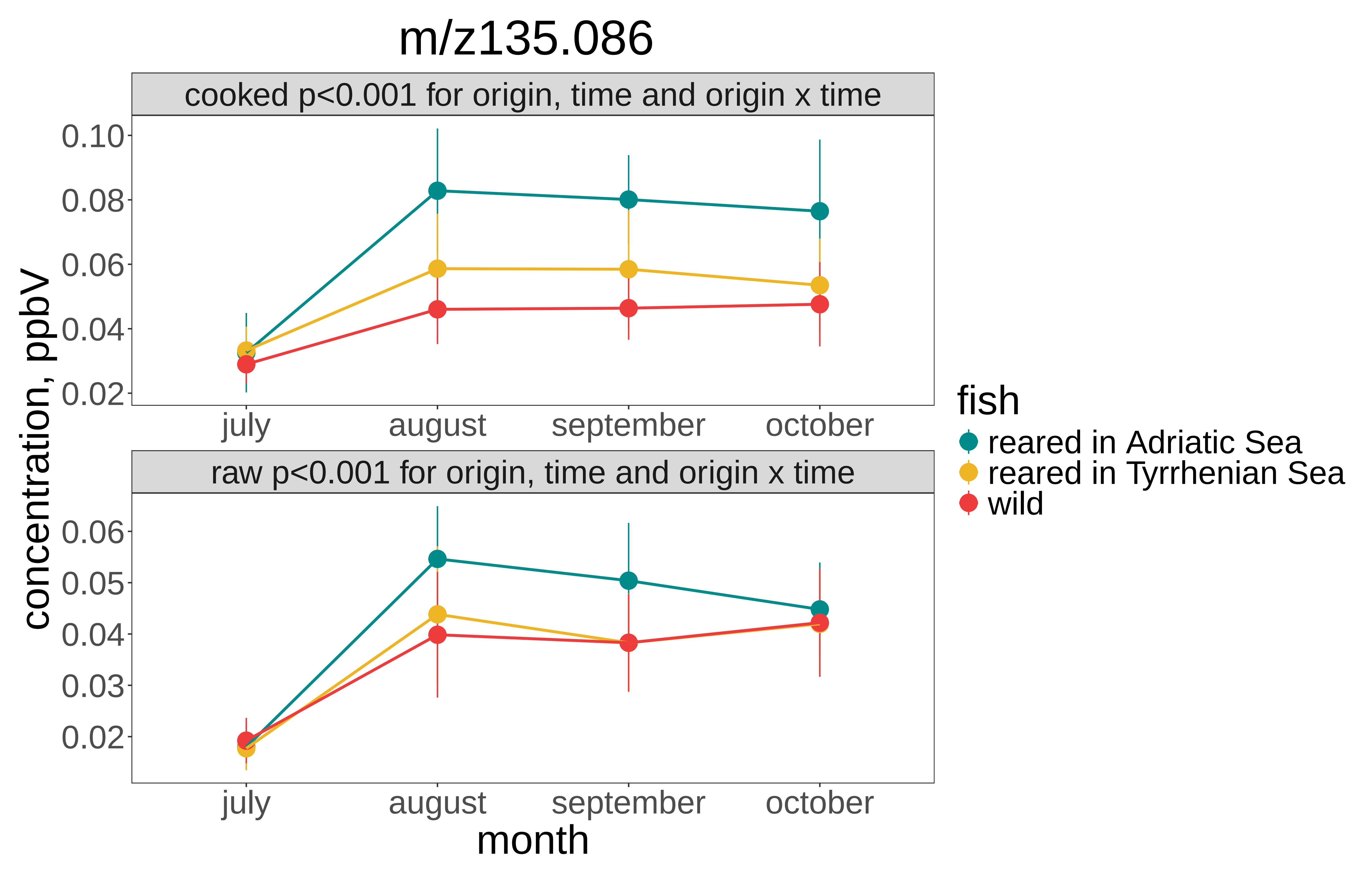
m/z135.086
cooked p<0.001 for origin, time and origin x time
raw p<0.001 for origin, time and origin x time
concentration, ppbV
0.10
0.08
0.06
0.04
0.02
0.05
0.03
july
august
september
october
month
fish
reared in Adriatic Sea
reared in Tyrrhenian Sea
wild

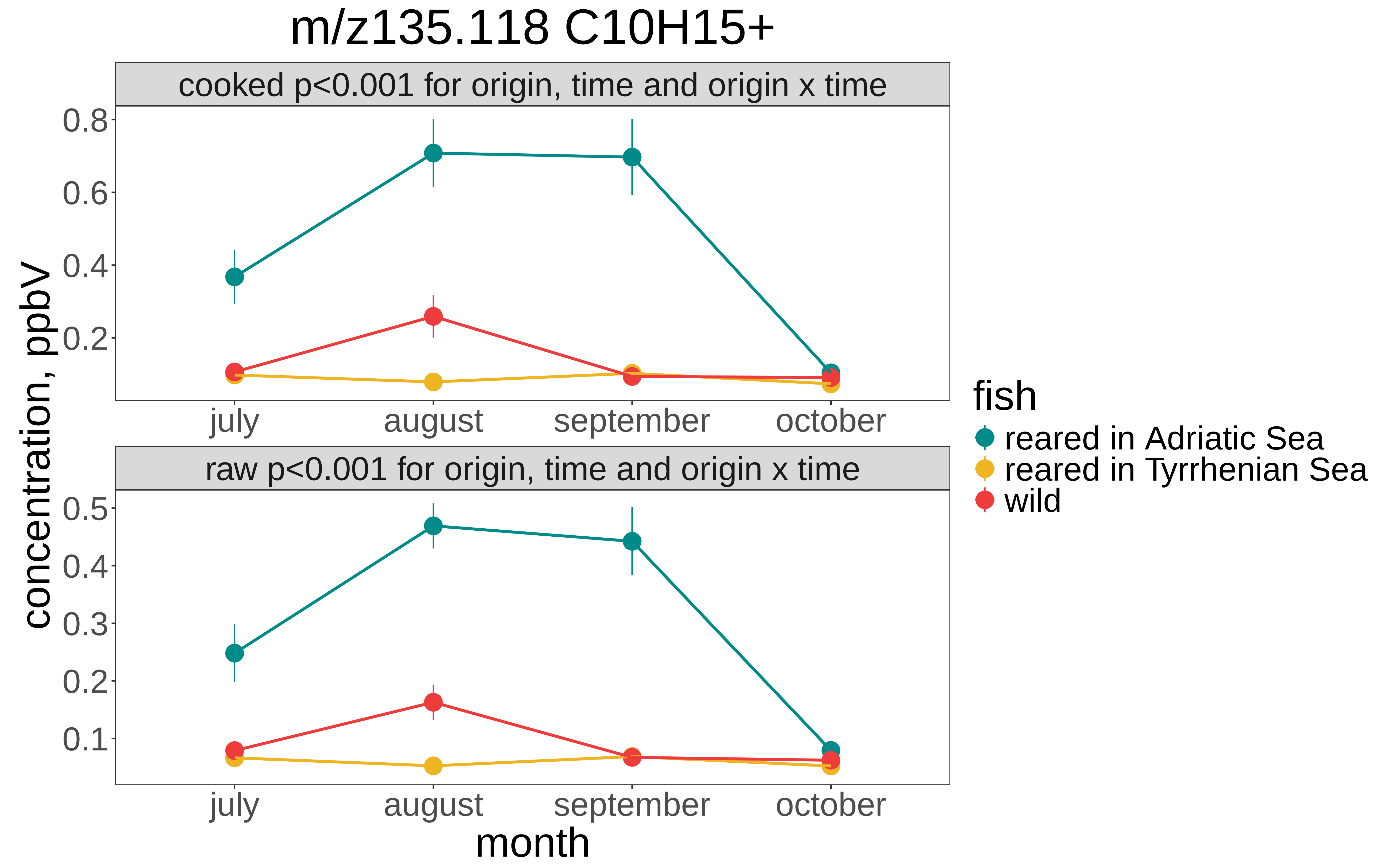
m/z135.118 C10H15+
cooked p<0.001 for origin, time and origin x time
raw p<0.001 for origin, time and origin x time
concentration, ppbV
0.8
0.6
0.4
0.2
0.5
0.4
0.3
0.2
0.1
july
august
september
october
month
fish
reared in Adriatic Sea
reared in Tyrrhenian Sea
wild

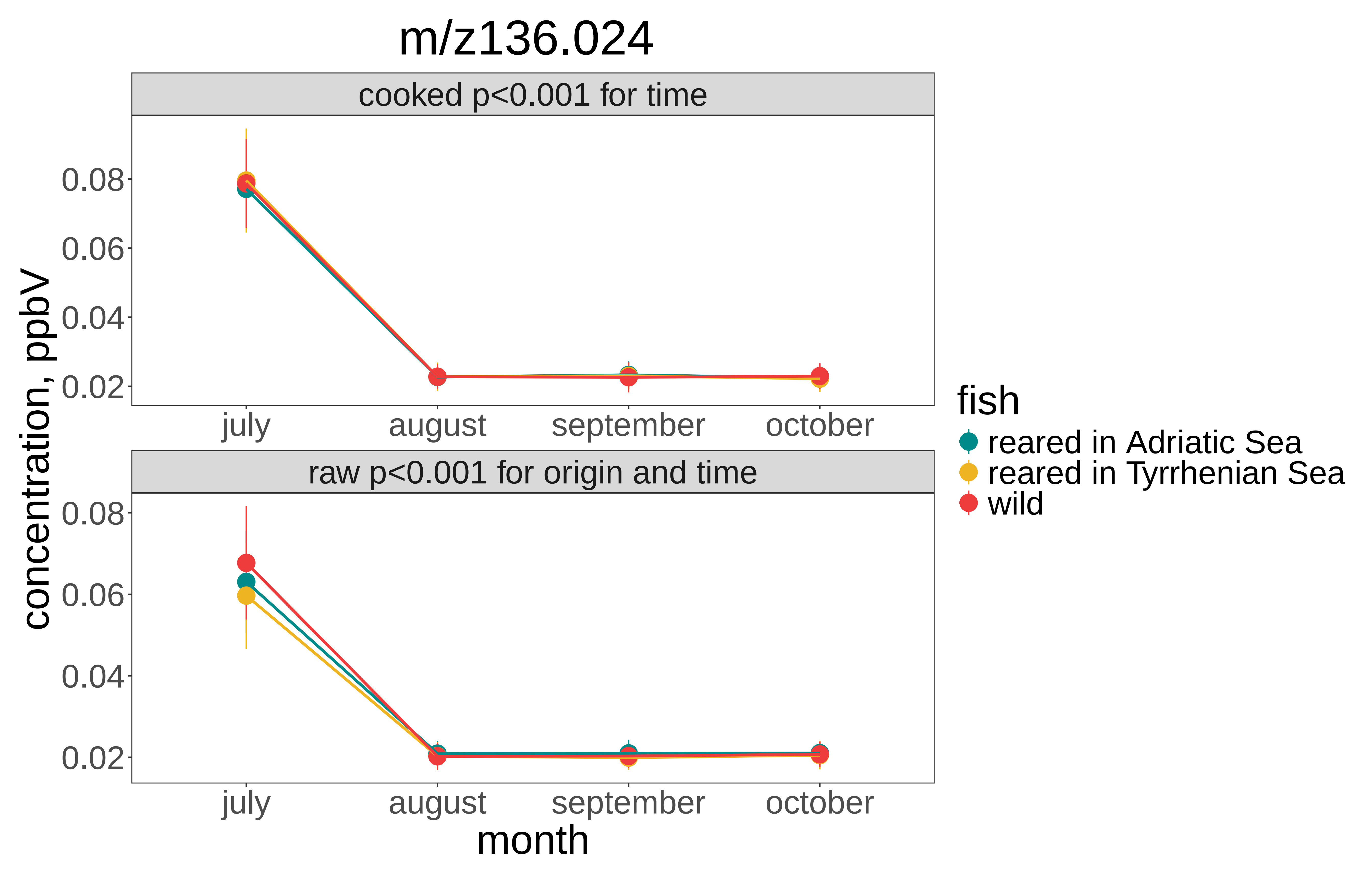
m/z136.024
cooked p<0.001 for time
raw p<0.001 for origin and time
concentration, ppbV
0.08
0.06
0.04
0.02
july
august
september
october
month
fish
reared in Adriatic Sea
reared in Tyrrhenian Sea
wild

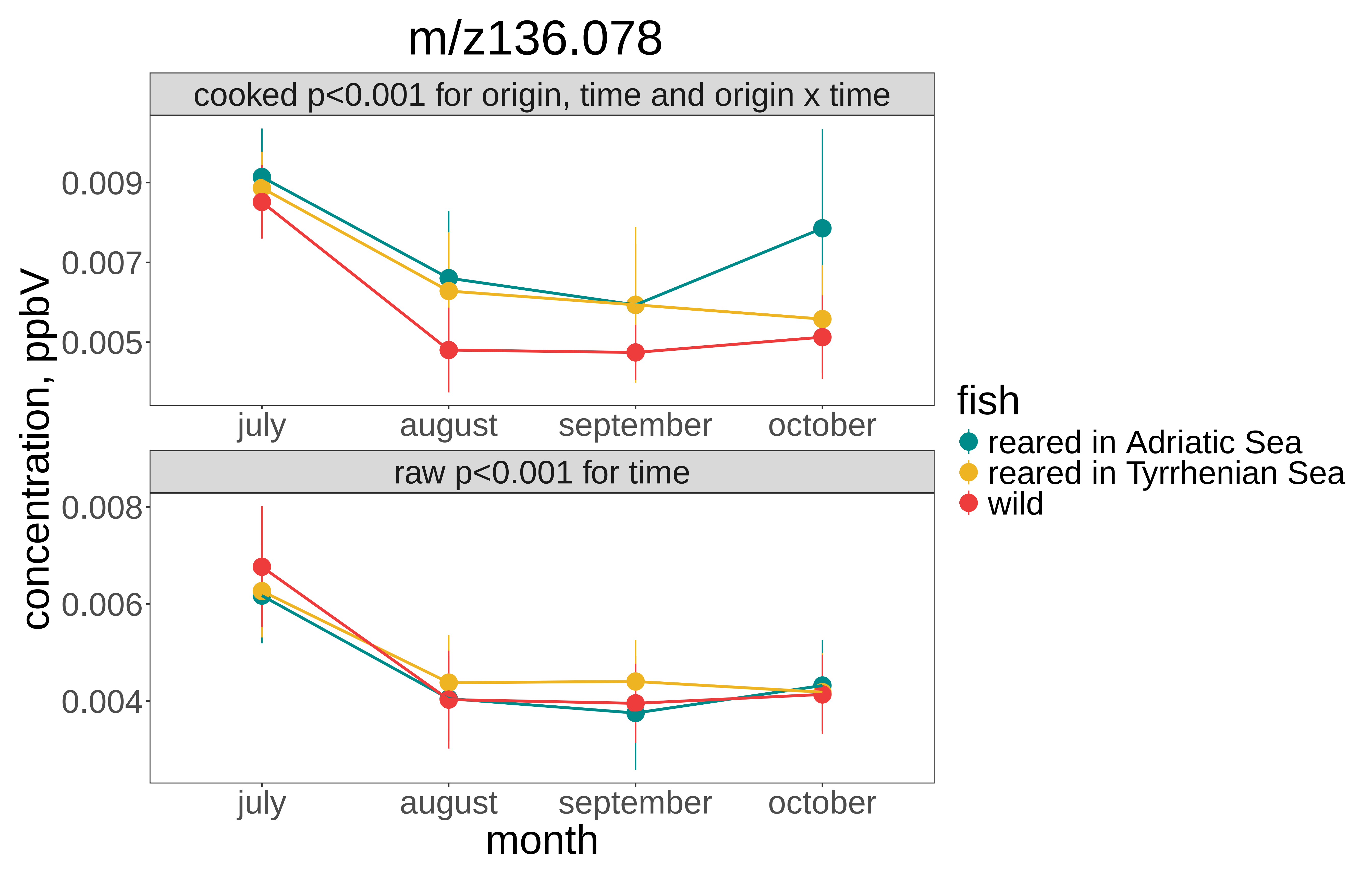
m/z136.078
cooked p<0.001 for origin, time and origin x time
raw p<0.001 for time
concentration, ppbV
0.009
0.007
0.005
0.008
0.006
0.004
july
august
september
october
month
fish
reared in Adriatic Sea
reared in Tyrrhenian Sea
wild

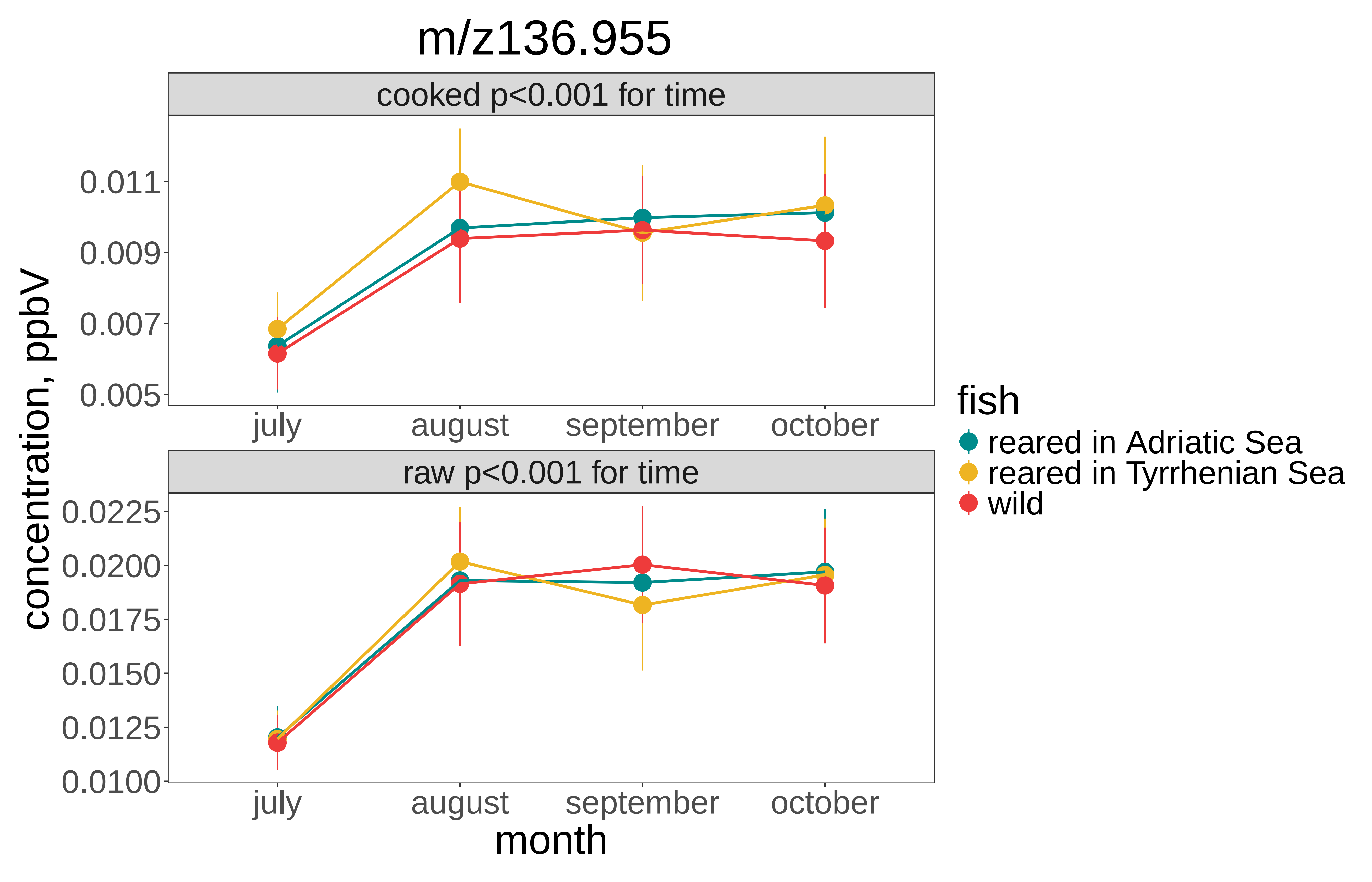

m/z136.955
cooked p<0.001 for time
0.011
0.009
0.007
0.005
july
august
september
october
raw p<0.001 for time
0.0225
0.0200
0.0175
0.0150
0.0125
0.0100
july
august
september
october
concentration, ppbV
month
fish
reared in Adriatic Sea
reared in Tyrrhenian Sea
wild

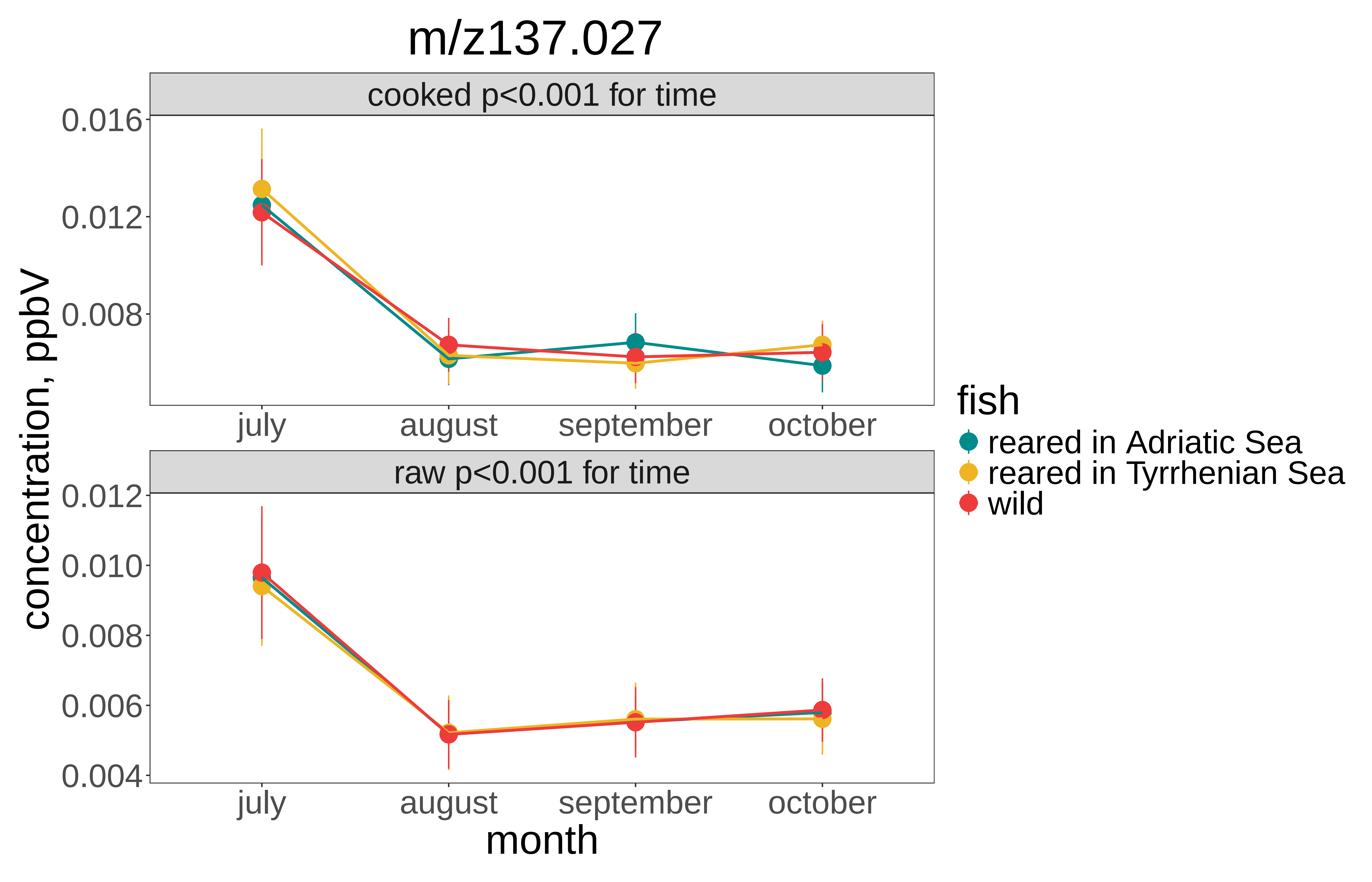
m/z137.027
cooked p<0.001 for time
raw p<0.001 for time
0.016
0.012
0.008
0.012
0.010
0.008
0.006
0.004
concentration, ppbV
july
august
september
october
month
fish
reared in Adriatic Sea
reared in Tyrrhenian Sea
wild

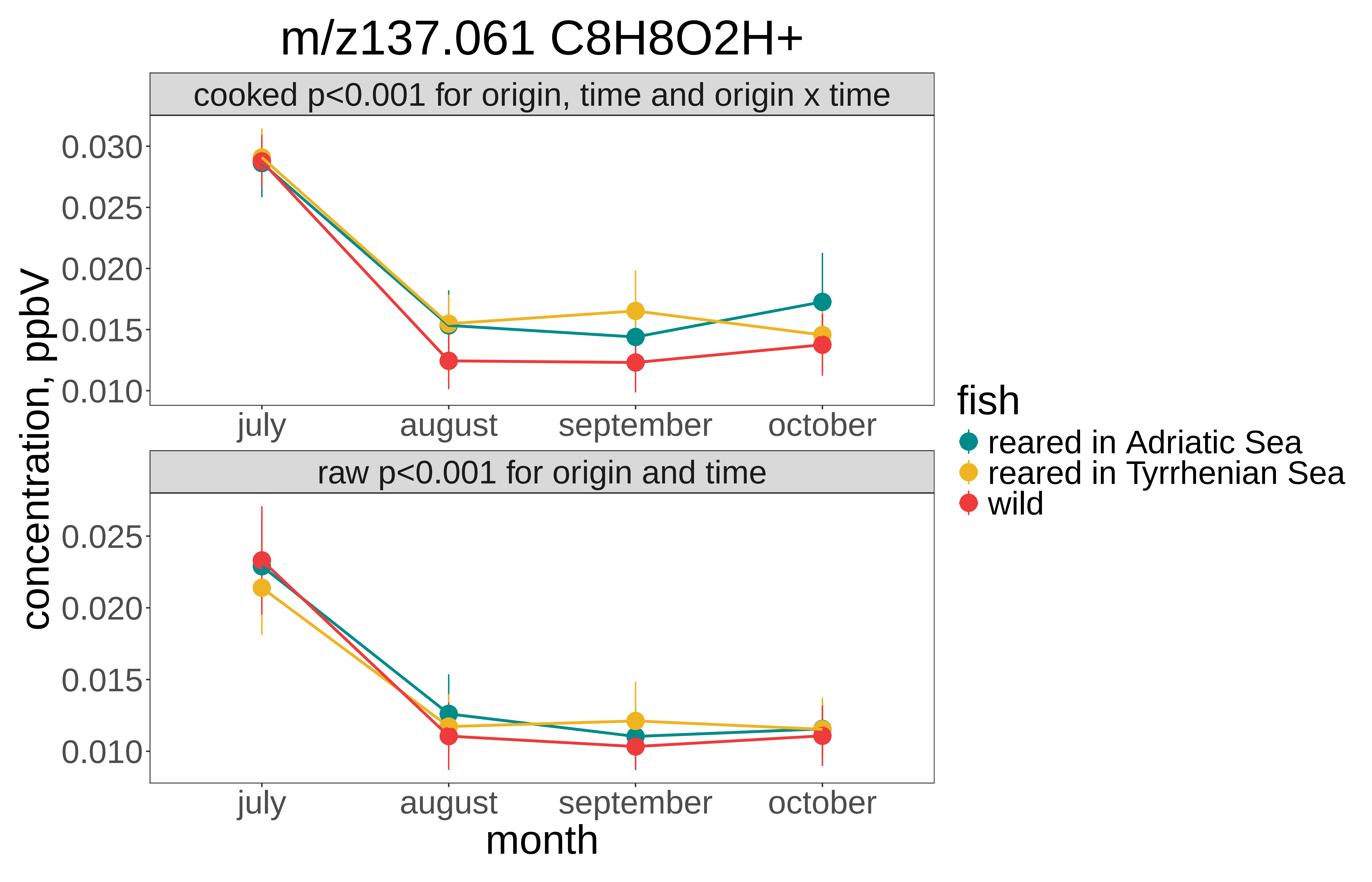
m/z137.061 C8H8O2H+
cooked p<0.001 for origin, time and origin x time
raw p<0.001 for origin and time
0.030
0.025
0.020
0.015
0.010
july
august
september
october
concentration, ppbV
month
fish
reared in Adriatic Sea
reared in Tyrrhenian Sea
wild

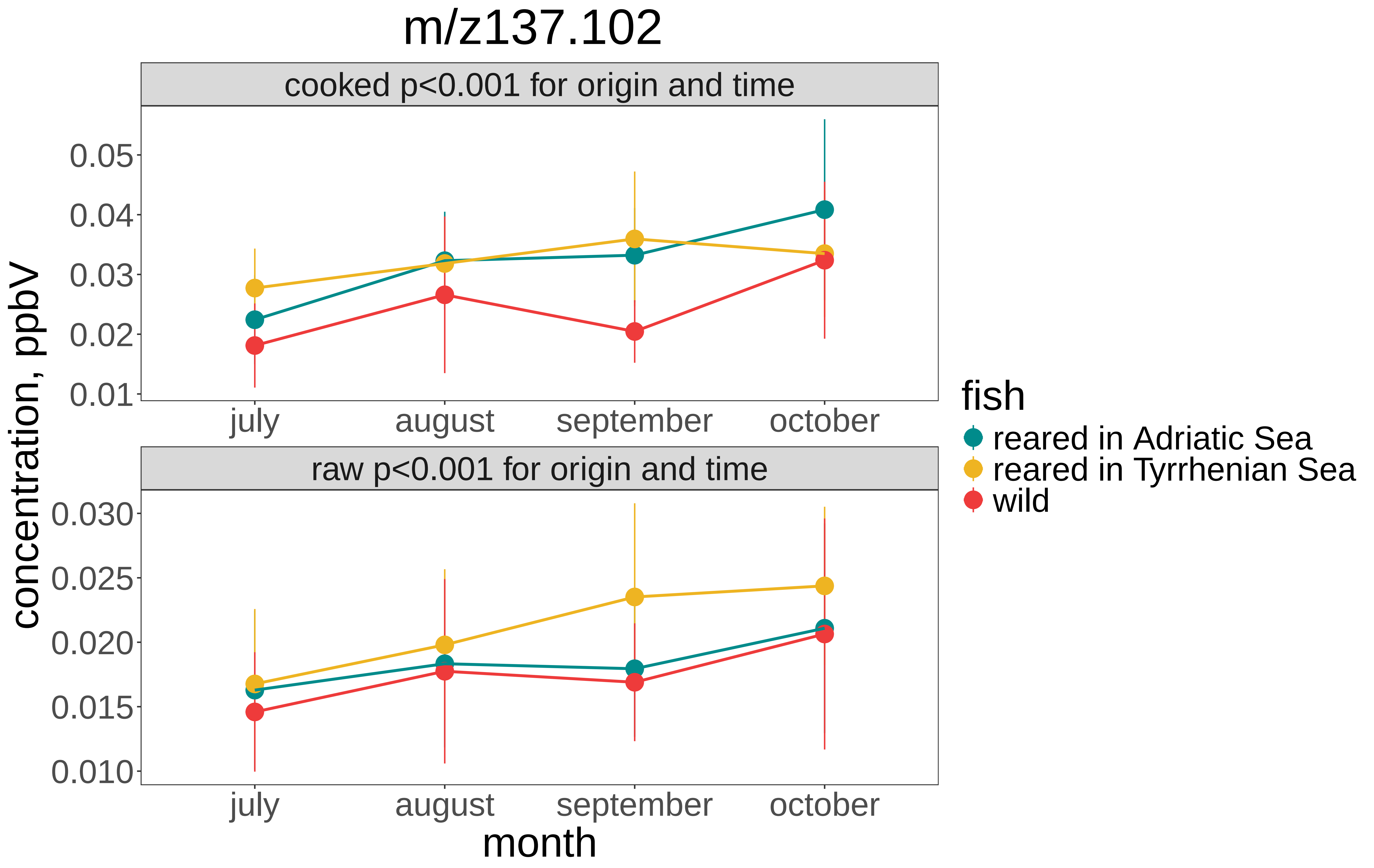
m/z137.102
cooked p<0.001 for origin and time
raw p<0.001 for origin and time
concentration, ppbV
0.05
0.04
0.03
0.02
0.01
0.030
0.025
0.020
0.015
0.010
july
august
september
october
month
fish
reared in Adriatic Sea
reared in Tyrrhenian Sea
wild

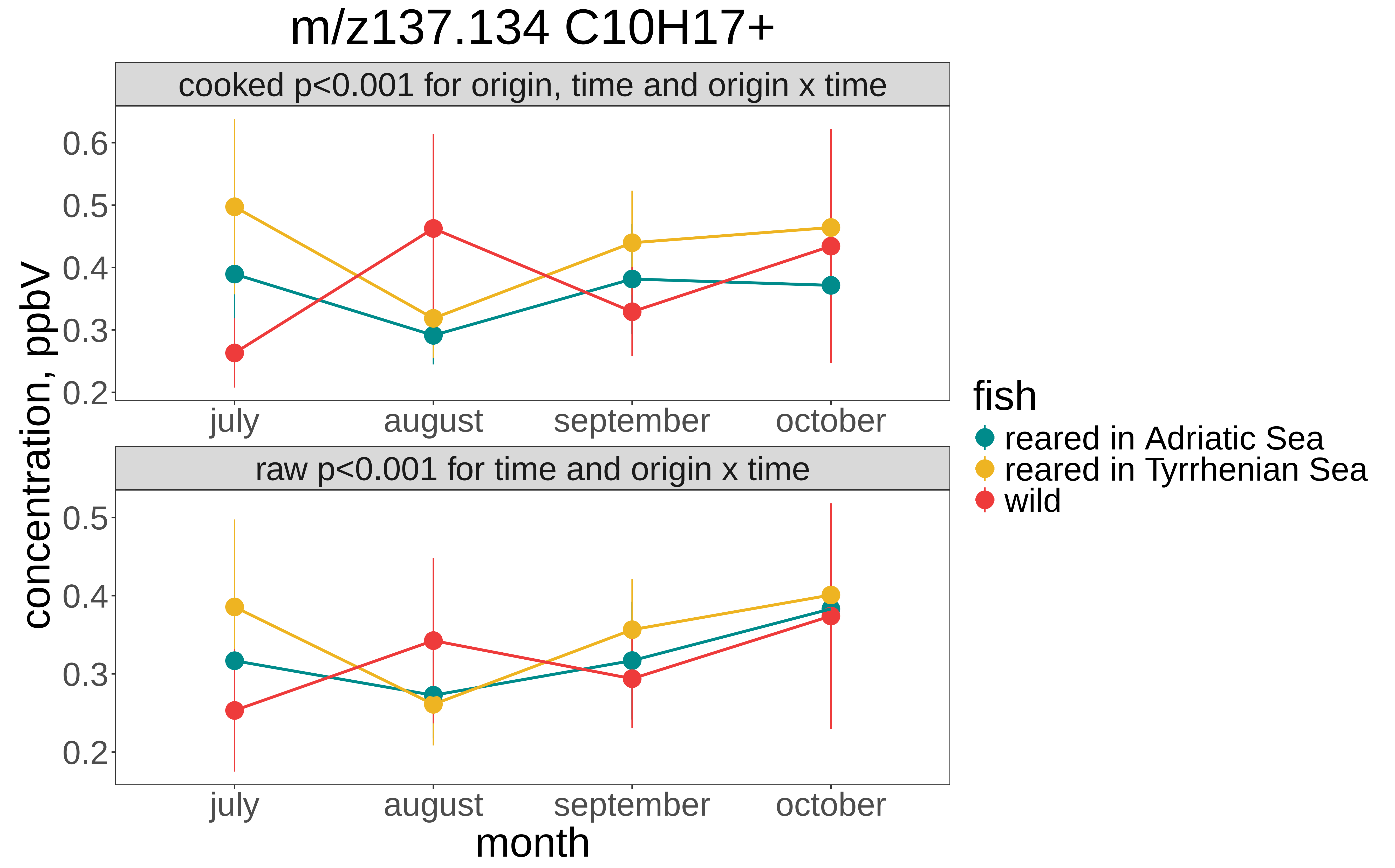
m/z137.134 C10H17+
cooked p<0.001 for origin, time and origin x time
raw p<0.001 for time and origin x time
concentration, ppbV
0.2
0.3
0.4
0.5
0.6
july
august
september
october
month
fish
reared in Adriatic Sea
reared in Tyrrhenian Sea
wild

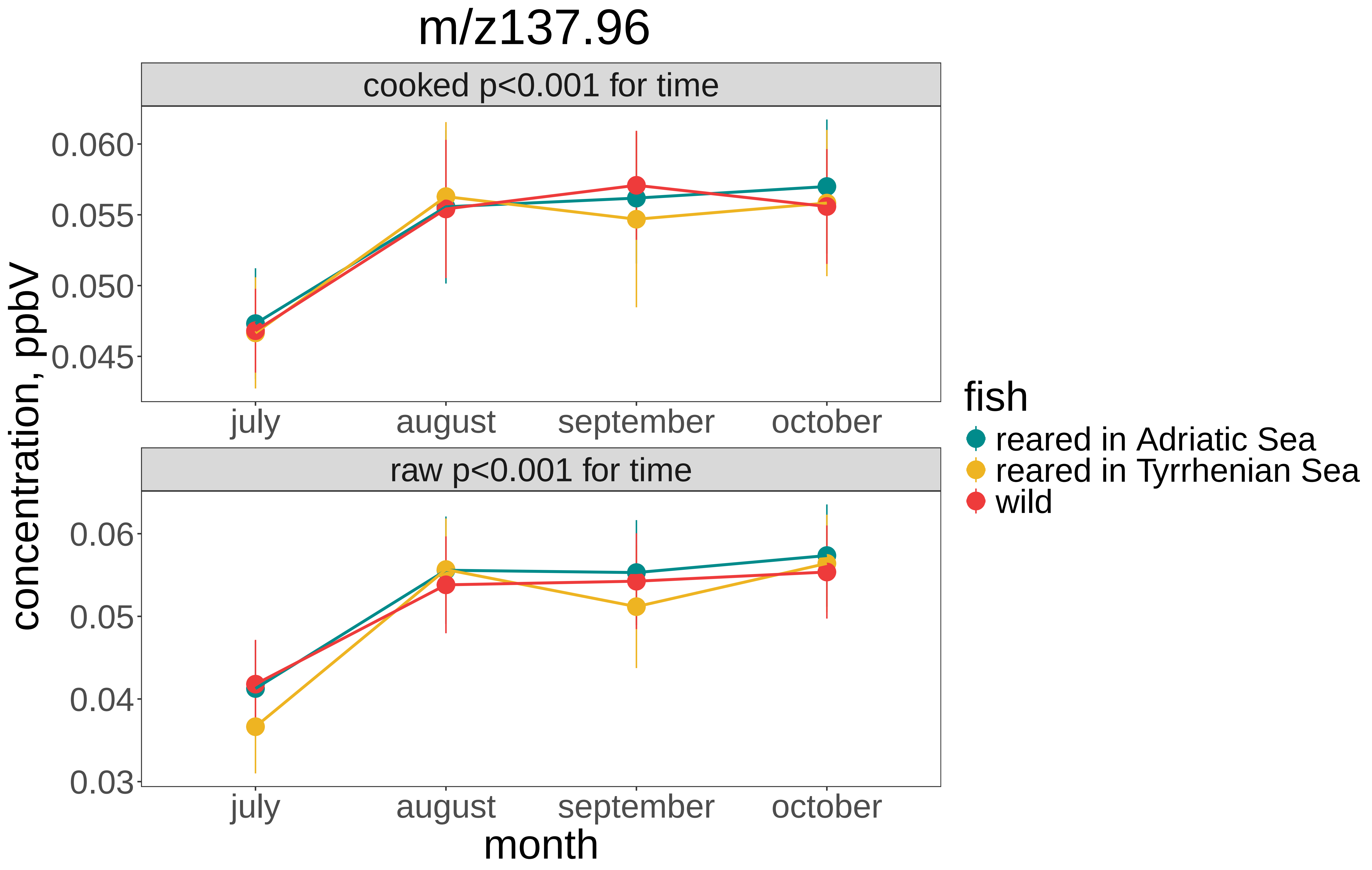

m/z137.96
cooked p<0.001 for time
raw p<0.001 for time
0.060
0.055
0.050
0.045
0.06
0.05
0.04
0.03
concentration, ppbV
july
august
september
october
month
fish
reared in Adriatic Sea
reared in Tyrrhenian Sea
wild

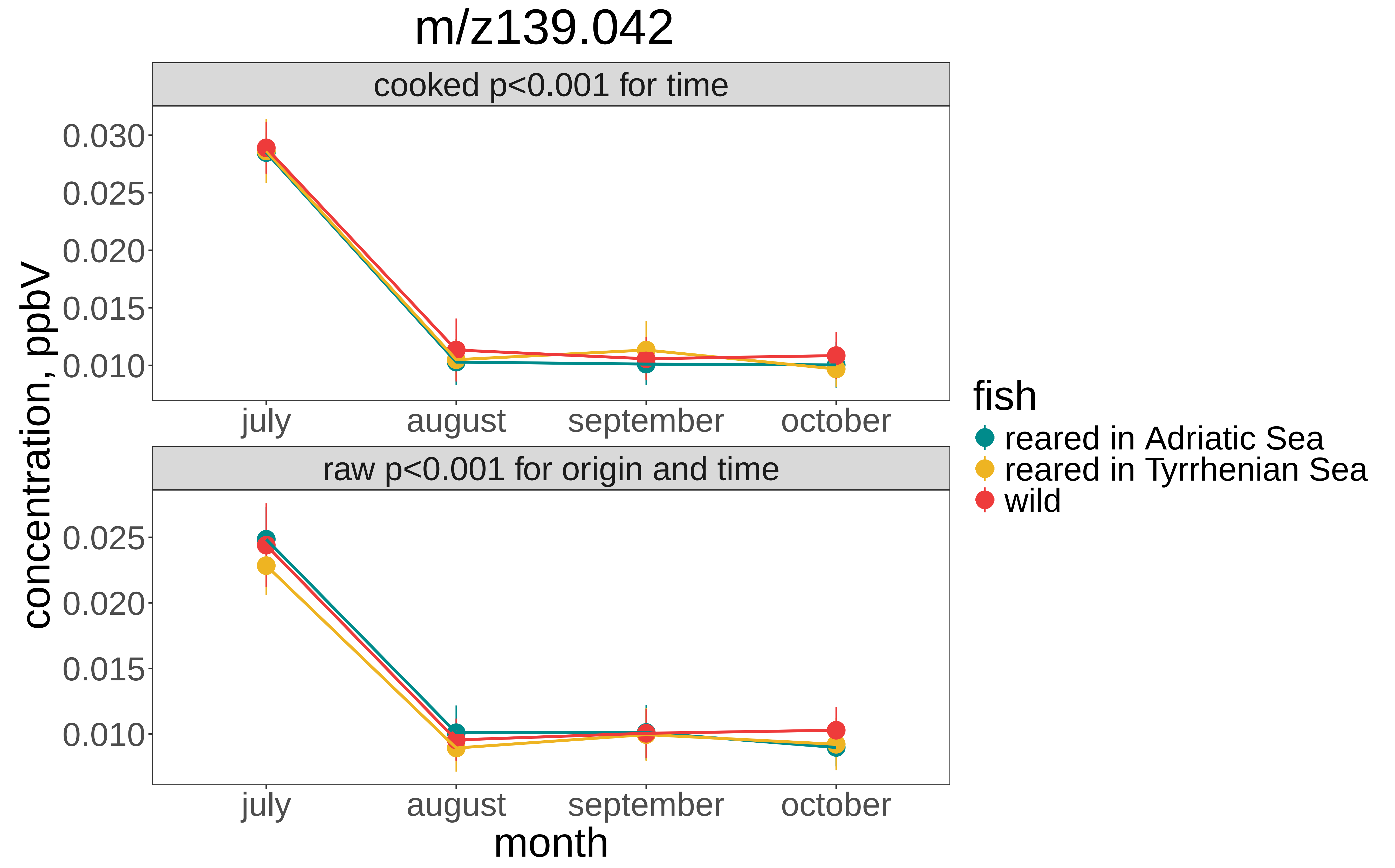
m/z139.042
cooked p<0.001 for time
raw p<0.001 for origin and time
concentration, ppbV
0.030
0.025
0.020
0.015
0.010
july
august
september
october
month
fish
reared in Adriatic Sea
reared in Tyrrhenian Sea
wild

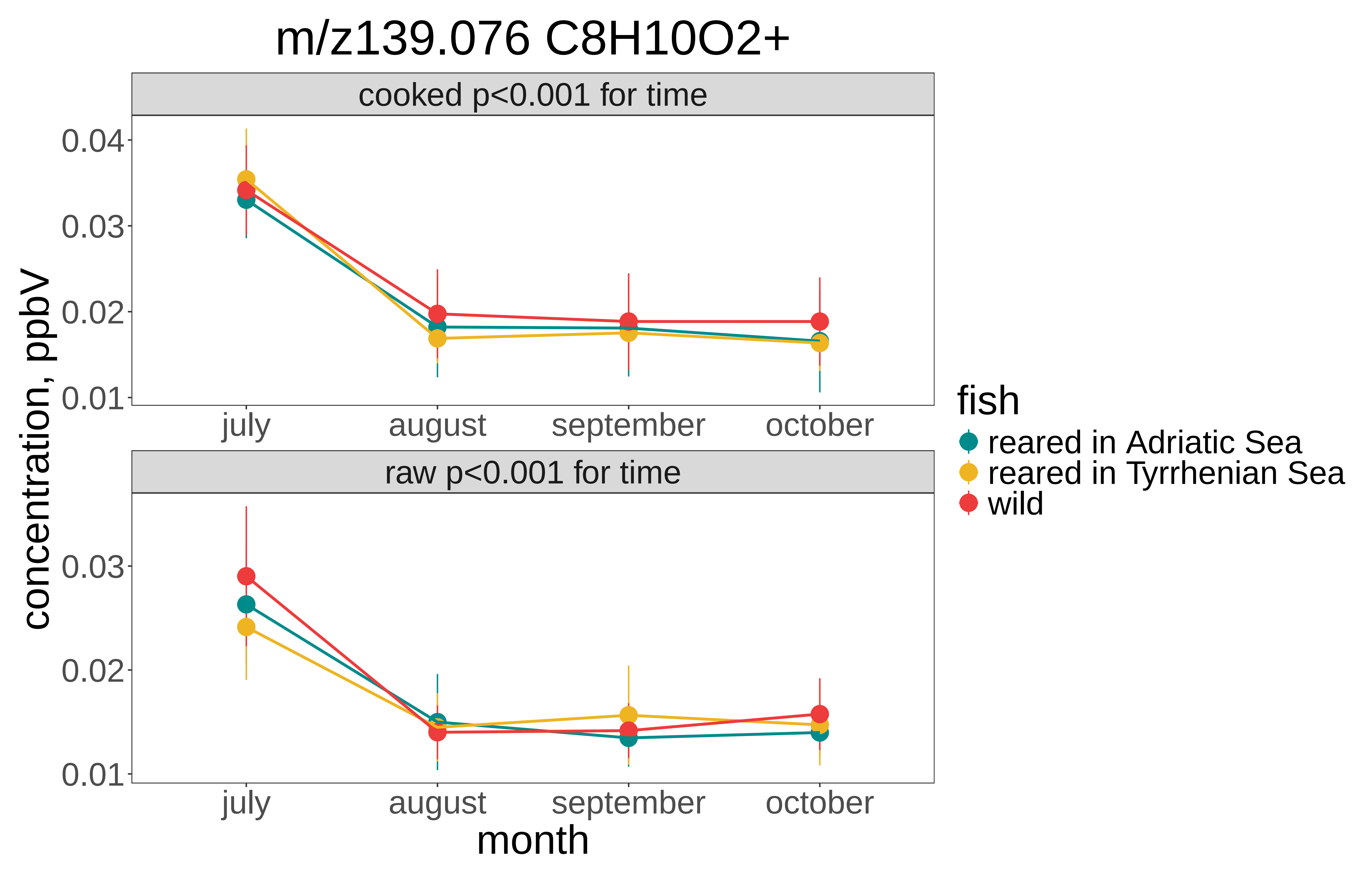
m/z139.076 C8H10O2+
cooked p<0.001 for time
raw p<0.001 for time
concentration, ppbV
0.04
0.03
0.02
0.01
july
august
september
october
month
fish
reared in Adriatic Sea
reared in Tyrrhenian Sea
wild

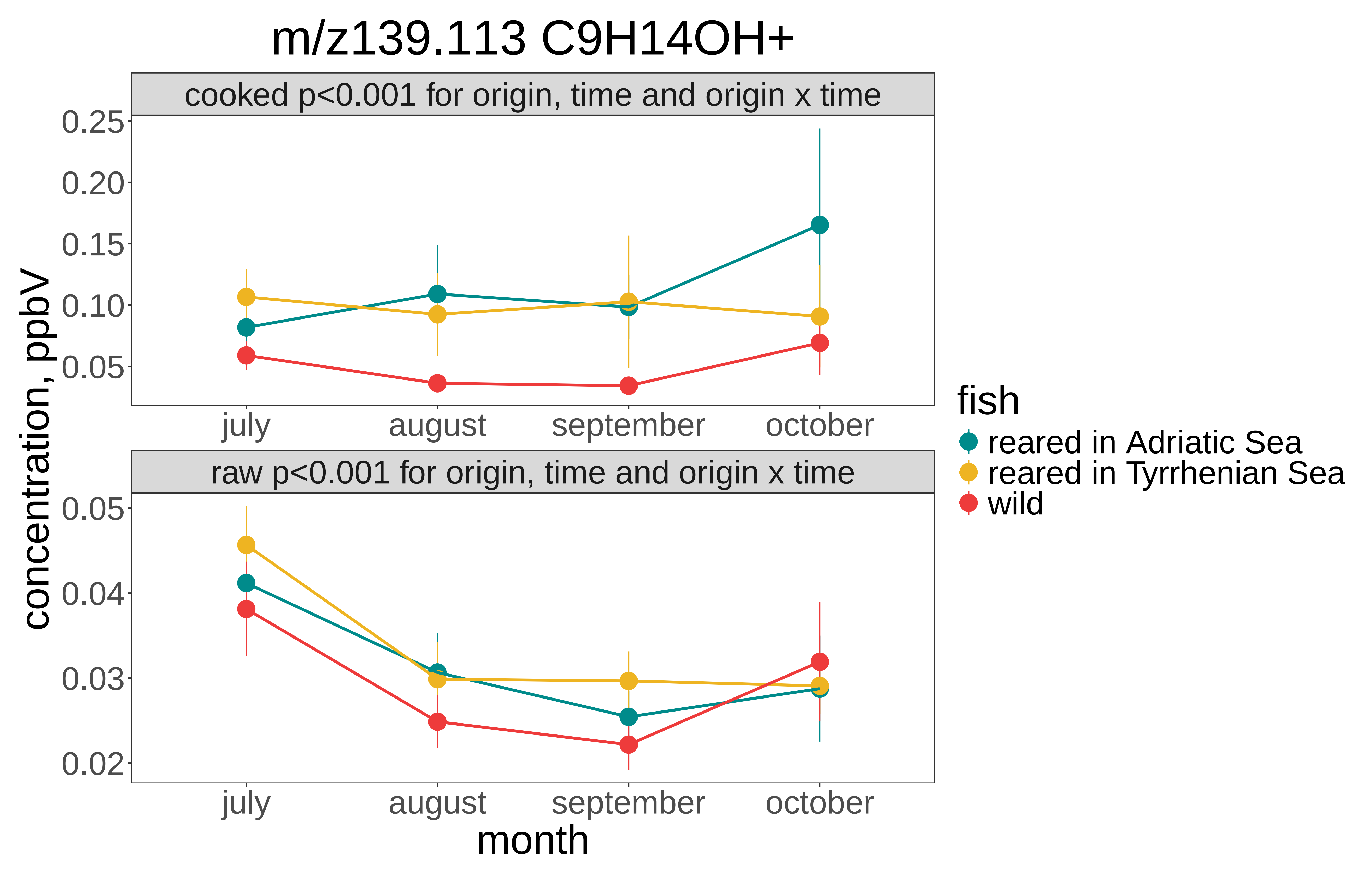

m/z139.113 C9H14OH+
cooked p<0.001 for origin, time and origin x time
raw p<0.001 for origin, time and origin x time
concentration, ppbV
0.25
0.20
0.15
0.10
0.05
0.05
0.04
0.03
0.02
july
august
september
october
month
fish
reared in Adriatic Sea
reared in Tyrrhenian Sea
wild

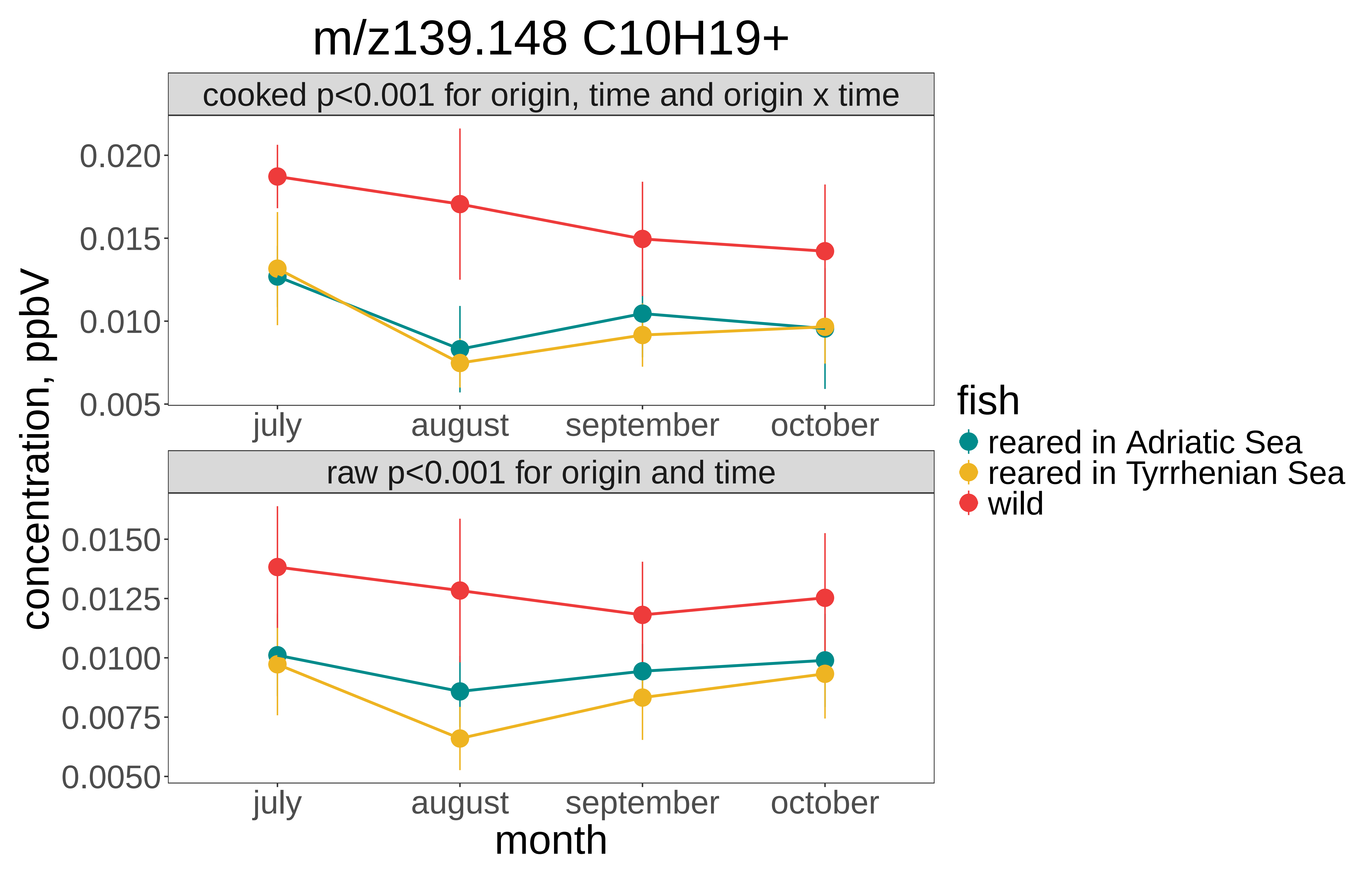

m/z139.148 C10H19+
cooked p<0.001 for origin, time and origin x time
raw p<0.001 for origin and time
concentration, ppbV
0.020
0.015
0.010
0.005
0.0150
0.0125
0.0100
0.0075
0.0050
july
august
september
october
month
fish
reared in Adriatic Sea
reared in Tyrrhenian Sea
wild

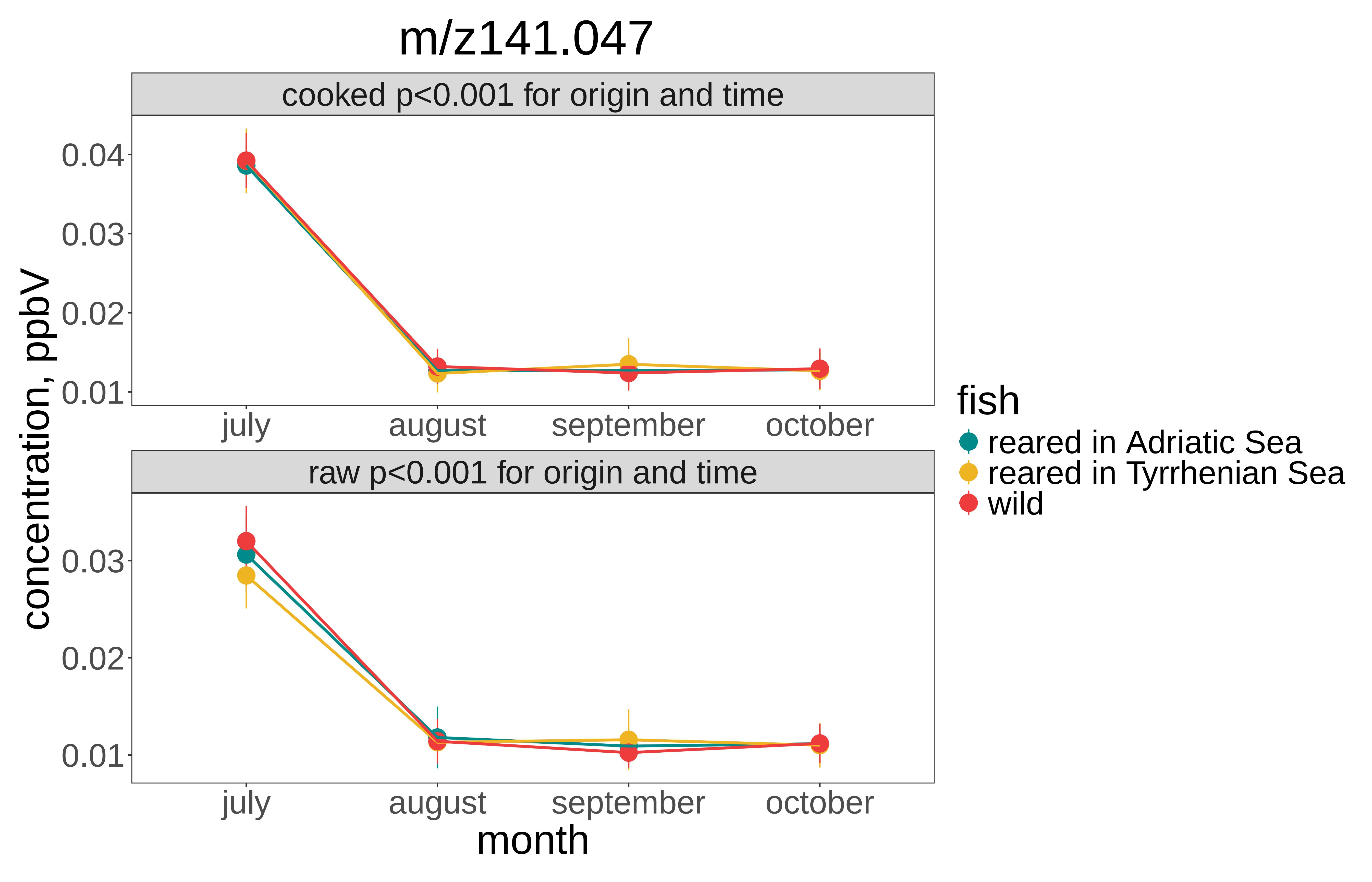

m/z141.047
cooked p<0.001 for origin and time
raw p<0.001 for origin and time
concentration, ppbV
0.04
0.03
0.02
0.01
july
august
september
october
month
fish
reared in Adriatic Sea
reared in Tyrrhenian Sea
wild

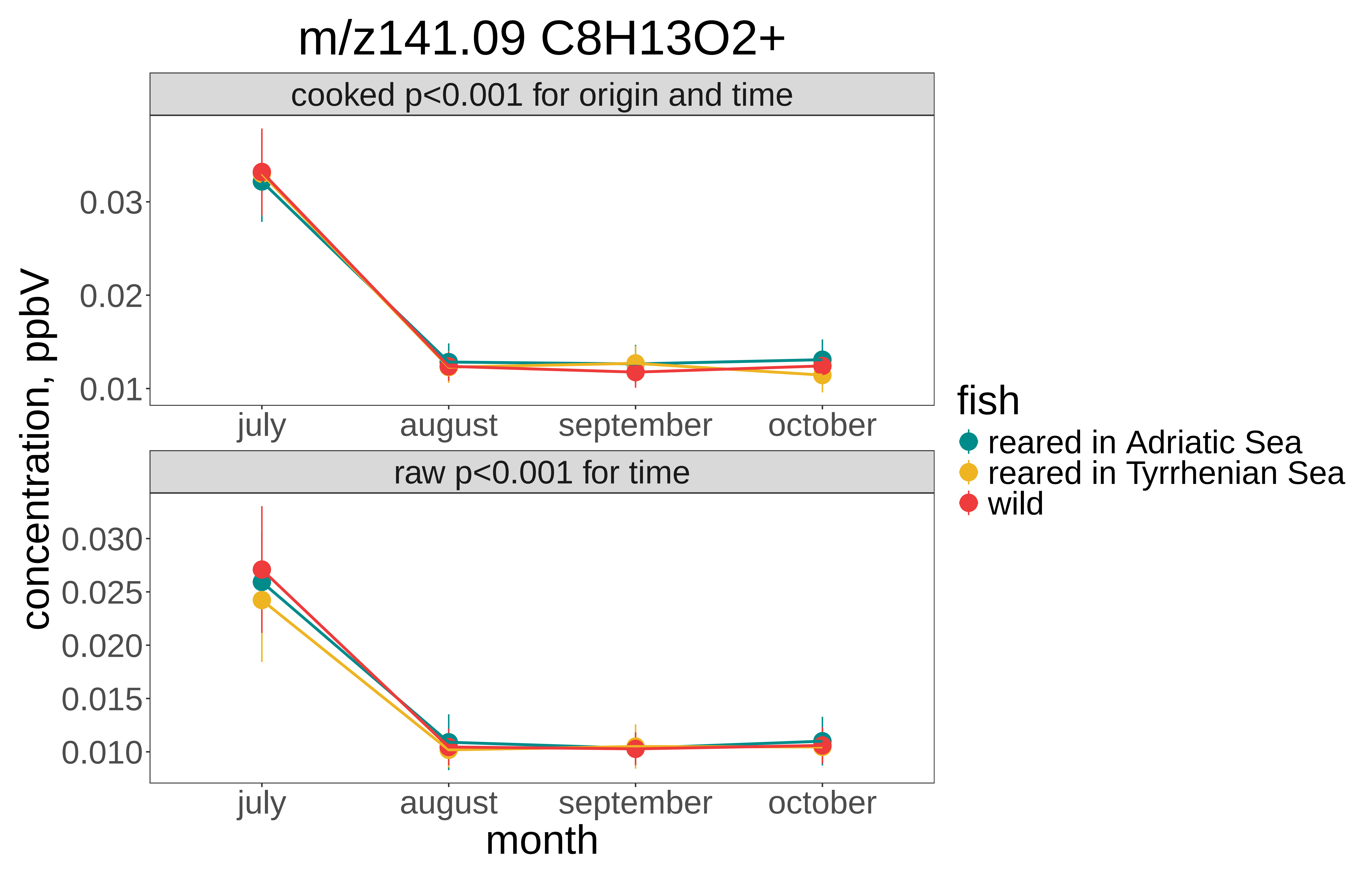
m/z141.09 C8H13O2+
cooked p<0.001 for origin and time
raw p<0.001 for time
concentration, ppbV
0.03
0.02
0.01
0.030
0.025
0.020
0.015
0.010
july
august
september
october
month
fish
reared in Adriatic Sea
reared in Tyrrhenian Sea
wild

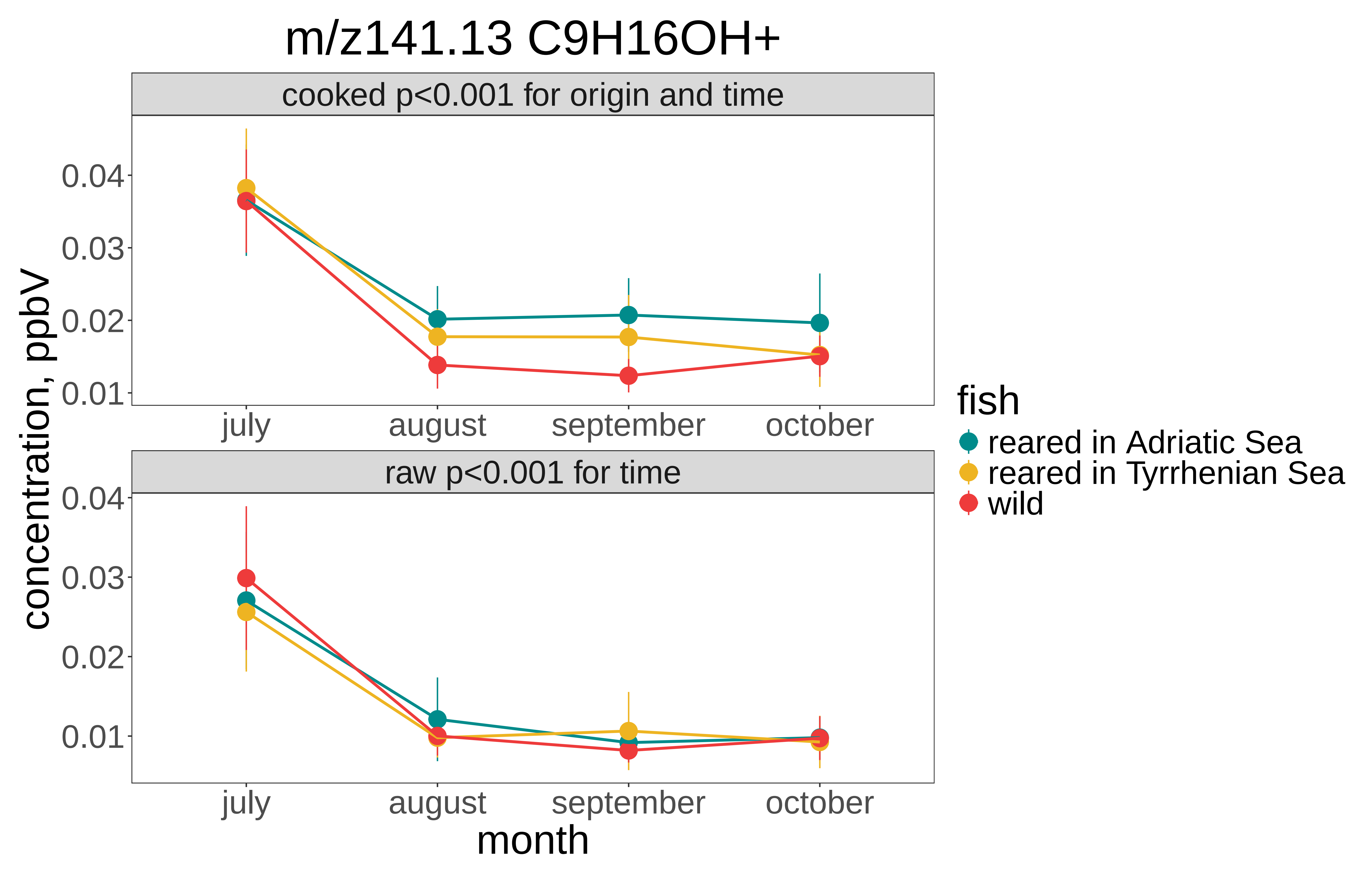

m/z141.13 C9H16OH+
cooked p<0.001 for origin and time
raw p<0.001 for time
concentration, ppbV
0.04
0.03
0.02
0.01
july
august
september
october
month
fish
reared in Adriatic Sea
reared in Tyrrhenian Sea
wild

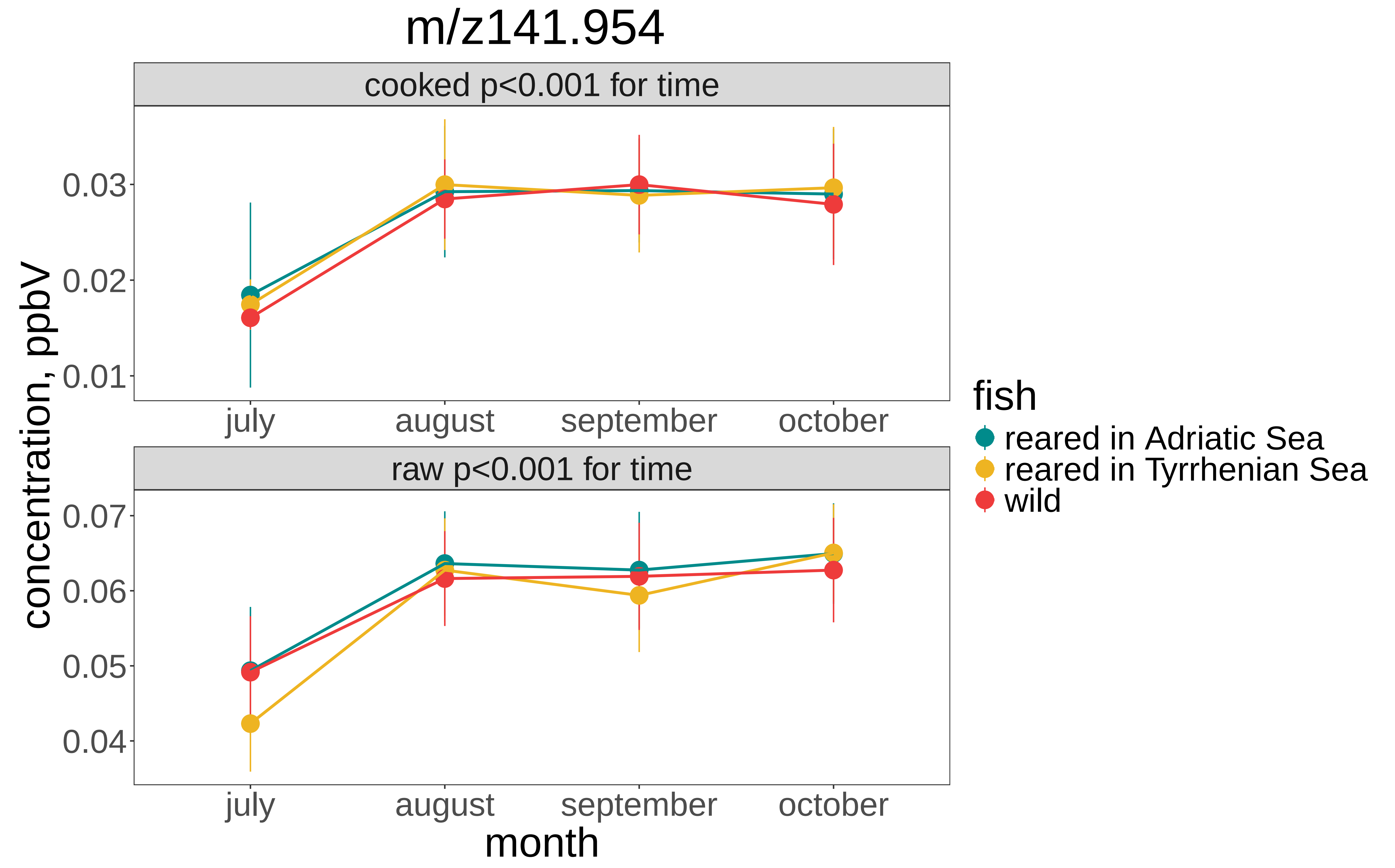

m/z141.954
cooked p<0.001 for time
raw p<0.001 for time
concentration, ppbV
0.03
0.02
0.01
0.07
0.06
0.05
0.04
july
august
september
october
month
fish
reared in Adriatic Sea
reared in Tyrrhenian Sea
wild

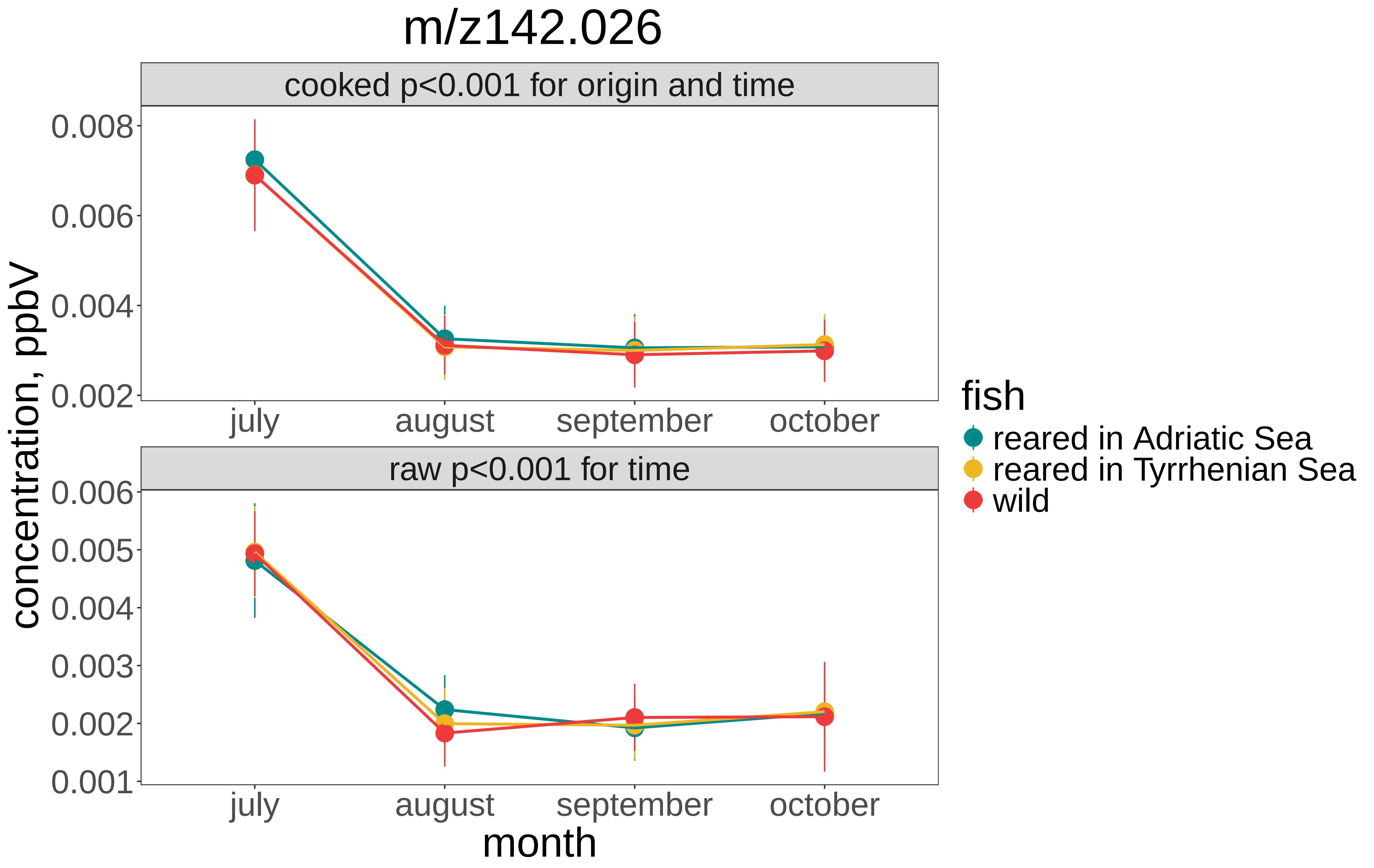
m/z142.026
cooked p<0.001 for origin and time
raw p<0.001 for time
0.008
0.006
0.004
0.002
0.006
0.005
0.004
0.003
0.002
0.001
concentration, ppbV
july
august
september
october
month
fish
reared in Adriatic Sea
reared in Tyrrhenian Sea
wild

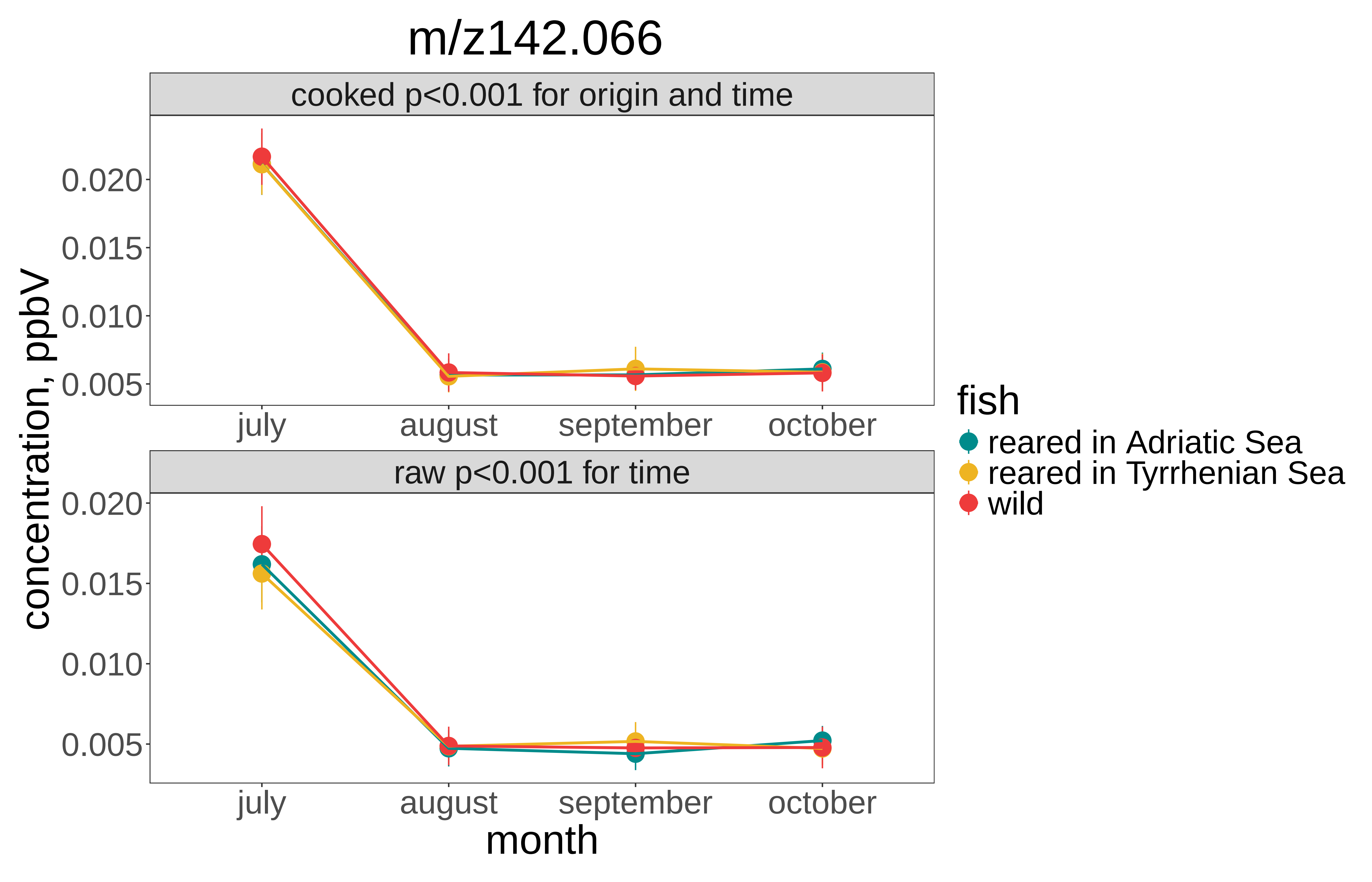

m/z142.066
cooked p<0.001 for origin and time
raw p<0.001 for time
concentration, ppbV
0.020
0.015
0.010
0.005
july
august
september
october
month
fish
reared in Adriatic Sea
reared in Tyrrhenian Sea
wild

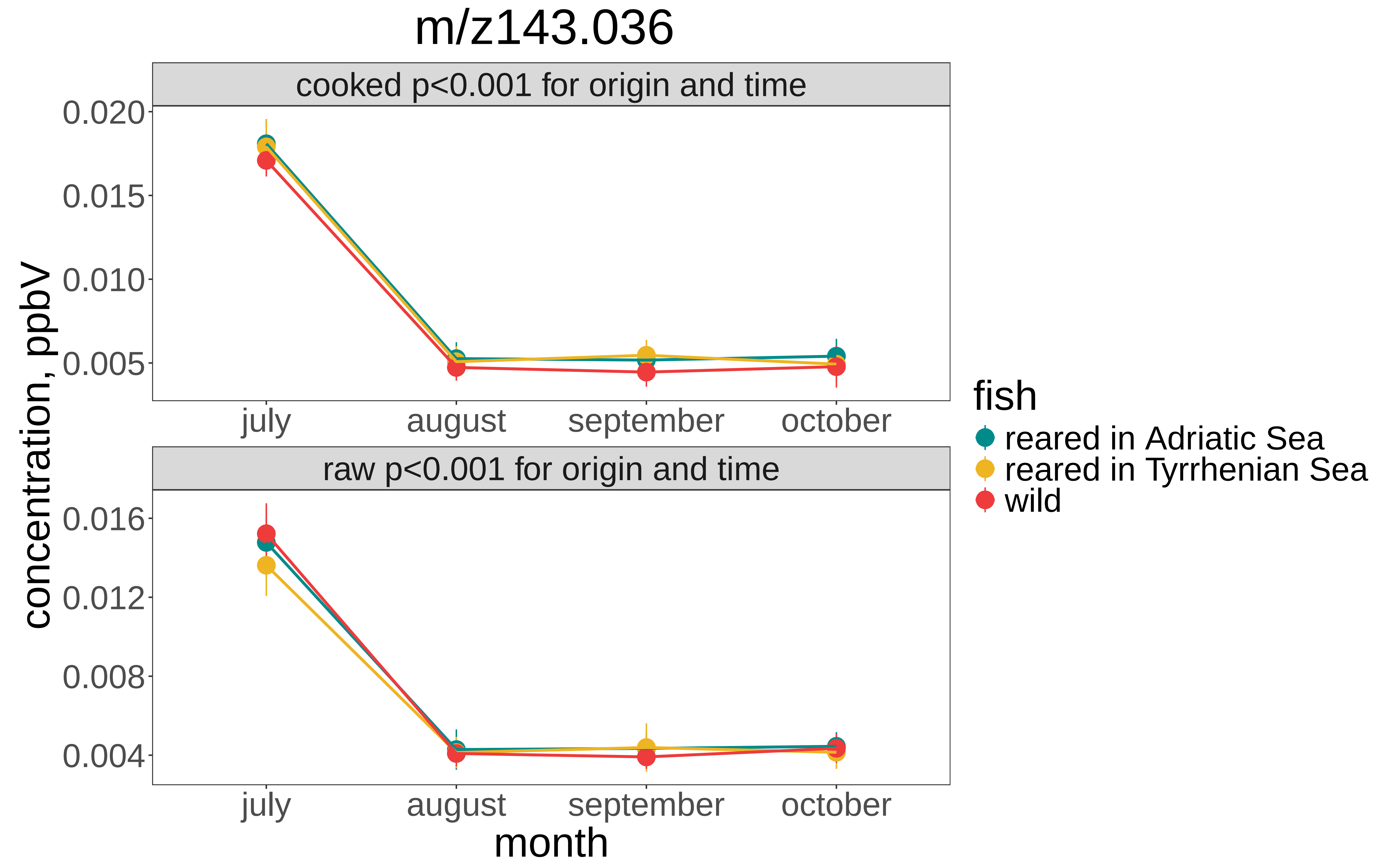

m/z143.036
cooked p<0.001 for origin and time
0.020
0.015
0.010
0.005
july
august
september
october
raw p<0.001 for origin and time
0.016
0.012
0.008
0.004
july
august
september
october
concentration, ppbV
month
fish
reared in Adriatic Sea
reared in Tyrrhenian Sea
wild

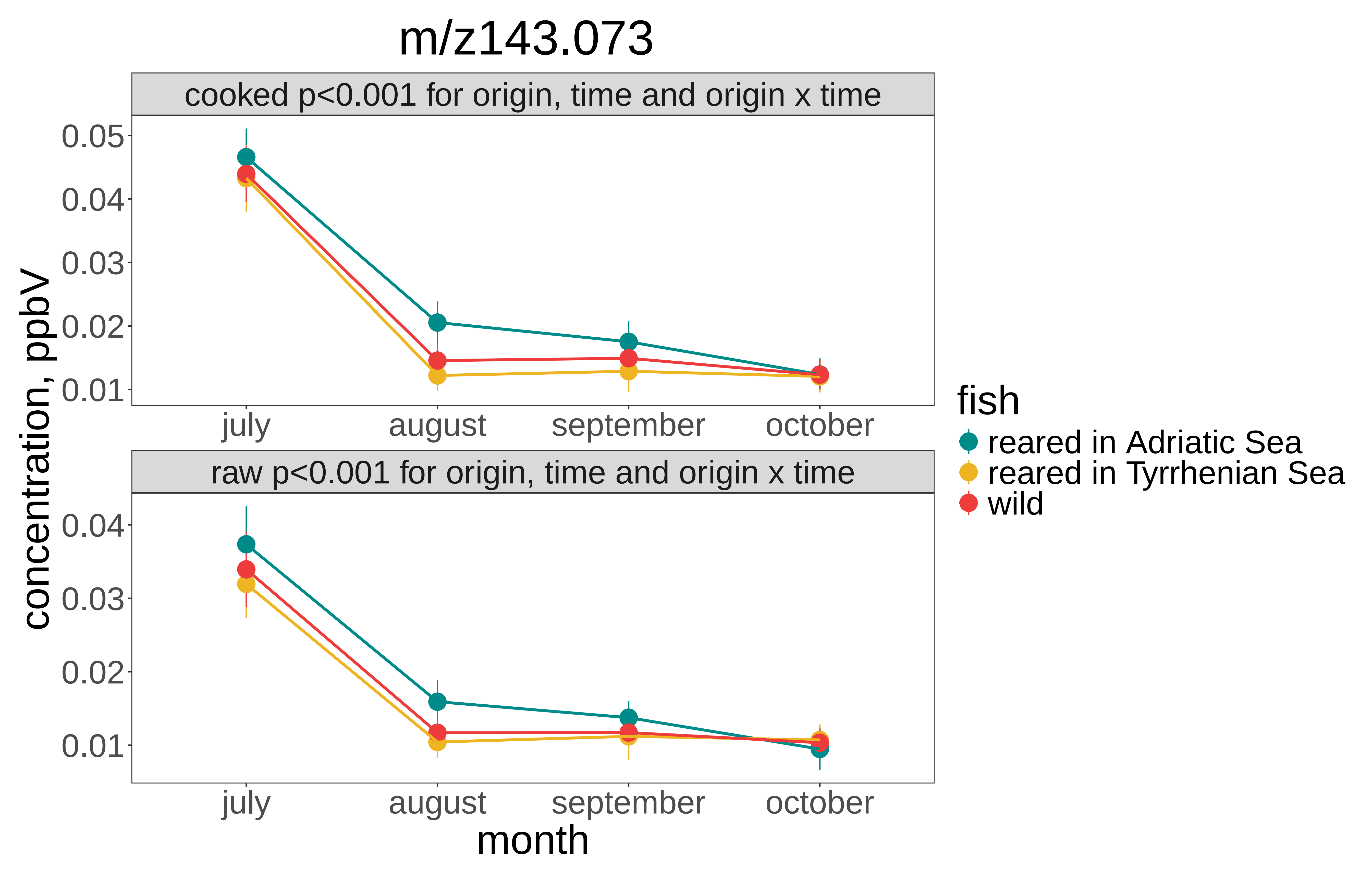
m/z143.073
cooked p<0.001 for origin, time and origin x time
raw p<0.001 for origin, time and origin x time
0.05
0.04
0.03
0.02
0.01
concentration, ppbV
july
august
september
october
month
fish
reared in Adriatic Sea
reared in Tyrrhenian Sea
wild

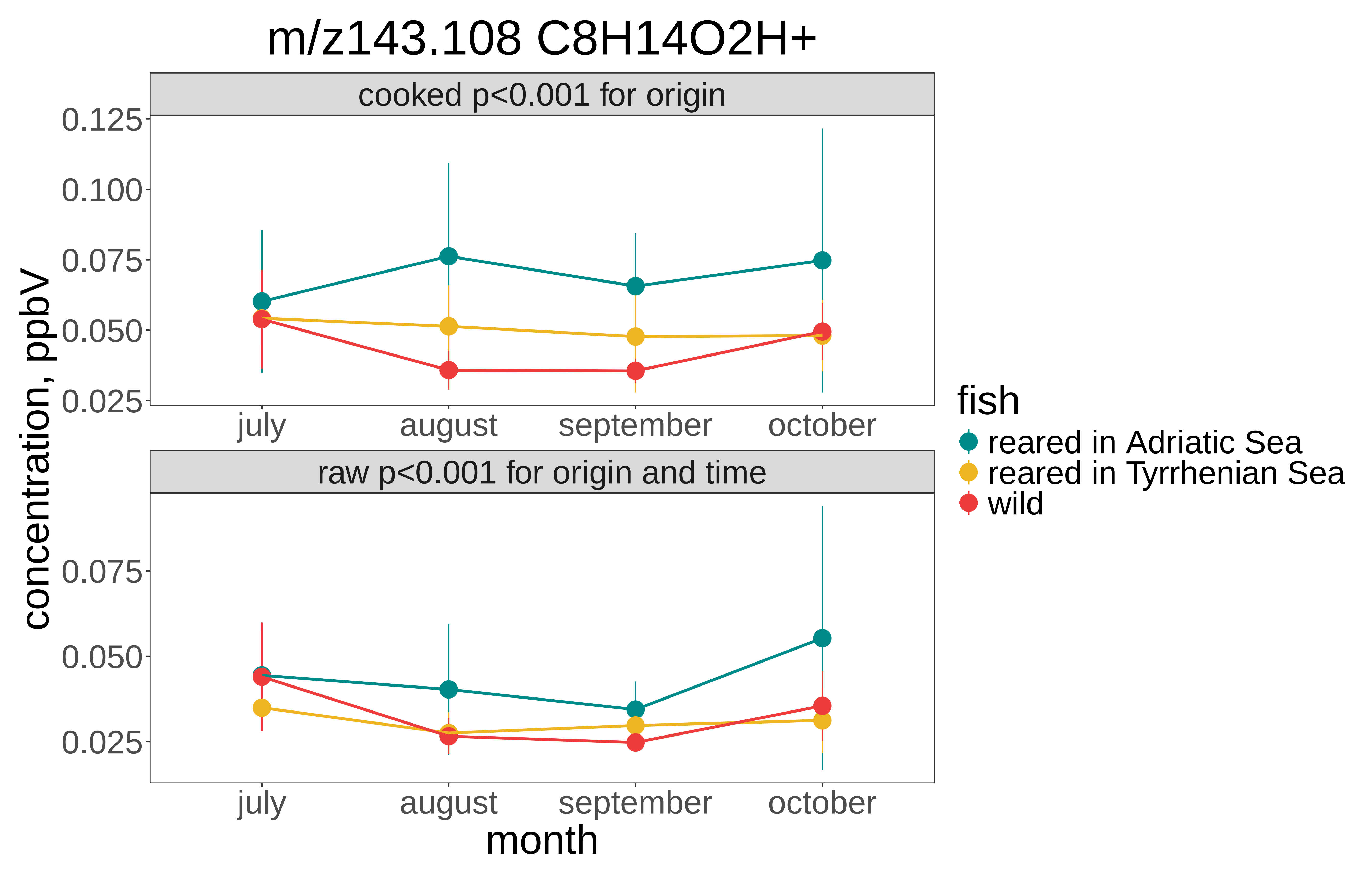
m/z143.108 C8H14O2H+
cooked p<0.001 for origin
raw p<0.001 for origin and time
concentration, ppbV
0.125
0.100
0.075
0.050
0.025
july
august
september
october
month
fish
reared in Adriatic Sea
reared in Tyrrhenian Sea
wild

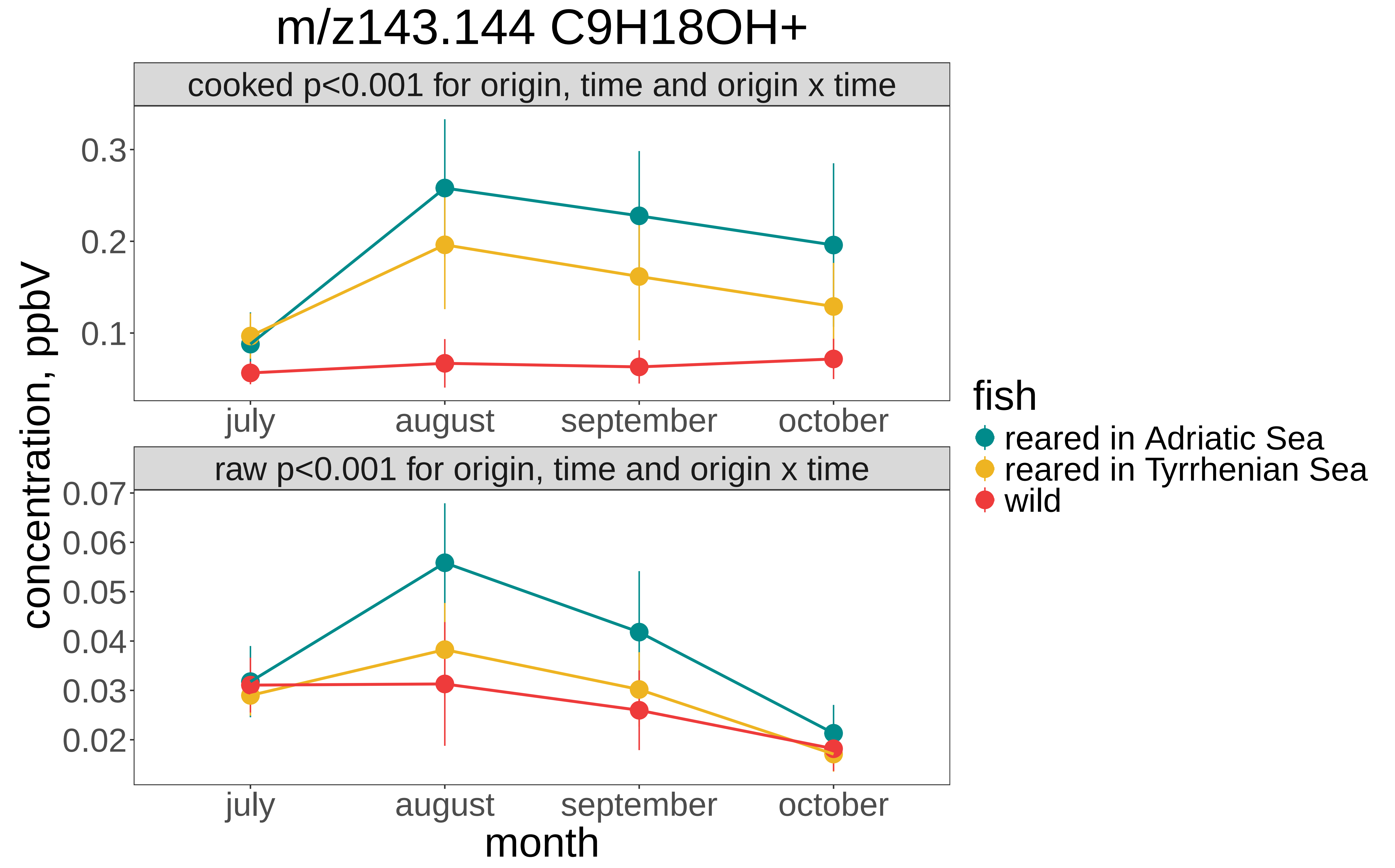
m/z143.144 C9H18OH+
cooked p<0.001 for origin, time and origin x time
raw p<0.001 for origin, time and origin x time
concentration, ppbV
0.3
0.2
0.1
0.07
0.06
0.05
0.04
0.03
0.02
july
august
september
october
month
fish
reared in Adriatic Sea
reared in Tyrrhenian Sea
wild

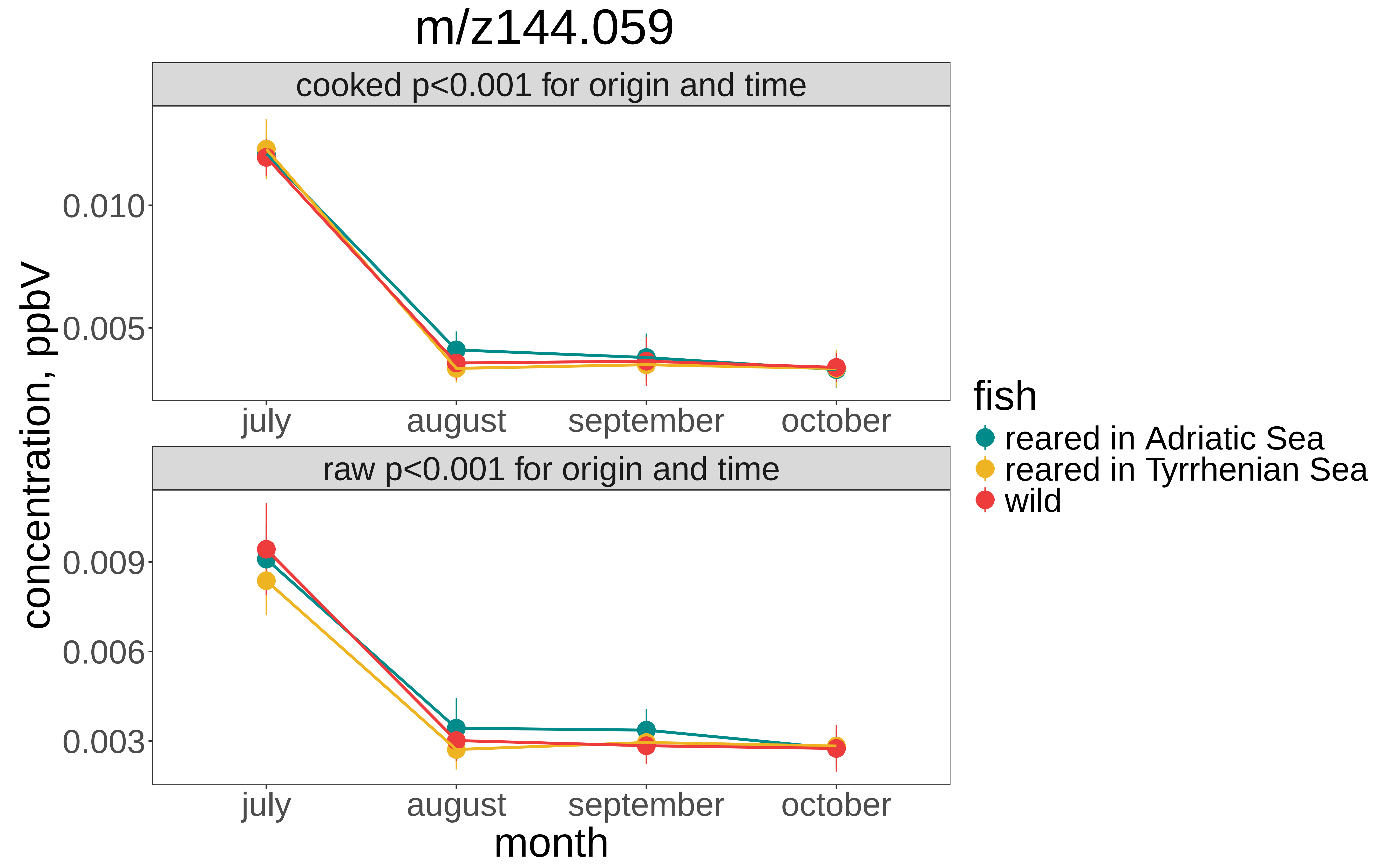

m/z144.059
cooked p<0.001 for origin and time
raw p<0.001 for origin and time
concentration, ppbV
0.010
0.005
0.009
0.006
0.003
july
august
september
october
month
fish
reared in Adriatic Sea
reared in Tyrrhenian Sea
wild

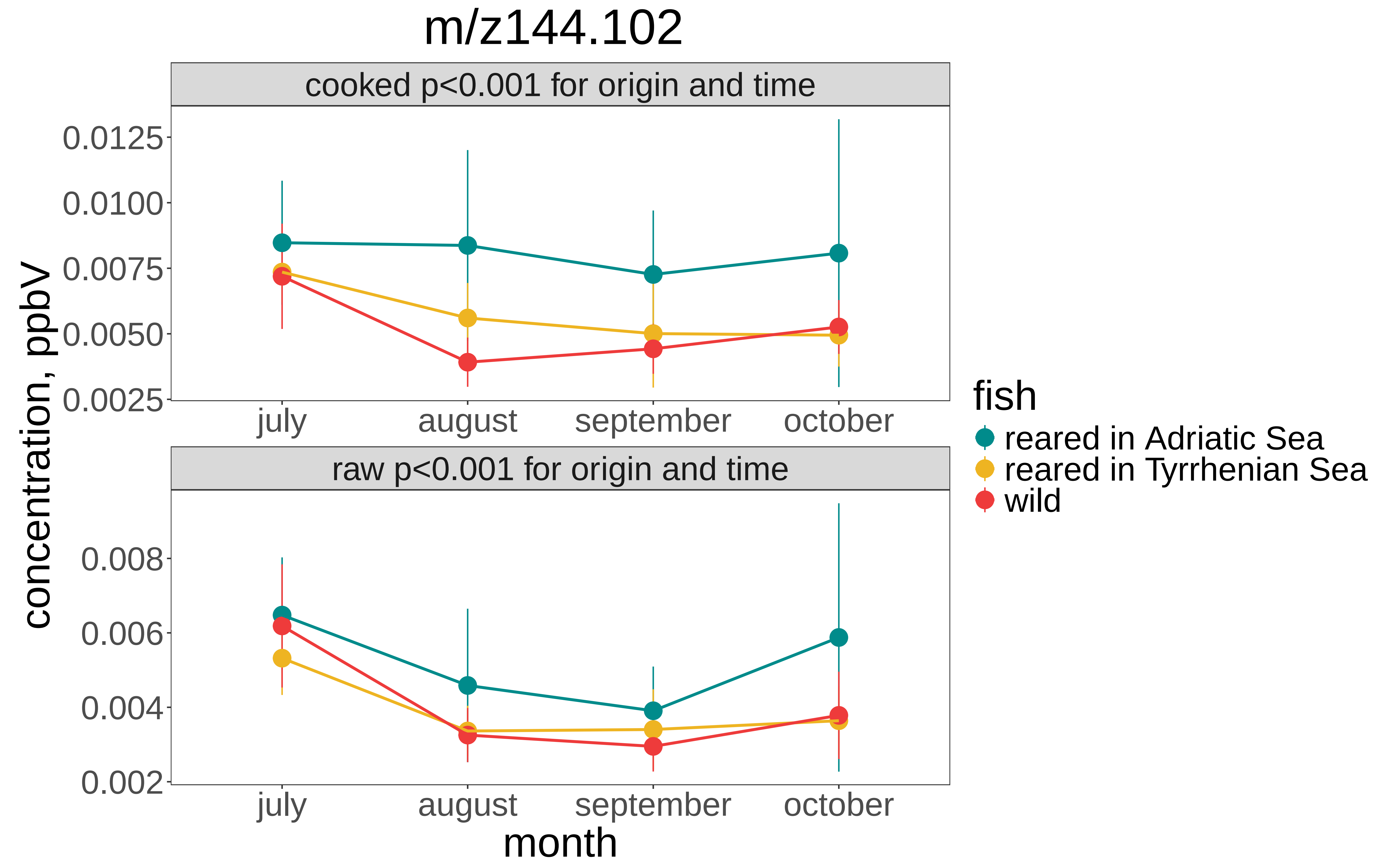
m/z144.102
cooked p<0.001 for origin and time
raw p<0.001 for origin and time
0.0125
0.0100
0.0075
0.0050
0.0025
0.008
0.006
0.004
0.002
july
august
september
october
concentration, ppbV
month
fish
reared in Adriatic Sea
reared in Tyrrhenian Sea
wild

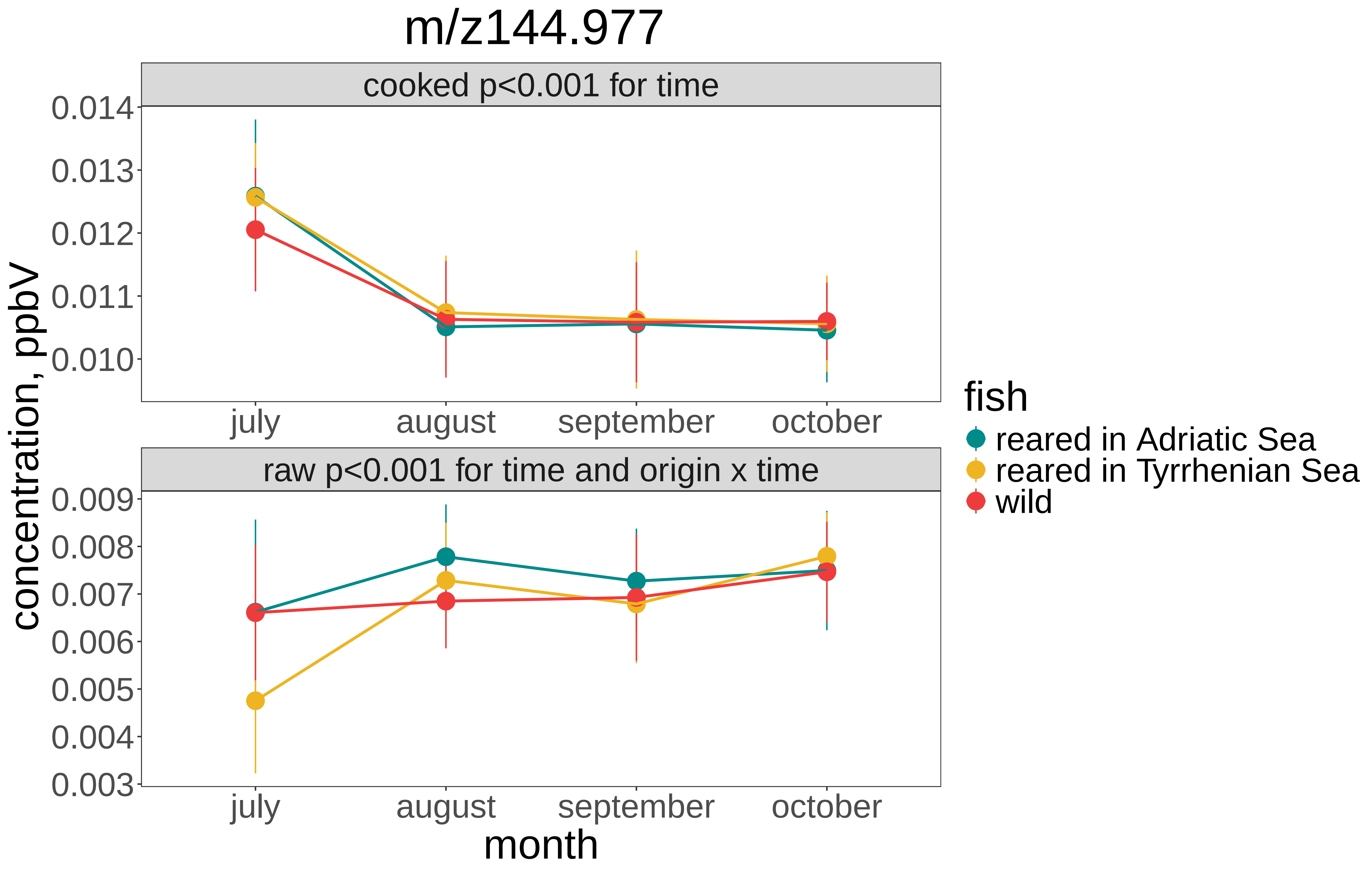
m/z144.977
cooked p<0.001 for time
raw p<0.001 for time and origin x time
0.014
0.013
0.012
0.011
0.010
0.009
0.008
0.007
0.006
0.005
0.004
0.003
concentration, ppbV
july
august
september
october
month
fish
reared in Adriatic Sea
reared in Tyrrhenian Sea
wild

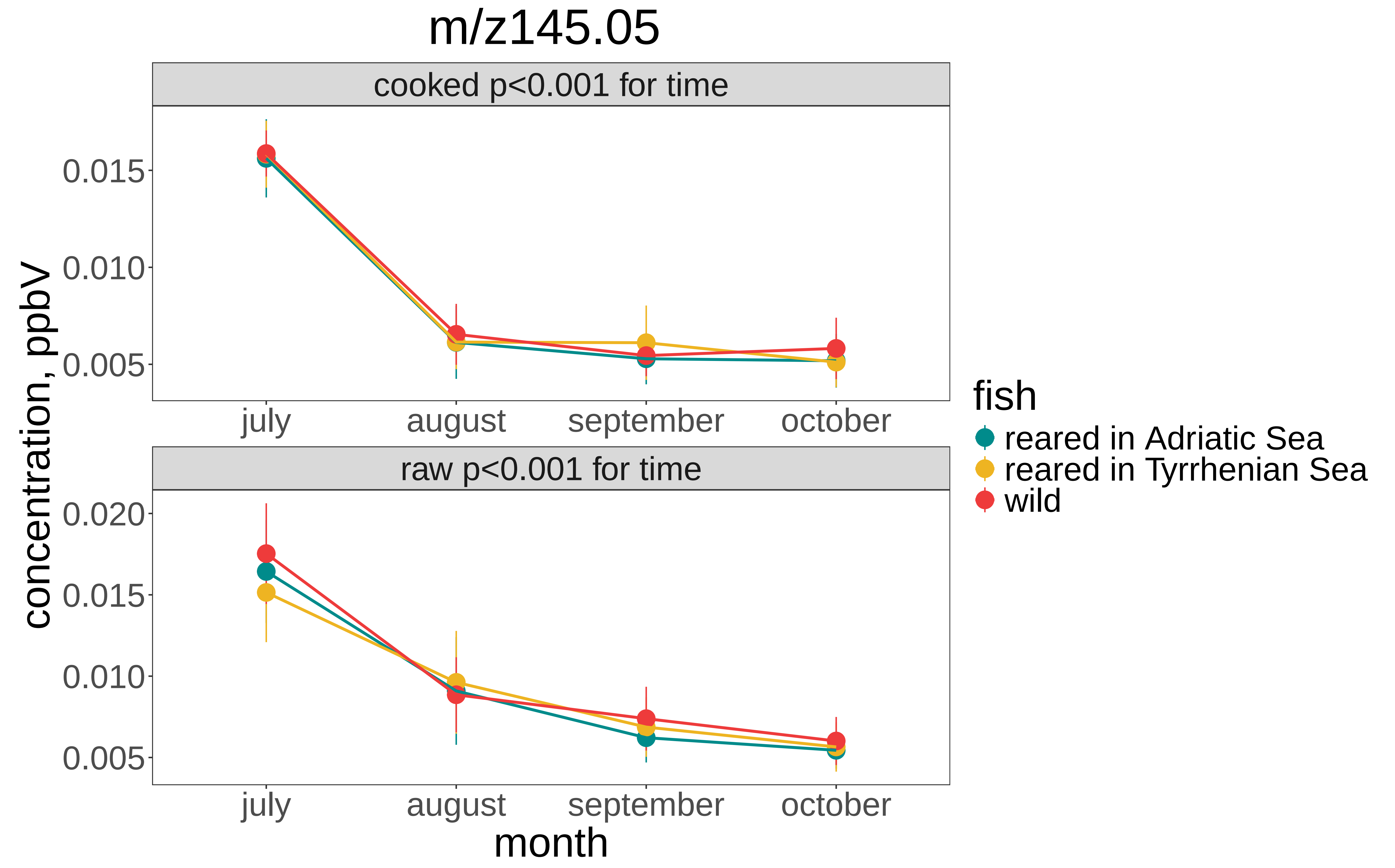

m/z145.05
cooked p<0.001 for time
raw p<0.001 for time
concentration, ppbV
0.015
0.010
0.005
0.020
0.015
0.010
0.005
july
august
september
october
july
august
september
october
month
fish
reared in Adriatic Sea
reared in Tyrrhenian Sea
wild

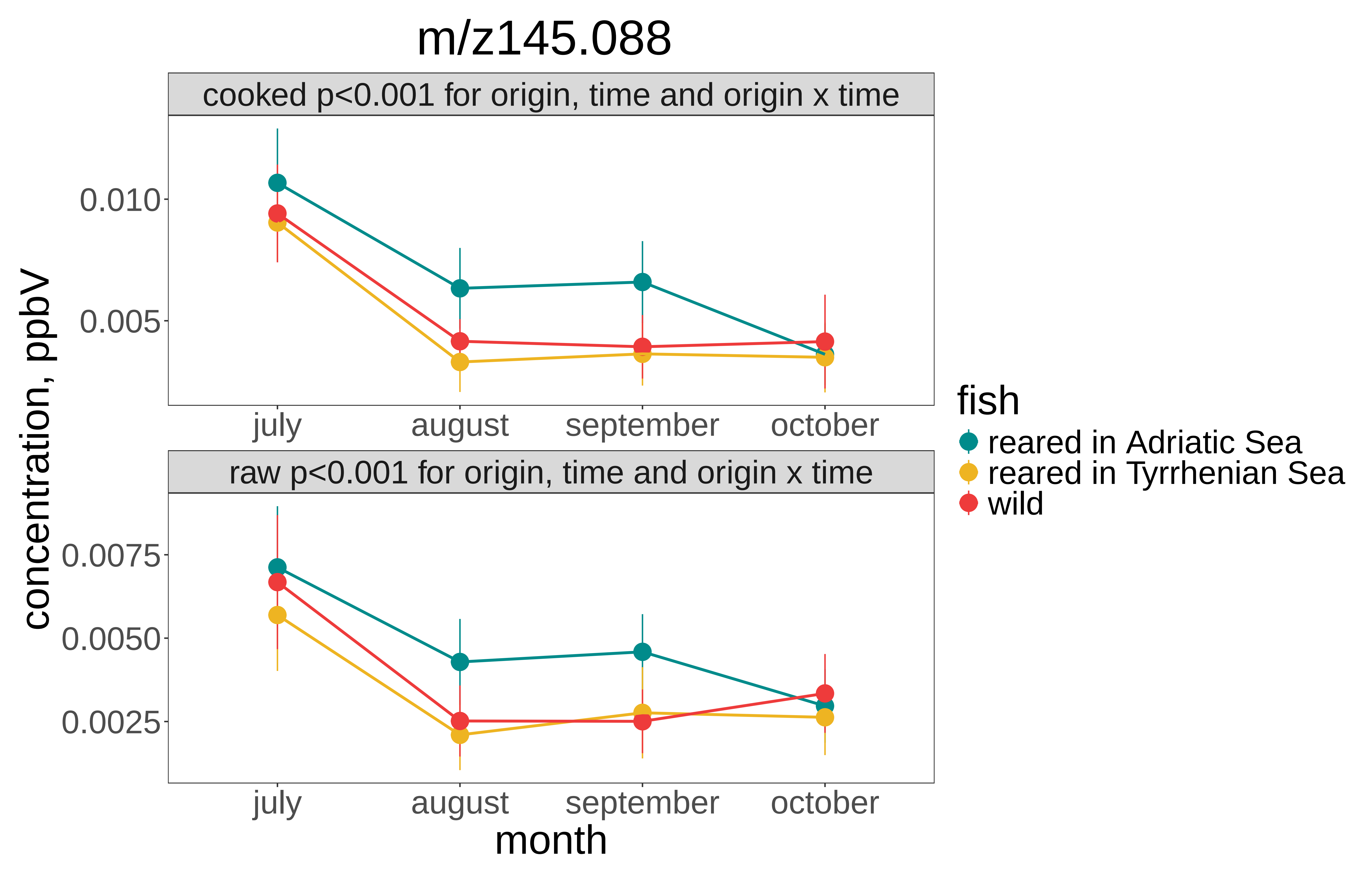

m/z145.088
cooked p<0.001 for origin, time and origin x time
raw p<0.001 for origin, time and origin x time
concentration, ppbV
0.010
0.005
0.0075
0.0050
0.0025
july
august
september
october
month
fish
reared in Adriatic Sea
reared in Tyrrhenian Sea
wild

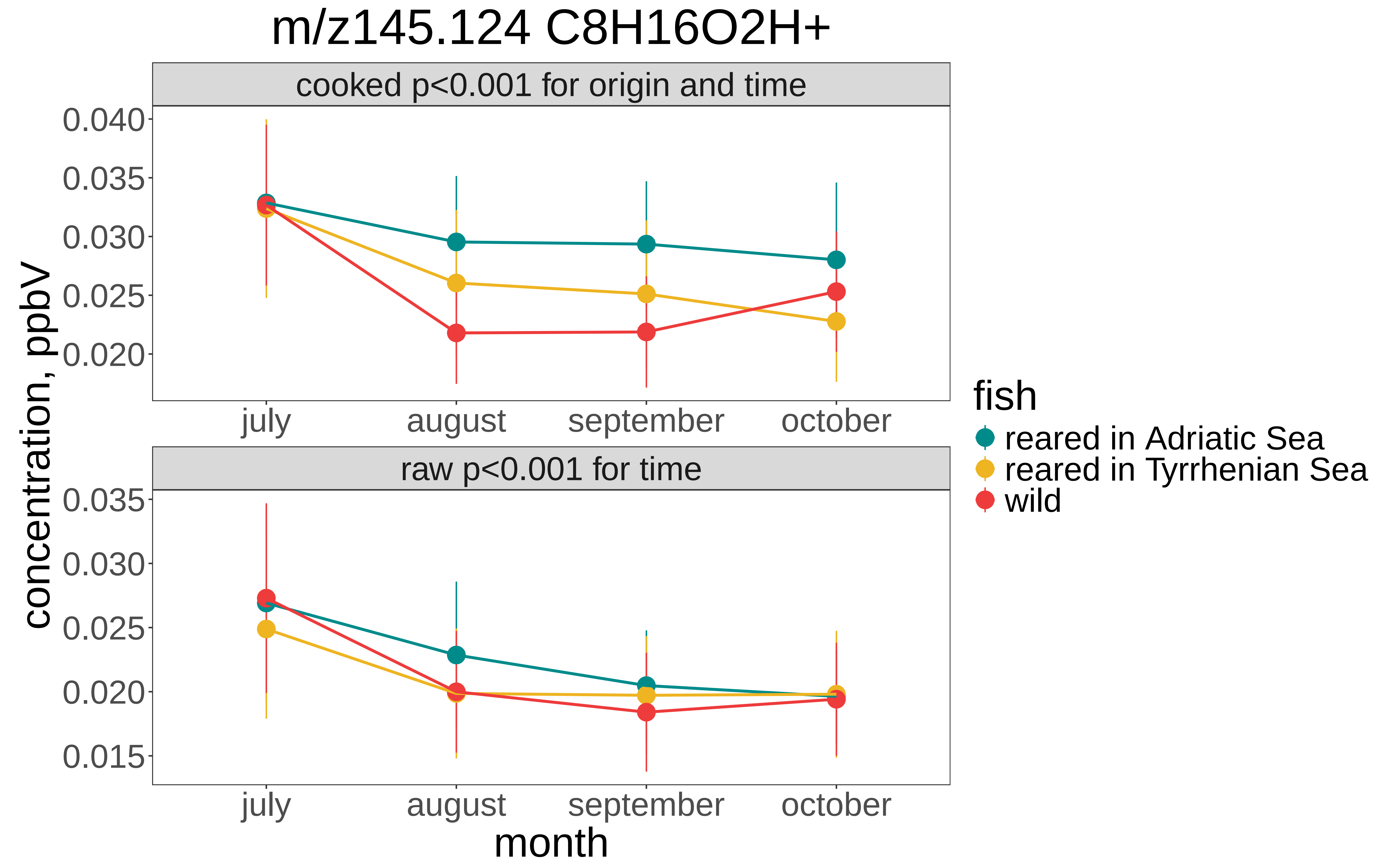

m/z145.124 C8H16O2H+
cooked p<0.001 for origin and time
raw p<0.001 for time
0.040
0.035
0.030
0.025
0.020
0.015
concentration, ppbV
july
august
september
october
month
fish
reared in Adriatic Sea
reared in Tyrrhenian Sea
wild

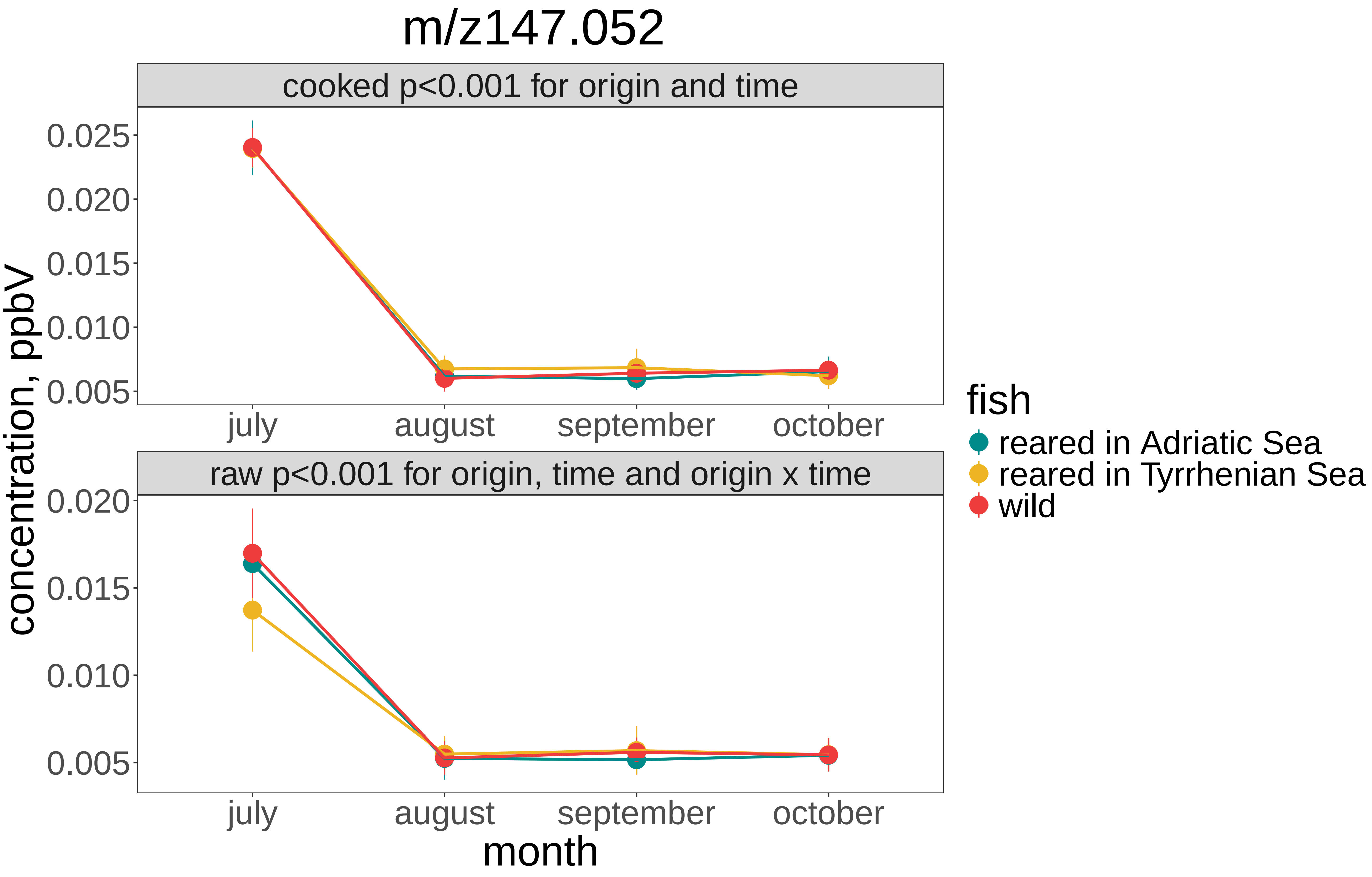

m/z147.052
cooked p<0.001 for origin and time
raw p<0.001 for origin, time and origin x time
concentration, ppbV
0.025
0.020
0.015
0.010
0.005
july
august
september
october
month
fish
reared in Adriatic Sea
reared in Tyrrhenian Sea
wild

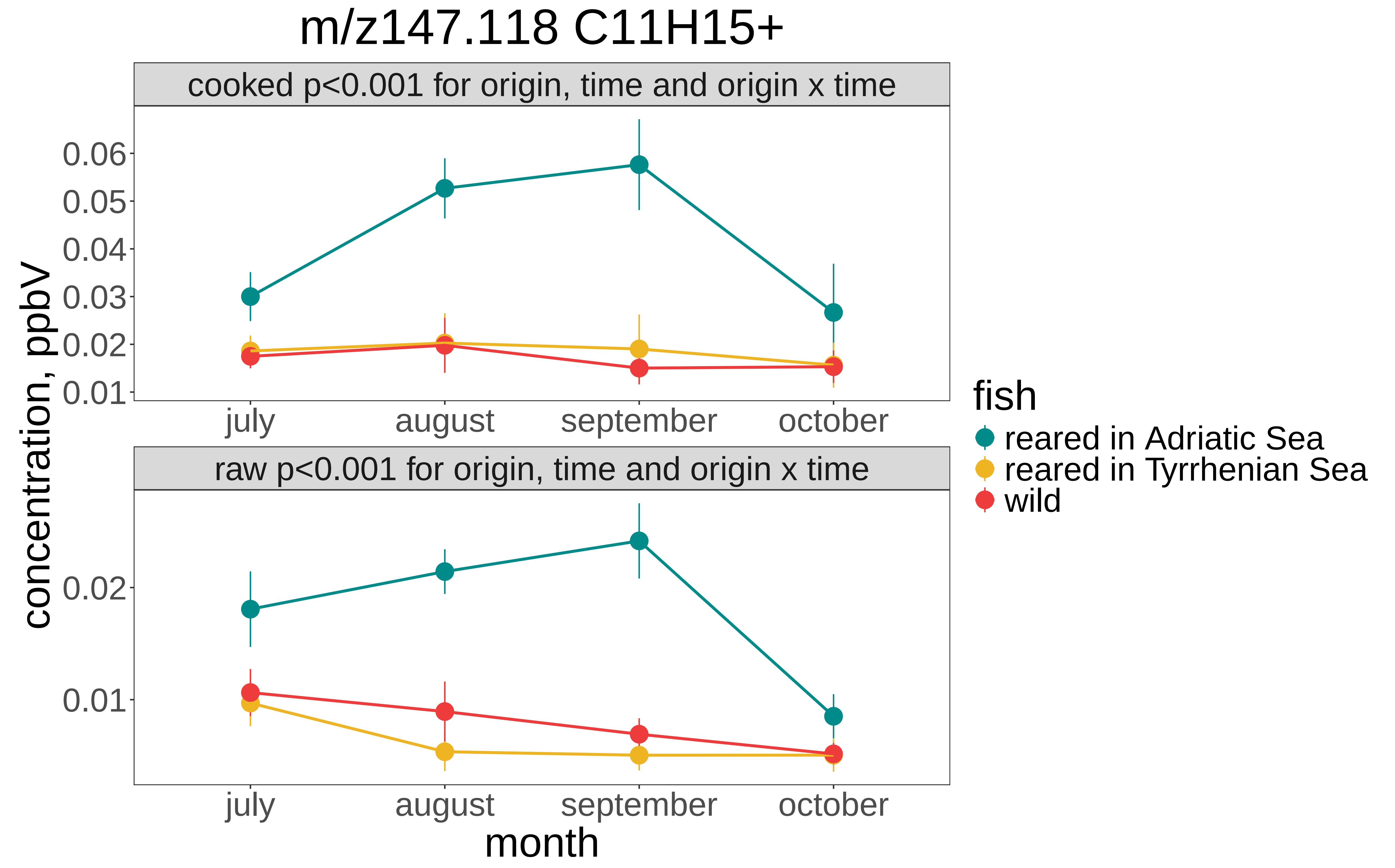

m/z147.118 C11H15+
cooked p<0.001 for origin, time and origin x time
raw p<0.001 for origin, time and origin x time
0.06
0.05
0.04
0.03
0.02
0.01
concentration, ppbV
july
august
september
october
month
fish
reared in Adriatic Sea
reared in Tyrrhenian Sea
wild

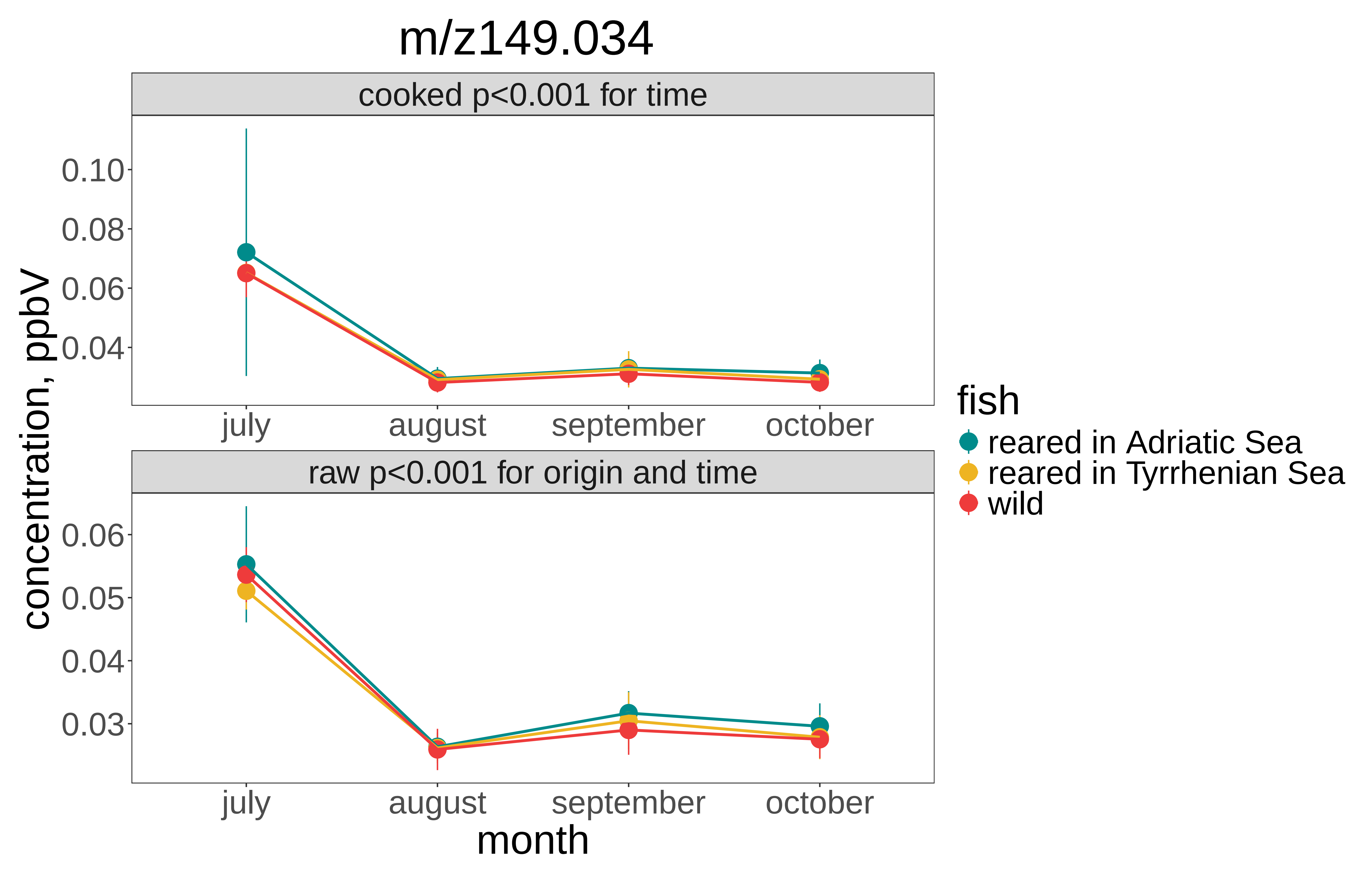
m/z149.034
cooked p<0.001 for time
raw p<0.001 for origin and time
concentration, ppbV
0.10
0.08
0.06
0.04
0.06
0.05
0.04
0.03
july
august
september
october
month
fish
reared in Adriatic Sea
reared in Tyrrhenian Sea
wild

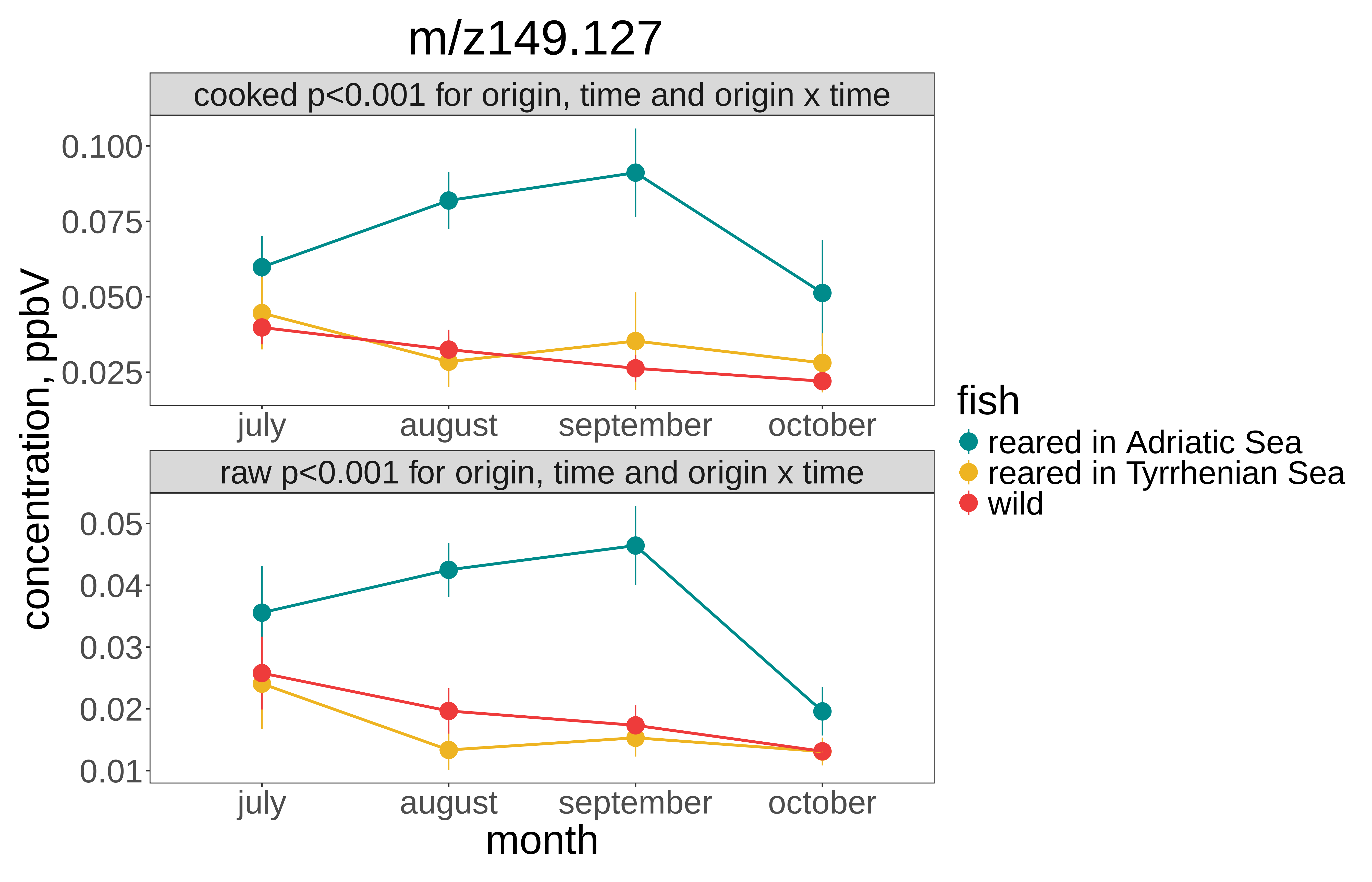
m/z149.127
cooked p<0.001 for origin, time and origin x time
raw p<0.001 for origin, time and origin x time
concentration, ppbV
0.100
0.075
0.050
0.025
0.05
0.04
0.03
0.02
0.01
july
august
september
october
month
fish
reared in Adriatic Sea
reared in Tyrrhenian Sea
wild

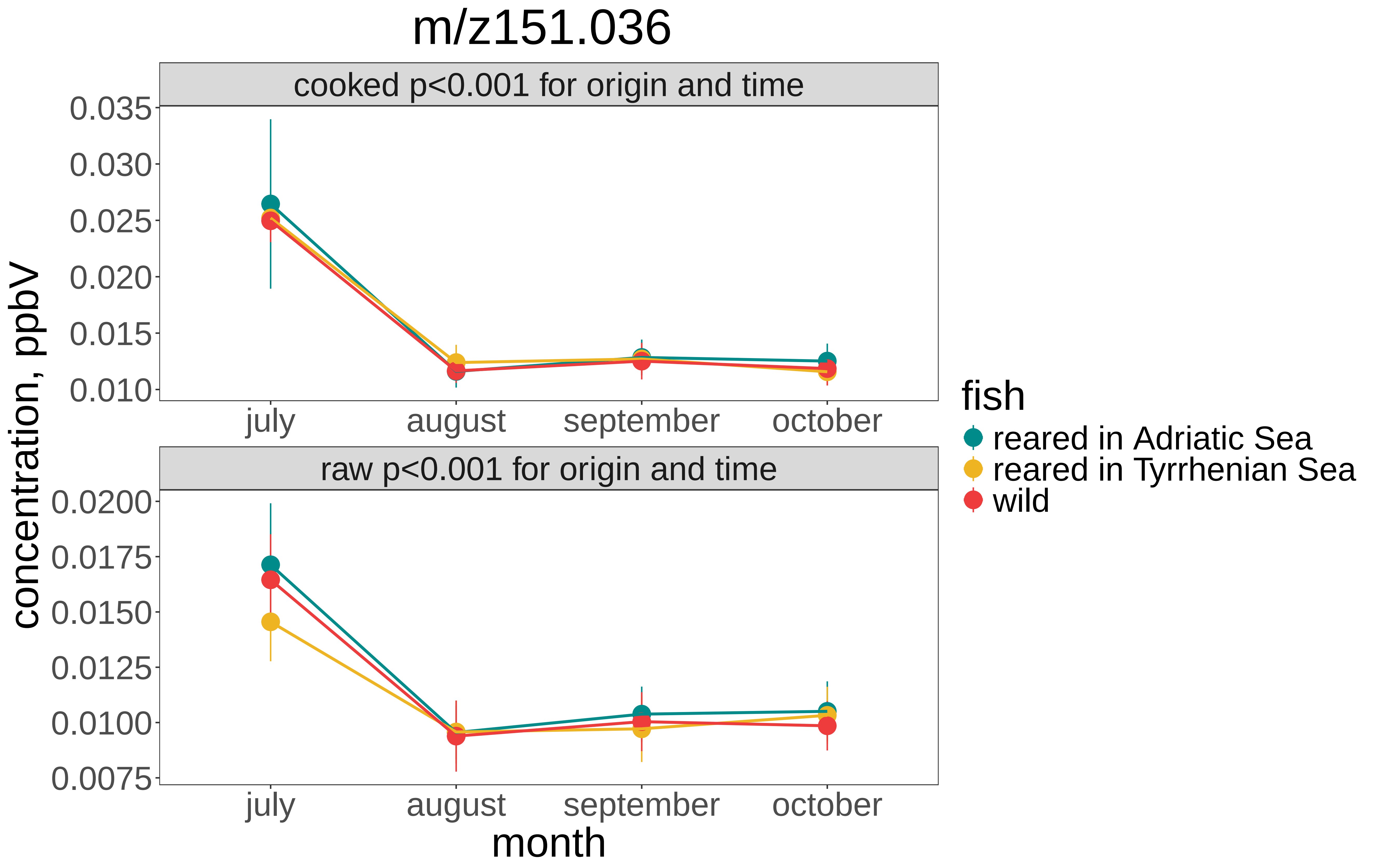
m/z151.036
cooked p<0.001 for origin and time
0.035
0.030
0.025
0.020
0.015
0.010
july
august
september
october
raw p<0.001 for origin and time
0.0200
0.0175
0.0150
0.0125
0.0100
0.0075
concentration, ppbV
month
fish
reared in Adriatic Sea
reared in Tyrrhenian Sea
wild

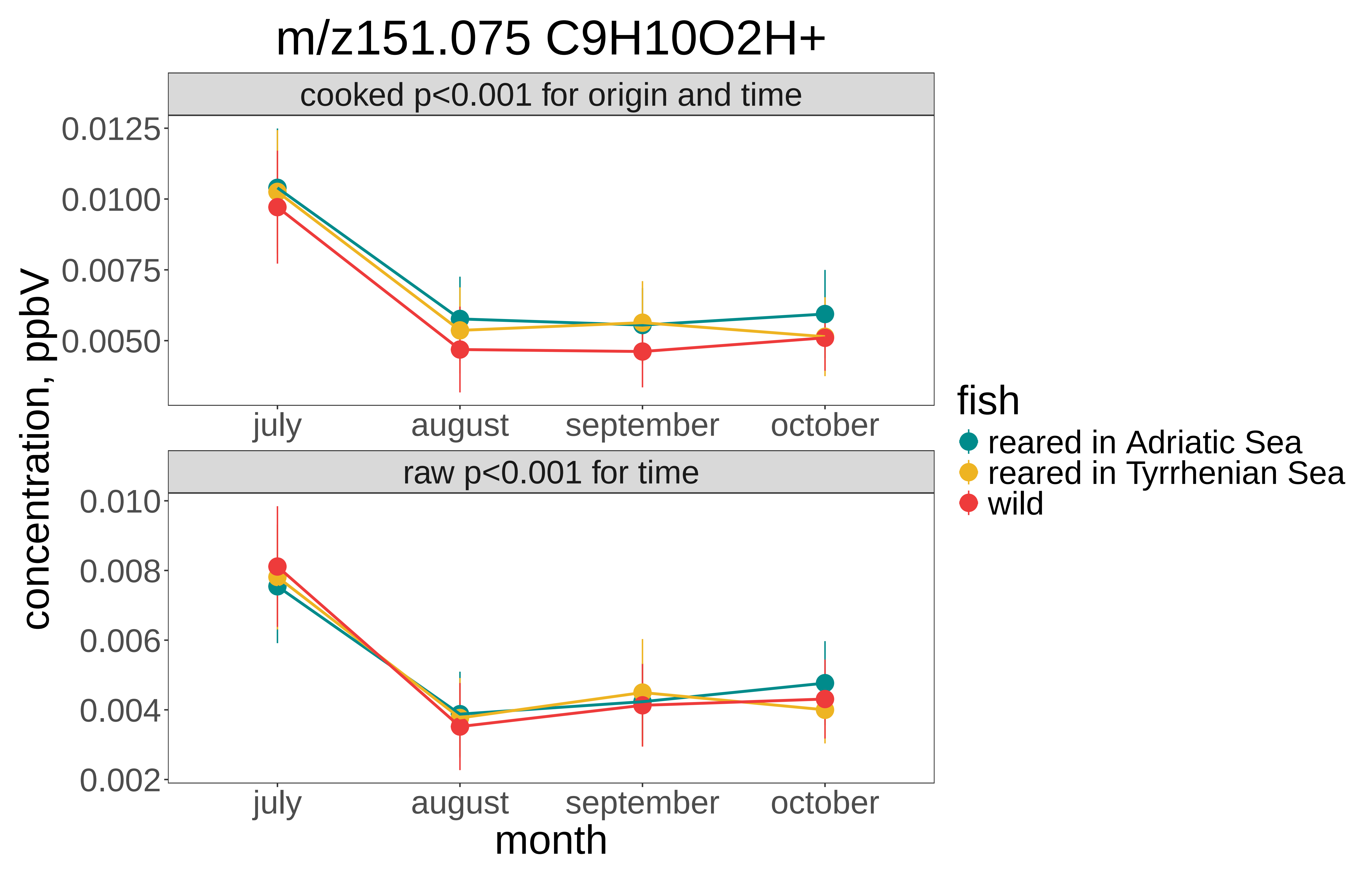

m/z151.075 C9H10O2H+
cooked p<0.001 for origin and time
0.0125
0.0100
0.0075
0.0050
july
august
september
october
raw p<0.001 for time
0.010
0.008
0.006
0.004
0.002
july
august
september
october
month
concentration, ppbV
fish
reared in Adriatic Sea
reared in Tyrrhenian Sea
wild

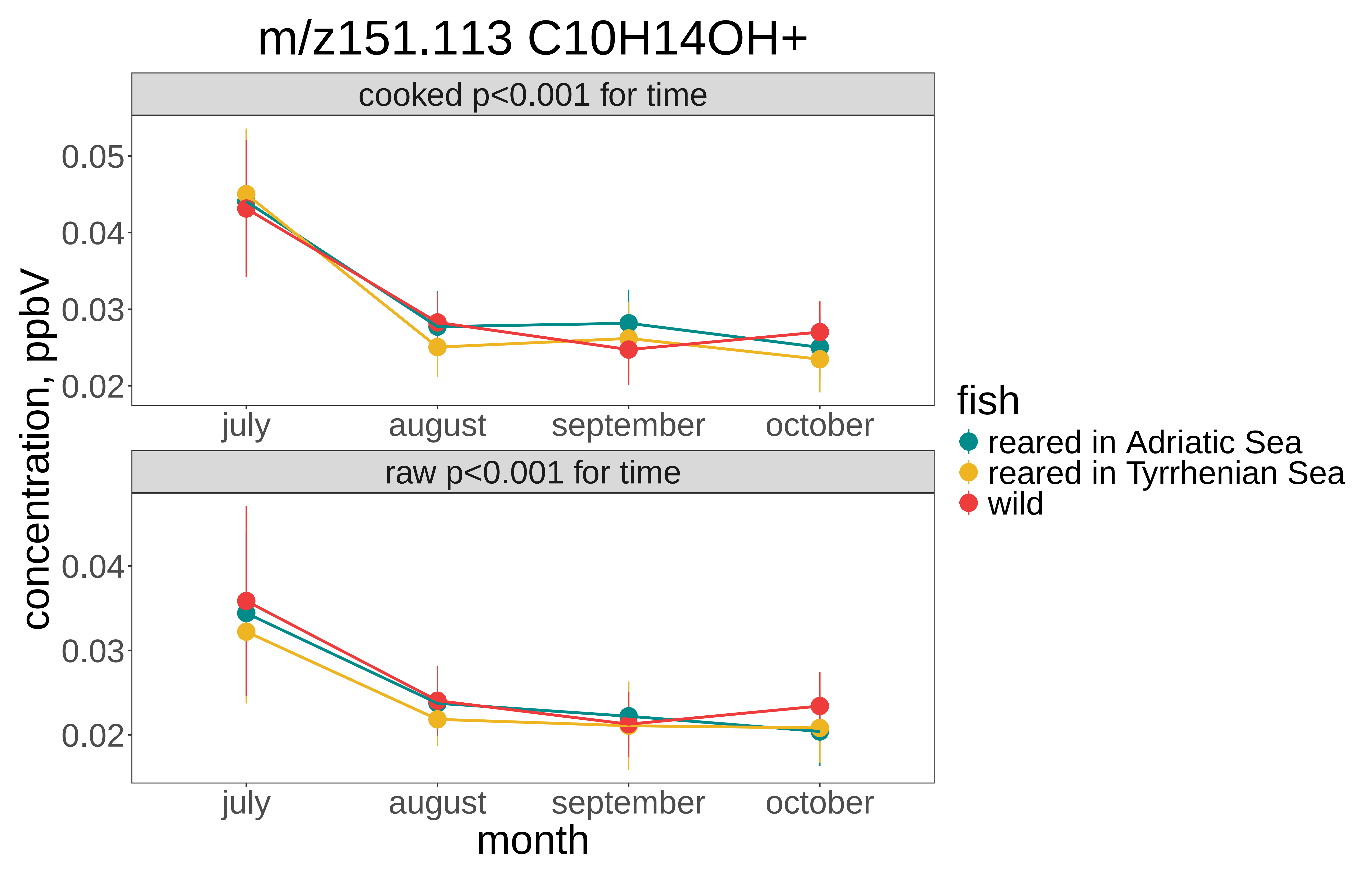
m/z151.113 C10H14OH+
cooked p<0.001 for time
raw p<0.001 for time
concentration, ppbV
0.05
0.04
0.03
0.02
july
august
september
october
month
fish
reared in Adriatic Sea
reared in Tyrrhenian Sea
wild

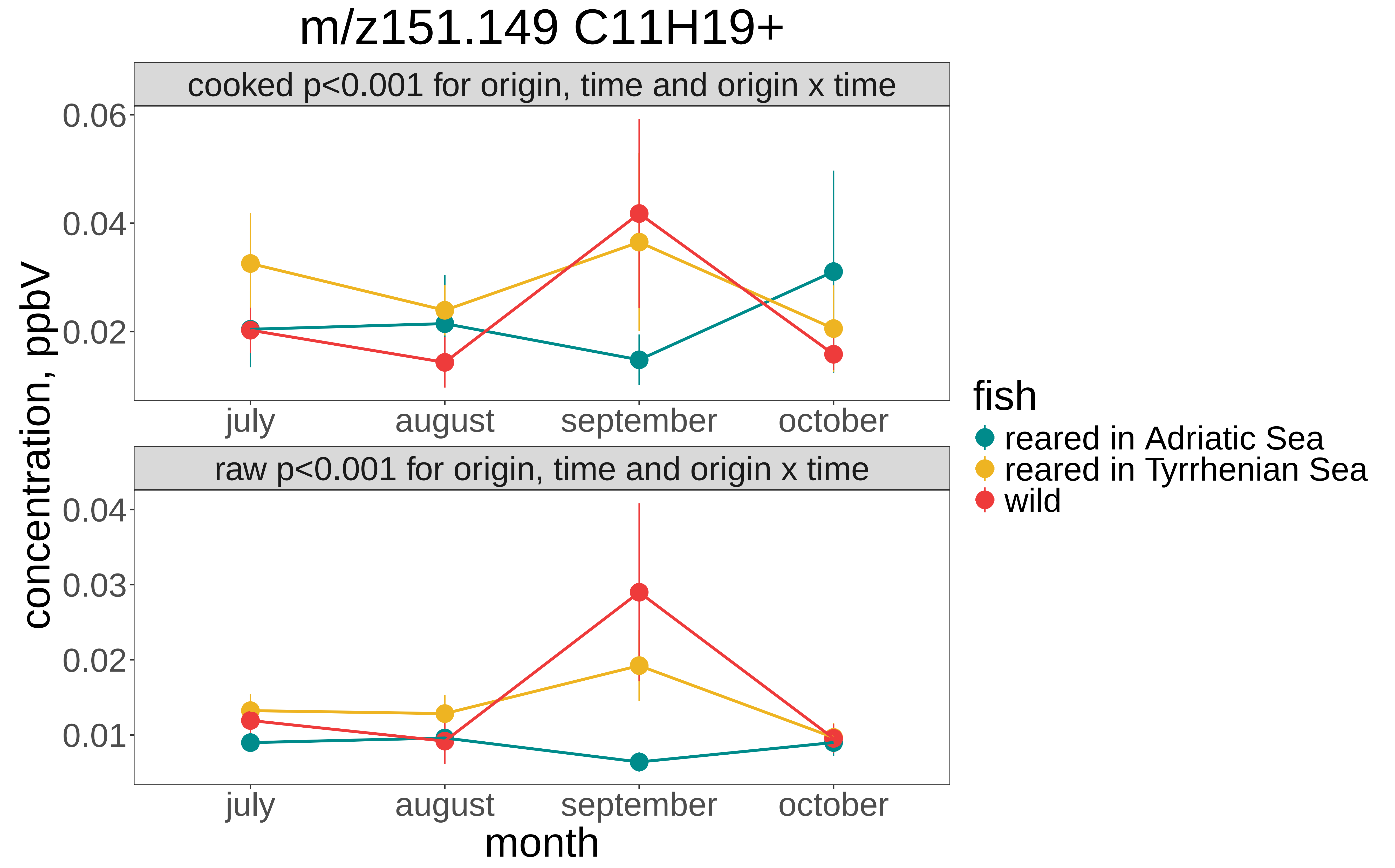
m/z151.149 C11H19+
cooked p<0.001 for origin, time and origin x time
raw p<0.001 for origin, time and origin x time
concentration, ppbV
0.06
0.04
0.02
0.04
0.03
0.02
0.01
july
august
september
october
month
fish
reared in Adriatic Sea
reared in Tyrrhenian Sea
wild

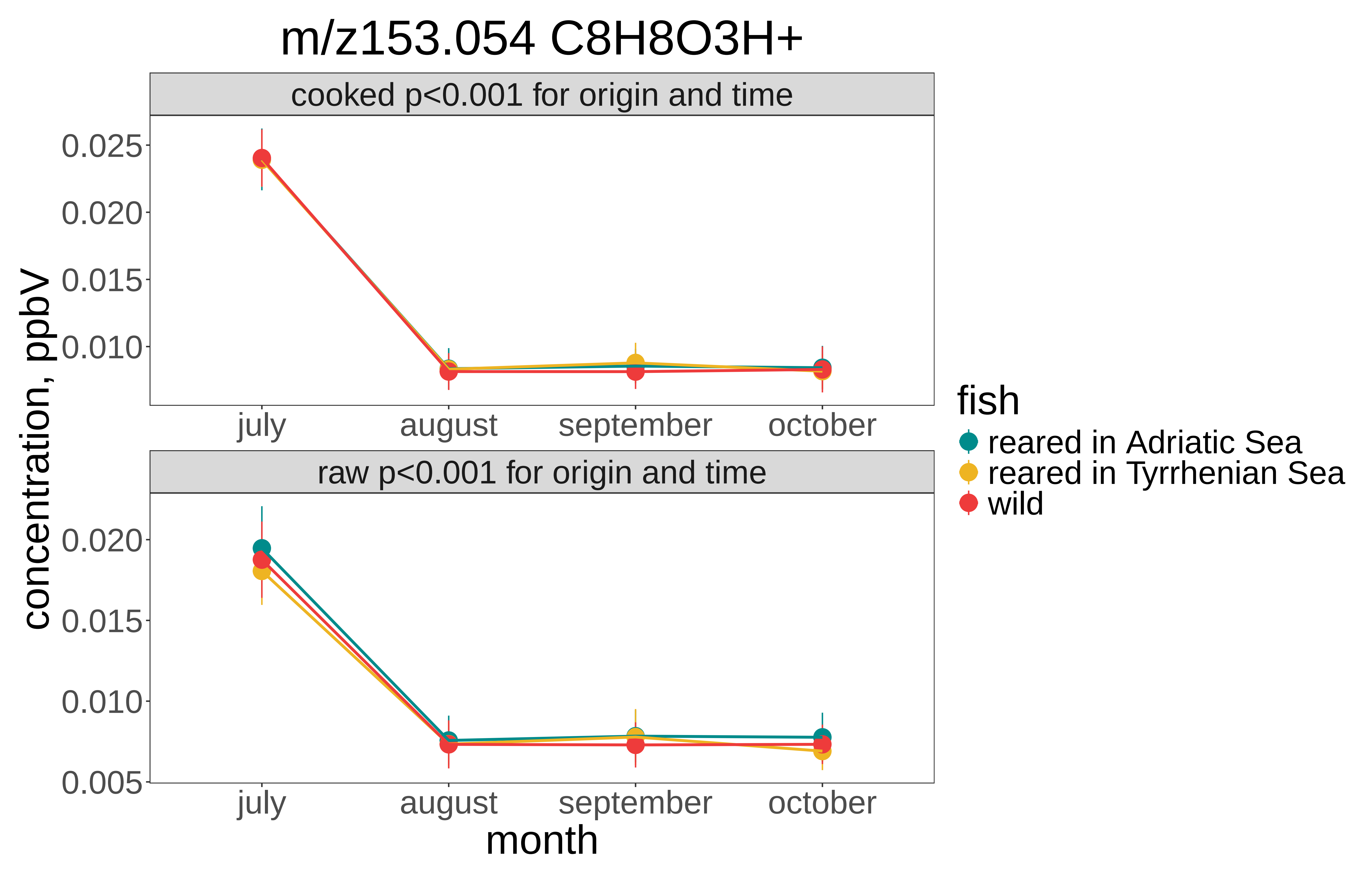
m/z153.054 C8H8O3H+
cooked p<0.001 for origin and time
raw p<0.001 for origin and time
concentration, ppbV
0.025
0.020
0.015
0.010
0.005
july
august
september
october
month
fish
reared in Adriatic Sea
reared in Tyrrhenian Sea
wild

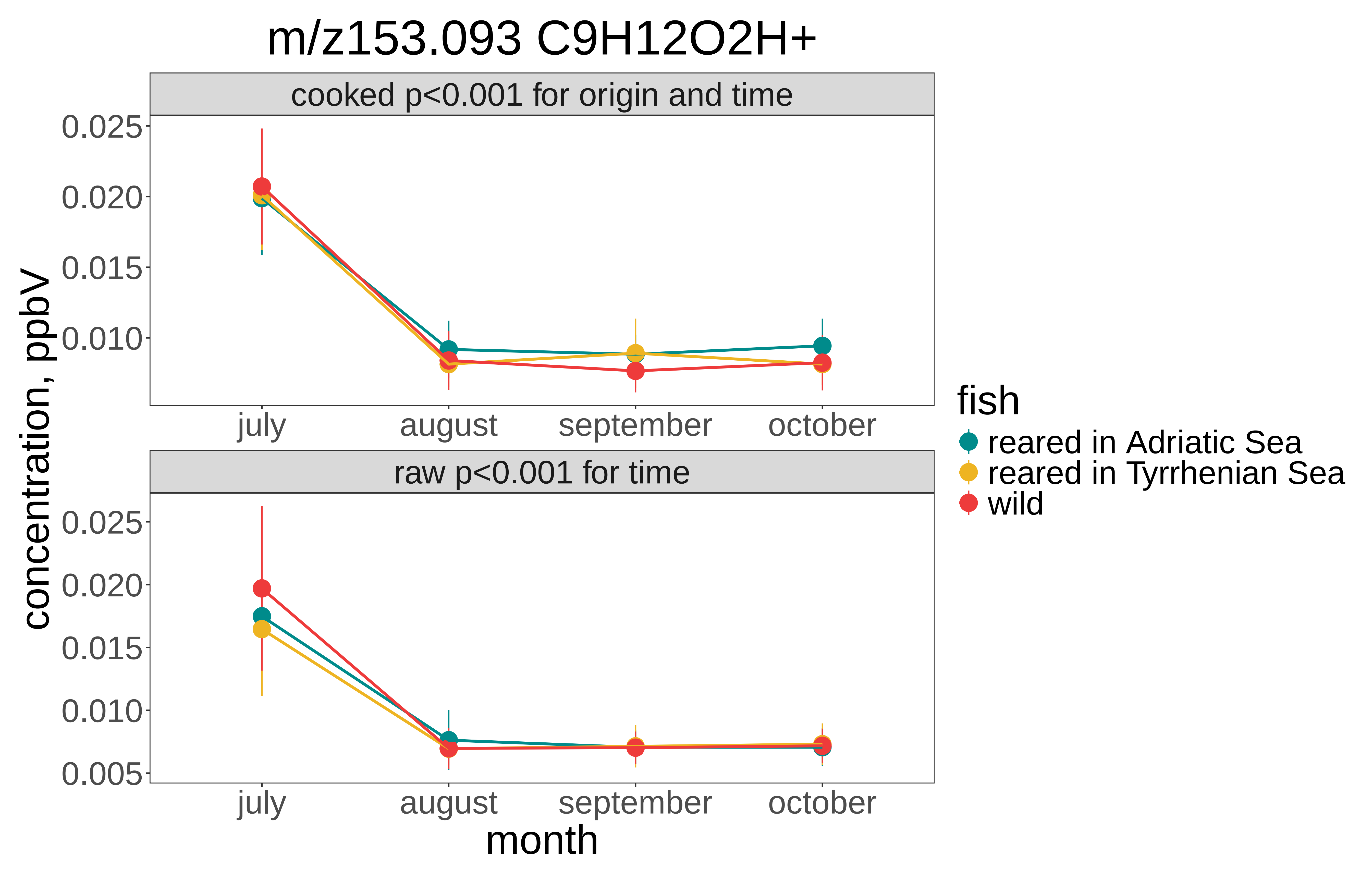

m/z153.093 C9H12O2H+
cooked p<0.001 for origin and time
raw p<0.001 for time
concentration, ppbV
0.025
0.020
0.015
0.010
0.005
july
august
september
october
month
fish
reared in Adriatic Sea
reared in Tyrrhenian Sea
wild

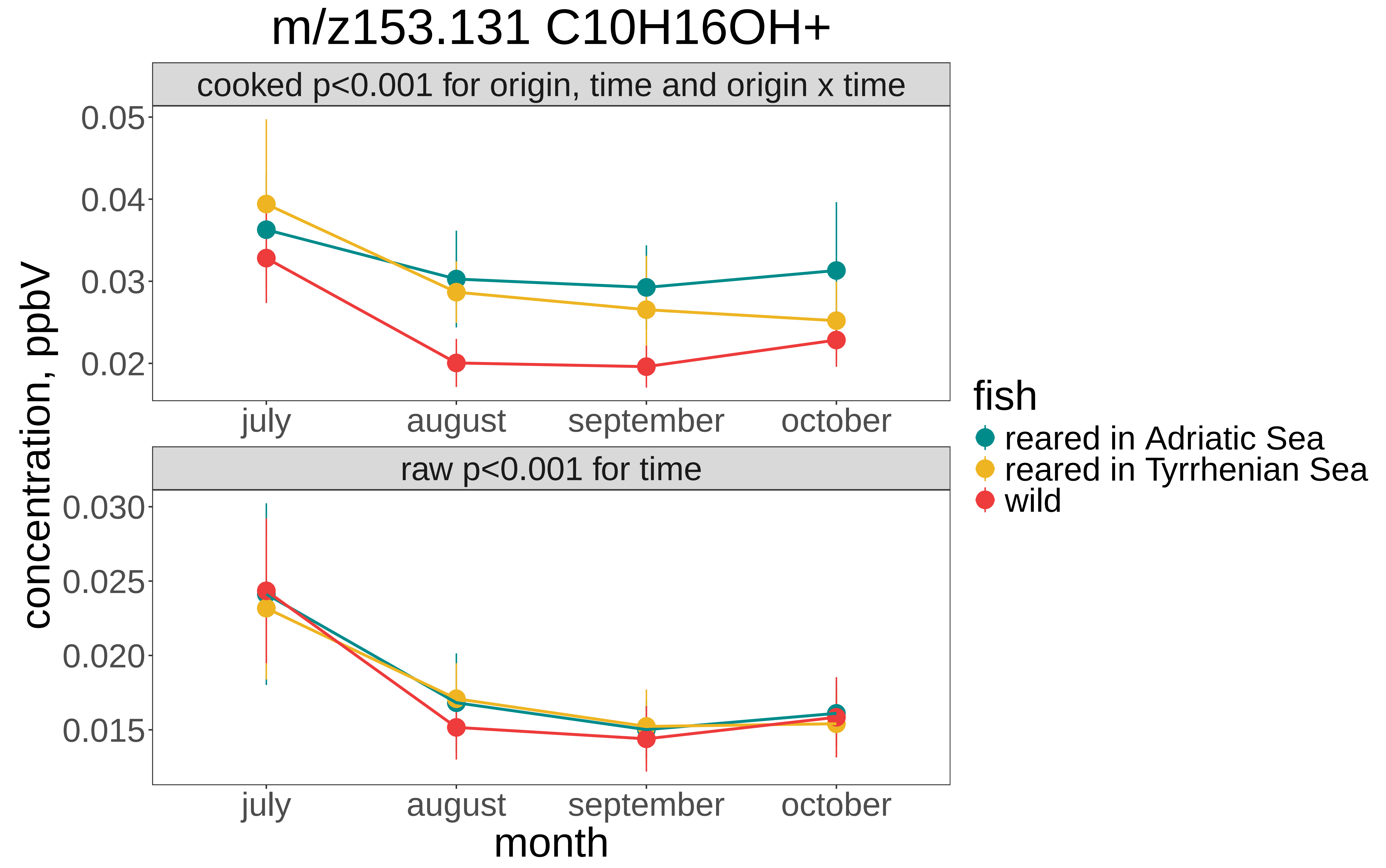
m/z153.131 C10H16OH+
cooked p<0.001 for origin, time and origin x time
0.05
0.04
0.03
0.02
july
august
september
october
raw p<0.001 for time
0.030
0.025
0.020
0.015
july
august
september
october
concentration, ppbV
month
fish
reared in Adriatic Sea
reared in Tyrrhenian Sea
wild

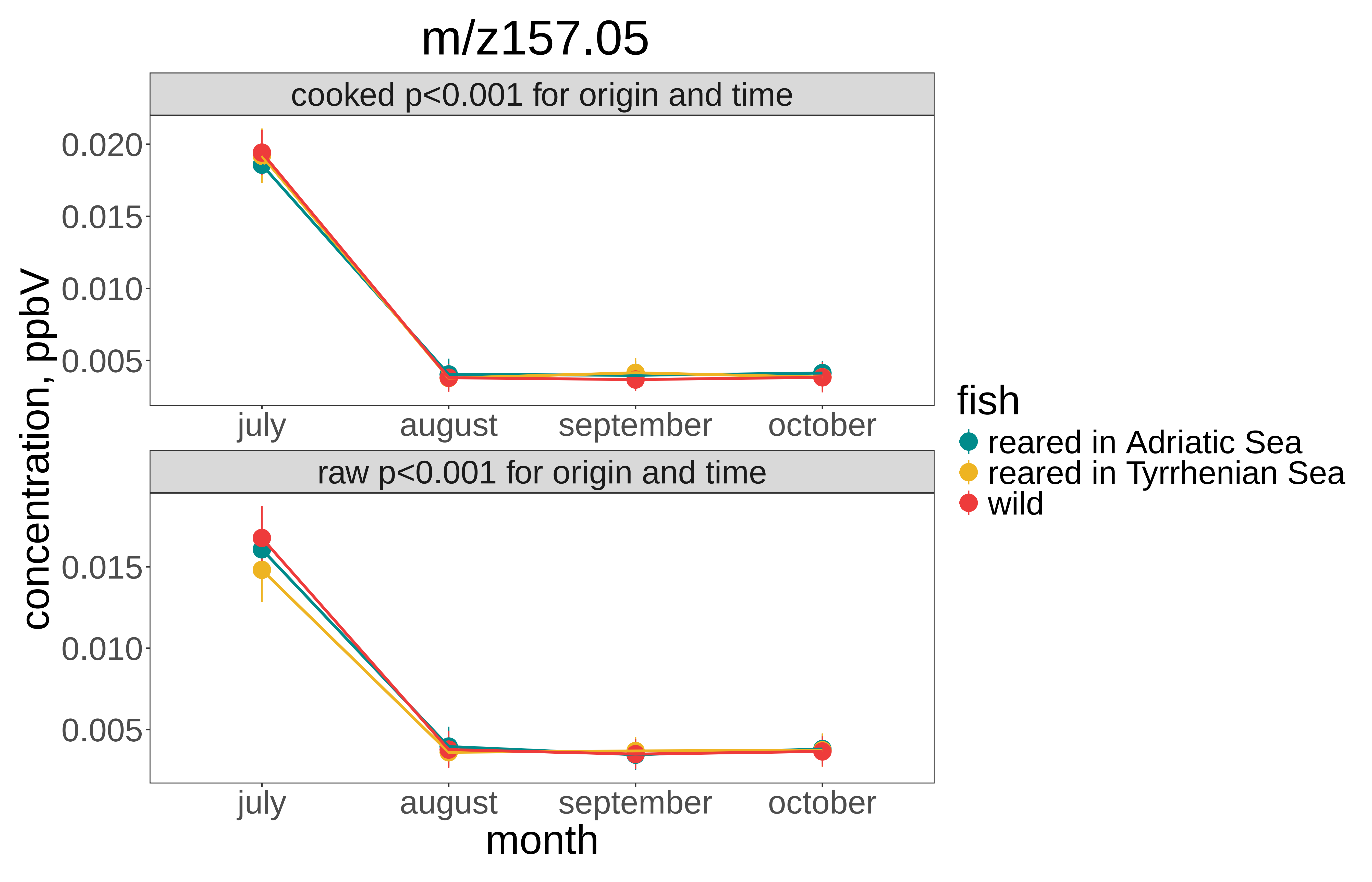
m/z157.05
cooked p<0.001 for origin and time
raw p<0.001 for origin and time
concentration, ppbV
0.020
0.015
0.010
0.005
july
august
september
october
month
fish
reared in Adriatic Sea
reared in Tyrrhenian Sea
wild

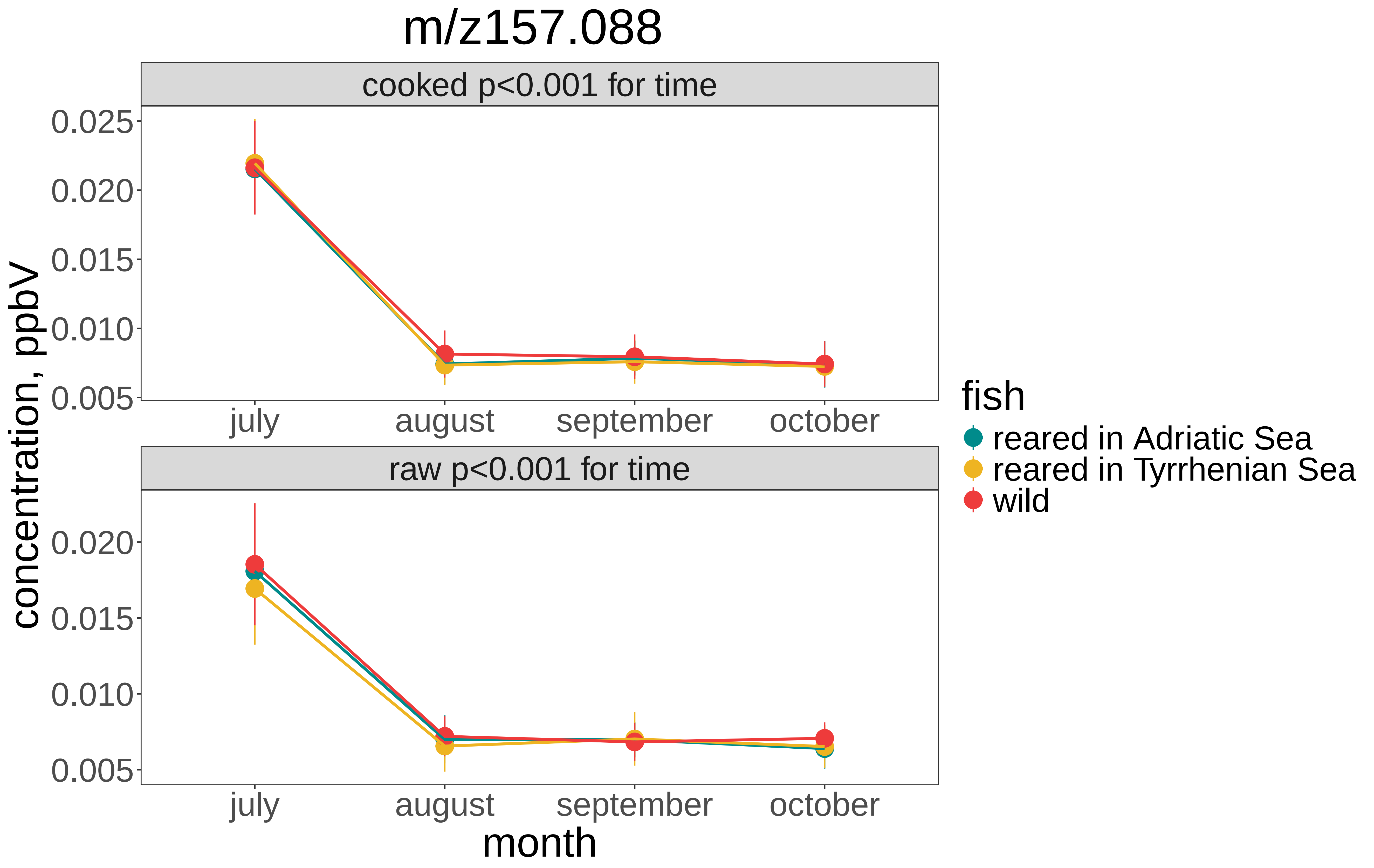

m/z157.088
cooked p<0.001 for time
raw p<0.001 for time
0.025
0.020
0.015
0.010
0.005
july
august
september
october
concentration, ppbV
month
fish
reared in Adriatic Sea
reared in Tyrrhenian Sea
wild

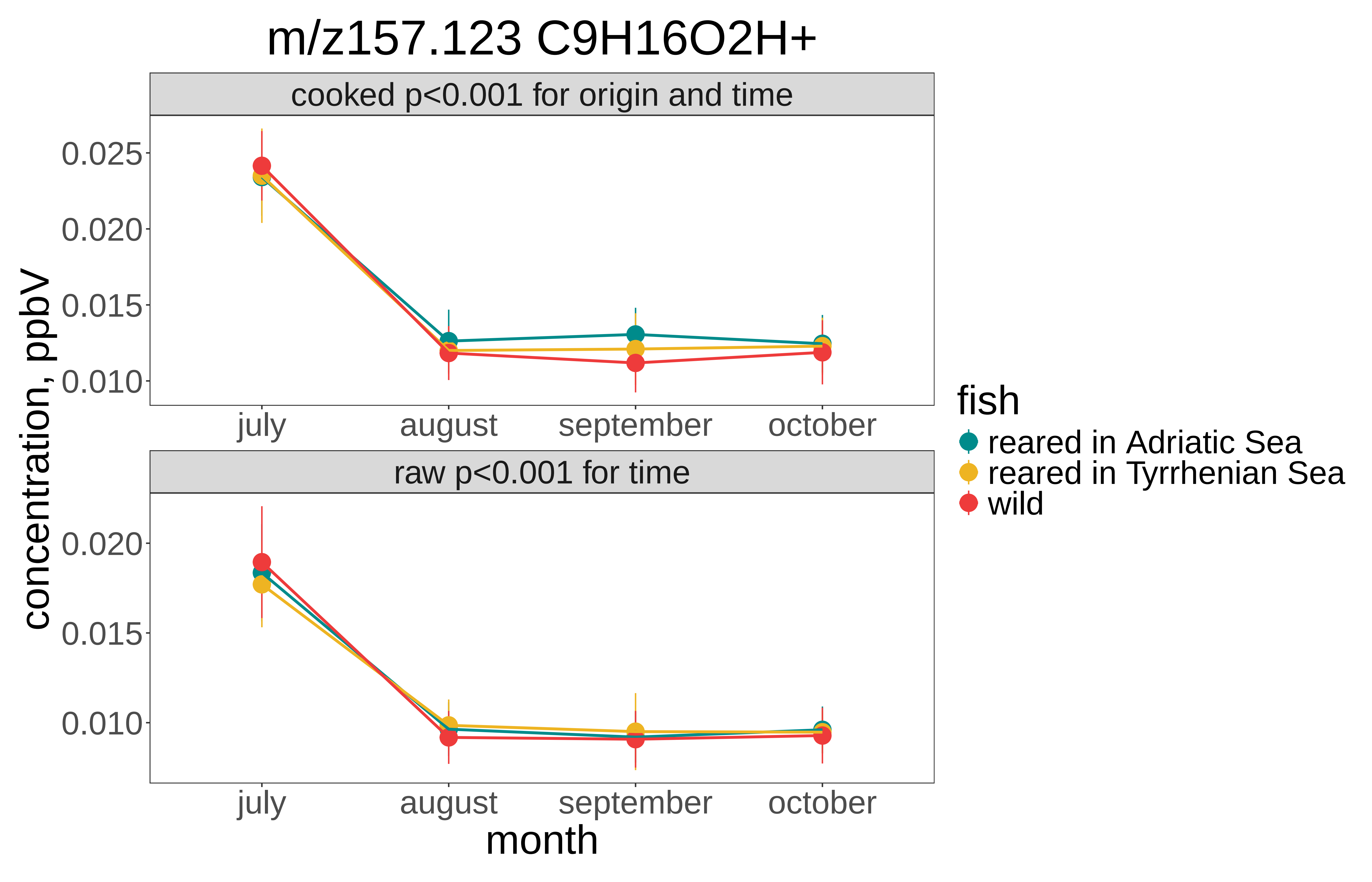

m/z157.123 C9H16O2H+
cooked p<0.001 for origin and time
raw p<0.001 for time
concentration, ppbV
0.025
0.020
0.015
0.010
july
august
september
october
month
fish
reared in Adriatic Sea
reared in Tyrrhenian Sea
wild

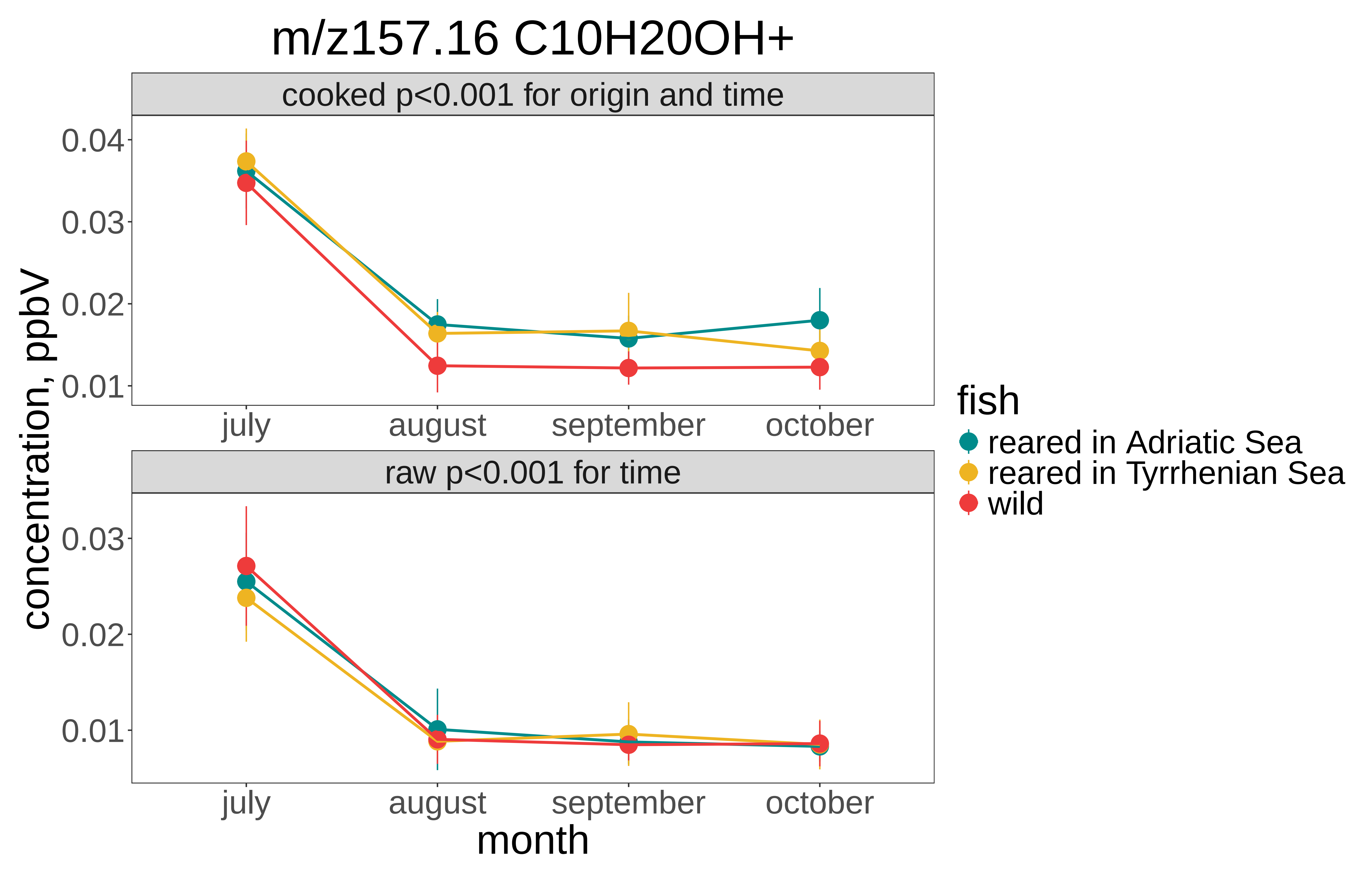

m/z157.16 C10H20OH+
cooked p<0.001 for origin and time
raw p<0.001 for time
concentration, ppbV
0.04
0.03
0.02
0.01
july
august
september
october
month
fish
reared in Adriatic Sea
reared in Tyrrhenian Sea
wild

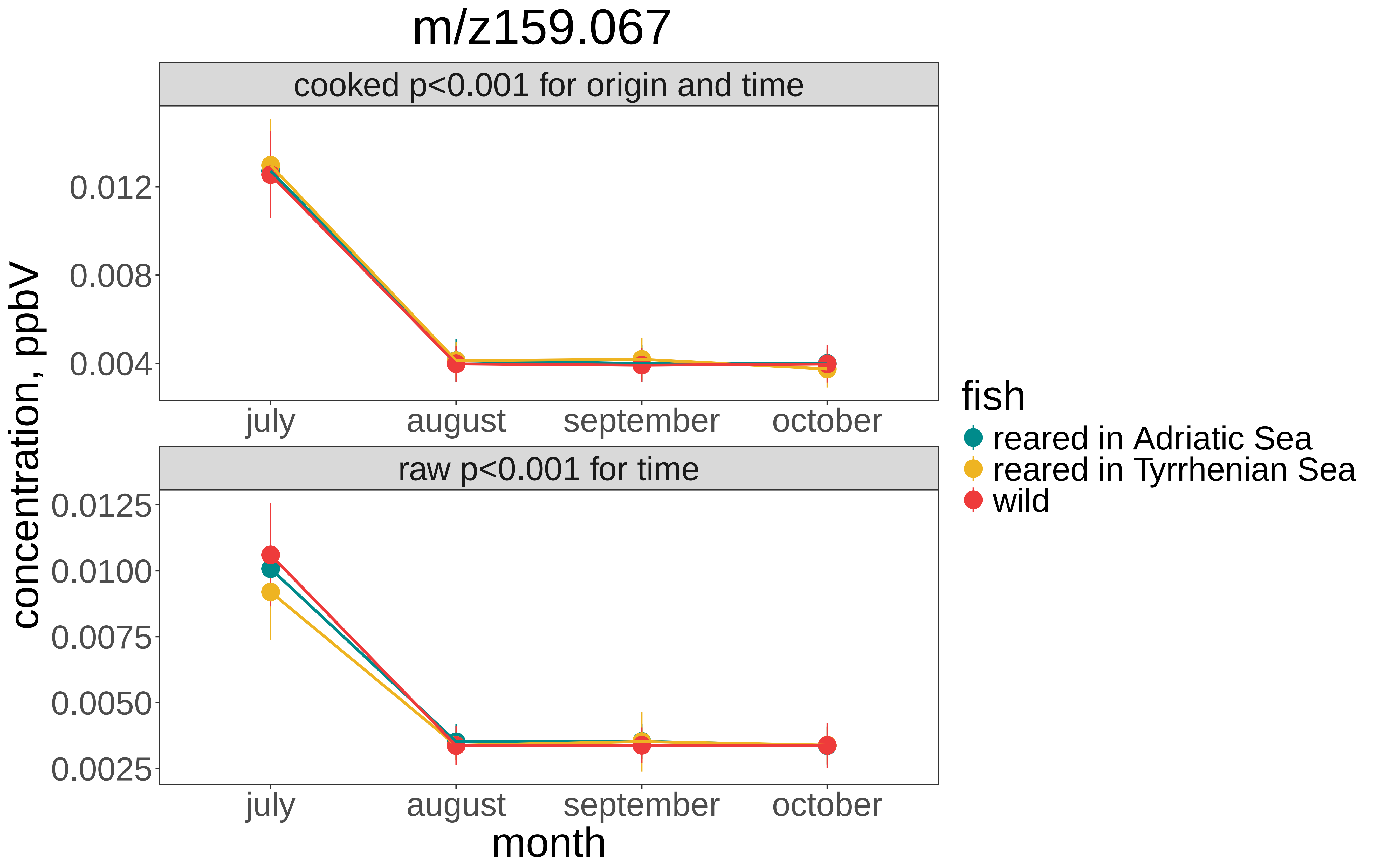

m/z159.067
cooked p<0.001 for origin and time
raw p<0.001 for time
concentration, ppbV
0.012
0.008
0.004
0.0125
0.0100
0.0075
0.0050
0.0025
july
august
september
october
month
fish
reared in Adriatic Sea
reared in Tyrrhenian Sea
wild

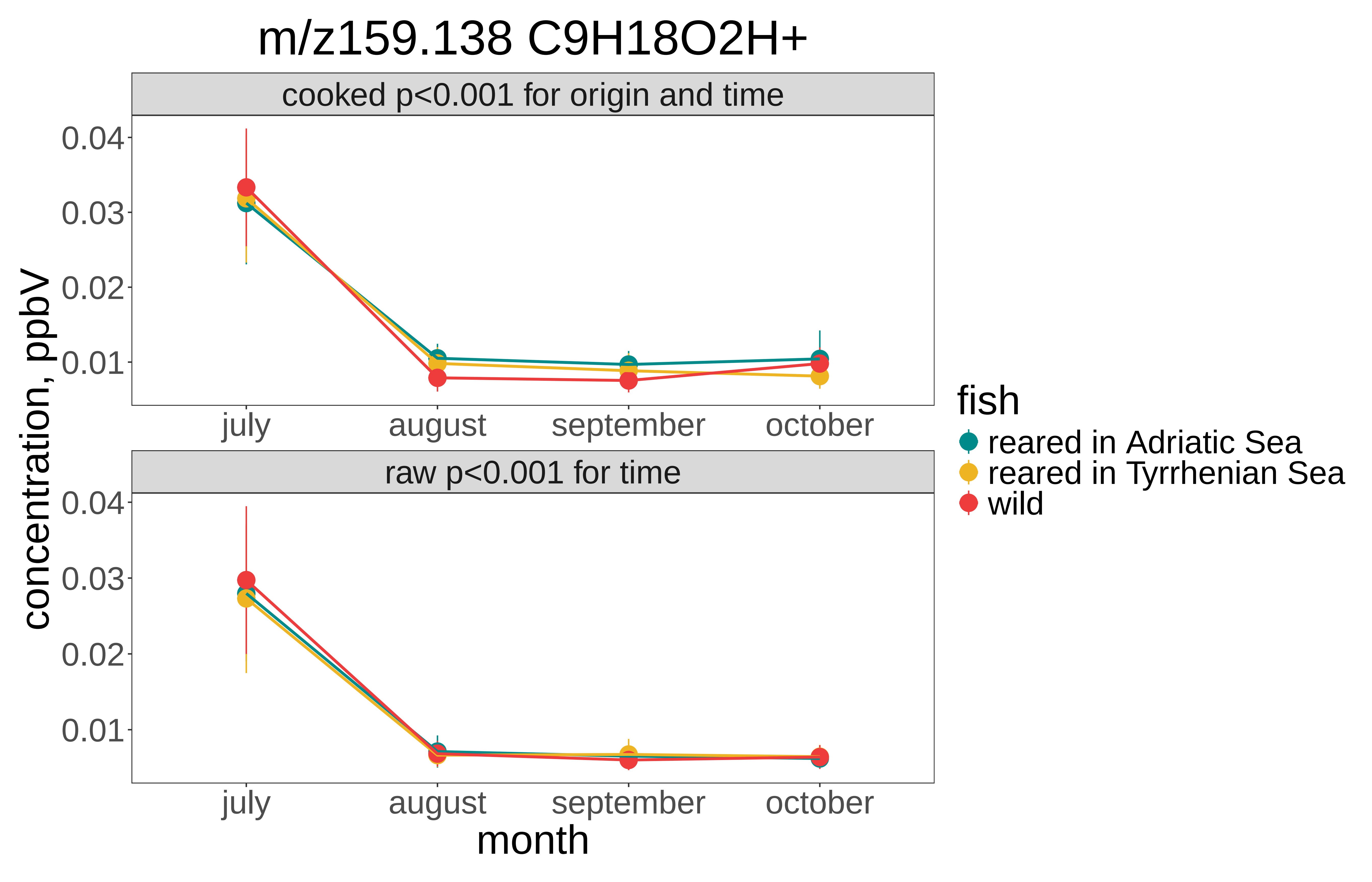

m/z159.138 C9H18O2H+
cooked p<0.001 for origin and time
raw p<0.001 for time
0.04
0.03
0.02
0.01
concentration, ppbV
july
august
september
october
month
fish
reared in Adriatic Sea
reared in Tyrrhenian Sea
wild

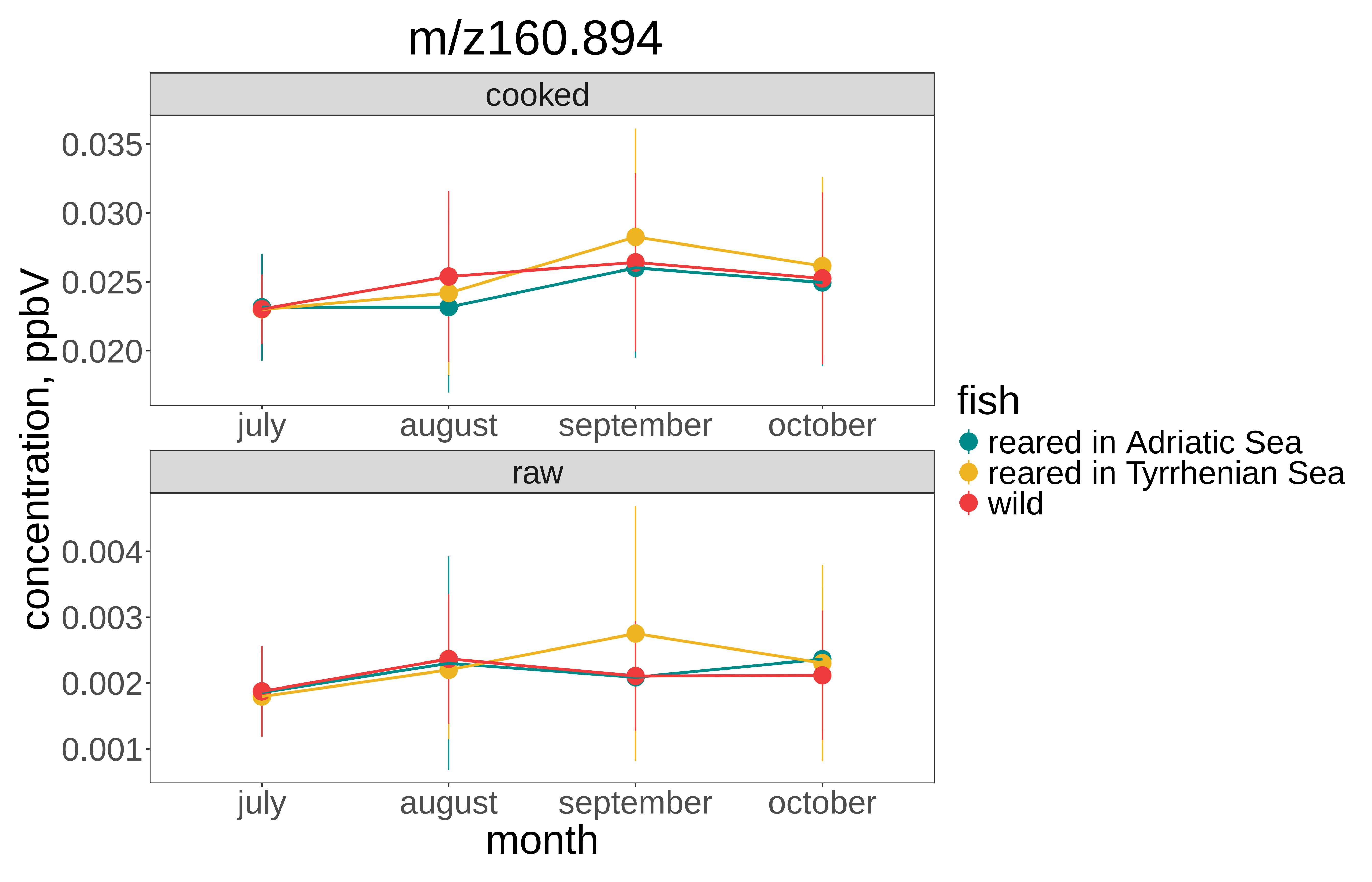
m/z160.894
cooked
raw
0.035
0.030
0.025
0.020
0.004
0.003
0.002
0.001
concentration, ppbV
july
august
september
october
month
fish
reared in Adriatic Sea
reared in Tyrrhenian Sea
wild

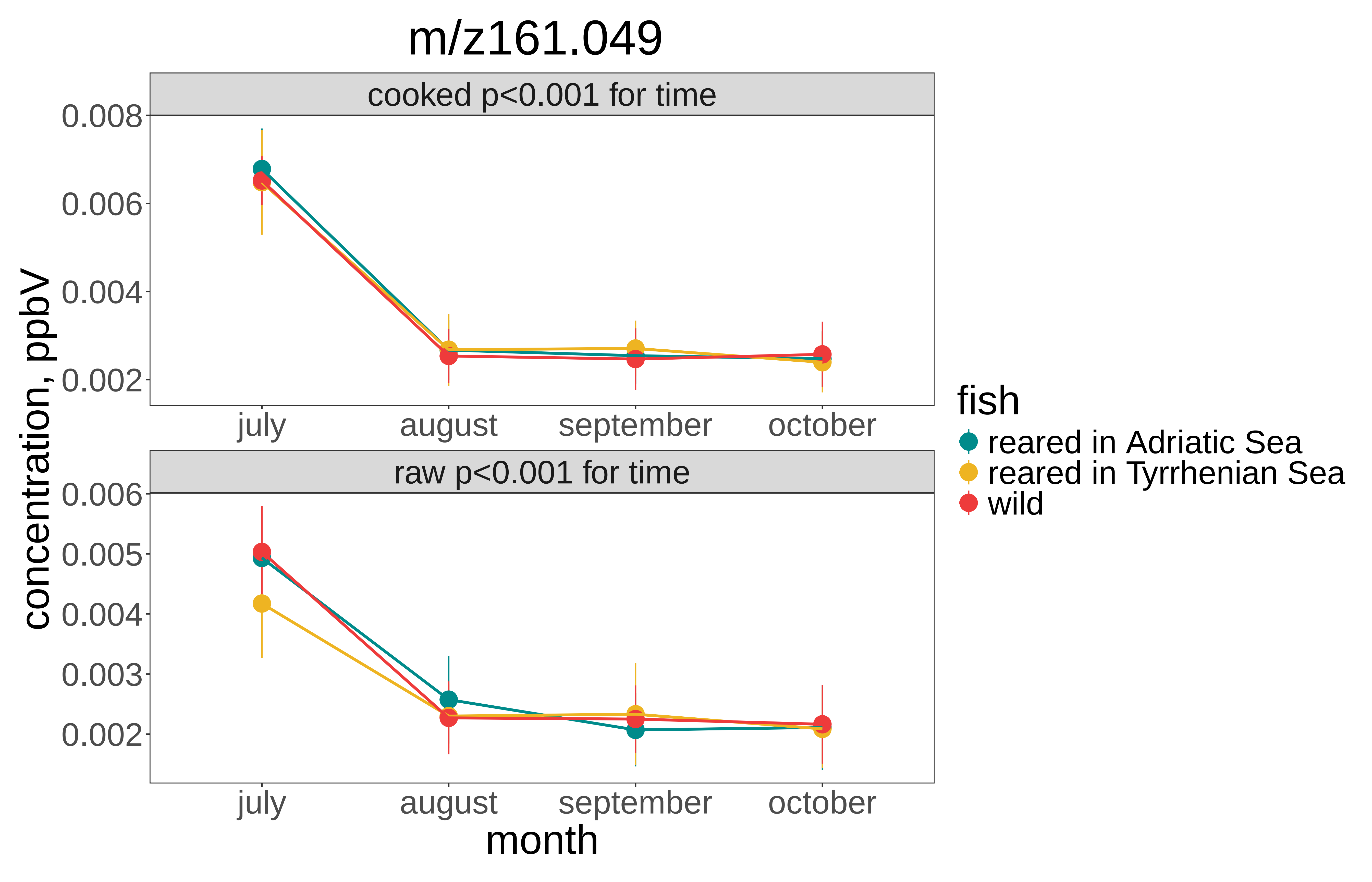
m/z161.049
cooked p<0.001 for time
raw p<0.001 for time
concentration, ppbV
0.008
0.006
0.004
0.002
0.006
0.005
0.004
0.003
0.002
july
august
september
october
month
fish
reared in Adriatic Sea
reared in Tyrrhenian Sea
wild

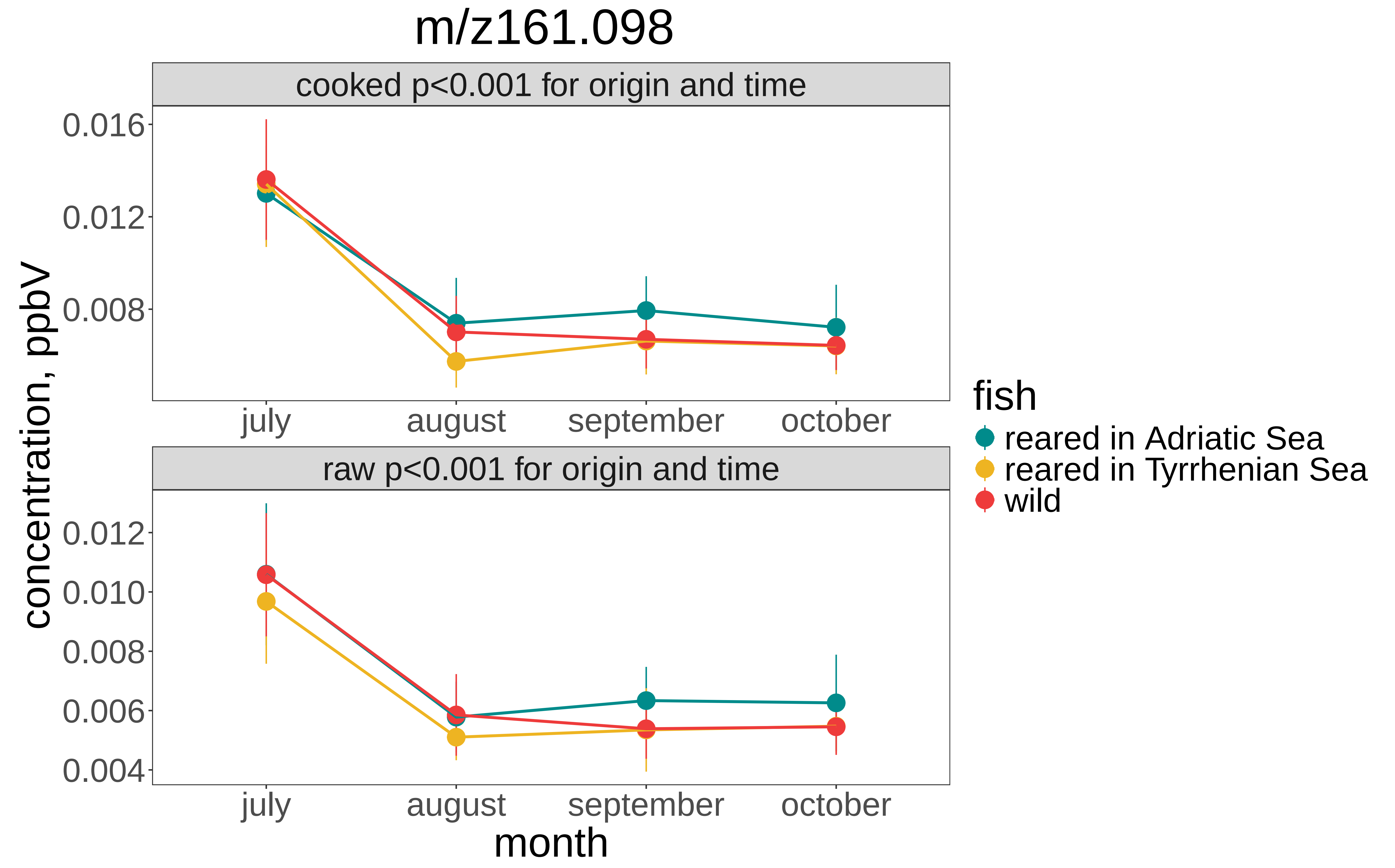

m/z161.098
cooked p<0.001 for origin and time
0.016
0.012
0.008
july
august
september
october
raw p<0.001 for origin and time
0.012
0.010
0.008
0.006
0.004
july
august
september
october
month
concentration, ppbV
fish
reared in Adriatic Sea
reared in Tyrrhenian Sea
wild

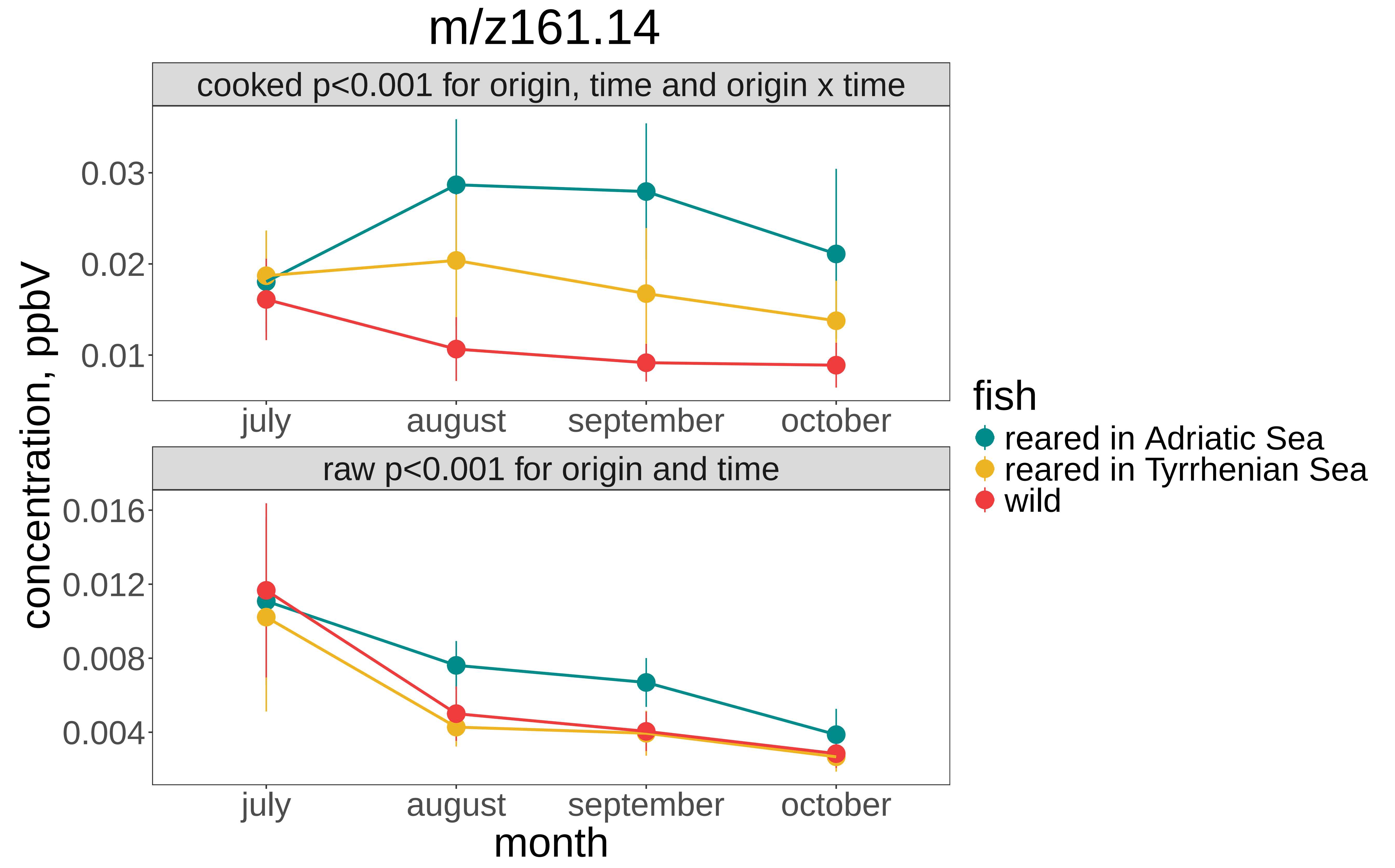
m/z161.14
cooked p<0.001 for origin, time and origin x time
0.03
0.02
0.01
july
august
september
october
raw p<0.001 for origin and time
0.016
0.012
0.008
0.004
july
august
september
october
month
concentration, ppbV
fish
reared in Adriatic Sea
reared in Tyrrhenian Sea
wild

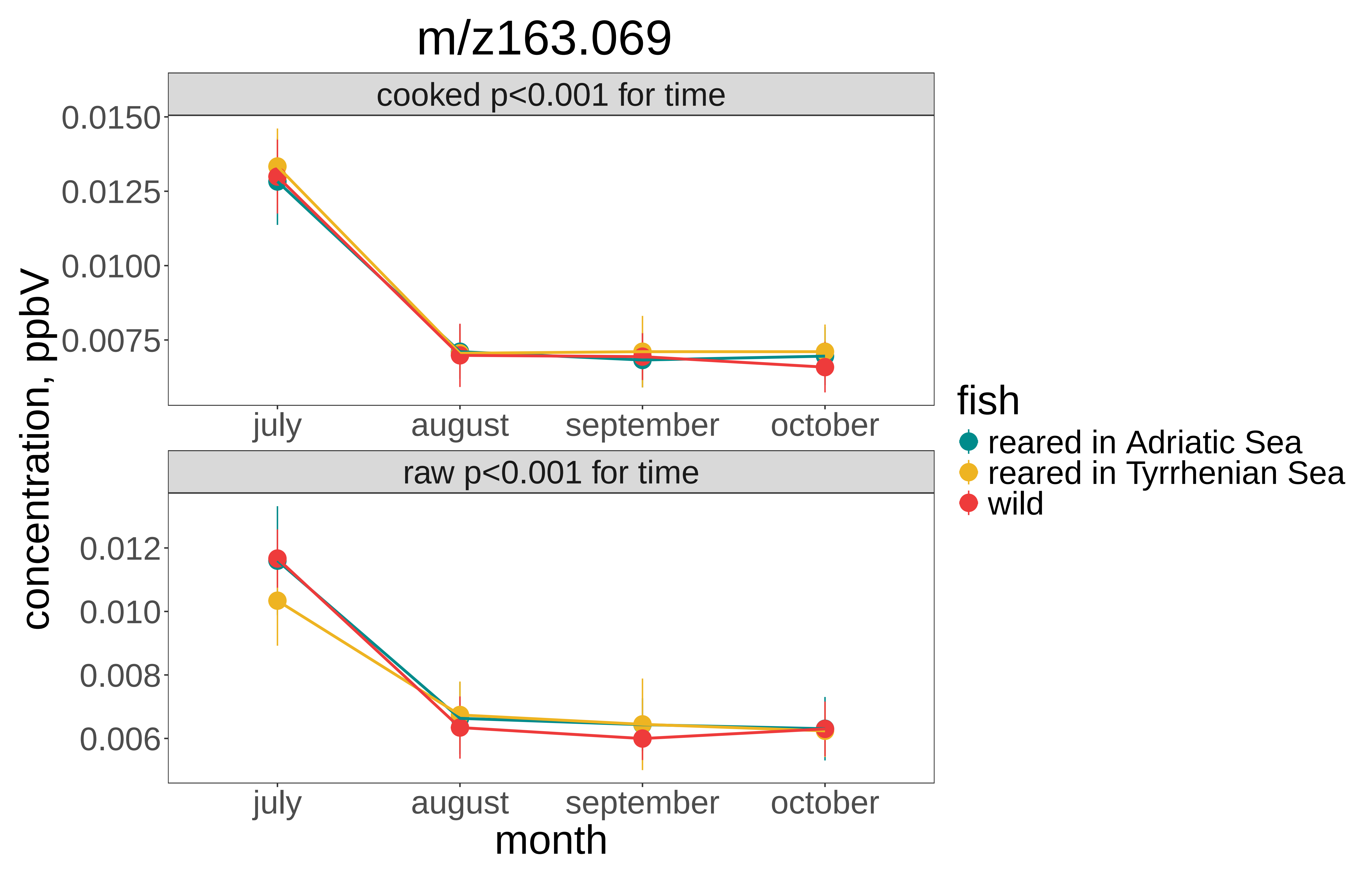
m/z163.069
cooked p<0.001 for time
0.0150
0.0125
0.0100
0.0075
july
august
september
october
raw p<0.001 for time
0.012
0.010
0.008
0.006
july
august
september
october
concentration, ppbV
month
fish
reared in Adriatic Sea
reared in Tyrrhenian Sea
wild

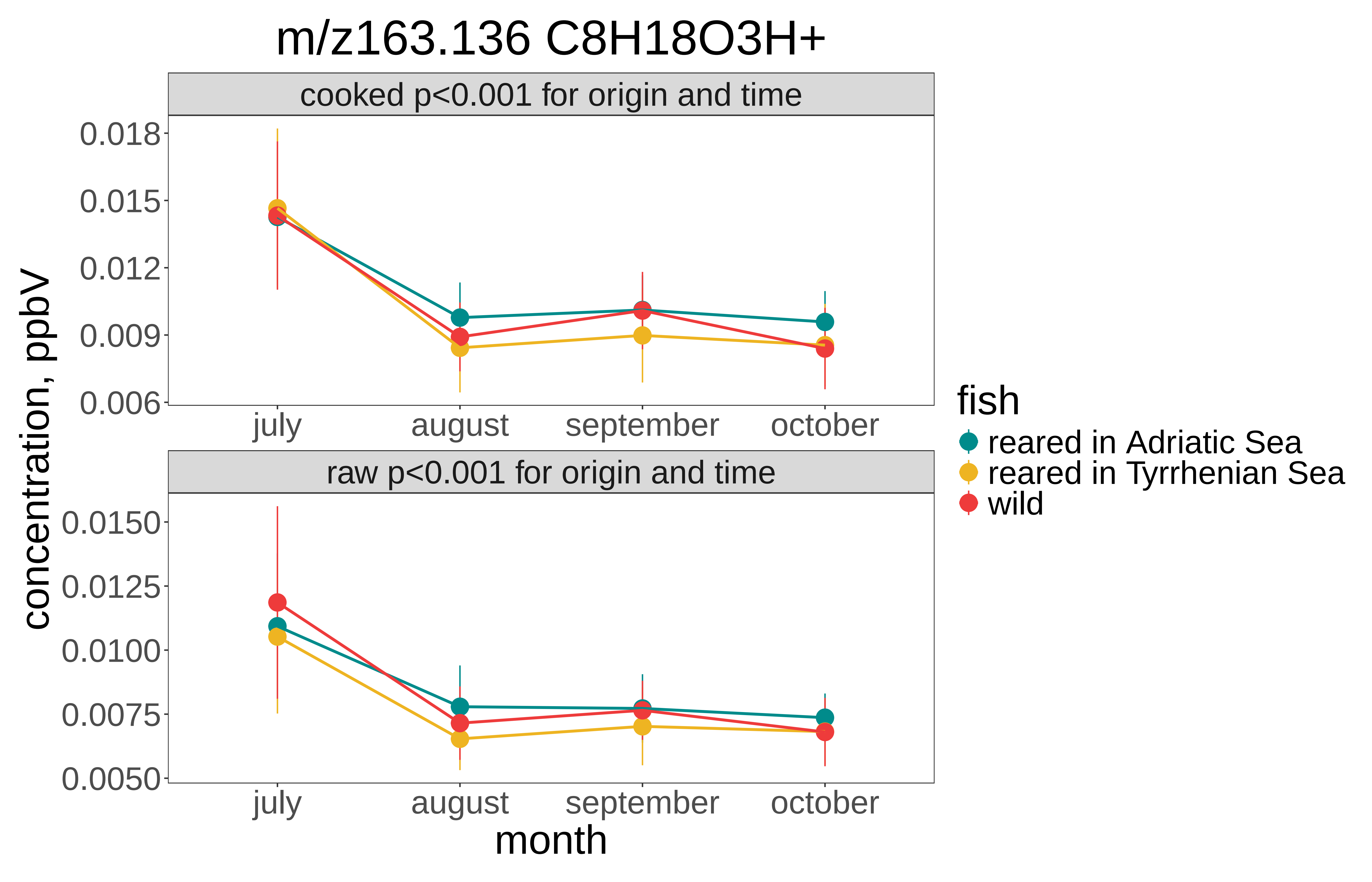
m/z163.136 C8H18O3H+
cooked p<0.001 for origin and time
raw p<0.001 for origin and time
concentration, ppbV
0.018
0.015
0.012
0.009
0.006
0.0150
0.0125
0.0100
0.0075
0.0050
july
august
september
october
month
fish
reared in Adriatic Sea
reared in Tyrrhenian Sea
wild

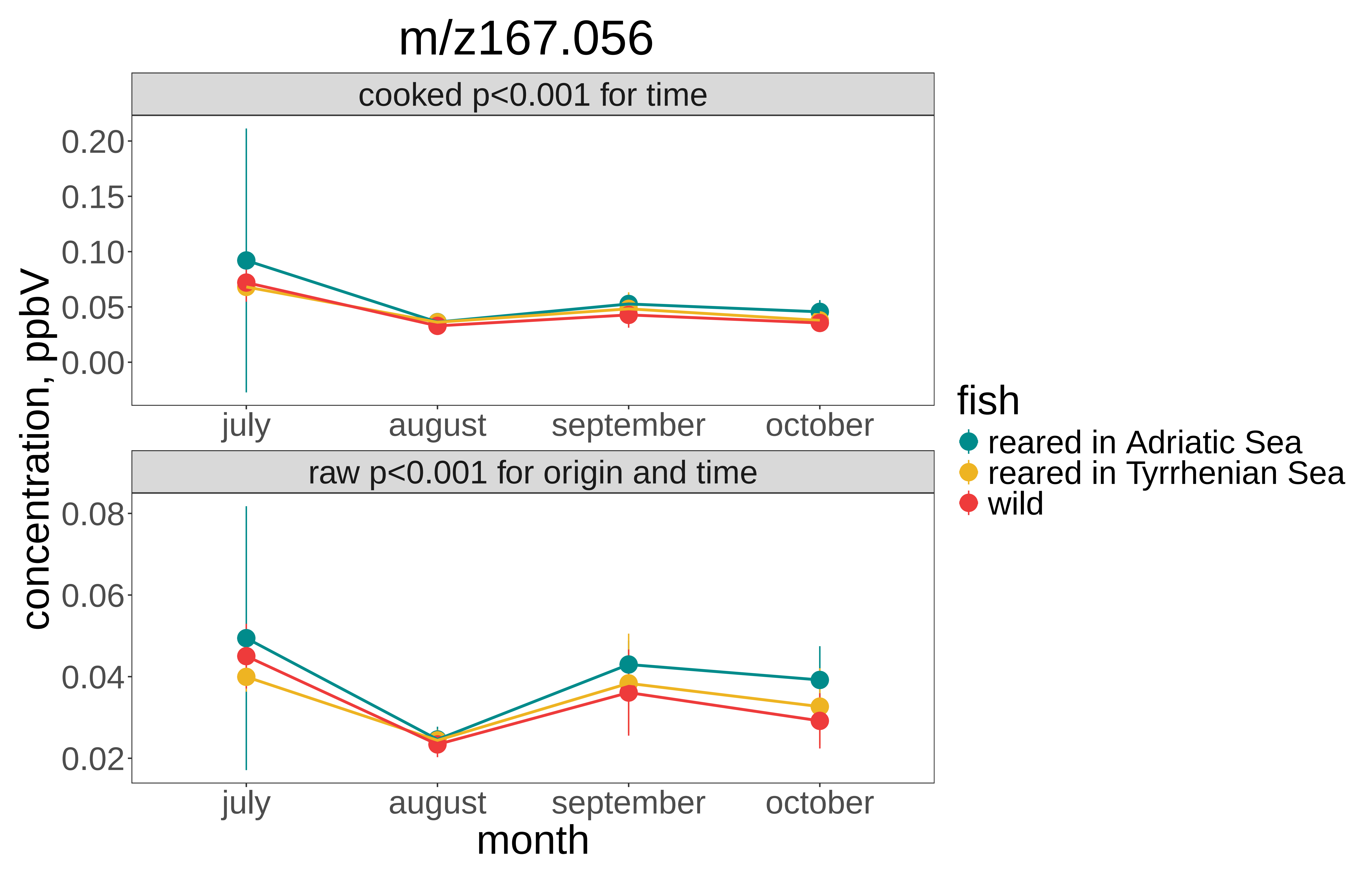
m/z167.056
cooked p<0.001 for time
raw p<0.001 for origin and time
concentration, ppbV
0.20
0.15
0.10
0.05
0.00
0.08
0.06
0.04
0.02
july
august
september
october
month
fish
reared in Adriatic Sea
reared in Tyrrhenian Sea
wild

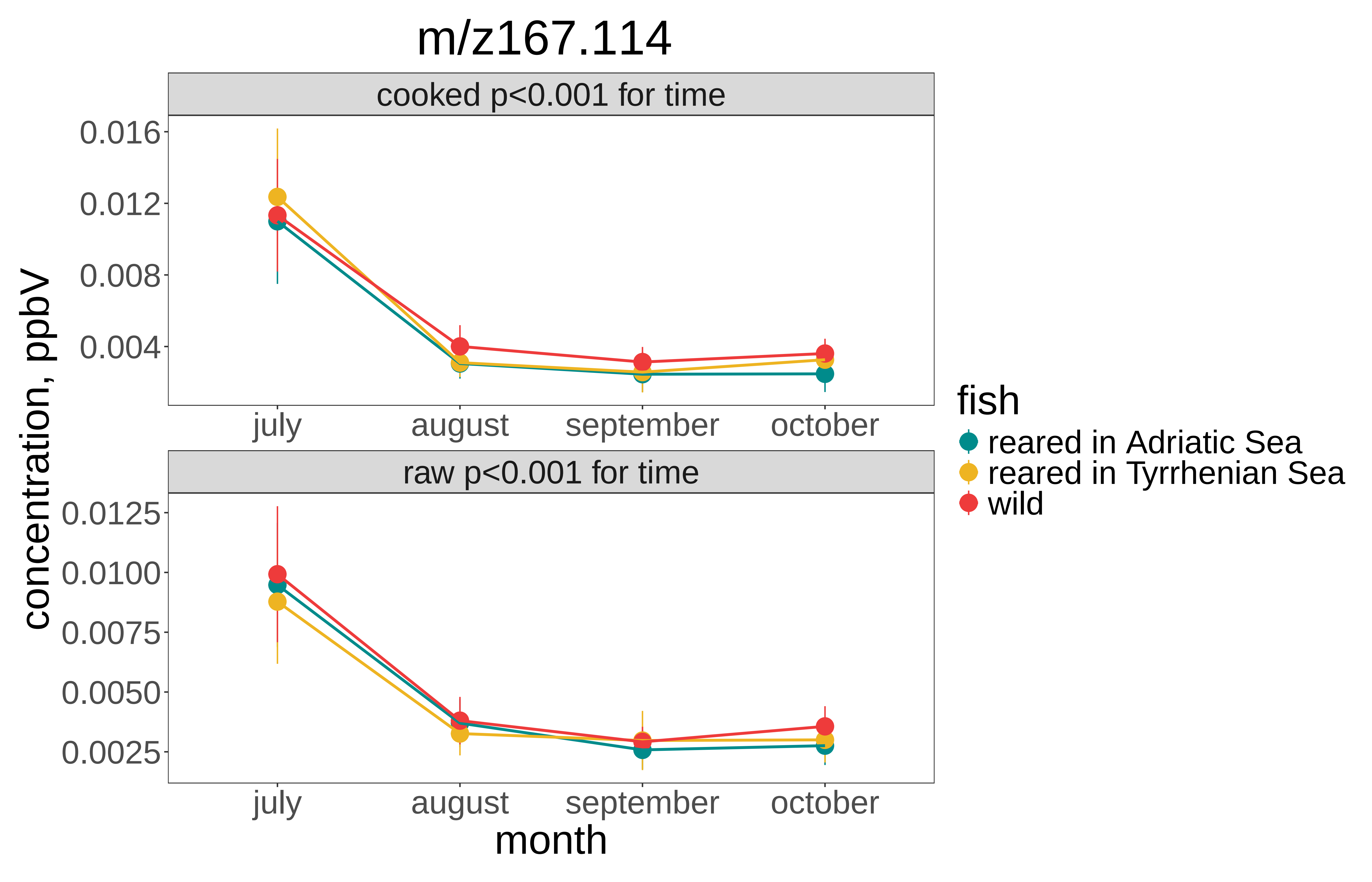
m/z167.114
cooked p<0.001 for time
0.016
0.012
0.008
0.004
july
august
september
october
raw p<0.001 for time
0.0125
0.0100
0.0075
0.0050
0.0025
july
august
september
october
month
concentration, ppbV
fish
reared in Adriatic Sea
reared in Tyrrhenian Sea
wild

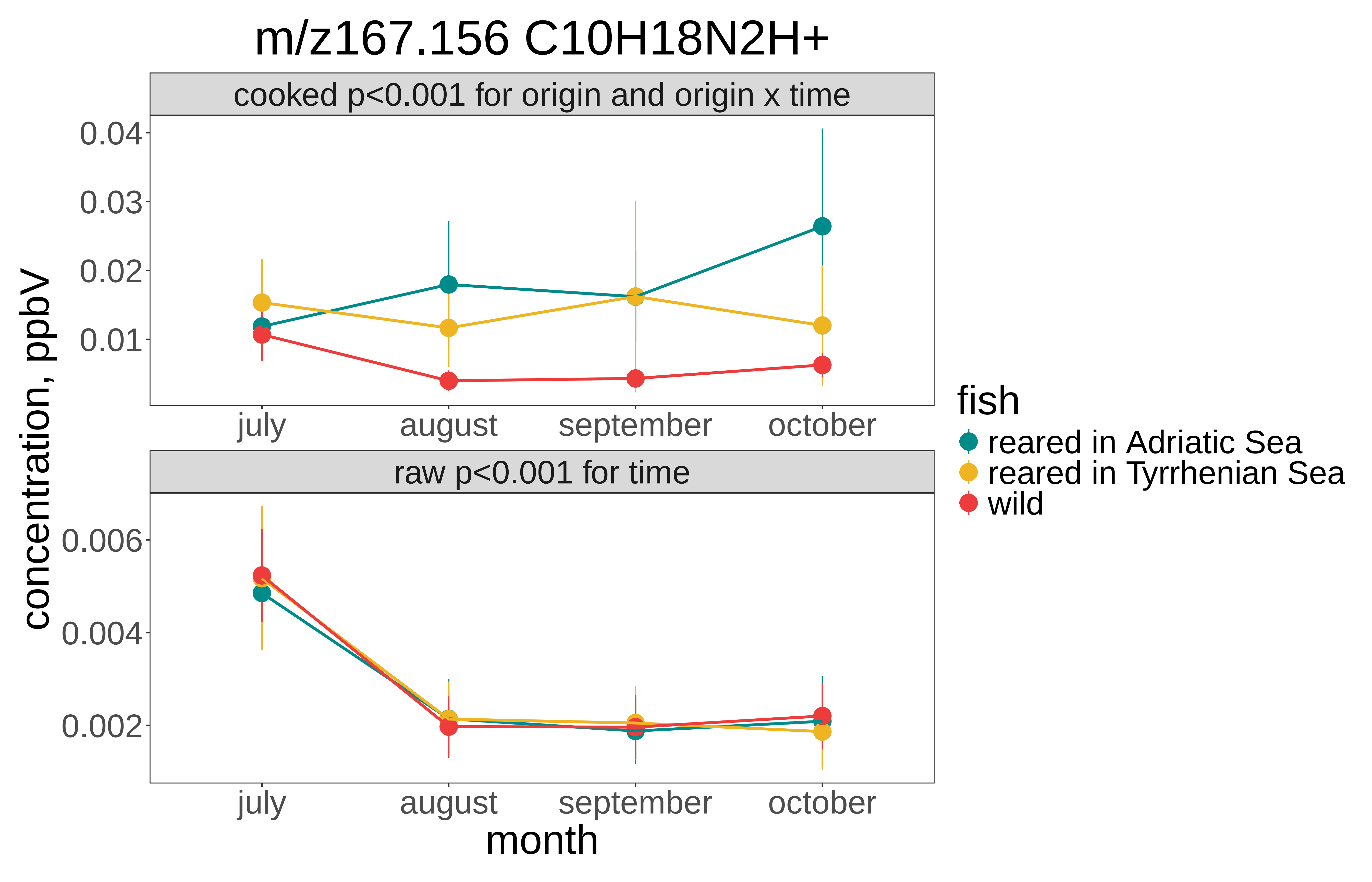

m/z167.156 C10H18N2H+
cooked p<0.001 for origin and origin x time
raw p<0.001 for time
concentration, ppbV
0.04
0.03
0.02
0.01
0.006
0.004
0.002
july
august
september
october
month
fish
reared in Adriatic Sea
reared in Tyrrhenian Sea
wild

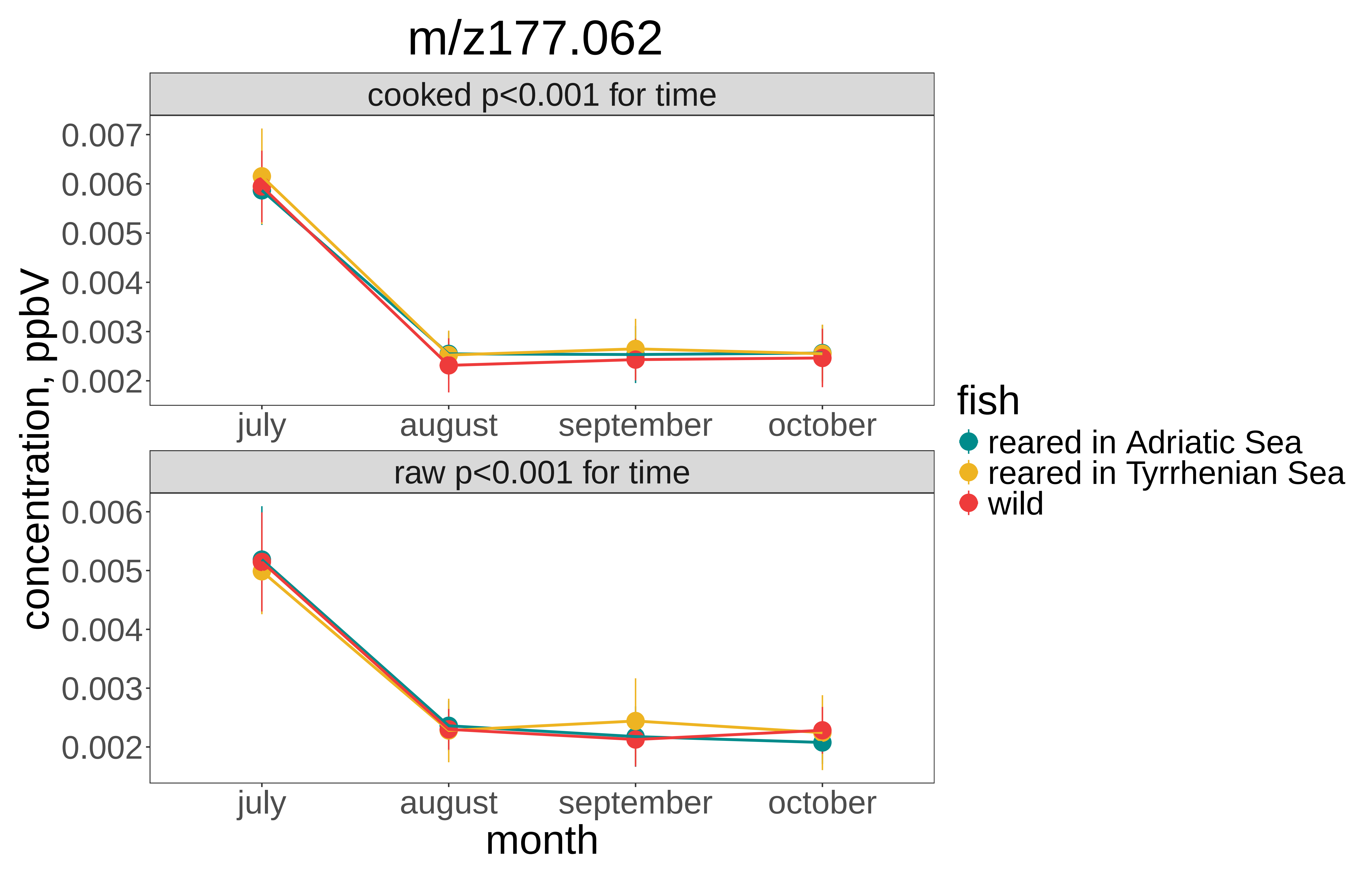
m/z177.062
cooked p<0.001 for time
raw p<0.001 for time
0.007
0.006
0.005
0.004
0.003
0.002
july
august
september
october
concentration, ppbV
month
fish
reared in Adriatic Sea
reared in Tyrrhenian Sea
wild

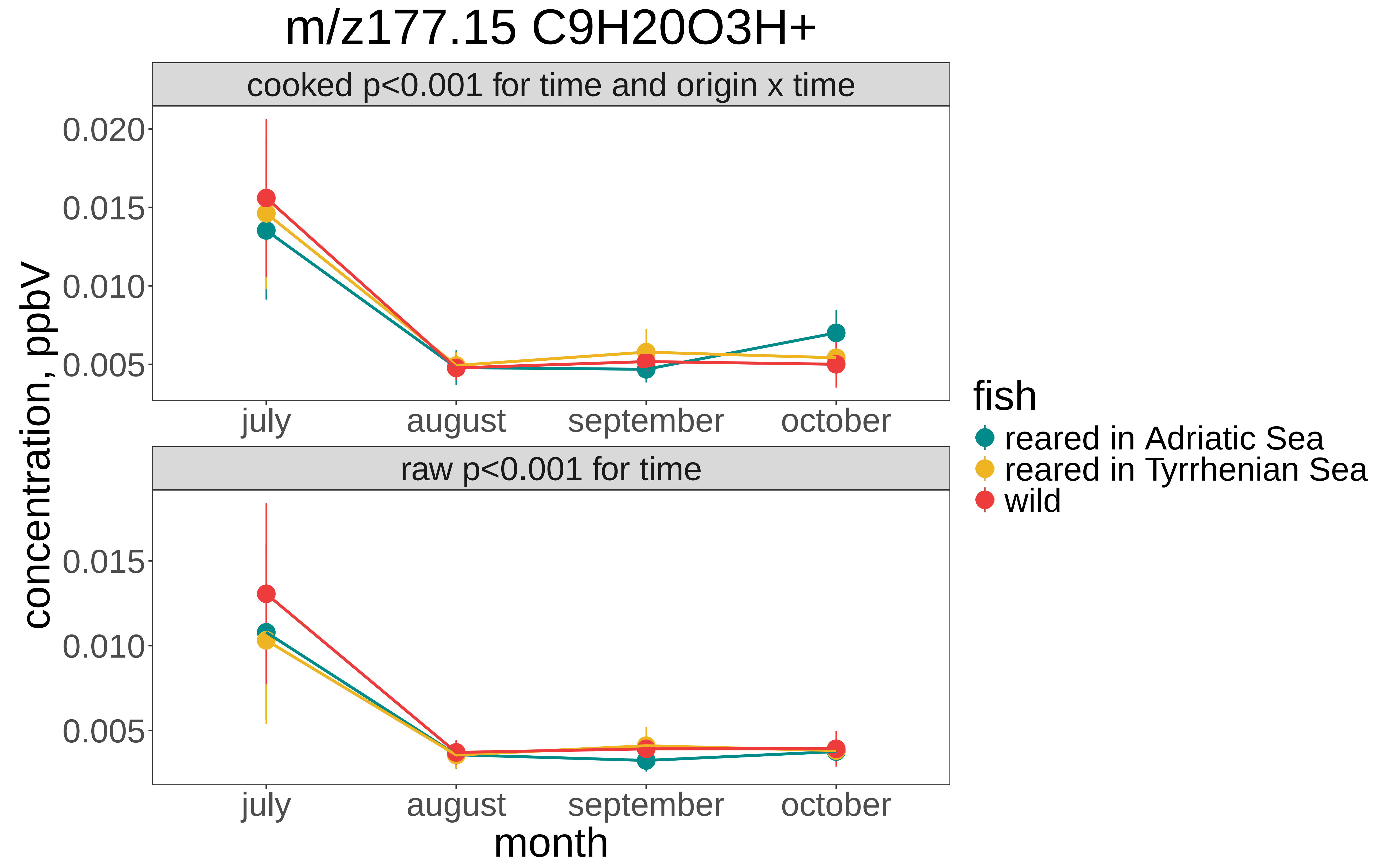
m/z177.15 C9H20O3H+
cooked p<0.001 for time and origin x time
raw p<0.001 for time
concentration, ppbV
0.020
0.015
0.010
0.005
july
august
september
october
month
fish
reared in Adriatic Sea
reared in Tyrrhenian Sea
wild

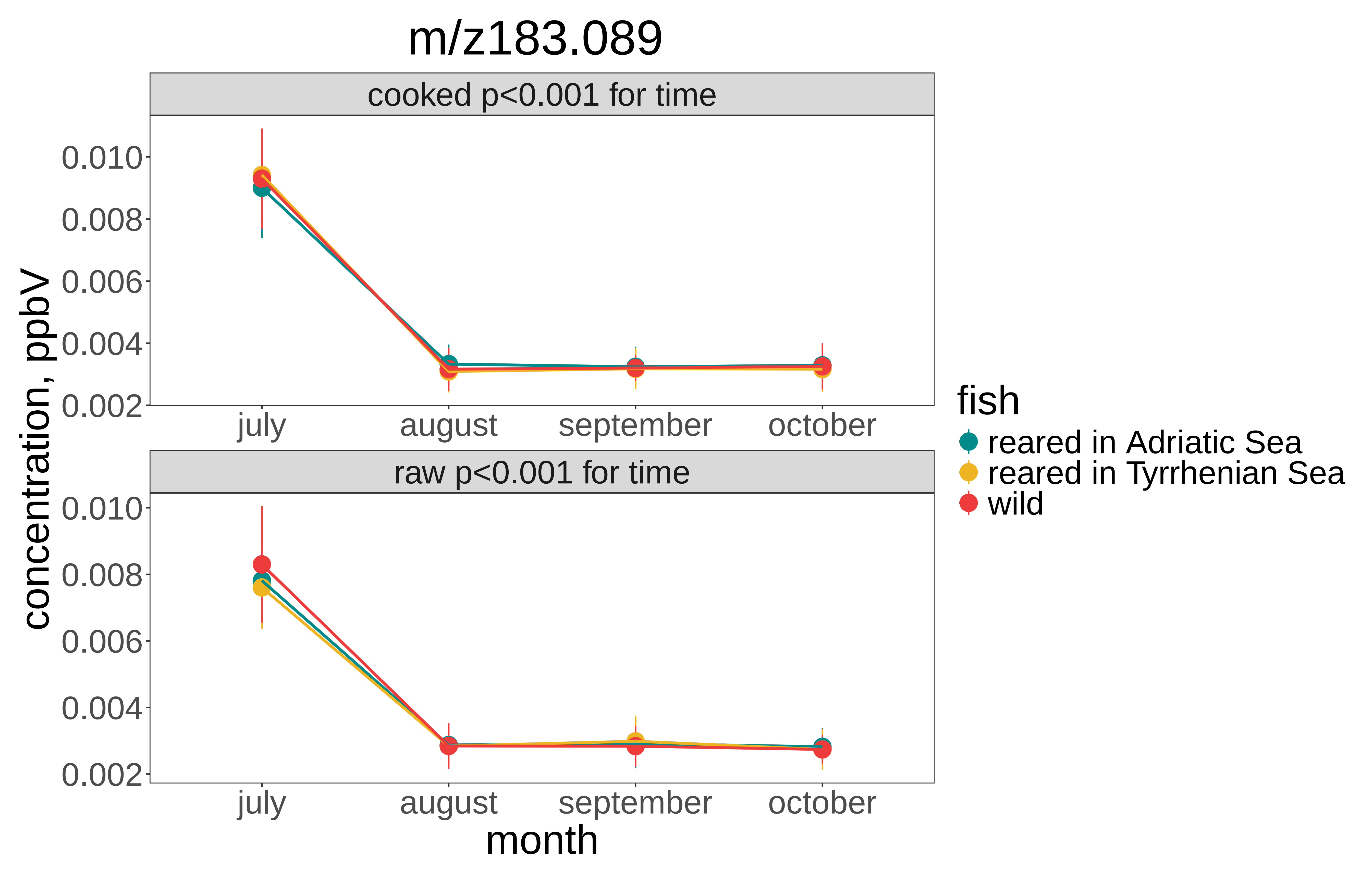
m/z183.089
cooked p<0.001 for time
raw p<0.001 for time
concentration, ppbV
0.010
0.008
0.006
0.004
0.002
july
august
september
october
month
fish
reared in Adriatic Sea
reared in Tyrrhenian Sea
wild

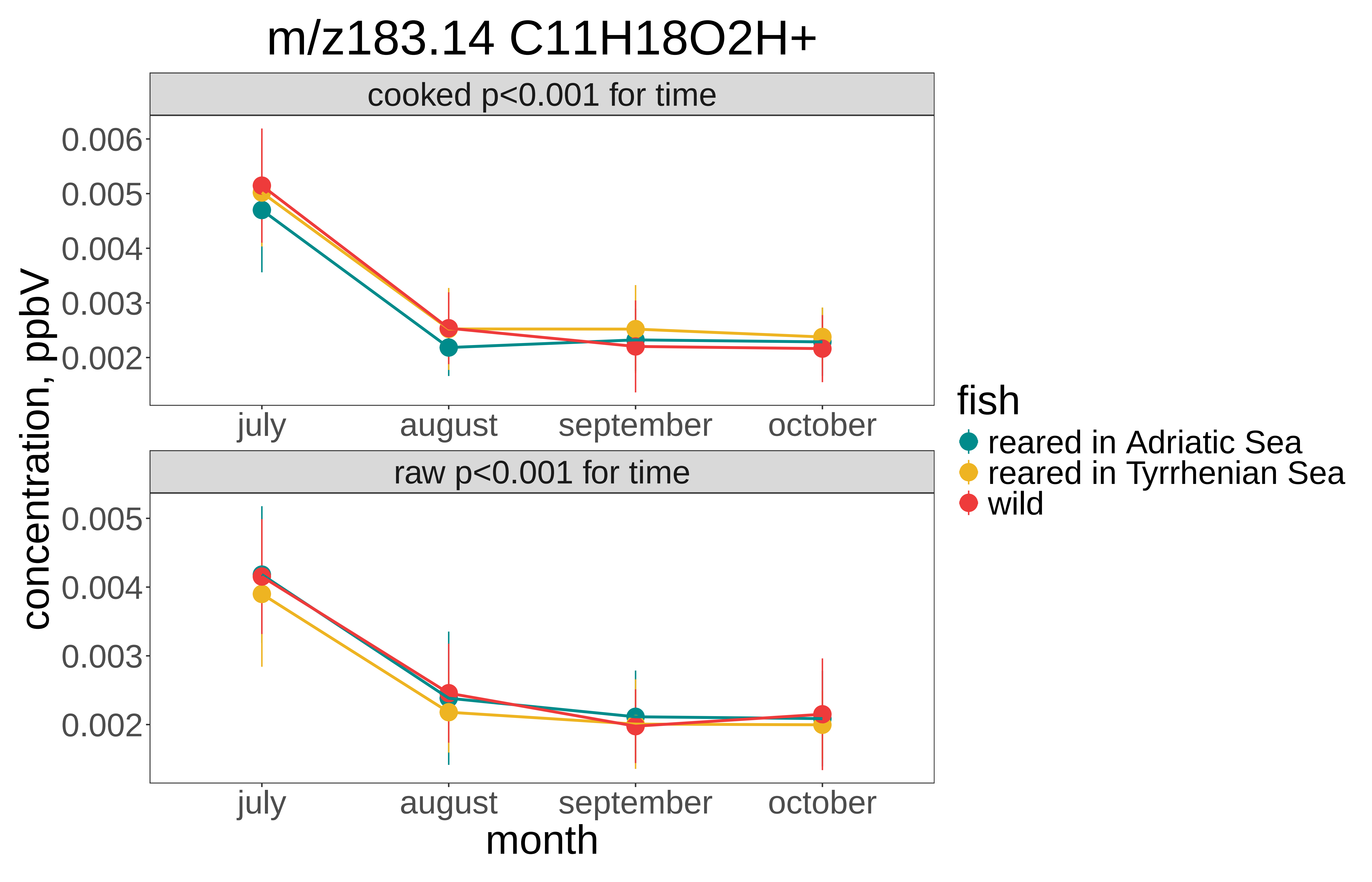

m/z183.14 C11H18O2H+
cooked p<0.001 for time
0.006
0.005
0.004
0.003
0.002
july
august
september
october
raw p<0.001 for time
0.005
0.004
0.003
0.002
july
august
september
october
concentration, ppbV
month
fish
reared in Adriatic Sea
reared in Tyrrhenian Sea
wild

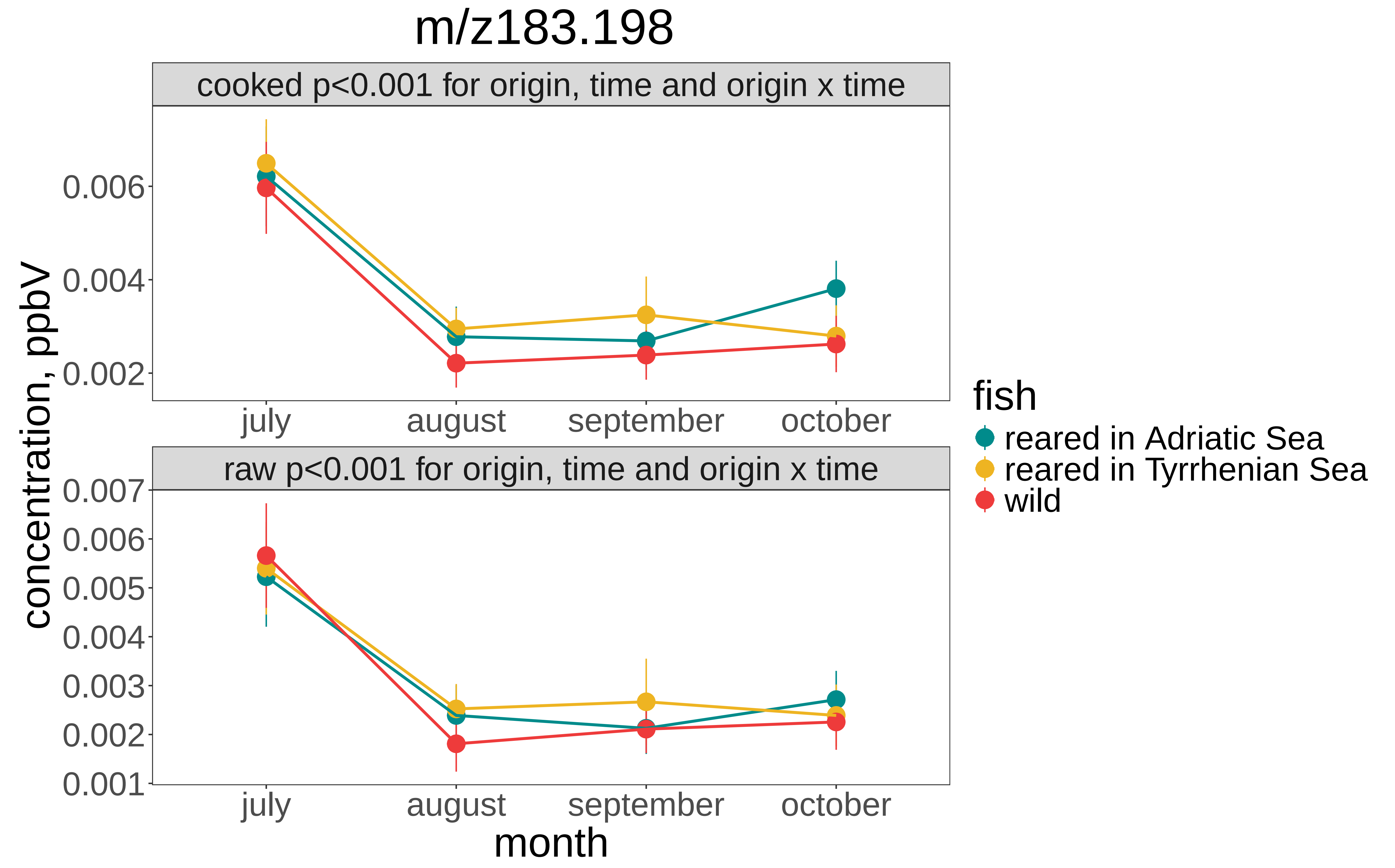
m/z183.198
cooked p<0.001 for origin, time and origin x time
raw p<0.001 for origin, time and origin x time
concentration, ppbV
0.006
0.004
0.002
0.007
0.006
0.005
0.004
0.003
0.002
0.001
july
august
september
october
month
fish
reared in Adriatic Sea
reared in Tyrrhenian Sea
wild
