# Supplementary figures and images for: PTR-ToF-MS VOC Profiling of Raw and Cooked Gilthead Sea Bream Fillet (Sparus aurata): Effect of Rearing System, Season, and Geographical Origin
Source: Molecules. 2025 Jan 18;30(2):402. doi: 10.3390/molecules30020402 (PMC11767258; doi:10.3390/molecules30020402)

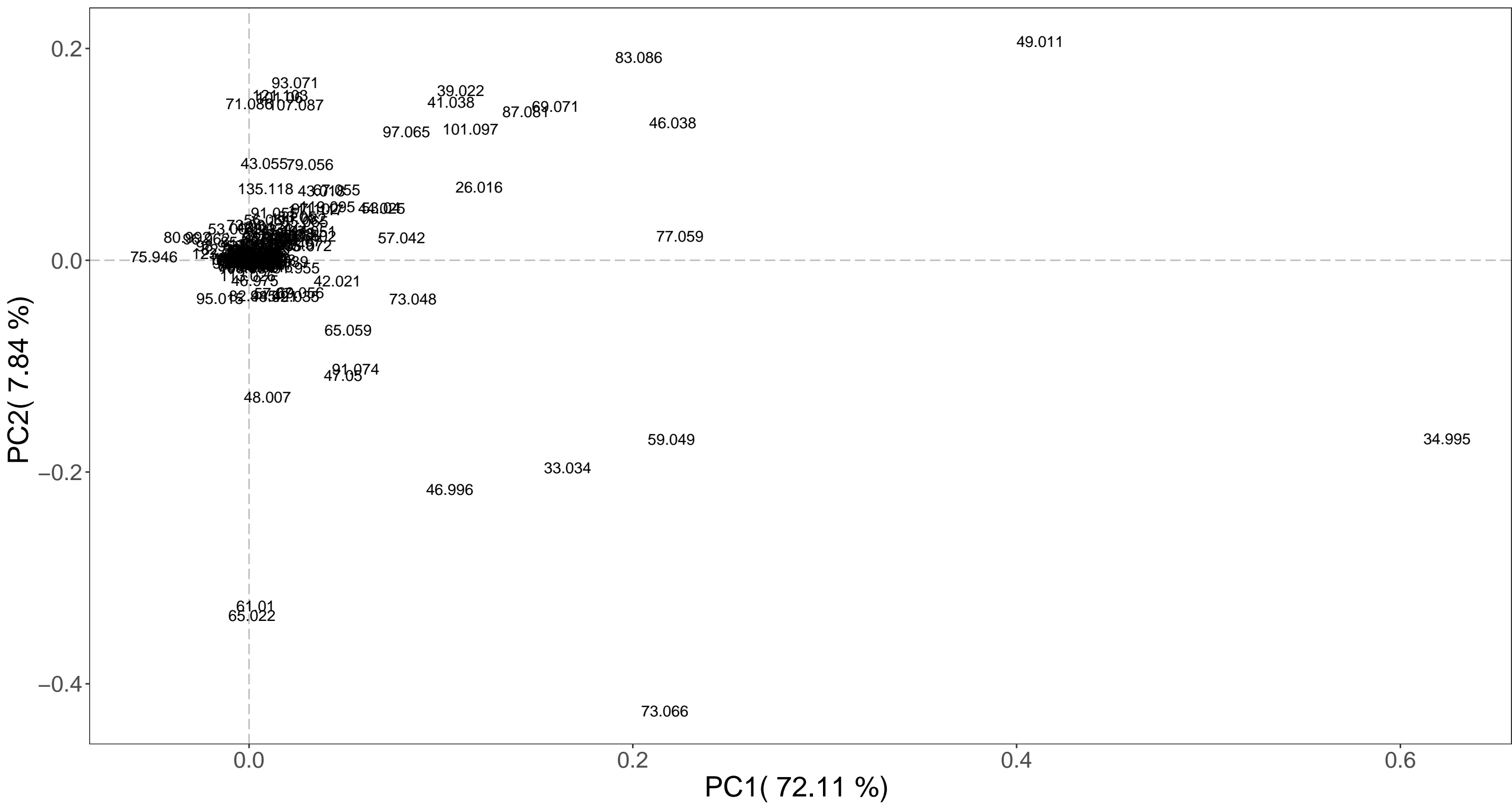

Supplement: Supplementary file 1 [file molecules-30-00402-s001.zip › Figure_S2.pdf]
