# Supplementary material for: PTR-ToF-MS VOC Profiling of Raw and Cooked Gilthead Sea Bream Fillet (Sparus aurata): Effect of Rearing System, Season, and Geographical Origin
Source: Molecules. 2025 Jan 18;30(2):402. doi: 10.3390/molecules30020402 (PMC11767258; doi:10.3390/molecules30020402)
Supplement: Supplementary file 1 [file Figure_S3.pdf › Figure_S3.pdf]

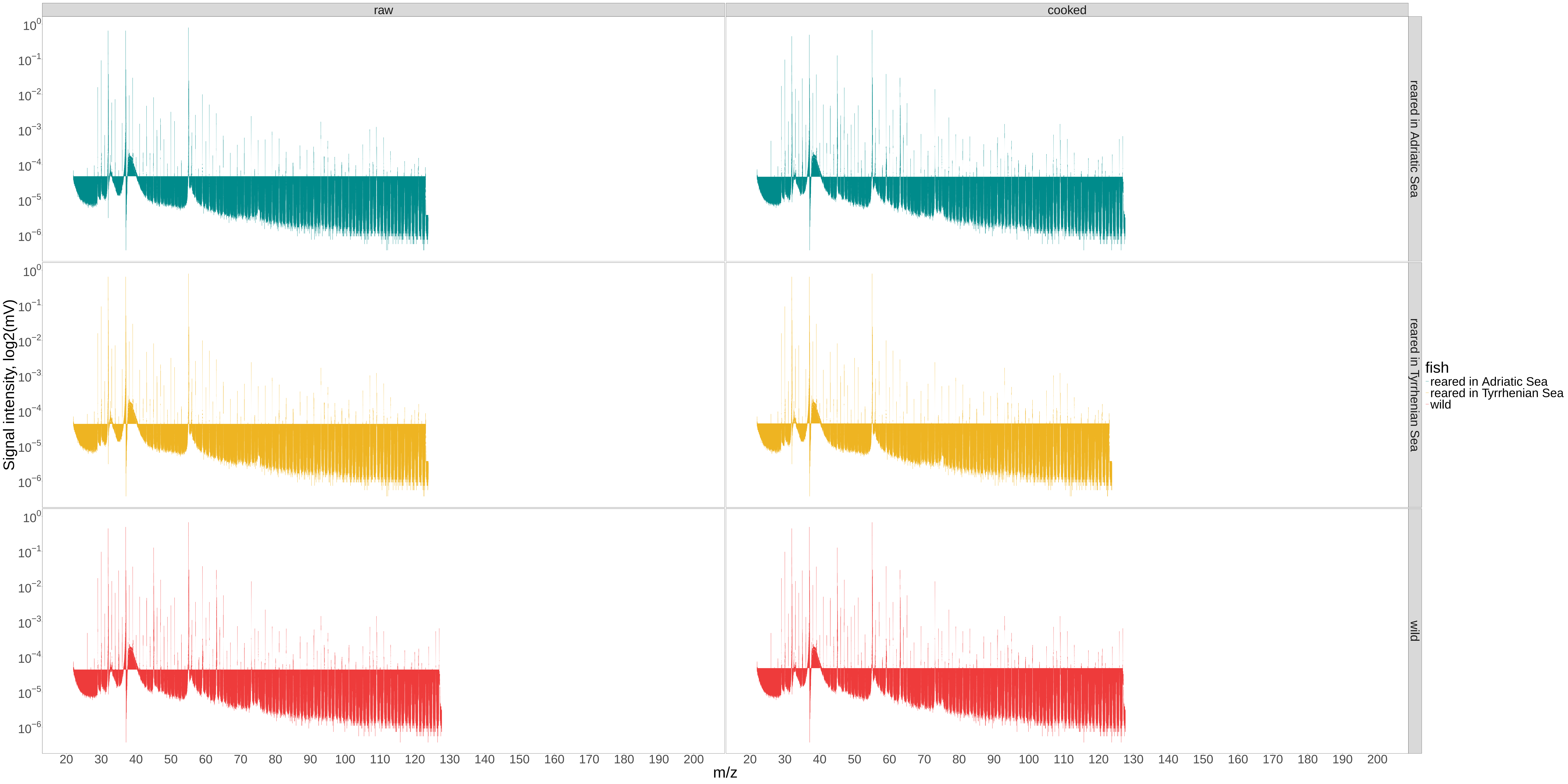
raw
cooked
reared in Adriatic Sea
reared in Tyrrhenian Sea
wild
Signal intensity, log2(mV)
10^{0}
10^{-1}
10^{-2}
10^{-3}
10^{-4}
10^{-5}
10^{-6}
20 30 40 50 60 70 80 90 100 110 120 130 140 150 160 170 180 190 200
m/z
fish
reared in Adriatic Sea
reared in Tyrrhenian Sea
wild
